# Ring-opening carbonyl–olefin metathesis of norbornenes†

**DOI:** 10.1039/d0sc02243h

**Published:** 2020-07-01

**Authors:** Janis Jermaks, Phong K. Quach, Zara M. Seibel, Julien Pomarole, Tristan H. Lambert

**Affiliations:** Department of Chemistry and Chemical Biology, Cornell University Ithaca New York 14853 USA thl36@cornell.edu; Department of Chemistry, Columbia University New York New York 10025 USA

## Abstract

A computational and experimental study of the hydrazine-catalyzed ring-opening carbonyl–olefin metathesis of norbornenes is described. Detailed theoretical investigation of the energetic landscape for the full reaction pathway with six different hydrazines revealed several crucial aspects for the design of next-generation hydrazine catalysts. This study indicated that a [2.2.2]-bicyclic hydrazine should offer substantially increased reactivity *versus* the previously reported [2.2.1]-hydrazine due to a lowered activation barrier for the rate-determining cycloreversion step, a prediction which was verified experimentally. Optimized conditions for both cycloaddition and cycloreversion steps were identified, and a brief substrate scope study for each was conducted. A complication for catalysis was found to be the slow hydrolysis of the ring-opened hydrazonium intermediates, which were shown to suffer from a competitive and irreversible cycloaddition with a second equivalent of norbornene. This problem was overcome by the strategic incorporation of a bridgehead methyl group on the norbornene ring, leading to the first demonstrated catalytic carbonyl–olefin metathesis of norbornene rings.

## Abstract 2

A computational and experimental study has uncovered a second generation hydrazine that enables the catalytic ring-opening carbonyl–olefin metathesis of norbornenes.

## Graphical abstract

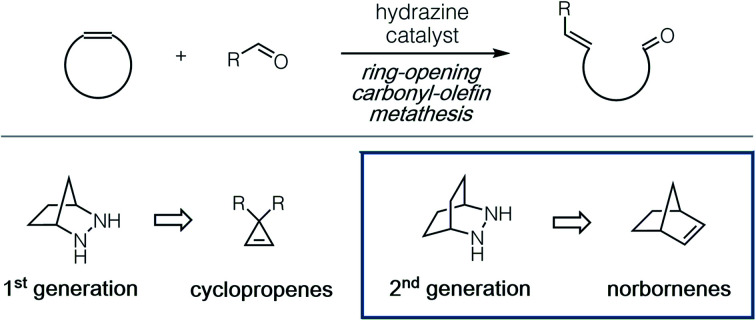

## 1 Introduction

**Fig. 1:**
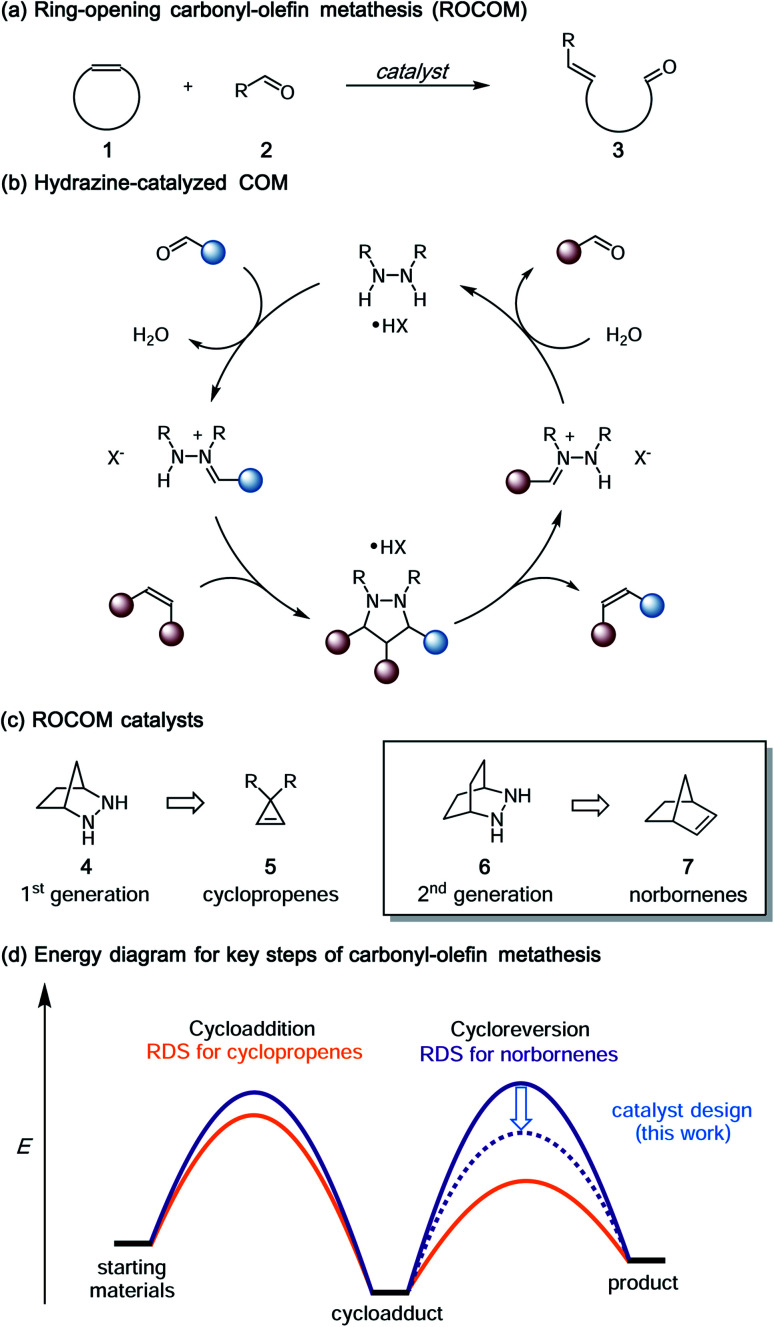
(a) Generalized ring-opening carbonyl–olefin metathesis, (b) catalytic cycle for hydrazine-catalyzed COM, (c) hydrazine-based ROCOM catalysts, (d) stylized energy diagram for ROCOM of cyclopropenes and norbornenes.

Inspired in large part by the great success of catalytic olefin metathesis,^1^ there has been increasing interest in the development of other catalytic metathetical reactions.^2,3^ In particular, carbonyl–olefin metathesis (COM) has the potential to enable a number of important new catalytic transformations,^4–7^ including the ring-opening carbonyl–olefin metathesis (ROCOM) of cyclic olefins to generate alkenyl aldehydes (Fig. 1a). As such, catalytic COM has received increasing attention in recent years. At the present time, however, this area is still in its infancy, and the generality of available catalytic platforms falls far short of what would be needed to realize many of the potential applications of this reaction. Recent exciting developments in the use of organocatalysts,^8,9^ Lewis acids,^10–21^ and Brønsted acids^22,23^ notwithstanding, this area requires breakthrough advances in catalyst and strategy design if it is to approach the levels of utility realized by other mature catalytic processes.

Toward this goal, we had previously reported the first catalytic strategy for carbonyl–olefin metathesis.^8,9^ This approach was based on the concept of utilizing reversible, locally symmetric, 1,3-dipolar cycloadditions^24^ to circumvent some of the difficulties presented by [2 + 2] cycloadditions of carbonyls and alkenes (Fig. 1b). We implemented this conceptual design with azomethine imine-type cycloadditions^25,26^ using the simple bicyclic hydrazine **4** as catalyst (Fig. 1c).^27^ With this approach, we were able to realize the catalytic ring-opening carbonyl–olefin metathesis (ROCOM) of cyclopropenes.^8^ Although we have recently expanded this chemistry to ring-closing carbonyl–olefin metathesis (RCCOM) reactions as well,^9^ for ring-opening reactions this method was effectively limited to cyclopropenes, the high strain of which^28,29^ enabled facile cycloreversion of what are otherwise quite stable cycloadducts. Indeed, computational evaluation of a variety of olefins revealed that the activation energy of cycloreversion for less strained olefins was typically >33 kcal mol^−1^.^30^ Thus, potentially useful reactions such as the ROCOM of norbornenes^31^ were not viable using our first-generation catalyst/reaction conditions. In fact, because such substrates do not readily support the formation of carbocation intermediates, they are also not amenable to acid catalyzed approaches either. Thus, norbornenes represent an important forefront challenge for carbonyl–olefin metathesis.

Despite the fact that our original conditions were not productive with norbornene, we reasoned that with sufficient energy input the hydrazine-mediated ROCOM of this substrate should be attainable, as long as undesired side reactions were not to render the process grossly inefficient. More usefully, we posited that modifications to the hydrazine structure should allow for lowering of the cycloreversion activation barrier to the point that a useful generality of scope at practicable reaction temperatures could be realized (Fig. 1d). On the other hand, relatively little work has been done regarding [3 + 2]-cycloreversions generally,^32^ or azomethine imine cycloreversions specifically,^33,34^ and so it was not clear to what extent such an undertaking would be successful. Nevertheless, we felt that gaining a deeper understanding of the catalyst and condition parameters that control the efficiency of hydrazine-catalyzed COM held great promise to make advancements in this area. In this article, we describe the extension of the hydrazine-catalyzed carbonyl–olefin metathesis strategy to norbornene substrates. As key aspects of this work, we (1) demonstrate that computational modeling of hydrazine structure can be used as a predictive tool for reaction efficiency, (2) optimize conditions for both the cycloaddition and cycloreversion steps, (3) achieve hydrazine-catalyzed ROCOM reactions of norbornenes, and (4) reveal that the hydrolytic cleavage of hydrazonium intermediates is a crucial turnover-limiting step for these reactions.

## 2 Computational analysis

The carbonyl–olefin metathesis strategy described in this article is based on a [3 + 2] cycloaddition/cycloreversion sequence. In contrast to other double-bond metathesis paradigms, both steps of this design are thermally-allowed pericyclic reactions. Frontier molecular orbital (FMO) theory thus allows for the straightforward approximation and rationalization of reactivity for these steps by examining the HOMO–LUMO energy gap of the reactants as well as their symmetries according to the canonical Woodward–Hoffman rules.^35,36^

Previous work by Houk and our groups^30^ showed that the energetic barriers for the hydrazine-catalyzed ROCOM of olefins with less strain than cyclopropenes was substantial using the [2.2.1]-bicyclic hydrazine catalyst **4** from our previous study. It was thus imperative for us to identify alternative catalyst structures that would engender lower activation barriers and thereby extend the scope of this catalytic strategy to less-strained substrates such as norbornenes. Rather than take an empirical approach to such an undertaking, we aimed to develop a computational model for catalyst design that would offer reliable predictions of the relevant cycloaddition and cycloreversion energy barriers.

**Fig. 2:**
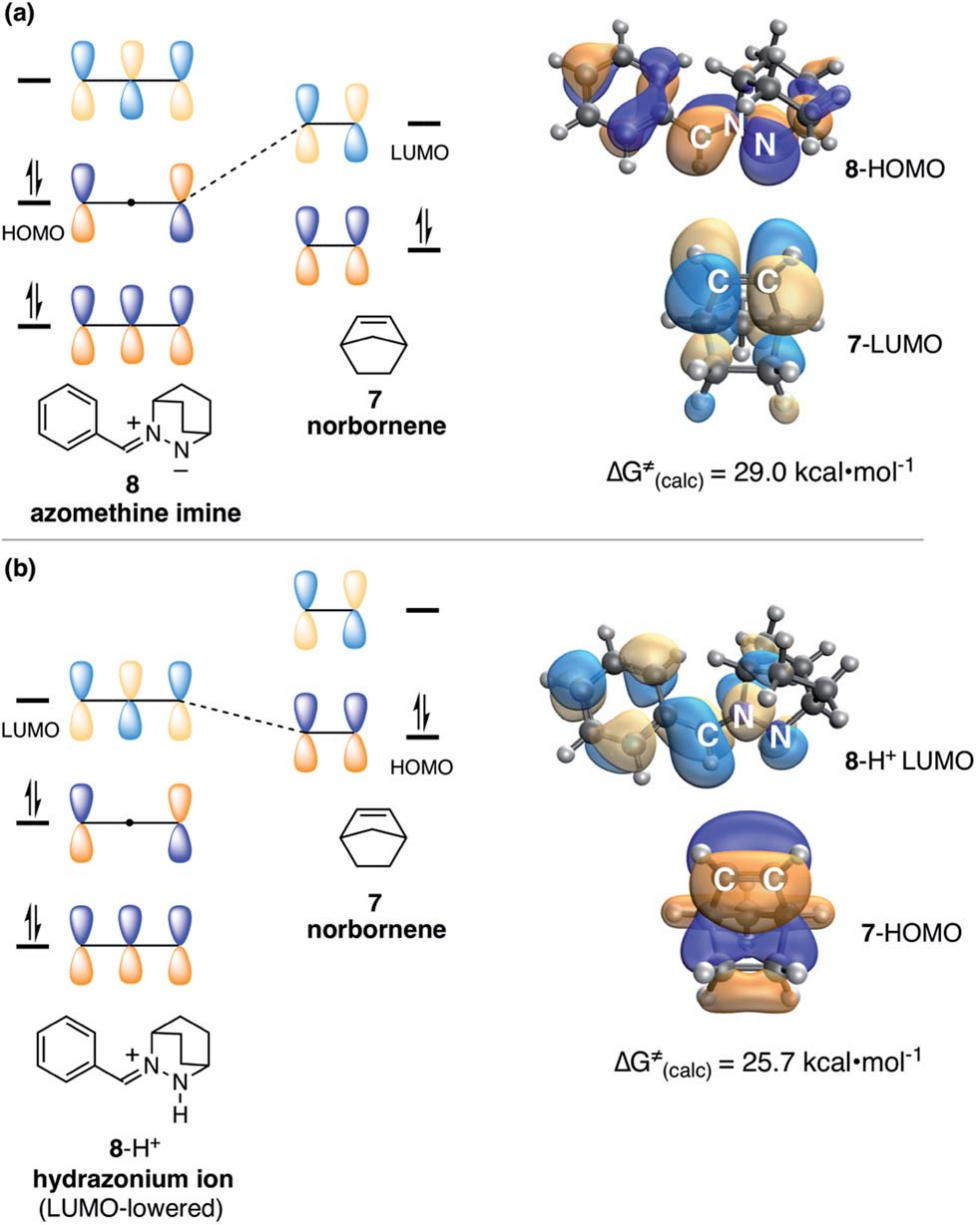
(a) FMO representation of HOMO–LUMO interaction for cycloaddition of azomethine imine **8** (HOMO) and norbornene (**7**) (LUMO). (b) FMO representation of HOMO–LUMO interaction for cycloaddition of hydrazonium ion **8**-H^+^ (LUMO) and norbornene (**7**) (HOMO).

Before describing those efforts, we first make an important point about the nature of the 1,3-dipolar cycloadditions/cycloreversions at issue in this work. The vast majority of azomethine-imine cycloadditions^37^ utilize hydrazine components bearing electron-withdrawing substituents on the formally anionic nitrogen atom. This structural feature supports the formation of the zwitterionic 1,3-dipole and dictates a normal electron-demand cycloaddition (Fig. 2a). That is to say, these reactions typically proceed *via* interaction of the HOMO of the azomethine imine and the LUMO of a dipolarophile, which thus usually possess electron-withdrawing groups. The interaction of a non-stabilized azomethine imine such as **8** with an electron-neutral olefin like norbornene, both of which possess high-lying FMOs, is much less favorable. Indeed, we have calculated that the cycloaddition between azomethine imine **8** and norbornene (**7**) also proceeds *via* the normal electron-demand pathway, with an activation barrier of 29.0 kcal mol^−1^.

**Fig. 3:**
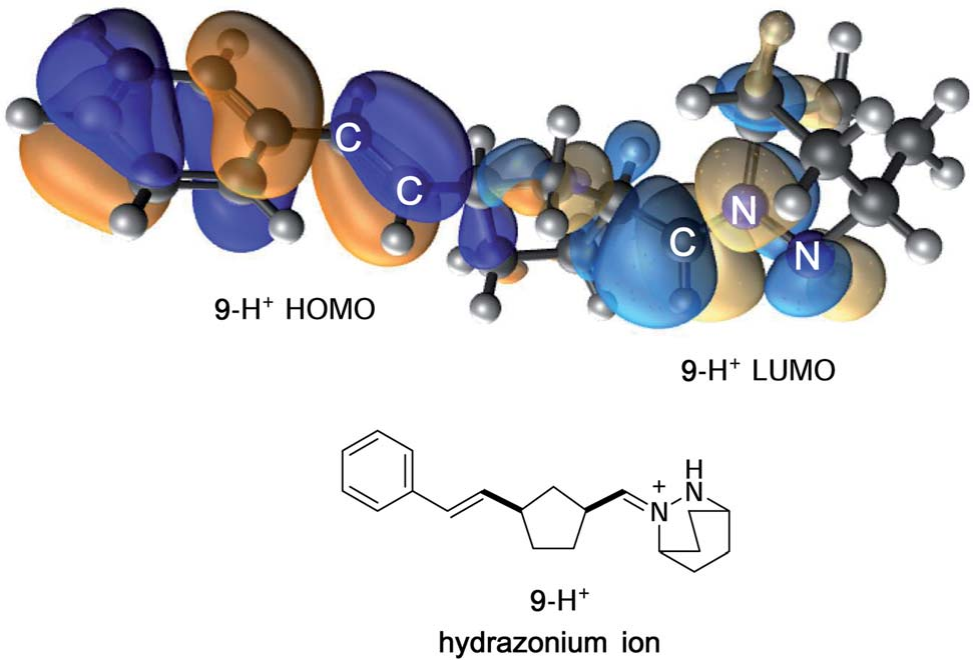
FMO representation of ring-opened hydrazonium ion **9**-H^+^, including styrenyl fragment (HOMO) and hydrazonium fragment (LUMO).

In contrast, protonation of the azomethine imine produces hydrazonium intermediate **8**-H^+^, which results in substantial lowering of the LUMO of this component. In this case, a more favorable inverse electron-demand interaction between the hydrazonium LUMO and norbornene HOMO leads to a calculated energy barrier of only 25.7 kcal mol^−1^. This finding aligns with our observation that the incorporation of a Brønsted acid greatly accelerates the cycloaddition step in the hydrazine-catalyzed COM process (*vide infra*). The same consideration was found to be true for cycloreversion, where inspection of the HOMO and LUMO of the ring-opened product revealed a similar inverse electron-demand reaction (Fig. 3). In accordance with this finding, the activation barrier for cycloreversion of protonated cycloadduct **32**-H^+^ to form intermediate hydrazonium **9**-H^+^ was calculated to be 33.5 kcal mol^−1^ (see below for details), whereas the corresponding barrier for unprotonated **32** was over 40 kcal mol^−1^. These results make clear why the incorporation of an acid co-catalyst was found to be necessary for the successful cycloadditions, cycloreversions, and catalytic reactions described in this article.

### 2.1 Computational analysis of norbornene ROCOM with [2.2.1]-hydrazine

**Fig. 4:**
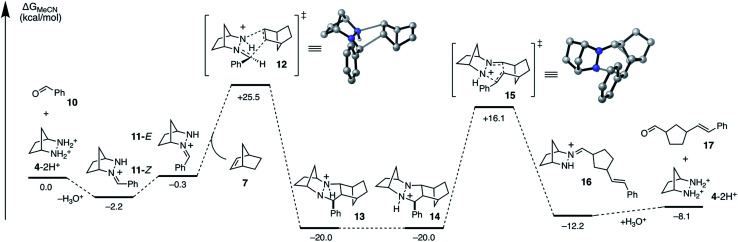
DFT calculated Gibbs free energies (M06-2X/6-31G(d)//M06-2X/6-311+G(2d,p)/PCM-(acetonitrile)) for ROCOM of norbornene and benzaldehyde with [2.2.1]-bicyclic hydrazine **4**.

To accelerate the search for more reactive hydrazines, we set as our first task to calculate the full energetic landscape for the ROCOM reaction of norbornene and benzaldehyde using [2.2.1]-hydrazine **4**. The results are shown in Fig. 4. Because a similar analysis was accomplished in our previous publication for a cyclopropene substrate, this undertaking allowed us to establish a reliable referent for the evaluation of alternative hydrazines. We conducted this density functional theory (DFT) study with the M06-2X functional,^38–40^ which has been shown to provide relatively accurate energetics for cycloadditions.^41,42^ The role of the counterion will remain undefined for now; however, for our initial calculations we assumed it to be an innocent spectator ion. The calculations were performed with acetonitrile as solvent to correlate computational analysis with experimental conditions.

As previously shown, the condensation of hydrazinium salt **4**-2H^+^ and benzaldehyde leads to the formation of hydrazonium **11**-*Z* selectively as the (*Z*)-isomer, a process that is mildly exergonic (−2.2 kcal mol^−1^). However, isomerization to the (*E*)-hydrazonium **11**-*E* is facile and only slightly endergonic (1.9 kcal mol^−1^). As discussed below, the cycloaddition occurs *via* the (*E*)-isomer **11**-*E*, and so this additional energy must be taken in to account when estimating the full activation energy required for cycloaddition.

**Fig. 5:**
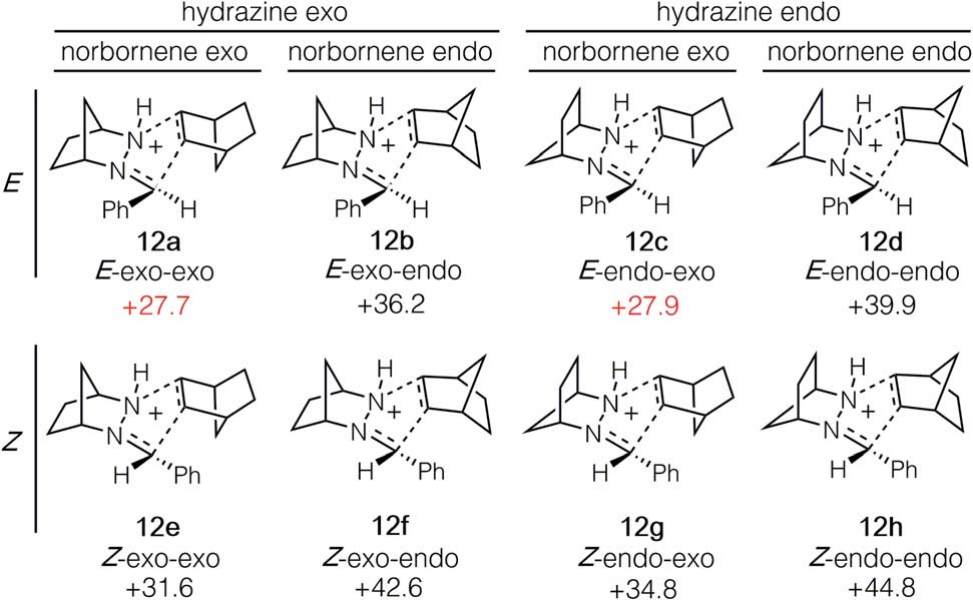
Isomeric transition state structures **12a–h** for cycloaddition of hydrazonium ion **11**-*E* and norbornene (**7**). Numbers represent relative energies in kcal mol^−1^.

The cycloaddition of hydrazonium **11**-*E* and norbornene (**7**) could conceivably occur *via* eight different transition states comprised of different combinations of the *E* and *Z* hydrazine isomers, endo or exo relative to the [2.2.1]-hydrazine fragment, and endo or exo relative to norbornene (Fig. 4). We calculated each of these possibilities to ensure that we had an accurate picture of the lowest energy pathway and to understand the potential impact of structural features that might enforce an alternative pathway. As expected, we found that all transition states involving the (*Z*)-hydrazonium (**12e–h**) were significantly higher in energy than their corresponding (*E*)-hydrazonium counterparts, undoubtedly due to steric congestion of the phenyl ring and the incoming norbornene. Similarly, all four transition states that occurred endo with respect to norbornene (**12b**, **d**, **f**, **h**) were found to be higher in energy by substantial margins over the exo transition states. Interestingly, among the four norbornene–exo transition states, the calculations suggested that there was essentially no energetic difference between activation barriers for cycloadditions occurring exo (**12a**, **c**) or endo (**12e**, **g**) with respect to the hydrazine component (Fig. 5).

Taken together, the calculations predict that the cycloaddition should be reasonably facile at mildly elevated temperatures and should occur competitively *via* transition states **12a** and **12c**. Indeed, experiments verify this prediction within a reasonable level of accuracy: this cycloaddition was found to occur efficiently at 60 °C to produce the cycloadduct in a 3 : 1 ratio of stereoisomers **14a** and **14c**. This result indicated that the actual energy difference between the two transition states **12a** and **12c** is 0.7 kcal mol^−1^. Thermodynamically speaking, the cycloaddition is a highly favorable event (Δ*G* = −19.7 kcal mol^−1^), and the barrier of cycloreversion to reform **11**-*E* and norbornene is sufficiently high (−45.5 kcal mol^−1^) as to render this step effectively irreversible.

**Fig. 6:**
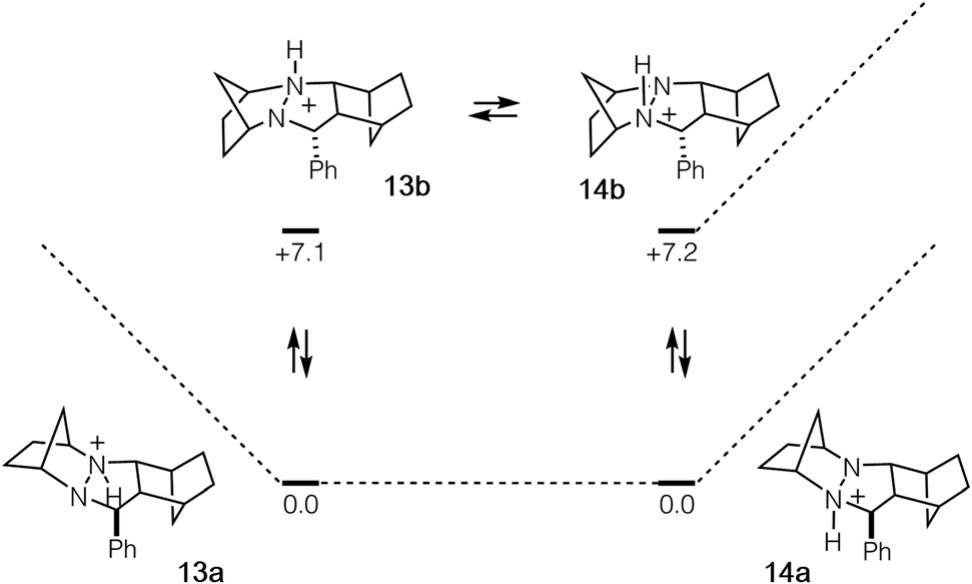
Cycloadduct isomers arising from protonation and nitrogen inversion with relative energies in kcal mol^−1^.

In order to undergo cycloreversion to form the ring-opened hydrazonium intermediate, proton transfer between the two hydrazine nitrogens is necessary (Fig. 3, **13** to **14**). Perhaps not surprisingly, this step was found to be essentially thermoneutral (Fig. 6). It should be noted that there are four possible monoprotonated cycloadducts: two diastereomers for both **13b** and **14b**. Although the other isomers for this system were found to be substantially higher in energy and thus not of concern, with other hydrazines, such structures might not be easily dismissed. Indeed, as discussed in the next section, we have found that such isomers need to be considered to gain an accurate understanding of the cycloreversion energetics.

**Fig. 7:**
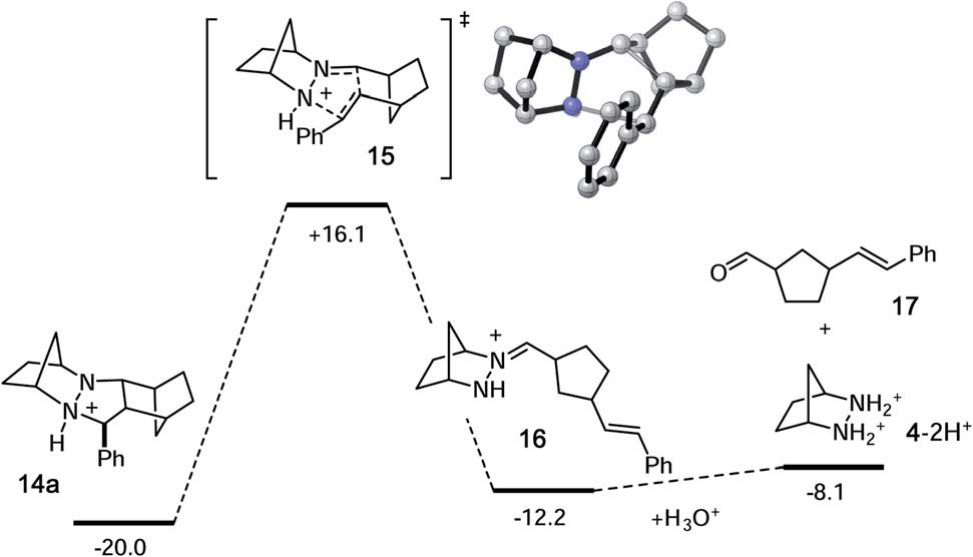
Calculated energies for cycloreversion and hydrolysis of cycloadduct **14a**. Numbers represent relative energies in kcal mol^−1^.

As expected, the cycloreversion was found to have the highest energy barrier of all steps, with a Δ*G*^‡^ = 36.1 kcal mol^−1^, reflecting the substantial stability of the pyrazolidine ring (Fig. 7). The cycloreversion produces the new olefin moiety with the *E*-geometry, which is a consequence of the stereoselectivity of the cycloaddition. Furthermore, the intermediate hydrazonium **16** is revealed preferentially as the *Z*-isomer, which was calculated to be 1.2 kcal mol^−1^ more stable than the corresponding *E*-isomer. In terms of thermodynamics, this step was endergonic (+7.8 kcal mol^−1^), as was the subsequent hydrolysis (+4.1 kcal mol^−1^). This analysis demonstrated that the cycloadduct is the resting state of the catalyst. It also indicated that steps need to be taken to prevent the back reaction of aldehyde **17** to reform the cycloadduct. We demonstrate how this goal can be accomplished in a later section.

### 2.2 Computational evaluation of different hydrazines

**Fig. 8:**
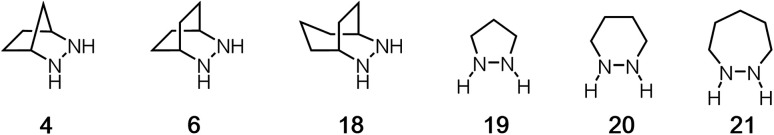
Hydrazines evaluated computationally for ROCOM of norbornene and benzaldehyde.

In the studies described above, we established a full computational profile for the ROCOM reaction between norbornene and benzaldehyde using one specific hydrazine: the [2.2.1]-bicyclic structure **4** we had previously reported. Our next goal was to calculate the same energetic landscape for a set of other representative hydrazines. The aim of this effort was to identify structural features of hydrazines that might impact the various steps along the reaction pathway and that in particular might facilitate the rate-determining cycloreversion step. Due to the large number of individual steps and conformations that needed to be considered, we limited our selection to five additional structures **6**, **18–21** to fully evaluate (Fig. 8). Thus, in addition to the [2.2.1]-bicycle **4**, we examined [2.2.2]-bicycle **6**, [3.2.2]-bicycle **18**, and the 5-, 6-, and 7-membered ring hydrazines **19–21**. In the following sections, we discuss each step of the reaction pathway in turn for each of these alternative structures.

### 2.3 Condensation and *E*/*Z* isomerization

**Fig. 9:**
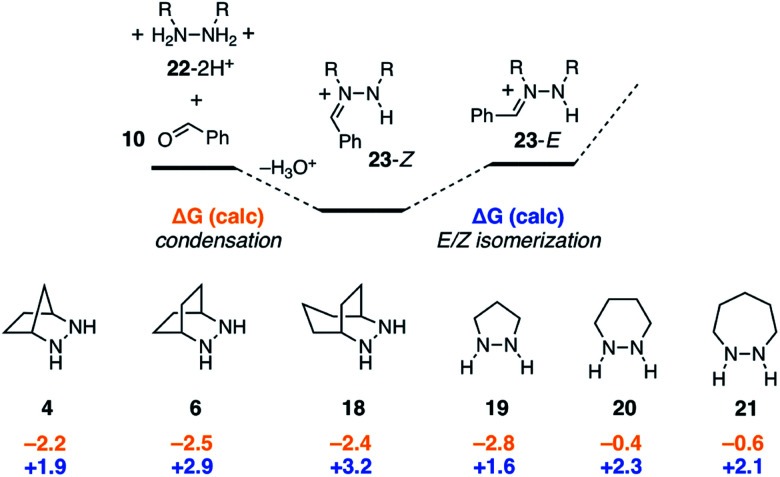
Comparison of condensation and *E*/*Z* isomerization energies for six hydrazines.

As shown in Fig. 9, condensation of the protonated hydrazines **22**-2H^+^ with benzaldehyde to produce the hydrazonium intermediates **23**-*Z* was found to be mildly exergonic by 2.5 kcal mol^−1^ for all hydrazines except for **20** and **21**, which were nearly thermoneutral. In these cases, it would seem that the inherent enthalpic favorability of the condensation is offset by the well-known increase in ring-strain that arises from the incorporation of sp^2^-hybridized atoms in six and seven-membered rings.^43^ Meanwhile, the *Z*-isomers were universally more stable than the *E*-isomers, but the difference between the two was relatively small (1.6–3.2 kcal mol^−1^). In combination, the two steps offset one another, rendering the conversion of free hydrazine **22**-2H^+^ to *E*-hydrazonium **23**-*E* nearly thermoneutral. We also conclude that, at least for simple hydrazines, the condensation and isomerization events are minimally impacted by hydrazine structure. The situation would surely be different, however, if for example additional substituents were present on the hydrazine α-carbons.

### 2.4 Cycloaddition

**Fig. 10:**
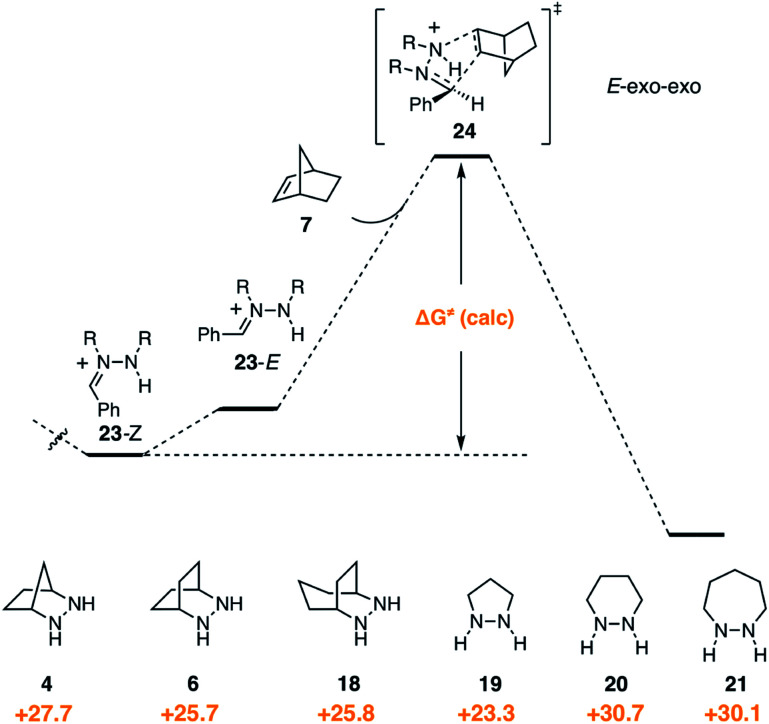
Gibbs free activation energy of cycloaddition and activation barrier dependence on hydrazine structures **4**, **6**, **18–21**.

In contrast to the condensation step, we found that hydrazine structure had noticeable impact on the activation barrier for cycloaddition (Fig. 10). It should be stressed that, even though the cycloadditions proceed *via* the (*E*)-hydrazoniums, the energies shown reflect the difference between the more stable (*Z*)-hydrazoniums **23**-*Z* and transition state structures **24**, which thus represent the full energetic cost of cycloaddition. Interestingly, bicyclic hydrazines **6** and **18**, as well as pyrazolidine (**19**), had lower activation barriers than the [2.2.1]-hydrazine **4**, while the barriers for the larger ring monocyclic hydrazines **20** and **21** were noticeably higher.

**Fig. 11:**
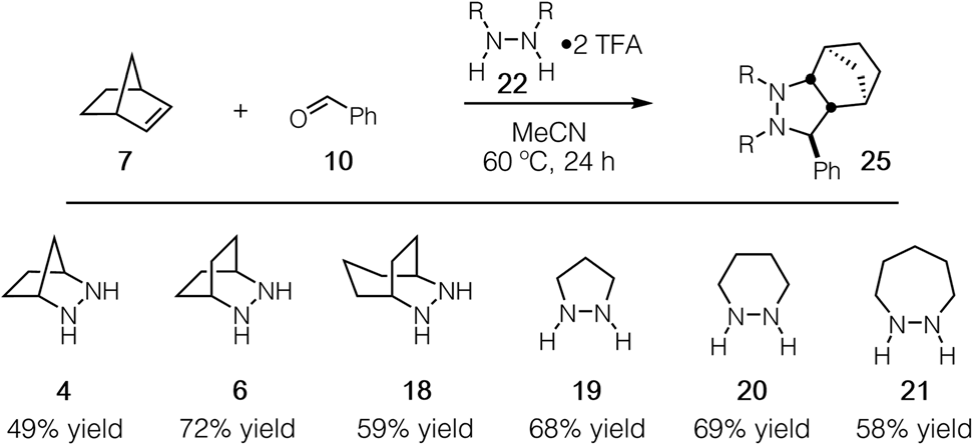
Efficacy of [3 + 2] cycloaddition with hydrazine TFA salts of **4**, **6**, **18–21**; norbornene; and benzaldehyde.

Experimentally, we have found that each of these hydrazines participates in the cycloaddition with similar efficiency (Fig. 11) at 60 °C in acetonitrile over 24 h, conditions identified in our optimization studies (see below). We note that, even though the reaction efficiencies were similar in each case, the isolated yields were often modest due to the tedious purification of the highly polar cycloadducts. Because the catalytic protocol does not require isolation of these intermediates, this difficulty was not of great concern.

### 2.5 Proton transfer

**Fig. 12:**
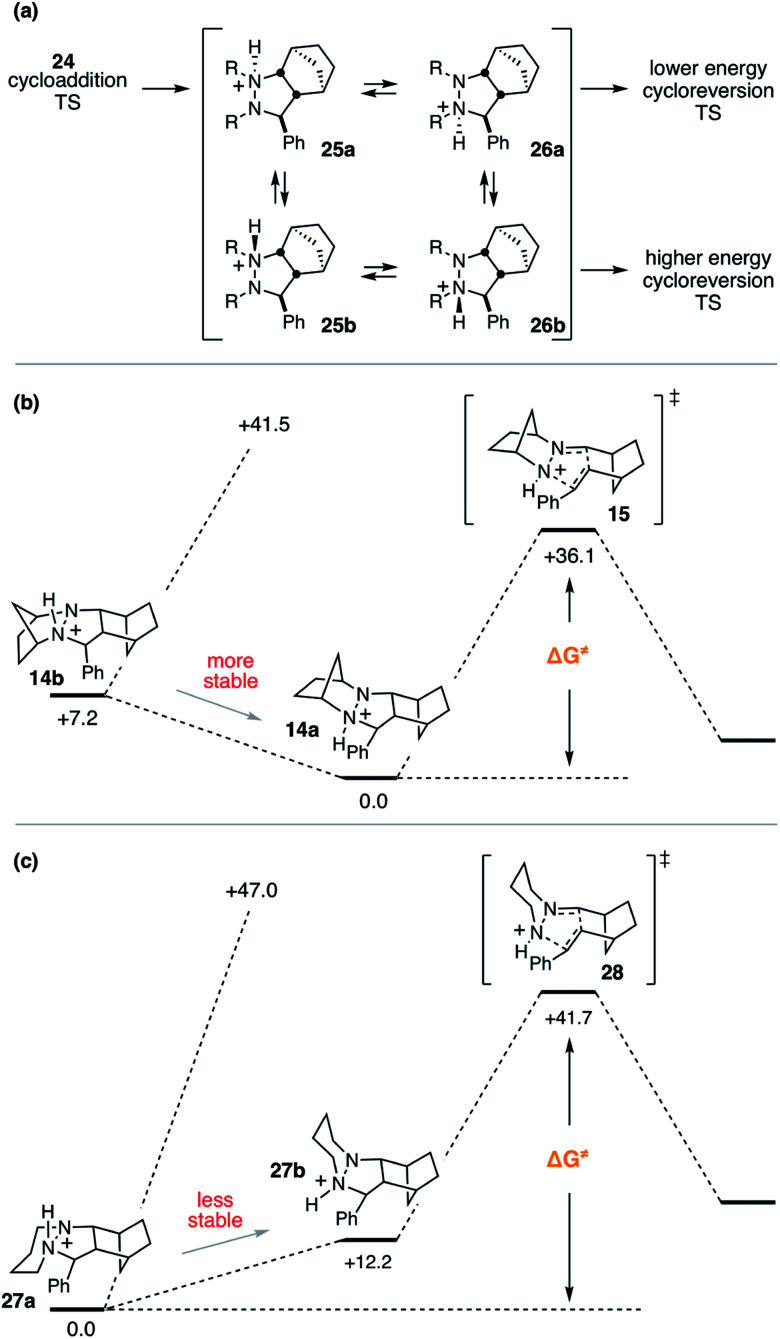
Protonated hydrazine stereochemistry. (a) Generic depiction of protonation isomers and examples in which the (b) more stable isomer or (c) less stable isomer leads to the lowest energy transition state.

Despite the seeming simplicity of the proton transfer between the two cycloadduct nitrogens that must occur before cycloreversion, the analysis of this event was found to involve a surprising level of complexity. Much of this complexity derives from the fact that there exist two diastereomers for each of the two regioisomeric protonated cycloadducts **25** and **26** (Fig. 12a), and it is necessary to determine which of these isomers is most stable in order to have an accurate understanding of the full energy requirements for cycloreversion.

In the case of cycloadduct **14** discussed above [arising from reaction of the [2.2.1]-hydrazine **4**], the diastereomer **14a** was found to be the most stable, while the other diastereomer **14b** was 7.2 kcal mol^−1^ less stable (Fig. 12b). Although it might have been the case that cycloreversion of this less stable isomer proceeded through a lower transition state barrier, we found that the opposite was true; the barrier from **14b** was 41.5 kcal mol^−1^, significantly higher than *via***14a** (36.1 kcal mol^−1^). Thus, in this case the most stable isomer has the lowest cycloreversion activation barrier, and the difference between **14a** and **15** reflects the total energy requirement for this step.

On the other hand, many other hydrazine structures have the opposite situation. For example, the six-membered hydrazine **20**, the lowest energy cycloreversion transition state arises from isomer **27b** (Fig. 12c), but the diastereomeric compound **27a** was more than 12 kcal mol^−1^ more stable. Thus, while cycloreversion from **27b** was calculated to be only 29.5 kcal mol^−1^, the overall barrier including isomerization from **27a** to **28** was 41.7 kcal mol^−1^. Meanwhile, cycloreversion *via* diastereomer **27a** was calculated to be 47.0 kcal mol^−1^. These findings illustrate the need to control the conformational freedom of the hydrazine catalysts and underscore the importance of considering the various isomeric forms during a computational investigation of catalyst structure.

### 2.6 Cycloreversion

**Fig. 13:**
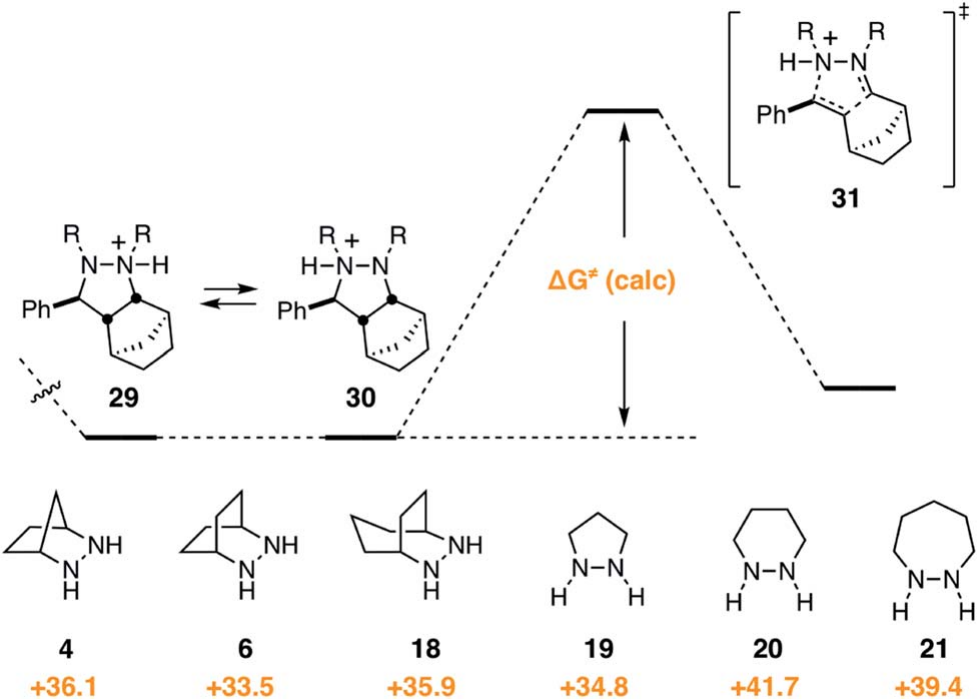
Gibbs free activation energy of cycloreversion and activation barrier dependence on hydrazine structures **4**, **6**, **18–21**.

Given that the cycloreversion represented the highest barrier step, it was of greatest interest to see how hydrazine structure impacted the energetics of this process. The calculated energies for each of the six hydrazines are shown in Fig. 13. Here again, the values indicate the total energy barrier from the most stable protonated cycloadduct intermediate. Interestingly, both the [2.2.2]-bicyclic hydrazine **6** and the five-membered pyrazolidine **19** led to cycloreversion steps with significantly lower energy barriers than the [2.2.1]-hydrazine **4**. Among these, the [2.2.2]-hydrazine **6** was found to have the lowest barrier to cycloreversion. Meanwhile, both the six- and seven-membered hydrazines **20** and **21** were calculated to have significantly higher barriers.

**Fig. 14:**
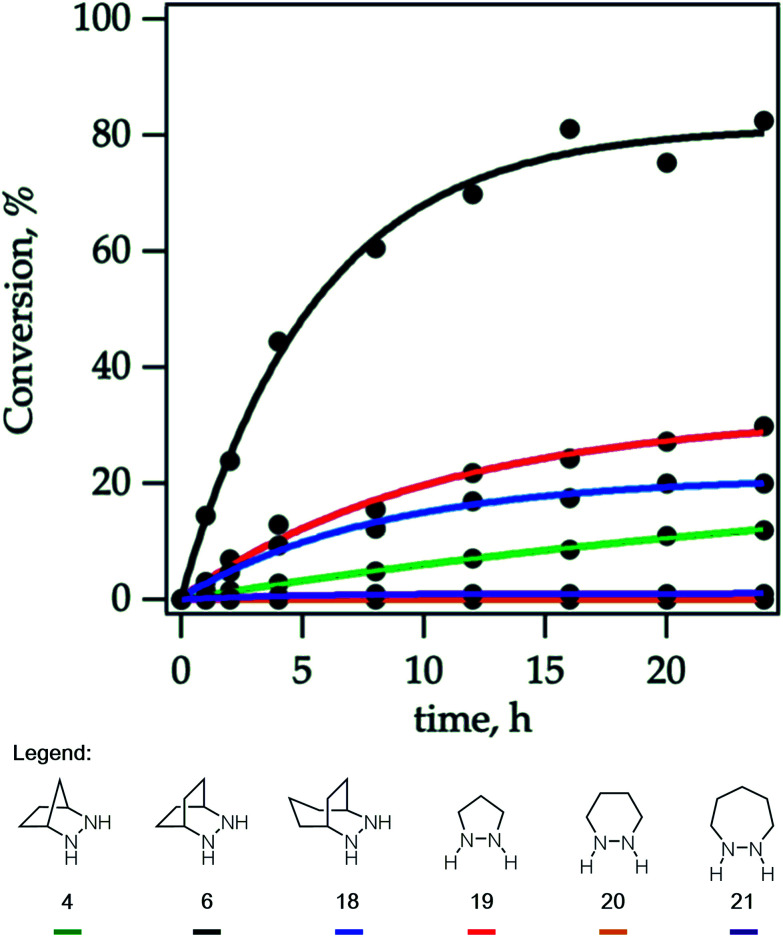
Cycloreversion rates of cycloadducts **30** derived from hydrazines: **4**, **6**, **18–21**.

Due to the importance of this step, we wanted to see if these calculated energies could be experimentally verified. To pursue this goal, we monitored by quantitative ^1^H NMR the cycloadducts **30** under reaction conditions identified in our optimization studies for cycloreversion (*vide infra*). The computationally determined reaction activation energies of 35 kcal mol^−1^ or greater for the cycloreversions demanded elevated temperatures. Thus, to accurately monitor the reaction conversion over time, we heated the reaction mixture at 140 °C in a medium-walled, sealed NMR tube in a silicone oil bath, and acquired ^1^H NMR spectra at regular intervals. Due to some overlap in both aliphatic and aromatic regions of the starting material and product for some of the hydrazines, we followed the growth of the ring-opened product by integration of the styrenyl peaks *versus* mesitylene as an internal standard (Fig. 14).

By heating a 0.2 M solution of the cycloadducts with 2 equiv. TFA in acetonitrile at 140 °C, the reactions proceeded cleanly enough to produce creditable data. In one instance – the 5-membered pyrazolidine – there was some discrepancy between the growth of the product and the decay of the parent starting material.

Hexahydropyridazine-(**20**)- and 1,2-diazepane-(**21**)-derived cycloadducts led to no ring-opened product under the standard conditions. However, we did observe small amounts (∼4%) of product formation from [7-membered] cycloadduct derived from **21** under more forcing conditions (160 °C). The cycloadduct **14** derived from the [2.2.1]-bicyclic hydrazine **4** used in our previous studies showed low reactivity, with only 12% conversion after 24 h. Meanwhile [5-membered pyrazolidine] cycloadduct derived from **19** and [3.2.2]-bicyclic hydrazine cycloadduct derived from **18** showed slight improvement with modest conversions of 30% and 20% respectively after 24 h. Most notably, [2.2.2]-bicyclic hydrazine cycloadduct **32** underwent cycloreversion at a significantly higher rate than the other structures, leading to 80% conversion after 24 h. It should be stressed that the ordering of hydrazine reactivity accurately mimics the computational data.

**Fig. 15:**
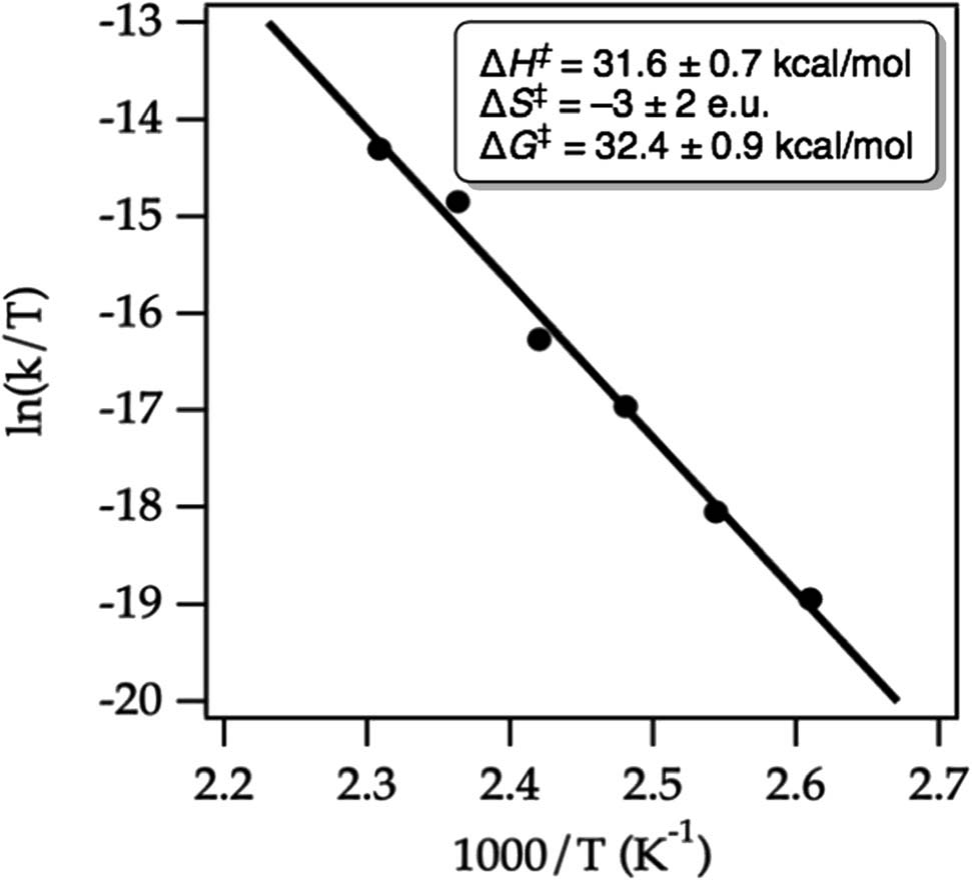
Eyring plot for the cycloreversion of cycloadduct **32** to form product **49** in MeCN-*d*_3_. The line represents the least-squares fit to the data points.

We conducted an Eyring analysis to validate the calculated activation energies with experimental data. As expected, both starting material decay and product growth were consistent with first-order behavior. First-order fitting allowed the extraction of first-order rate constants. The obtained Eyring plot (Fig. 15) showed an enthalpy of activation of Δ*G*^‡^ = 31.6 ± 0.7 kcal mol^−1^, indicating considerable bond breaking in the transition state, while the negligible entropy of activation Δ*S*^†^ = −3 ± 2 e.u. was fully consistent with a unimolecular ring-opening. Notably, the experimentally determined Gibbs free activation energy of Δ*G*^‡^ = 32.4 ± 0.9 kcal mol^−1^ was very close to the calculated Gibbs free activation energy Δ*G*^‡^ = 33.5 kcal mol^−1^. These results thus give us confidence that our chosen computational method has merit for hydrazine design and development.

**Fig. 16:**
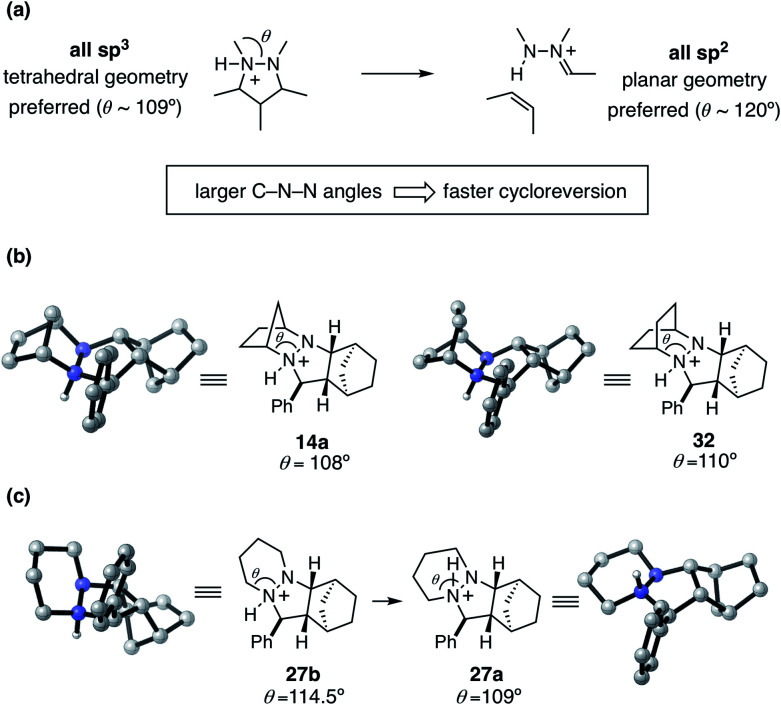
Hydrazine reactivity for cycloreversion. (a) C–N–N angle *θ* and hybridization change during cycloreversion (b) C–N–N angle *θ* comparison in rigid hydrazines **14a** and **32** (c) C–N–N angle *θ* in flexible cycloadduct conformers **27a** and **27b**.

To help explain the observed trend in hydrazine reactivity we considered the atoms undergoing rehybridization during cycloreversion. Specifically, during the conversion of the starting material pyrazolidine ring to the product hydrazonium, the two nitrogen atoms undergo sp^3^ to sp^2^ rehybridization (Fig. 16). This reorganization corresponds to a change in preferred bonding angles of roughly 109° to 120°. Thus, we predict that the larger the C–N–N angle *θ* enforced by the hydrazine structure, the more facile the cycloreversion should be. This rationale is consistent with the relative reactivities of the [2.2.1]- and [2.2.2]-hydrazines **4** and **6**, with the latter having a larger angle of 110° *versus* 108° for the former.

On the other hand, we have found that this trend is counterbalanced in larger ring hydrazines by an increase in the number of available conformations for the protonated cycloadducts. This increased conformational flexibility can lead to stable but unproductive transoid hydrazine conformations, as with the six-membered adduct **27a** described earlier. Although in these cases a Curtin–Hammett situation might exist, the additional energetic requirement to switch from the transoid to cisoid conformation is often high enough to make the cycloreversions with these hydrazines significantly less effective. Thus, a key goal of future hydrazine design efforts is to identify structures that enlarge the N–N–C bond angle while restricting the hydrazine moiety to the cisoid conformation.

## 3 Experimental optimization

### 3.1 Cycloaddition

Because the pyrazolidine intermediates of the hydrazine-mediated norbornene carbonyl–olefin metathesis reaction are stable, we recognized it would be possible to study the cycloaddition and cycloreversion processes independently to determine how different parameters impacted each step. Because of their substantially lower activation energies, cycloaddition reactions were anticipated to be much more facile and to proceed at lower temperatures than the cycloreversions. Indeed, we found that generally cycloadditions occurred efficiently at 60 °C in acetonitrile (Table 1, entry 1). An increase in concentration improved the conversion, which makes sense for a bimolecular cycloaddition process (entry 2). However, increasing the concentration further was not possible due to insolubility of the hydrazinium salt. At a higher reaction temperature of 80 °C, norbornene was observed to condense at the top of the reaction flask, which thus inhibited reaction progress (entry 3). The high volatility of norbornene could be mitigated by the use of 10 equiv. of the alkene, but the use of a lower reaction temperature was more economical. The reaction proved to be robust with regard to the use of different solvents such as MeOH (entry 4) and various co-acids (*e.g.* HCl) (entry 5). We also examined the introduction of additives such as H_2_O (entry 6) and catalytic Sc(OTf)_3_ (entry 7), since they were found to be beneficial for optimization of the cycloreversion step (*vide infra*). Under the standard conditions, these additives suppressed the yield of cycloaddition; however, we did not view this diminished efficiency as overly worrisome, since the conditions for cycloreversion were considerably more forcing and thus, we reasoned, likely to mitigate these effects.

**Table 1:** Exploration of effect of reaction conditions on [3 + 2] cycloaddition of hydrazine salt **6**, norbornene, and benzaldehyde^a^

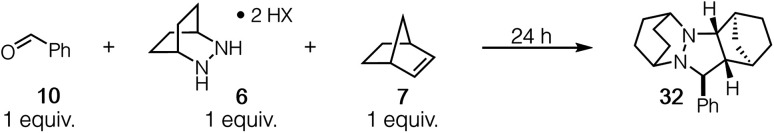						
Entry	HX	Temp (°C)	Solvent	Conc. (M)	Additive	Yield (%)
1	TFA	60	MeCN	0.1	—	55
2	TFA	60	MeCN	0.2	—	72
3	TFA	80	MeCN	0.2	—	43
4	TFA	60	MeOH	0.2	—	56
5	HCl	60	MeCN	0.2	—	53
6	TFA	60	MeCN	0.2	H_2_O (20 eq.)	42
7	TFA	60	MeCN	0.2	Sc(OTf)_3_ (0.2 eq.)	36

a See ESI for experimental details. Yields reflect isolated and purified product.

With the conditions identified in Table 1, entry 2, we prepared a series of cycloadducts derived from hydrazinium salt **6**, norbornene (**7**), and a series of aldehydes (Table 2). The goal here was to discover how aldehyde structure impacted the efficiency of the cycloaddition step. Both *p*- and *o*-tolualdehydes led to cycloadducts (entries 1 and 2), although the latter afforded a substantially higher yield of 90% for reasons that are not clear. In terms of *ortho*-substituted benzaldehydes, the yield of the cycloadducts decreased in correlation to the steric demand of the substituent (entries 2–5). It is understandable that congestion around the hydrazonium fragment would inhibit the cycloaddition step. Not surprisingly, 2,4-dimethylbenzaldehyde led to a similar yield as *o*-tolualdehyde (entry 6). The more electron-rich *p*-anisaldehyde was significantly less productive (entry 7), which accords with the finding that these cycloadditions operate *via* the LUMO of the hydrazonium intermediate. On the other hand, *p*-nitrobenzaldehyde was also somewhat less effective than benzaldehyde (entry 8). The best performing substrate was *p*-chlorobenzaldehyde (entry 9), which afforded the cycloadduct in a superior 94% yield. Similarly effective was *o*-chlorobenzaldehyde (entry 10), which afforded an 88% yield of the cycloadduct. The analogous *o*-fluorobenzaldehyde was also productive (entry 11), albeit somewhat less so than the chloro substrate. In terms of heteroaromatic substrates, both furfural (entry 12) and thiophene carboxaldehyde (entry 13) yielded cycloadducts, however only the former did so in good yield. Finally, an α-branched aliphatic substrate, cyclohexane carboxaldehyde, furnished product in good yield (entry 14); however, a non α-branched substrate led to poor yield (entry 15). We speculate that the latter may participate in unwanted hydrazenamine formation and subsequent aldol or Mannich type side reactions.

**Table 2:** Substrate scope for [3 + 2] cycloaddition of aldehydes with norbornene and hydrazine salt **6**^a^

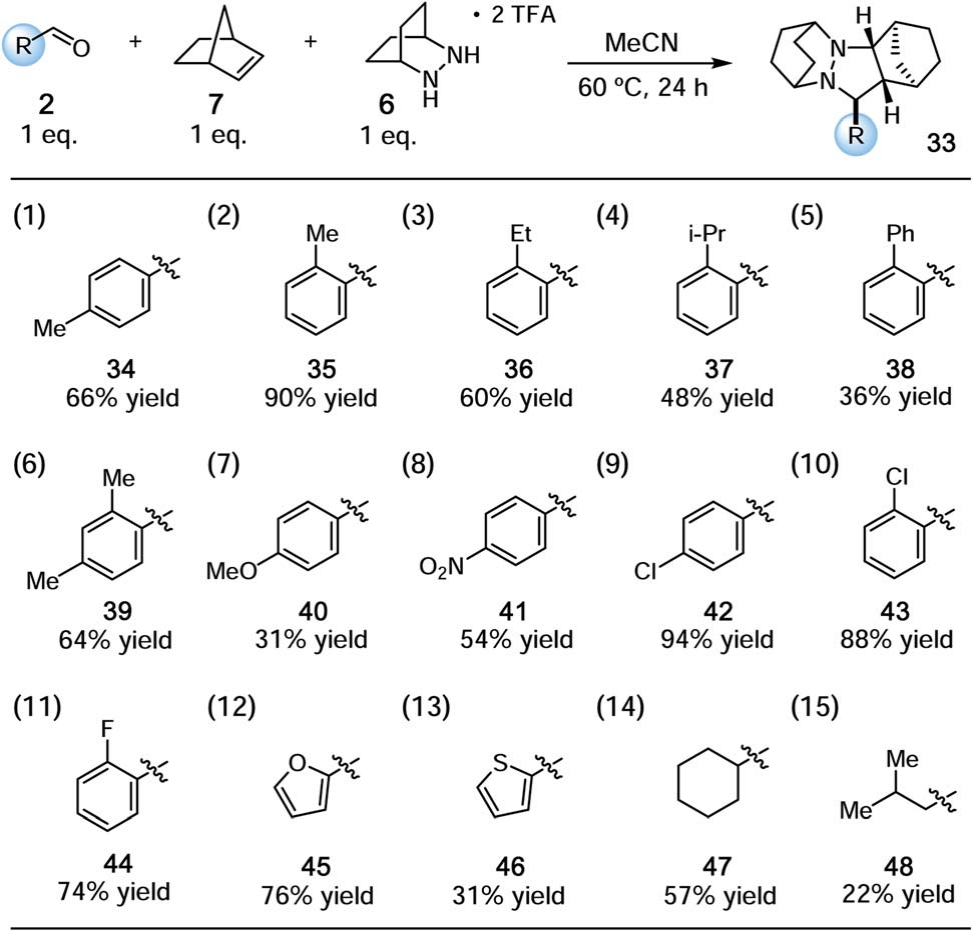

a See ESI for experimental details. Yields reflect isolated and purified product.

### 3.2 Cycloreversion

With ready access to pyrazolidine cycloadducts in hand, we set about optimizing the cycloreversion step. Initially, we found that thermolysis of the cycloadduct **32** in the absence of acid led to the formation of only a trace amount of ring-opened product **17** (entry 1). In the presence of 2 equiv. of TFA, which mimics the conditions identified for the cycloaddition step, a 26% yield of ring-opened product was observed by ^1^H NMR as judged by the styrenyl proton peaks (entry 2); however, no aldehyde product **17** was isolated. We suspected that the ring-opened product was likely present in the form of unhydrolyzed hydrazonium **9** or hydrazenamine **50** intermediates, and indeed we have been able to isolate and characterize both of these species. Although changing from acetonitrile to methanol solvent was not effective in remedying this situation (entry 3), the inclusion of 20 equiv. of ethylene glycol resulted in a small increase in the observed styrenyl peaks. More importantly, we also were able to isolate a small amount of product acetal **49** (entry 4). When 20 equiv. of water were also added to the reaction mixture (entry 5), both measures of conversion and yield of **49** were increased further. A slight benefit was observed by increasing the concentration of the reaction (entry 6), but the isolated yield remained low and substantially less than the NMR yield. It has been reported that Sc(OTf)_3_ catalyzes hydrazine exchange,^44^ and we speculated that this behavior might also serve in the present reaction. Indeed, when 20 mol% Sc(OTf)_3_ was included, the conversion was increased to 80% and the isolated yield of acetal **49** was improved to 60% (entry 7). To maximize the isolated yield, we found that an additional time for conversion to the acetal product led to an isolated yield of 78% (entry 8). These results stand as a stark demonstration that a major-perhaps the major-impediment for this transformation is not the cycloreversion step, but rather the hydrolysis of the hydrazonium intermediate following cycloreversion. This issue is discussed further in the catalysis section.

A brief mention of the stereochemistry of ROCOM product **49** should be made. Although the cycloreversion step initially delivers the *cis*-hydrazonium intermediate **9**, the product **49** is isolated as a 1 : 1 mixture of *cis* and *trans* isomers. Epimerization of the formyl-bearing stereocenter undoubtedly occurs *via* either isomerization between the hydrazonium and hydrazenamine intermediates **9** and **50**, keto–enol tautomerization of the free aldehyde **17**, or by way of the oxocarbenium intermediate on the way to the formation of acetal **49**. The lack of diastereoselectivity in this transformation reflects the thermal equilibrium of the two equienergetic 1,3-disubstituted cyclopentanes. It should be noted that this type of equilibration is a general feature of cyclopentanecarboxaldehydes, which is likely to occur with any carbonyl–olefin metathesis strategy. With an eye toward synthetic applicability, more highly substituted substrates (*e.g.* see eqn (1) and Fig. 18) can and do lead to diastereoselective products.

Using these optimized conditions, we next looked at the effect of modifying the substituent “R” (originally derived from the aldehyde component in the cycloaddition) on the cycloreversion efficiency (Table 4). Both *para* and *ortho* methyl substitution on the phenyl ring were tolerated (entries 1 and 2). An examination of the steric demand of this *ortho* substituent was inconclusive, since Me and i-Pr (entries 2 and 4) produced lower yields than Et (entry 3) or the parent compound (see Table 3, entry 7 for comparison). Notably, however, the presence of an *ortho* phenyl substituent led to a superior yield of 90% (entry 5). Meanwhile a 2,4-dimethylphenyl substituent also led to an efficient reaction (entry 6). In terms of electronic variation, an electron-donating methoxy substituent (entry 7) was better than an electron-withdrawing nitro group (entry 8), although both proved to be productive. Significantly better than either of these was a 4-chlorophenyl group (entry 9), which furnished the product in 90% yield. Interestingly, a 2-chlorophenyl moiety was significantly less effective (entry 10), but a 2-fluorophenyl group was more so (entry 11). Meanwhile, a furan group did not survive the cycloreversion conditions intact (entry 12); however, a thiophene ring was a viable substituent (entry 13), leading to product in reasonable yield. With the current catalyst and conditions, we did not observe any reaction with aliphatic substituents (entries 14 and 15). This result was not surprising because our calculations suggested cycloreversion of these substrates has an energy barrier of approximately 40 kcal mol^−1^. As an additional point, we also verified that cycloreversion of cycloadduct **67** bearing substituents on the norbornyl ring was also viable (eqn (1)), leading to the ring-opened product **68** in 46% isolated yield.

**Table 3:** Optimization study for [3 + 2] cycloreversion of cycloadduct **32**^a^

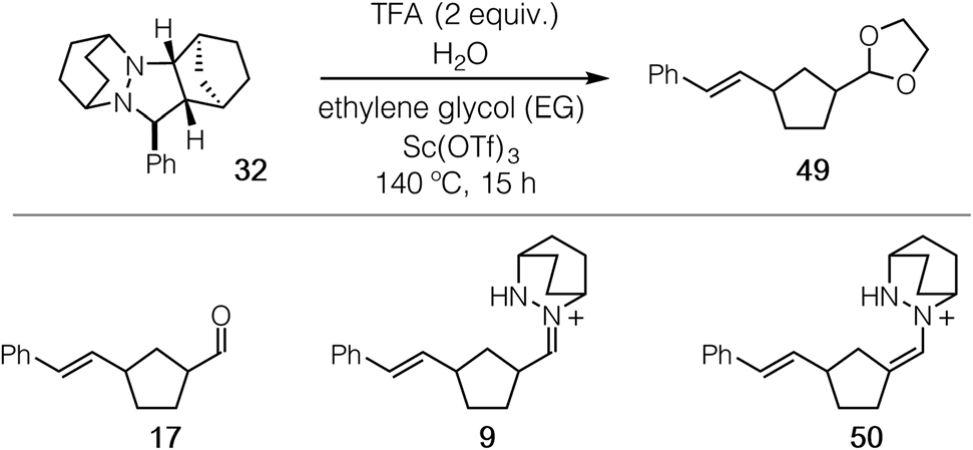						
Entry	Solvent	Conc. (M)	EG (eq.)	H_2_O (eq.)	Sc(OTf)_3_ (eq.)	Yield^a^ (%)
1^b^	MeCN	0.1	—	—	—	— (4)
2	MeCN	0.1	—	—	—	— (26)
3	MeOH	0.1	—	—	—	— (20)
4	MeCN	0.1	20	—	—	5 (31)
5	MeCN	0.1	20	20	—	22 (67)
6	MeCN	0.2	20	20	—	35 (74)
7	MeCN	0.2	20	20	0.2	60 (80)
8^c^	MeCN	0.2	20	20	0.2	78 (83)

a Yields reflect isolated and purified product. Numbers in parentheses reflect yield determined by ^1^H NMR *versus* an internal mesitylene standard, *via* integration of the styrenyl proton peaks, which represent all forms of the ring-opened product (acetal, aldehyde, hydrazonium, hydrazenamine).

b No TFA added.

c Procedure included additional 1 h hydrolysis at 140 °C with subsequent addition of TFA (2 equiv.) and ethylene glycol (20 equiv).

**Table 4:** Effect of substituents for [3 + 2] cycloreversion of cycloadducts **33**^a^

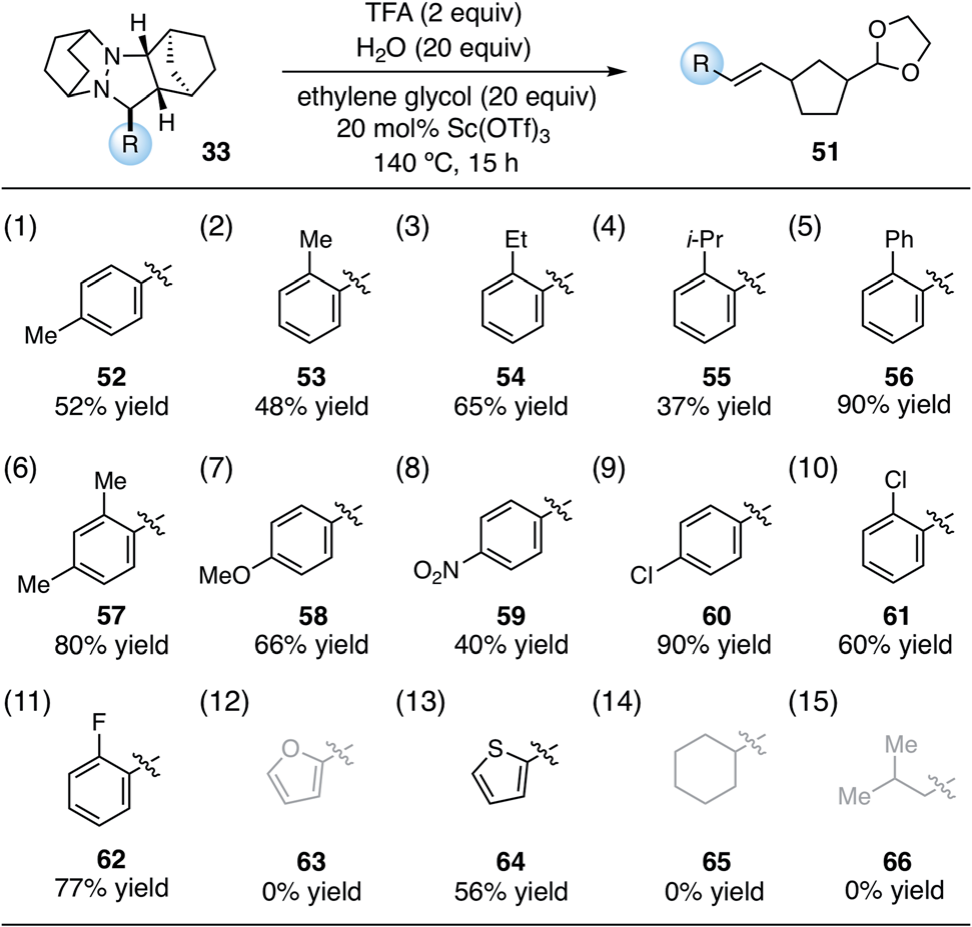

a See ESI for experimental details. Yields reflect isolated and purified product.

From the data in Table 4, it is difficult to draw strong conclusions about the impact of the aldehyde-derived substituent on cycloreversion efficiency. It appears that in terms of both steric and electronic factors, a “Goldilocks” principle may be operative, *i.e.* groups that are not at either extreme are the most productive. On the other hand, the trends are not strong and other factors (*e.g.* solubility, propensity for acetal formation) may well be factoring in to the observed yields. One significant conclusion to be drawn, however, is that a variety of aryl groups are tolerated for the key cycloreversion step, and that the nature of this group is not overly deterministic of the success of the reaction.1
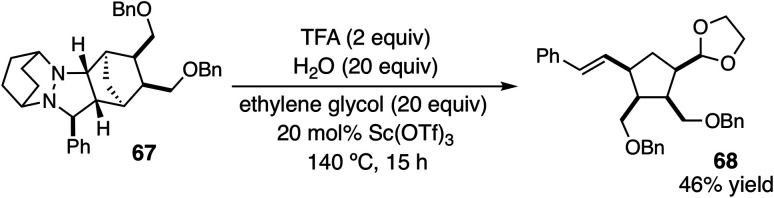


## 4 Catalysis

**Fig. 17:**
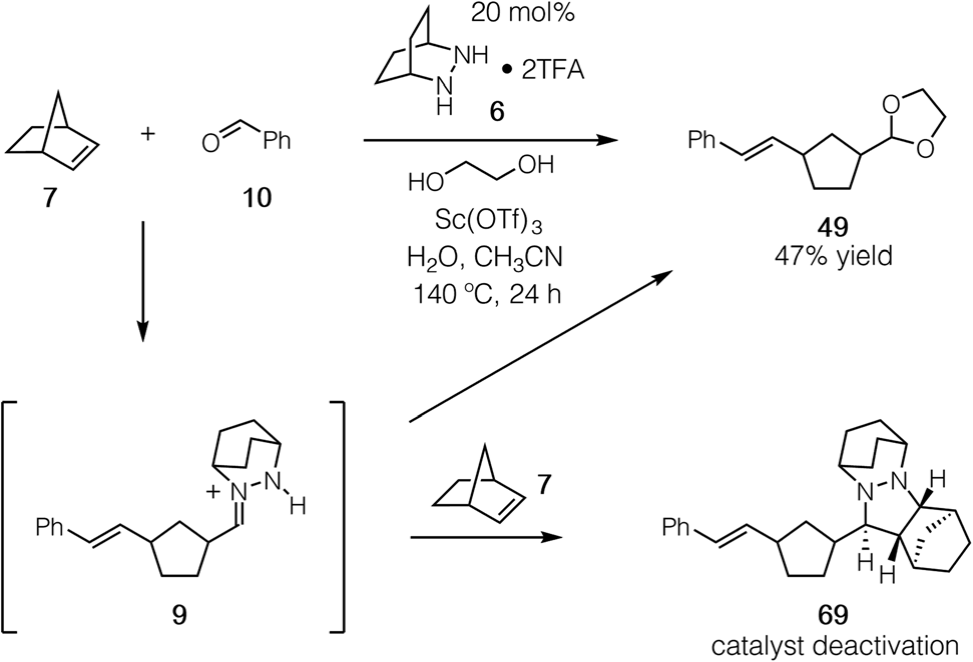
Catalytic ROCOM of norbornene to produce **49** and the undesired formation of cycloadduct **69**.

Having established that both the cycloaddition and cycloreversion steps of the norbornene ROCOM are viable, we set our sights on developing the catalytic process. For these attempts we employed the conditions identified in our optimization of the cycloreversion step (see Table 3, entry 8). Using 20 mol% **6** and a 24 h reaction time, we observed the formation of the ROCOM acetal product **49** in up to 47% yield, which represents slightly less than 2.5 turnovers (Fig. 17). Although this result demonstrated conclusively that catalysis was feasible, further improvements proved elusive because of catalyst consumption.

Crucially, we found that this catalyst side reaction was a result of the ring-opened hydrazonium intermediate **9** undergoing cycloaddition with a second equivalent of norbornene to generate cycloadduct **69**. Because the current catalyst and conditions do not enable cycloreversion with aliphatic substituents, this cycloadduct is unreactive and thus represents a dead-end for the catalyst. The conclusion we draw then is that hydrazonium hydrolysis is a major factor for this catalytic reaction, not simply the cycloreversion step as we had previously assumed.

**Fig. 18:**
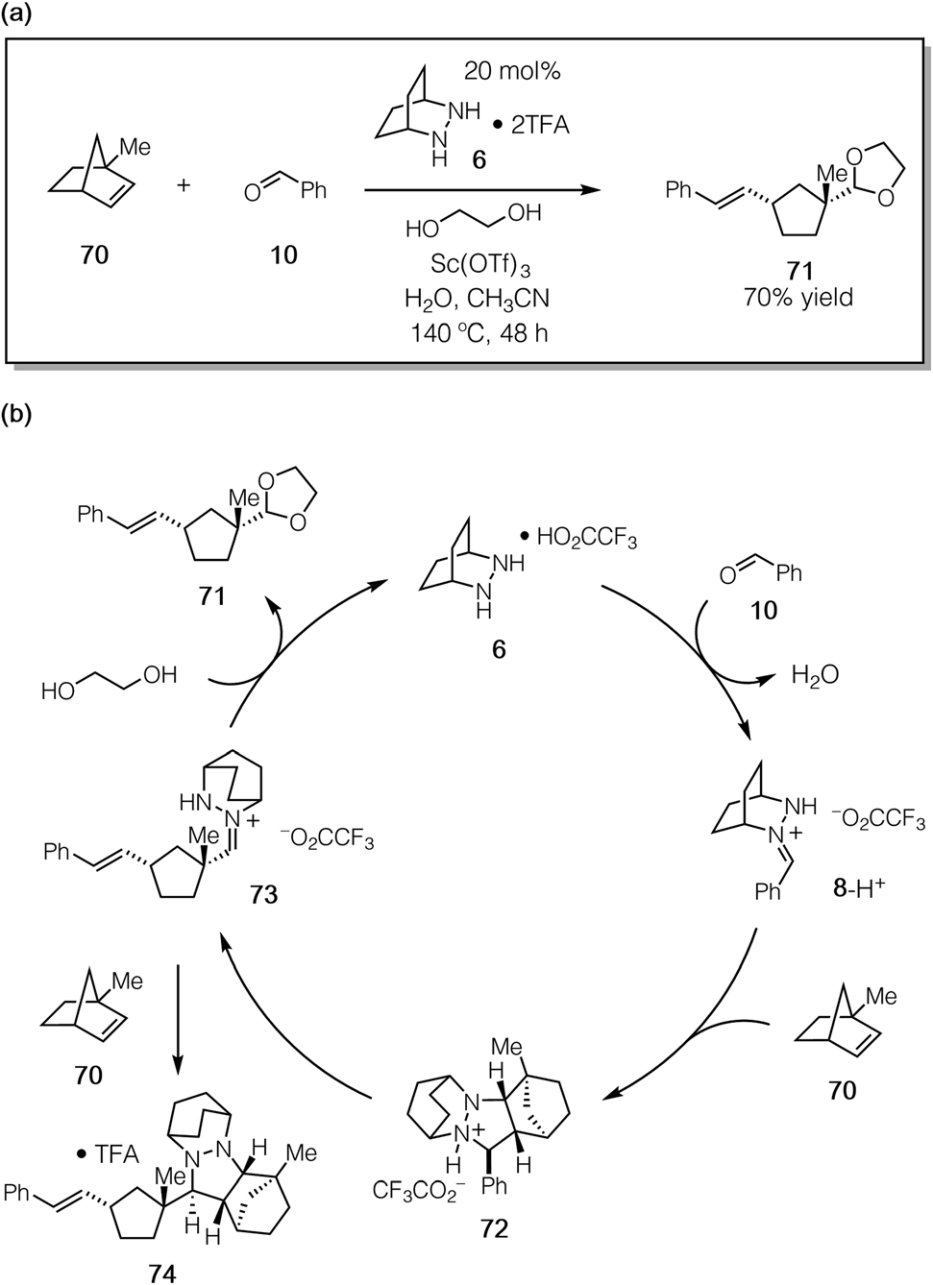
(a) Catalytic ROCOM of 1-methylnorbornene (**70**) and (b) mechanistic rationale.

To confirm that the hydrolysis step was in fact the problematic one, we conducted the reaction of 1-methylnorbornene **70** (Fig. 18a). We reasoned that this substrate would (a) undergo regioselective cycloaddition to produce intermediate **72***via* a transition state that minimized the steric conflict of the bridgehead methyl and hydrazonium aryl groups, (b) preclude hydrazenamine formation from intermediate **73**, (c) destabilize the hydrazonium **73** and thus accelerate hydrolysis/alcoholysis of this intermediate, and/or (d) retard the rate of cycloaddition of **73** with a second equivalent of 1-methylnorbornene **70**, thus hindering the formation of the catalyst-deactivating cycloadduct **74** (Fig. 18b). In the event, when **70** was subjected to the catalytic protocol, we observed the formation of adduct **71** in 70% isolated yield. This result demonstrated conclusively that hydrazine-catalyzed COM can effect the ring-opening of norbornene substrates.

On the other hand, even with this more hindered substrate, the formation of the undesired cycloadduct **74** was still competitive and resulted in deactivation of the hydrazine catalyst. It is thus abundantly clear that future catalyst design should include structural elements that facilitate the rapid hydrolysis of the hydrazonium intermediates. Fortunately, the same structural features that should accelerate hydrolysis (*e.g.* electron-withdrawing functionality, sterically demanding groups) are the same ones that can be expected to lower the barrier for cycloreversion. There are thus grounds for optimism that the design of next-generation catalysts will continue to be a productive undertaking for this chemistry.

## 5 Conclusions

In summary, we have shown that hydrazine-catalyzed carbonyl–olefin metathesis can be extended to the ring-opening reactions of less-strained olefins, specifically norbornenes. Computational analysis was found to reliably predict reaction energetics and allowed for the identification of a bicyclic hydrazine that displayed significantly enhanced efficiency. In particular, we identified a structure–activity relationship between the hydrazine C–N–N bond angle, with greater angles facilitating cycloreversion. Our studies revealed that cycloadditions with electronically unbiased hydrazines, aldehydes, and norbornenes are relatively facile and occur *via* an inverse electron-demand pathway. Meanwhile the cycloreversion step of these reactions are also inverse-demand, leading to the conclusion that catalyst design should benefit from LUMO-lowering structural features. Perhaps most surprisingly, a major roadblock to catalytic turnover turned out not to be cycloreversion but rather hydrolysis of the hydrazonium intermediates. Nevertheless, proof-of-principle catalysis was achieved, laying the groundwork for the development of a robust catalytic platform for norbornene ROCOM. More broadly, the findings from this study set the stage for the development of next-generation catalysts for carbonyl–olefin metathesis with an expanded range of substrates.

## Conflicts of interest

There are no conflicts to declare.

## Supplementary Material

SC-011-D0SC02243H-s001

SC-011-D0SC02243H-s002
