# Supplementary material for: Ring-opening carbonyl–olefin metathesis of norbornenes
Source: Chem Sci. 2020 Jul 1;11(30):7884–95. doi: 10.1039/d0sc02243h (PMC8163149; doi:10.1039/d0sc02243h)
Supplement: SC-011-D0SC02243H-s001 [file SC-011-D0SC02243H-s001.pdf]

Electronic Supplementary Material (ESI) for Chemical Science.
This journal is © The Royal Society of Chemistry 2020

Supporting Information

Ring-Opening Carbonyl-Olefin Metathesis of Norbornenes

Janis Jermaks, Phong Quach, Zara M. Seibel, Julien Pomarole, and Tristan H. Lambert

Table of Contents

General Information *3*

Experimental Details *4*

2,3-Diazabicyclo[2.2.2]oct-2-ene synthesis **4**

Procedures for hydrazine bis-trifluoroacetic acid salt synthesis **5**

6,7-Diazabicyclo[3.2.2]non-6-ene synthesis **7**

Procedure for cycloadduct synthesis **9**

Substituted norbornene synthesis **23**

Procedure for cycloreversion **25**

NMR Spectra *34*

X-ray Crystallography *140*

Kinetic Data *150*

Computational Data *153*

References *225*

General Information

Commercial reagents were purchased from Fisher Chemicals, Sigma-Aldrich, J.T. Baker Chemical Company, TCI and Acros Organics and used without purification. All reactions were performed in the fume hood under atmospheric pressure, unless otherwise noted. Reaction products were stored in scintillation vials at ambient temperature. All cycloaddition and cycloreversion reactions were performed in the Biotage Initiator+ Sixty.

Reactions were monitored by thin-layer chromatography (TLC) on EMD Silica Gel 60 F254 plates under UV light (254 nm) or visualized with I_2. Flash chromatography was performed using silica gel 60 (230-400 mesh) from SilicaFlash on a Biotage Isolera One system. Organic solutions were concentrated under reduced pressure on a Büchi rotary evaporator R-200. Proton nuclear magnetic resonance (^1H NMR) spectra and carbon nuclear magnetic resonance (^{13}C NMR) spectra were recorded on Bruker Magnet System 500 MHz, Varian Magnet System 300 MHz and 400 MHz. All chemical shifts are reported in parts per million (ppm) downfield from tetramethylsilane. Proton resonances are referenced to residual protium in the NMR solvent (7.26 ppm for $CHCl_3$, 2.50 ppm for d_5-DMSO, 3.31 ppm for d_3-methanol, and 7.16 ppm for d_5-benzene). Carbon resonances are referenced to the carbon resonances of the NMR solvent (77.16 ppm for $CDCl_3$, 39.52 ppm for d_6-DMSO, 49.00 ppm for d_4-methanol, and 128.06 ppm for d_6-benzene). Data are represented as follows: chemical shift, multiplicity (br = broad, s = singlet, d = doublet, t = triplet, q = quartet, m = multiplet), coupling constants in hertz (Hz), integration. Mass spectral (MS) data were obtained on Advion Mass Spectrometer equipped with an APCI (Atmospheric Pressure Chemical Ionization) module and HRMS data with direct analysis in real-time mass spectrometry (DART-MS).

Experimental Details

2,3-Diazabicyclo[2.2.2]oct-2-ene synthesis

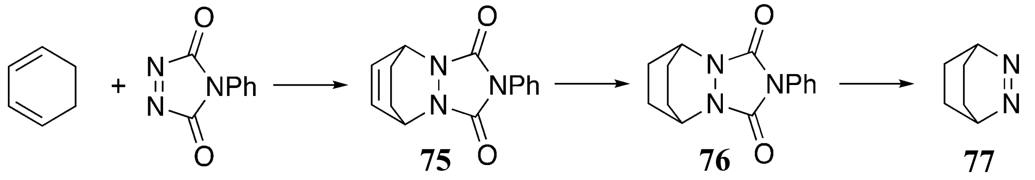

2-Phenyl-5,8-dihydro-1*H*-5,8-ethano[1,2,4]triazolo[1,2-*a*]pyridazine-1,3(2*H*)-dione (75):
In a scintillation vial equipped with a stir bar, 1,3-cyclohexadiene (3.3 mL, 34 mmol) was added dropwise slowly to a 1 M solution of PTAD (6.0 g, 34 mmol) in acetone (34 mL). The color of the solution changed from bright red to dull yellow upon reaction completion. The reaction was left to stir for 30 min. Hexanes (10 mL) was added into the solution mixture to facilitate the precipitation of the product. The white solid product (8.6 g, 98% yield) was collected and rinsed with hexanes (10 mL) via vacuum filtration and dried *in vacuo*. ^{1}H NMR (500 MHz, Chloroform-*d*) δ 7.49 – 7.38 (m, 4H), 7.34 (dq, *J* = 5.5, 2.8 Hz, 1H), 6.73 – 6.26 (m, 2H), 4.95 (dt, *J* = 4.3, 1.5 Hz, 2H), 2.21 (dt, *J* = 9.1, 1.6 Hz, 2H), 1.60 (ddd, *J* = 11.2, 3.5, 2.0 Hz, 2H). ^{13}C NMR (126 MHz, $CDCl_3$) δ 152.5, 131.9, 129.2, 128.0, 125.6, 48.2, 24.9. DART-MS m/z calcd for $C_{14}H_{14}N_3O_2$ $(M + H)^+$ = 256.10805, found 256.10785.

2-Phenyltetrahydro-1*H*-5,8-ethano[1,2,4]triazolo[1,2-*a*]pyridazine-1,3(2*H*)-dione (76):
Compound **75** (8.6 g, 34 mmol), Pd/C (0.2 g, 1.7 mmol) and MeOH (110 mL, 0.3 M) were combined in a 250 mL round bottom flask equipped with a stir bar. The reaction was purged with H_2 for 5 times with a balloon and left to stir vigorously in atmospheric pressure H_2 for 16 h. The reaction mixture was filtered through Celite and then concentrated *in vacuo*, yielding a white solid product (8.5 g, 99% yield). ^{1}H NMR (500 MHz, Chloroform-*d*) δ 7.54 (d, *J* = 7.6 Hz, 2H), 7.46 (t, *J* = 7.8 Hz, 2H), 7.35 (t, *J* = 7.5 Hz, 1H), 4.41 (s, 2H), 2.21 – 2.00 (m, 4H), 1.87 (d, *J* = 8.5 Hz, H). ^{13}C NMR (126 MHz, $CDCl_3$) δ 152.5, 131.9, 129.2, 128.0, 125.6, 48.2, 24.9. DART-MS m/z calcd for $C_{14}H_{16}N_3O_2$ $(M + H)^+$ = 258.12370, found 258.12363.

2,3-Diazabicyclo[2.2.2]oct-2-ene (77):
Cycloadduct **76** (6.0 g, 23 mmol) and *i*-PrOH (310 mL) were charged to a 1 L round bottom flask equipped with a stir bar and a reflux condenser. The flask was sonicated and degassed with N_2 for 15 min. Solid KOH pellets were added under a flow of N_2. The reaction was heated to reflux for 2 h and then left to cool to room temperature. The mixture was cooled to 0 ºC and diluted with 500 mL of water. At this point the reaction was transferred to a 3 L round bottom flask. The pH of the reaction was then adjusted to 1-2 via dropwise addition of concentrated HCl. Afterwards, the pH was raised to 5-6 via addition of 5 M aqueous NH_4OH. 310 mL of 3 M $CuCl_2$ aqueous solution was added to the mixture. 500 mL of hexanes was added to the dark brown solution followed by 1.0 L of 5 M aqueous NH_4OH, resulting in the brown solution turning deep blue. The reaction was

left to stir for 30 min at room temperature. The organic layer was collected and the aqueous layer was extracted with methylene chloride (4 x 200 mL). The combined organic layers were dried over Na_2SO_4, filtered, and concentrated *in vacuo*. The concentrate was then diluted with methylene chloride and washed with 1M aqueous HCl (2 x 100 mL). The organic layer was dried over Na_2SO_4, filtered, and concentrated *in vacuo*. The resultant yellow solid was rinsed with hot pentane (2 x 100 mL). Care was taken to remove only the clear pentane solution and leaving the undesired amber solid behind. The combined pentane solution was gently heated to recrystallize the desired product as white crystalline needles (1.8 g, 70% yield). ^{1}H NMR (500 MHz, Chloroform-*d*) δ 5.10 (s, 2H), 1.57 (d, J = 7.9 Hz, 4H), 1.30 – 1.27 (m, 4H). ^{13}C NMR (126 MHz, $CDCl_3$) δ 61.1, 21.3. DART-MS m/z calcd for $C_6H_{11}N_2$ $(M + H)^+$ = 111.09167, found 111.09159.

Procedures for hydrazine bis-trifluoroacetic acid salt synthesis

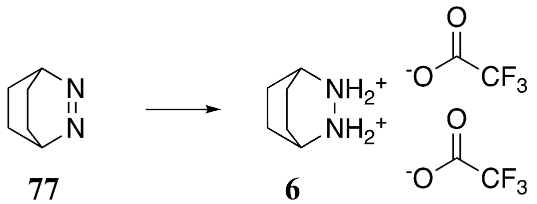

77 **6**

2,3-Diazabicyclo[2.2.2]octane bis-trifluoroacetic acid (6): Diazene **77** (1.0 g, 9.1 mmol), Pd/C (48 mg, 0.45 mmol) and MeOH (50 mL, 0.18 M) were combined in a 100 mL round bottom flask equipped with a stir bar. The reaction was evacuated and purged with H_2 with the sequence repeated 5 times and left to stir vigorously in atmospheric pressure under a balloon of H_2 for 16 h. The reaction mixture was filtered through Celite directly onto trifluoroacetic acid (1.7 mL, 22.7 mmol). The mixture was then concentrated *in vacuo*, yielding a white solid product, which could be further recrystallized with Et_2O/MeOH giving the product (2.1 g, 68% yield) as white needles. ^{1}H NMR (500 MHz, Methanol-d_4) δ 4.88 (s, 1H), 3.28 (s, 1H), 2.09 – 1.97 (m, 2H), 1.93 – 1.76 (m, 2H). ^{13}C NMR (126 MHz, MeOD) δ 163.3 (q, J = 34.5 Hz), 118.3 (q, J = 292.9 Hz), 46.6, 23.8. DART-MS m/z calcd for $C_6H_{13}N_2$ $(M + H)^+$ = 113.10732, found 113.10707.

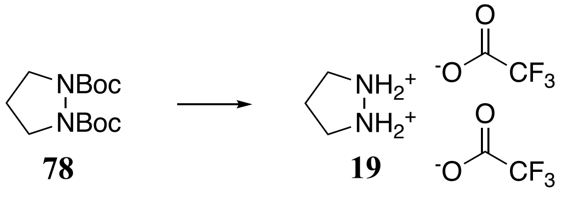

78 **19**

Pyrazolidine-1,2-diium 2,2,2-trifluoroacetate (19): Di-*tert*-butyl pyrazolidine-1,2-dicarboxylate **78** (3.90 g, 14.3 mmol) was dissolved in of DCM (140 mL, 0.1 M). Trifluoroacetic acid (11.0 mL, 143 mmol) was added to the reaction mixture which was then refluxed for 3 h. The mixture was then concentrated *in vacuo*, and dried under vacuum to give the desired product (3.10 g, 72%)

as a yellow oil. ^{1}H NMR (500 MHz, Methanol-d_4) δ 4.91 (s, 11H), 3.24 (t, J = 7.1 Hz, 4H), 2.16 (p, J = 7.3 Hz, 2H). ^{13}C NMR (126 MHz, MeOD) δ 163.1 (q, J = 34.9 Hz), 118.1 (q, J = 292.4 Hz), 47.8, 26.9. DART-MS m/z calcd for $C_3H_9N_2$ $(M + H)^+$ = 73.07602, found 73.07699.

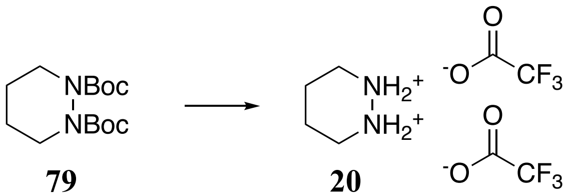

79 **20**

Hexahydropyridazine-1,2-diium 2,2,2-trifluoroacetate (20): Di-*tert*-butyl tetrahydro-pyridazine-1,2-dicarboxylate **79** (5.78 g, 20.2 mmol) was dissolved in of DCM (200 mL, 0.1 M). Trifluoroacetic acid (15.5 mL, 202 mmol) was added to the reaction mixture which was then re-fluxed for 3 h. The mixture was then concentrated *in vacuo*, and dried under vacuum to give the desired product (5.30 g, 84%) as a yellow solid. ^{1}H NMR (500 MHz, Methanol-d_4) δ 4.95 (s, 4H), 3.20 – 3.04 (m, 2H), 1.80 (t, J = 3.2 Hz, 2H). ^{13}C NMR (126 MHz, MeOD) δ 162.9 (q, J = 35.0 Hz), 118.1 (q, J = 292.1 Hz), 46.8, 22.8. DART-MS m/z calcd for $C_4H_{11}N_2$ $(M + H)^+$ = 87.09167, found 87.09261.

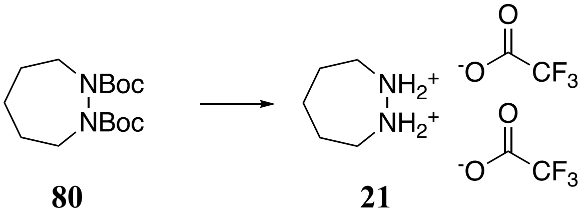

80 **21**

1,2-Diazepane-1,2-diium 2,2,2-trifluoroacetate (21): Di-tert-butyl 1,2-diazepane-1,2-dicarbox-ylate **80** (5.78 g, 20.2 mmol) was dissolved in of DCM (200 mL, 0.1 M). Trifluoroacetic acid (15.5 mL, 202 mmol) was added to the reaction mixture which was then refluxed for 3 h. The mixture was then concentrated *in vacuo*, and dried under vacuum to give the desired product (5.30 g, 84%) as a yellow solid. ^{1}H NMR (500 MHz, Methanol-d_4) δ 4.92 (s, 2H), 3.26 – 3.15 (m, 2H), 1.86 (qd, J = 6.0, 3.7 Hz, 2H), 1.79 (ddt, J = 7.7, 4.9, 2.9 Hz, 1H). ^{13}C NMR (126 MHz, MeOD) δ 162.8 (q, J = 35.3 Hz), 118.0 (q, J = 292.0 Hz), 50.2, 27.8, 26.1. DART-MS m/z calcd for $C_5H_{13}N_2$ $(M + H)^+$ = 101.10732, found 101.10828.

6,7-Diazabicyclo[3.2.2]non-6-ene synthesis

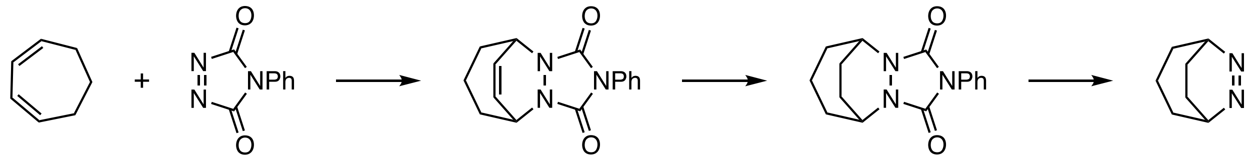

81 **82** **83**

2-Phenyl-6,7,8,9-tetrahydro-1*H*,5*H*-5,9-etheno[1,2,4]triazolo[1,2-*a*][1,2]diazepine-1,3(2*H*)-dione (81): In a scintillation vial equipped with a stir bar, 1,3-cycloheptadiene (100 µL, 0.92 mmol) was added dropwise to a 1.8 M solution of PTAD (161.5 mg, 0.922 mmol) in acetone (520 µL). The color of the solution changed from bright red to yellow upon reaction completion. The reaction was left to stir for 30 min. Hexanes (200 µL) was added to the mixture to precipitate the product. The product (246 mg, 99% yield) was collected a white solid and rinsed with hexanes (1 mL) via vacuum filtration and dried *in vacuo*. ^{1}H NMR (500 MHz, Chloroform-*d*) δ 7.53 – 7.48 (m, 2H), 7.45 (dd, *J* = 8.7, 7.0 Hz, 2H), 7.38 – 7.32 (m, 1H), 6.32 (dd, *J* = 4.7, 3.0 Hz, 2H), 4.99 (tt, *J* = 5.2, 2.7 Hz, 2H), 2.03 – 1.95 (m, 2H), 1.90 (dddd, *J* = 14.7, 10.4, 5.1, 2.6 Hz, 2H), 1.82 (dp, *J* = 14.4, 4.6 Hz, 1H), 1.64 (tdt, *J* = 10.5, 8.6, 5.2 Hz, 1H). ^{13}C NMR (126 MHz, $CDCl_3$) δ 151.3, 132.0, 129.3, 128.8, 128.2, 125.8, 51.2, 29.3, 20.2. APCI-MS m/z calcd for $C_{15}H_{16}N_3O_2$ $(M + H)^+$ = 270.1 , found 270.1.

2-Phenyltetrahydro-1*H*,5*H*-5,9-ethano[1,2,4]triazolo[1,2-*a*][1,2]diazepine-1,3(2*H*)-dione (82): Compound **81** (1.05 g, 3.9 mmol), Pd/C (21.9 mg, 0.20 mmol) and MeOH (13.0 mL, 0.3 M) were combined in a 50 mL round bottom flask equipped with a stir bar. The reaction was purged with H_2 and evacuated 5 times and left to stir vigorously under atmospheric pressure of H_2 for 16 h. The reaction mixture was filtered through Celite and then concentrated *in vacuo*, yielding a white solid (998 mg, 94% yield). ^{1}H NMR (300 MHz, Chloroform-*d*) δ 7.55 (d, *J* = 7.9 Hz, 2H), 7.46 (t, *J* = 7.6 Hz, 2H), 7.36 (d, *J* = 7.4 Hz, 1H), 4.71 (s, 2H), 2.23 – 1.69 (m, 10H). ^{13}C NMR (126 MHz, $CDCl_3$) δ 148.7, 132.0, 129.1, 127.9, 125.7, 48.9, 33.6, 22.7, 19.7. APCI-MS m/z calcd for $C_{15}H_{18}N_3O_2$ $(M + H)^+$ = 272.1, found 272.1.

6,7-diazabicyclo[3.2.2]non-6-ene (83): Cycloadduct **82** (1.093 g, 4.0 mmol) and *i*-PrOH (53.7 mL, 75 mM) were charged to a 250 mL round bottom flask equipped with a stir bar and a reflux condenser. The flask was sonicated and degassed with N_2 for 15 min. Solid KOH pellets (4.294 g, 77 mmol) were added under a flow of N_2. The reaction was heated to reflux for 2 h and then left to cool to room temperature. The mixture was cooled to 0 ºC and diluted with 100 mL of water. At this point the reaction was transferred to a 1 L round bottom flask. The pH of the reaction was then adjusted to 1-2 via dropwise addition of concentrated HCl. Afterwards, the pH was raised to 5-6 via addition of 5 M aqueous NH_4OH. 53.7 mL of 3 M $CuCl_2$ aqueous solution was added to the mixture. 100 mL of hexanes was added to the dark brown solution followed by 160 mL of 5 M aqueous NH_4OH, resulting in the brown solution turning deep blue. The reaction was left to stir

for 30 min at room temperature. The organic layer was collected and the aqueous layer was extracted with methylene chloride (4 x 35 mL). The combined organic layers were dried over Na_2SO_4, filtered, and concentrated *in vacuo*. The concentrate was then diluted with methylene chloride and washed with 1M aqueous HCl (2 x 20 mL). The organic layer was dried with Na_2SO_4 then concentrated *in vacuo*, and purified using column chromatography with 0–25% hexanes/ethyl acetate gradient. The product containing fractions was concentrated *in vacuo*, resulting in a white solid (128 mg, 26 % yield). ^{1}H NMR (500 MHz, Chloroform-*d*) δ 5.04 (t, *J* = 5.4 Hz, 2H), 1.97 – 1.84 (m, 1H), 1.79 (p, *J* = 5.5, 4.9 Hz, 2H), 1.73 – 1.65 (m, 2H), 1.63 (qd, *J* = 4.6, 2.0 Hz, 1H), 1.62 – 1.57 (m, 1H), 1.57 – 1.44 (m, 3H). ^{13}C NMR (126 MHz, $CDCl_3$) δ 62.8, 25.4, 22.2, 19.2. APCI-MS m/z calcd for $C_7H_{13}N_2$ $(M + H)^+$ = 125.1, found 125.1.

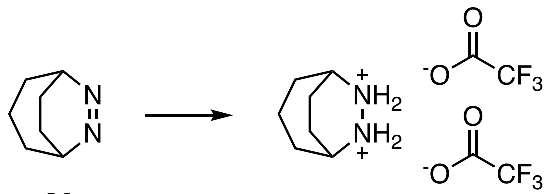

83 **18**

6,7-Diazabicyclo[3.2.2]nonane-6,7-diium 2,2,2-trifluoroacetate (18): Diazene **83** (156.0 mg, 1.3 mmol), Pd/C (6.7 mg, 0.07 mmol) and MeOH (4.2 mL, 0.3 M) were combined in a 25 mL round bottom flask equipped with a stir bar. The reaction was evacuated and purged with H_2 with the sequence repeated 5 times and left to stir vigorously in atmospheric pressure under a balloon of H_2 for 16 h. The reaction mixture was filtered through Celite directly onto trifluoroacetic acid (1.7 mL, 22.7 mmol). The mixture was then concentrated *in vacuo*, yielding a white solid product (251.3 mg, 57% yield). ^{1}H NMR (500 MHz, Methanol-d_4) δ 3.58 (t, *J* = 5.5 Hz, 2H), 2.11 (dtd, *J* = 14.3, 5.4, 3.2 Hz, 2H), 2.02 (dt, *J* = 13.2, 4.5 Hz, 2H), 2.00 – 1.87 (m, 4H), 1.87 – 1.71 (m, 2H). ^{13}C NMR (126 MHz, MeOD) δ 163.2 (q, J = 34.8 Hz), 118.2 (q, J = 292.6 Hz), 51.5, 34.0, 20.8, 20.3. APCI-MS m/z calcd for $C_7H_{15}N_2$ $(M + H)^+$ = 127.1, found 127.1.

Procedure for cycloadduct synthesis

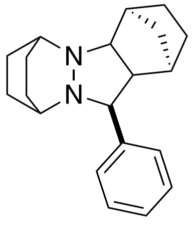

32

11-Phenyldecahydro-1*H*-6,9-ethano-1,4-methanopyridazino[1,2-*a*]indazole (32): [2.2.2] hydrazinium TFA salt **6** (40.0 mg, 0.1 mmol), norbornene (11.1 mg, 0.1 mmol), benzaldehyde (12 µl, 0.1 mmol) and anhydrous MeCN (588 µL, 0.2 M) were combined in a 1 dram vial equipped with a stir bar. After being purged with nitrogen, the reaction was stirred at 60 ºC for 24 hours. The reaction mixture was diluted with DCM (1 mL) and then neutralized with Et_3N (33 µL, 0.2 mmol). The mixture was washed with water (1 mL). The aqueous layer was further washed with DCM for two more times (2 mL). The organic layer was then concentrated *in vacuo*, and purified using column chromatography with 0–45% hexanes/ethyl acetate gradient. The product containing fractions was concentrated *in vacuo*, resulting in a light yellow solid (25.0 mg, 72 % yield). ^{1}H NMR (500 MHz, Chloroform-*d*) δ 7.39 (d, *J* = 7.5 Hz, 2H), 7.31 (t, *J* = 7.5 Hz, 2H), 7.23 (t, *J* = 7.4 Hz, 1H), 3.42 (d, *J* = 7.4 Hz, 1H), 2.92 (d, *J* = 6.7 Hz, 2H), 2.53 (d, *J* = 4.0 Hz, 1H), 2.26 (d, *J* = 2.7 Hz, 0H), 2.13 – 2.06 (m, 2H), 2.06 – 1.84 (m, 4H), 1.79 (tdd, *J* = 9.9, 5.0, 2.6 Hz, 1H), 1.58 (d, *J* = 7.7 Hz, 0H), 1.53 – 1.18 (m, 8H), 1.06 – 0.92 (m, 3H). ^{13}C NMR (126 MHz, $CDCl_3$) δ 141.8, 132.2, 128.3, 126.9, 66.7, 65.8, 60.0, 50.0, 47.7, 39.1, 37.7, 33.2, 28.9, 28.4, 27.2, 25.1, 22.7, 21.2. DART-MS m/z calcd for $C_{20}H_{27}N_2$ $(M + H)^+$ = 295.21688, found 295.21681.

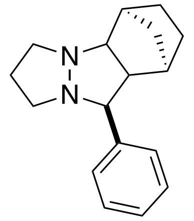

84

9-Phenyloctahydro-1*H*,6*H*-5,8-methanopyrazolo[1,2-*a*]indazole (84): Hydrazinium TFA salt **19** (40.0 mg, 0.13 mmol), norbornene (12.5 mg, 0.13 mmol), benzaldehyde (14 µl, 0.13 mmol) and anhydrous MeCN (666 µL, 0.2 M) were combined in a 1 dram vial equipped with a stir bar. After being purged with nitrogen, the reaction was stirred at 60 ºC for 24 hours. The reaction mixture was neutralized with Et_3N (37 µL, 0.27 mmol). The mixture was then purified using column chromatography with 0–45% hexanes/ethyl acetate gradient. The product containing fractions was concentrated *in vacuo*, resulting in a yellow oil (23.0 mg, 68 % yield). ^{1}H NMR (500 MHz, Chloroform-*d*) δ 7.46 (d, *J* = 7.6 Hz, 2H), 7.33 (t, *J* = 7.6 Hz, 2H), 7.27 (d, *J* = 7.9 Hz, 1H), 3.97 (s, 1H), 3.20 (td, *J* = 10.2, 6.0 Hz, 1H), 2.89 (s, 1H), 2.74 (d, *J* = 7.9 Hz, 1H), 2.55 (t, *J* = 8.1 Hz, 1H), 2.42 (t, *J* = 7.6 Hz, 2H), 2.21 – 2.10 (m, 3H), 2.02 (d, *J* = 15.7 Hz, 1H), 1.96 (dt, *J* = 10.2, 2.2

Hz, 1H), 1.54 (tt, J = 9.3, 3.5 Hz, 2H), 1.11 (dt, J = 10.3, 1.7 Hz, 2H), 1.08 – 1.00 (m, 1H). ^{13}C NMR (126 MHz, $CDCl_3$) δ 139.7, 128.4, 128.2, 128.1, 127.3, 72.31, 69.7, 50.6, 46.5, 40.1, 38.5, 33.1, 28.6, 26.8, 25.5. DART-MS m/z calcd for $C_{17}H_{23}N_2$ $(M + H)^+$ = 255.18558, found 255.18651.

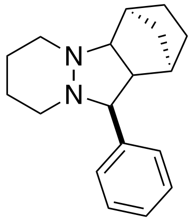

27

11-Phenyldecahydro-1*H*-1,4-methanopyridazino[1,2-*a*]indazole (27): Hydrazinium TFA salt **20** (41.7 mg, 0.13 mmol), norbornene (12.5 mg, 0.13 mmol), benzaldehyde (14 µl, 0.13 mmol) and anhydrous MeCN (664 µL, 0.2 M) were combined in a 1 dram vial equipped with a stir bar. After being purged with nitrogen, the reaction was stirred at 60 ºC for 24 hours. The reaction mixture was neutralized with Et_3N (37 µL, 0.27 mmol). The mixture was then purified using column chromatography with 0–45% hexanes/ethyl acetate gradient. The product containing fractions was concentrated *in vacuo*, resulting in a yellow oil (24.5 mg, 69 % yield). ^{1}H NMR (500 MHz, Chloroform-*d*) δ 7.37 (d, J = 7.5 Hz, 2H), 7.31 (t, J = 7.5 Hz, 2H), 7.25 (d, J = 6.0 Hz, 2H), 3.11 (d, J = 11.0 Hz, 1H), 2.89 (d, J = 8.0 Hz, 1H), 2.77 – 2.70 (m, 1H), 2.52 (d, J = 7.8 Hz, 1H), 2.49 – 2.41 (m, 1H), 2.11 (t, J = 4.4 Hz, 2H), 2.08 – 2.00 (m, 1H), 1.90 (d, J = 9.7 Hz, 2H), 1.72 (s, 1H), 1.67 – 1.60 (m, 1H), 1.57 – 1.38 (m, 2H), 1.06 (d, J = 9.8 Hz, 1H), 1.05 – 0.92 (m, 2H). ^{13}C NMR (126 MHz, $CDCl_3$) δ 141.9, 128.5, 128.2, 127.3, 74.4, 73.3, 56.8, 54.7, 52.8, 39.1, 37.6, 33.6, 28.6, 25.1, 24.7, 24.4. DART-MS m/z calcd for $C_{18}H_{25}N_2$ $(M + H)^+$ = 269.20123, found 269.20215.

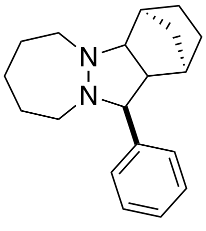

85

12-Phenyldecahydro-1*H*,6*H*-1,4-methano[1,2]diazepino[1,2-*a*]indazole (85): Hydrazinium TFA salt **21** (43.6 mg, 0.13 mmol), norbornene (12.5 mg, 0.13 mmol), benzaldehyde (14 µl, 0.13 mmol) and anhydrous MeCN (664 µL, 0.2 M) were combined in a 1 dram vial equipped with a stir bar. After being purged with nitrogen, the reaction was stirred at 60 ºC for 24 hours. The reaction mixture was neutralized with Et_3N (37 µL, 0.27 mmol). The mixture was then purified using column chromatography with 0–45% hexanes/ethyl acetate gradient. The product containing fractions was concentrated *in vacuo*, resulting in a yellow oil (21.8 mg, 58 % yield). ^{1}H NMR (500 MHz, Chloroform-*d*) δ 7.40 (d, J = 7.5 Hz, 2H), 7.32 (t, J = 7.5 Hz, 2H), 7.28 – 7.21 (m, 1H), 3.14 (dt, J = 13.3, 3.9 Hz, 1H), 3.07 (d, J = 8.1 Hz, 1H), 2.76 (dt, J = 13.4, 4.5 Hz, 1H), 2.71 (s, 0H), 2.57 (d, J = 8.3 Hz, 1H), 2.42 (td, J = 12.9, 12.3, 3.9 Hz, 1H), 2.11 (dd, J = 11.5, 4.1 Hz, 2H), 1.93

(q, J = 8.9, 6.5 Hz, 2H), 1.81 (d, J = 19.3 Hz, 0H), 1.73 – 1.57 (m, 1H), 1.55 – 1.39 (m, 3H), 1.09 (d, J = 9.8 Hz, 1H), 1.02 – 0.89 (m, 2H). ^{13}C NMR (126 MHz, $CDCl_3$) δ 143.3, 128.5, 128.0, 127.1, 76.4, 75.6, 59.8, 57.1, 55.7, 39.9, 38.5, 33.3, 28.3, 27.0, 26.5, 25.8, 25.2. DART-MS m/z calcd for $C_{19}H_{27}N_2$ $(M + H)^+$ = 283.21688, found 283.21768.

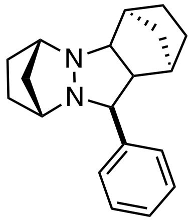

14

11-phenyldecahydro-1*H*-1,4:6,9-dimethanopyridazino[1,2-*a*]indazole (14): [2.2.1] hydrazinium TFA salt **4** (40.0 mg, 0.1 mmol), norbornene (11.5 mg, 0.1 mmol), benzaldehyde (13 µl, 0.1 mmol) and anhydrous MeCN (614 µL, 0.2 M) were combined in a 1 dram vial equipped with a stir bar. After being purged with nitrogen, the reaction was stirred at 60 ºC for 24 hours. The reaction mixture was diluted with DCM (1 mL) and then neutralized with Et_3N (33 µL, 0.2 mmol). The mixture was washed with water (1 mL). The aqueous layer was further washed with DCM for two more times (2 mL). The organic layer was then concentrated *in vacuo*, and purified using column chromatography with 0–10% MeOH/DCM gradient. The product containing fractions was concentrated *in vacuo*, resulting in a light yellow solid (17.0 mg, 49 % yield). ^{1}H NMR (500 MHz, Chloroform-*d*) δ 7.38 (d, J = 4.4 Hz, 4H), 7.32 (h, J = 3.9 Hz, 1H), 4.35 (s, 1H), 3.52 (d, J = 3.7 Hz, 1H), 2.83 (d, J = 9.3 Hz, 2H), 2.48 – 2.32 (m, 1H), 2.27 (d, J = 4.1 Hz, 1H), 1.99 (d, J = 4.0 Hz, 1H), 1.85 (dt, J = 10.7, 2.2 Hz, 1H), 1.69 – 1.58 (m, 1H), 1.58 – 1.39 (m, 6H), 1.30 – 1.16 (m, 2H), 1.12 (dd, J = 17.7, 10.3 Hz, 2H), 1.08 – 1.01 (m, 2H). ^{13}C NMR (126 MHz, $CDCl_3$) δ 143.0, 137.7, 129.7, 128.8, 128.5, 128.1, 127.9, 127.4, 78.4, 77.5, 77.2, 76.9, 73.1, 73.0, 70.7, 70.4, 66.5, 61.4, 61.3, 58.3, 55.9, 42.2, 40.5, 39.2, 37.2, 34.1, 33.7, 33.2, 32.9, 30.7, 29.9, 29.2, 28.3, 28.1, 27.7, 27.5, 26.2. APCI-MS m/z calcd for $C_{19}H_{25}N_2$ $(M + H)^+$ = 281.2, found 281.2.

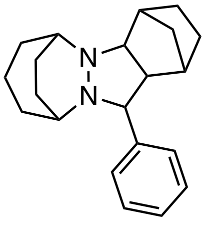

86

12-phenyldecahydro-1*H*,6*H*-6,10-ethano-1,4-methano[1,2]diazepino[1,2-*a*]indazole (86): [3.2.2] hydrazinium TFA salt **18** (40.0 mg, 0.1 mmol), norbornene (10.6 mg, 0.1 mmol), benzaldehyde (12 µl, 0.1 mmol) and anhydrous MeCN (564 µL, 0.2 M) were combined in a 1 dram vial equipped with a stir bar. After being purged with nitrogen, the reaction was stirred at 60 ºC for 24 hours. The reaction mixture was diluted with DCM (1 mL) and then neutralized with Et_3N (33 µL, 0.2 mmol). The mixture was washed with water (1 mL). The aqueous layer was further washed

with DCM for two more times (2 mL). The organic layer was then concentrated *in vacuo*, and purified using column chromatography with 0–25% ethyl acetate/hexanes gradient. The product containing fractions was concentrated *in vacuo*, resulting in a light yellow solid (20.5 mg, 59 % yield). Diastereomer 1: ^{1}H NMR (500 MHz, Chloroform-*d*) δ 7.40 – 7.35 (m, 2H), 7.29 (dd, *J* = 8.3, 6.7 Hz, 2H), 7.25 – 7.19 (m, 1H), 3.31 (d, *J* = 7.4 Hz, 1H), 3.28 (d, *J* = 7.4 Hz, 1H), 2.71 (dt, *J* = 5.5, 2.7 Hz, 1H), 2.67 (d, *J* = 7.5 Hz, 1H), 2.34 (dt, *J* = 14.0, 8.8 Hz, 1H), 2.20 – 2.05 (m, 3H), 2.03 (d, *J* = 4.4 Hz, 1H), 1.82 – 1.70 (m, 2H), 1.62 (ddt, *J* = 17.0, 12.5, 6.3 Hz, 3H), 1.54 – 1.31 (m, 7H), 1.02 – 0.88 (m, 3H). ^{13}C NMR (126 MHz, $CDCl_3$) δ 143.4, 128.6, 128.3, 127.0, 69.7, 65.3, 59.8, 53.6, 51.1, 38.7, 37.5, 36.7, 33.4, 29.2, 25.7, 25.0, 24.4, 22.3, 20.6. Diastereomer 2:^{1}H NMR (500 MHz, Chloroform-*d*) δ 7.39 (d, *J* = 7.5 Hz, 2H), 7.30 (t, *J* = 7.5 Hz, 2H), 7.23 (t, *J* = 7.3 Hz, 1H), 3.11 (d, *J* = 7.3 Hz, 1H), 3.08 – 3.00 (m, 1H), 2.79 (d, *J* = 7.4 Hz, 1H), 2.28 (dq, *J* = 12.1, 5.7 Hz, 1H), 2.24 – 2.14 (m, 1H), 2.12 (d, *J* = 3.7 Hz, 1H), 2.10 – 2.05 (m, 1H), 2.02 (d, *J* = 4.3 Hz, 1H), 1.77 (t, *J* = 7.4 Hz, 1H), 1.73 – 1.65 (m, 2H), 1.60 (dt, *J* = 8.6, 4.1 Hz, 4H), 1.56 – 1.38 (m, 4H), 1.22 (ddt, *J* = 14.0, 7.1, 3.4 Hz, 1H), 1.03 – 0.88 (m, 3H). ^{13}C NMR (126 MHz, $CDCl_3$) δ 142.9, 128.4, 128.1, 127.0, 69.0, 66.6, 58.7, 54.3, 51.7, 39.2, 38.0, 37.6, 33.1, 29.2, 25.1, 25.0, 23.1, 22.5, 20.7. APCI-MS m/z calcd for $C_{21}H_{29}N_2$ $(M + H)^+$ = 309.2, found 309.2.

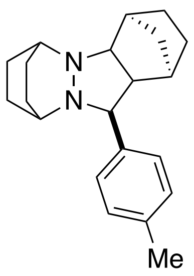

34

11-(*p*-tolyl)decahydro-1*H*-6,9-ethano-1,4-methanopyridazino[1,2-*a*]indazole (34): [2.2.2] hydrazinium TFA salt **6** (40.0 mg, 0.1 mmol), norbornene (11.1 mg, 0.1 mmol), p-tolualdehyde (14 µl, 0.1 mmol) and anhydrous MeCN (588 µL, 0.2 M) were combined in a 1 dram vial equipped with a stir bar. After being purged with nitrogen, the reaction was stirred at 60 ºC for 24 hours. The reaction mixture was diluted with DCM (1 mL) and then neutralized with Et_3N (33 µL, 0.2 mmol). The mixture was washed with water (1 mL). The aqueous layer was further washed with DCM for two more times (2 mL). The organic layer was then concentrated *in vacuo*, and purified using column chromatography with 0–50% hexanes/ethyl acetate gradient. The product containing fractions was concentrated *in vacuo*, resulting in a light-yellow oil (24.0 mg, 66 % yield). ^{1}H NMR (500 MHz, Chloroform-*d*) δ 7.32 (d, *J* = 7.7 Hz, 2H), 7.15 (d, *J* = 7.7 Hz, 2H), 3.44 (d, *J* = 7.3 Hz, 1H), 2.97 (d, *J* = 7.3 Hz, 2H), 2.55 (q, *J* = 3.4 Hz, 1H), 2.37 (s, 3H), 2.30 (ddq, *J* = 8.5, 5.7, 2.7 Hz, 1H), 2.17 – 2.10 (m, 2H), 2.08 – 1.97 (m, 2H), 1.94 (dddt, *J* = 12.2, 9.7, 4.8, 2.2 Hz, 1H), 1.82 (tdd, *J* = 9.7, 4.9, 2.6 Hz, 1H), 1.55 – 1.46 (m, 3H), 1.45 – 1.38 (m, 2H), 1.32 (ddt, *J* = 12.3, 9.5, 2.7 Hz, 1H), 1.08 – 0.96 (m, 3H). ^{13}C NMR (126 MHz, $CDCl_3$) δ 138.7, 136.6, 129.0, 128.2, 66.7, 66.0, 60.0, 50.0, 47.7, 39.2, 37.8, 33.3, 29.1, 28.5, 27.3, 25.2, 22.9, 21.9, 21.2. DART-MS m/z calcd for $C_{21}H_{29}N_2$ $(M + H)^+$ = 309.23253, found 309.23255.

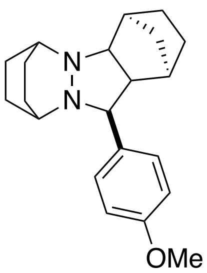

40

11-(4-methoxyphenyl)decahydro-1*H*-6,9-ethano-1,4-methanopyridazino[1,2-*a*]indazole (40): [2.2.2] hydrazinium TFA salt **6** (40.0 mg, 0.1 mmol), norbornene (11.1 mg, 0.1 mmol), anisaldehyde (14 µl, 0.1 mmol) and anhydrous MeCN (588 µL, 0.2 M) were combined in a 1 dram vial equipped with a stir bar. After being purged with nitrogen, the reaction was stirred at 60 ºC for 24 hours. The reaction mixture was diluted with DCM (1 mL) and then neutralized with Et_3N (33 µL, 0.2 mmol). The mixture was washed with water (1 mL). The aqueous layer was further washed with DCM for two more times (2 mL). The organic layer was then concentrated *in vacuo*, and purified using column chromatography with 25–75% hexanes/ethyl acetate gradient. The product containing fractions was concentrated *in vacuo*, resulting in a light-yellow oil (11.9 mg, 31 % yield). ^{1}H NMR (500 MHz, Chloroform-*d*) δ 7.35 – 7.29 (m, 2H), 6.88 – 6.84 (m, 2H), 3.80 (s, 3H), 3.42 (d, *J* = 7.5 Hz, 1H), 2.99 – 2.91 (m, 2H), 2.51 (q, *J* = 3.3, 2.9 Hz, 1H), 2.32 – 2.23 (m, 1H), 2.13 – 2.05 (m, 2H), 2.05 – 1.96 (m, 3H), 1.92 (ddp, *J* = 12.3, 9.7, 2.3 Hz, 1H), 1.77 (dtt, *J* = 14.7, 8.3, 6.6, 3.0 Hz, 1H), 1.54 – 1.44 (m, 2H), 1.44 – 1.35 (m, 2H), 1.30 (ddt, *J* = 12.3, 9.4, 2.8 Hz, 1H), 1.07 – 0.93 (m, 3H). ^{13}C NMR (126 MHz, $CDCl_3$) δ 158.7, 133.4, 129.3, 113.7, 66.4, 66.1, 59.7, 55.2, 50.0, 47.5, 39.2, 37.9, 33.3, 27.1, 25.2, 21.7. DART-MS m/z calcd for $C_{21}H_{29}N_2O$ $(M + H)^+$ = 325.22744, found 325.22743.

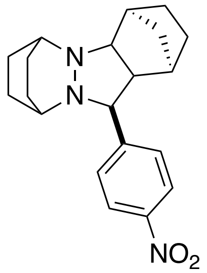

41

11-(4-nitrophenyl)decahydro-1*H*-6,9-ethano-1,4-methanopyridazino[1,2-*a*]indazole(41): [2.2.2] hydrazinium TFA salt **6** (40.0 mg, 0.1 mmol), norbornene (11.1 mg, 0.1 mmol), 4-nitro-benzaldehyde (17.8 mg, 0.1 mmol) and anhydrous MeCN (588 µL, 0.2 M) were combined in a 1 dram vial equipped with a stir bar. After being purged with nitrogen, the reaction was stirred at 60 ºC for 24 hours. The reaction mixture was diluted with DCM (1 mL) and then neutralized with Et_3N (33 µL, 0.2 mmol). The mixture was washed with water (1 mL). The aqueous layer was further washed with DCM for two more times (2 mL). The organic layer was then concentrated *in*

vacuo, and purified using column chromatography with 0–35% hexanes/ethyl acetate gradient. The product containing fractions was concentrated *in vacuo*, resulting in a red-orange solid (21.4 mg, 54 % yield). ^{1}H NMR (400 MHz, Chloroform-*d*) δ 8.15 (d, *J* = 8.7 Hz, 2H), 7.54 (d, *J* = 8.7 Hz, 2H), 3.38 (d, *J* = 7.0 Hz, 1H), 2.95 – 2.87 (m, 1H), 2.82 (d, *J* = 7.2 Hz, 1H), 2.51 (p, *J* = 2.3 Hz, 1H), 2.29 – 2.17 (m, 1H), 2.14 – 1.98 (m, 4H), 1.92 – 1.75 (m, 3H), 1.53 – 1.36 (m, 5H), 1.29 (ddt, *J* = 15.6, 9.6, 4.9 Hz, 1H), 1.06 – 0.87 (m, 3H). ^{13}C NMR (126 MHz, $CDCl_3$) δ 150.6, 147.1, 128.6, 123.6, 65.8, 65.3, 61.3, 50.1, 48.4, 38.8, 37.5, 33.4, 29.1, 28.3, 27.5, 24.9, 22.2, 22.1. DART-MS m/z calcd for $C_{20}H_{26}N_3O_2$ $(M + H)^+$ = 340.20195, found 340.20205.

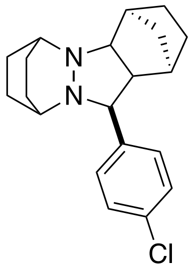

42

11-(4-chlorophenyl)decahydro-1*H*-6,9-ethano-1,4-methanopyridazino[1,2-*a*]indazole (42): [2.2.2] hydrazinium TFA salt **6** (40.0 mg, 0.1 mmol), norbornene (11.1 mg, 0.1 mmol), 4-chloro-benzaldehyde (17.8 mg, 0.1 mmol) and anhydrous MeCN (588 µL, 0.2 M) were combined in a 1 dram vial equipped with a stir bar. After being purged with nitrogen, the reaction was stirred at 60 ºC for 24 hours. The reaction mixture was diluted with DCM (1 mL) and then neutralized with Et_3N (33 µL, 0.2 mmol). The mixture was washed with water (1 mL). The aqueous layer was further washed with DCM for two more times (2 mL). The organic layer was then concentrated *in vacuo*, and purified using column chromatography with 0–50% hexanes/ethyl acetate gradient. The product containing fractions was concentrated *in vacuo*, resulting in (36.5 mg, 94 % yield). ^{1}H NMR (500 MHz, Chloroform-d) δ 7.34 (d, J = 8.3 Hz, 2H), 7.29 (d, J = 8.5 Hz, 2H), 3.35 (d, J = 7.2 Hz, 1H), 2.93 (t, J = 4.2 Hz, 1H), 2.89 (d, J = 7.3 Hz, 1H), 2.52 (dt, J = 5.2, 2.7 Hz, 1H), 2.25 (ddt, J = 15.1, 11.3, 5.8 Hz, 1H), 2.10 (dd, J = 11.6, 3.0 Hz, 2H), 2.06 – 1.97 (m, 2H), 1.95 – 1.84 (m, 2H), 1.79 (tdd, J = 10.4, 5.1, 2.5 Hz, 1H), 1.71 – 1.63 (m, 1H), 1.47 (dtd, J = 14.2, 7.3, 5.6, 3.7 Hz, 3H), 1.43 – 1.37 (m, 2H), 1.31 (ddt, J = 20.1, 10.4, 3.5 Hz, 1H), 0.99 (tdd, J = 16.7, 10.4, 5.7 Hz, 3H). ^{13}C NMR (126 MHz, $CDCl_3$) δ 140.6, 132.5, 130.2, 129.4, 128.4, 127.8, 65.9, 65.5, 60.4, 50.0, 47.9, 38.9, 37.5, 33.2, 29.0, 28.3, 27.3, 25.0, 22.4, 21.9. APCI-MS m/z calcd for $C_{20}H_{26}ClN_2$ $(M + H)^+$ = 329.2, found 329.2.

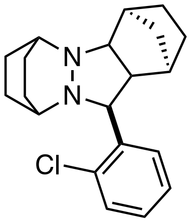

43

11-(2-chlorophenyl)decahydro-1*H*-6,9-ethano-1,4-methanopyridazino[1,2-*a*]indazole (43): [2.2.2] hydrazinium TFA salt **6** (40.0 mg, 0.1 mmol), norbornene (11.1 mg, 0.1 mmol), 2-chloro-benzaldehyde (13 μL, 0.1 mmol) and anhydrous MeCN (588 μL, 0.2 M) were combined in a 1 dram vial equipped with a stir bar. After being purged with nitrogen, the reaction was stirred at 60 ºC for 24 hours. The reaction mixture was diluted with DCM (1 mL) and then neutralized with Et_3N (33 μL, 0.2 mmol). The mixture was washed with water (1 mL). The aqueous layer was further washed with DCM for two more times (2 mL). The organic layer was then concentrated *in vacuo*, and purified using column chromatography with 0–40% hexanes/ethyl acetate gradient. The product containing fractions was concentrated *in vacuo*, resulting in a yellow oil (34.2 mg, 88 % yield). ^{1}H NMR (500 MHz, Chloroform-*d*) δ 7.68 (dd, *J* = 7.8, 1.8 Hz, 1H), 7.32 (d, *J* = 8.0 Hz, 1H), 7.22 (t, *J* = 7.5 Hz, 1H), 7.13 (td, *J* = 7.6, 1.8 Hz, 1H), 3.90 (d, *J* = 7.0 Hz, 1H), 2.89 (dd, *J* = 5.4, 3.1 Hz, 1H), 2.80 (d, *J* = 7.2 Hz, 1H), 2.49 (t, *J* = 3.7 Hz, 1H), 2.37 (d, *J* = 3.6 Hz, 1H), 2.23 (dtd, *J* = 9.9, 5.7, 5.0, 3.1 Hz, 1H), 2.18 (dt, *J* = 9.6, 2.0 Hz, 1H), 2.15 – 2.06 (m, 1H), 2.01 (d, *J* = 3.9 Hz, 1H), 1.89 (qd, *J* = 10.0, 9.4, 4.7 Hz, 1H), 1.81 (ddt, *J* = 11.5, 8.3, 4.6 Hz, 2H), 1.48 – 1.36 (m, 5H), 1.30 (td, *J* = 9.2, 4.5 Hz, 1H), 1.01 (d, *J* = 9.9 Hz, 1H), 0.98 – 0.84 (m, 2H). ^{13}C NMR (126 MHz, $CDCl_3$) δ 140.2, 134.4, 129.4, 129.4, 127.7, 127.0, 65.3, 61.3, 61.2, 50.2, 48.4, 38.7, 37.7, 33.6, 29.4, 28.5, 27.7, 25.1, 22.4, 22.3. APCI-MS m/z calcd for $C_{20}H_{26}ClN_2$ $(M + H)^+$ = 329.2, found 329.2.

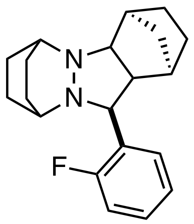

44

11-(2-fluorophenyl)decahydro-1*H*-6,9-ethano-1,4-methanopyridazino[1,2-*a*]indazole (44): [2.2.2] hydrazinium TFA salt **6** (40.0 mg, 0.1 mmol), norbornene (11.1 mg, 0.1 mmol), 2-fluoro-benzaldehyde (12 μL, 0.1 mmol) and anhydrous MeCN (588 μL, 0.2 M) were combined in a 1 dram vial equipped with a stir bar. After being purged with nitrogen, the reaction was stirred at 60 ºC for 24 hours. The reaction mixture was diluted with DCM (1 mL) and then neutralized with Et_3N (33 μL, 0.2 mmol). The mixture was washed with water (1 mL). The aqueous layer was further washed with DCM for two more times (2 mL). The organic layer was then concentrated *in vacuo*, and purified using column chromatography with 0–15% hexanes/ethyl acetate gradient. The product containing fractions was concentrated *in vacuo*, resulting in a yellow oil (27.3 mg, 74 % yield). ^{1}H NMR (500 MHz, Chloroform-*d*) δ 7.60 (td, *J* = 7.5, 1.9 Hz, 1H), 7.20 (tdd, *J* = 7.5, 5.1, 1.9 Hz, 1H), 7.12 (t, *J* = 7.4 Hz, 1H), 7.06 – 6.99 (m, 1H), 3.75 (d, *J* = 7.1 Hz, 1H), 2.93 (dd, *J* = 5.5, 3.1 Hz, 1H), 2.85 (d, *J* = 7.2 Hz, 1H), 2.59 (p, *J* = 2.5 Hz, 1H), 2.31 – 2.22 (m, 2H), 2.18 (dt, *J* = 9.6, 2.1 Hz, 1H), 2.10 (dddt, *J* = 12.4, 9.8, 7.3, 2.4 Hz, 1H), 2.04 (d, *J* = 4.0 Hz, 1H), 1.97 – 1.82 (m, 3H), 1.55 – 1.38 (m, 5H), 1.31 (ddd, *J* = 15.1, 9.3, 2.9 Hz, 1H), 1.06 – 0.93 (m, 3H). ^{13}C NMR (126 MHz, $CDCl_3$) δ 161.7 (d, *J* = 244.5 Hz), 129.2 (d, *J* = 13.4 Hz), 129.0 (d, *J* = 5.1 Hz), 128.0 (d, *J* = 8.3 Hz), 124.3 (d, *J* = 3.4 Hz), 115.3 (d, *J* = 22.7 Hz), 65.4, 60.2, 58.1, 50.1,

48.4, 38.8, 37.7 (d, $J = 1.4$ Hz), 33.6, 29.3, 28.5, 27.5, 25.1, 22.3, 22.2. DART-MS m/z calcd for $C_{20}H_{26}FN_2$ $(M + H)^+ = 313.20746$, found 313.20507.

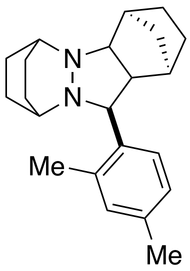

39

11-(2,4-dimethylphenyl)decahydro-1H-6,9-ethano-1,4-methanopyridazino[1,2-a]indazole (39): [2.2.2] hydrazinium TFA salt **6** (40.0 mg, 0.1 mmol), norbornene (11.1 mg, 0.1 mmol), 2,4-dimethylbenzaldehyde (16 µL, 0.1 mmol) and anhydrous MeCN (588 µL, 0.2 M) were combined in a 1 dram vial equipped with a stir bar. After being purged with nitrogen, the reaction was stirred at 60 ºC for 24 hours. The reaction mixture was diluted with DCM (1 mL) and then neutralized with Et_3N (33 µL, 0.2 mmol). The mixture was washed with water (1 mL). The aqueous layer was further washed with DCM for two more times (2 mL). The organic layer was then concentrated *in vacuo*, and purified using column chromatography with 0–50% hexanes/ethyl acetate gradient. The product containing fractions was concentrated *in vacuo*, resulting in (24.2 mg, 64 % yield). ^{1}H NMR (500 MHz, Chloroform-d) δ 7.42 (s, 1H), 6.97 (d, J = 7.9 Hz, 1H), 6.94 (s, 1H), 3.69 (s, 1H), 2.96 (d, J = 7.4 Hz, 1H), 2.93 – 2.86 (m, 1H), 2.34 (d, J = 8.6 Hz, 4H), 2.27 (s, 3H), 2.23 (q, J = 2.9 Hz, 1H), 2.13 – 2.02 (m, 3H), 2.00 (d, J = 4.0 Hz, 1H), 1.98 – 1.84 (m, 2H), 1.72 – 1.63 (m, 1H), 1.58 (s, 2H), 1.51 – 1.36 (m, 2H), 1.36 – 1.30 (m, 1H), 1.26 (ddt, J = 17.9, 7.1, 3.8 Hz, 1H), 1.02 – 0.93 (m, 3H). ^{13}C NMR (126 MHz, $CDCl_3$) δ 137.5, 136.7, 136.2, 131.1, 130.6, 126.8, 50.2, 47.6, 47.6, 39.3, 38.2, 33.4, 29.2, 28.5, 27.1, 25.4, 23.5, 23.5, 23.3, 21.8, 21.1, 20.1. APCI-MS m/z calcd for $C_{22}H_{31}N_2$ $(M + H)^+ = 323.2$, found 323.2.

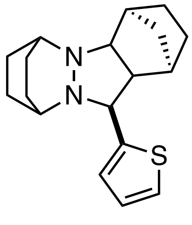

46

11-(thiophen-2-yl)decahydro-1*H*-6,9-ethano-1,4-methanopyridazino[1,2-*a*]indazole (46): [2.2.2] hydrazinium TFA salt **6** (40.0 mg, 0.1 mmol), norbornene (11.1 mg, 0.1 mmol), thiophene-carboxaldehyde (11 µL, 0.1 mmol) and anhydrous MeCN (588 µL, 0.2 M) were combined in a 1 dram vial equipped with a stir bar. After being purged with nitrogen, the reaction was stirred at 60 ºC for 24 hours. The reaction mixture was diluted with DCM (1 mL) and then neutralized with Et_3N (33 µL, 0.2 mmol). The mixture was washed with water (1 mL). The aqueous layer was further washed with DCM for two more times (2 mL). The organic layer was then concentrated *in*

vacuo, and purified using column chromatography with 0–30% hexanes/ethyl acetate gradient. The product containing fractions was concentrated *in vacuo*, resulting in a yellow oil (30.4 mg, 86 % yield). ^{1}H NMR (500 MHz, Chloroform-*d*) δ 7.21 (d, *J* = 5.0 Hz, 1H), 6.98 (d, *J* = 3.4 Hz, 1H), 6.94 (dd, *J* = 5.1, 3.4 Hz, 1H), 3.76 (d, *J* = 7.3 Hz, 1H), 2.99 (d, *J* = 7.5 Hz, 1H), 2.96 – 2.89 (m, 1H), 2.65 (q, *J* = 2.8, 2.1 Hz, 1H), 2.32 – 2.19 (m, 1H), 2.12 (d, *J* = 3.1 Hz, 1H), 2.08 – 1.95 (m, 4H), 1.90 (dddt, *J* = 12.7, 7.4, 5.1, 2.6 Hz, 1H), 1.81 (dddt, *J* = 12.4, 7.5, 4.8, 2.7 Hz, 2H), 1.48 (qt, *J* = 11.1, 5.8 Hz, 3H), 1.42 – 1.29 (m, 3H), 1.05 – 0.95 (m, 3H). ^{13}C NMR (126 MHz, $CDCl_3$) δ 145.52, 126.58, 125.02, 124.64, 66.10, 62.62, 61.14, 50.24, 48.03, 39.34, 38.12, 33.38, 28.93, 28.41, 26.92, 25.23, 23.23, 21.79, 21.41. DART-MS m/z calcd for $C_{18}H_{25}N_2S$ $(M + H)^+$ = 301.16748, found 301.17096.

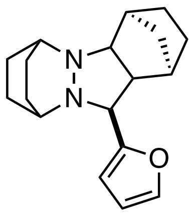

45

11-(furan-2-yl)decahydro-1*H*-6,9-ethano-1,4-methanopyridazino[1,2-*a*]indazole (45): [2.2.2] hydrazinium TFA salt **6** (40.0 mg, 0.1 mmol), norbornene (11.1 mg, 0.1 mmol), furfural (10 µL, 0.1 mmol) and anhydrous MeCN (588 µL, 0.2 M) were combined in a 1 dram vial equipped with a stir bar. After being purged with nitrogen, the reaction was stirred at 60 ºC for 24 hours. The reaction mixture was diluted with DCM (1 mL) and then neutralized with Et_3N (33 µL, 0.2 mmol). The mixture was washed with water (1 mL). The aqueous layer was further washed with DCM for two more times (2 mL). The organic layer was then concentrated *in vacuo*, and purified using column chromatography with 0–10% DCM/methanol gradient. The product containing fractions was concentrated *in vacuo*, resulting in a yellow oil (25.3 mg, 76 % yield). ^{1}H NMR (500 MHz, Chloroform-*d*) δ 7.44 (s, 1H), 6.72 (s, 1H), 6.43 – 6.33 (m, 1H), 5.28 (d, *J* = 2.8 Hz, 1H), 4.81 (s, 1H), 3.55 (d, *J* = 8.3 Hz, 1H), 3.28 (s, 1H), 2.88 (t, *J* = 8.7 Hz, 1H), 2.62 (s, 1H), 2.37 – 2.28 (m, 2H), 2.25 (s, 1H), 2.13 (s, 1H), 1.93 (d, *J* = 11.1 Hz, 2H), 1.82 (t, *J* = 12.7 Hz, 1H), 1.57 (s, 1H), 1.47 (dt, *J* = 22.7, 11.9 Hz, 3H), 1.24 – 1.17 (m, 2H), 1.17 – 1.02 (m, 2H). ^{13}C NMR (126 MHz, $CDCl_3$) δ 145.75, 144.27, 114.81, 111.52, 70.19, 64.86, 53.59, 51.78, 50.25, 50.09, 41.11, 39.98, 33.06, 27.46, 26.31, 25.02, 22.63, 21.05, 17.91. DART-MS m/z calcd for $C_{18}H_{25}N_2O$ $(M + H)^+$ = 285.19614, found 285.19378.

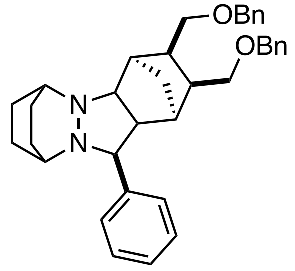

67

2,3-bis((benzyloxy)methyl)-11-phenyldecahydro-1*H*-6,9-ethano-1,4-methanopyridazino[1,2-*a*]indazole (67): [2.2.2] hydrazinium TFA salt **6** (40.0 mg, 0.1 mmol), di-endo-benzyloxymethyl acetal (BOM) norbornene **89** (39.3 mg, 0.1 mmol), benzaldehyde (12 µl, 0.1 mmol) and anhydrous MeCN (588 µL, 0.2 M) were combined in a 1 dram vial equipped with a stir bar. After being purged with nitrogen, the reaction was stirred at 60 ºC for 24 hours. The reaction mixture was diluted with DCM (1 mL) and then neutralized with Et_3N (33 µL, 0.2 mmol). The mixture was washed with water (1 mL). The aqueous layer was further washed with DCM for two more times (2 mL). The organic layer was then concentrated *in vacuo*, and purified using column chromatography with 0–10% DCM/methanol gradient. The product containing fractions was concentrated *in vacuo*, resulting in a yellow oil (47.3 mg, 75 % yield). ^{1}H NMR (400 MHz, Chloroform-*d*) δ 7.42 – 7.27 (m, 9H), 7.25 – 7.15 (m, 4H), 6.99 – 6.91 (m, 2H), 4.50 (d, *J* = 12.1 Hz, 1H), 4.41 (d, *J* = 12.1 Hz, 1H), 4.31 (d, *J* = 12.1 Hz, 1H), 4.21 (d, *J* = 12.0 Hz, 1H), 3.46 (t, *J* = 7.3 Hz, 3H), 3.32 – 3.23 (m, 1H), 3.05 (d, *J* = 7.4 Hz, 1H), 2.89 (s, 1H), 2.63 (s, 1H), 2.41 – 2.29 (m, 3H), 2.21 (d, *J* = 17.1 Hz, 3H), 2.03 (s, 1H), 1.89 (s, 1H), 1.62 (s, 2H), 1.50 – 1.36 (m, 3H), 1.29 (d, *J* = 6.3 Hz, 1H), 1.16 – 1.09 (m, 1H). ^{13}C NMR (126 MHz, $CDCl_3$) δ 141.8, 138.8, 138.5, 128.6, 128.5, 128.5, 127.9, 127.7, 127.7, 127.7, 127.5, 127.0, 73.5, 73.3, 68.0, 67.9, 66.3, 61.1, 59.2, 53.0, 50.1, 48.1, 42.7, 41.4, 40.6, 38.9, 34.9, 28.5, 27.5, 22.6, 22.2, 21.4. APCI-MS m/z calcd for $C_{36}H_{43}N_2O_2$ $(M + H)^+$ = 535.3, found 535.3.

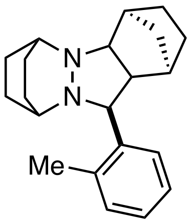

35

11-(*o*-tolyl)decahydro-1*H*-6,9-ethano-1,4-methanopyridazino[1,2-*a*]indazole (35): [2.2.2] hydrazinium TFA salt **6** (40.0 mg, 0.1 mmol), norbornene (11.1 mg, 0.1 mmol), o-tolyaldehyde (17.4 mg, 0.1 mmol) and anhydrous MeCN (588 µL, 0.2 M) were combined in a 1 dram vial equipped with a stir bar. After being purged with nitrogen, the reaction was stirred at 60 ºC for 24 hours. The reaction mixture was diluted with DCM (1 mL) and then neutralized with Et_3N (33 µL, 0.2 mmol). The mixture was washed with water (1 mL). The aqueous layer was further washed with DCM for two more times (2 mL). The organic layer was then concentrated *in vacuo*, and purified

using column chromatography with 0–25% hexanes/ethyl acetate gradient. The product containing fractions was concentrated *in vacuo*, resulting in a white solid (32.6 mg, 90 % yield). ^{1}H NMR (500 MHz, Chloroform-*d*) δ 7.57 (s, 1H), 7.17 (dt, *J* = 7.8, 4.2 Hz, 1H), 7.12 (d, *J* = 3.7 Hz, 2H), 3.73 (s, 1H), 2.98 (d, *J* = 7.4 Hz, 1H), 2.93 (d, *J* = 4.6 Hz, 1H), 2.41 (s, 3H), 2.39 – 2.34 (m, 1H), 2.26 (tt, *J* = 13.8, 10.8, 7.8 Hz, 1H), 2.16 – 2.06 (m, 3H), 2.06 – 1.96 (m, 2H), 1.96 – 1.87 (m, 1H), 1.73 (q, *J* = 9.9, 8.7 Hz, 1H), 1.47 (pt, *J* = 11.0, 4.5 Hz, 3H), 1.40 – 1.28 (m, 3H), 1.05 – 0.96 (m, 3H). ^{13}C NMR (126 MHz, $CDCl_3$) δ 139.6, 137.5, 130.2, 127.6, 126.6, 126.1, 66.3, 62.6, 58.7, 50.2, 47.7, 39.2, 38.2, 33.5, 29.2, 28.5, 27.1, 25.4, 23.3, 21.8, 20.2. DART-MS m/z calcd for $C_{21}H_{29}N_2$ $(M + H)^+$ =309.2, found 309.2.

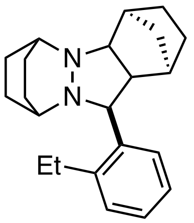

36

11-(2-ethylphenyl)decahydro-1*H*-6,9-ethano-1,4-methanopyridazino[1,2-*a*]indazole (36): [2.2.2] hydrazinium TFA salt **6** (40.0 mg, 0.1 mmol), norbornene (11.1 mg, 0.1 mmol), 2-ethylbenzaldehyde (17.4 mg, 0.1 mmol) and anhydrous MeCN (588 µL, 0.2 M) were combined in a 1 dram vial equipped with a stir bar. After being purged with nitrogen, the reaction was stirred at 60 ºC for 24 hours. The reaction mixture was diluted with DCM (1 mL) and then neutralized with Et_3N (33 µL, 0.2 mmol). The mixture was washed with water (1 mL). The aqueous layer was further washed with DCM for two more times (2 mL). The organic layer was then concentrated *in vacuo*, and purified using column chromatography with 0–25% hexanes/ethyl acetate gradient. The product containing fractions was concentrated *in vacuo*, resulting in a white solid (22.6 mg, 60 % yield). ^{1}H NMR (500 MHz, Chloroform-*d*) δ 7.61 (d, *J* = 7.0 Hz, 1H), 7.20 – 7.10 (m, 3H), 3.74 (d, *J* = 7.5 Hz, 1H), 2.97 (d, *J* = 7.3 Hz, 1H), 2.93 (d, *J* = 4.5 Hz, 1H), 2.75 (dp, *J* = 41.4, 7.2 Hz, 2H), 2.38 (d, *J* = 4.6 Hz, 1H), 2.26 (tt, *J* = 14.0, 10.7, 7.6 Hz, 1H), 2.16 – 2.06 (m, 3H), 2.06 – 1.96 (m, 2H), 1.92 (d, *J* = 12.7 Hz, 1H), 1.72 (d, *J* = 21.3 Hz, 1H), 1.55 – 1.40 (m, 3H), 1.40 – 1.28 (m, 3H), 1.20 (t, *J* = 7.6 Hz, 3H), 1.06 – 0.94 (m, 3H). ^{13}C NMR (126 MHz, $CDCl_3$) δ 143.7, 139.0, 128.6, 128.2, 126.8, 126.1, 66.2, 61.5, 60.0, 50.3, 47.7, 39.2, 38.0, 33.4, 29.3, 28.5, 27.3, 26.1, 25.3, 23.2, 21.8, 16.5. APCI-MS m/z calcd for $C_{22}H_{31}N_2$ $(M + H)^+$ = 323.2, found 323.2.

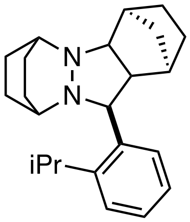

37

11-(2-isopropylphenyl)decahydro-1*H*-6,9-ethano-1,4-methanopyridazino[1,2-*a*]indazole (37): [2.2.2] hydrazinium TFA salt **6** (40.0 mg, 0.1 mmol), norbornene (11.1 mg, 0.1 mmol), 2-isopropylbenzladehyde (17.4 mg, 0.1 mmol) and anhydrous MeCN (588 µL, 0.2 M) were combined in a 1 dram vial equipped with a stir bar. After being purged with nitrogen, the reaction was stirred at 60 ºC for 24 hours. The reaction mixture was diluted with DCM (1 mL) and then neutralized with Et_3N (33 µL, 0.2 mmol). The mixture was washed with water (1 mL). The aqueous layer was further washed with DCM for two more times (2 mL). The organic layer was then concentrated *in vacuo*, and purified using column chromatography with 0–25% hexanes/ethyl acetate gradient. The product containing fractions was concentrated *in vacuo*, resulting in a white solid (19.1 mg, 48 % yield). ^{1}H NMR (500 MHz, Chloroform-*d*) δ 7.63 (d, *J* = 7.7 Hz, 1H), 7.30 (d, *J* = 7.7 Hz, 1H), 7.24 (t, *J* = 7.4 Hz, 1H), 7.19 (t, *J* = 7.4 Hz, 1H), 3.88 (d, *J* = 7.6 Hz, 1H), 3.48 (hept, *J* = 7.0 Hz, 1H), 3.06 (d, *J* = 7.5 Hz, 1H), 2.98 (s, 1H), 2.39 (s, 1H), 2.32 (d, *J* = 13.4 Hz, 1H), 2.20 – 2.11 (m, 2H), 2.11 – 2.05 (m, 2H), 1.98 (t, *J* = 13.3 Hz, 2H), 1.84 – 1.69 (m, 1H), 1.51 (dtd, *J* = 16.9, 8.4, 8.0, 4.1 Hz, 3H), 1.38 (q, *J* = 6.2, 5.1 Hz, 2H), 1.28 (d, *J* = 6.6 Hz, 4H), 1.21 (d, *J* = 6.8 Hz, 3H), 1.04 (q, *J* = 11.0, 9.2 Hz, 3H). ^{13}C NMR (126 MHz, $CDCl_3$) δ 148.3, 137.8, 127.8, 127.0, 125.8, 125.1, 66.5, 61.6, 59.4, 50.2, 47.5, 39.4, 38.0, 33.4, 29.2, 28.4, 28.3, 27.0, 25.4, 24.6, 24.2, 23.5, 21.7. APCI-MS m/z calcd for $C_{23}H_{33}N_2$ $(M + H)^+$ = 337.3, found 337.2.

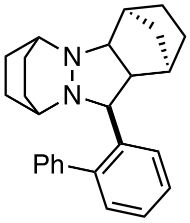

38

11-([1,1'-biphenyl]-2-yl)decahydro-1*H*-6,9-ethano-1,4-methanopyridazino[1,2-*a*]indazole (38): [2.2.2] hydrazinium TFA salt **6** (40.0 mg, 0.1 mmol), norbornene (11.1 mg, 0.1 mmol), 2-isopropylbenzladehyde (17.4 mg, 0.1 mmol) and anhydrous MeCN (588 µL, 0.2 M) were combined in a 1 dram vial equipped with a stir bar. After being purged with nitrogen, the reaction was stirred at 60 ºC for 24 hours. The reaction mixture was diluted with DCM (1 mL) and then neutralized with Et_3N (33 µL, 0.2 mmol). The mixture was washed with water (1 mL). The aqueous layer was further washed with DCM for two more times (2 mL). The organic layer was then concentrated *in vacuo*, and purified using column chromatography with 0–25% hexanes/ethyl acetate gradient. The product containing fractions was concentrated *in vacuo*, resulting in a white solid

(15.5 mg, 36 % yield). ^{1}H NMR (500 MHz, Chloroform-*d*) δ 7.73 (d, *J* = 7.8 Hz, 1H), 7.41 – 7.31 (m, 4H), 7.25 – 7.22 (m, 2H), 7.21 – 7.15 (m, 1H), 3.45 (d, *J* = 7.2 Hz, 1H), 2.81 (d, *J* = 7.8 Hz, 2H), 2.55 (s, 1H), 2.21 (t, *J* = 8.7 Hz, 1H), 1.93 (t, *J* = 7.3 Hz, 1H), 1.88 (d, *J* = 4.4 Hz, 1H), 1.82 – 1.71 (m, 4H), 1.65 (d, *J* = 9.6 Hz, 1H), 1.36 (td, *J* = 11.2, 10.6, 6.4 Hz, 6H), 0.73 (d, *J* = 9.5 Hz, 1H). ^{13}C NMR (126 MHz, $CDCl_3$) δ 143.6, 141.8, 139.4, 129.7, 129.6, 128.7, 128.0, 127.6, 126.9, 126.3, 65.7, 61.6, 60.9, 50.2, 48.0, 38.8, 37.2, 33.2, 29.2, 28.5, 27.8, 25.1, 22.7, 22.2. APCI-MS m/z calcd for $C_{26}H_{31}N_2$ $(M + H)^+$ = 371.2, found 371.2.

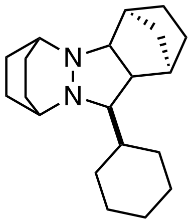

47

11-cyclohexyldecahydro-1*H*-6,9-ethano-1,4-methanopyridazino[1,2-*a*]indazole (47): [2.2.2] hydrazinium TFA salt **6** (40.0 mg, 0.1 mmol), norbornene (11.1 mg, 0.1 mmol), cyclohexacarbox-aldehyde (14.0 µL, 0.1 mmol) and anhydrous MeCN (588 µL, 0.2 M) were combined in a 1 dram vial equipped with a stir bar. After being purged with nitrogen, the reaction was stirred at 60 ºC for 24 hours. The reaction mixture was diluted with DCM (1 mL) and then neutralized with Et_3N (33 µL, 0.2 mmol). The mixture was washed with water (1 mL). The aqueous layer was further washed with DCM for two more times (2 mL). The organic layer was then concentrated *in vacuo*, and purified using column chromatography with 0–10% DCM/methanol gradient on basic alumina support. The product containing fractions was concentrated *in vacuo*, resulting in a clear liquid (20.2 mg, 57 % yield). ^{1}H NMR (300 MHz, Chloroform-*d*) δ 3.19 (d, *J* = 8.2 Hz, 1H), 3.02 (s, 1H), 2.88 (s, 1H), 2.53 (s, 1H), 2.25 (d, *J* = 8.6 Hz, 1H), 1.95 (s, 3H), 1.78 (dd, *J* = 31.4, 19.4 Hz, 11H), 1.60 – 1.36 (m, 5H), 1.24 (t, *J* = 9.1 Hz, 3H), 1.16 – 0.91 (m, 5H). ^{13}C NMR (126 MHz, $CDCl_3$) δ 69.9, 68.6, 54.8, 49.3, 43.4, 40.6, 40.2, 40.1, 33.1, 32.6, 31.1, 29.9, 28.4, 28.1, 26.8, 26.8, 26.4, 26.3, 25.9, 20.5. APCI-MS m/z calcd for $C_{20}H_{33}N_2$ $(M + H)^+$ = 301.3, found 301.2.

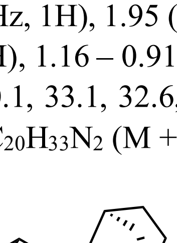

48

11-isobutyldecahydro-1*H*-6,9-ethano-1,4-methanopyridazino[1,2-*a*]indazole (48): [2.2.2] hy-drazinium TFA salt **6** (40.0 mg, 0.1 mmol), norbornene (11.1 mg, 0.1 mmol), isovaleraldehyde (13.0 µL, 0.1 mmol) and anhydrous MeCN (588 µL, 0.2 M) were combined in a 1 dram vial equipped with a stir bar. After being purged with nitrogen, the reaction was stirred at 60 ºC for 24 hours. The reaction mixture was diluted with DCM (1 mL) and then neutralized with Et_3N (33 µL,

0.2 mmol). The mixture was washed with water (1 mL). The aqueous layer was further washed with DCM for two more times (2 mL). The organic layer was then concentrated *in vacuo*, and purified using column chromatography with 0–10% DCM/methanol gradient on basic alumina support. The product containing fractions was concentrated *in vacuo*, resulting in a clear liquid (7.2 mg, 22 % yield). ^{1}H NMR (400 MHz, Chloroform-*d*) δ 3.22 (d, *J* = 8.1 Hz, 1H), 3.01 (s, 1H), 2.87 (s, 2H), 2.27 (d, *J* = 6.5 Hz, 1H), 2.07 – 1.87 (m, 4H), 1.87 – 1.80 (m, 1H), 1.80 – 1.61 (m, 3H), 1.59 – 1.34 (m, 6H), 1.34 – 1.17 (m, 2H), 1.11 – 0.86 (m, 9H). ^{13}C NMR (126 MHz, $CDCl_3$) δ 69.9, 66.0, 54.9, 49.6, 49.2, 41.0, 40.8, 38.6, 32.9, 27.5, 26.2, 25.6, 24.9, 24.0, 21.8, 21.6, 21.0, 17.4. APCI-MS m/z calcd for $C_{18}H_{31}N_2$ $(M + H)^+$ = 275.2, found 275.2.

Substituted norbornene synthesis

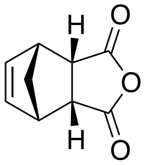

87

(3a*R*,4*S*,7*R*,7a*S*)-3a,4,7,7a-tetrahydro-4,7-methanoisobenzofuran-1,3-dione (87): The compound was prepared and characterized according to the literature.[1)] Maleic anhydride (5.0 g, 0.051 mol) was dissolved in ethyl acetate (6.8 mL, 7.5 M) in a mL round bottom flask. The solution was cooled in an ice bath prior to a dropwise addition of freshly distilled cyclopentadiene (5.0 mL, 0.060 mol) over the span of 90 minutes. The reaction was left to stir overnight at room temperature. The formed white precipitate was collected and wash with hexanes (5 mL x 3) via vacuum filtration. The product was dried *in vacuo* to afford white powder solid (7.87 g, 93% yield). ^{1}H NMR matched the reported spectrum.[1)]

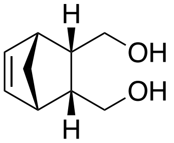

88

((1*R*,2*S*,3*R*,4*S*)-bicyclo[2.2.1]hept-5-ene-2,3-diyl)dimethanol (88): The endo cyclo-adduct **87** of maleic anhydride and cyclopentadiene (3.0 g, 0.018 mol) was dissolved in anhydrous THF (60 mL, 0.3 M) in a flamed dried 250 mL round bottom flask. A stir bar was added and the flask was chilled down to 0 ºC in an ice bath. Lithium aluminum hydride (1.37 g, 0.036 mol) was slowly added into the vigorously stirring mixture. The reaction was left to stir overnight. The reaction was then worked up via Fieser work-up procedure. The mixture was diluted with ether (60 mL) and cooled to 0 ºC, followed by a slow addition of water (1.37 mL). Then, 15% aqueous sodium hydroxide solution (1.37 mL) was added followed by another addition of water (4.11 mL). The reaction was warmed to room temperature and left to stir for an additional 15 minutes. Magnesium sulfate was added to dry the mixture, which was stirred for another 15 minutes. The solution was filtered and concentrate *in vacuo* to yield white solid (2.5 g, 89% yield). ^{1}H NMR matched the reported spectrum.[2)]

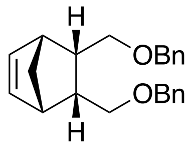

89

(1*R*,4*S*,5*R*,6*S*)-5,6-bis((benzyloxy)methyl)bicyclo[2.2.1]hept-2-ene (89): The compound was prepared and characterized according to the literature. Sodium hydride (0.768 g, 60 wt% in mineral oil, 19.2 mmol) was washed with anhydrous hexanes (2 x 3 mL) in flame dried 100 mL round bottom flask equipped with a stir bar under nitrogen. The sodium hydride was then resuspended in anhydrous THF (32 mL) and cooled to 0 ºC. A solution of the endo diol (**88**) (1.00 g, 6.40 mmol) in anhydrous THF (32 mL, 0.2 M) was added slowly into the NaH suspension. The reaction was left to stir at 0 ºC for one hour, followed by a dropwise addition of benzyl bromide (2.4 mL, 20.5 mmol). The mixture was stirred at 0 ºC for an additional hour and then at room temperature over-night. Water was carefully added to quench the reaction and the mixture was washed with ether (3 x 30 mL). The organic layers were combined and washed with brine (20 mL), followed by drying with $MgSO_4$ and concentrate *in vacuo*. The mixture was purified using column chromatography (0–10% ethyl acetate/ hexanes) to give clear colorless oil (0.4149 g, 20% yield) after concentrated *in vacuo*. ^{1}H NMR matched the reported spectrum.[3)]

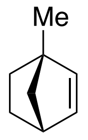

70

Racemic 1-methylbicyclo[2.2.1]hept-2-ene (**70**) was synthesized according to a reported literature procedure.[4)] ^{1}H NMR (500 MHz, Chloroform-*d*) δ 5.99 (dd, *J* = 5.7, 3.1 Hz, 1H), 5.78 (d, *J* = 5.6 Hz, 1H), 2.77 (d, *J* = 3.8 Hz, 1H), 1.76 (ddt, *J* = 11.0, 9.0, 3.6 Hz, 1H), 1.41 (ddd, *J* = 11.0, 9.2, 3.6 Hz, 1H), 1.33 (s, 3H), 1.19 (dq, *J* = 7.4, 2.3 Hz, 1H), 1.15 – 0.96 (m, 3H). ^{13}C NMR (126 MHz, $CDCl_3$) δ 140.0, 135.7, 54.7, 49.6, 43.0, 32.3, 27.8, 19.0. DART-MS m/z calcd for C_8H_{13} (M + H)$^+$ = 109.10118, found 109.10192.

Procedure for cycloreversion

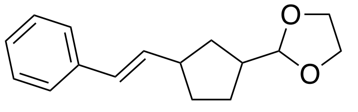

49

(*E*)-2-(3-styrylcyclopentyl)-1,3-dioxolane (49): In a 10 mL Schlenk tube, cycloadduct **32** (43.2 mg, 0.14 mmol), scandium(III) triflate (13.4 mg, 0.027 mmol) were dissolved in acetonitrile (0.68 mL, 0.2 M). Trifluoroacetic acid (21 µL, 0.27 mmol), H_2O (49 µL, 2.7 mmol), and ethylene glycol (152 µL, 2.7 mmol) were added to the reaction mixture using a micropipette. After being purged with nitrogen for around 1 min, the tube was sealed with a Teflon stopper and the reaction was stirred at 140 ºC for 16 hours. The mixture was cooled down to room temperature. Additional trifluoroacetic acid (21 µL, 0.27 mmol) and ethylene glycol (152 µL, 2.7 mmol) were added to the mixture which was stirred for 1 hour at 140 ºC to convert all of the opened product to the acetal form. The reaction mixture was directly purified using column chromatography with 0–15% hexanes/ethyl acetate gradient. The product containing fractions were concentrated *in vacuo*, resulting in (27.9 mg, 78 % yield). ^{1}H NMR (500 MHz, Chloroform-d) δ 7.36 (dt, J = 8.2, 1.5 Hz, 2H), 7.31 (dd, J = 8.5, 6.9 Hz, 2H), 7.24 – 7.18 (m, 1H), 6.41 (dd, J = 15.9, 5.5 Hz, 1H), 6.22 (td, J = 15.7, 7.6 Hz, 1H), 4.79 (d, J = 5.4 Hz, 1H), 4.06 – 3.95 (m, 2H), 3.95 – 3.85 (m, 2H), 2.81 – 2.61 (m, 1H), 2.43 – 2.23 (m, 1H), 2.09 – 1.77 (m, 3H), 1.76 – 1.61 (m, 1H), 1.56 – 1.34 (m, 2H). ^{13}C NMR (126 MHz, $CDCl_3$) δ 137.8, 137.8, 134.9, 134.7, 128.5, 128.3, 128.3, 126.8, 126.0, 107.4, 107.4, 65.1, 65.1, 65.1, 44.0, 43.0, 42.8, 41.9, 35.0, 33.9, 33.6, 32.6, 29.7, 27.2, 26.5. DART-MS m/z calcd for $C_{16}H_{21}O_2$ $(M + H)^+$ = 245.15361, found 245.15364.

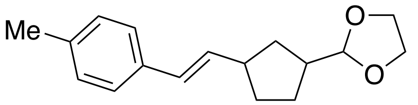

52

(*E*)-2-(3-(4-methylstyryl)cyclopentyl)-1,3-dioxolane (52): In a 10 mL Schlenk tube, cycloadduct **34** (30.9 mg, 0.10 mmol), scandium(III) triflate (9.9 mg, 0.020 mmol) were dissolved in acetonitrile (0.50 mL, 0.2 M). Trifluoroacetic acid (15 µL, 0.20 mmol), H_2O (36 µL, 2.0 mmol), and ethylene glycol (112 µL, 2.0 mmol) were added to the reaction mixture using a micropipette. After being purged with nitrogen for around 1 min, the tube was sealed with a Teflon stopper and the reaction was stirred at 140 ºC for 16 hours. The mixture was cooled down to room temperature. Additional trifluoroacetic acid (15 µL, 0.20 mmol) and ethylene glycol (112 µL, 2.0 mmol) were added to the mixture which was stirred for 1 hour at 140 ºC to convert all of the opened product to the acetal form. The reaction mixture was directly purified using column chromatography with 0–15% hexanes/ethyl acetate gradient. The product containing fractions were concentrated *in vacuo*, resulting in (13.5 mg, 52 % yield). ^{1}H NMR (500 MHz, Chloroform-d) δ 7.23 (d, *J* = 7.8 Hz, 2H), 7.09 (d, *J* = 7.8 Hz, 2H), 6.35 (dd, *J* = 15.9, 5.5 Hz, 1H), 6.13 (td, *J* = 15.5, 7.6 Hz, 1H), 4.76 (d, *J* = 5.3 Hz, 1H), 4.05 – 3.93 (m, 2H), 3.92 – 3.84 (m, 2H), 2.75 – 2.59 (m, 1H), 2.32 (s, 3H), 2.27 (q, *J* = 8.9 Hz, 0H), 2.00 (dd, *J* = 13.1, 6.7 Hz, 1H), 1.96 – 1.74 (m, 2H), 1.72 – 1.23 (m, 3H). ^{13}C NMR (126 MHz, $CDCl_3$) δ 136.6, 135.1, 135.1, 134.0, 133.8, 129.3, 128.3, 128.2, 126.0, 107.6,

107.6, 65.2, 65.2, 65.2, 44.1, 43.1, 42.9, 42.0, 35.1, 34.1, 33.7, 32.8, 27.3, 26.6, 21.3. DART-MS m/z calcd for $C_{17}H_{23}O_2$ $(M + H)^+$ = 259.16926, found 259.17023.

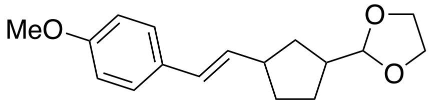

58

(*E*)-2-(3-(4-methoxystyryl)cyclopentyl)-1,3-dioxolane (58): In a 10 mL Schlenk tube, cycloadduct **40** (33.6 mg, 0.10 mmol), scandium(III) triflate (10.2 mg, 0.021 mmol) were dissolved in acetonitrile (0.52 mL, 0.2 M). Trifluoroacetic acid (16 µL, 0.21 mmol), H_2O (37 µL, 2.1 mmol), and ethylene glycol (116 µL, 2.1 mmol) were added to the reaction mixture using a micropipette. After being purged with nitrogen for around 1 min, the tube was sealed with a Teflon stopper and the reaction was stirred at 140 ºC for 16 hours. The mixture was cooled down to room temperature. Additional trifluoroacetic acid (16 µL, 0.21 mmol) and ethylene glycol (116 µL, 2.1 mmol) were added to the mixture which was stirred for 1 hour at 140 ºC to convert all of the opened product to the acetal form. The reaction mixture was directly purified using column chromatography with 0–15% hexanes/ethyl acetate gradient. The product containing fractions were concentrated *in vacuo*, resulting in (18.6 mg, 66 % yield). ^{1}H NMR (500 MHz, Chloroform-d) δ 7.27 (d, *J* = 8.8 Hz, 2H), 6.87 – 6.79 (m, 2H), 6.33 (dd, *J* = 15.8, 5.7 Hz, 1H), 6.05 (td, *J* = 15.5, 7.6 Hz, 1H), 4.76 (d, *J* = 5.4 Hz, 1H), 3.98 (dt, *J* = 5.7, 1.8 Hz, 2H), 3.92 – 3.84 (m, 2H), 3.80 (s, 3H), 2.79 – 2.56 (m, 1H), 2.42 – 2.19 (m, 1H), 2.07 – 1.95 (m, 1H), 1.95 – 1.74 (m, 1H), 1.72 – 1.19 (m, 2H). ^{13}C NMR (126 MHz, $CDCl_3$) δ 158.8, 132.9, 132.7, 130.8, 130.8, 127.8, 127.7, 127.2, 114.0, 107.6, 107.6, 65.2, 65.2, 65.2, 65.2, 55.4, 44.1, 43.1, 42.9, 42.0, 35.2, 34.1, 33.8, 32.8, 27.3, 26.6. DART-MS m/z calcd for $C_{17}H_{23}O_3$ $(M + H)^+$ = 275.16417, found 275.16513.

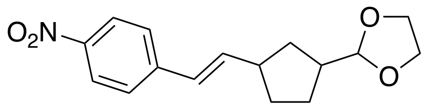

59

(*E*)-2-(3-(4-nitrostyryl)cyclopentyl)-1,3-dioxolane (59): In a 10 mL Schlenk tube, cycloadduct **41** (32.6 mg, 0.10 mmol), scandium(III) triflate (9.5 mg, 0.019 mmol) were dissolved in acetonitrile (0.48 mL, 0.2 M). Trifluoroacetic acid (15 µL, 0.19 mmol), H_2O (35 µL, 1.9 mmol), and ethylene glycol (107 µL, 1.9 mmol) were added to the reaction mixture using a micropipette. After being purged with nitrogen for around 1 min, the tube was sealed with a Teflon stopper and the reaction was stirred at 140 ºC for 16 hours. The mixture was cooled down to room temperature. Additional trifluoroacetic acid (15 µL, 0.19 mmol) and ethylene glycol (107 µL, 1.9 mmol) were added to the mixture which was stirred for 1 hour at 140 ºC to convert all of the opened product to the acetal form. The reaction mixture was directly purified using column chromatography with 0–15% hexanes/ethyl acetate gradient. The product containing fractions were concentrated *in vacuo*, resulting in (11.2 mg, 40 % yield). ^{1}H NMR (500 MHz, Chloroform-d) δ 8.19 – 8.11 (m, 2H), 7.49 – 7.42 (m, 2H), 6.50 – 6.33 (m, 2H), 4.78 (dd, *J* = 5.2, 1.8 Hz, 1H), 4.05 – 3.94 (m, 2H), 3.94 – 3.83 (m, 2H), 2.84 – 2.65 (m, 1H), 2.43 – 2.26 (m, 1H), 2.08 – 1.35 (m, 8H). ^{13}C NMR (126 MHz, $CDCl_3$) δ 146.6, 144.5, 140.3, 140.2, 126.9, 126.8, 126.5, 124.1, 107.3, 65.3, 65.3, 65.2, 65.2, 44.2,

43.3, 42.9, 42.0, 34.7, 33.8, 33.5, 32.6, 27.3, 26.6. DART-MS m/z calcd for $C_{16}H_{20}NO_4$ $(M + H)^+$ = 290.13868, found 290.13971.

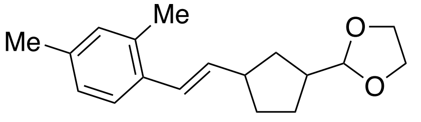

57

(*E*)-2-(3-(2,4-dimethylstyryl)cyclopentyl)-1,3-dioxolane (57): In a 10 mL Schlenk tube, cycloadduct **39** (35.4 mg, 0.11 mmol), scandium(III) triflate (10.8 mg, 0.022 mmol) were dissolved in acetonitrile (0.55 mL, 0.2 M). Trifluoroacetic acid (17 µL, 0.22 mmol), H_2O (40 µL, 2.2 mmol), and ethylene glycol (123 µL, 2.2 mmol) were added to the reaction mixture using a micropipette. After being purged with nitrogen for around 1 min, the tube was sealed with a Teflon stopper and the reaction was stirred at 140 ºC for 16 hours. The mixture was cooled down to room temperature. Additional trifluoroacetic acid (17 µL, 0.22 mmol) and ethylene glycol (123 µL, 2.2 mmol) were added to the mixture which was stirred for 1 hour at 140 ºC to convert all of the opened product to the acetal form. The reaction mixture was directly purified using column chromatography with 0–15% hexanes/ethyl acetate gradient. The product containing fractions were concentrated *in vacuo*, resulting in (24.0 mg, 80 % yield). ^{1}H NMR (500 MHz, Chloroform-d) δ 7.31 (dd, *J* = 7.8, 1.7 Hz, 1H), 6.95 (d, *J* = 9.2 Hz, 2H), 6.54 (dd, *J* = 15.6, 3.8 Hz, 1H), 6.02 (td, *J* = 15.8, 7.6 Hz, 1H), 4.77 (dd, *J* = 5.4, 2.9 Hz, 1H), 3.99 (dq, *J* = 4.4, 2.5 Hz, 2H), 3.93 – 3.82 (m, 2H), 2.78 – 2.61 (m, 1H), 2.29 (s, 6H), 2.02 (dt, *J* = 13.6, 7.2 Hz, 1H), 1.97 – 1.76 (m, 2H), 1.73 – 1.33 (m, 3H). ^{13}C NMR (126 MHz, $CDCl_3$) δ 136.5, 135.4, 135.3, 134.9, 134.1, 134.1, 131.1, 126.8, 126.0, 126.0, 125.4, 125.4, 107.6, 107.6, 65.2, 65.2, 65.2, 44.4, 43.5, 43.0, 42.0, 35.3, 34.2, 33.9, 32.9, 27.3, 26.6, 21.1, 19.9. DART-MS m/z calcd for $C_{18}H_{25}O_2$ $(M + H)^+$ = 273.18491, found 273.18573.

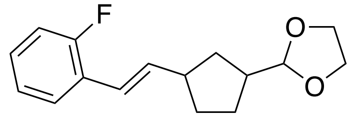

62

(*E*)-2-(3-(2-fluorostyryl)cyclopentyl)-1,3-dioxolane (62): In a 10 mL Schlenk tube, cycloadduct **44** (23.4 mg, 0.075 mmol), scandium(III) triflate (7.4 mg, 0.015 mmol) were dissolved in acetonitrile (0.37 mL, 0.2 M). Trifluoroacetic acid (11 µL, 0.15 mmol), H_2O (27 µL, 1.5 mmol), and ethylene glycol (84 µL, 1.5 mmol) were added to the reaction mixture using a micropipette. After being purged with nitrogen for around 1 min, the tube was sealed with a Teflon stopper and the reaction was stirred at 140 ºC for 16 hours. The mixture was cooled down to room temperature. Additional trifluoroacetic acid (11 µL, 0.15 mmol) and ethylene glycol (84 µL, 1.5 mmol) were added to the mixture which was stirred for 1 hour at 140 ºC to convert all of the opened product to the acetal form. The reaction mixture was directly purified using column chromatography with 0–15% hexanes/ethyl acetate gradient. The product containing fractions were concentrated *in vacuo*, resulting in (15.2 mg, 77 % yield). ^{1}H NMR (500 MHz, Chloroform-d) δ 7.42 (tt, *J* = 7.8, 1.7 Hz, 1H), 7.15 (tdd, *J* = 7.3, 5.1, 1.8 Hz, 1H), 7.06 (td, *J* = 7.5, 1.3 Hz, 1H), 7.00 (ddd, *J* = 9.7, 8.3, 1.3 Hz, 1H), 6.54 (dd, *J* = 16.0, 4.7 Hz, 1H), 6.26 (td, *J* = 16.0, 7.7 Hz, 1H), 4.77 (d, *J* = 5.3 Hz, 1H),

4.06 – 3.93 (m, 2H), 3.93 – 3.82 (m, 2H), 2.80 – 2.63 (m, 1H), 2.40 – 2.24 (m, 1H), 2.02 (dt, *J* = 13.8, 7.2 Hz, 1H), 1.98 – 1.84 (m, 1H), 1.84 – 1.76 (m, 0H), 1.74 – 1.32 (m, 4H). ^{13}C NMR (126 MHz, $CDCl_3$) δ 160.1 (d, *J* = 248 Hz), 137.5 (dd, *J* = 22.3, 4.2 Hz), 128.1 (d, *J* = 8.2 Hz), 127.1 (dd, *J* = 4.1, 1.1 Hz), 125.6 (dd, *J* = 12.3, 1.6 Hz), 124.1 (d, *J* = 3.5 Hz), 120.8 (dd, *J* = 5.4, 3.8 Hz), 115.72 (d, *J* = 22.2 Hz), 107.48 (d, *J* = 3.8 Hz), 65.3, 65.2, 65.2, 44.5, 43.5, 42.9, 42.0, 35.0, 34.0, 33.7, 32.7, 27.3, 26.6. DART-MS m/z calcd for $C_{16}H_{20}FO_2$ $(M + H)^+$ = 263.14418, found 263.14517.

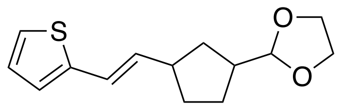

64

(*E*)-2-(3-(2-(thiophen-2-yl)vinyl)cyclopentyl)-1,3-dioxolane (64): In a 10 mL Schlenk tube, cycloadduct **46** (30.0 mg, 100 µmol), scandium(III) triflate (9.8 mg, 20 µmol) were dissolved in acetonitrile (0.50 mL, 0.2 M). Trifluoroacetic acid (15 µL, 200 µmol), H_2O (36 µL, 2.0 mmol), and ethylene glycol (112 µL, 2.0 mmol) were added to the reaction mixture using a micropipette. After being purged with nitrogen for around 1 min, the tube was sealed with a Teflon stopper and the reaction was stirred at 140 ºC for 16 hours. The mixture was cooled down to room temperature. Additional trifluoroacetic acid (15 µL, 200 µmol) and ethylene glycol (112 µL, 2.0 mmol) were added to the mixture which was stirred for 1 hour at 140 ºC to convert all of the opened product to the acetal form. The reaction mixture was directly purified using column chromatography with 0–15% hexanes/ethyl acetate gradient. The product containing fractions were concentrated *in vacuo*, resulting in (14.0 mg, 56 % yield). ^{1}H NMR (500 MHz, Chloroform-*d*) δ 7.08 (d, *J* = 5.1 Hz, 1H), 6.92 (dd, *J* = 5.1, 3.5 Hz, 1H), 6.86 (d, *J* = 3.5 Hz, 1H), 6.50 (dd, *J* = 15.7, 6.5 Hz, 1H), 6.04 (td, *J* = 15.9, 7.6 Hz, 1H), 4.76 (d, *J* = 5.3 Hz, 1H), 4.01 – 3.95 (m, 2H), 3.90 – 3.84 (m, 2H), 2.73 – 2.55 (m, 1H), 2.39 – 2.21 (m, 1H), 2.06 – 1.74 (m, 2H), 1.73 – 1.52 (m, 2H), 1.50 – 1.30 (m, 1H), 0.95 – 0.78 (m, 1H). ^{13}C NMR (126 MHz, $CDCl_3$) δ 143.2, 143.2, 135.0, 134.8, 127.3, 124.4, 123.3, 123.2, 121.8, 121.7, 107.5, 107.5, 65.3, 65.2, 65.2, 65.2, 44.0, 43.0, 42.9, 42.0, 34.9, 33.9, 33.6, 32.6, 27.2, 26.6. DART-MS m/z calcd for $C_{14}H_{19}O_2S$ $(M + H)^+$ = 251.11003, found 251.11086.

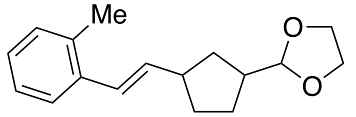

53

(*E*)-2-(3-(2-methylstyryl)cyclopentyl)-1,3-dioxolane (53): In a 10 mL Schlenk tube, cycloadduct **35** (40.2 mg, 0.13 mmol), scandium(III) triflate (12.8 mg, 0.026 mmol) were dissolved in acetonitrile (0.65 mL, 0.2 M). Trifluoroacetic acid (20 µL, 0.26 mmol), H_2O (47 µL, 2.6 mmol), and ethylene glycol (146 µL, 2.6 mmol) were added to the reaction mixture using a micropipette. After being purged with nitrogen for around 1 min, the tube was sealed with a Teflon stopper and the reaction was stirred at 140 ºC for 16 hours. The mixture was cooled down to room temperature. Additional trifluoroacetic acid (20 µL, 0.26 mmol) and ethylene glycol (146 µL, 2.6 mmol) were

added to the mixture which was stirred for 1 hour at 140 ºC to convert all of the opened product to the acetal form. The reaction mixture was directly purified using column chromatography with 0–15% hexanes/ethyl acetate gradient. The product containing fractions were concentrated *in vacuo*, resulting in (16.0 mg, 48 % yield). ^{1}H NMR (500 MHz, Chloroform-*d*) δ 7.45 – 7.38 (m, 1H), 7.17 – 7.07 (m, 3H), 6.57 (dd, *J* = 15.6, 3.9 Hz, 1H), 6.06 (td, *J* = 16.0, 7.7 Hz, 1H), 4.77 (dd, *J* = 5.4, 2.8 Hz, 1H), 4.04 – 3.93 (m, 2H), 3.93 – 3.83 (m, 2H), 2.70 (ddt, *J* = 27.8, 10.2, 7.6 Hz, 1H), 2.40 – 2.22 (m, 4H), 2.07 – 1.75 (m, 2H), 1.75 – 1.32 (m, 4H). ^{13}C NMR (126 MHz, $CDCl_3$) δ 136.9, 136.9, 136.2, 136.1, 135.0, 130.2, 126.8, 126.1, 126.0, 126.0, 125.4, 125.4, 107.5, 107.4, 65.1, 65.1, 65.1, 44.3, 43.3, 42.9, 41.9, 35.1, 34.1, 33.7, 32.7, 27.2, 26.5, 19.8. DART-MS m/z calcd for $C_{17}H_{23}O_2$ $(M + H)^+$ = 259.16926, found 259.17023.

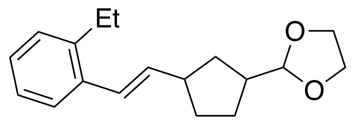

54

(*E*)-2-(3-(2-ethylstyryl)cyclopentyl)-1,3-dioxolane (54): In a 10 mL Schlenk tube, cycloadduct **36** (24.2 mg, 0.075 mmol), scandium(III) triflate (7.4 mg, 0.015 mmol) were dissolved in acetonitrile (0.38 mL, 0.2 M). Trifluoroacetic acid (11 µL, 0.15 mmol), H_2O (27 µL, 1.5 mmol), and ethylene glycol (84 µL, 1.5 mmol) were added to the reaction mixture using a micropipette. After being purged with nitrogen for around 1 min, the tube was sealed with a Teflon stopper and the reaction was stirred at 140 ºC for 16 hours. The mixture was cooled down to room temperature. Additional trifluoroacetic acid (11 µL, 0.15 mmol) and ethylene glycol (84 µL, 1.5 mmol) were added to the mixture which was stirred for 1 hour at 140 ºC to convert all of the opened product to the acetal form. The reaction mixture was directly purified using column chromatography with 0–15% hexanes/ethyl acetate gradient. The product containing fractions were concentrated *in vacuo*, resulting in (13.2 mg, 65 % yield). ^{1}H NMR (500 MHz, Chloroform-*d*) δ 7.42 (dt, *J* = 6.8, 2.1 Hz, 1H), 7.14 (dd, *J* = 5.9, 3.7 Hz, 3H), 6.67 – 6.59 (m, 1H), 6.06 (td, *J* = 15.2, 7.6 Hz, 1H), 4.77 (dd, *J* = 5.5, 4.3 Hz, 1H), 4.03 – 3.94 (m, 2H), 3.92 – 3.82 (m, 2H), 2.70 (dqd, *J* = 15.1, 8.2, 7.6, 1.6 Hz, 3H), 2.40 – 2.22 (m, 1H), 2.07 – 1.75 (m, 3H), 1.74 – 1.24 (m, 5H), 1.19 (t, *J* = 7.6 Hz, 3H), 0.91 (dt, *J* = 12.1, 7.2 Hz, 1H). ^{13}C NMR (126 MHz, $CDCl_3$) δ 141.2, 141.2, 136.5, 136.4, 136.4, 136.3, 128.7, 128.7, 127.1, 126.1, 126.0, 125.9, 125.9, 125.9, 107.6, 107.6, 65.2, 65.2, 65.2, 44.4, 43.5, 43.0, 42.0, 35.2, 34.2, 33.8, 32.8, 27.3, 26.7, 26.5, 26.5, 15.4, 15.3. DART-MS m/z calcd for $C_{18}H_{25}O_2$ $(M + H)^+$ = 273.18491, found 273.18597.

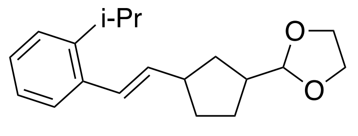

55

(*E*)-2-(3-(2-isopropylstyryl)cyclopentyl)-1,3-dioxolane (55): In a 10 mL Schlenk tube, cycloadduct **37** (31.1 mg, 0.092 mmol), scandium(III) triflate (9.1 mg, 0.018 mmol) were dissolved in acetonitrile (0.46 mL, 0.2 M). Trifluoroacetic acid (14 µL, 0.18 mmol), H_2O (33 µL, 1.8 mmol), and ethylene glycol (103 µL, 1.8 mmol) were added to the reaction mixture using a micropipette.

After being purged with nitrogen for around 1 min, the tube was sealed with a Teflon stopper and the reaction was stirred at 140 ºC for 16 hours. The mixture was cooled down to room temperature. Additional trifluoroacetic acid (14 µL, 0.18 mmol) and ethylene glycol (103 µL, 1.8 mmol) were added to the mixture which was stirred for 1 hour at 140 ºC to convert all of the opened product to the acetal form. The reaction mixture was directly purified using column chromatography with 0–15% hexanes/ethyl acetate gradient. The product containing fractions were concentrated *in vacuo*, resulting in (9.8 mg, 37 % yield). ^{1}H NMR (500 MHz, Chloroform-*d*) δ 7.38 (dd, *J* = 7.7, 1.8 Hz, 1H), 7.25 – 7.17 (m, 3H), 7.13 (td, *J* = 7.4, 1.6 Hz, 1H), 6.71 (dd, *J* = 15.6, 4.2 Hz, 1H), 6.01 (td, *J* = 15.2, 7.6 Hz, 1H), 4.77 (dd, *J* = 5.4, 4.4 Hz, 1H), 4.04 – 3.93 (m, 2H), 3.93 – 3.83 (m, 2H), 3.23 (heptd, *J* = 6.9, 2.4 Hz, 1H), 2.80 – 2.63 (m, 1H), 2.40 – 2.20 (m, 1H), 2.03 (dt, *J* = 12.1, 7.1 Hz, 1H), 1.96 – 1.76 (m, 2H), 1.74 – 1.25 (m, 5H), 1.22 (d, *J* = 6.9 Hz, 6H). ^{13}C NMR (126 MHz, $CDCl_3$) δ 145.5, 136.9, 136.8, 136.3, 136.2, 127.3, 126.4, 126.4, 126.2, 126.1, 125.9, 124.9, 107.6, 107.6, 65.3, 65.2, 65.2, 65.2, 44.4, 43.5, 43.0, 42.0, 35.2, 34.2, 33.8, 32.8, 29.2, 29.1, 27.3, 26.7, 23.5, 23.4. DART-MS m/z calcd for $C_{19}H_{27}O_2$ $(M + H)^+$ = 287.20056, found 287.20154.

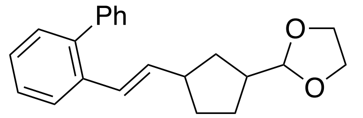

56

(*E*)-2-(3-(2-([1,1'-biphenyl]-2-yl)vinyl)cyclopentyl)-1,3-dioxolane (56): In a 10 mL Schlenk tube, cycloadduct **38** (16.5 mg, 0.042 mmol), scandium(III) triflate (4.4 mg, 8.9 µmol) were dissolved in acetonitrile (0.22 mL, 0.2 M). Trifluoroacetic acid (7 µL, 0.089 mmol), H_2O (16 µL, 0.89 mmol), and ethylene glycol (50 µL, 0.89 mmol) were added to the reaction mixture using a micropipette. After being purged with nitrogen for around 1 min, the tube was sealed with a Teflon stopper and the reaction was stirred at 140 ºC for 16 hours. The mixture was cooled down to room temperature. Additional trifluoroacetic acid (7 µL, 0.089 mmol) and ethylene glycol (50 µL, 0.89 mmol) were added to the mixture which was stirred for 1 hour at 140 ºC to convert all of the opened product to the acetal form. The reaction mixture was directly purified using column chromatography with 0–15% hexanes/ethyl acetate gradient. The product containing fractions were concentrated *in vacuo*, resulting in (12.8 mg, 90 % yield). ^{1}H NMR (500 MHz, Chloroform-*d*) δ 7.57 (dd, *J* = 7.2, 1.6 Hz, 1H), 7.43 – 7.37 (m, 2H), 7.37 – 7.32 (m, 3H), 7.31 – 7.25 (m, 4H), 6.36 (dd, *J* = 15.8, 6.9 Hz, 1H), 6.10 (ddd, *J* = 15.7, 12.2, 7.7 Hz, 1H), 4.72 (dd, *J* = 5.5, 4.4 Hz, 1H), 4.02 – 3.91 (m, 2H), 3.85 (tdd, *J* = 6.7, 4.9, 2.4 Hz, 2H), 2.68 – 2.50 (m, 1H), 2.34 – 2.15 (m, 1H), 1.99 – 1.18 (m, 10H), 0.96 – 0.79 (m, 1H). ^{13}C NMR (126 MHz, $CDCl_3$) δ 141.3, 141.3, 140.4, 140.4, 136.0, 135.9, 135.8, 130.3, 130.3, 130.0, 129.9, 128.1, 128.1, 127.5, 127.5, 127.3, 127.0, 126.9, 126.0, 125.9, 107.6, 107.5, 65.2, 65.2, 65.2, 44.2, 43.2, 42.9, 42.0, 35.1, 34.0, 33.6, 32.7, 27.2, 26.6. DART-MS m/z calcd for $C_{22}H_{25}O_2$ $(M + H)^+$ = 321.18491, found 321.18597.

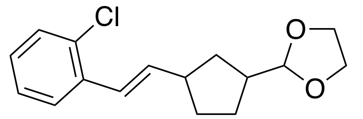

61

(*E*)-2-(3-(2-chlorostyryl)cyclopentyl)-1,3-dioxolane (61): In a 10 mL Schlenk tube, cycloadduct **43** (50.4 mg, 0.15 mmol), scandium(III) triflate (15.1 mg, 0.031 mmol) were dissolved in acetonitrile (0.77 mL, 0.2 M). Trifluoroacetic acid (23 µL, 0.31 mmol), H_2O (55 µL, 3.1 mmol), and ethylene glycol (171 µL, 3.1 mmol) were added to the reaction mixture using a micropipette. After being purged with nitrogen for around 1 min, the tube was sealed with a Teflon stopper and the reaction was stirred at 140 ºC for 16 hours. The mixture was cooled down to room temperature. Additional trifluoroacetic acid (23µL, 0.31 mmol) and ethylene glycol (171 µL, 3.1 mmol) were added to the mixture which was stirred for 1 hour at 140 ºC to convert all of the opened product to the acetal form. The reaction mixture was directly purified using column chromatography with 0–15% hexanes/ethyl acetate gradient. The product containing fractions were concentrated *in vacuo*, resulting in (25.7 mg, 60 % yield). ^{1}H NMR (500 MHz, Chloroform-*d*) δ 7.50 (d, *J* = 7.8 Hz, 1H), 7.32 (dd, *J* = 7.9, 1.4 Hz, 1H), 7.19 (td, *J* = 7.6, 1.4 Hz, 1H), 7.12 (td, *J* = 7.6, 1.7 Hz, 1H), 6.76 (dd, *J* = 15.8, 3.8 Hz, 1H), 6.18 (td, *J* = 16.2, 7.7 Hz, 1H), 4.77 (d, *J* = 5.3 Hz, 1H), 4.04 – 3.93 (m, 2H), 3.88 (q, *J* = 3.1, 2.1 Hz, 2H), 2.83 – 2.66 (m, 1H), 2.41 – 2.22 (m, 1H), 2.10 – 2.00 (m, 1H), 1.99 – 1.76 (m, 2H), 1.75 – 1.29 (m, 3H). ^{13}C NMR (126 MHz, $CDCl_3$) δ 137.8, 137.7, 135.9, 135.9, 132.7, 132.7, 129.7, 128.0, 126.8, 126.7, 124.7, 124.7, 107.5, 107.4, 65.2, 65.2, 65.2, 44.3, 43.3, 42.9, 42.0, 35.0, 34.0, 33.7, 32.7, 27.3, 26.6. DART-MS m/z calcd for $C_{16}H_{20}ClO_2$ $(M + H)^+$ = 279.11463, found 279.11563.

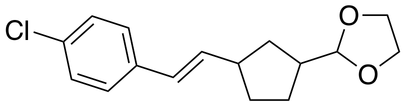

60

(*E*)-2-(3-(4-chlorostyryl)cyclopentyl)-1,3-dioxolane (60): In a 10 mL Schlenk tube, cycloadduct **42** (30.4 mg, 0.092 mmol), scandium(III) triflate (9.1 mg, 0.018 mmol) were dissolved in acetonitrile (0.46 mL, 0.2 M). Trifluoroacetic acid (14 µL, 0.19 mmol), H_2O (33 µL, 1.9 mmol), and ethylene glycol (103 µL, 1.9 mmol) were added to the reaction mixture using a micropipette. After being purged with nitrogen for around 1 min, the tube was sealed with a Teflon stopper and the reaction was stirred at 140 ºC for 16 hours. The mixture was cooled down to room temperature. Additional trifluoroacetic acid (14 µL, 0.19 mmol) and ethylene glycol (103 µL, 1.9 mmol) were added to the mixture which was stirred for 1 hour at 140 ºC to convert all of the opened product to the acetal form. The reaction mixture was directly purified using column chromatography with 0–15% hexanes/ethyl acetate gradient. The product containing fractions were concentrated *in vacuo*, resulting in (23.3 mg, 90 % yield). ^{1}H NMR (500 MHz, Chloroform-*d*) δ 7.26 (d, *J* = 1.4 Hz, 4H), 6.34 (ddd, *J* = 15.8, 5.2, 1.0 Hz, 1H), 6.18 (td, *J* = 16.1, 7.6 Hz, 1H), 4.78 (d, *J* = 5.3 Hz, 1H), 4.05 – 3.94 (m, 2H), 3.94 (s, 0H), 2.67 (dddd, *J* = 27.7, 14.3, 9.5, 7.1 Hz, 1H), 2.40 – 2.22 (m, 1H), 2.08 – 1.97 (m, 0H), 1.97 – 1.75 (m, 2H), 1.75 – 1.33 (m, 3H), 0.95 – 0.80 (m, 1H). ^{13}C NMR (126 MHz, $CDCl_3$) δ 136.3, 135.6, 135.4, 132.3, 128.6, 127.2, 127.1, 107.3, 107.3, 65.1, 65.1, 65.1,

44.0, 43.0, 42.8, 41.9, 34.8, 33.9, 33.5, 32.6, 27.1, 26.5. DART-MS m/z calcd for $C_{16}H_{20}ClO_2$ (M + H)$^+$ = 279.11463, found 279.11557.

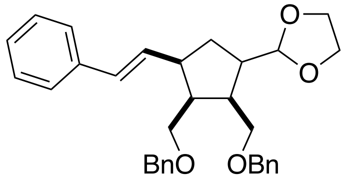

68

(*E*)-2-(2,3-bis((benzyloxy)methyl)-4-styrylcyclopentyl)-1,3-dioxolane (68): In a 10 mL Schlenk tube, cycloadduct **67** (44.5 mg, 0.083 mmol), scandium(III) triflate (8.2 mg, 0.017 mmol) were dissolved in acetonitrile (0.46 mL, 0.2 M). Trifluoroacetic acid (13 µL, 0.17 mmol), H_2O (30 µL, 1.7 mmol), and ethylene glycol (93 µL, 1.7 mmol) were added to the reaction mixture using a micropipette. After being purged with nitrogen for around 1 min, the tube was sealed with a Teflon stopper and the reaction was stirred at 140 ºC for 16 hours. The mixture was cooled down to room temperature. Additional trifluoroacetic acid (13 µL, 0.17 mmol) and ethylene glycol (93 µL, 1.7 mmol) were added to the mixture which was stirred for 1 hour at 140 ºC to convert all of the opened product to the acetal form. The reaction mixture was directly purified using column chromatography with 0–15% hexanes/ethyl acetate gradient. The product containing fractions were concentrated *in vacuo*, resulting in (18.5 mg, 46 % yield). ^{1}H NMR (500 MHz, Chloroform-*d*) δ 7.39 – 7.15 (m, 17H), 6.38 (d, *J* = 16.0 Hz, 1H), 6.32 – 6.16 (m, 1H), 4.88 (d, *J* = 4.3 Hz, 1H), 4.51 – 4.38 (m, 2H), 4.38 – 4.30 (m, 2H), 4.02 – 3.92 (m, 2H), 3.90 – 3.81 (m, 2H), 3.66 (dd, *J* = 9.2, 5.1 Hz, 1H), 3.61 – 3.46 (m, 3H), 2.90 (p, *J* = 8.0 Hz, 1H), 2.54 – 2.38 (m, 2H), 2.29 (tt, *J* = 9.7, 5.2 Hz, 1H), 2.14 – 1.74 (m, 2H). ^{13}C NMR (126 MHz, $CDCl_3$) δ 138.8, 138.8, 138.0, 132.3, 129.9, 128.7, 128.6, 128.5, 128.5, 128.4, 128.0, 127.9, 127.8, 127.6, 127.5, 126.9, 126.2, 126.2, 107.2, 73.3, 73.2, 71.3, 68.7, 65.3, 65.1, 46.6, 46.0, 44.1, 43.2, 32.5. DART-MS m/z calcd for $C_{32}H_{37}O_4$ (M + H)$^+$ = 485.26864, found 485.26961.

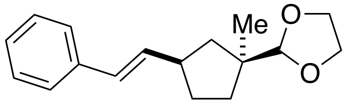

71

2-((1*S*,3*R*)-1-methyl-3-((*E*)-styryl)cyclopentyl)-1,3-dioxolane (71): In a 10 mL Schlenk tube, hydrazine TFA salt **6** (40.0 mg, 0.12 mmol, 20 mol%), scandium(III) triflate (11.6 mg, 0.024 mmol) were dissolved in acetonitrile (0.65 mL, 0.18 M). Benzaldehyde (62.4 mg, 59 µL, 0.59 mmol), substituted norbornene **70** (63.6 mg, 0.59 mmol), and ethylene glycol (131 µL, 2.4 mmol) were added to the reaction mixture using a micropipette. After being purged with nitrogen for around 1 min, the tube was sealed with a Teflon stopper and the reaction was stirred at 140 ºC for 48 hours. The mixture was cooled down to room temperature. Additional trifluoroacetic acid (21 µL, 0.27 mmol) and ethylene glycol (152 µL, 2.7 mmol) were added to the mixture which was stirred for 1 hour at 140 ºC to convert all of the opened product to the acetal form. The reaction mixture was directly purified using column chromatography with 0–15% hexanes/ethyl acetate gradient. The reaction mixture was analyzed using quantitative proton NMR using mesitylene as

an internal standard and determined to contain 70% of ring-opened product. ^{1}H NMR (500 MHz, Chloroform-d) δ 7.36 (d, *J* = 7.1 Hz, 2H), 7.31 (d, *J* = 7.5 Hz, 2H), 7.23 – 7.17 (m, 1H), 6.41 (d, *J* = 15.8 Hz, 1H), 6.22 (dd, *J* = 15.8, 7.6 Hz, 1H), 4.70 (s, 1H), 4.05 – 3.95 (m, 2H), 3.95 – 3.87 (m, 2H), 2.81 (ddt, *J* = 17.5, 10.1, 7.4 Hz, 1H), 1.93 (tdd, *J* = 12.4, 7.5, 3.5 Hz, 2H), 1.62 (dd, *J* = 9.0, 4.2 Hz, 2H), 1.57 – 1.49 (m, 1H), 1.43 – 1.34 (m, 1H), 1.10 (s, 3H). ^{13}C NMR (126 MHz, $CDCl_3$) ^{13}C NMR (126 MHz, $CDCl_3$) δ 138.0, 135.0, 128.6, 128.3, 126.9, 126.1, 110.1, 65.6, 65.5, 45.7, 42.9, 42.1, 35.1, 33.3, 23.3. DART-MS m/z calcd for $C_{17}H_{23}O_2$ $(M + H)^+$ = 259.16926, found 259.17009.

NMR Spectra

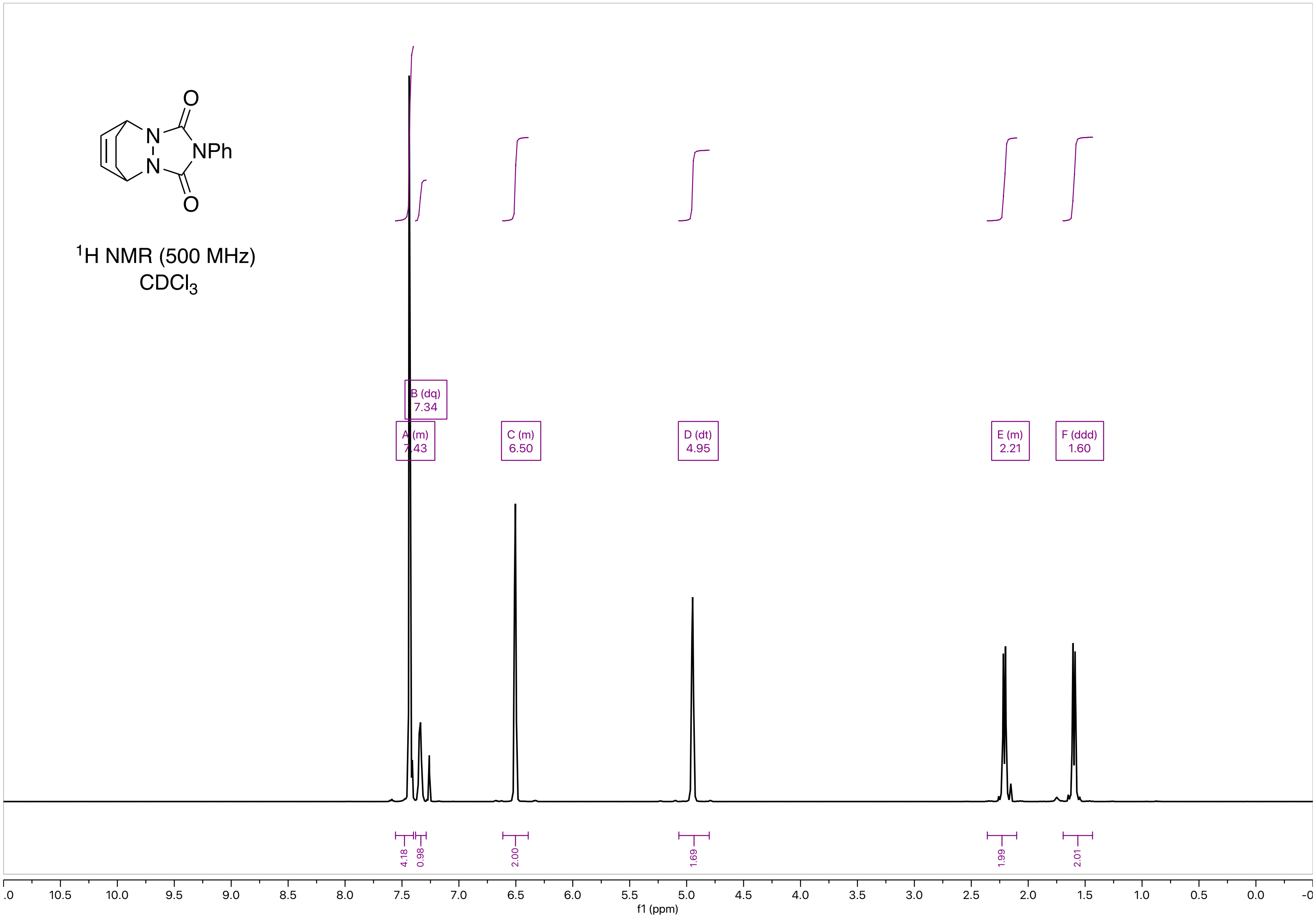
O
N
N
NPh
O
1H NMR (500 MHz)
CDCl3
B (dq)
7.34
A (m)
7.43
C (m)
6.50
D (dt)
4.95
E (m)
2.21
F (ddd)
1.60
4.18
0.98
2.00
1.69
1.99
2.01
f1 (ppm)

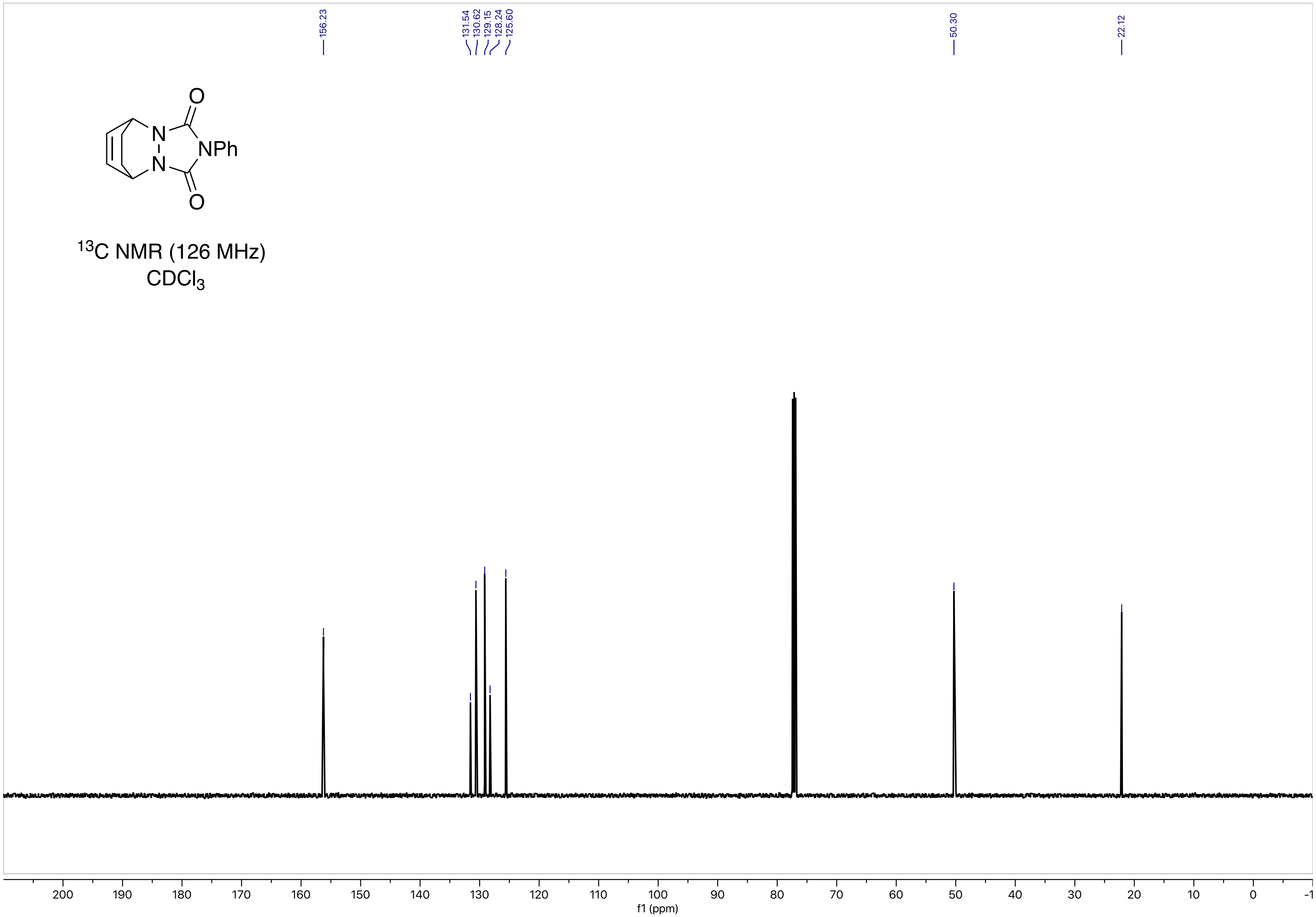

156.23
131.54
130.62
129.15
128.24
125.60
50.30
22.12
O
N
N
NPh
O
13C NMR (126 MHz)
CDCl3
200
190
180
170
160
150
140
130
120
110
100
90
80
70
60
50
40
30
20
10
0
-1
f1 (ppm)

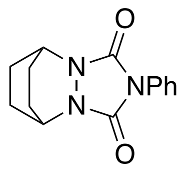

^{1}H NMR (500 MHz)
$CDCl_3$

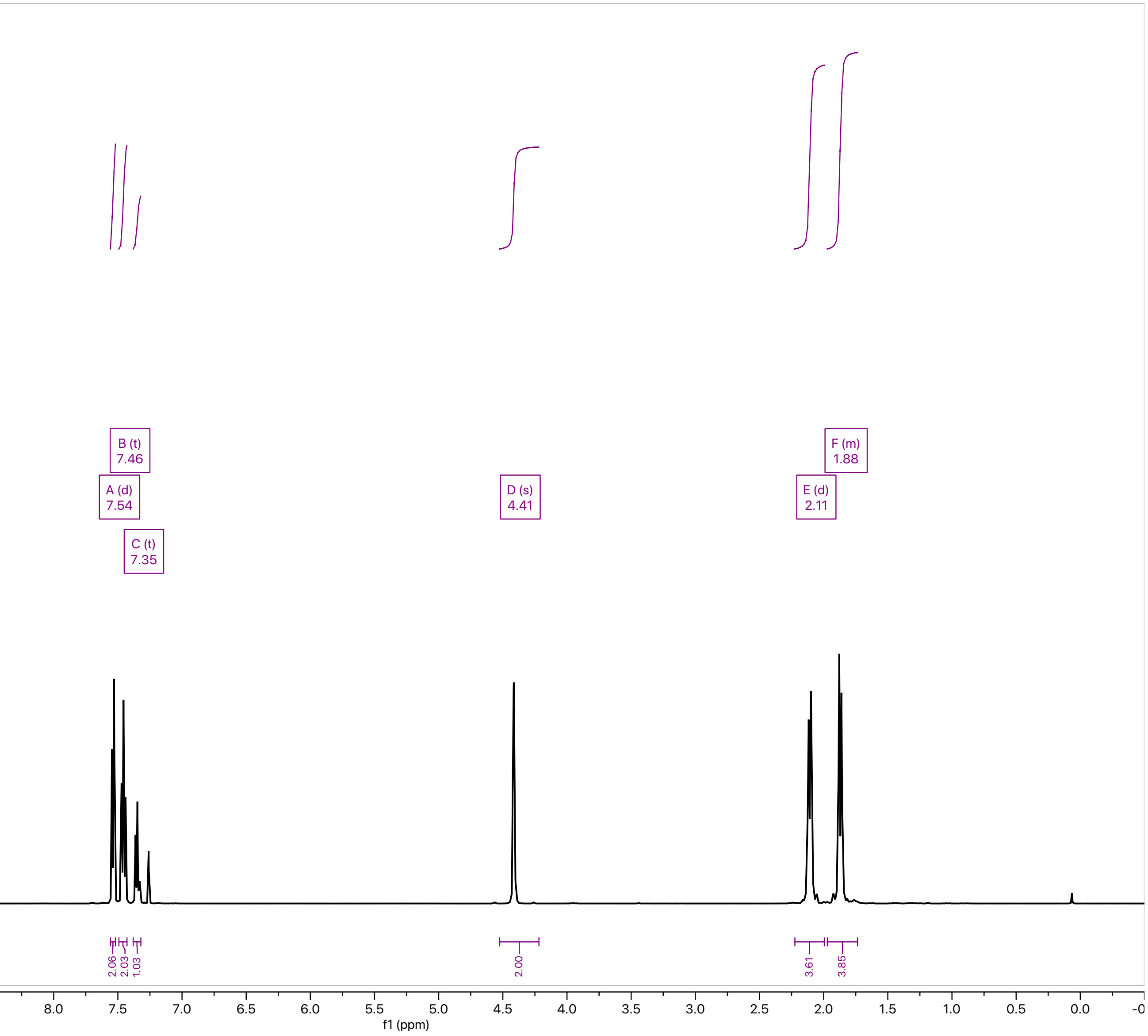

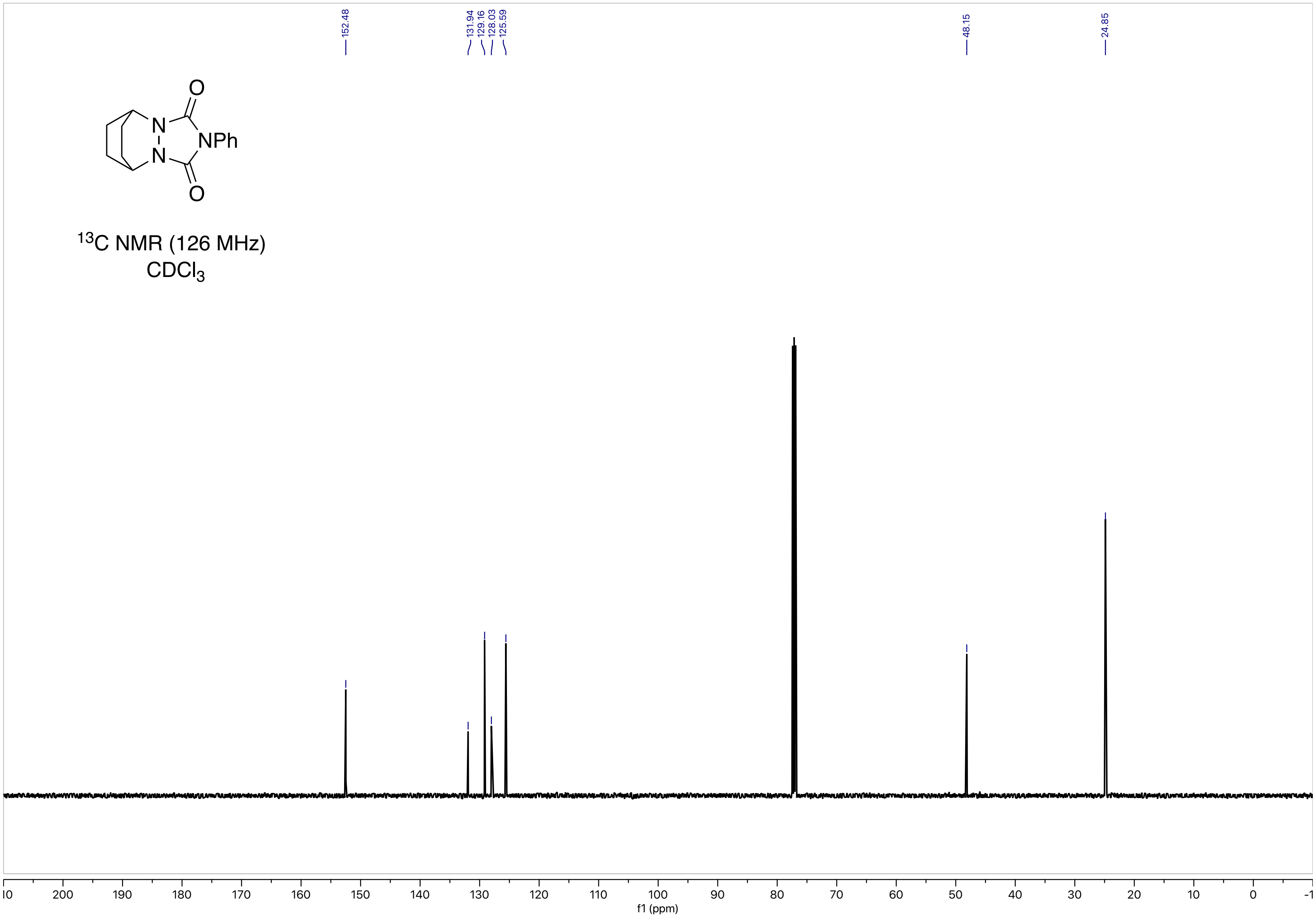
152.48
131.94
129.16
128.03
125.59
48.15
24.85
O
N
N
NPh
O
^{13}C NMR (126 MHz)
$CDCl_3$
f1 (ppm)

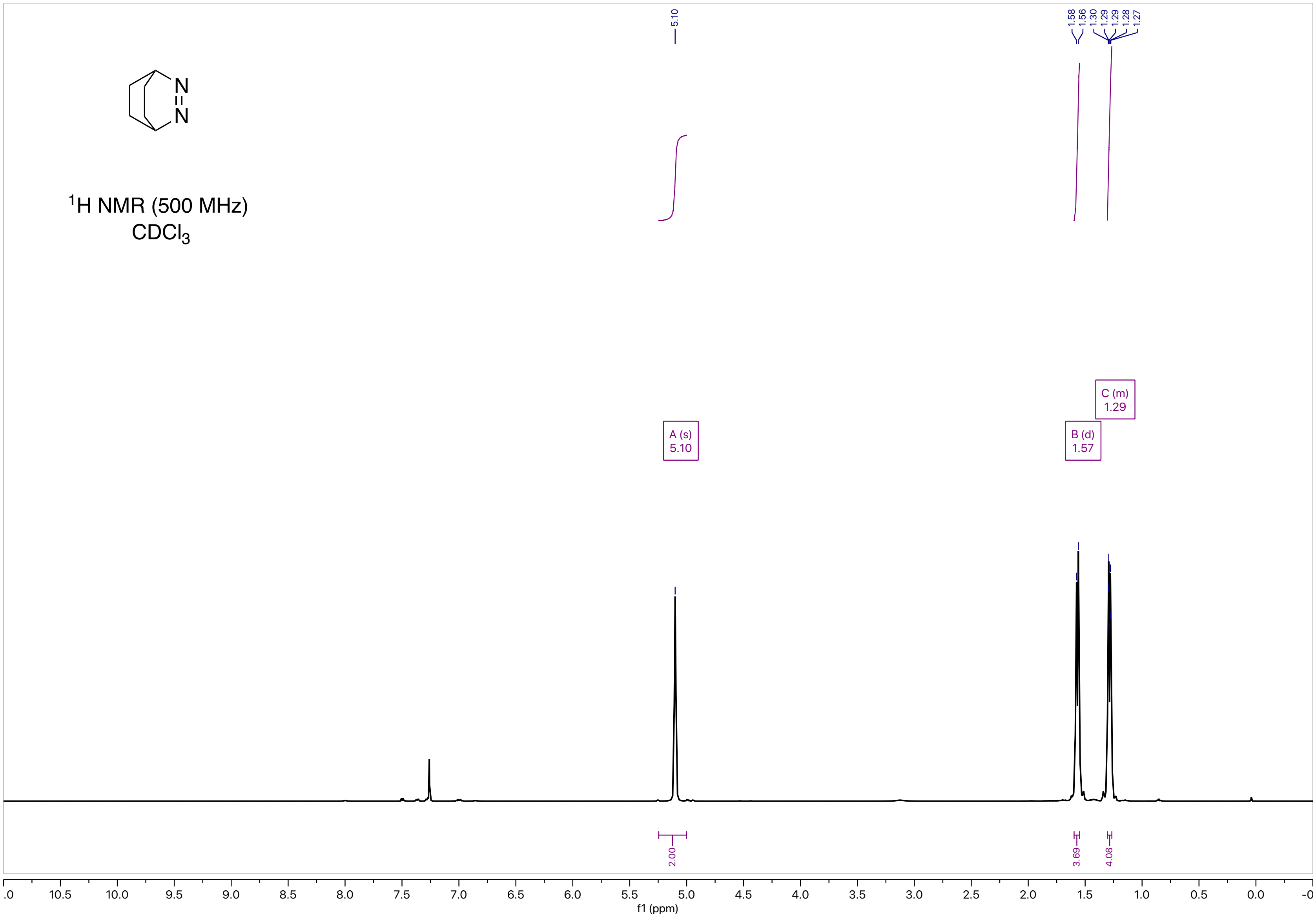

^{1}H NMR (500 MHz)
$CDCl_3$
5.10
1.58
1.56
1.30
1.29
1.29
1.28
1.27
A (s)
5.10
B (d)
1.57
C (m)
1.29
2.00
3.69
4.08
f1 (ppm)

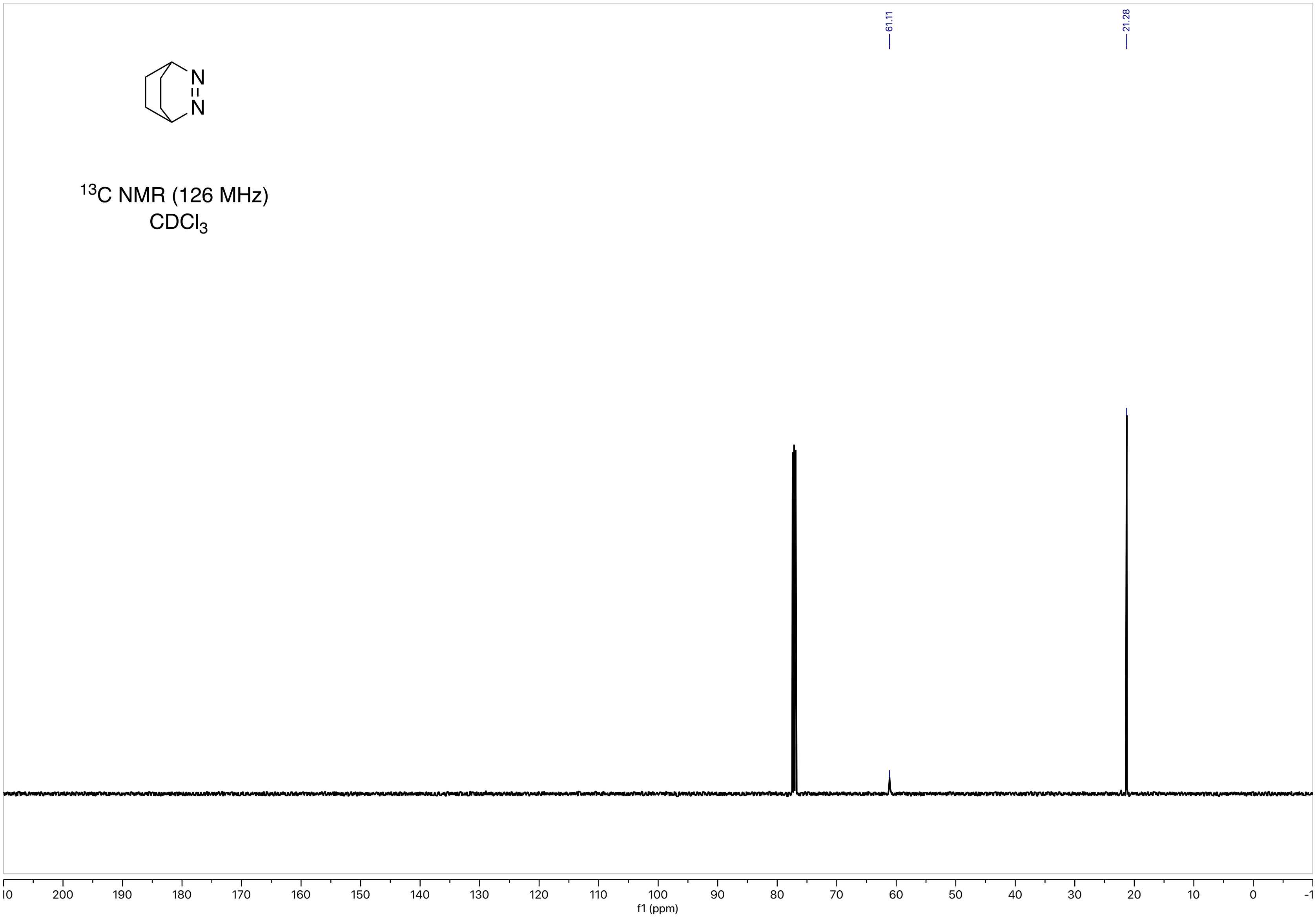
61.11
21.28
N
N
^{13}C NMR (126 MHz)
$CDCl_3$
200
190
180
170
160
150
140
130
120
110
100
90
80
70
60
50
40
30
20
10
0
f1 (ppm)

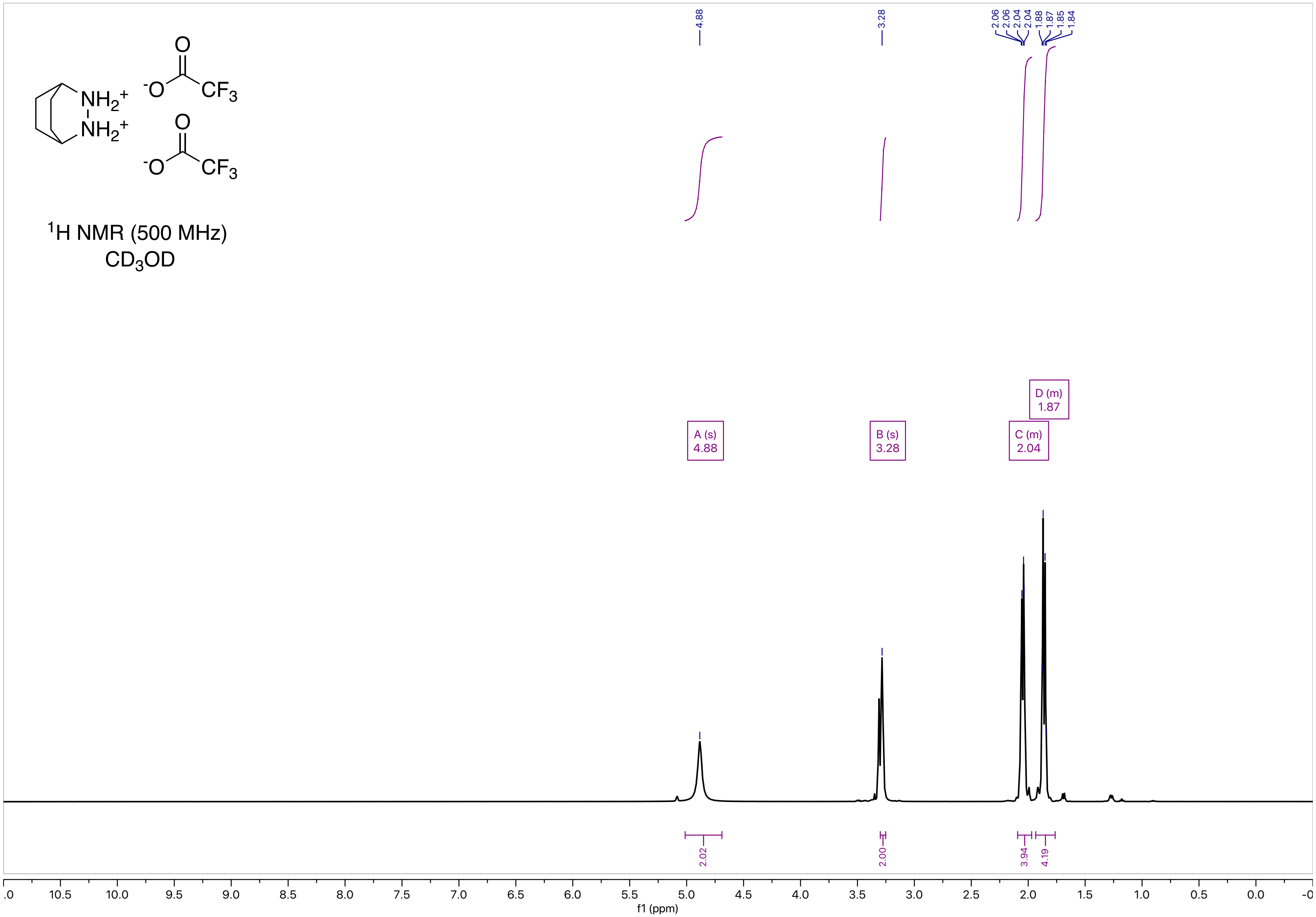
NH2+
NH2+
-O
CF3
O
1H NMR (500 MHz)
CD3OD
4.88
3.28
2.06
2.06
2.04
2.04
1.88
1.87
1.85
1.84
A (s)
4.88
B (s)
3.28
C (m)
2.04
D (m)
1.87
2.02
2.00
3.94
4.19
f1 (ppm)

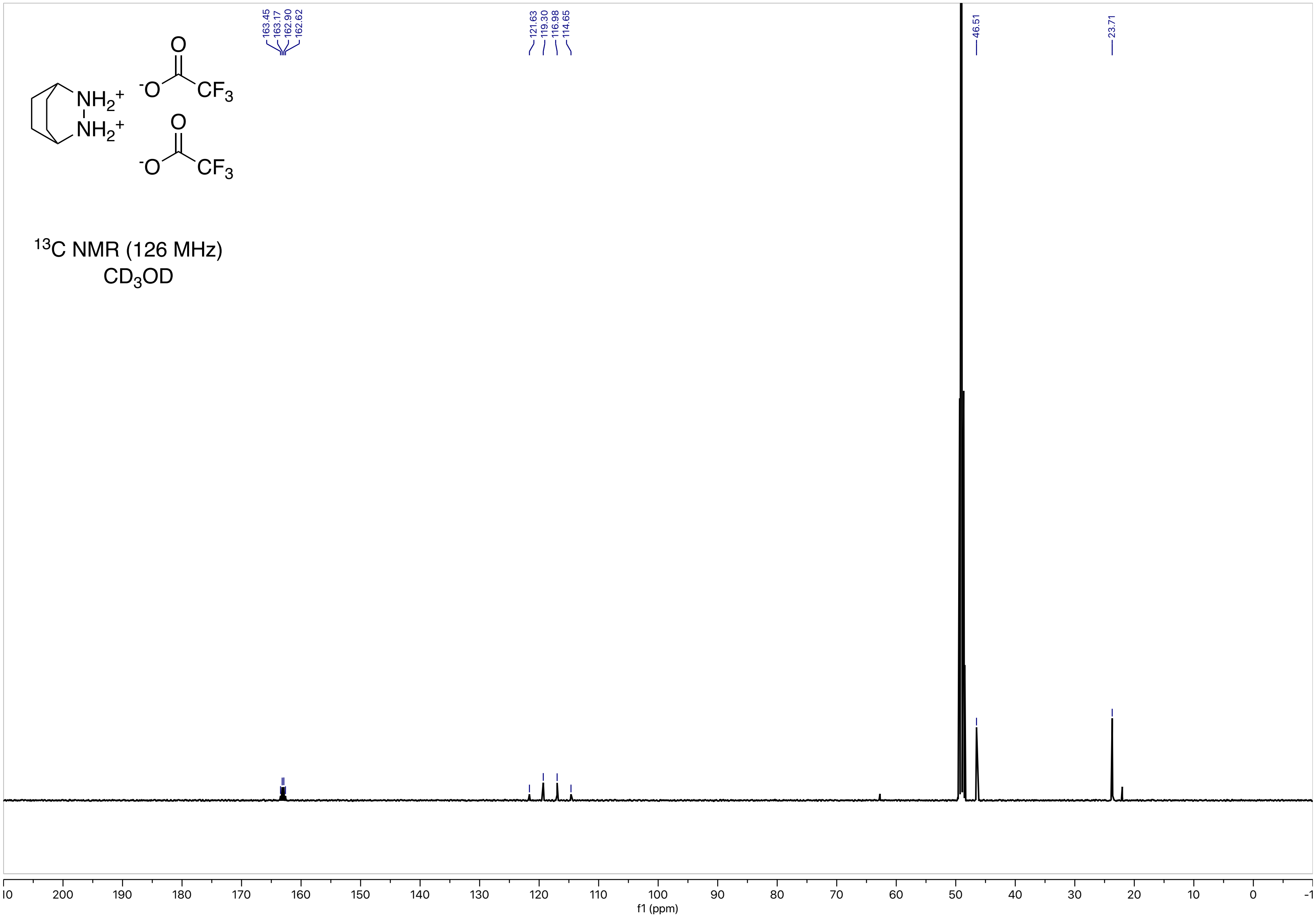

^{13}C NMR (126 MHz)
CD_3OD

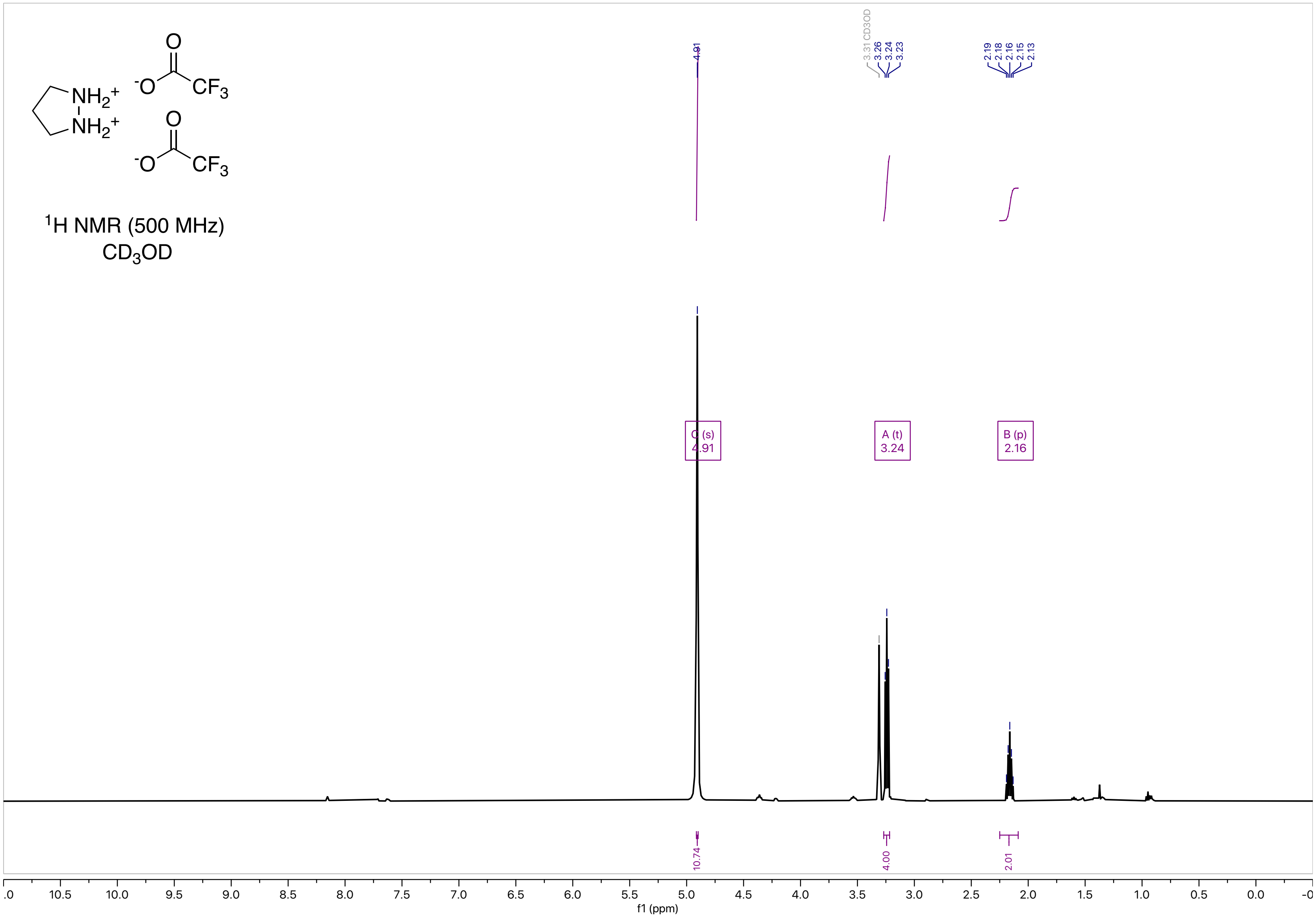

^{1}H NMR (500 MHz)
CD_3OD
3.31 CD3OD
4.91
3.26
3.24
3.23
2.19
2.18
2.16
2.15
2.13
C (s)
4.91
A (t)
3.24
B (p)
2.16
10.74
4.00
2.01
f1 (ppm)

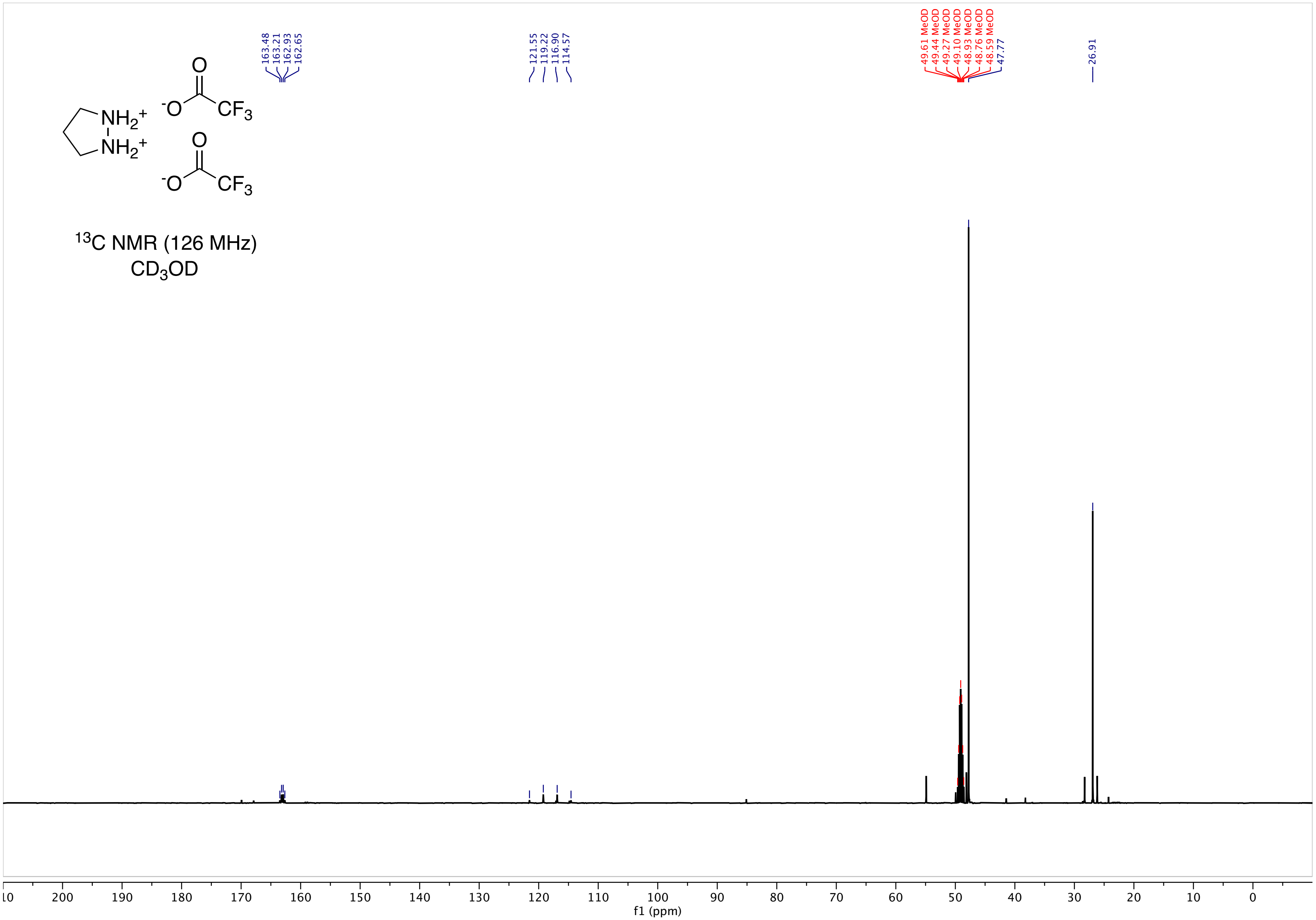

^{13}C NMR (126 MHz)
CD$_3$OD
163.48
163.21
162.93
162.65
121.55
119.22
116.90
114.57
49.61 MeOD
49.44 MeOD
49.27 MeOD
49.10 MeOD
48.93 MeOD
48.76 MeOD
48.59 MeOD
47.77
26.91
f1 (ppm)

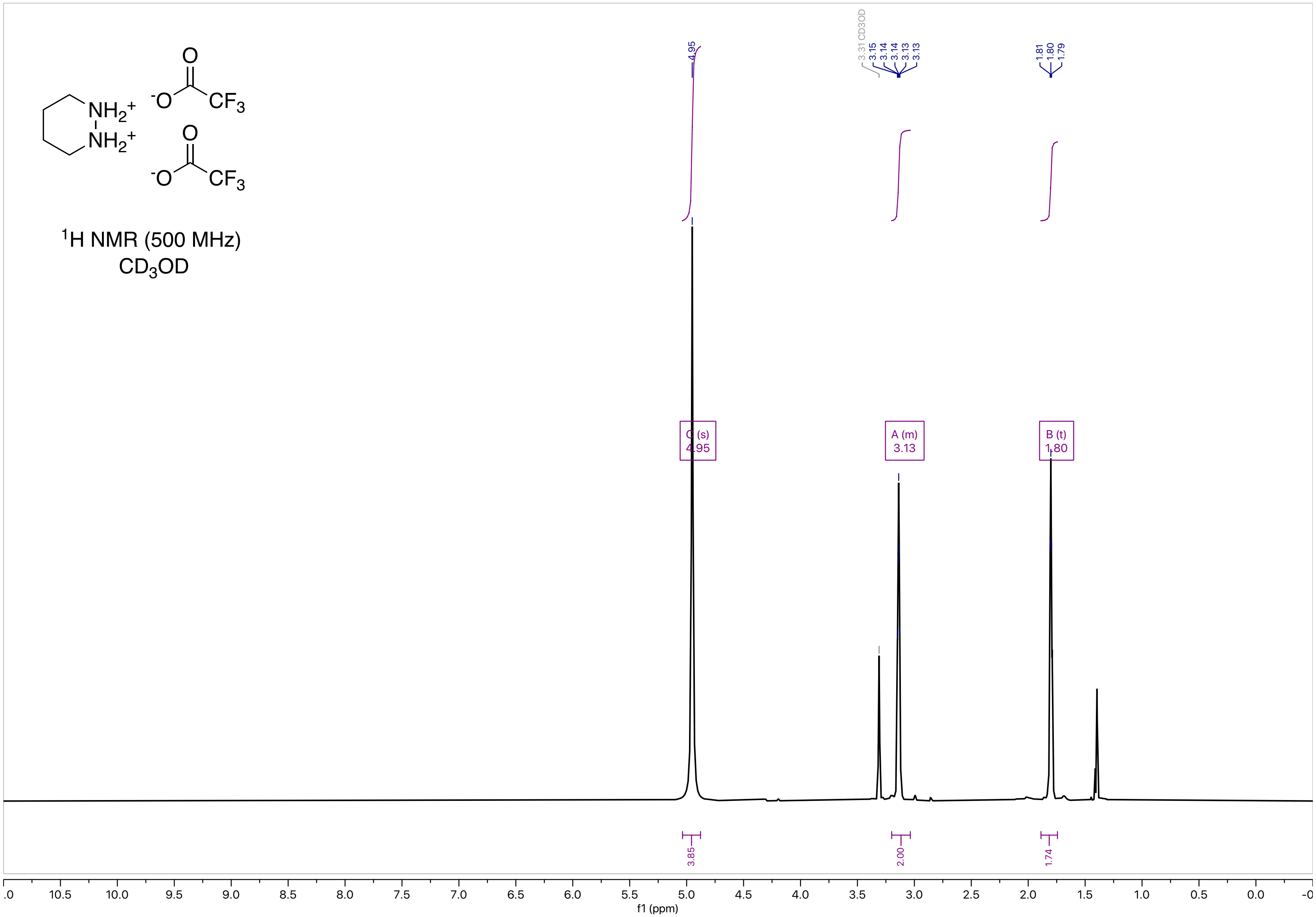

NH2+
NH2+
-O
CF3
O
1H NMR (500 MHz)
CD3OD
4.95
3.31 CD3OD
3.15
3.14
3.14
3.13
3.13
1.81
1.80
1.79
C (s)
4.95
A (m)
3.13
B (t)
1.80
3.85
2.00
1.74
f1 (ppm)

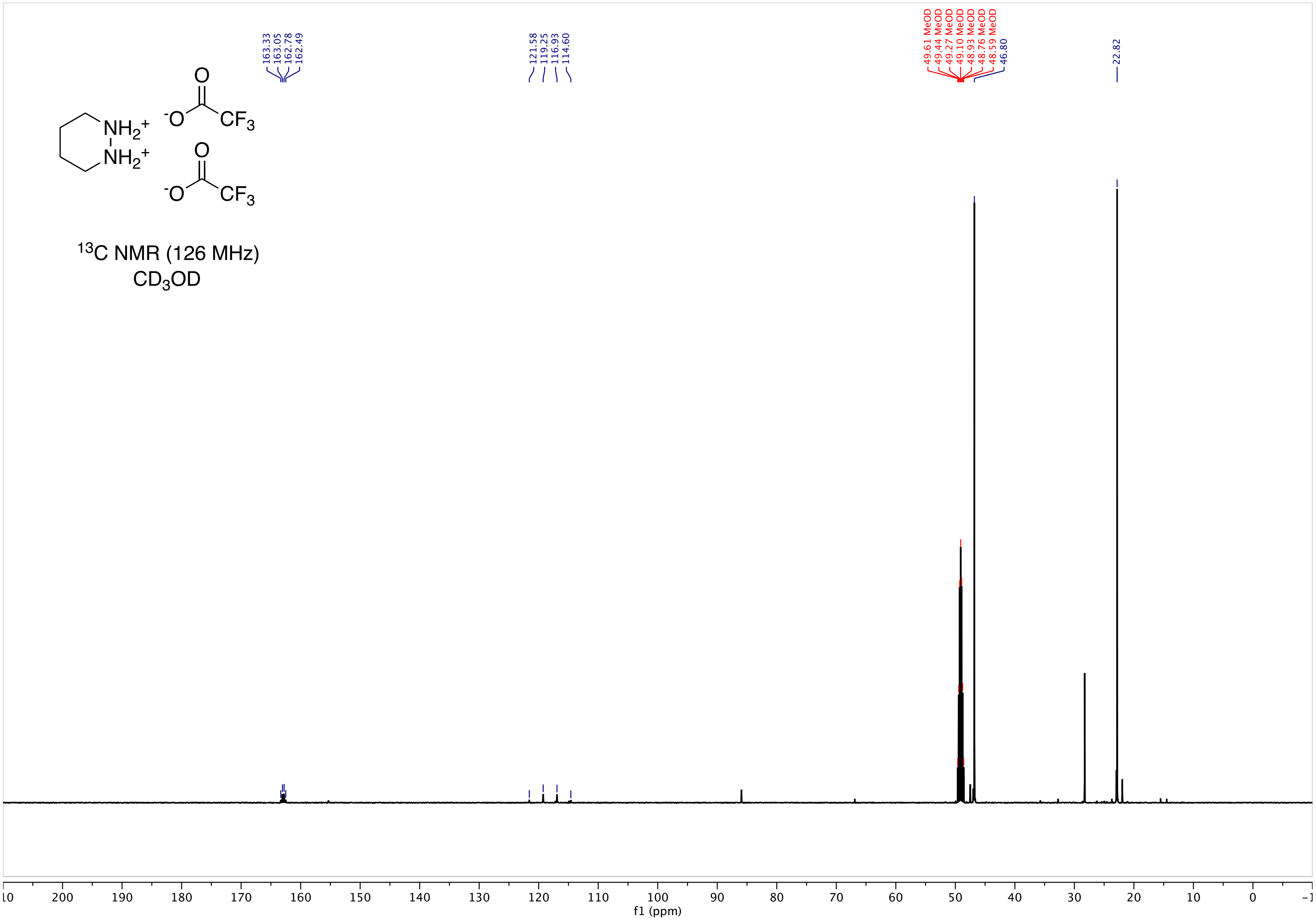

NH2+
NH2+
-O
O
CF3
-O
O
CF3
13C NMR (126 MHz)
CD3OD
163.33
163.05
162.78
162.49
121.58
119.25
116.93
114.60
49.61 MeOD
49.44 MeOD
49.27 MeOD
49.10 MeOD
48.93 MeOD
48.76 MeOD
48.59 MeOD
46.80
22.82
f1 (ppm)

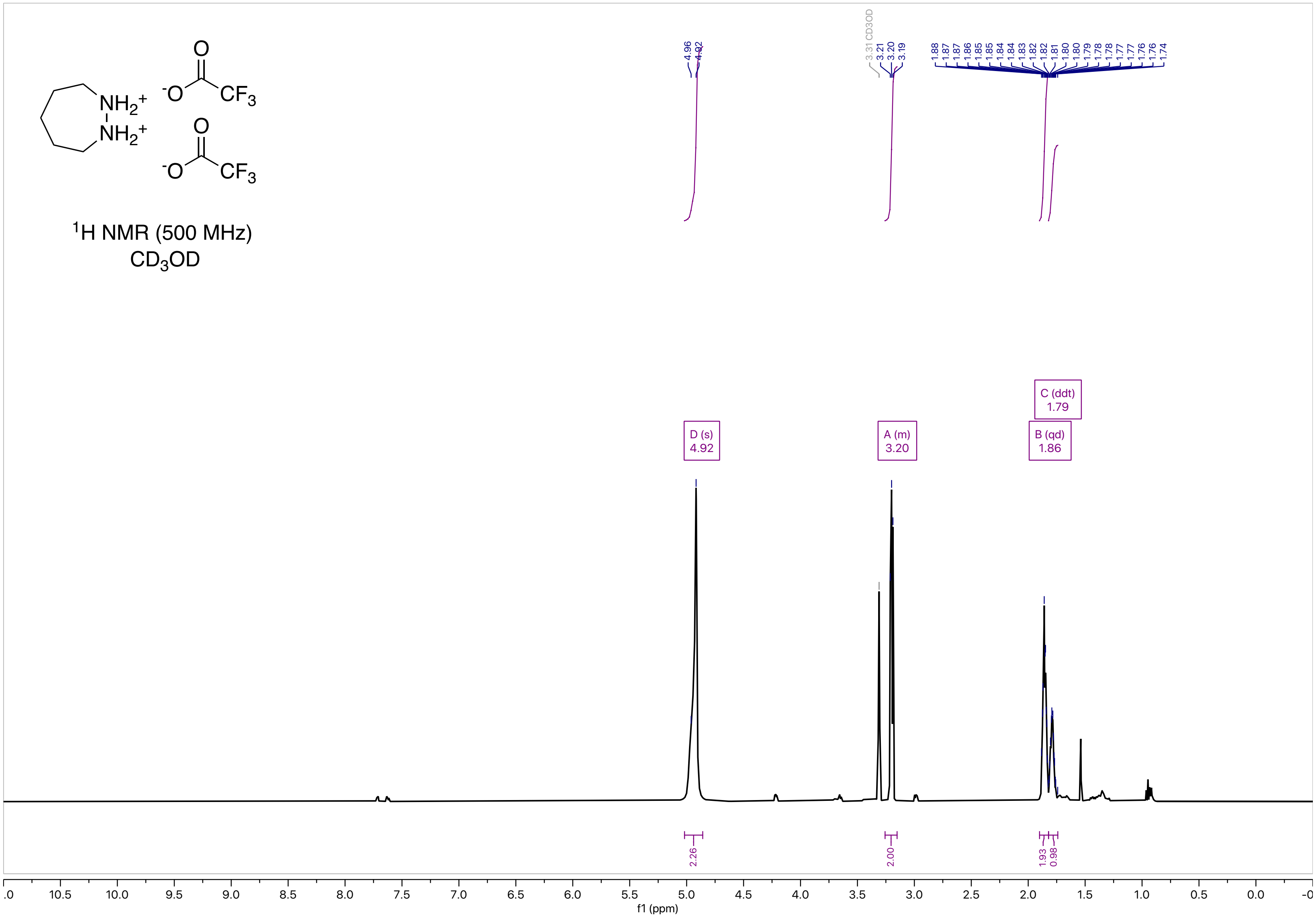
1H NMR (500 MHz)
CD3OD
D (s) 4.92
A (m) 3.20
B (qd) 1.86
C (ddt) 1.79
3.31 CD3OD
2.26
2.00
1.93
0.98
f1 (ppm)

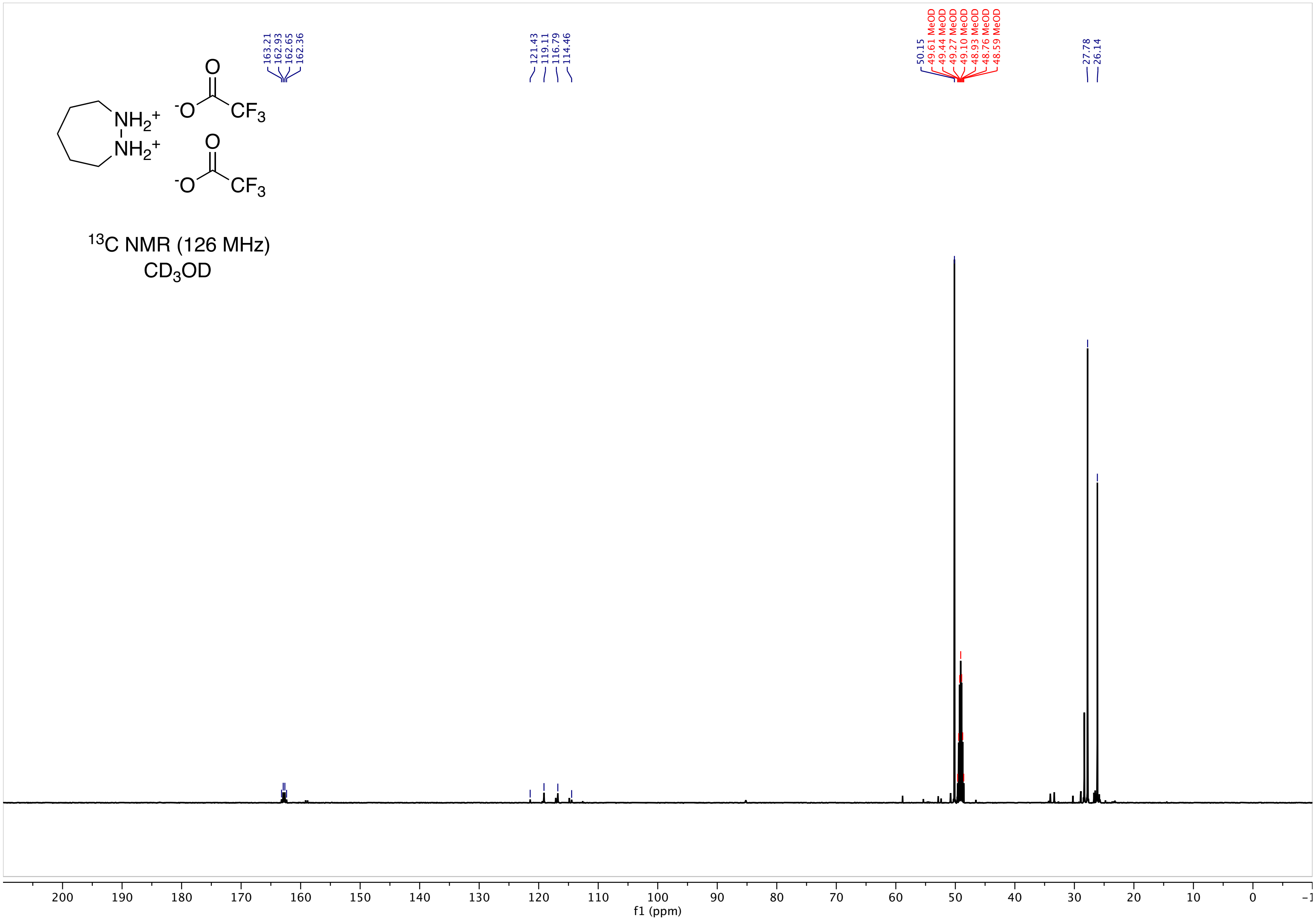

163.21
162.93
162.65
162.36
121.43
119.11
116.79
114.46
50.15
49.61 MeOD
49.44 MeOD
49.27 MeOD
49.10 MeOD
48.93 MeOD
48.76 MeOD
48.59 MeOD
27.78
26.14
NH2+
NH2+
-O
O
CF3
^{13}C NMR (126 MHz)
CD3OD
f1 (ppm)

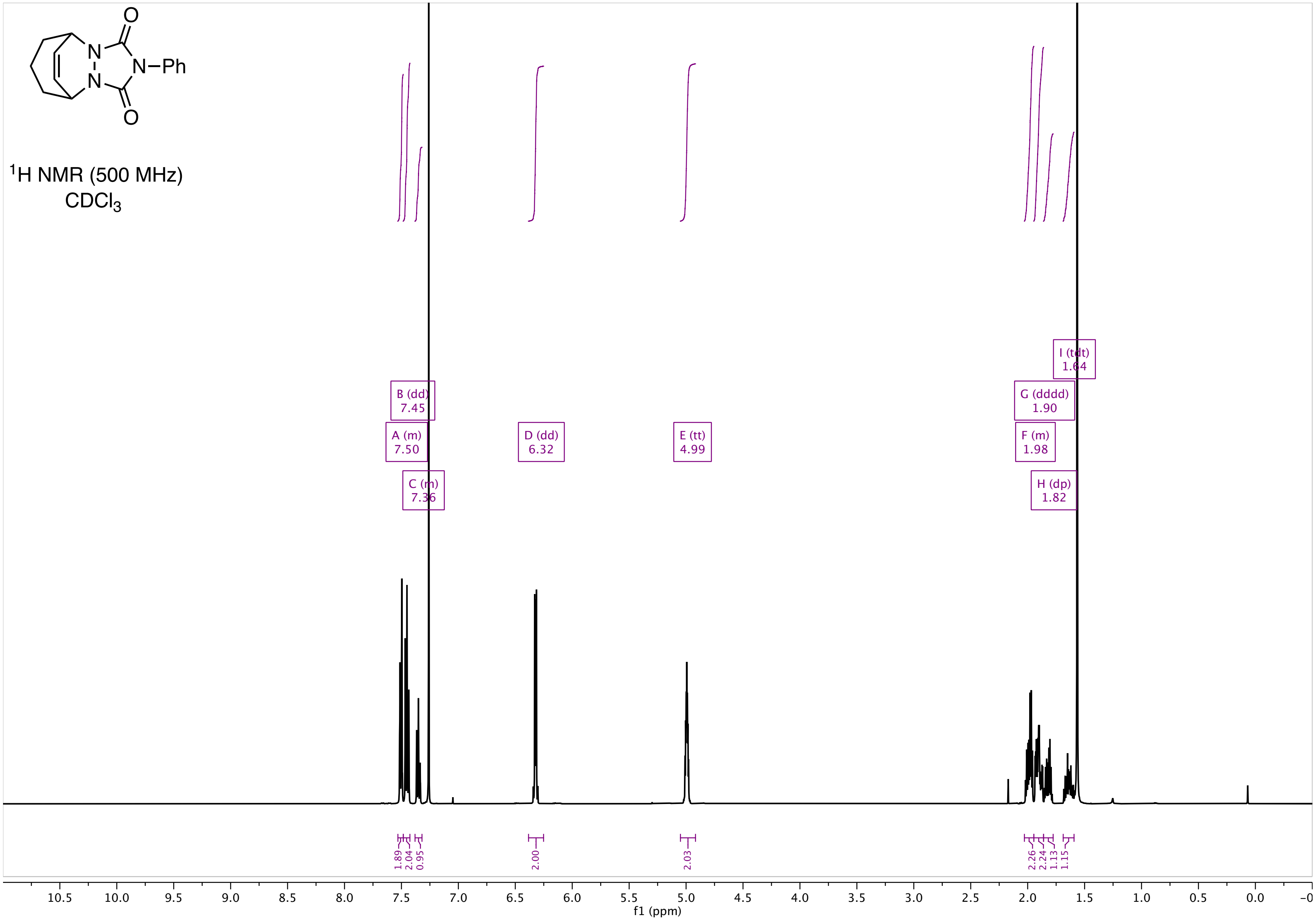

O
N
N
N–Ph
O
1H NMR (500 MHz)
CDCl3
B (dd)
7.45
A (m)
7.50
C (m)
7.36
D (dd)
6.32
E (tt)
4.99
I (tdt)
1.64
G (dddd)
1.90
F (m)
1.98
H (dp)
1.82
1.89
2.04
0.95
2.00
2.03
2.26
2.24
1.13
1.15
10.5 10.0 9.5 9.0 8.5 8.0 7.5 7.0 6.5 6.0 5.5 5.0 4.5 4.0 3.5 3.0 2.5 2.0 1.5 1.0 0.5 0.0
f1 (ppm)

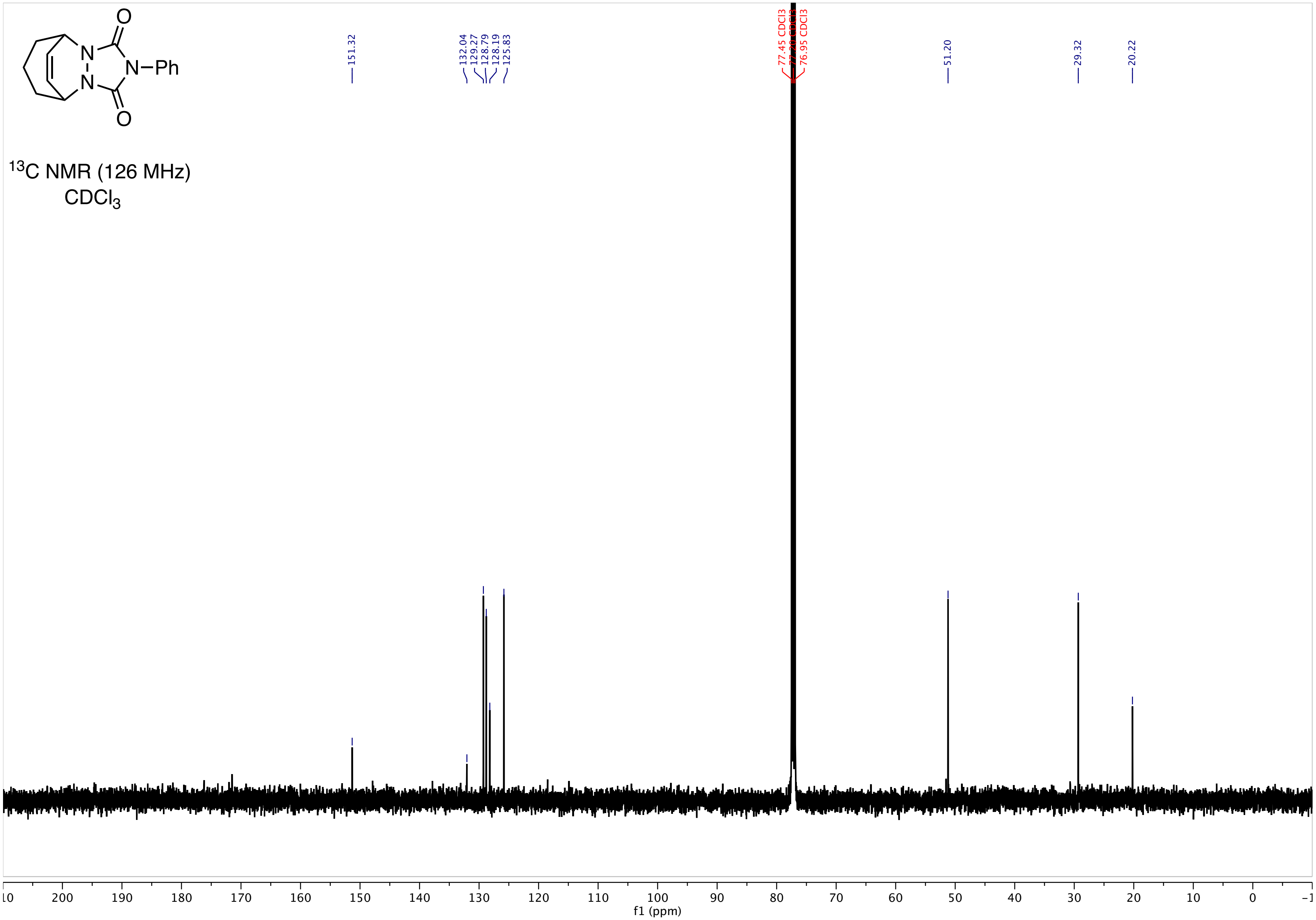
N
N
N–Ph
O
O
13C NMR (126 MHz)
CDCl3
151.32
132.04
129.27
128.79
128.19
125.83
77.45 CDCl3
76.95 CDCl3
51.20
29.32
20.22
f1 (ppm)

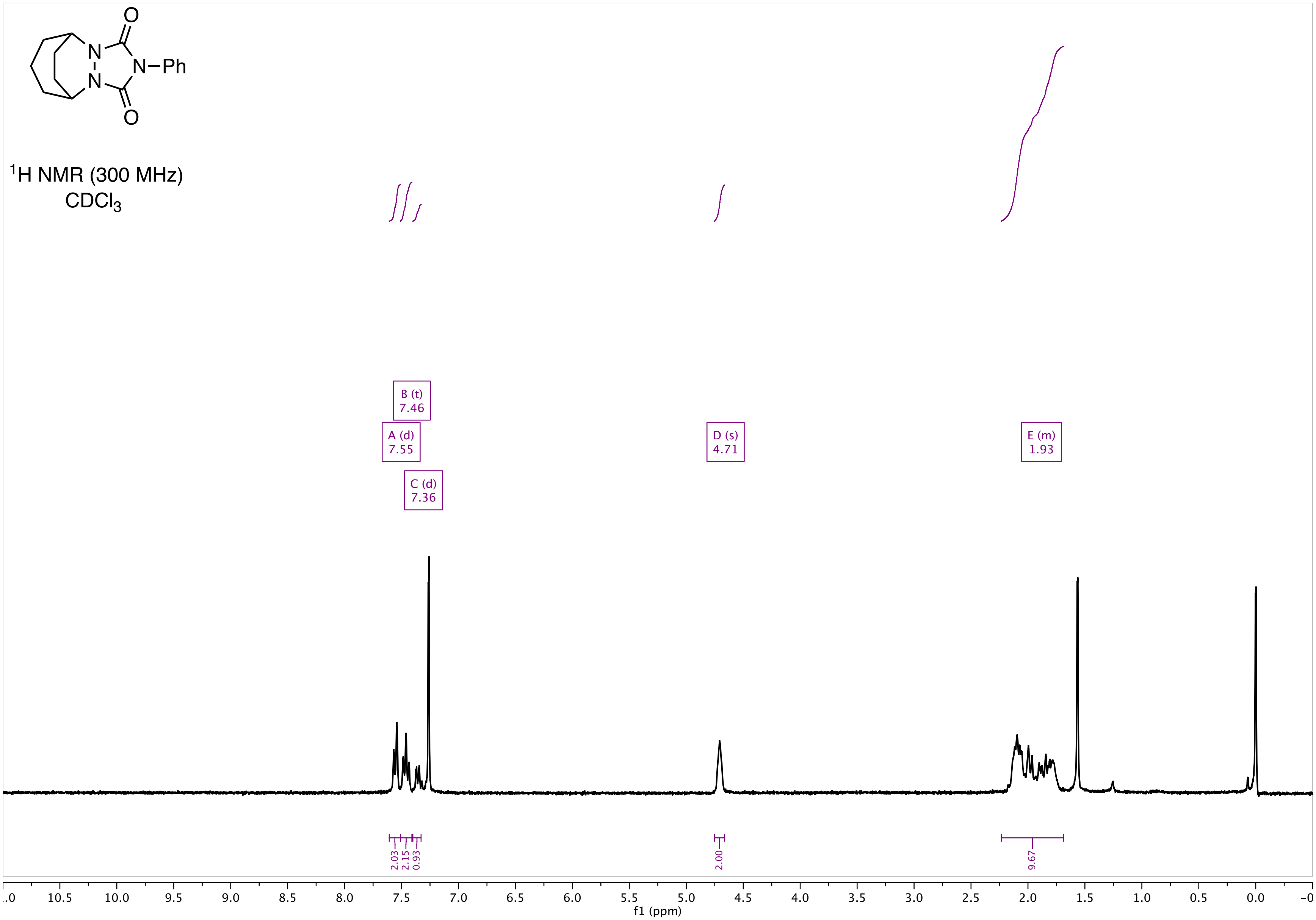
O
N
N
N−Ph
O
1H NMR (300 MHz)
CDCl3
B (t)
7.46
A (d)
7.55
C (d)
7.36
D (s)
4.71
E (m)
1.93
2.03
2.15
0.93
2.00
9.67
11.0
10.5
10.0
9.5
9.0
8.5
8.0
7.5
7.0
6.5
6.0
5.5
5.0
4.5
4.0
3.5
3.0
2.5
2.0
1.5
1.0
0.5
0.0
f1 (ppm)

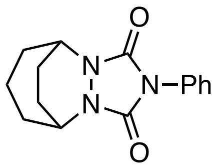

^{13}C NMR (126 MHz)
$CDCl_3$

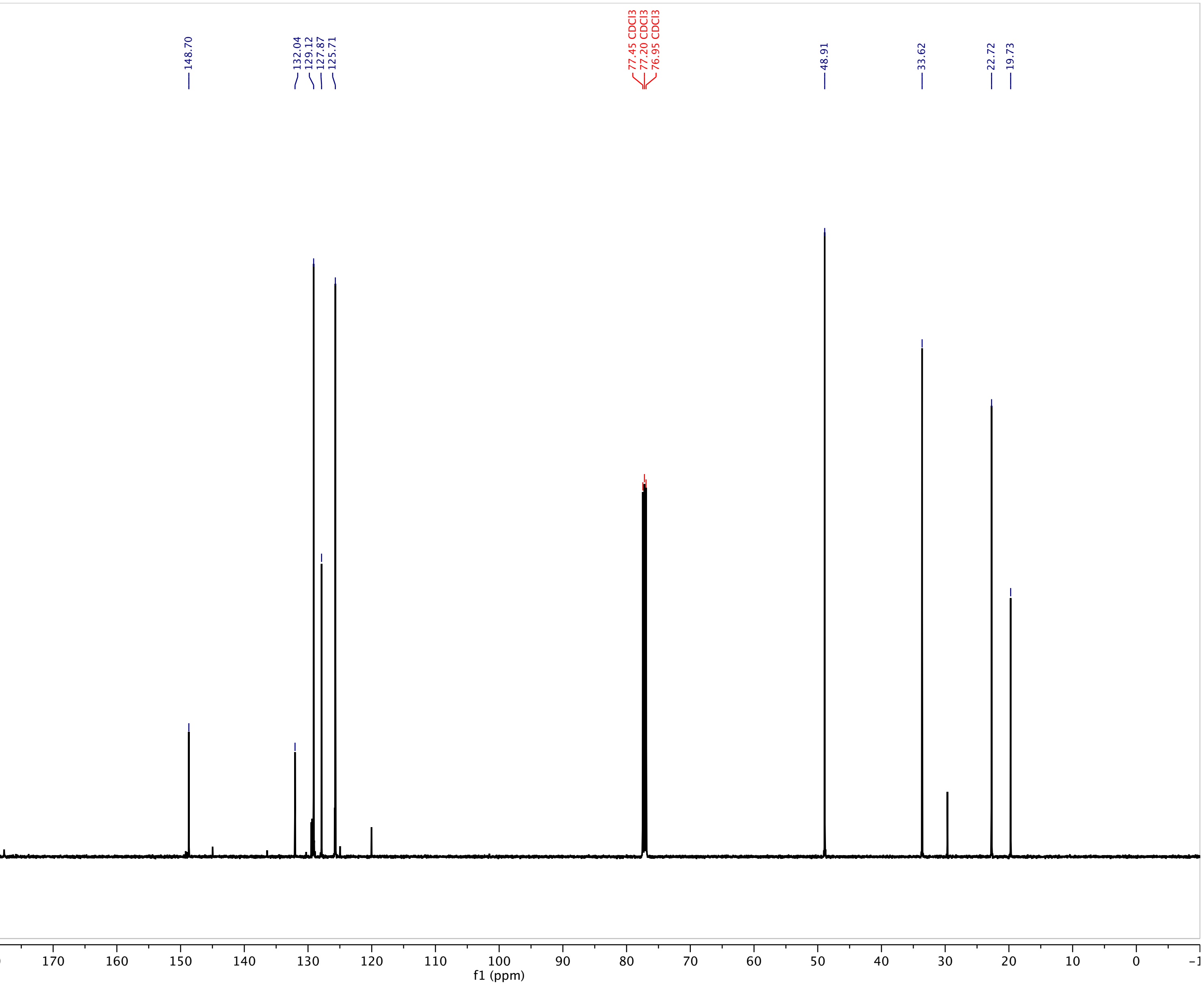

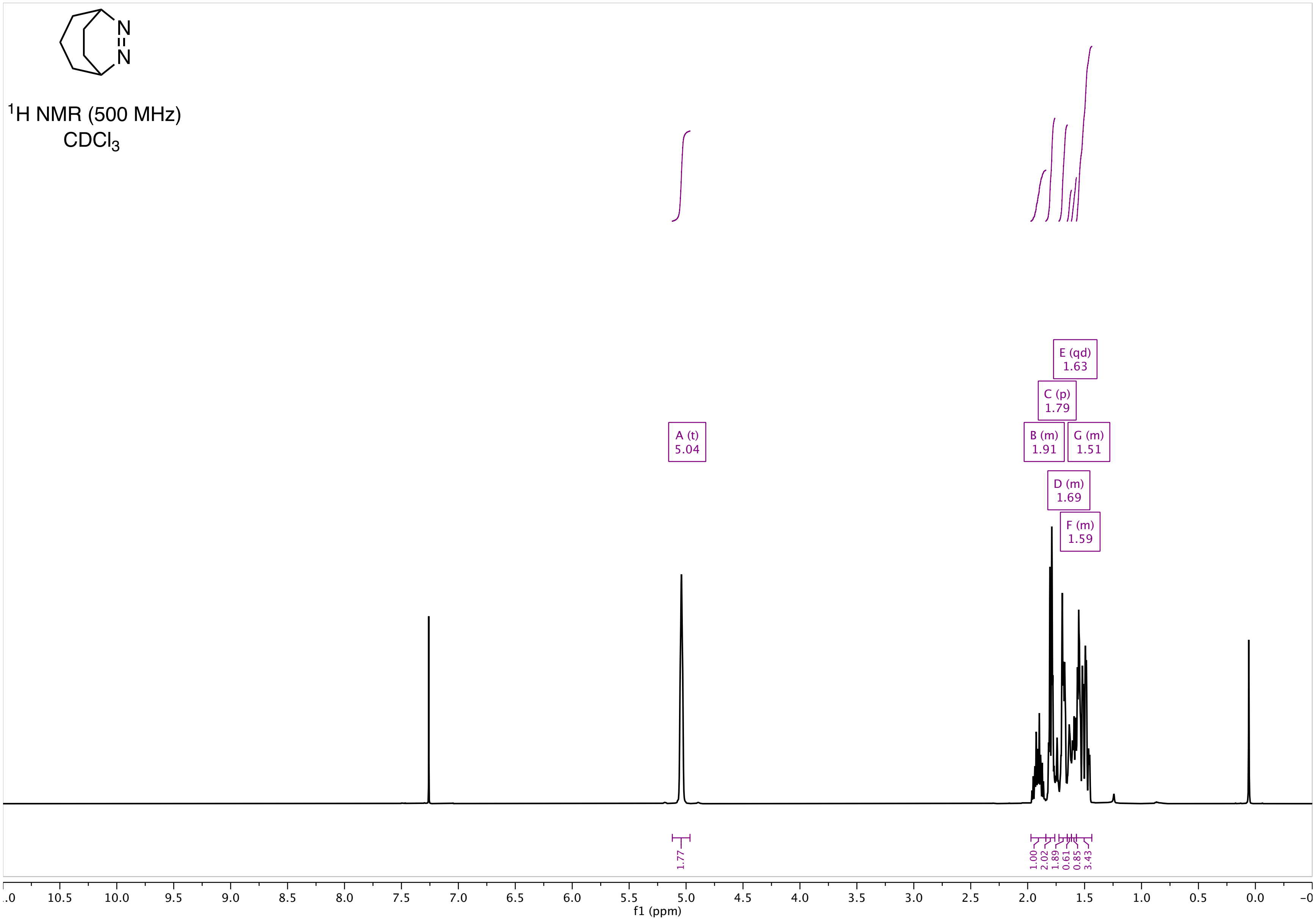

N
N
^{1}H NMR (500 MHz)
$CDCl_3$
A (t)
5.04
E (qd)
1.63
C (p)
1.79
B (m)
1.91
G (m)
1.51
D (m)
1.69
F (m)
1.59
1.77
1.00
2.02
1.89
0.61
0.85
3.43
f1 (ppm)

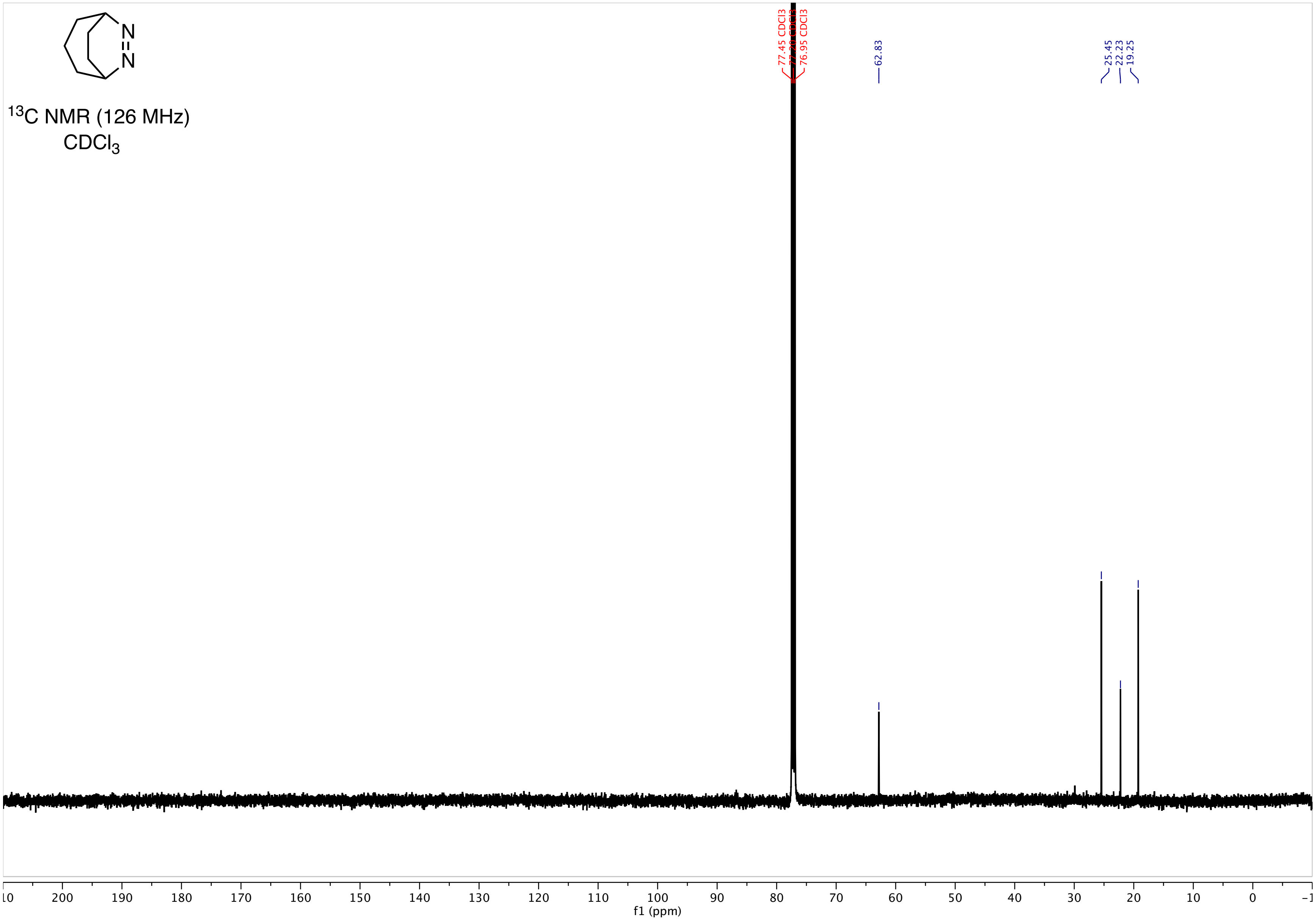

^{13}C NMR (126 MHz)
$CDCl_3$
77.45 CDCl3
77.20 CDCl3
76.95 CDCl3
62.83
25.45
22.23
19.25
f1 (ppm)

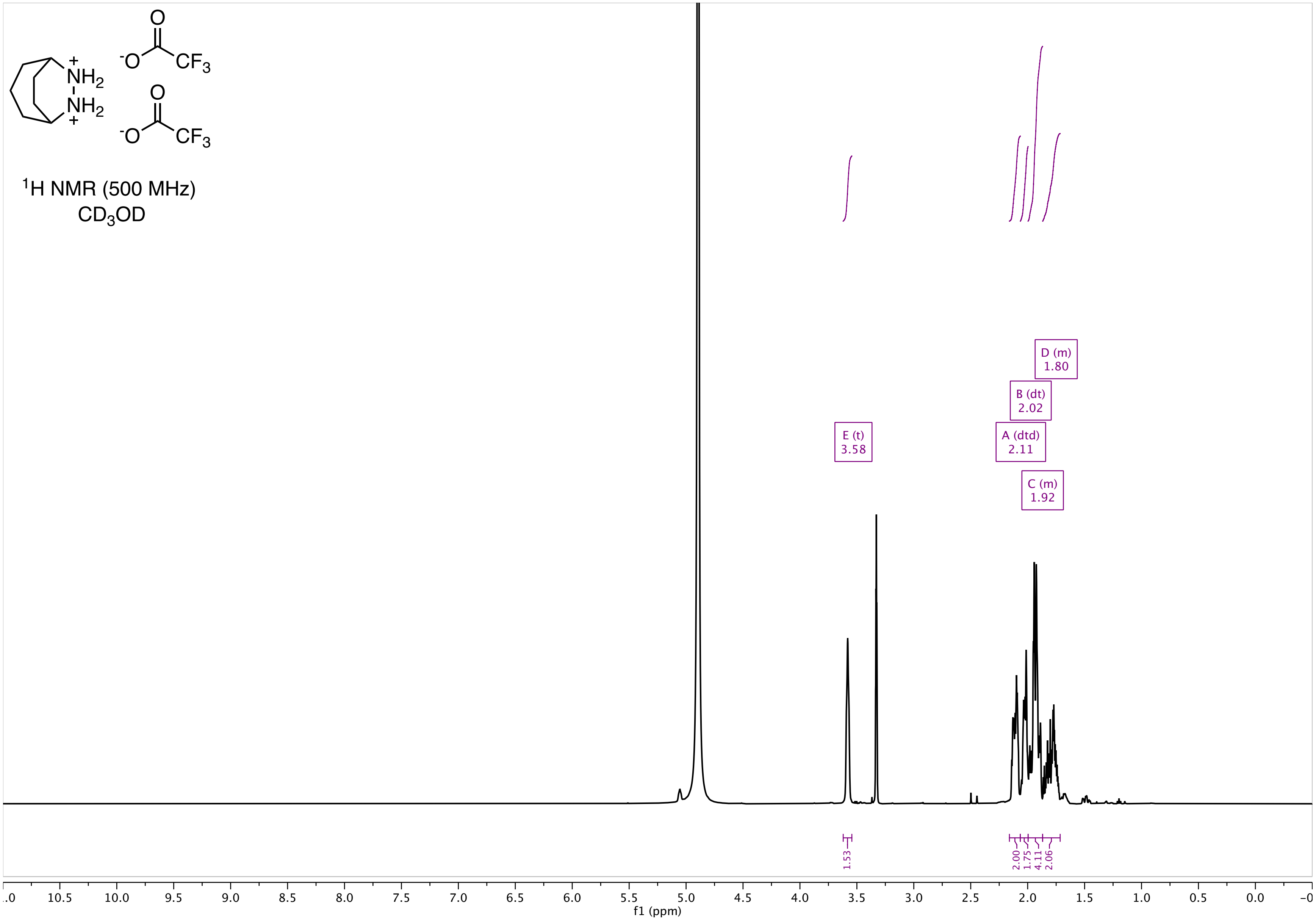

O
-O
CF3
NH2
NH2
1H NMR (500 MHz)
CD3OD
D (m)
1.80
B (dt)
2.02
E (t)
3.58
A (dtd)
2.11
C (m)
1.92
1.53
2.00
1.75
4.11
2.06
f1 (ppm)

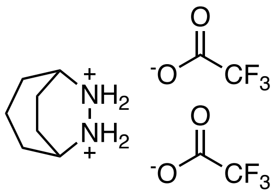

^{13}C NMR (126 MHz)
CD_3OD

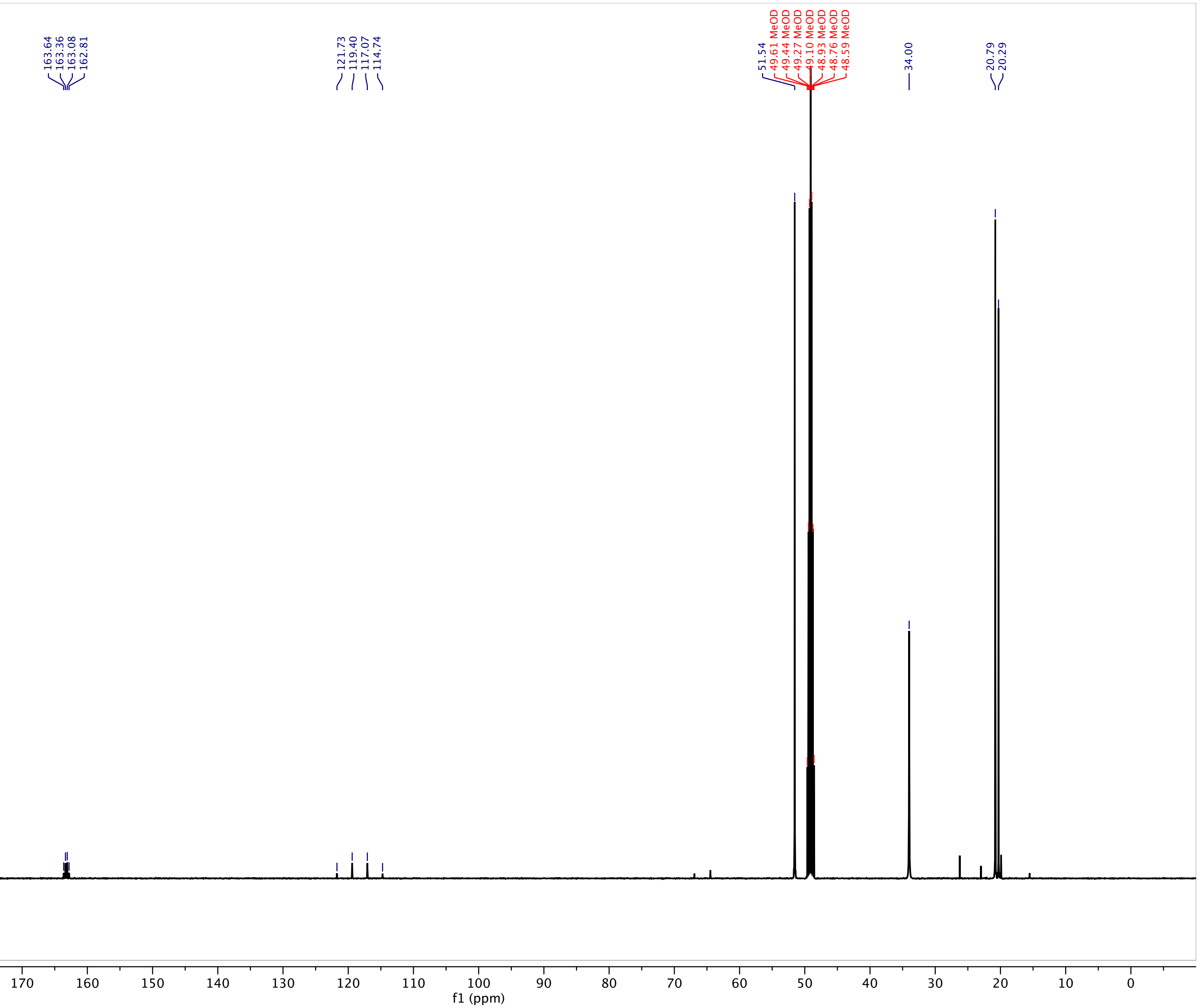

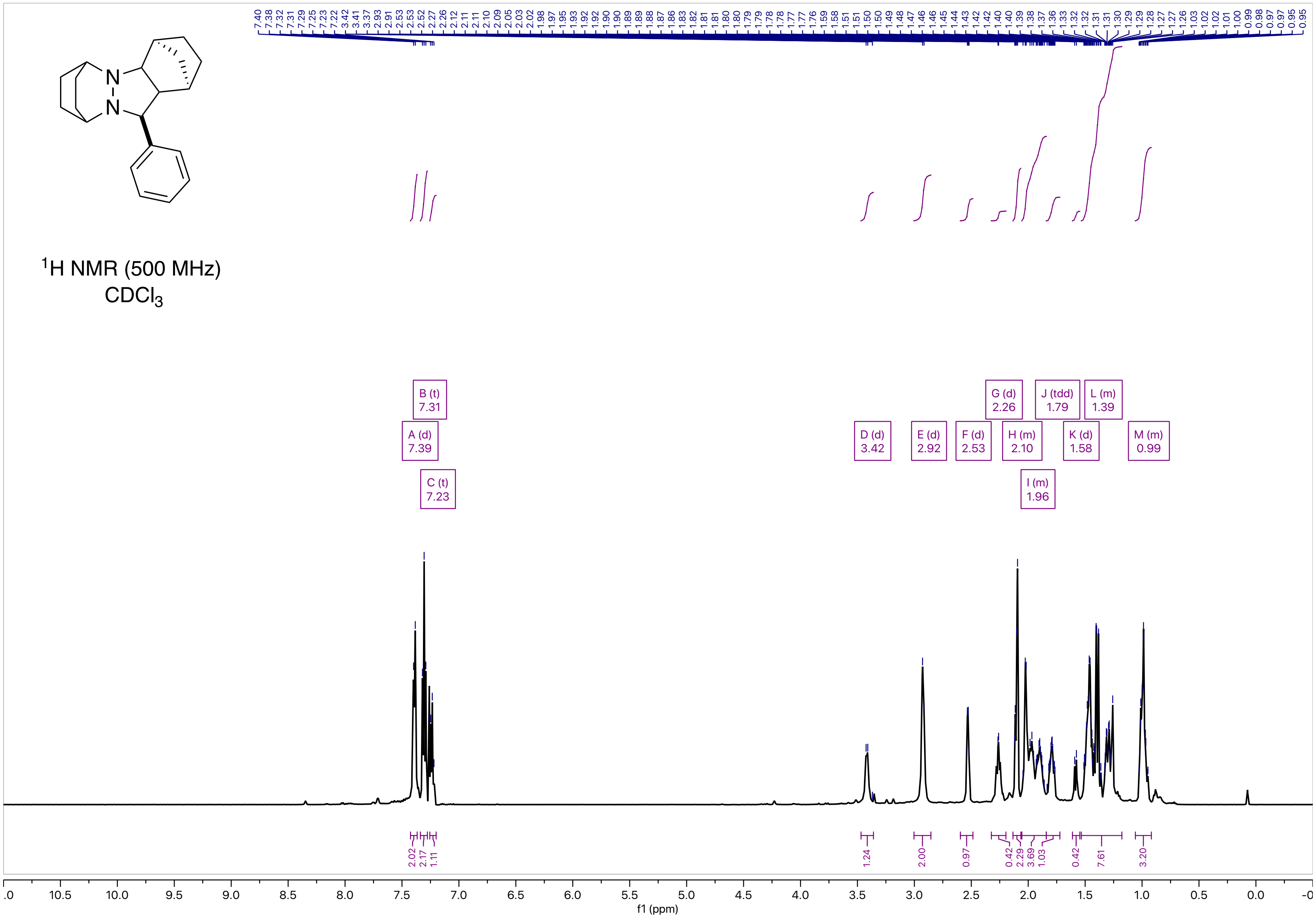

1H NMR (500 MHz)
CDCl3
A (d) 7.39
B (t) 7.31
C (t) 7.23
D (d) 3.42
E (d) 2.92
F (d) 2.53
G (d) 2.26
H (m) 2.10
I (m) 1.96
J (tdd) 1.79
K (d) 1.58
L (m) 1.39
M (m) 0.99
2.02
2.17
1.11
1.24
2.00
0.97
0.42
2.29
3.69
1.03
0.42
7.61
3.20
f1 (ppm)

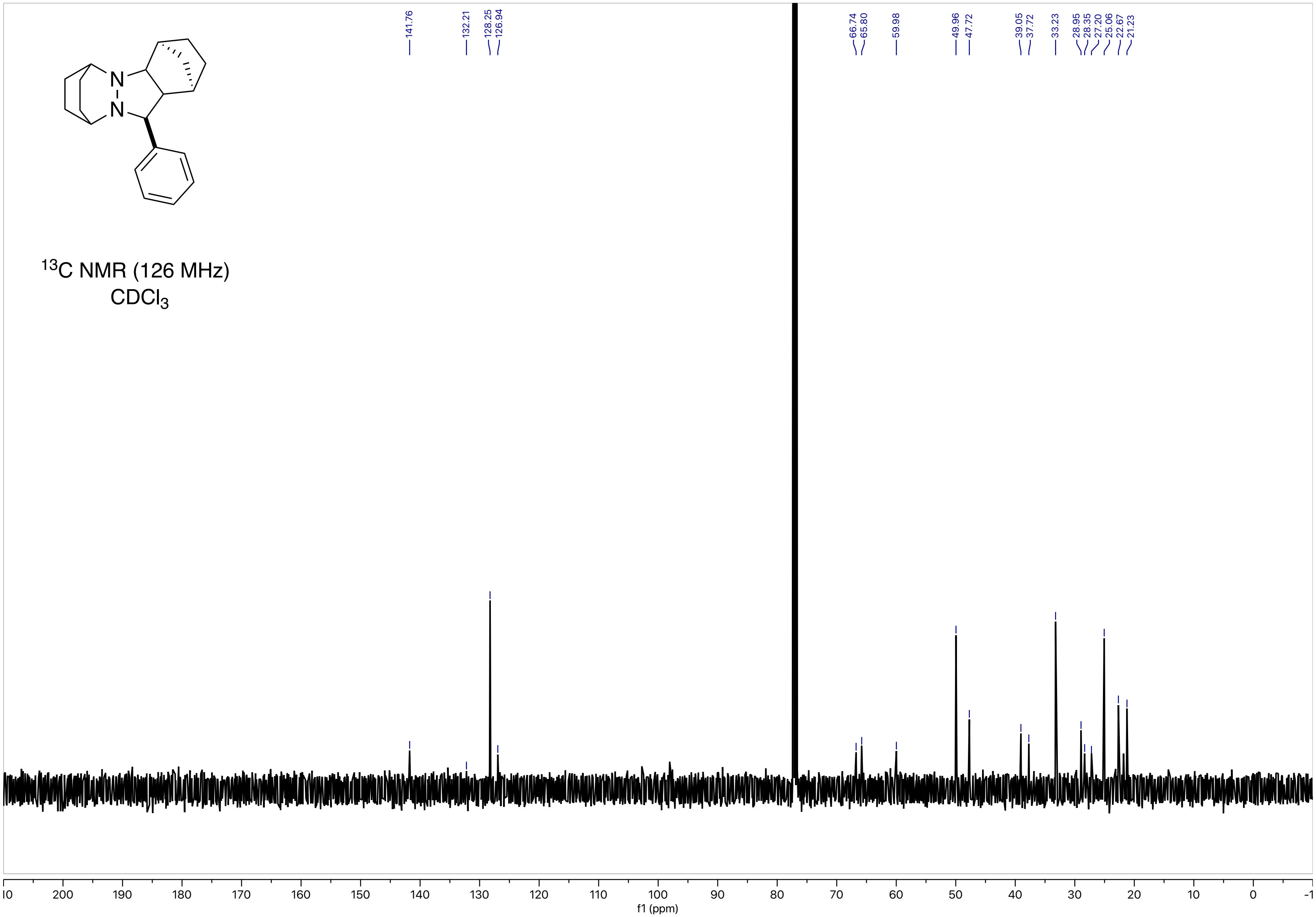
141.76
132.21
128.25
126.94
66.74
65.80
59.98
49.96
47.72
39.05
37.72
33.23
28.95
28.35
27.20
25.06
22.67
21.23
^{13}C NMR (126 MHz)
$CDCl_3$
200
190
180
170
160
150
140
130
120
110
100
90
80
70
60
50
40
30
20
10
0
f1 (ppm)

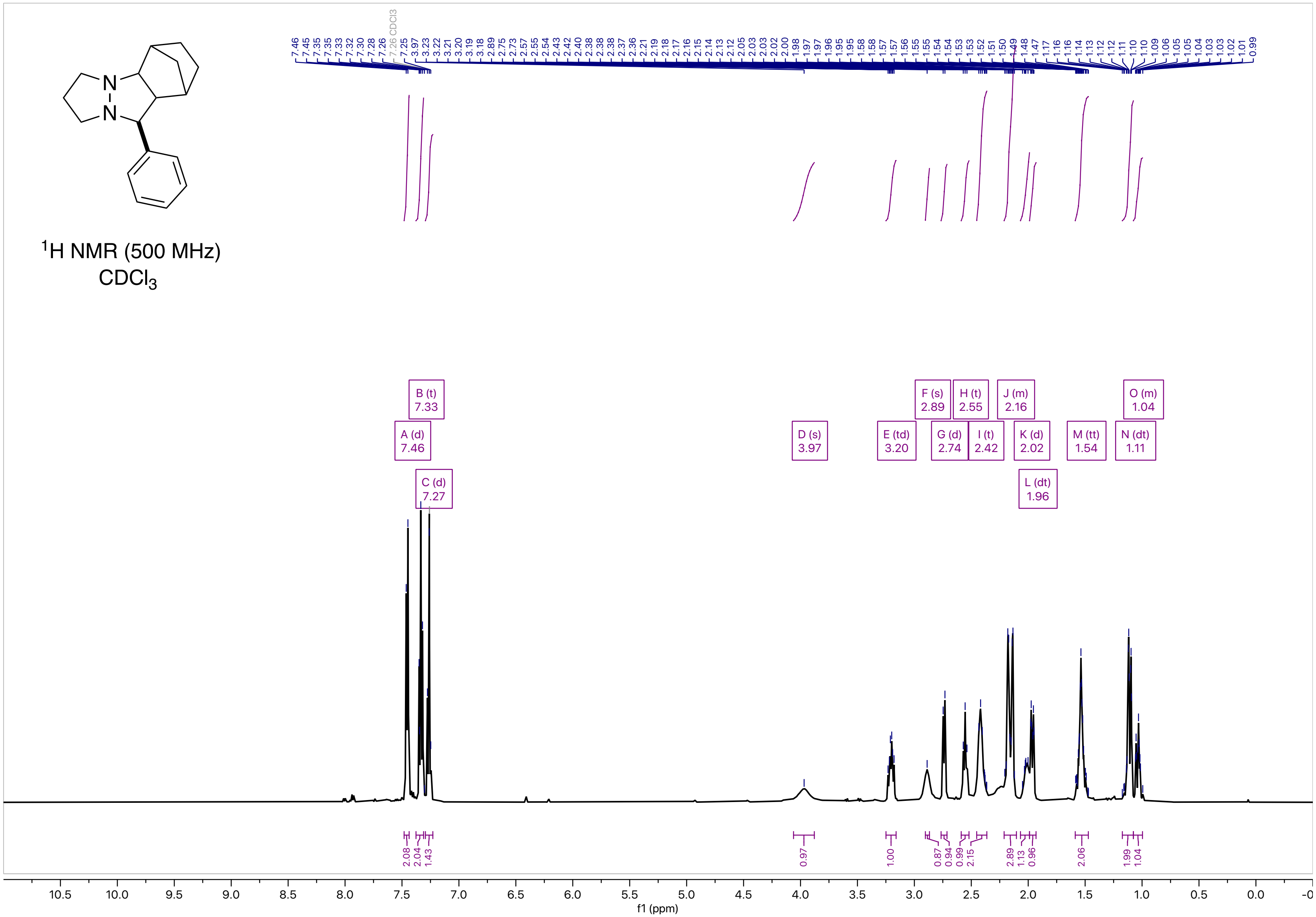

^{1}H NMR (500 MHz)
CDCl3
7.46 7.45 7.35 7.35 7.33 7.32 7.30 7.28 7.26 7.26 CDCl3 7.25 3.97 3.23 3.22 3.21 3.20 3.19 3.18 2.89 2.75 2.73 2.57 2.55 2.54 2.43 2.42 2.40 2.38 2.38 2.38 2.37 2.36 2.21 2.19 2.18 2.17 2.16 2.15 2.14 2.13 2.12 2.05 2.03 2.03 2.02 2.00 1.98 1.97 1.97 1.96 1.95 1.95 1.58 1.58 1.57 1.57 1.56 1.55 1.55 1.54 1.54 1.53 1.53 1.52 1.51 1.50 1.49 1.48 1.47 1.17 1.16 1.16 1.14 1.13 1.12 1.12 1.11 1.10 1.10 1.09 1.06 1.05 1.04 1.03 1.03 1.02 1.01 0.99
B (t) 7.33
A (d) 7.46
C (d) 7.27
D (s) 3.97
E (td) 3.20
F (s) 2.89
G (d) 2.74
H (t) 2.55
I (t) 2.42
J (m) 2.16
K (d) 2.02
L (dt) 1.96
M (tt) 1.54
N (dt) 1.11
O (m) 1.04
2.08 2.04 1.43 0.97 1.00 0.87 0.94 0.99 2.15 2.89 1.13 0.96 2.06 1.99 1.04
10.5 10.0 9.5 9.0 8.5 8.0 7.5 7.0 6.5 6.0 5.5 5.0 4.5 4.0 3.5 3.0 2.5 2.0 1.5 1.0 0.5 0.0 -0
f1 (ppm)

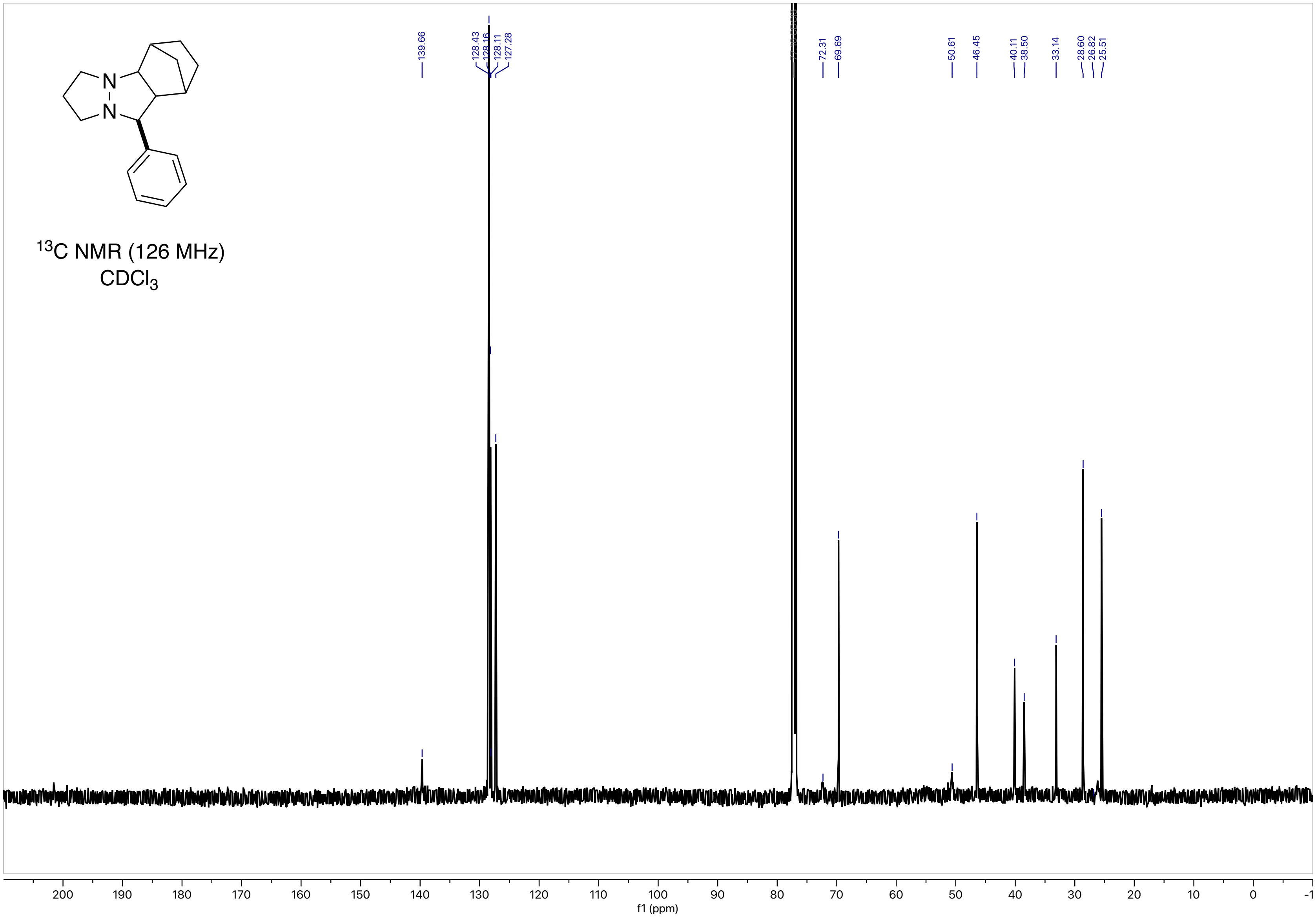

^{13}C NMR (126 MHz)
CDCl$_3$
139.66
128.43
128.16
128.11
127.28
72.31
69.69
50.61
46.45
40.11
38.50
33.14
28.60
26.82
25.51
200 190 180 170 160 150 140 130 120 110 100 90 80 70 60 50 40 30 20 10 0 -1
f1 (ppm)

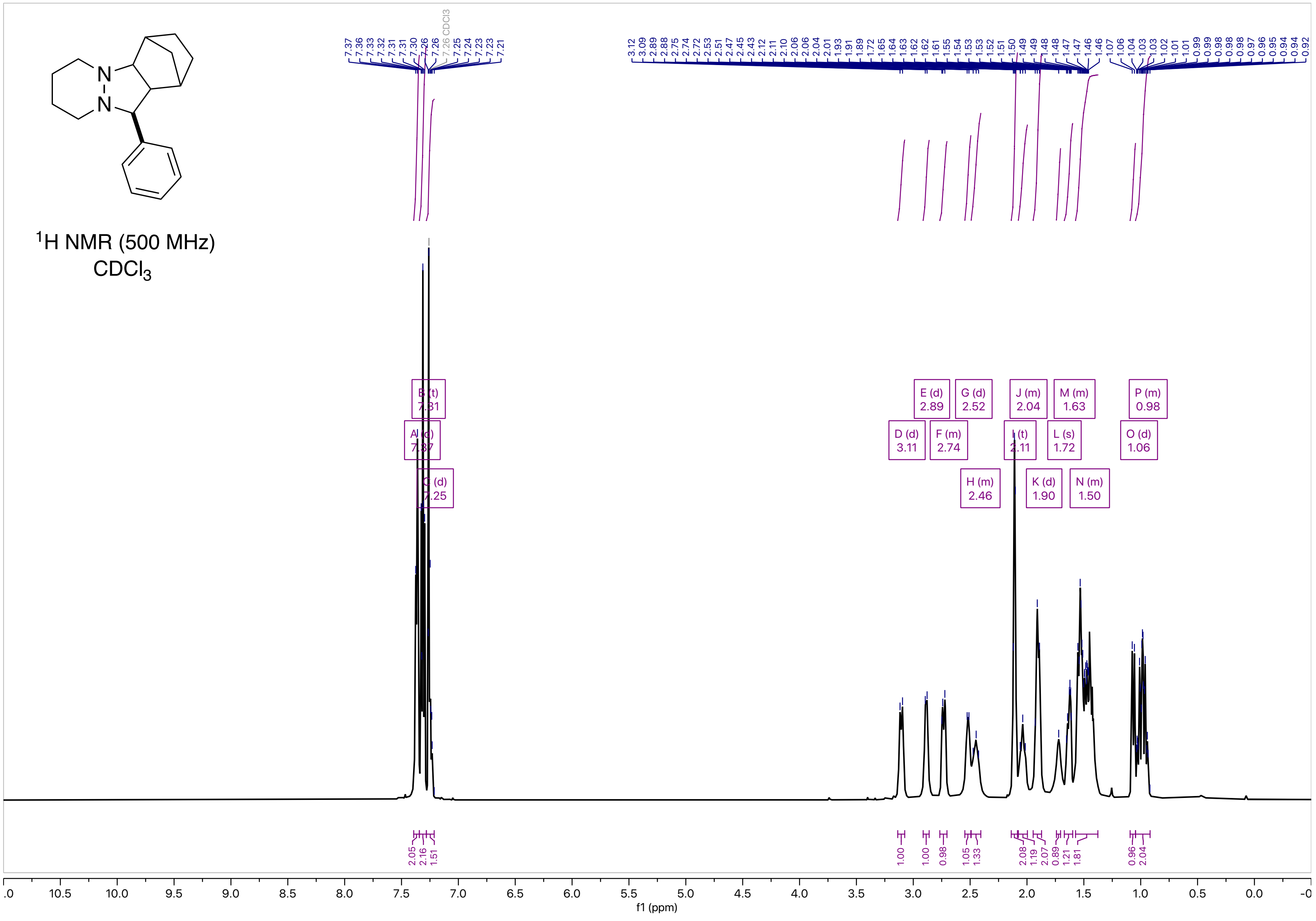

1H NMR (500 MHz)
CDCl3
A (d) 7.37
B (t) 7.31
C (d) 7.25
D (d) 3.11
E (d) 2.89
F (m) 2.74
G (d) 2.52
H (m) 2.46
I (t) 2.11
J (m) 2.04
K (d) 1.90
L (s) 1.72
M (m) 1.63
N (m) 1.50
O (d) 1.06
P (m) 0.98
f1 (ppm)

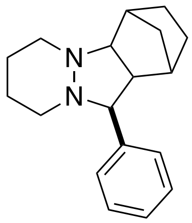

^{13}C NMR (126 MHz)
$CDCl_3$

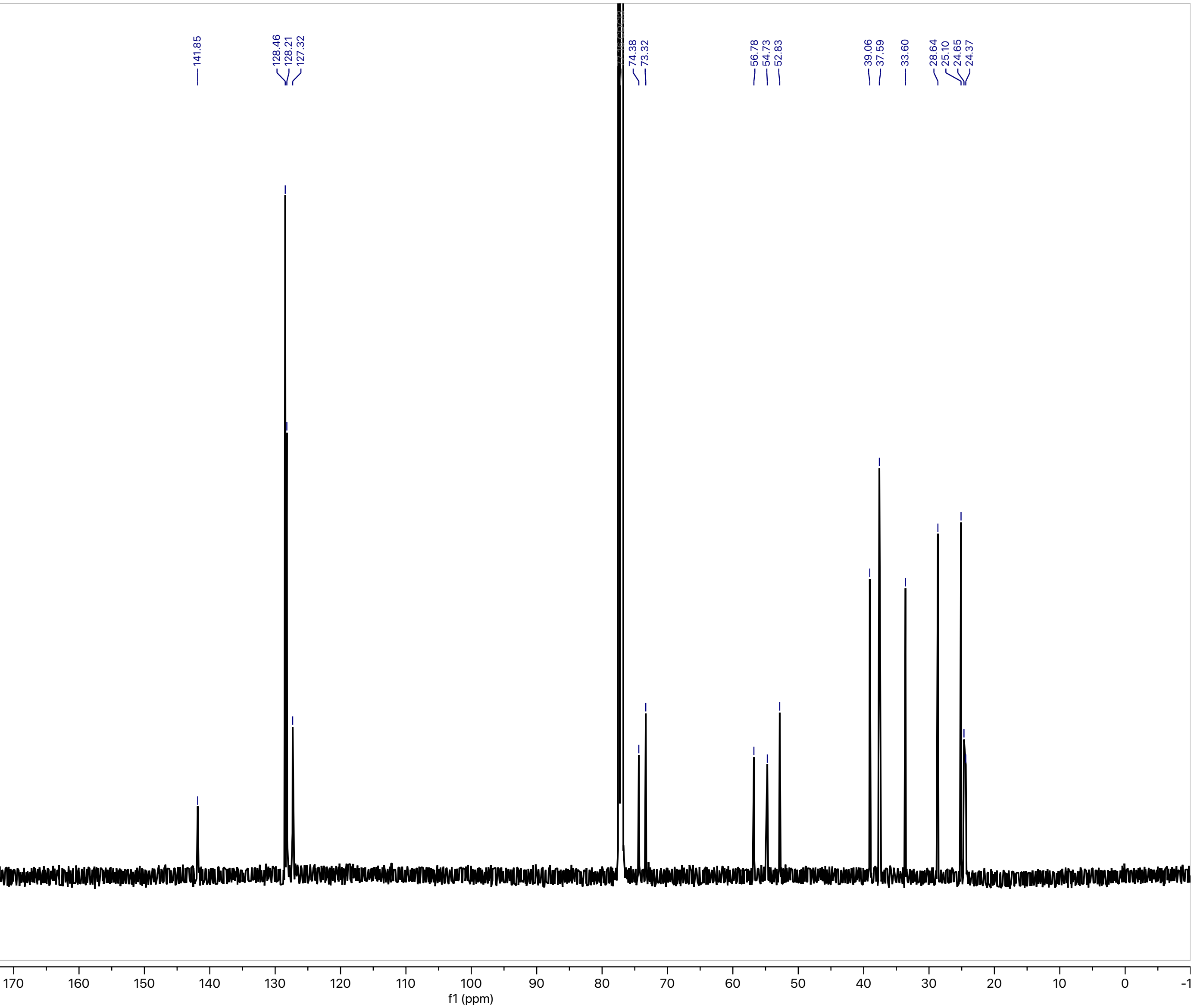

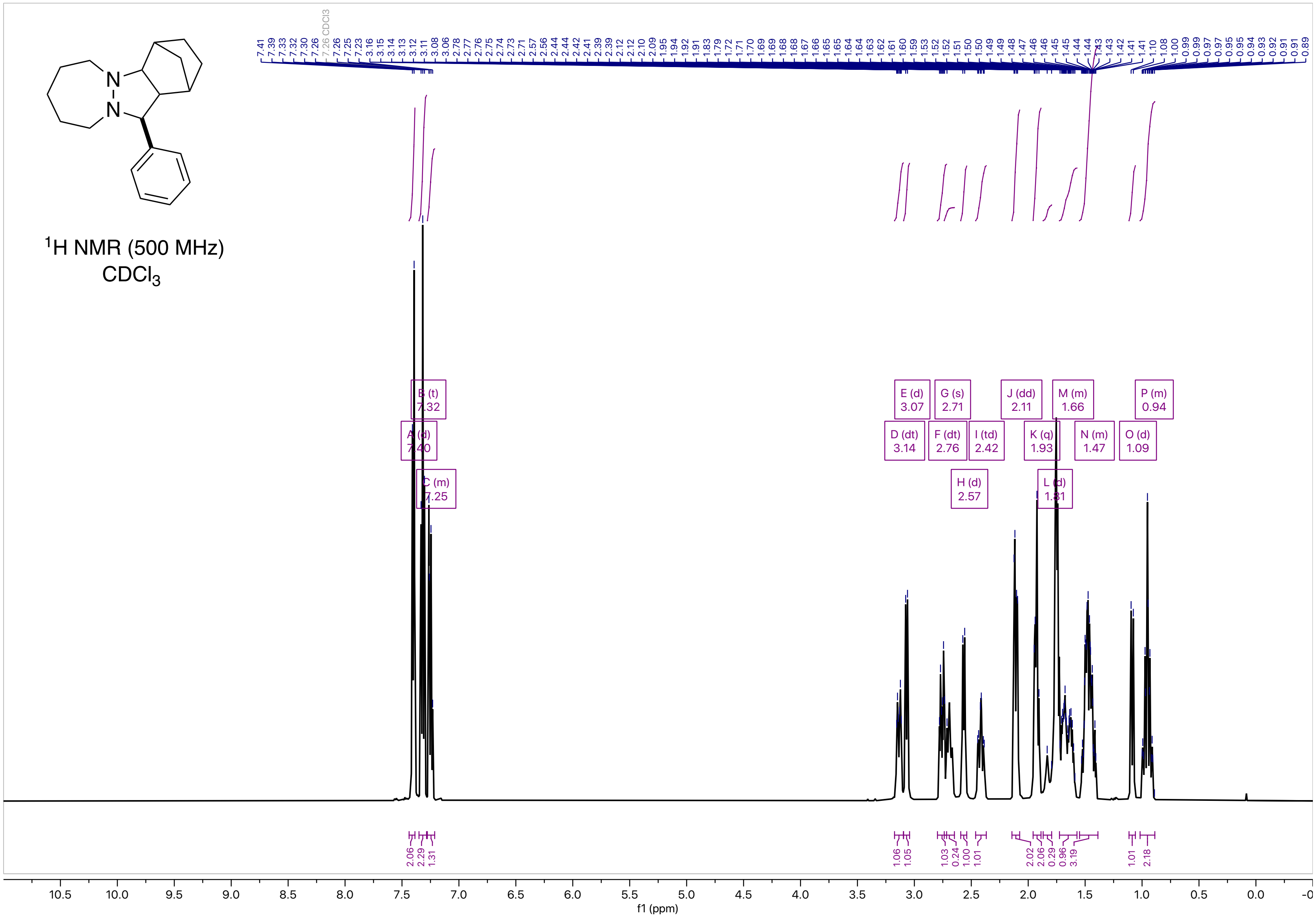

N
N
1H NMR (500 MHz)
CDCl3
A (d) 7.40
B (t) 7.32
C (m) 7.25
D (dt) 3.14
E (d) 3.07
F (dt) 2.76
G (s) 2.71
H (d) 2.57
I (td) 2.42
J (dd) 2.11
K (q) 1.93
L (d) 1.81
M (m) 1.66
N (m) 1.47
O (d) 1.09
P (m) 0.94
f1 (ppm)

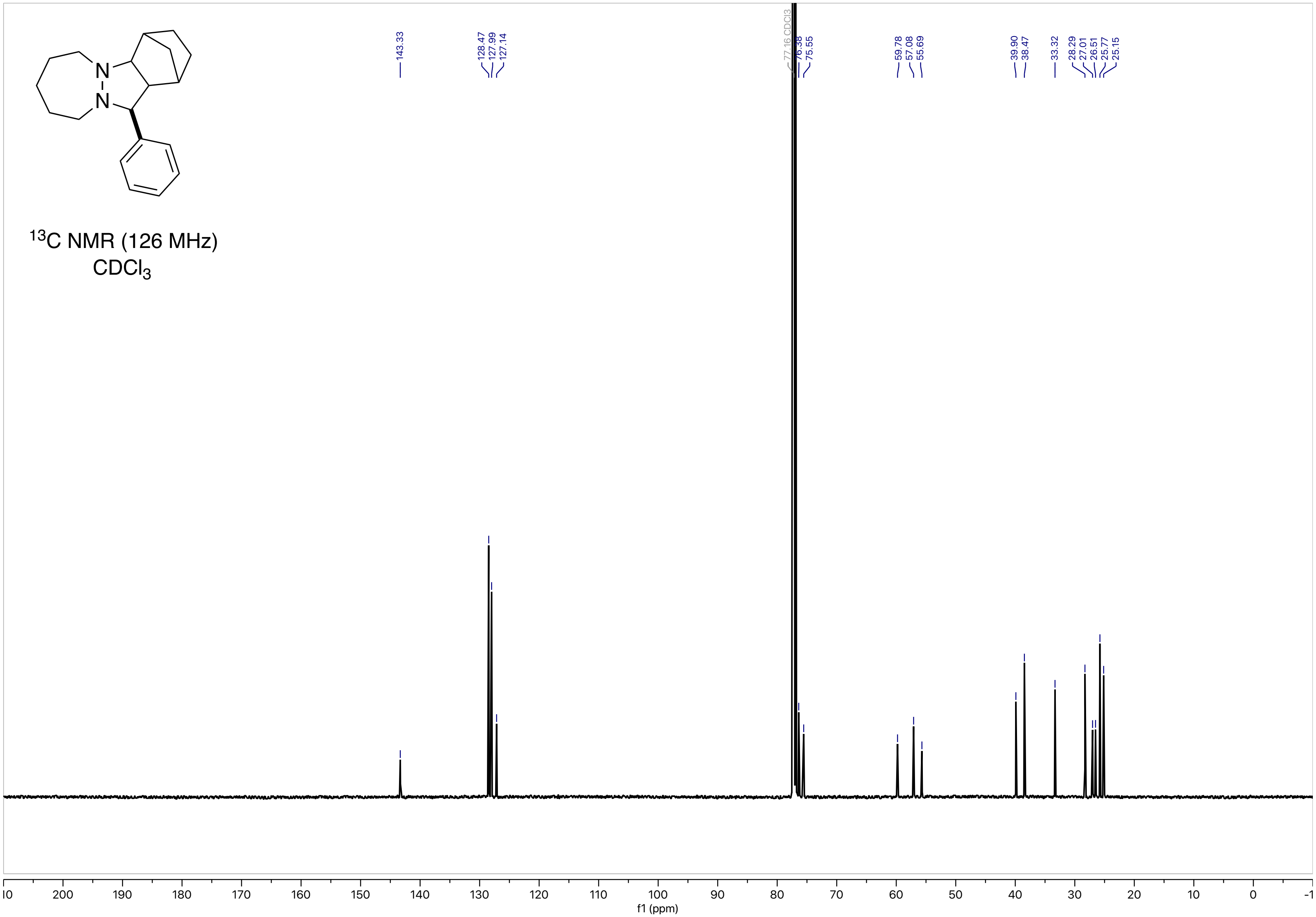

^{13}C NMR (126 MHz)
$CDCl_3$
143.33
128.47
127.99
127.14
77.16 CDCl3
76.38
75.55
59.78
57.08
55.69
39.90
38.47
33.32
28.29
27.01
26.51
25.77
25.15
f1 (ppm)

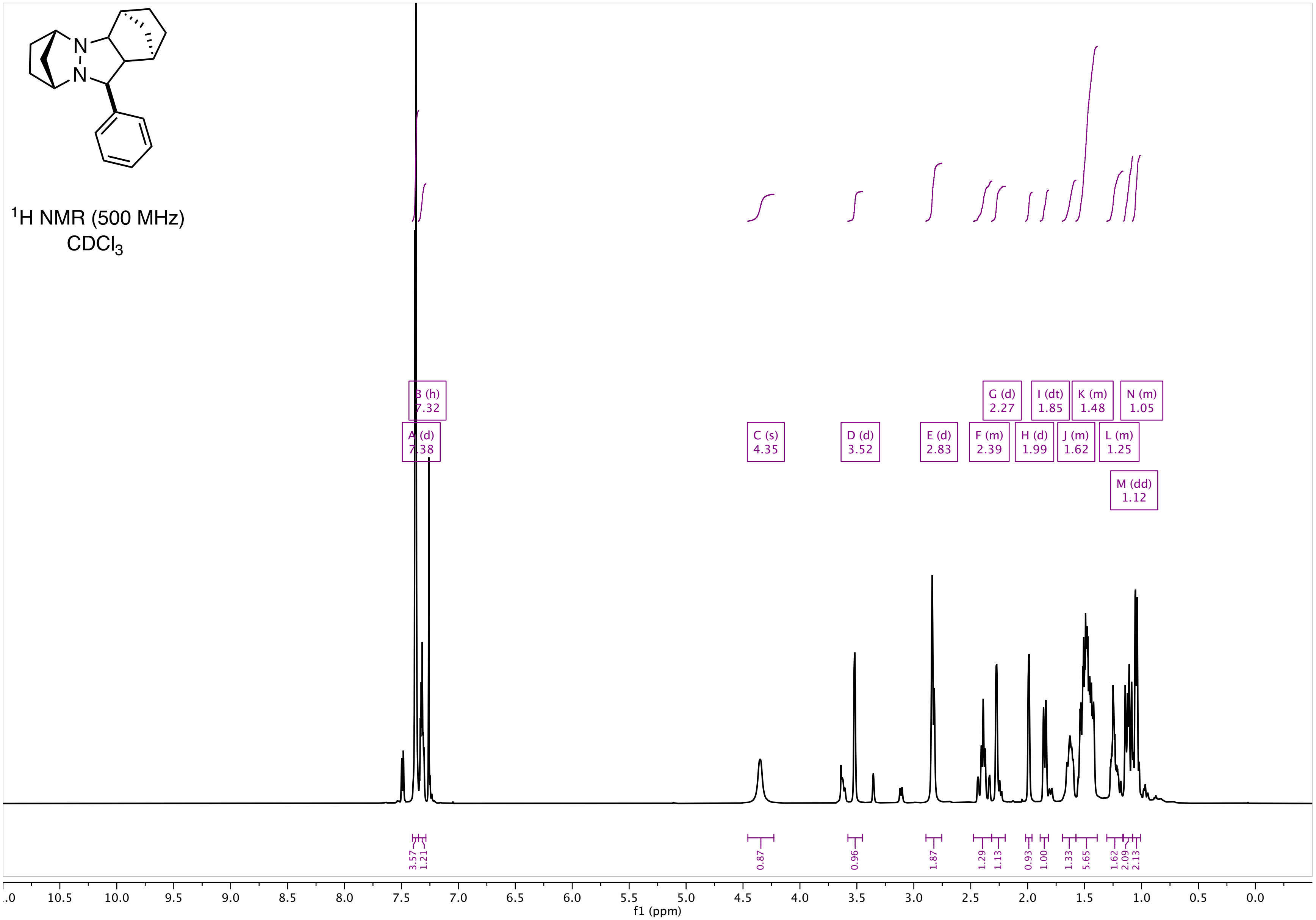
N
N
¹H NMR (500 MHz)
CDCl3
B (h) 7.32
A (d) 7.38
C (s) 4.35
D (d) 3.52
E (d) 2.83
F (m) 2.39
G (d) 2.27
H (d) 1.99
I (dt) 1.85
J (m) 1.62
K (m) 1.48
L (m) 1.25
M (dd) 1.12
N (m) 1.05
3.57
1.21
0.87
0.96
1.87
1.29
1.13
0.93
1.00
1.33
5.65
1.62
2.09
2.13
f1 (ppm)

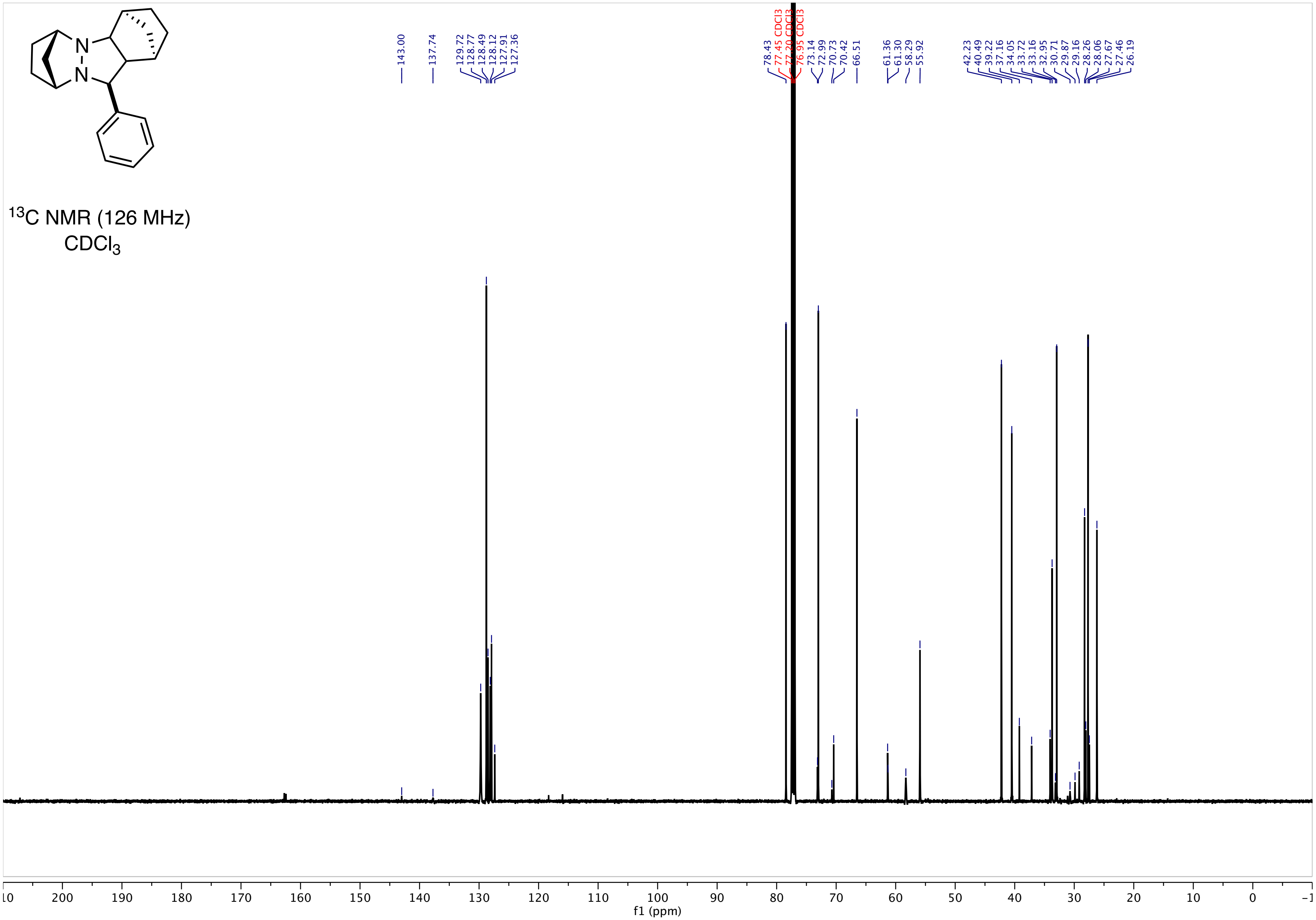

^{13}C NMR (126 MHz)
$CDCl_3$
143.00
137.74
129.72
128.77
128.49
128.12
127.91
127.36
78.43
77.45 CDCl3
77.20 CDCl3
76.95 CDCl3
73.14
72.99
70.73
70.42
66.51
61.36
61.30
58.29
55.92
42.23
40.49
39.22
37.16
34.05
33.72
33.16
32.95
30.71
29.87
29.16
28.26
28.06
27.67
27.46
26.19
f1 (ppm)

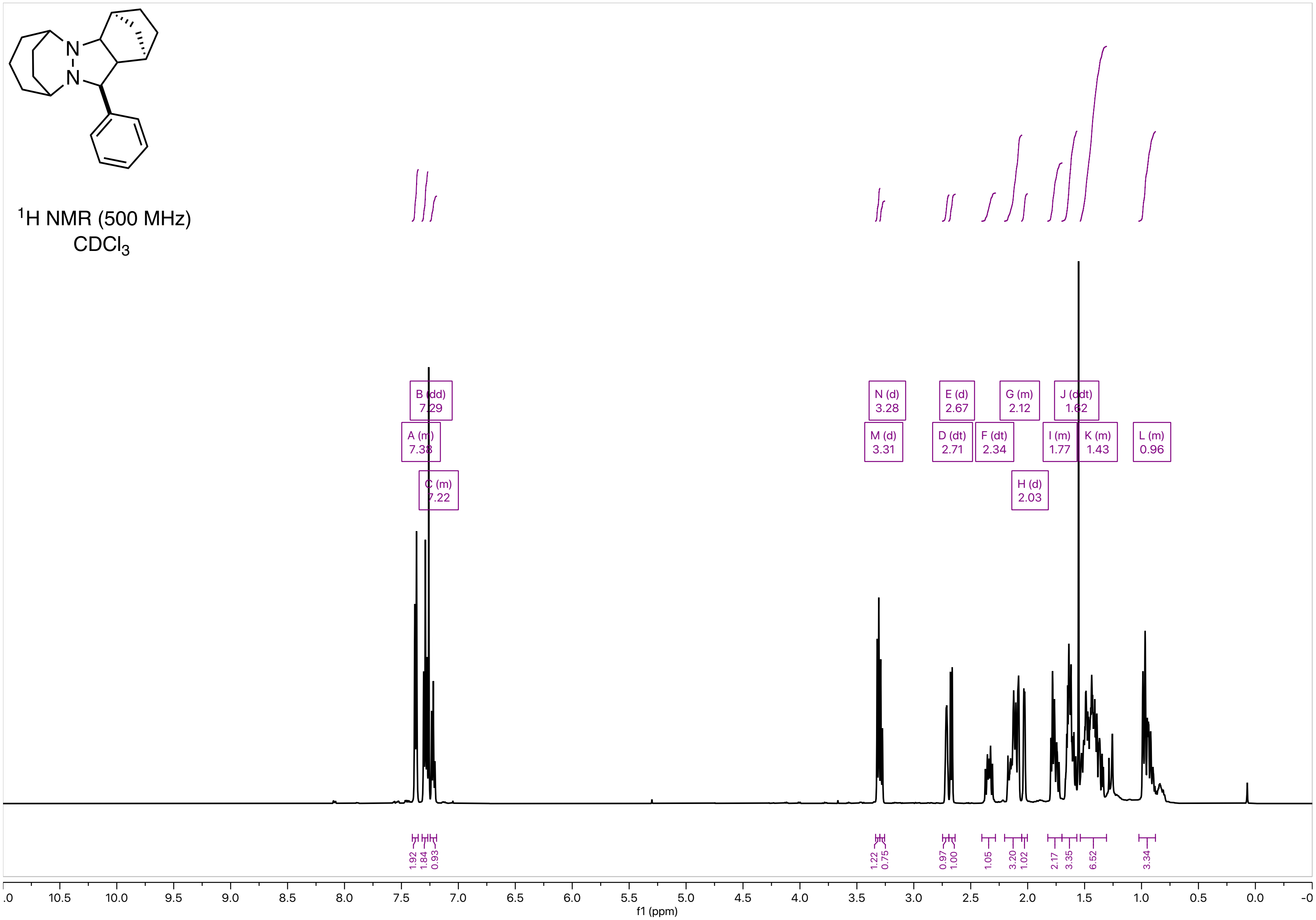

N
N
1H NMR (500 MHz)
CDCl3
B (dd) 7.29
A (m) 7.38
C (m) 7.22
N (d) 3.28
M (d) 3.31
E (d) 2.67
D (dt) 2.71
F (dt) 2.34
G (m) 2.12
H (d) 2.03
J (ddt) 1.62
I (m) 1.77
K (m) 1.43
L (m) 0.96
1.92
1.84
0.93
1.22
0.75
0.97
1.00
1.05
3.20
1.02
2.17
3.35
6.52
3.34
10.5
10.0
9.5
9.0
8.5
8.0
7.5
7.0
6.5
6.0
5.5
5.0
4.5
4.0
3.5
3.0
2.5
2.0
1.5
1.0
0.5
0.0
f1 (ppm)

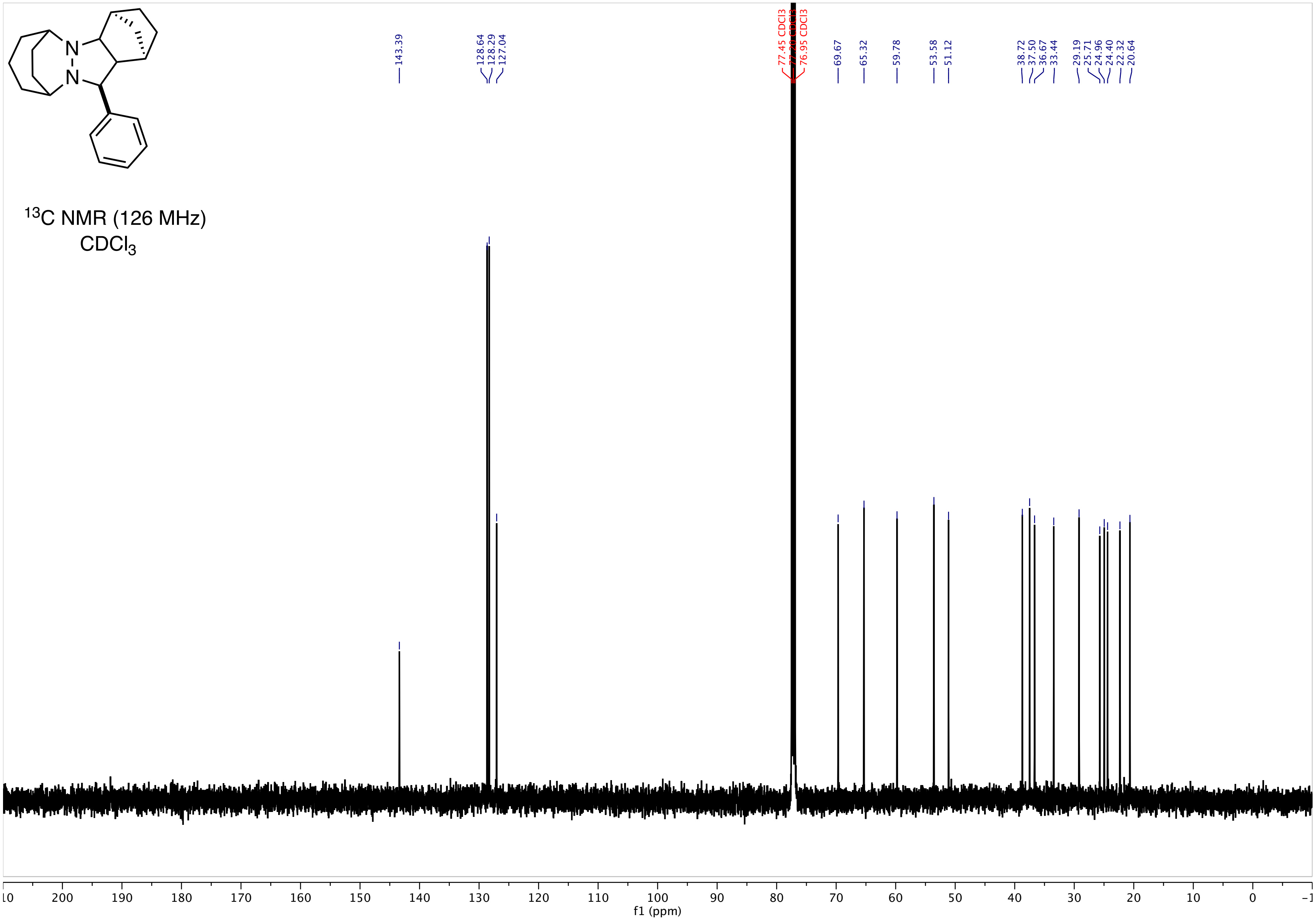

^{13}C NMR (126 MHz)
CDCl$_3$
143.39
128.64
128.29
127.04
77.45 CDCl3
77.20 CDCl3
76.95 CDCl3
69.67
65.32
59.78
53.58
51.12
38.72
37.50
36.67
33.44
29.19
25.71
24.96
24.40
22.32
20.64
f1 (ppm)

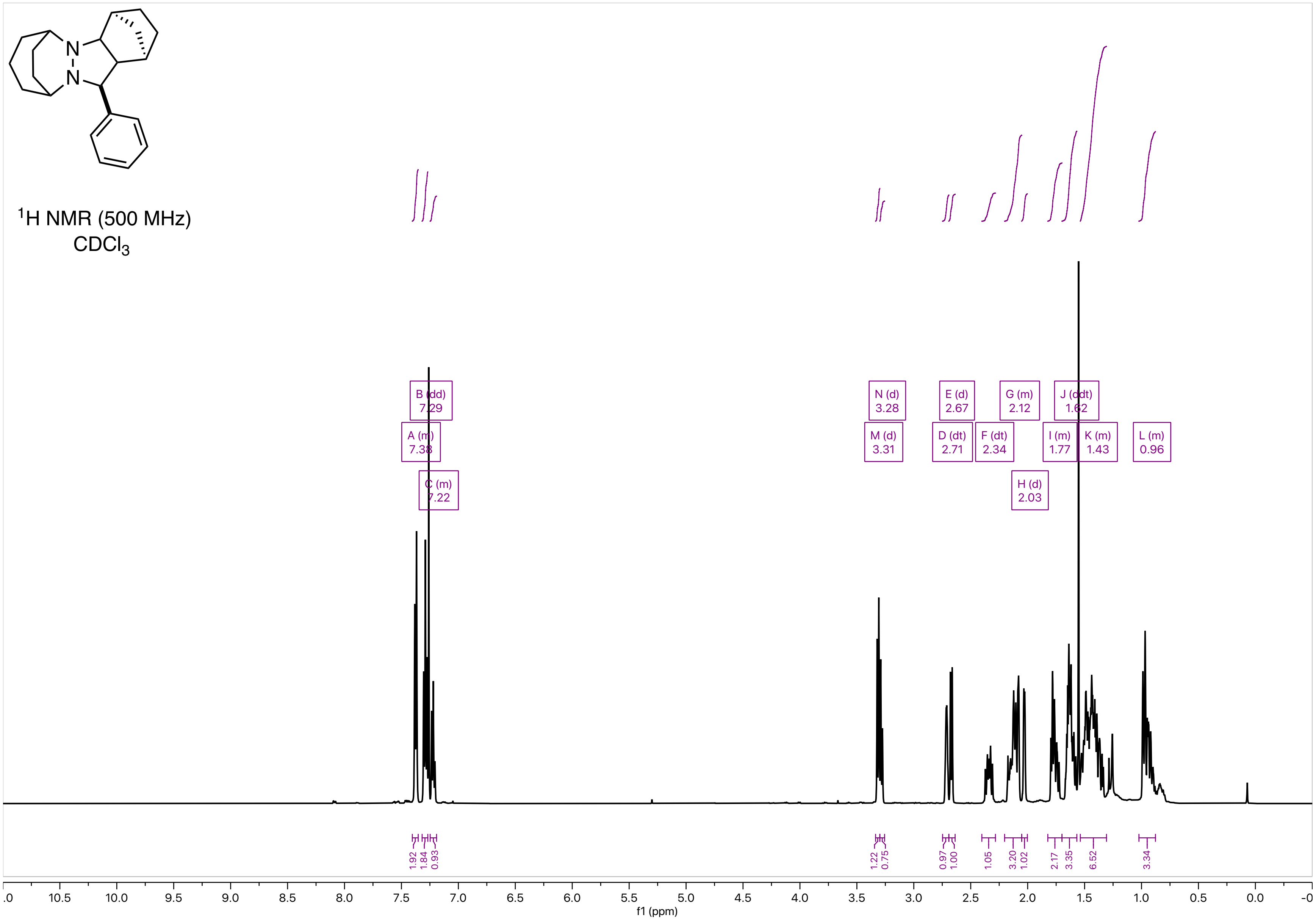

1H NMR (500 MHz)
CDCl3
A (m) 7.38
B (dd) 7.29
C (m) 7.22
N (d) 3.28
M (d) 3.31
E (d) 2.67
D (dt) 2.71
F (dt) 2.34
G (m) 2.12
H (d) 2.03
J (ddt) 1.62
I (m) 1.77
K (m) 1.43
L (m) 0.96
1.92 1.84 0.93 1.22 0.75 0.97 1.00 1.05 3.20 1.02 2.17 3.35 6.52 3.34
f1 (ppm)

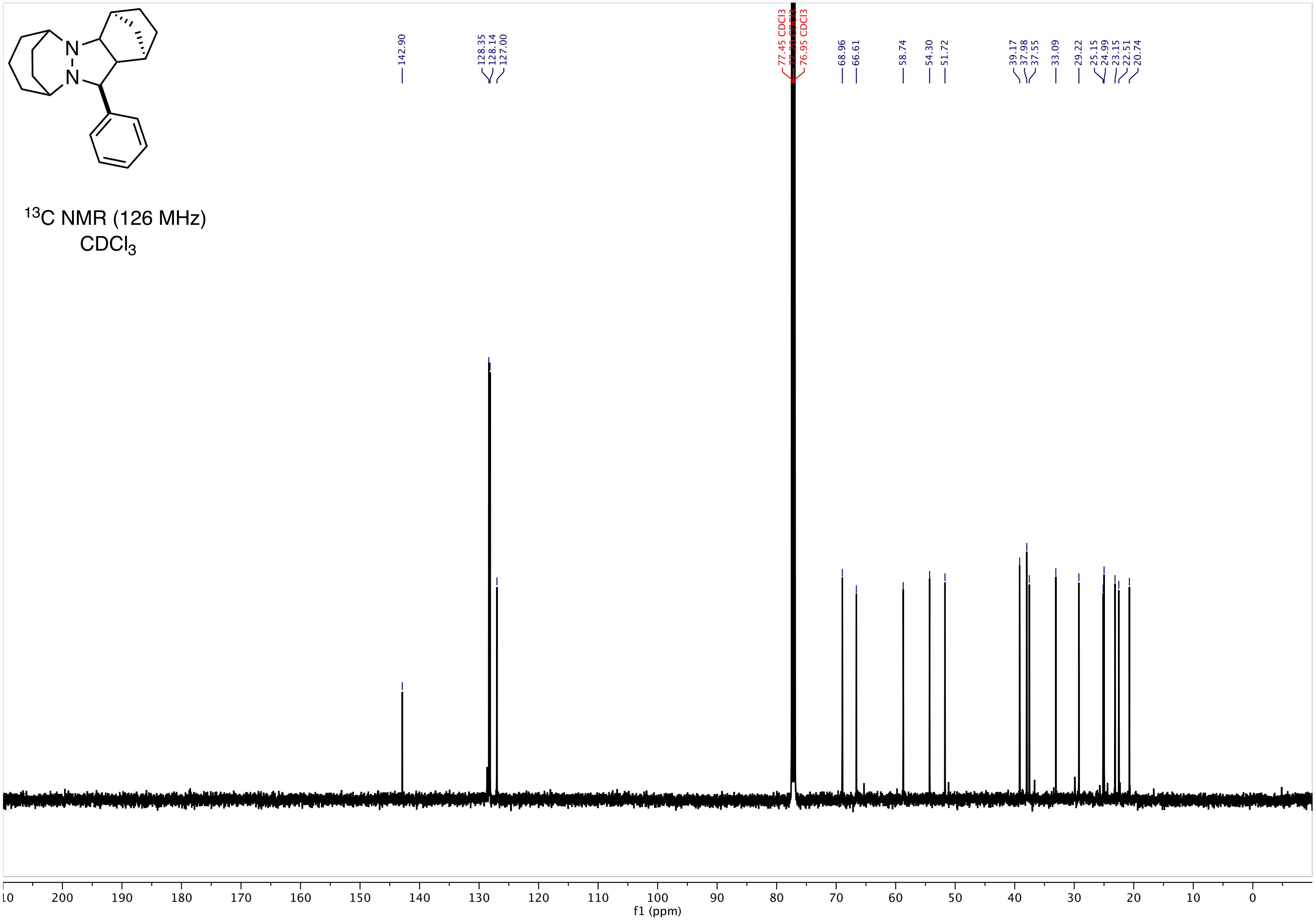

N
N
^{13}C NMR (126 MHz)
CDCl$_3$
142.90
128.35
128.14
127.00
77.45 CDCl3
77.20 CDCl3
76.95 CDCl3
68.96
66.61
58.74
54.30
51.72
39.17
37.98
37.55
33.09
29.22
25.15
24.99
23.15
22.51
20.74
f1 (ppm)

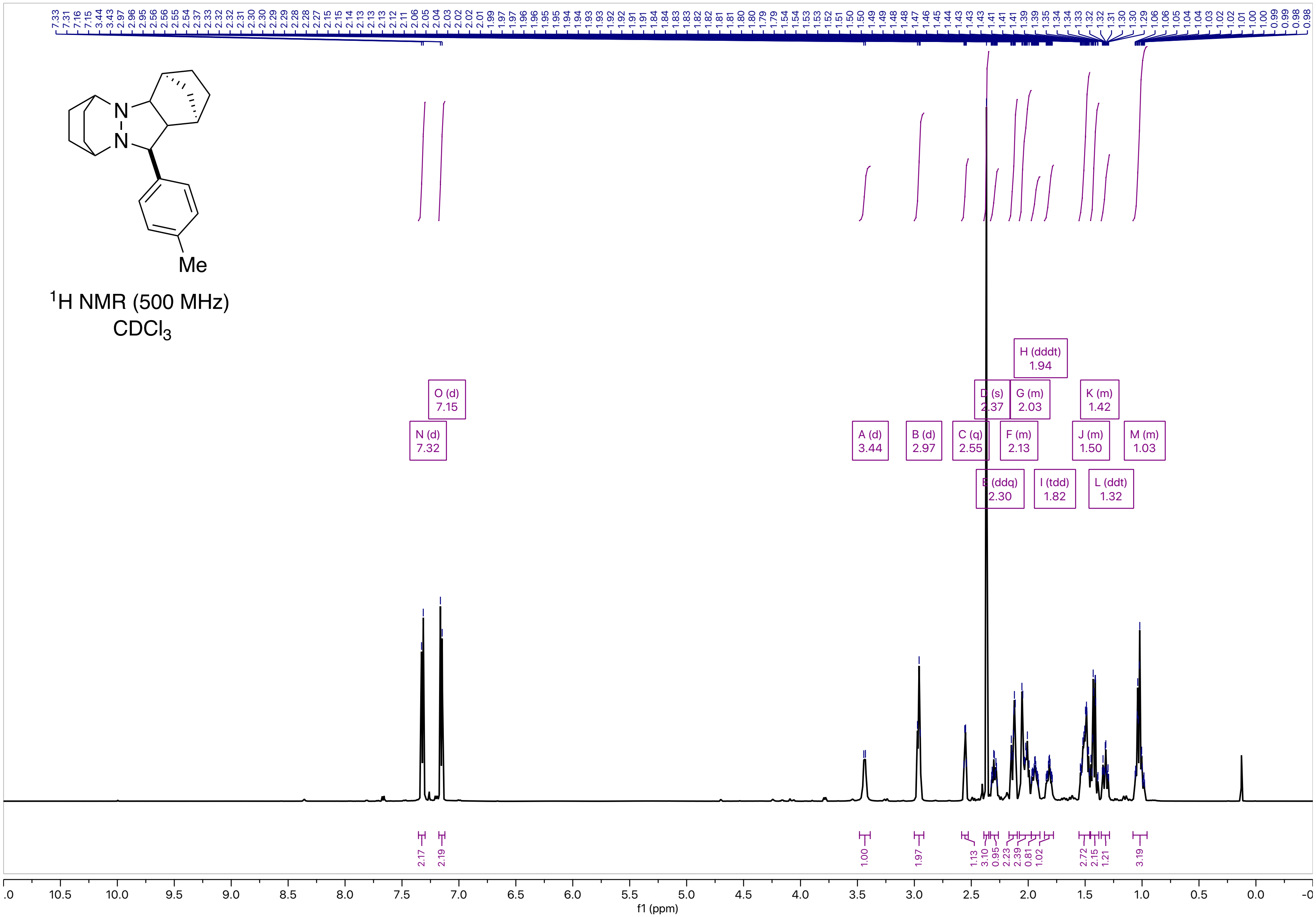
Me
¹H NMR (500 MHz)
CDCl₃
N (d) 7.32
O (d) 7.15
A (d) 3.44
B (d) 2.97
C (q) 2.55
D (s) 2.37
E (ddq) 2.30
F (m) 2.13
G (m) 2.03
H (dddt) 1.94
I (tdd) 1.82
J (m) 1.50
K (m) 1.42
L (ddt) 1.32
M (m) 1.03
f1 (ppm)

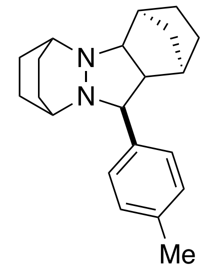

^{13}C NMR (126 MHz)
$CDCl_3$

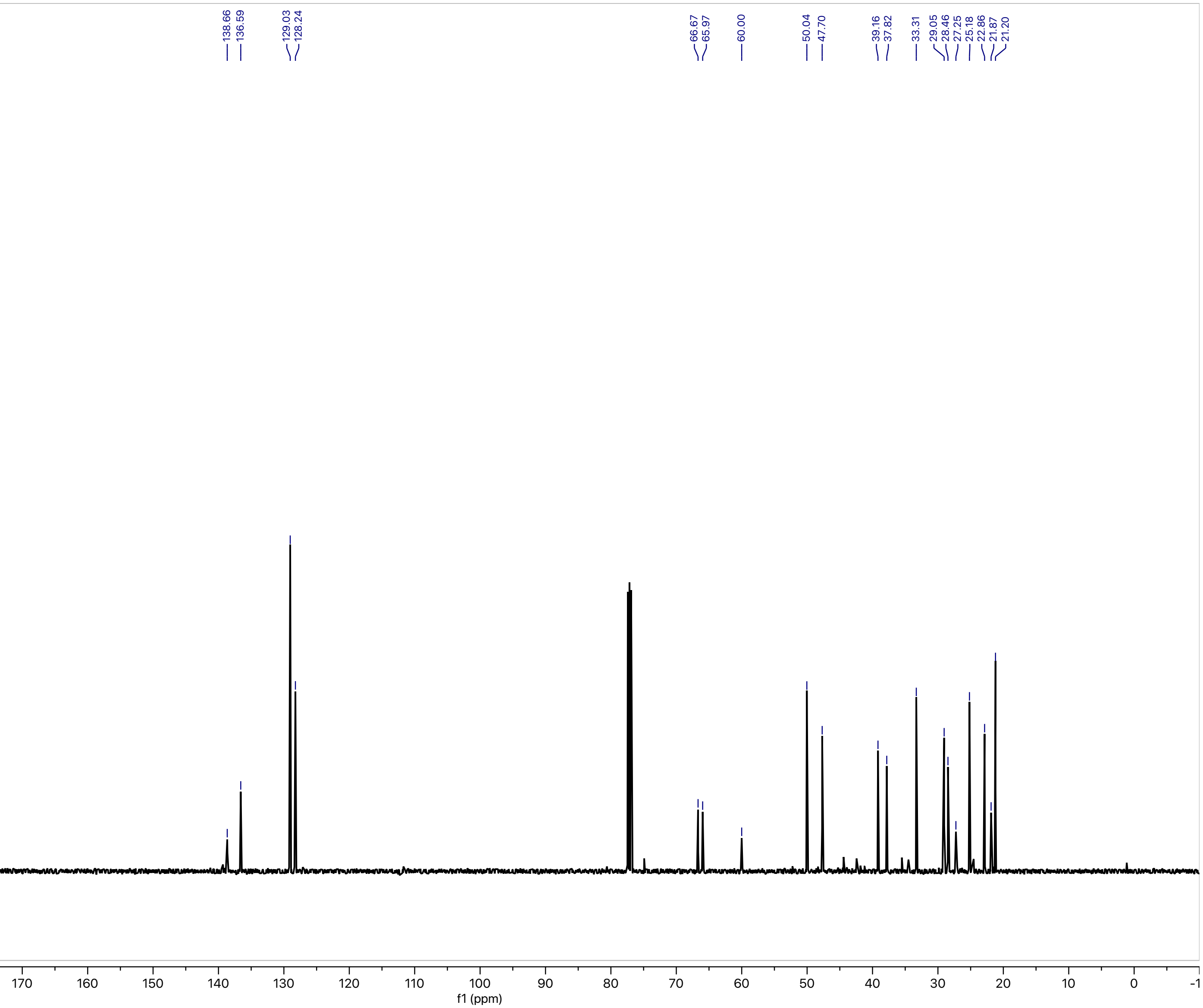

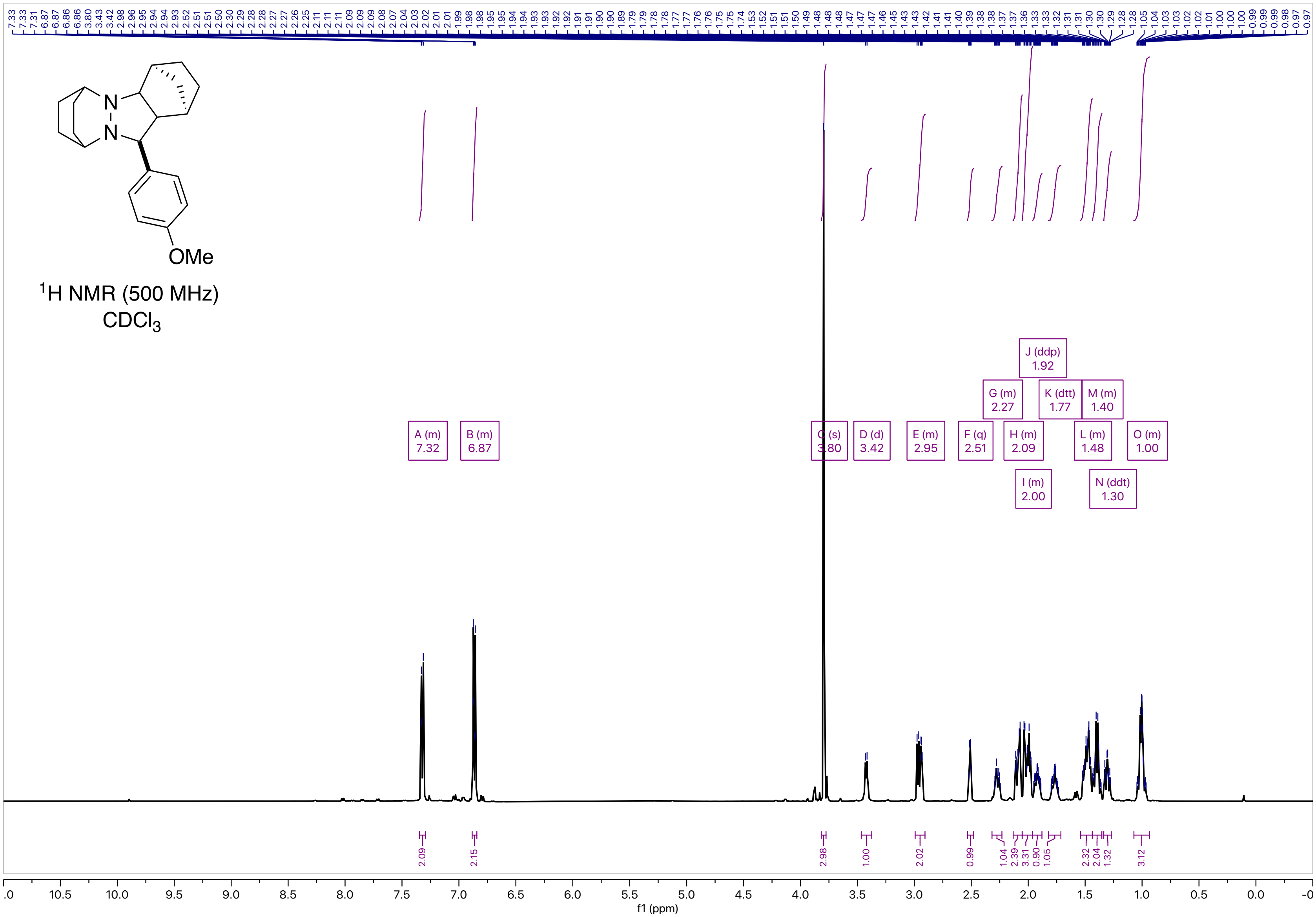
^{1}H NMR (500 MHz)
$CDCl_3$
OMe
A (m) 7.32
B (m) 6.87
C (s) 3.80
D (d) 3.42
E (m) 2.95
F (q) 2.51
G (m) 2.27
H (m) 2.09
I (m) 2.00
J (ddp) 1.92
K (dtt) 1.77
L (m) 1.48
M (m) 1.40
N (ddt) 1.30
O (m) 1.00
f1 (ppm)

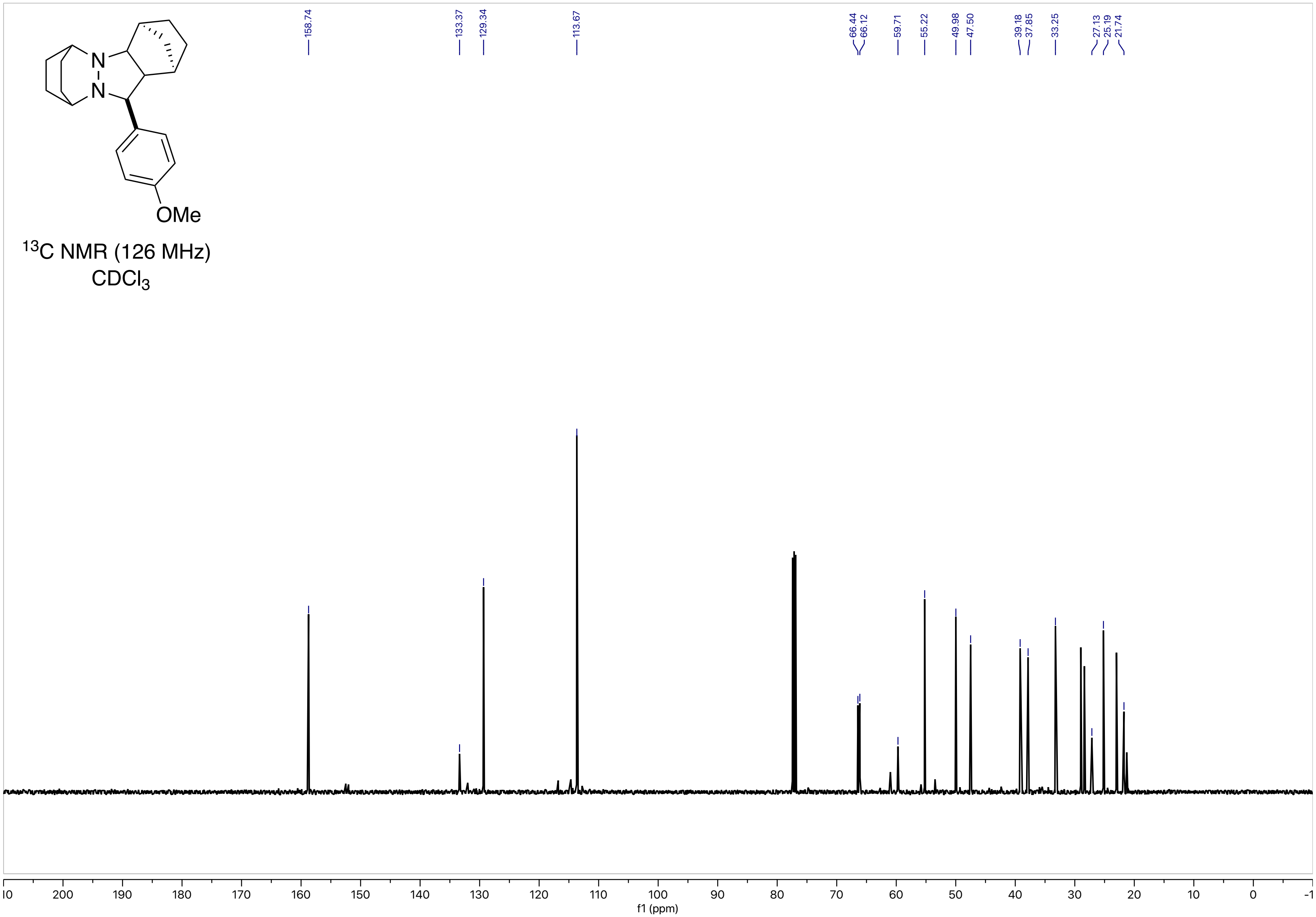
OMe
13C NMR (126 MHz)
CDCl3
158.74
133.37
129.34
113.67
66.44
66.12
59.71
55.22
49.98
47.50
39.18
37.85
33.25
27.13
25.19
21.74
f1 (ppm)

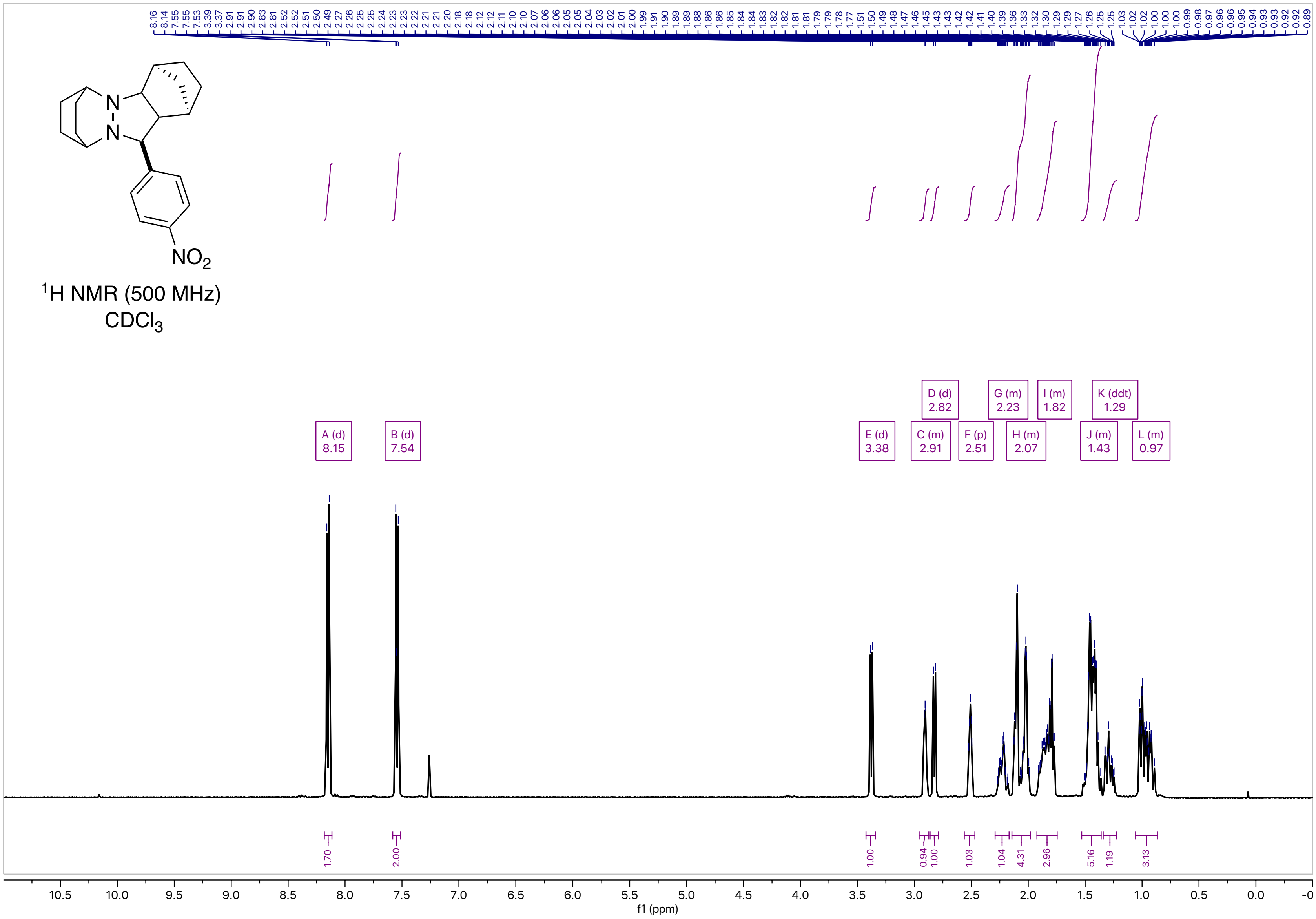

NO2
1H NMR (500 MHz)
CDCl3
A (d) 8.15
B (d) 7.54
E (d) 3.38
C (m) 2.91
D (d) 2.82
F (p) 2.51
G (m) 2.23
H (m) 2.07
I (m) 1.82
J (m) 1.43
K (ddt) 1.29
L (m) 0.97
1.70
2.00
1.00
0.94
1.00
1.03
1.04
4.31
2.96
5.16
1.19
3.13
f1 (ppm)

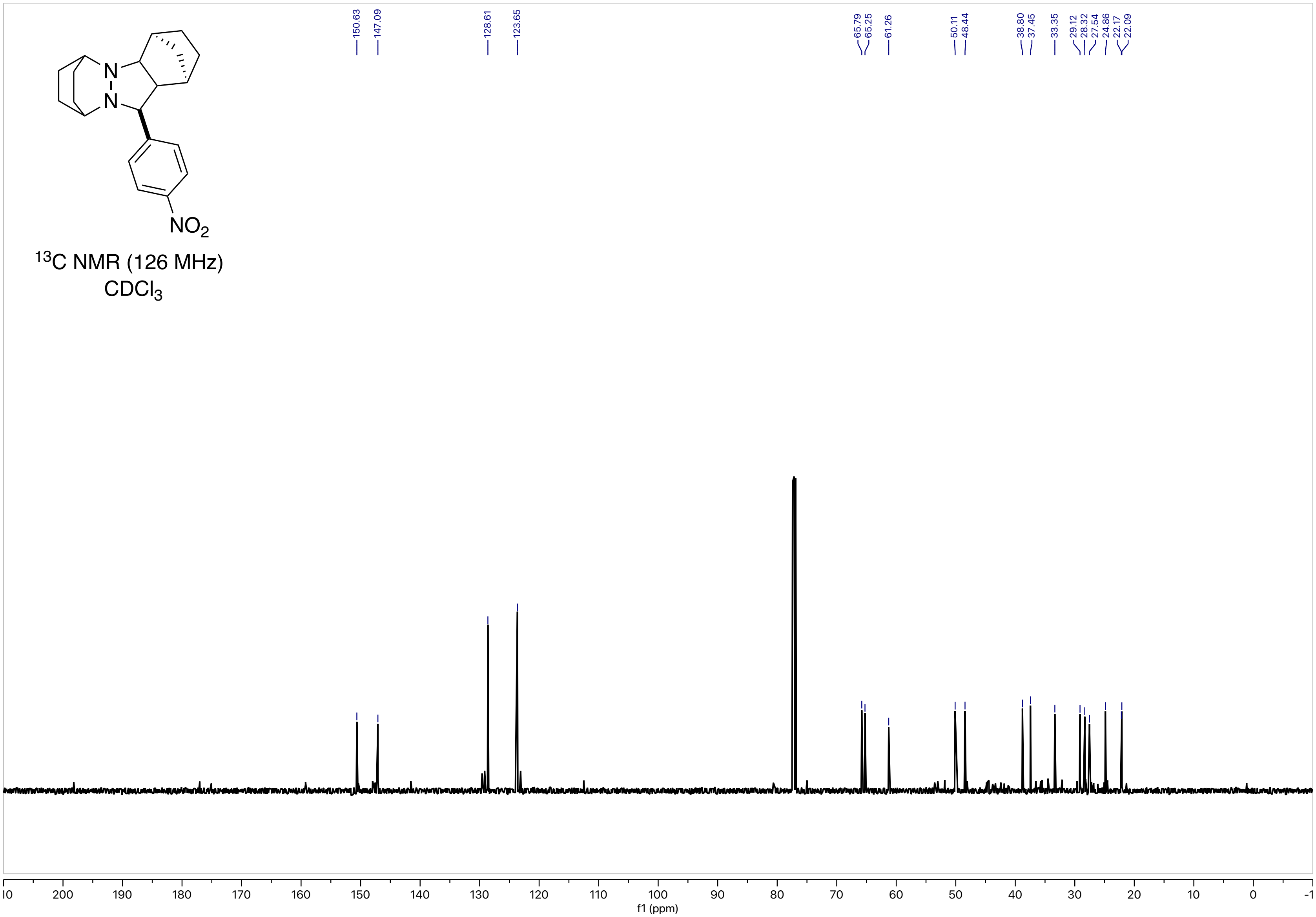
NO2
13C NMR (126 MHz)
CDCl3
150.63
147.09
128.61
123.65
65.79
65.25
61.26
50.11
48.44
38.80
37.45
33.35
29.12
28.32
27.54
24.86
22.17
22.09
I0
200
190
180
170
160
150
140
130
120
110
100
90
80
70
60
50
40
30
20
10
0
-1
f1 (ppm)

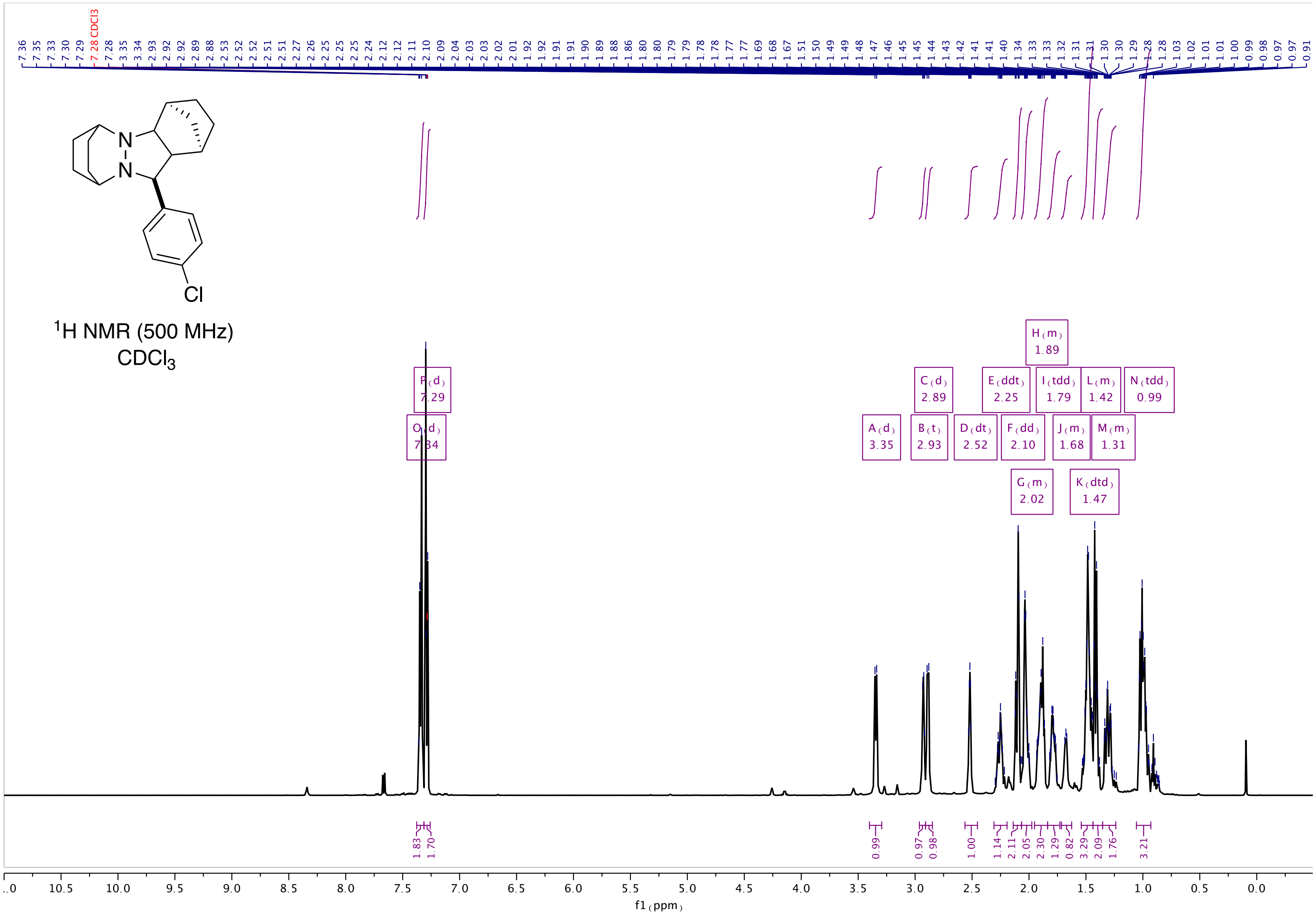

Cl
^{1}H NMR (500 MHz)
CDCl3
f1 (ppm)

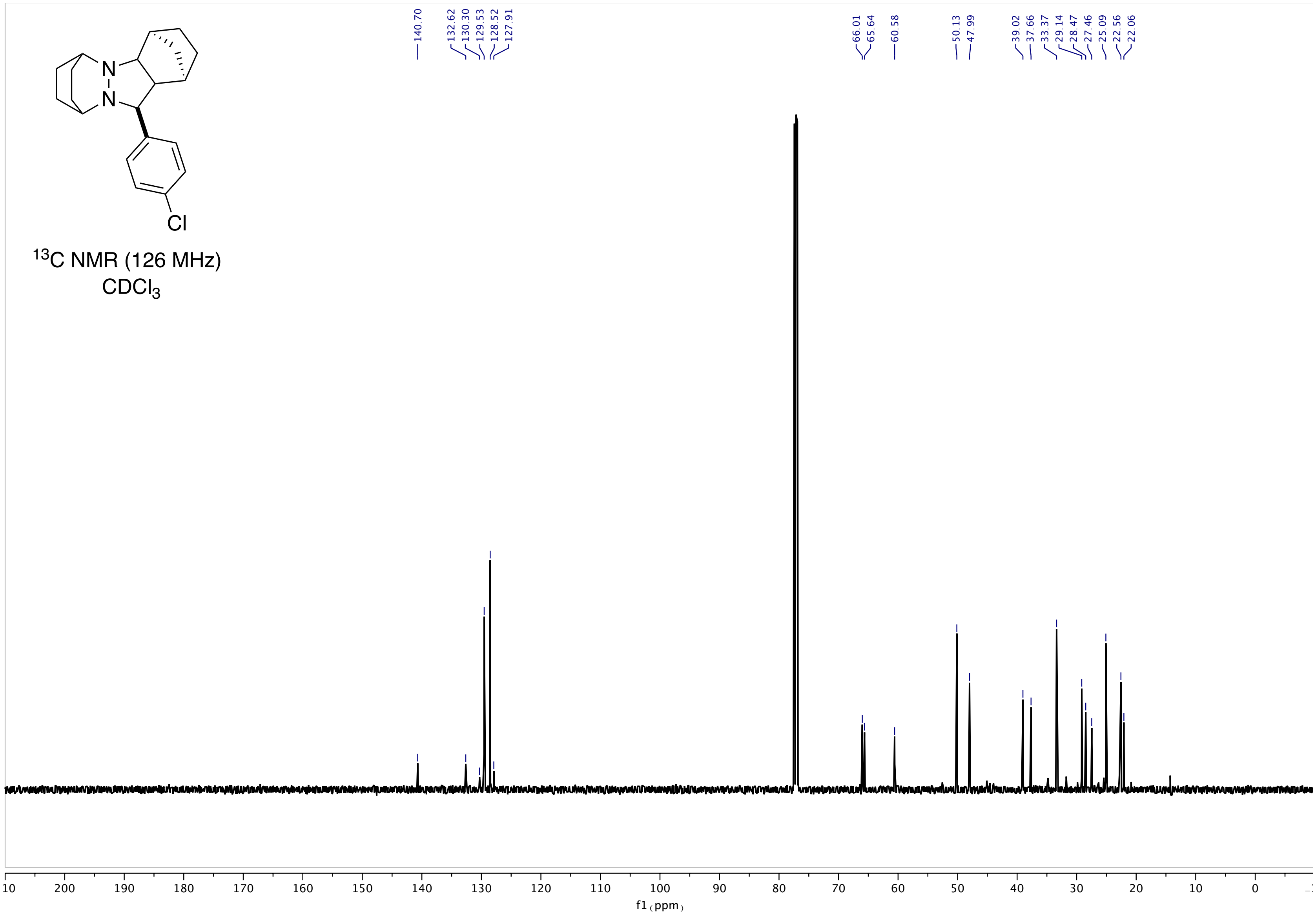

N
N
Cl
^{13}C NMR (126 MHz)
$CDCl_3$
140.70
132.62
130.30
129.53
128.52
127.91
66.01
65.64
60.58
50.13
47.99
39.02
37.66
33.37
29.14
28.47
27.46
25.09
22.56
22.06
f1 (ppm)

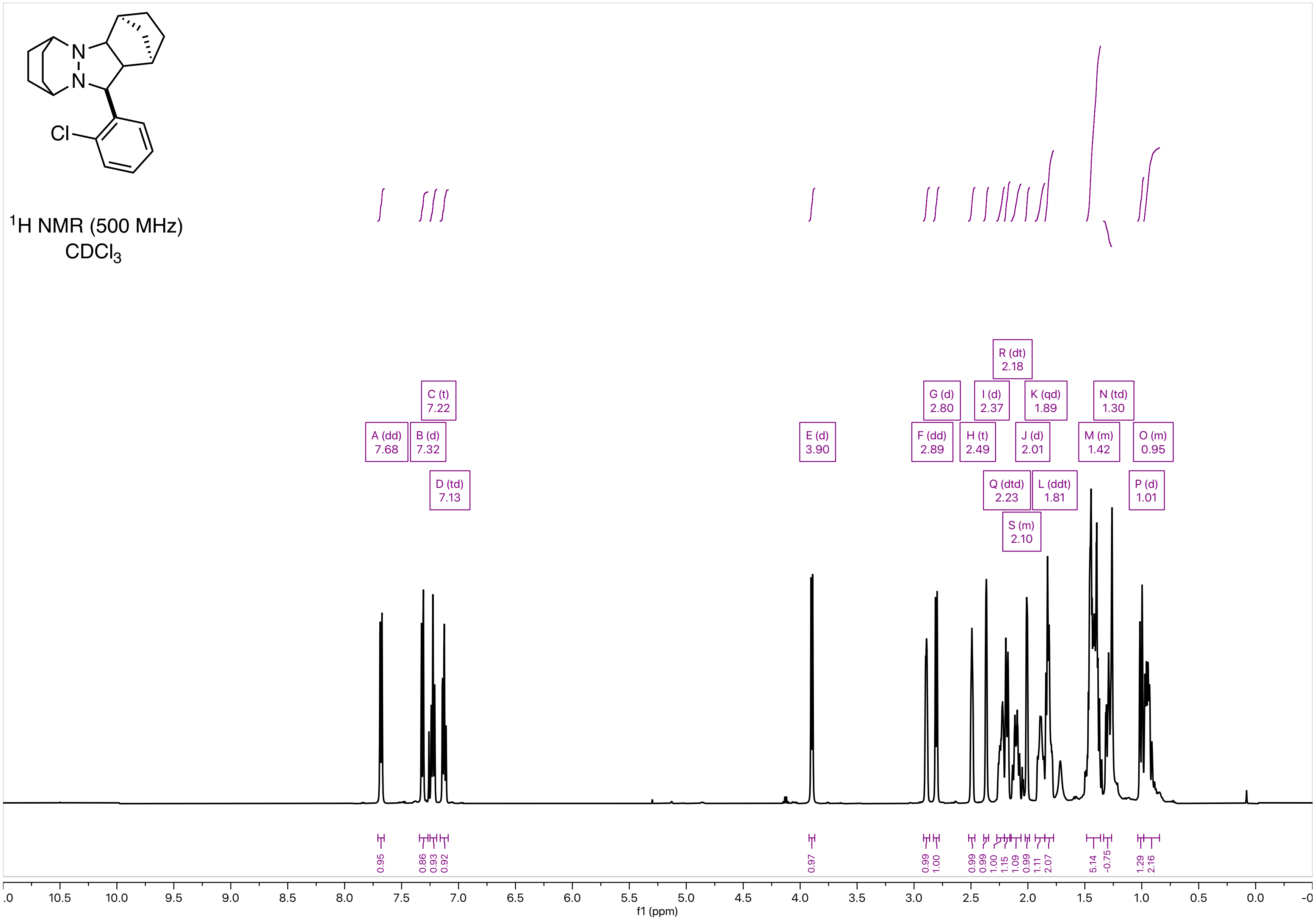

Cl
N
N
1H NMR (500 MHz)
CDCl3
A (dd) 7.68
B (d) 7.32
C (t) 7.22
D (td) 7.13
E (d) 3.90
F (dd) 2.89
G (d) 2.80
H (t) 2.49
I (d) 2.37
Q (dtd) 2.23
R (dt) 2.18
S (m) 2.10
J (d) 2.01
K (qd) 1.89
L (ddt) 1.81
M (m) 1.42
N (td) 1.30
P (d) 1.01
O (m) 0.95
0.95
0.86
0.93
0.92
0.97
0.99
1.00
0.99
0.99
1.00
1.15
1.09
0.99
1.11
2.07
5.14
-0.75
1.29
2.16
f1 (ppm)

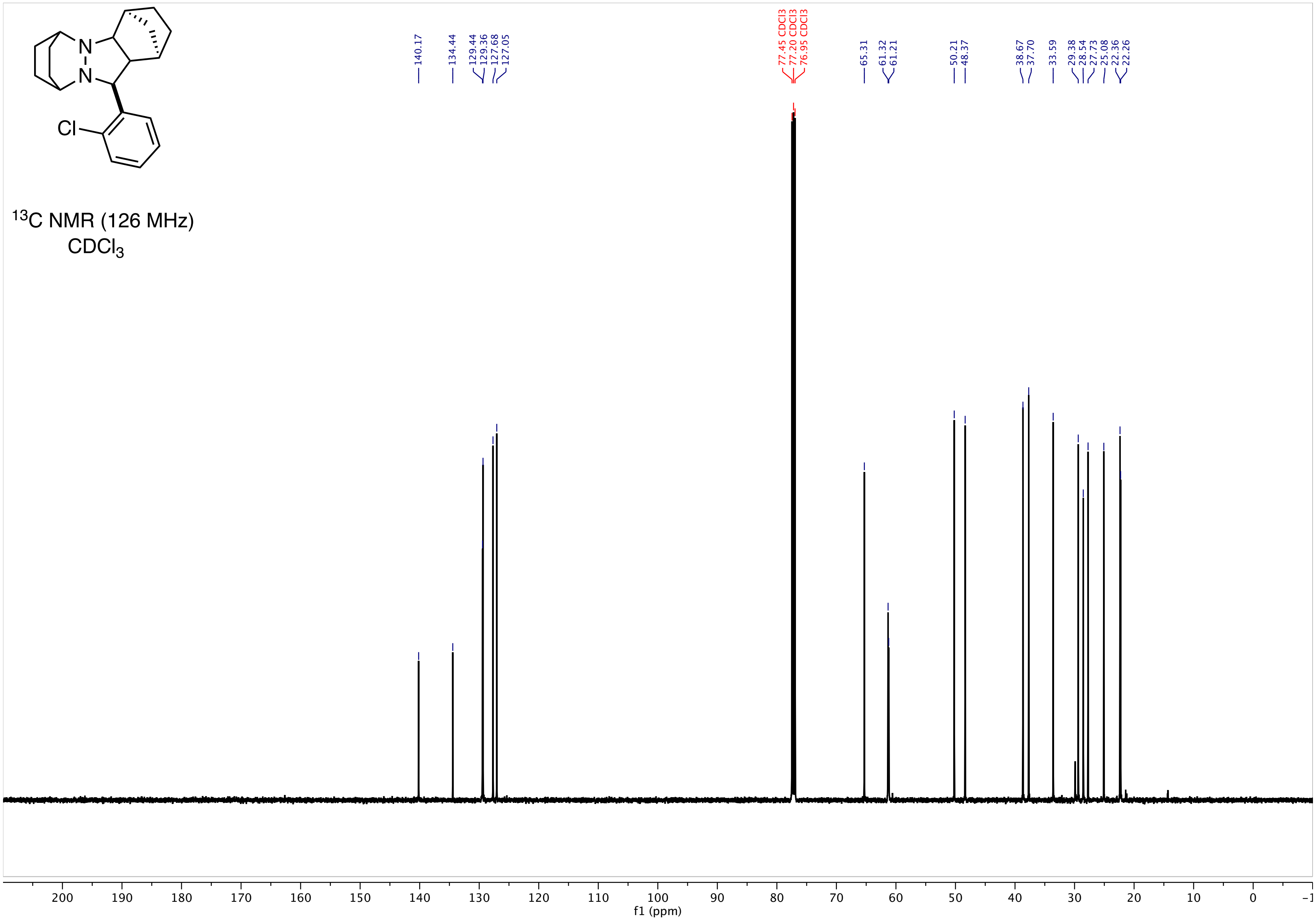

Cl
N
N
^{13}C NMR (126 MHz)
$CDCl_3$
140.17
134.44
129.44
129.36
127.68
127.05
77.45 CDCl3
77.20 CDCl3
76.95 CDCl3
65.31
61.32
61.21
50.21
48.37
38.67
37.70
33.59
29.38
28.54
27.73
25.08
22.36
22.26
f1 (ppm)

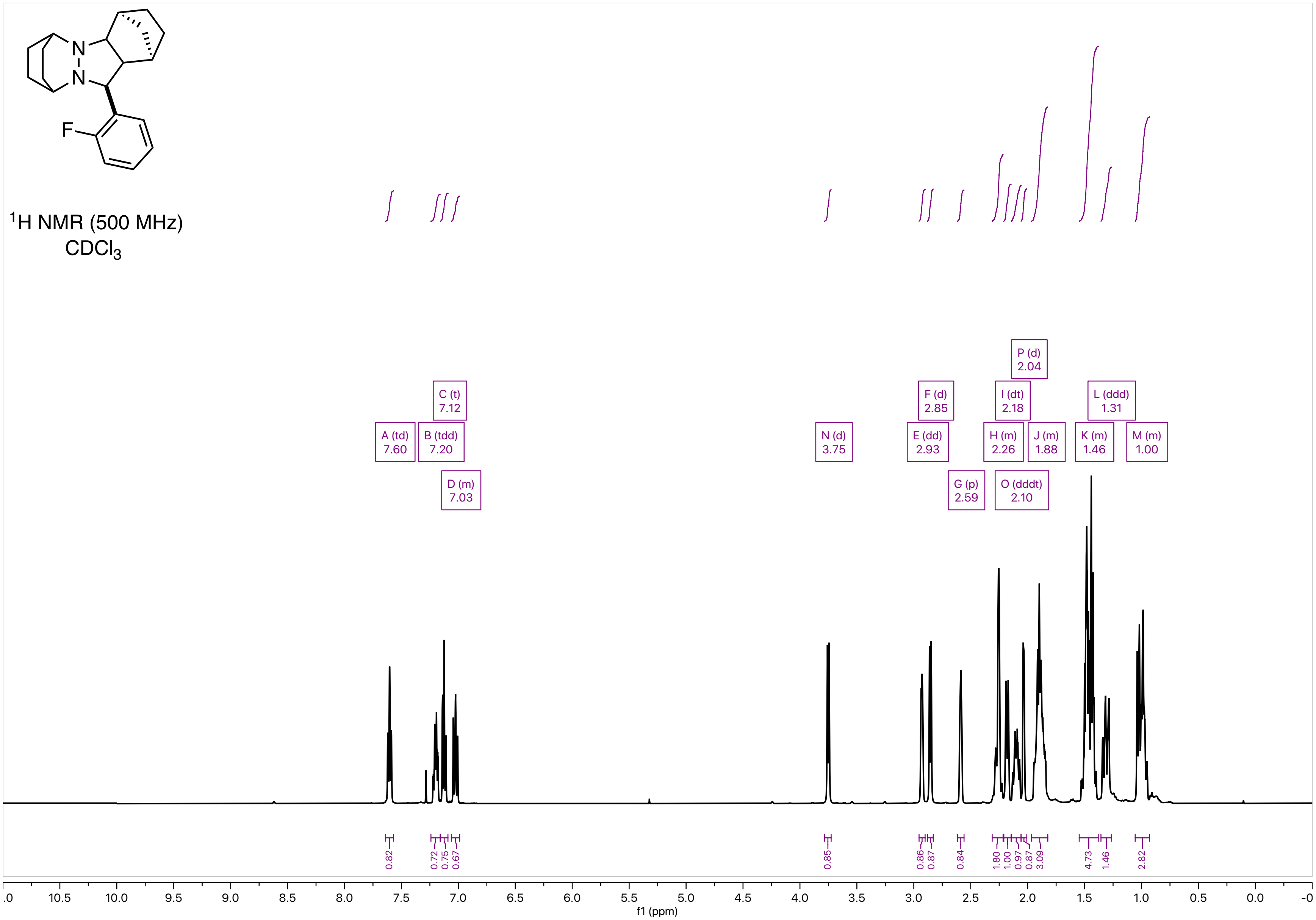
F
N
N
^{1}H NMR (500 MHz)
$CDCl_3$
A (td) 7.60
B (tdd) 7.20
C (t) 7.12
D (m) 7.03
N (d) 3.75
E (dd) 2.93
F (d) 2.85
G (p) 2.59
H (m) 2.26
I (dt) 2.18
O (dddt) 2.10
P (d) 2.04
J (m) 1.88
K (m) 1.46
L (ddd) 1.31
M (m) 1.00
0.82
0.72
0.75
0.67
0.85
0.86
0.87
0.84
1.80
1.00
0.97
0.87
3.09
4.73
1.46
2.82
f1 (ppm)

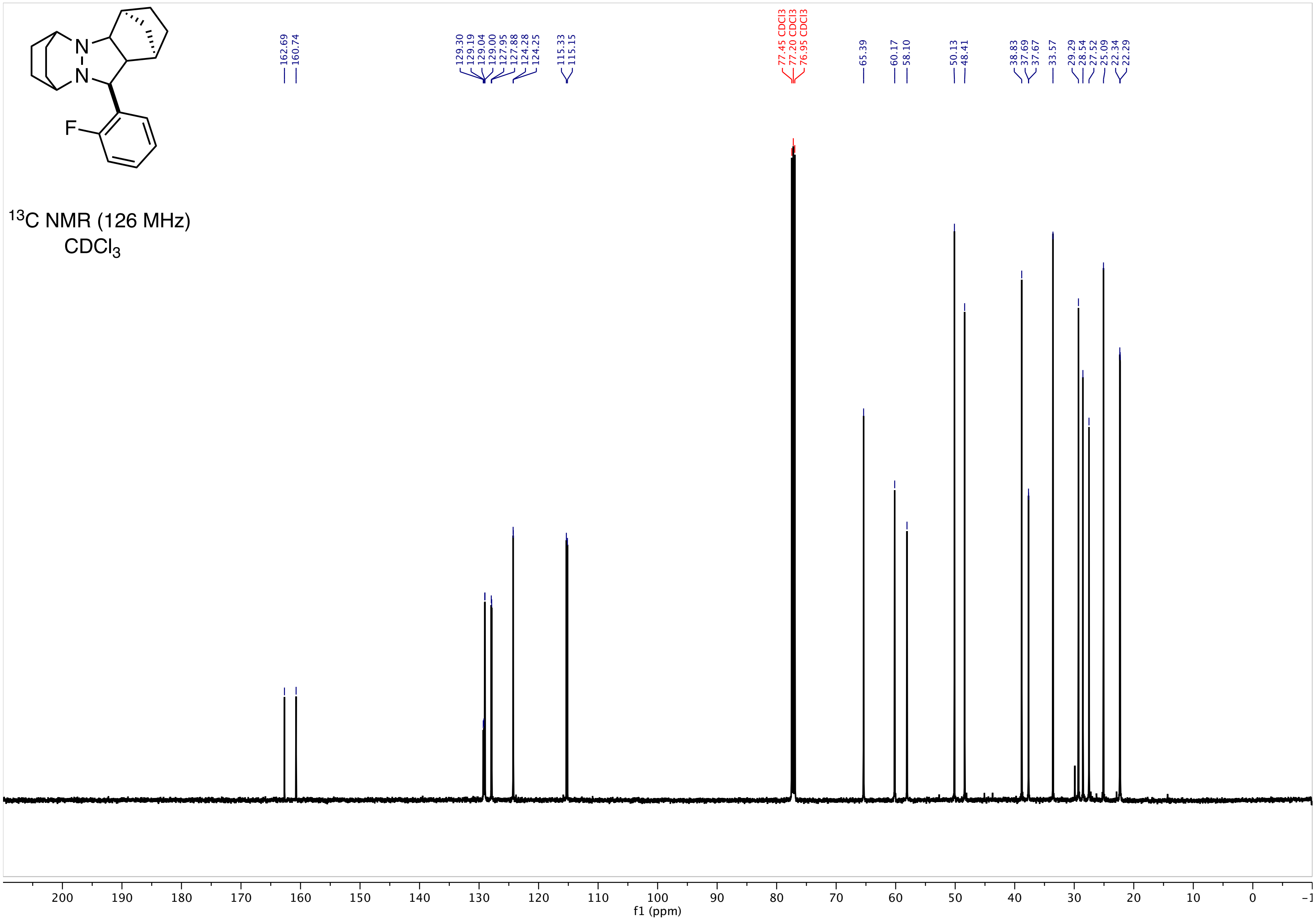

^{13}C NMR (126 MHz)
CDCl$_3$
162.69
160.74
129.30
129.19
129.04
129.00
127.95
127.88
124.28
124.25
115.33
115.15
77.45 CDCl3
77.20 CDCl3
76.95 CDCl3
65.39
60.17
58.10
50.13
48.41
38.83
37.69
37.67
33.57
29.29
28.54
27.52
25.09
22.34
22.29
f1 (ppm)

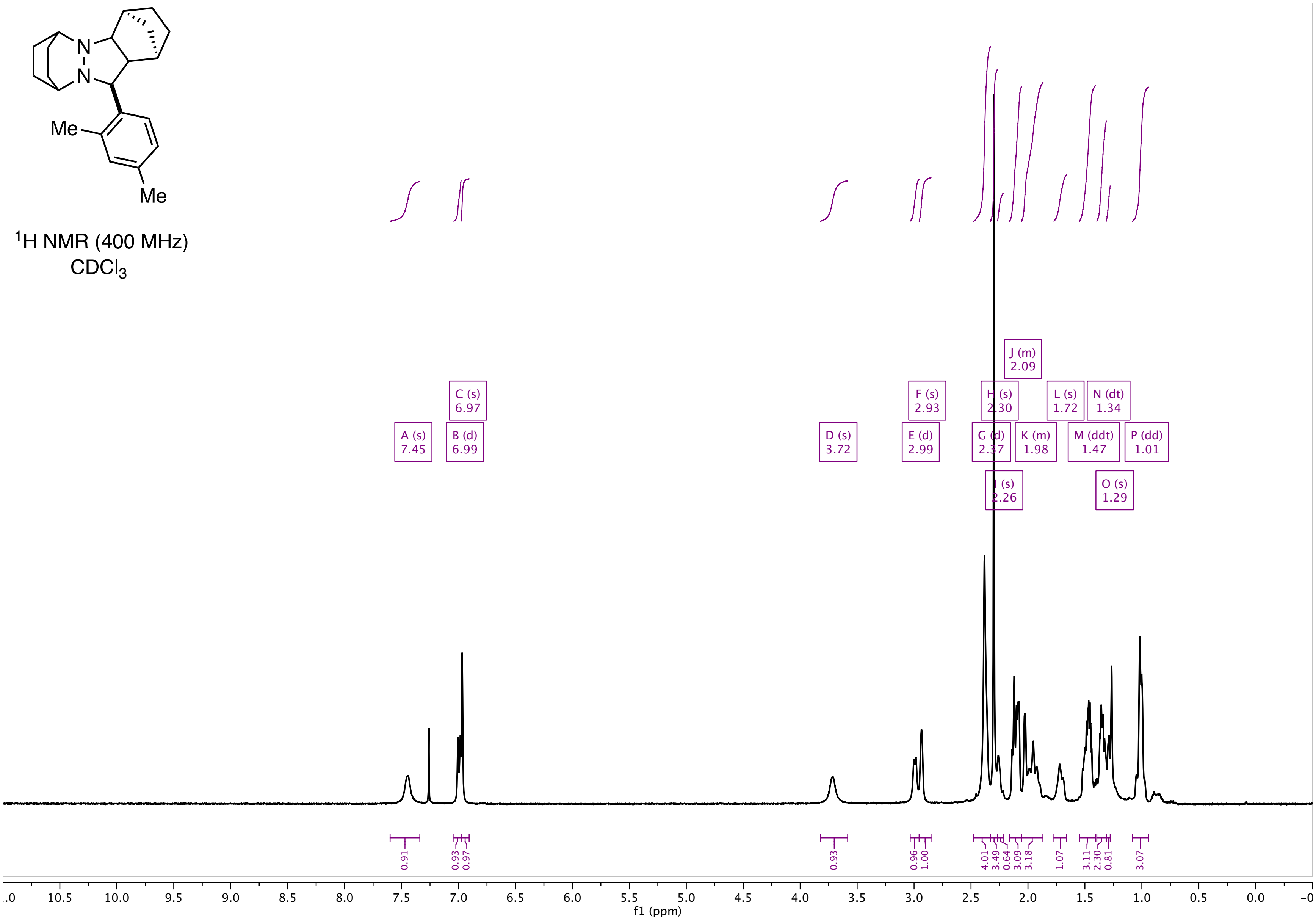

Me
Me
N
N
^{1}H NMR (400 MHz)
$CDCl_3$
A (s) 7.45
B (d) 6.99
C (s) 6.97
D (s) 3.72
E (d) 2.99
F (s) 2.93
G (d) 2.37
H (s) 2.30
I (s) 2.26
J (m) 2.09
K (m) 1.98
L (s) 1.72
M (ddt) 1.47
N (dt) 1.34
O (s) 1.29
P (dd) 1.01
0.91
0.93
0.97
0.93
0.96
1.00
4.01
3.49
0.64
3.09
3.18
1.07
3.11
2.30
0.81
3.07
f1 (ppm)

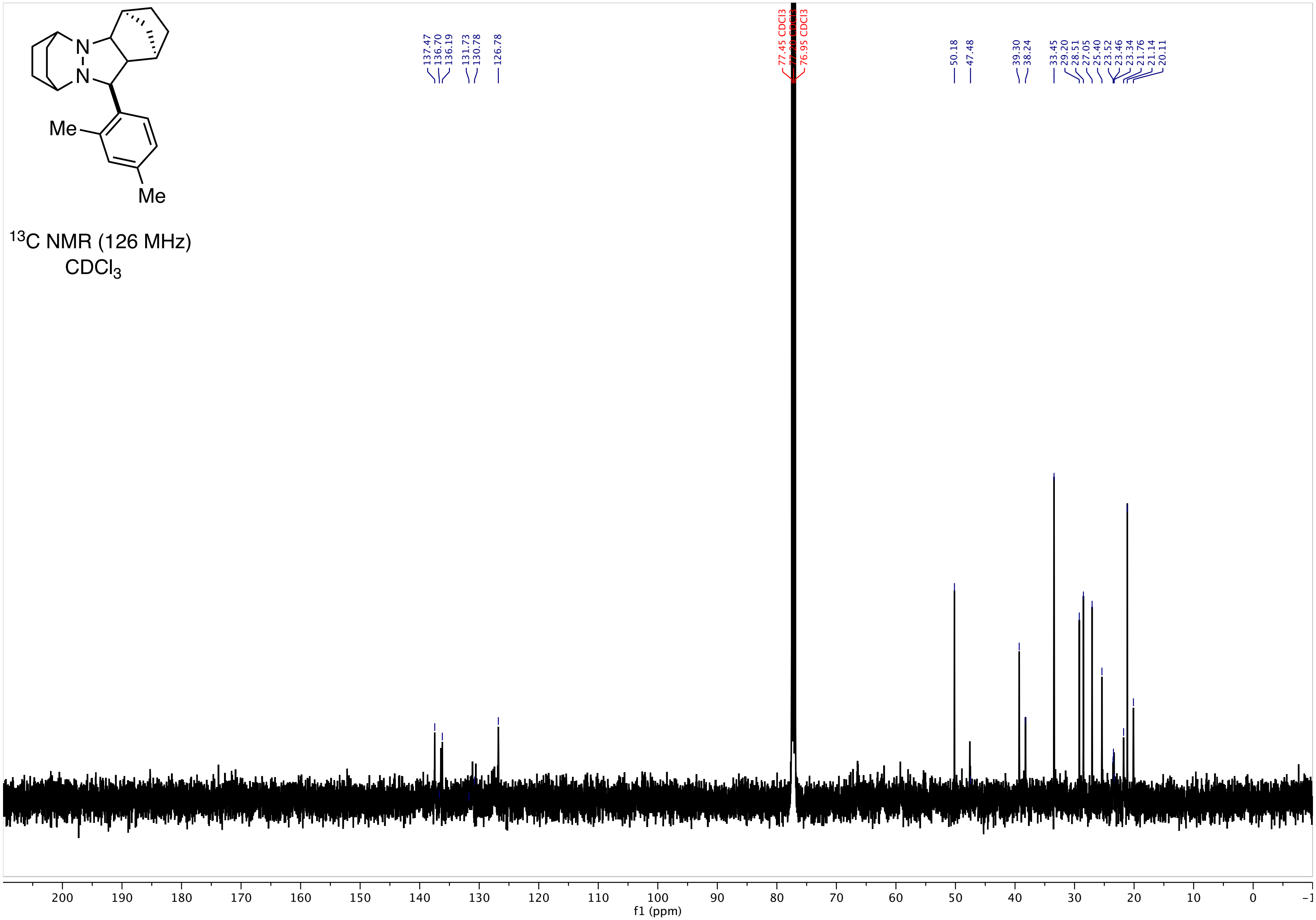

Me
Me
¹³C NMR (126 MHz)
CDCl3
137.47
136.70
136.19
131.73
130.78
126.78
77.45 CDCl3
77.20 CDCl3
76.95 CDCl3
50.18
47.48
39.30
38.24
33.45
29.20
28.51
27.05
25.40
23.52
23.46
23.34
21.76
21.14
20.11
f1 (ppm)

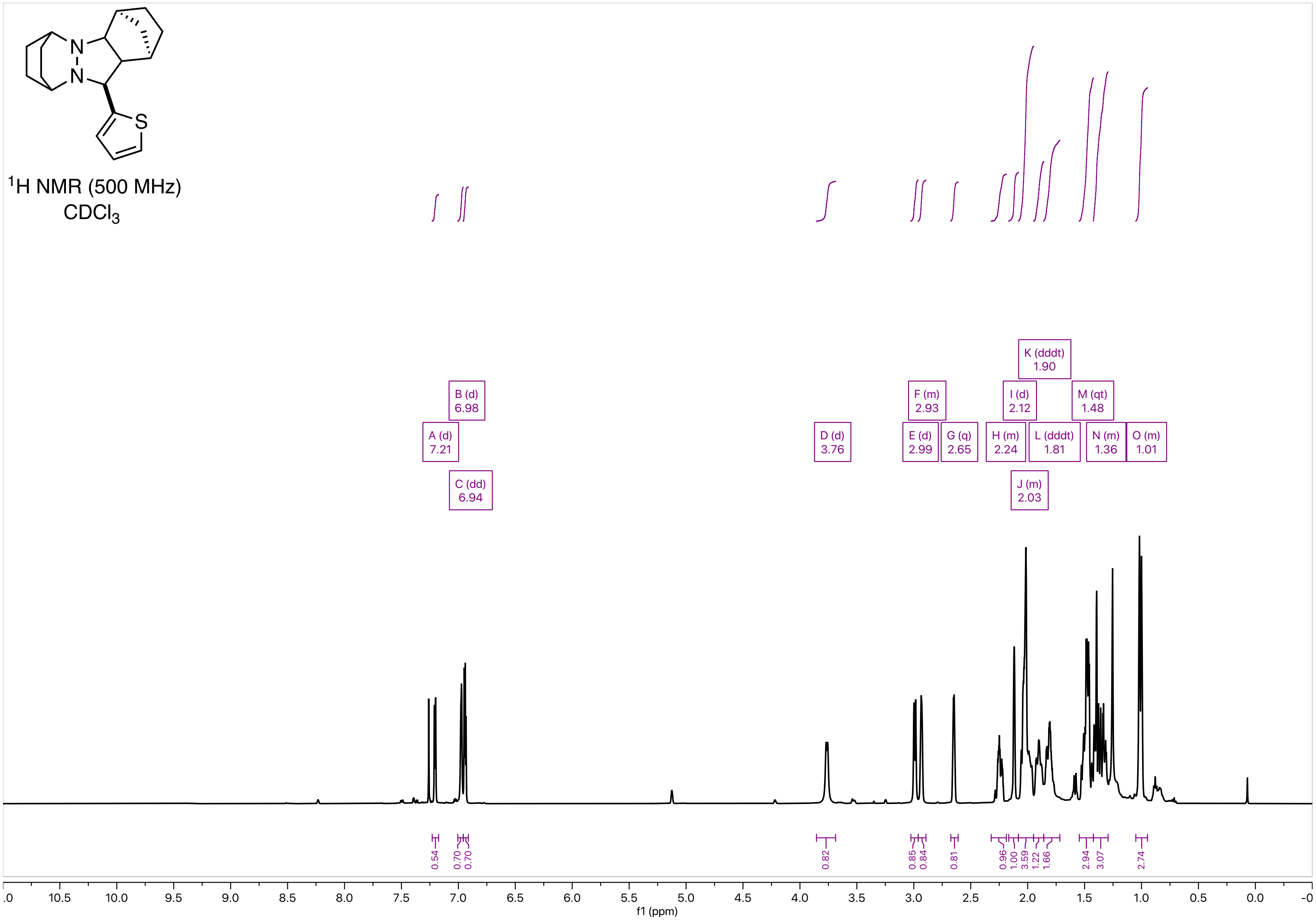

N
N
S
1H NMR (500 MHz)
CDCl3
A (d) 7.21
B (d) 6.98
C (dd) 6.94
D (d) 3.76
E (d) 2.99
F (m) 2.93
G (q) 2.65
H (m) 2.24
I (d) 2.12
J (m) 2.03
K (dddt) 1.90
L (dddt) 1.81
M (qt) 1.48
N (m) 1.36
O (m) 1.01
0.54
0.70
0.70
0.82
0.85
0.84
0.81
0.96
1.00
3.59
1.22
1.66
2.94
3.07
2.74
f1 (ppm)

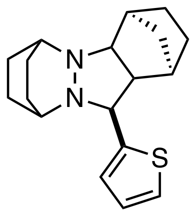

^{13}C NMR (126 MHz)
$CDCl_3$

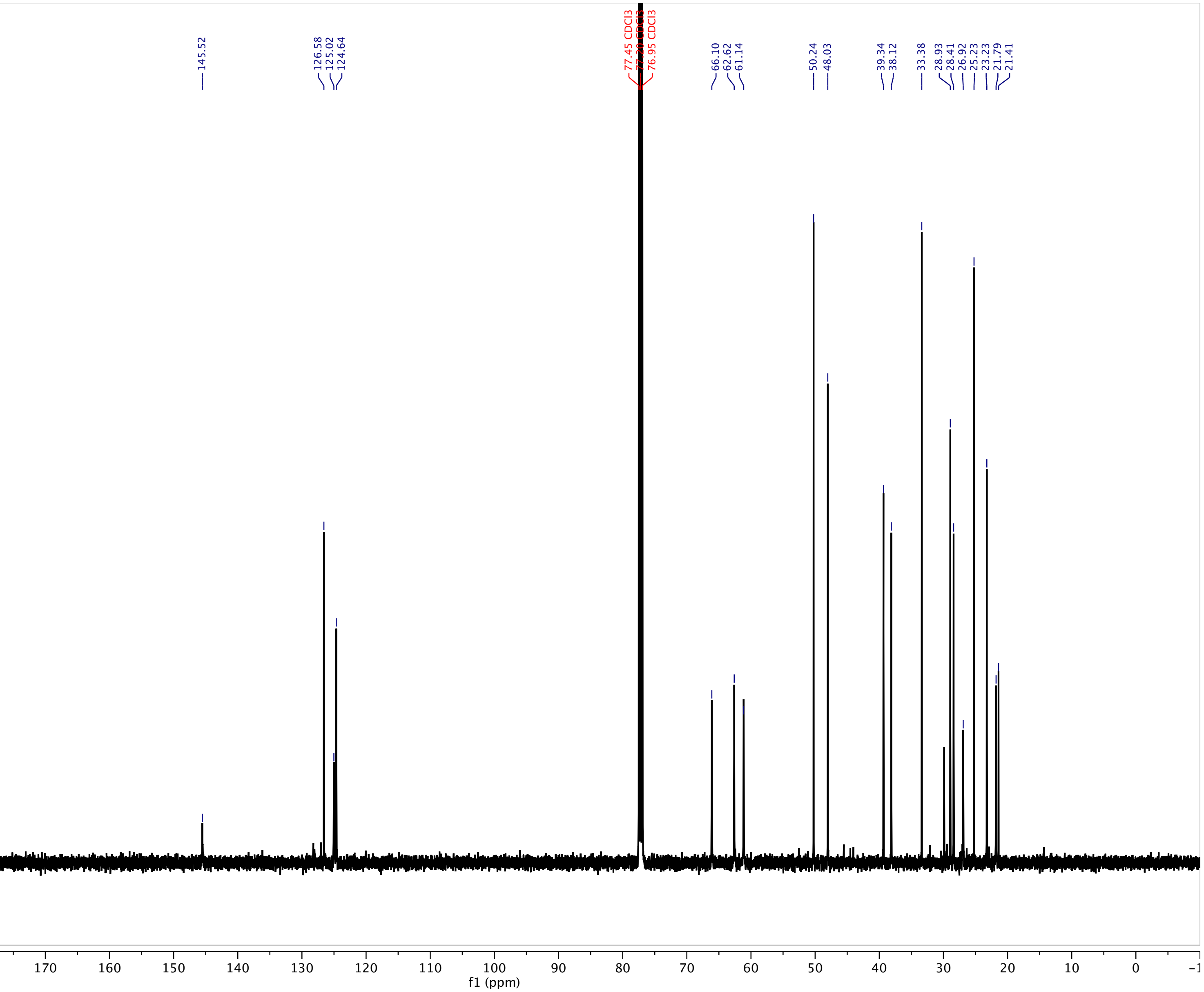

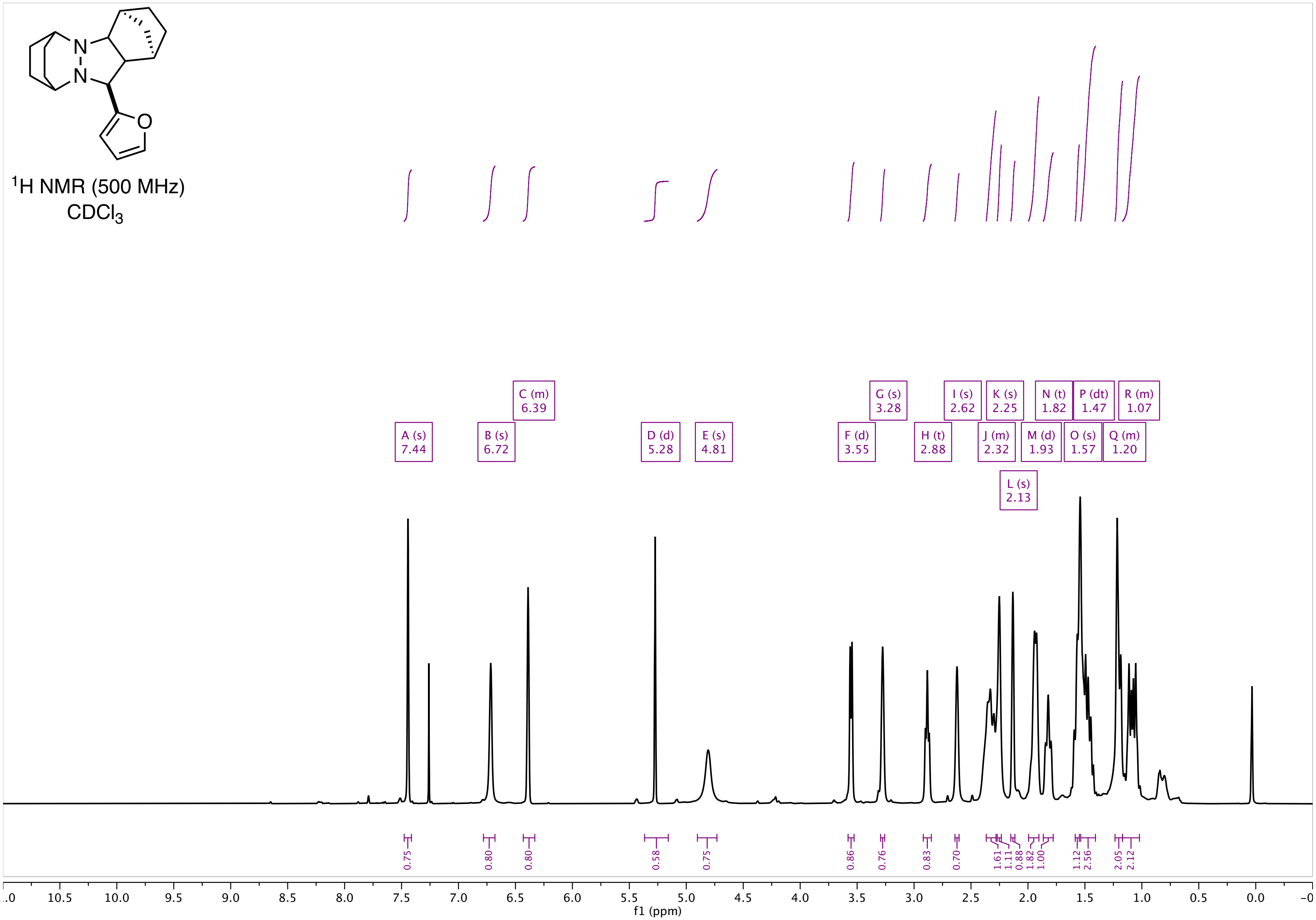

^{1}H NMR (500 MHz)
$CDCl_3$
A (s) 7.44
B (s) 6.72
C (m) 6.39
D (d) 5.28
E (s) 4.81
F (d) 3.55
G (s) 3.28
H (t) 2.88
I (s) 2.62
J (m) 2.32
K (s) 2.25
L (s) 2.13
M (d) 1.93
N (t) 1.82
O (s) 1.57
P (dt) 1.47
Q (m) 1.20
R (m) 1.07
0.75
0.80
0.80
0.58
0.75
0.86
0.76
0.83
0.70
1.61
1.11
0.88
1.82
1.00
1.12
2.56
2.05
2.12
f1 (ppm)

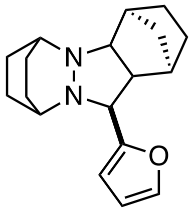

^{13}C NMR (126 MHz)
$CDCl_3$

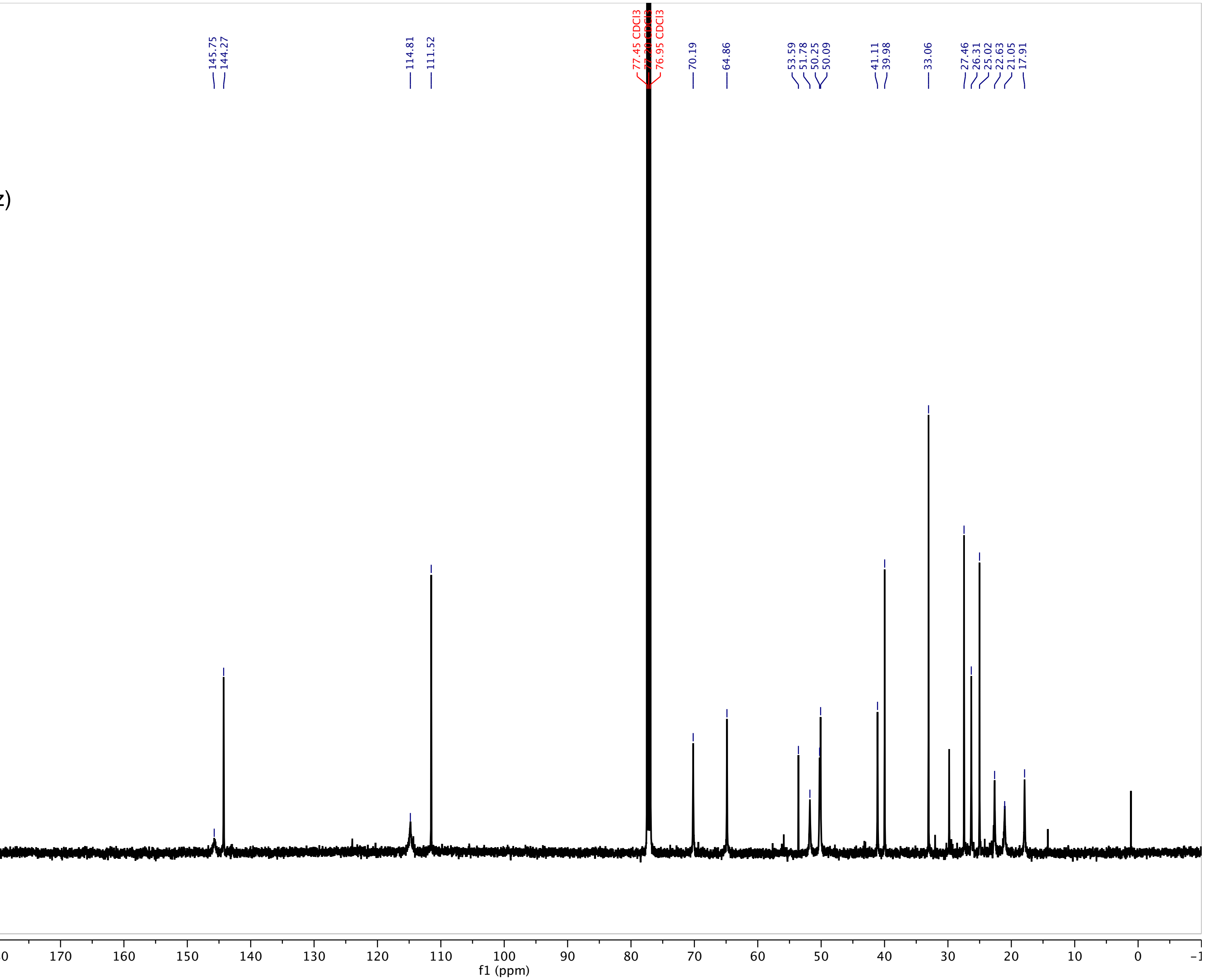

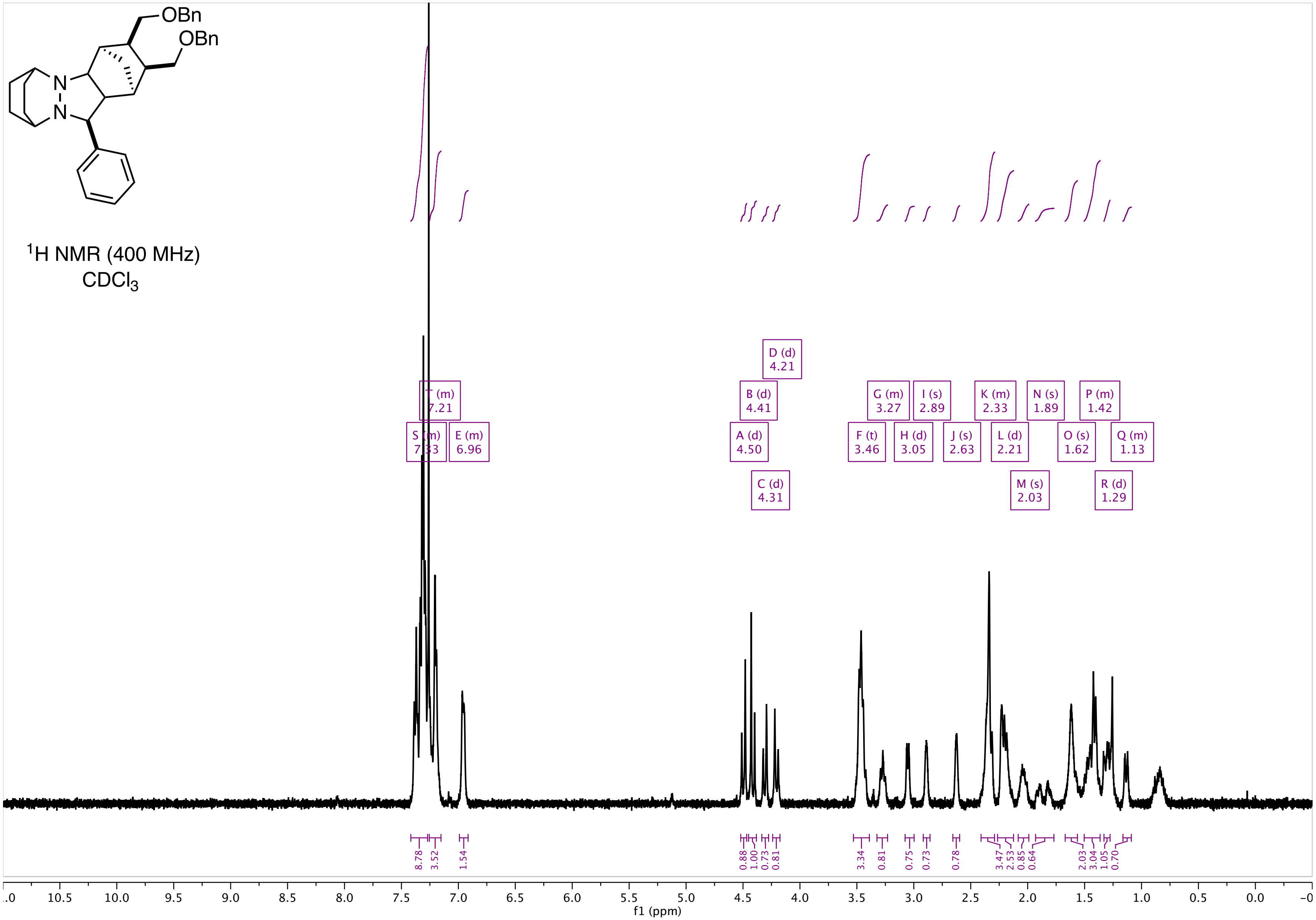
OBn
OBn
N
N
^{1}H NMR (400 MHz)
$CDCl_3$
S (m) 7.33
T (m) 7.21
E (m) 6.96
A (d) 4.50
B (d) 4.41
C (d) 4.31
D (d) 4.21
F (t) 3.46
G (m) 3.27
H (d) 3.05
I (s) 2.89
J (s) 2.63
K (m) 2.33
L (d) 2.21
M (s) 2.03
N (s) 1.89
O (s) 1.62
P (m) 1.42
R (d) 1.29
Q (m) 1.13
8.78
3.52
1.54
0.88
1.00
0.73
0.81
3.34
0.81
0.75
0.73
0.78
3.47
2.53
0.85
0.64
2.03
3.04
1.05
0.70
f1 (ppm)

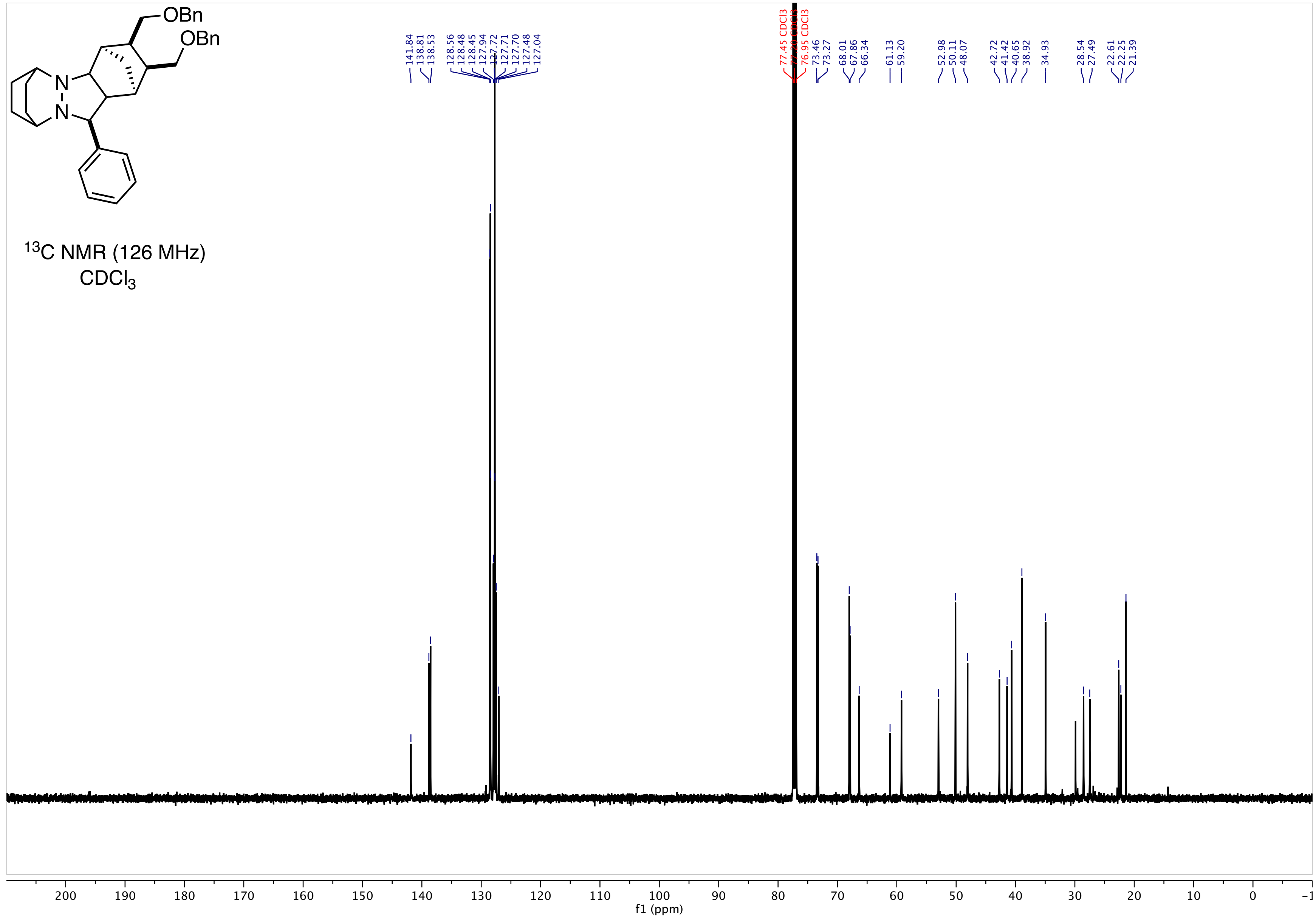
OBn
OBn
N
N
^{13}C NMR (126 MHz)
CDCl$_3$
141.84
138.81
138.53
128.56
128.48
128.45
127.94
127.72
127.71
127.70
127.48
127.04
77.45 CDCl3
77.20 CDCl3
76.95 CDCl3
73.46
73.27
68.01
67.86
66.34
61.13
59.20
52.98
50.11
48.07
42.72
41.42
40.65
38.92
34.93
28.54
27.49
22.61
22.25
21.39
f1 (ppm)

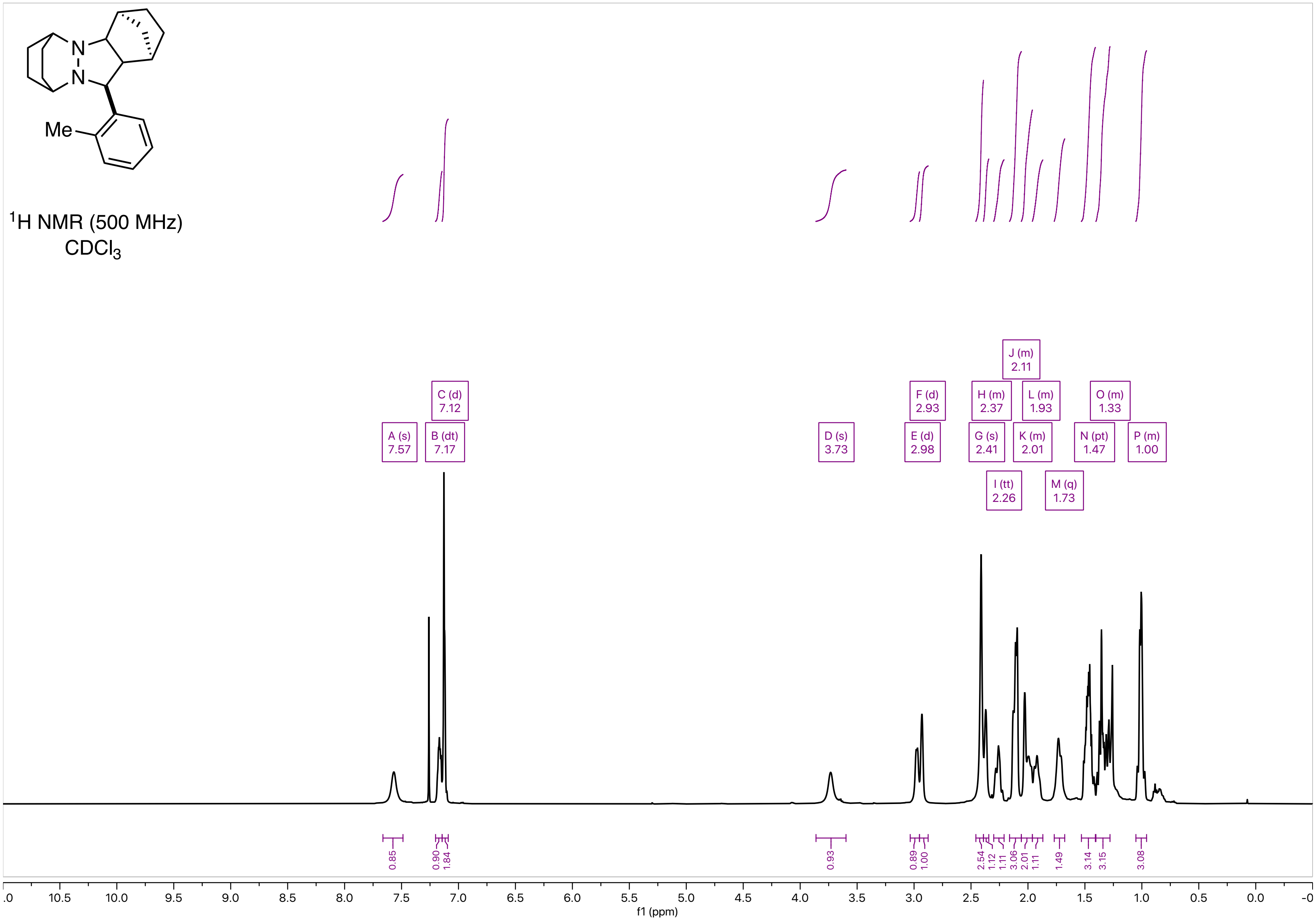

Me
N
N
^{1}H NMR (500 MHz)
$CDCl_3$
A (s) 7.57
B (dt) 7.17
C (d) 7.12
D (s) 3.73
E (d) 2.98
F (d) 2.93
G (s) 2.41
H (m) 2.37
I (tt) 2.26
J (m) 2.11
K (m) 2.01
L (m) 1.93
M (q) 1.73
N (pt) 1.47
O (m) 1.33
P (m) 1.00
0.85
0.90
1.84
0.93
0.89
1.00
2.54
1.12
1.11
3.06
2.01
1.11
1.49
3.14
3.15
3.08
f1 (ppm)

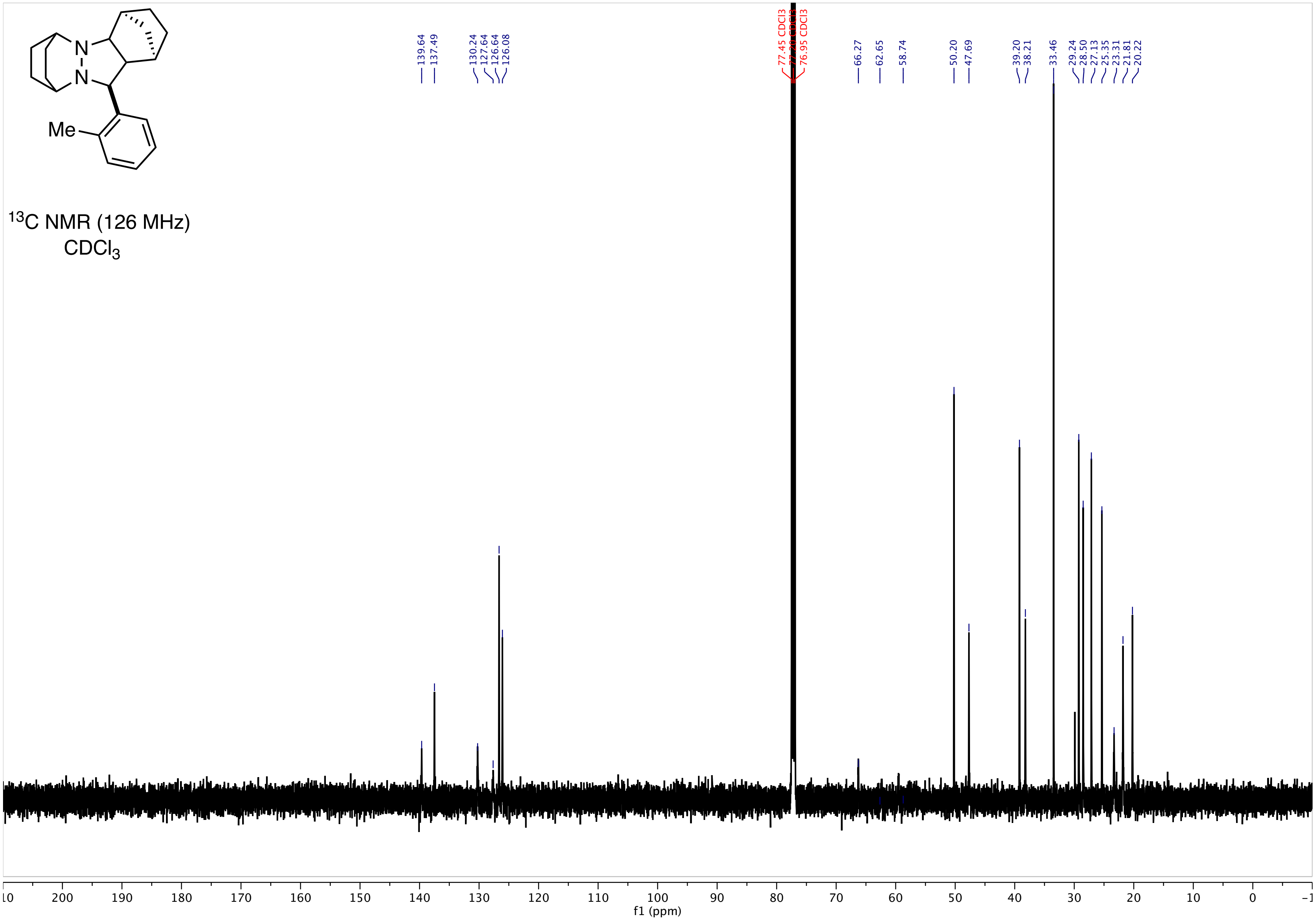

Me
N
N
^{13}C NMR (126 MHz)
$CDCl_3$
139.64
137.49
130.24
127.64
126.64
126.08
77.45 CDCl3
76.95 CDCl3
66.27
62.65
58.74
50.20
47.69
39.20
38.21
33.46
29.24
28.50
27.13
25.35
23.31
21.81
20.22
f1 (ppm)

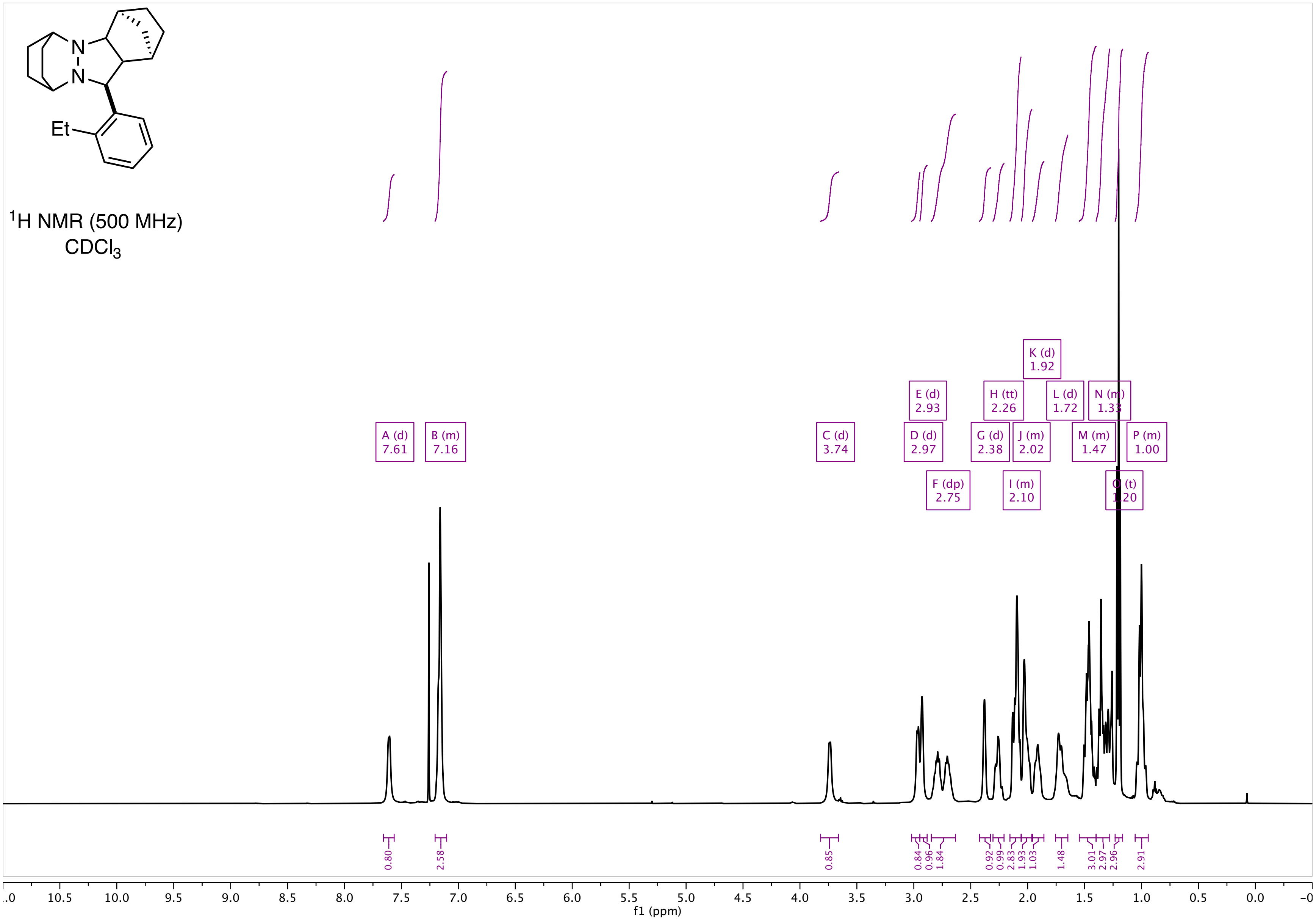

Et
N
N
1H NMR (500 MHz)
CDCl3
A (d) 7.61
B (m) 7.16
C (d) 3.74
D (d) 2.97
E (d) 2.93
F (dp) 2.75
G (d) 2.38
H (tt) 2.26
I (m) 2.10
J (m) 2.02
K (d) 1.92
L (d) 1.72
M (m) 1.47
N (m) 1.33
O (t) 1.20
P (m) 1.00
0.80
2.58
0.85
0.84
0.96
1.84
0.92
0.99
2.83
1.93
1.03
1.48
3.01
2.97
2.96
2.91
f1 (ppm)

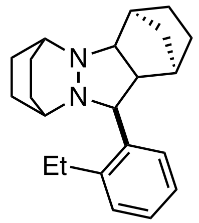

^{13}C NMR (126 MHz)
$CDCl_3$

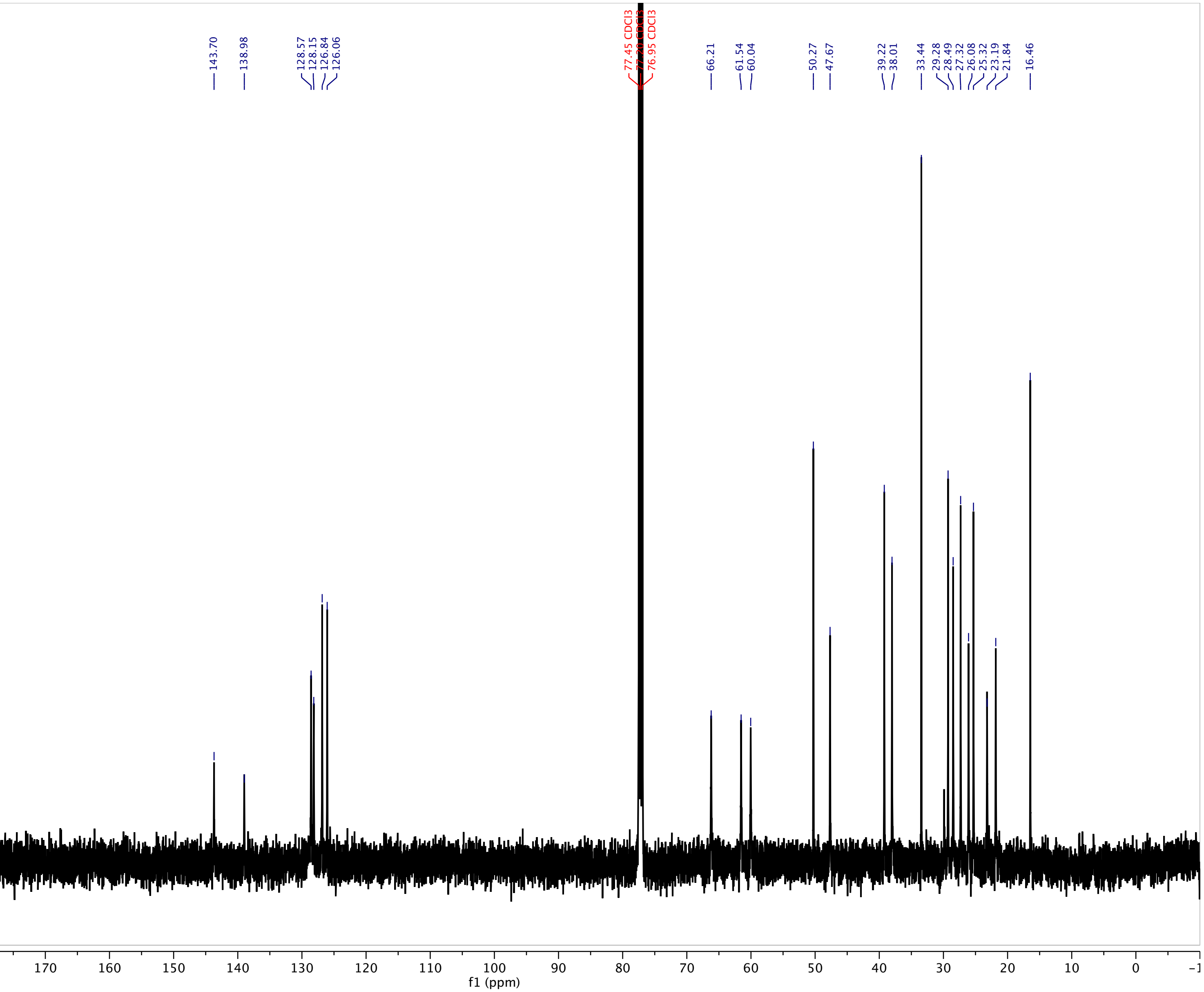

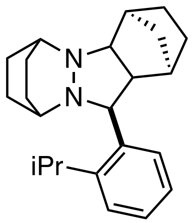

^{1}H NMR (500 MHz)
$CDCl_3$

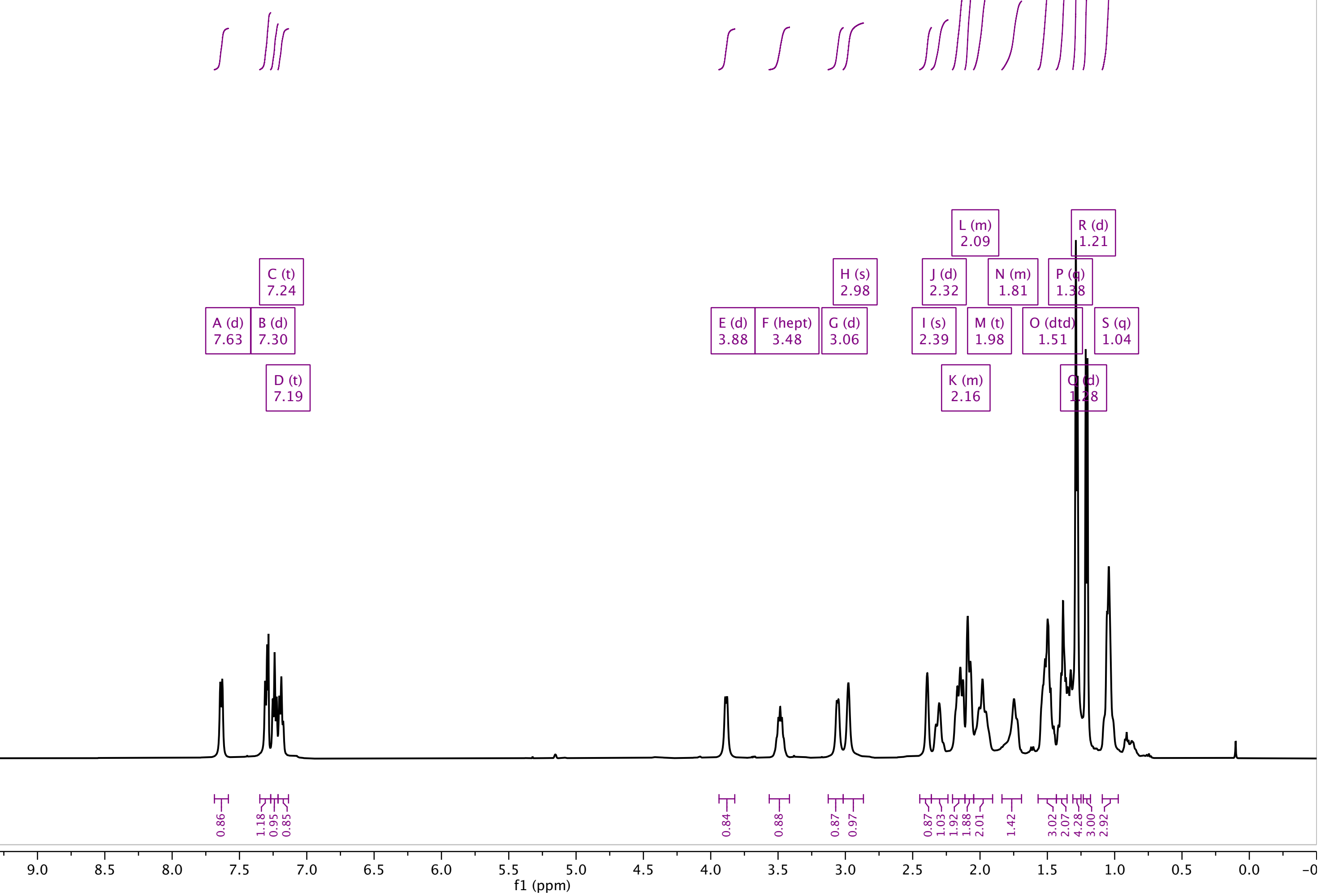

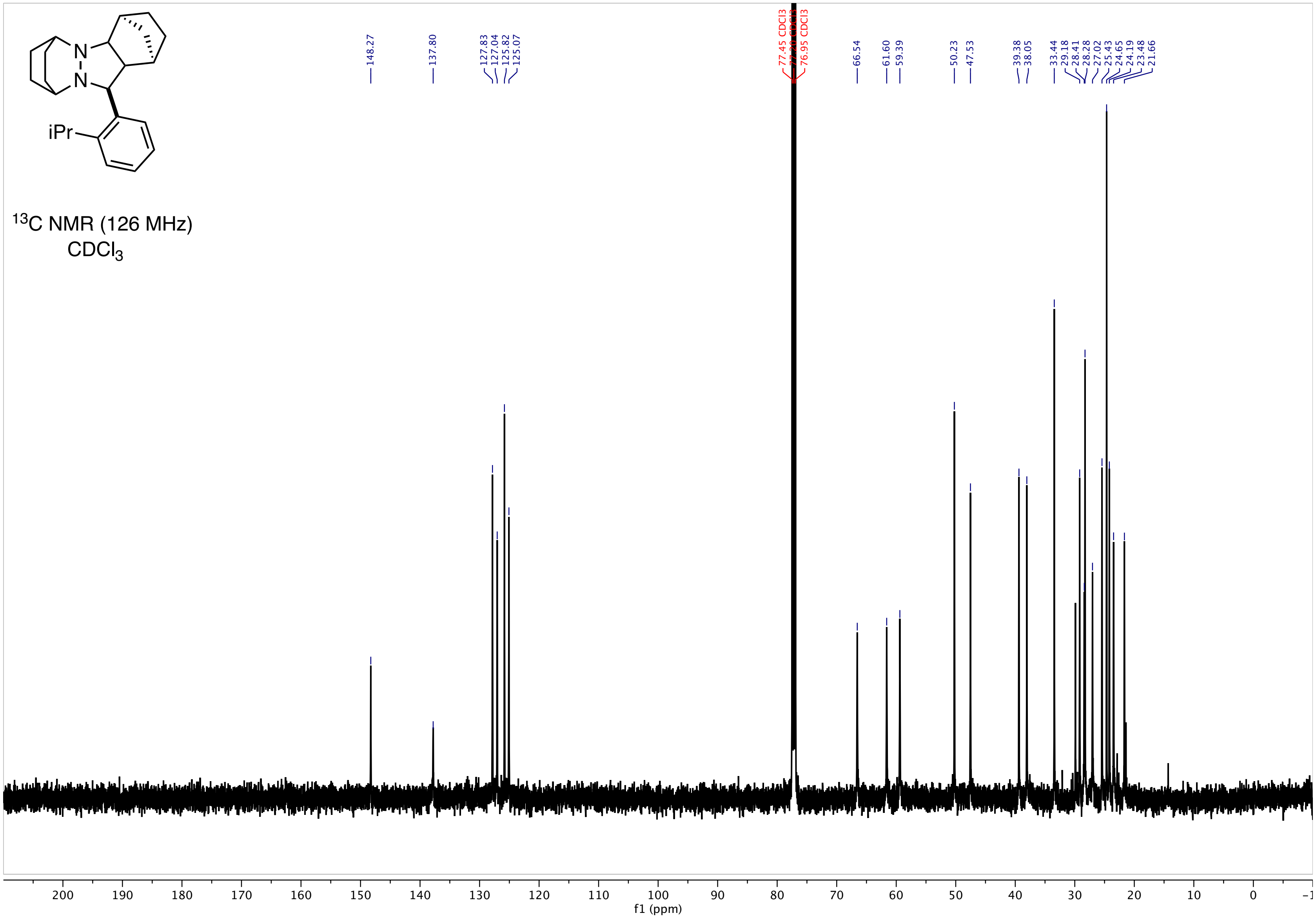

iPr
13C NMR (126 MHz)
CDCl3
148.27
137.80
127.83
127.04
125.82
125.07
77.45 CDCl3
76.95 CDCl3
66.54
61.60
59.39
50.23
47.53
39.38
38.05
33.44
29.18
28.41
28.28
27.02
25.43
24.65
24.19
23.48
21.66
f1 (ppm)

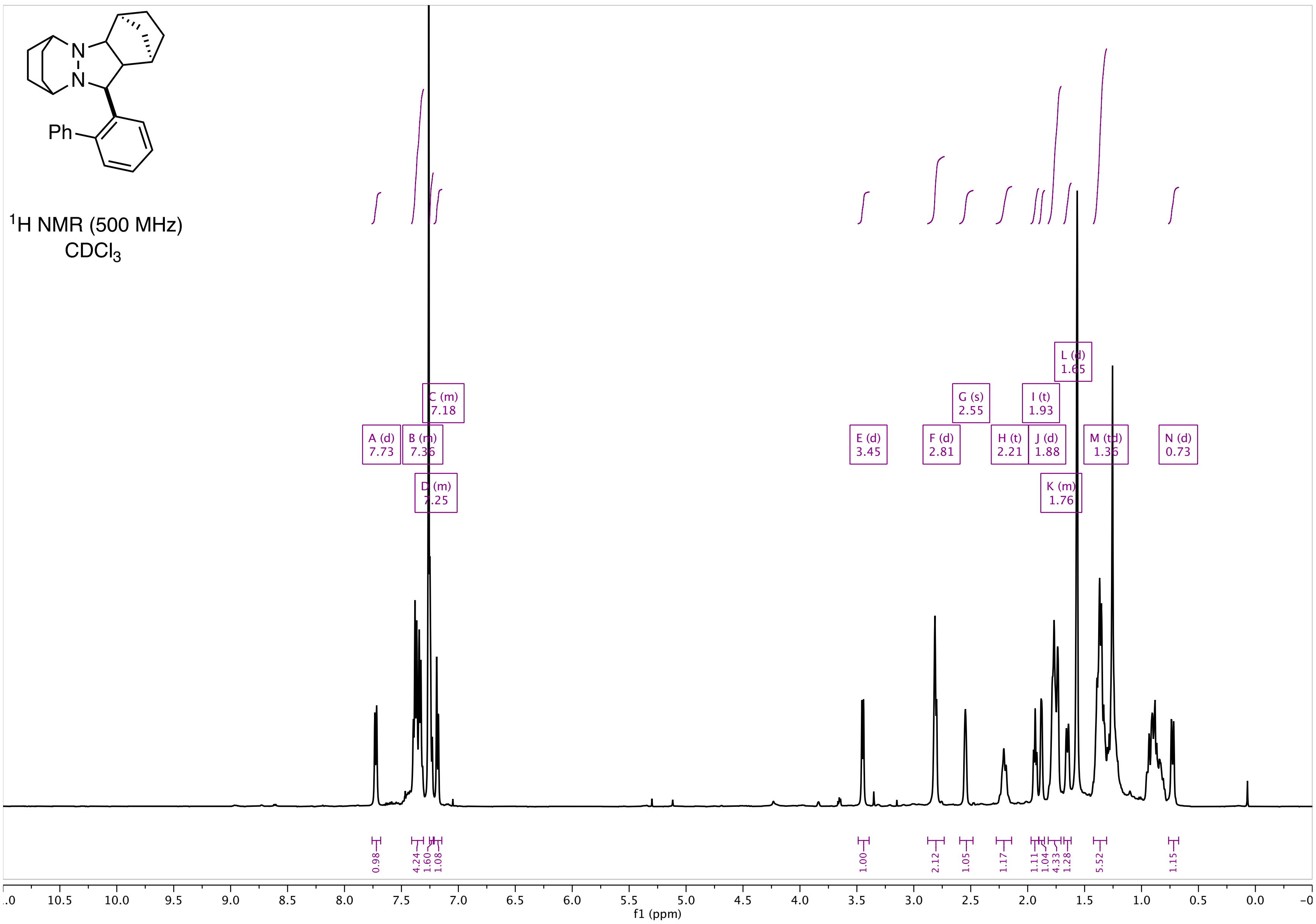

Ph
N
N
^{1}H NMR (500 MHz)
$CDCl_3$
A (d) 7.73
B (m) 7.36
C (m) 7.18
D (m) 7.25
E (d) 3.45
F (d) 2.81
G (s) 2.55
H (t) 2.21
I (t) 1.93
J (d) 1.88
K (m) 1.76
L (d) 1.65
M (td) 1.36
N (d) 0.73
0.98
4.24
1.60
1.08
1.00
2.12
1.05
1.17
1.11
1.04
4.33
1.28
5.52
1.15
f1 (ppm)

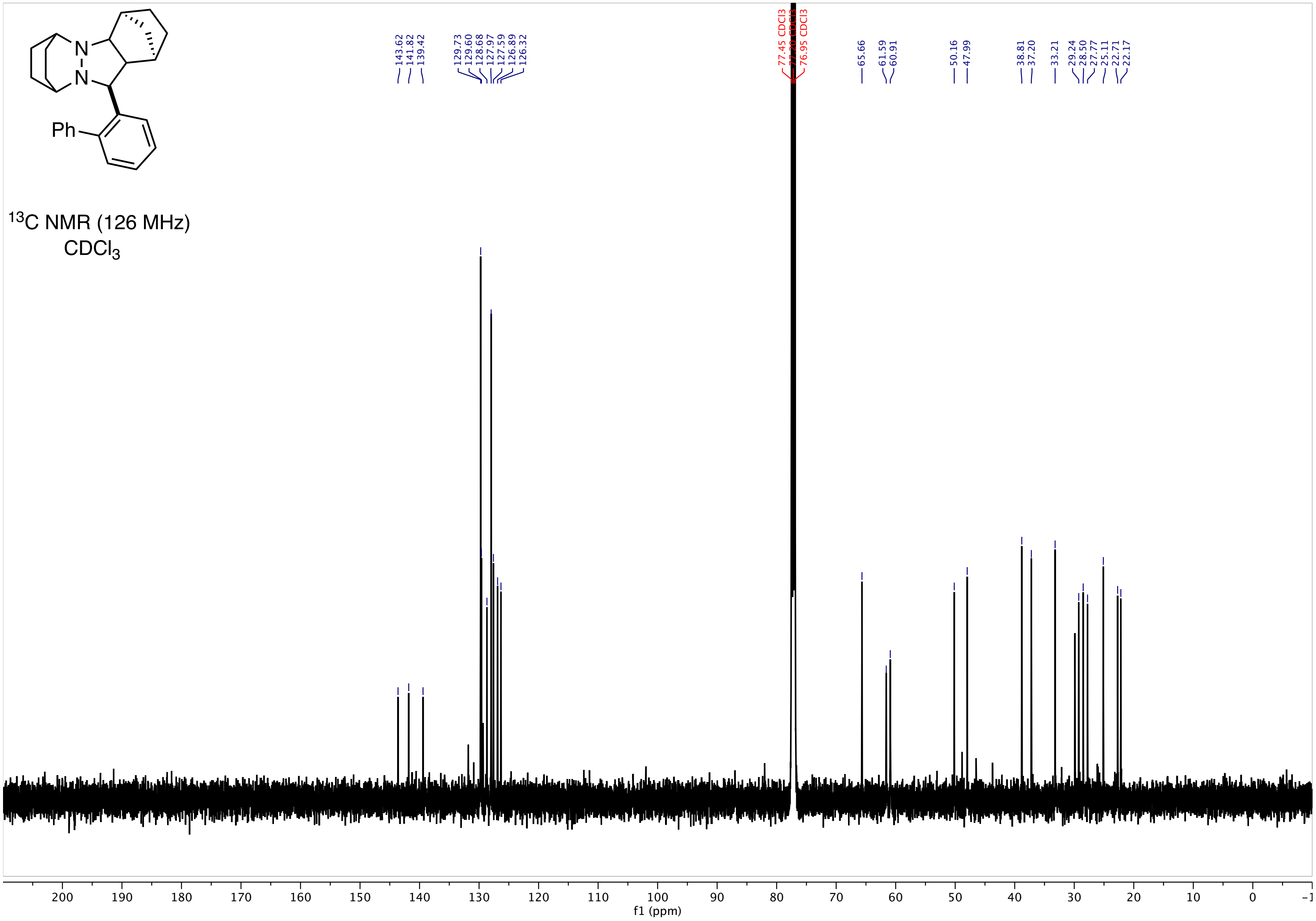
Ph
N
N
13C NMR (126 MHz)
CDCl3
143.62
141.82
139.42
129.73
129.60
128.68
127.97
127.59
126.89
126.32
77.45 CDCl3
77.20 CDCl3
76.95 CDCl3
65.66
61.59
60.91
50.16
47.99
38.81
37.20
33.21
29.24
28.50
27.77
25.11
22.71
22.17
200
190
180
170
160
150
140
130
120
110
100
90
80
70
60
50
40
30
20
10
0
f1 (ppm)

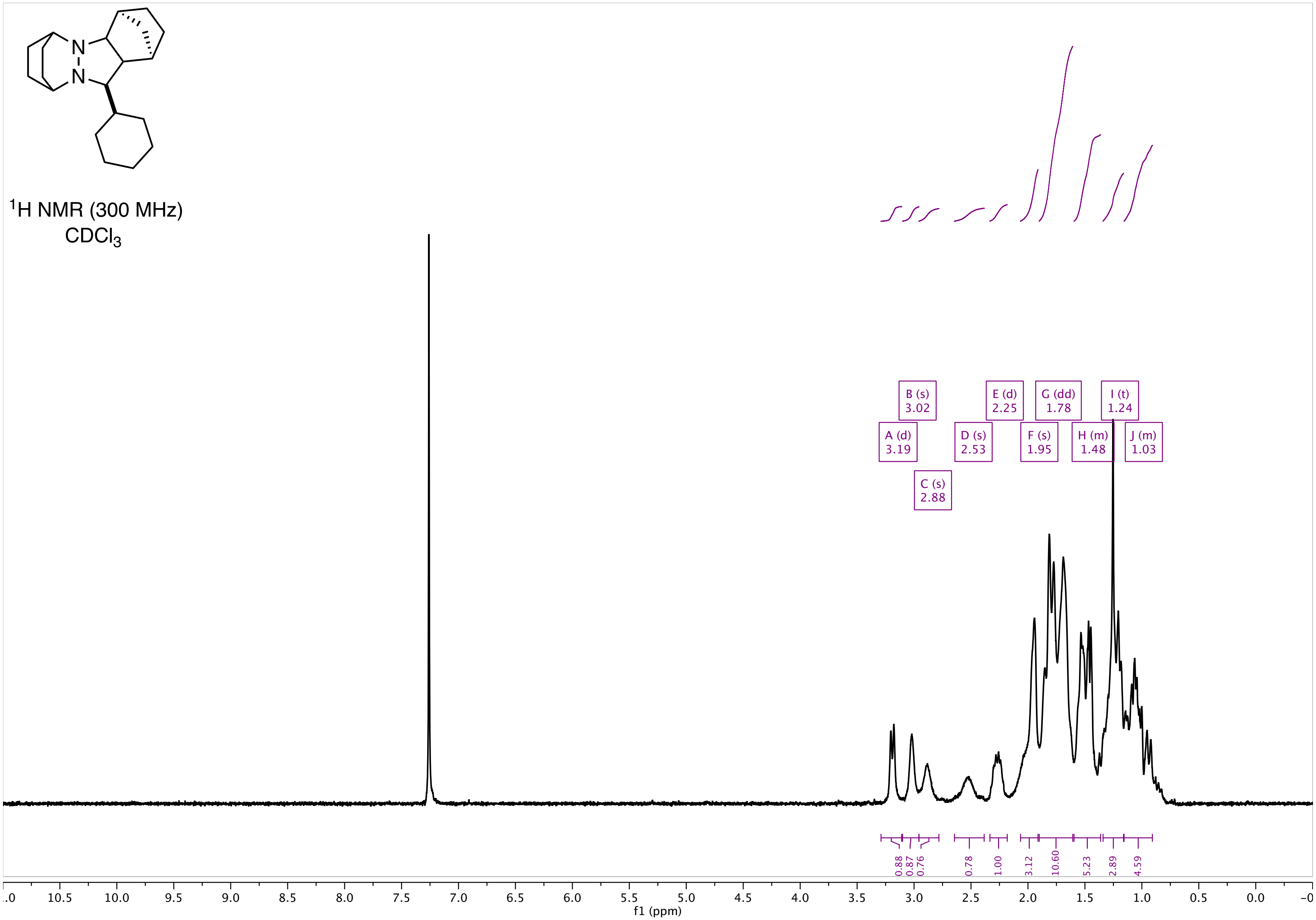
1H NMR (300 MHz)
CDCl3
A (d) 3.19
B (s) 3.02
C (s) 2.88
D (s) 2.53
E (d) 2.25
F (s) 1.95
G (dd) 1.78
H (m) 1.48
I (t) 1.24
J (m) 1.03
0.88
0.87
0.76
0.78
1.00
3.12
10.60
5.23
2.89
4.59
f1 (ppm)

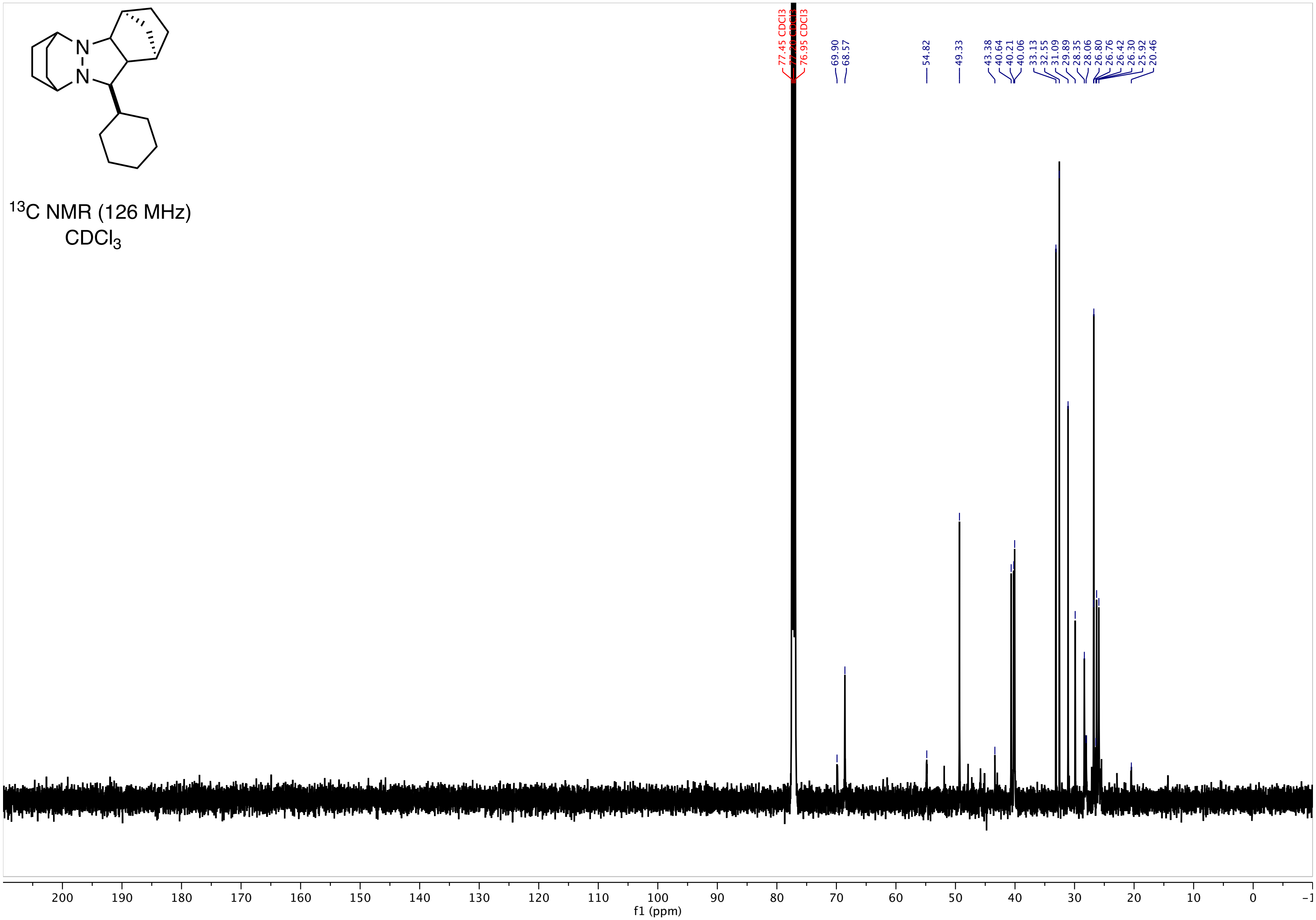

N
N
^{13}C NMR (126 MHz)
$CDCl_3$
77.45 CDCl3
[illegible]
76.95 CDCl3
69.90
68.57
54.82
49.33
43.38
40.64
40.21
40.06
33.13
32.55
31.09
29.89
28.35
28.06
26.80
26.76
26.42
26.30
25.92
20.46
200 190 180 170 160 150 140 130 120 110 100 90 80 70 60 50 40 30 20 10 0
f1 (ppm)

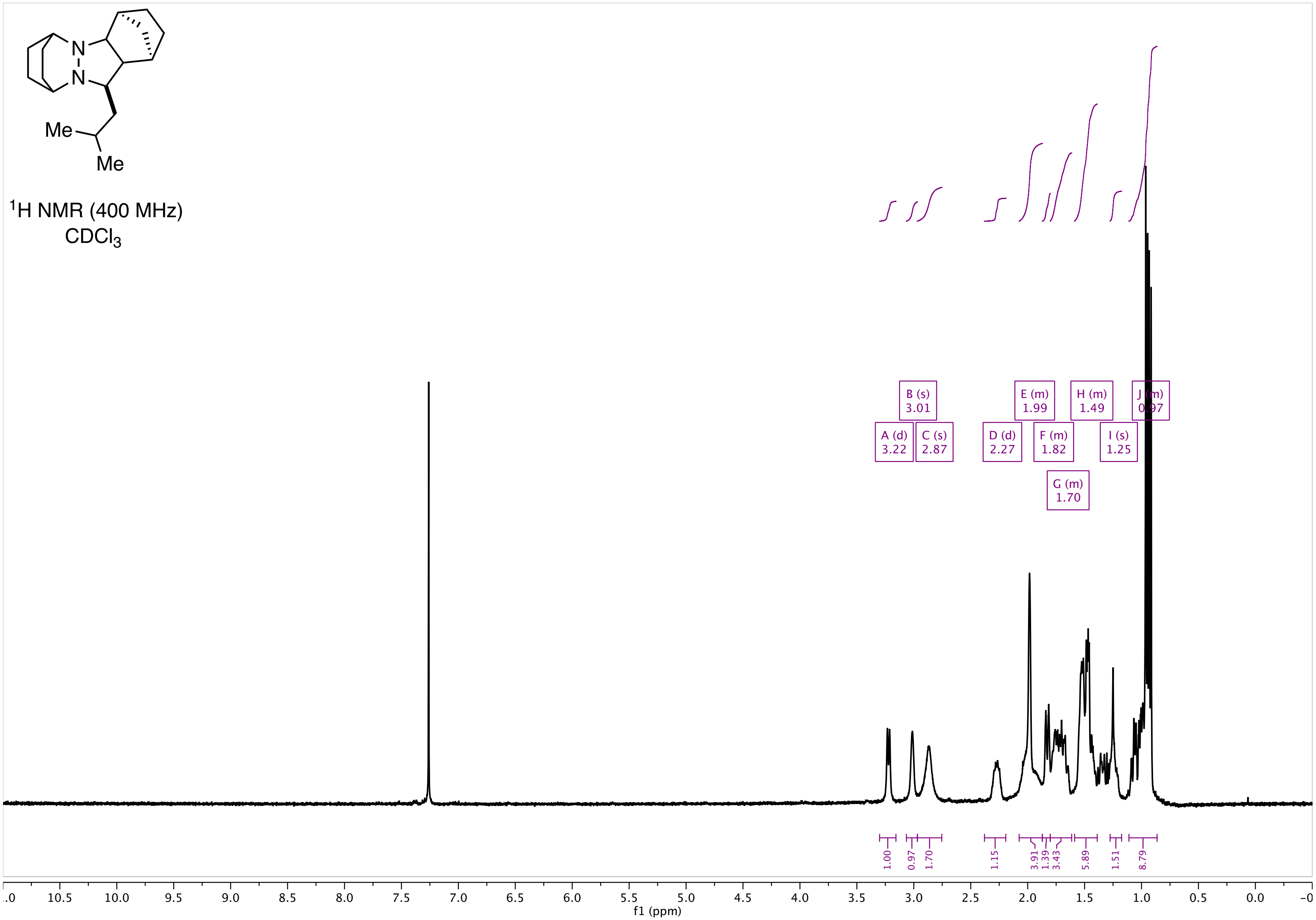

Me
Me
N
N
^{1}H NMR (400 MHz)
CDCl$_3$
A (d) 3.22
B (s) 3.01
C (s) 2.87
D (d) 2.27
E (m) 1.99
F (m) 1.82
G (m) 1.70
H (m) 1.49
I (s) 1.25
J (m) 0.97
1.00
0.97
1.70
1.15
3.91
1.39
3.43
5.89
1.51
8.79
f1 (ppm)

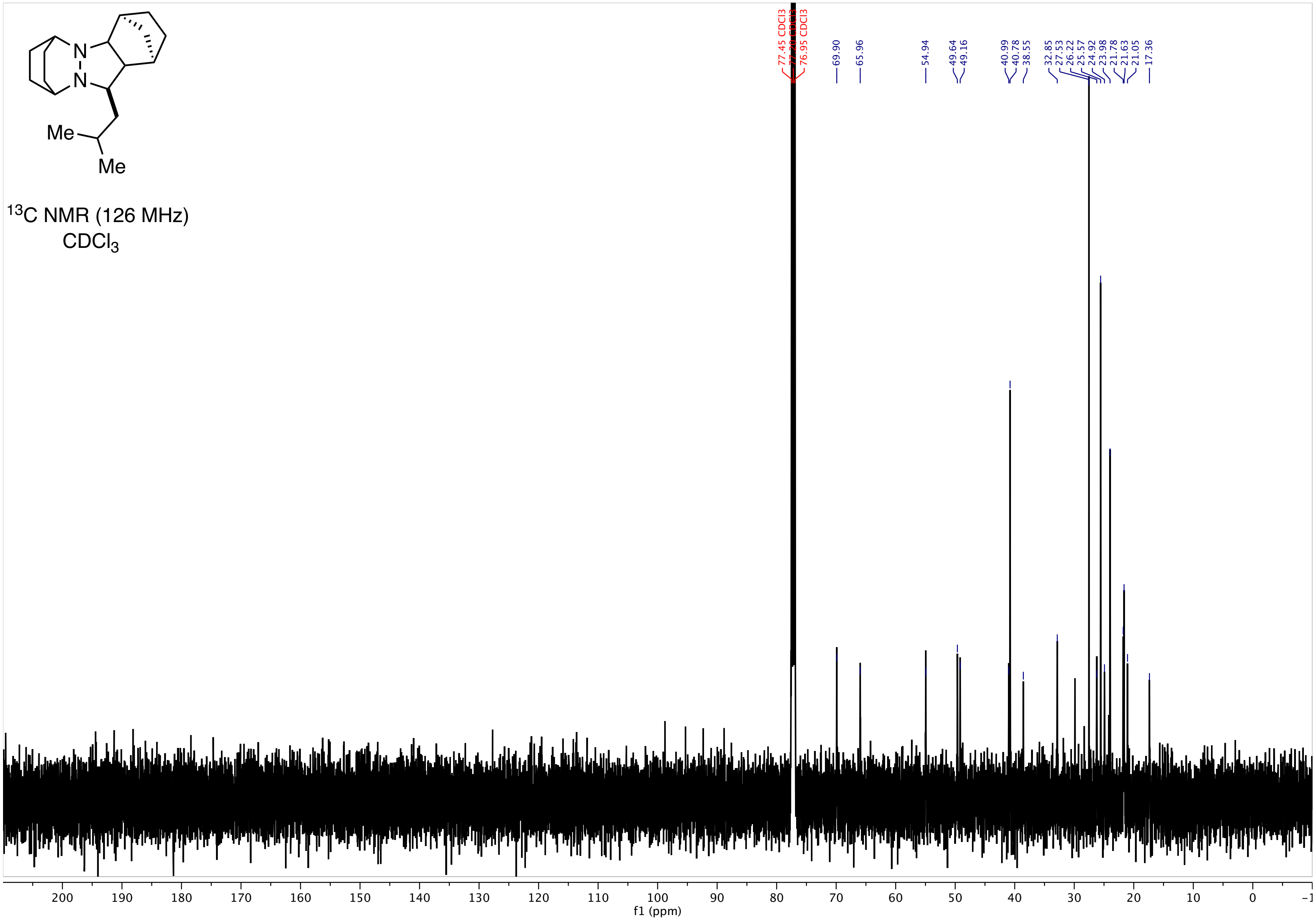

Me
Me
N
N
^{13}C NMR (126 MHz)
CDCl$_3$
77.45 CDCl3
77.20 CDCl3
76.95 CDCl3
69.90
65.96
54.94
49.64
49.16
40.99
40.78
38.55
32.85
27.53
26.22
25.57
24.92
23.98
21.78
21.63
21.05
17.36
f1 (ppm)

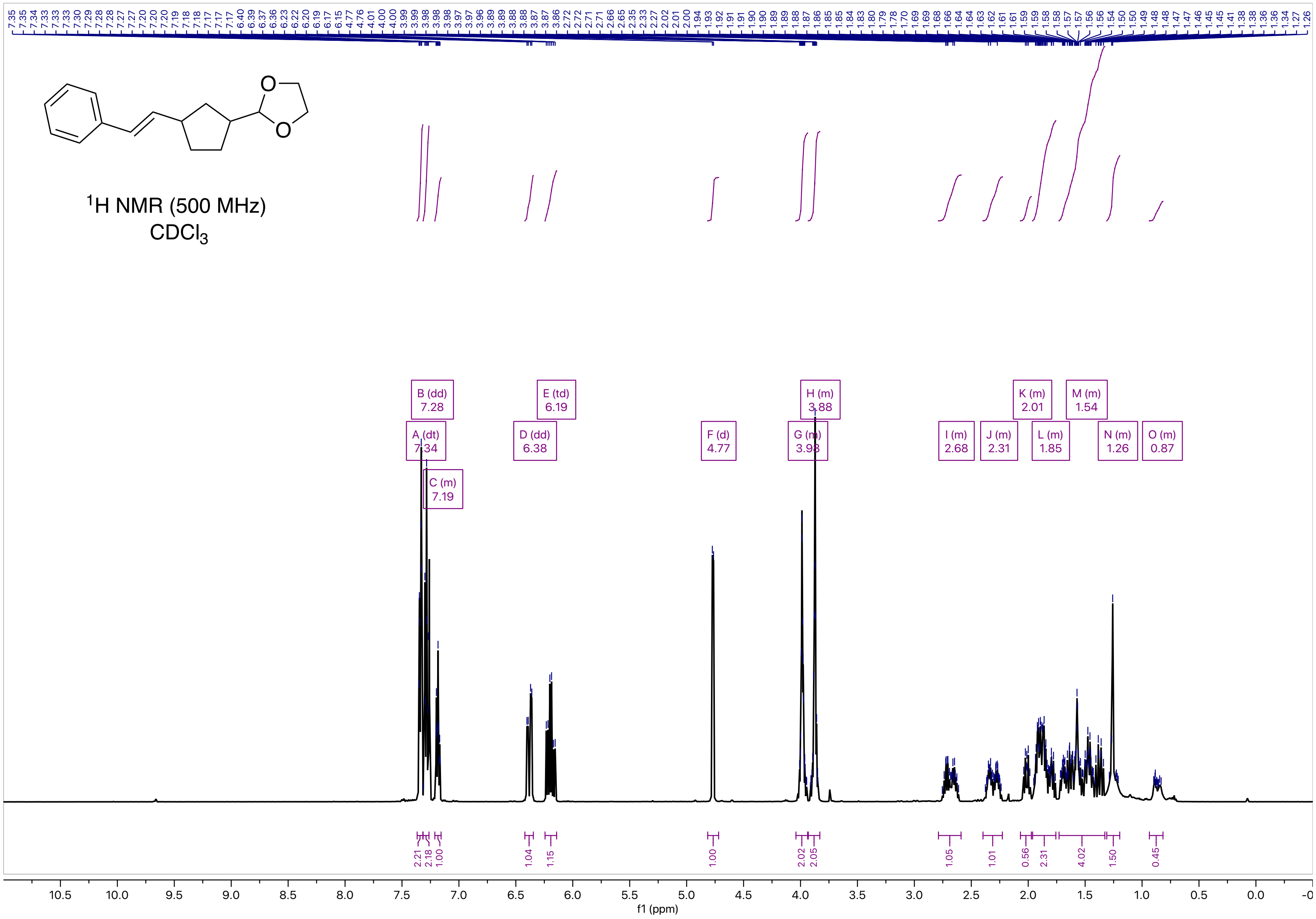

1H NMR (500 MHz)
CDCl3
A (dt) 7.34
B (dd) 7.28
C (m) 7.19
D (dd) 6.38
E (td) 6.19
F (d) 4.77
G (m) 3.98
H (m) 3.88
I (m) 2.68
J (m) 2.31
K (m) 2.01
L (m) 1.85
M (m) 1.54
N (m) 1.26
O (m) 0.87
f1 (ppm)

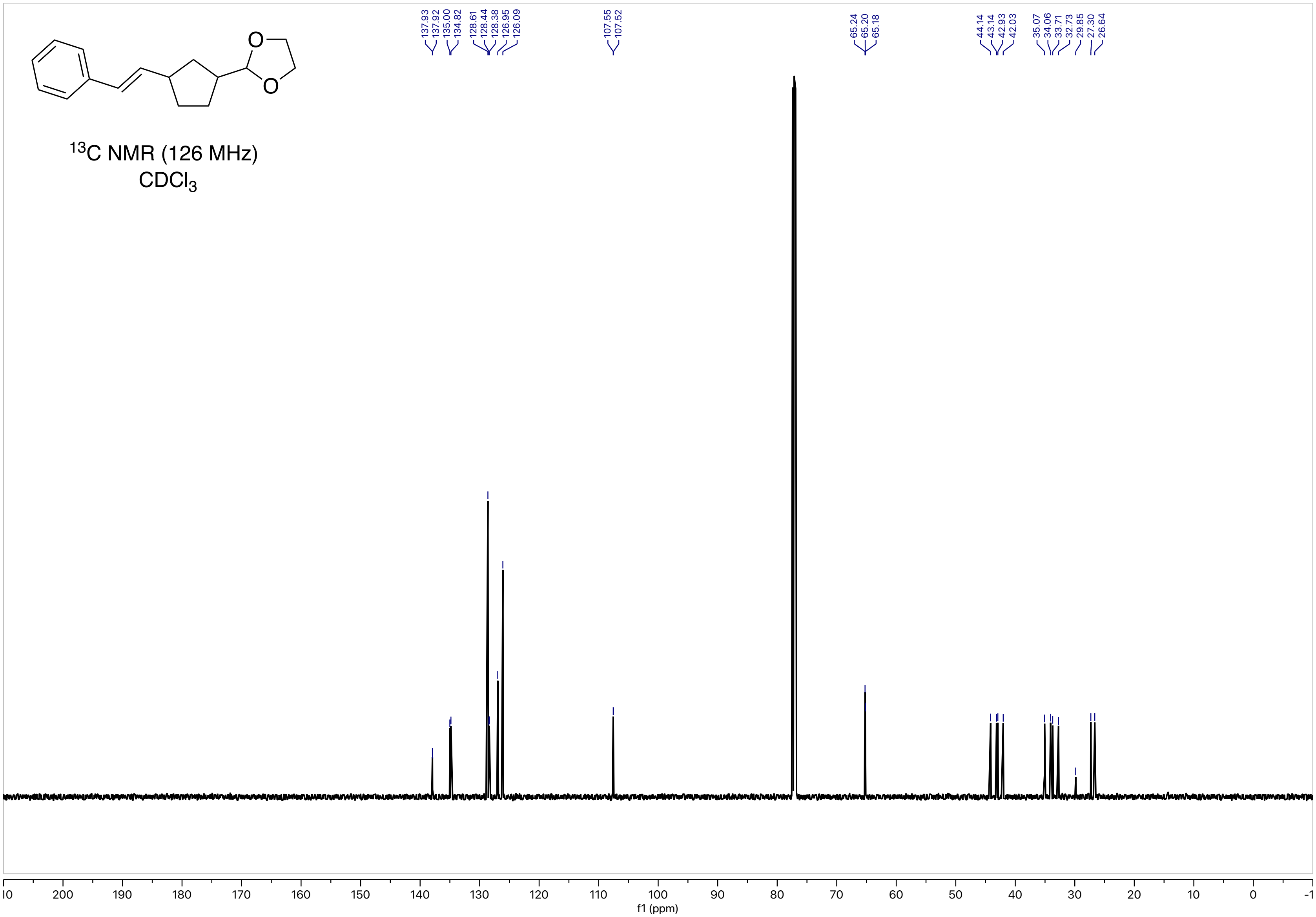

13C NMR (126 MHz)
CDCl3
137.93
137.92
135.00
134.82
128.61
128.44
128.38
126.95
126.09
107.55
107.52
65.24
65.20
65.18
44.14
43.14
42.93
42.03
35.07
34.06
33.71
32.73
29.85
27.30
26.64
200
190
180
170
160
150
140
130
120
110
100
90
80
70
60
50
40
30
20
10
0
f1 (ppm)

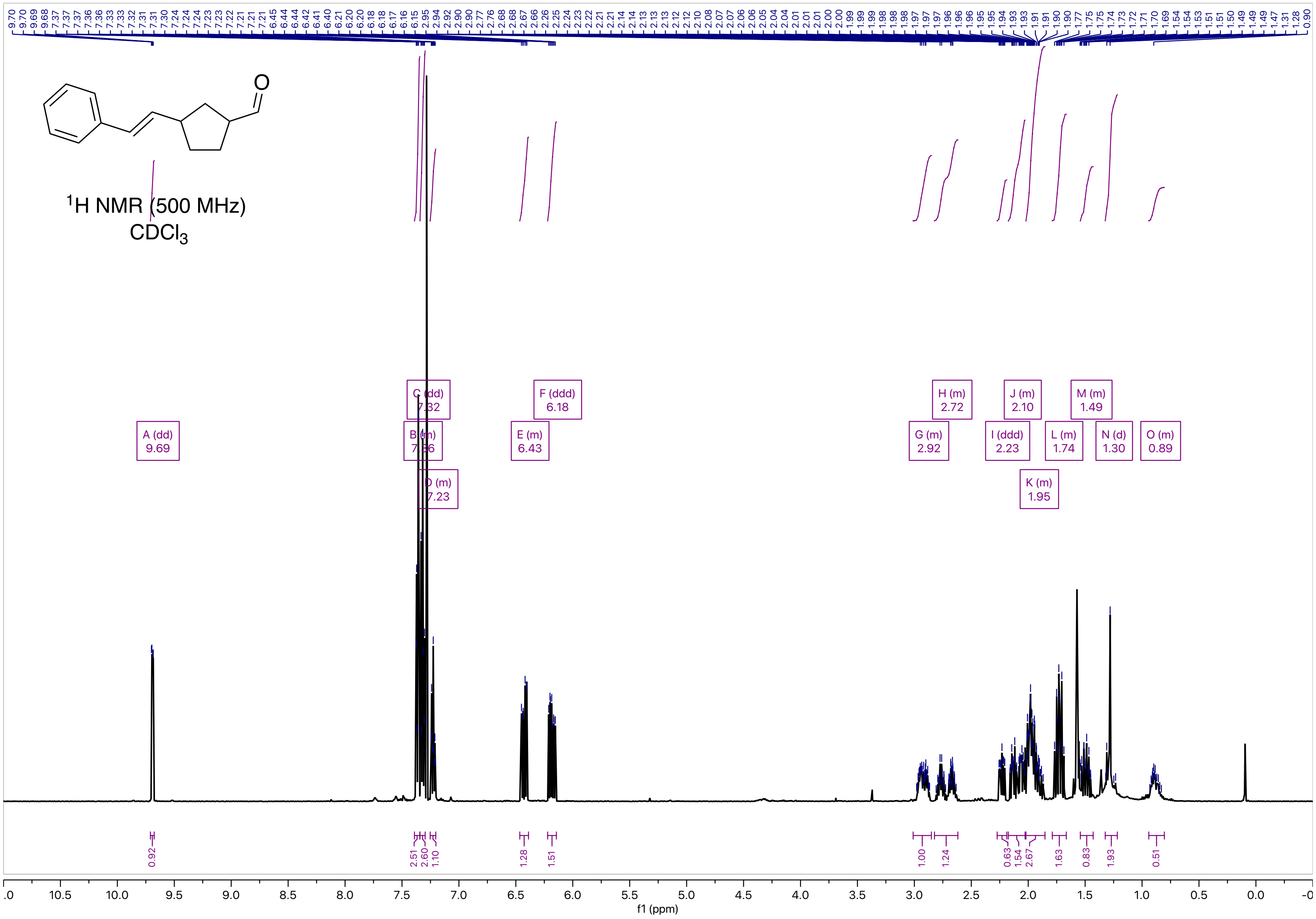

¹H NMR (500 MHz)
CDCl3
A (dd)
9.69
B (m)
7.36
C (dd)
7.32
D (m)
7.23
E (m)
6.43
F (ddd)
6.18
G (m)
2.92
H (m)
2.72
I (ddd)
2.23
J (m)
2.10
K (m)
1.95
L (m)
1.74
M (m)
1.49
N (d)
1.30
O (m)
0.89
f1 (ppm)

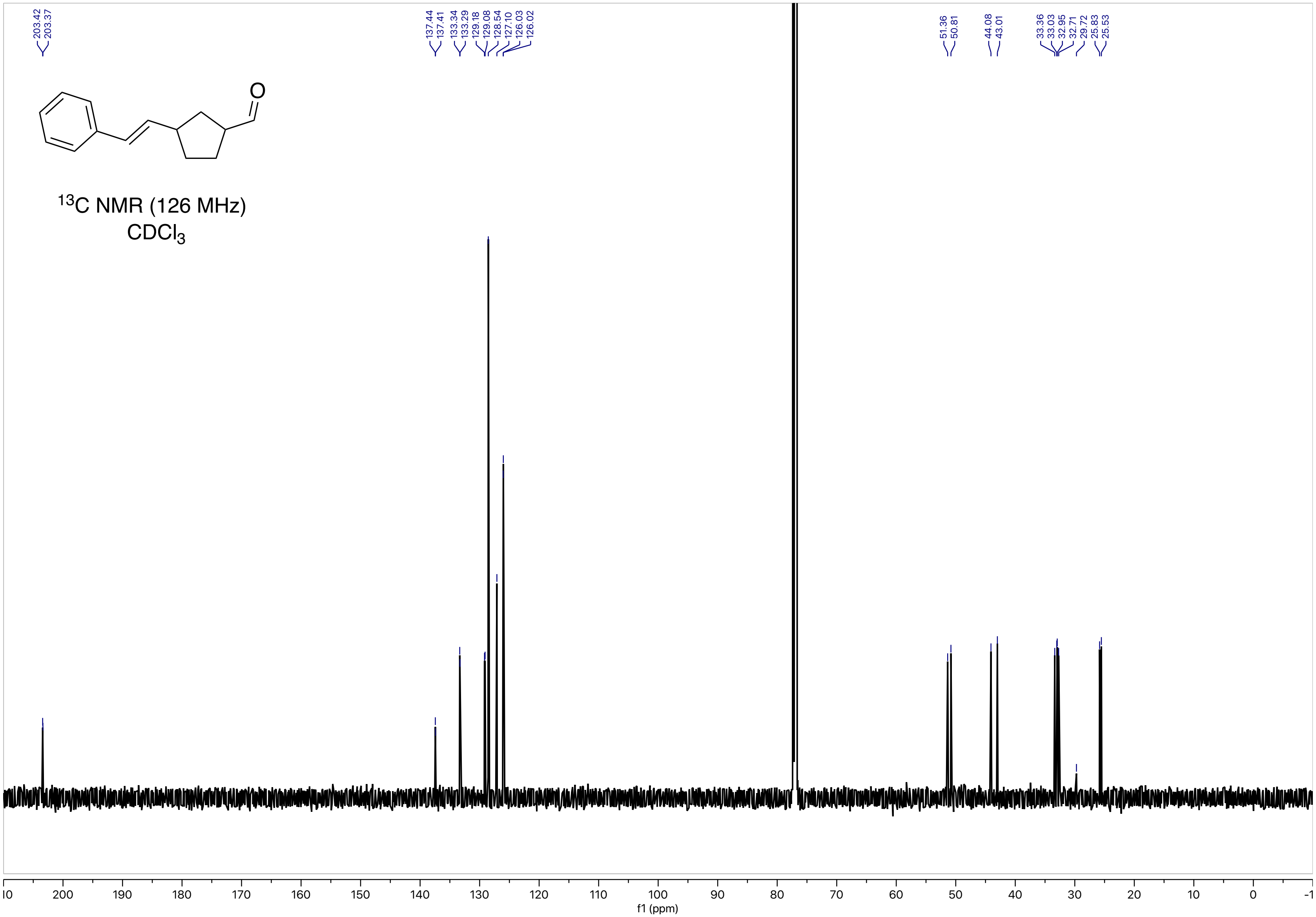

203.42
203.37
137.44
137.41
133.34
133.29
129.18
129.08
128.54
127.10
126.03
126.02
51.36
50.81
44.08
43.01
33.36
33.03
32.95
32.71
29.72
25.83
25.53
O
^{13}C NMR (126 MHz)
$CDCl_3$
f1 (ppm)

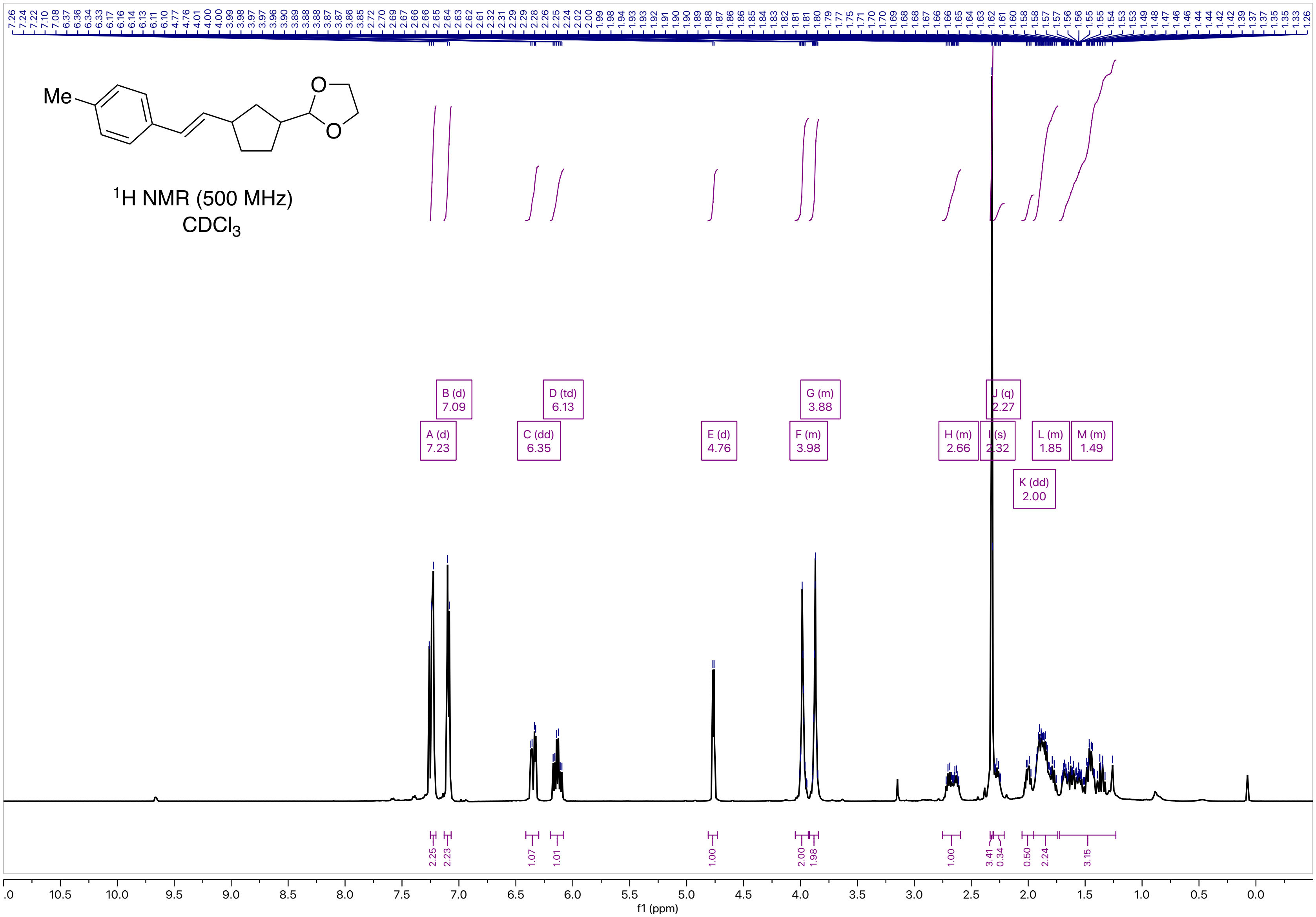
Me
O
O
^{1}H NMR (500 MHz)
$CDCl_3$
A (d) 7.23
B (d) 7.09
C (dd) 6.35
D (td) 6.13
E (d) 4.76
F (m) 3.98
G (m) 3.88
H (m) 2.66
I (s) 2.32
J (q) 2.27
K (dd) 2.00
L (m) 1.85
M (m) 1.49
f1 (ppm)

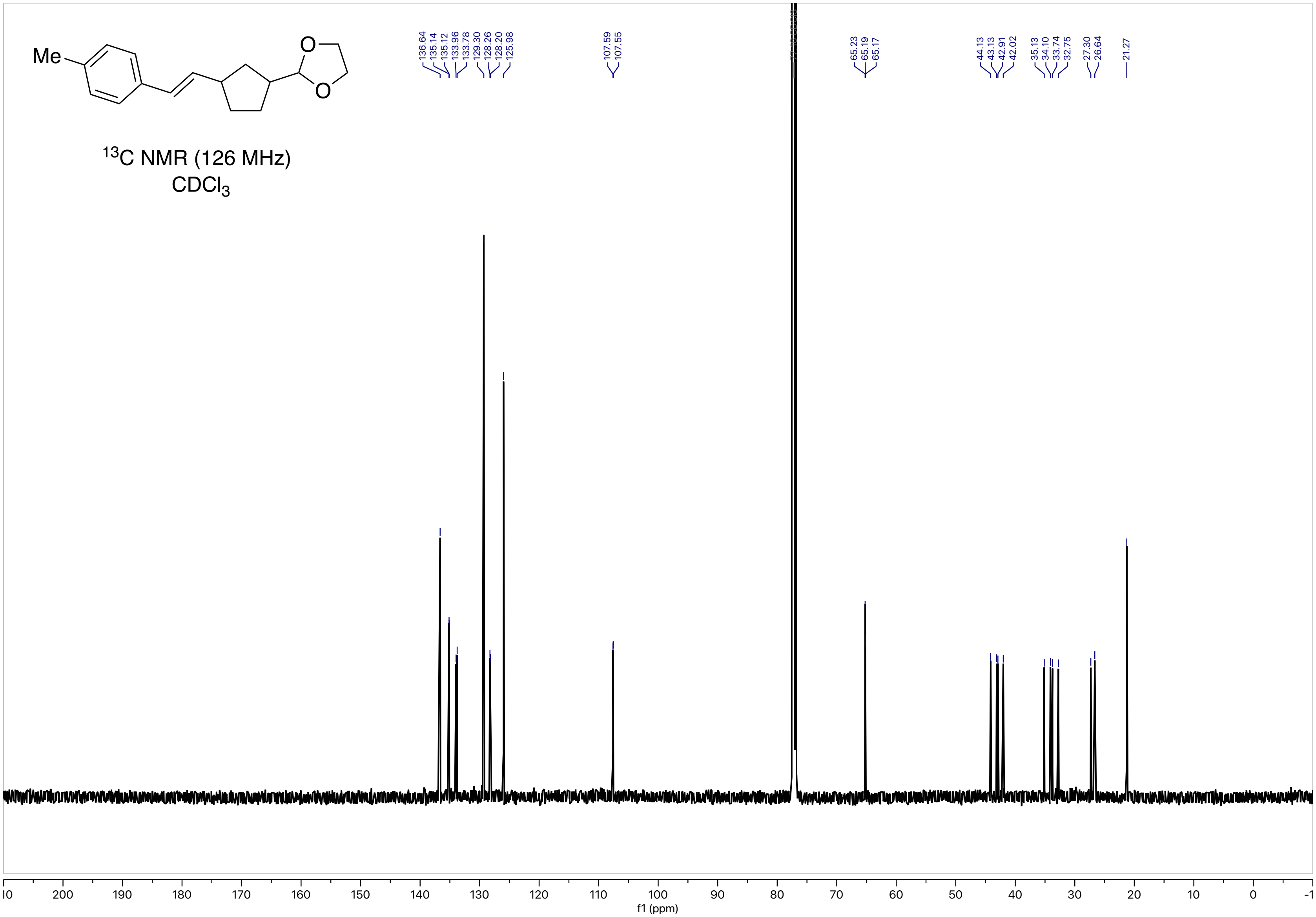
Me
O
O
13C NMR (126 MHz)
CDCl3
136.64
135.14
135.12
133.96
133.78
129.30
128.26
128.20
125.98
107.59
107.55
65.23
65.19
65.17
44.13
43.13
42.91
42.02
35.13
34.10
33.74
32.75
27.30
26.64
21.27
f1 (ppm)

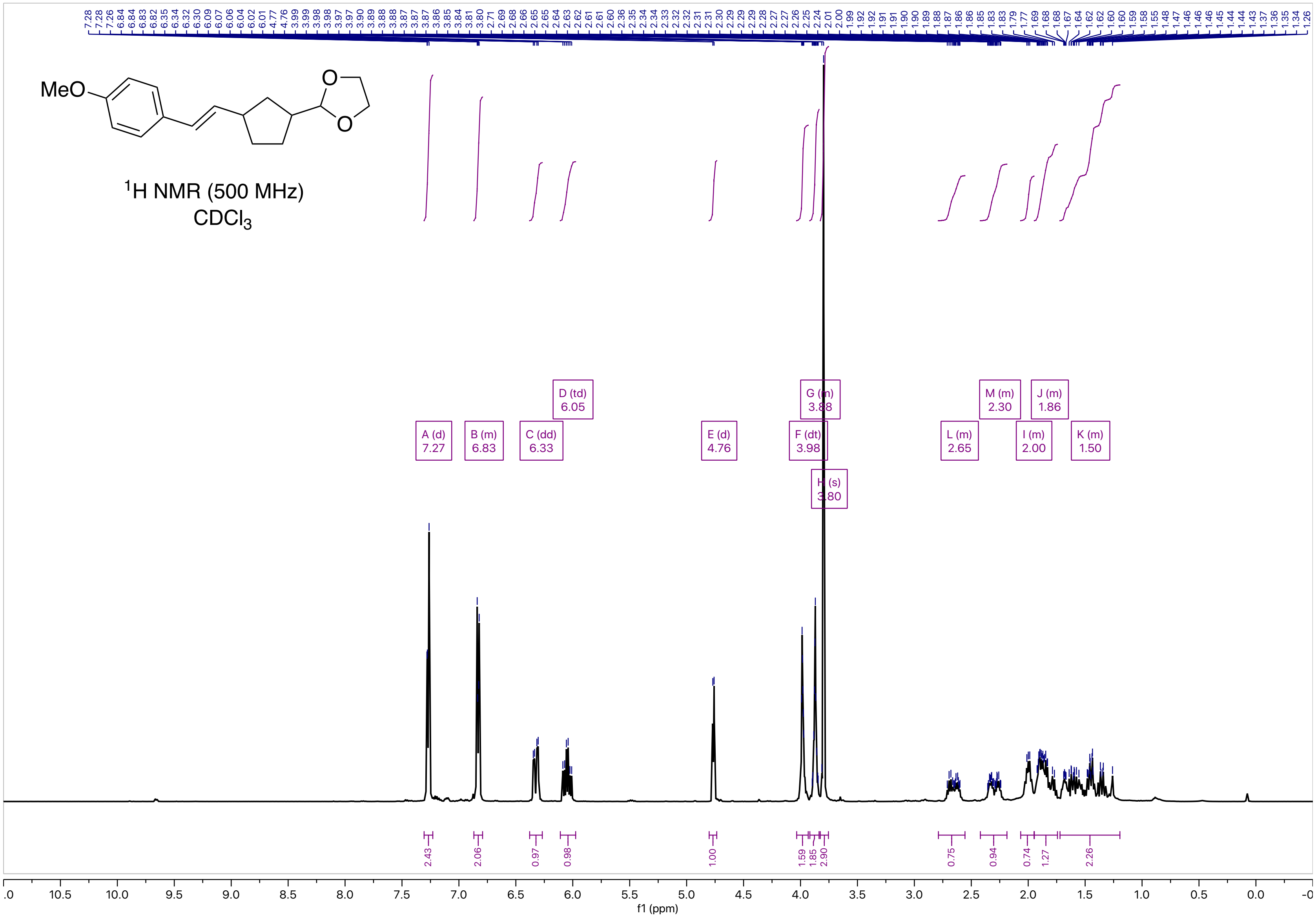

MeO
^{1}H NMR (500 MHz)
CDCl$_3$
A (d) 7.27
B (m) 6.83
C (dd) 6.33
D (td) 6.05
E (d) 4.76
F (dt) 3.98
G (m) 3.88
H (s) 3.80
L (m) 2.65
M (m) 2.30
I (m) 2.00
J (m) 1.86
K (m) 1.50
f1 (ppm)

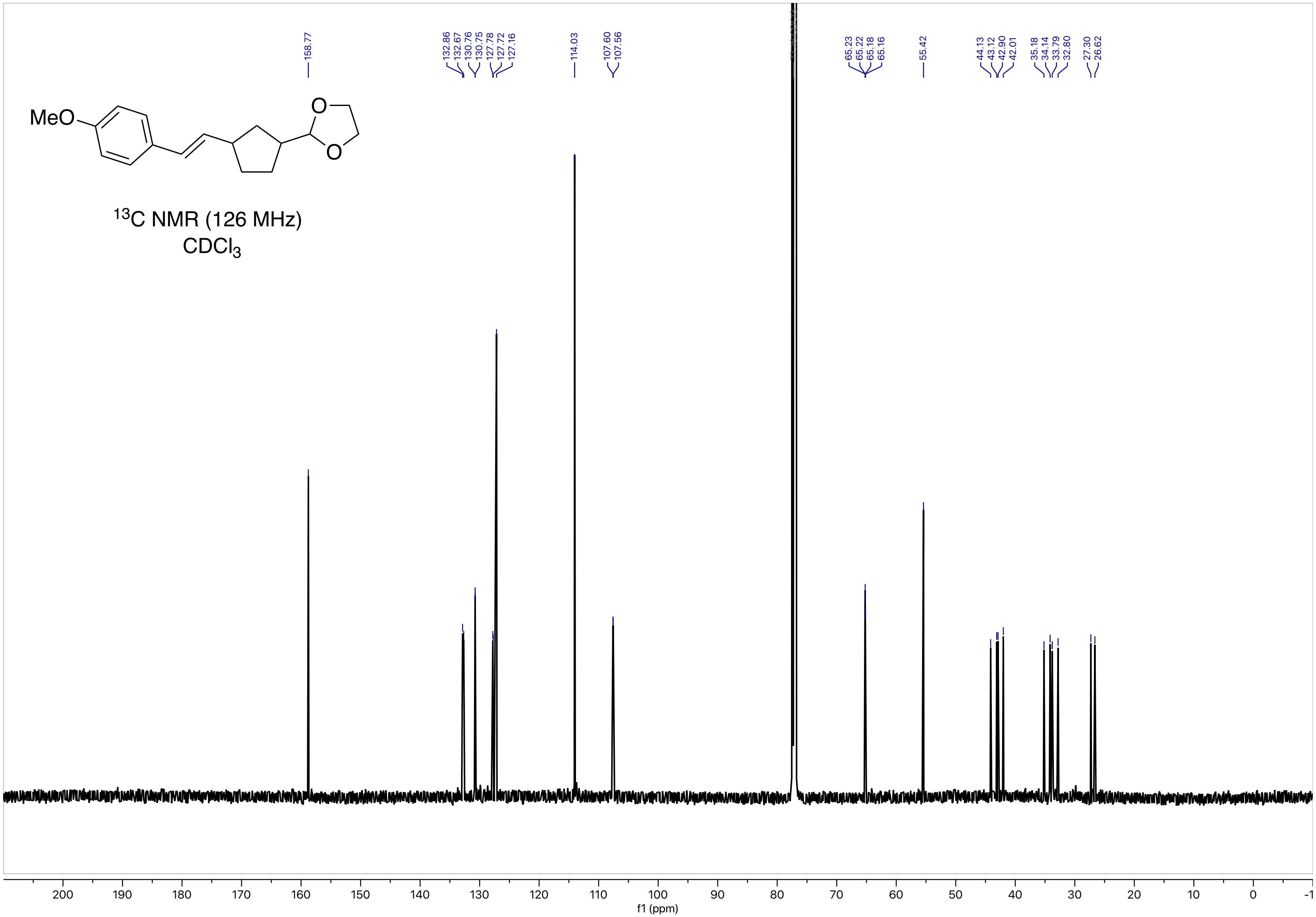

MeO
O
O
158.77
132.86
132.67
130.76
130.75
127.78
127.72
127.16
114.03
107.60
107.56
65.23
65.22
65.18
65.16
55.42
44.13
43.12
42.90
42.01
35.18
34.14
33.79
32.80
27.30
26.62
^{13}C NMR (126 MHz)
$CDCl_3$
f1 (ppm)

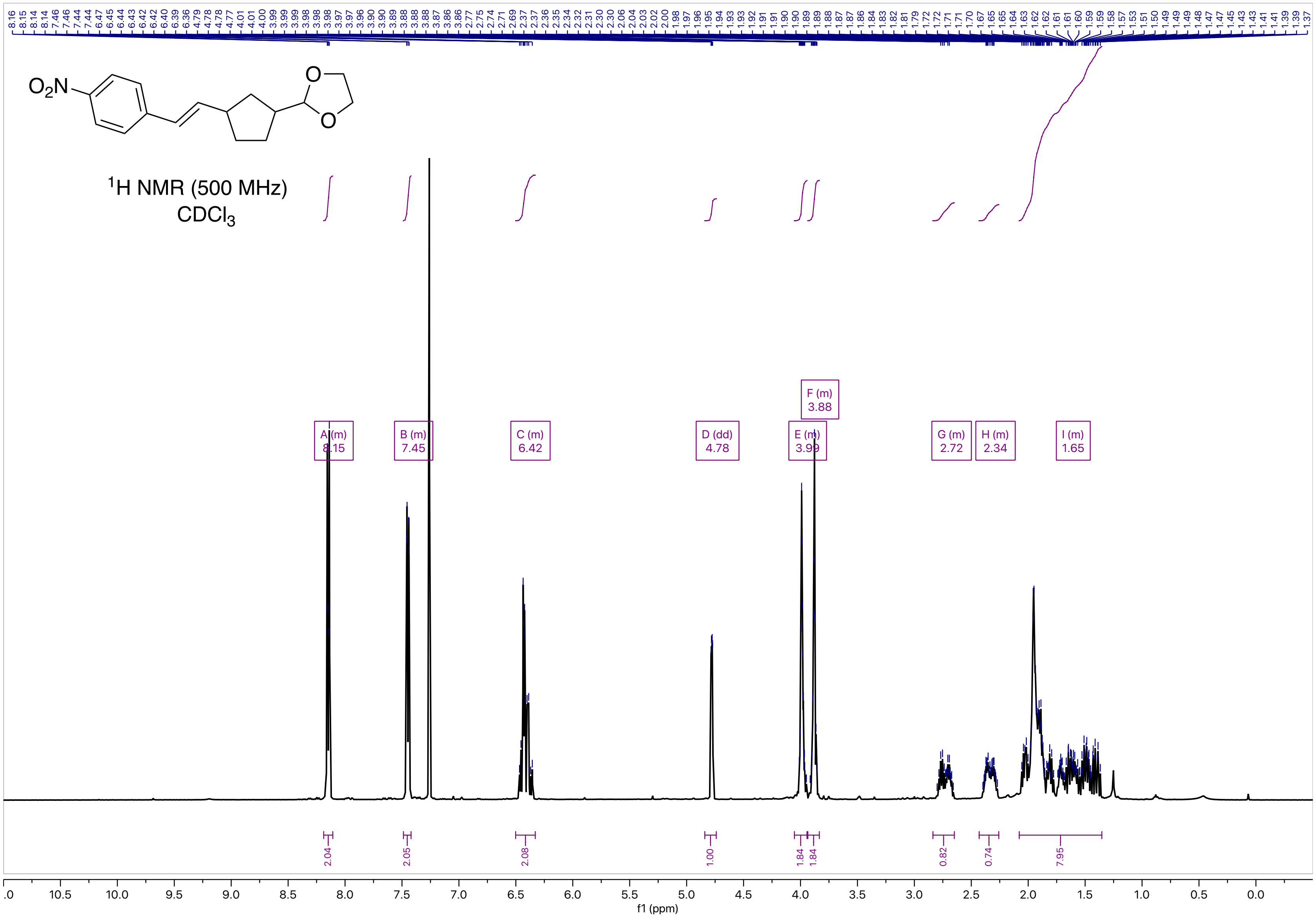

O2N
O
O
1H NMR (500 MHz)
CDCl3
A (m)
8.15
B (m)
7.45
C (m)
6.42
D (dd)
4.78
E (m)
3.99
F (m)
3.88
G (m)
2.72
H (m)
2.34
I (m)
1.65
2.04
2.05
2.08
1.00
1.84
1.84
0.82
0.74
7.95
f1 (ppm)

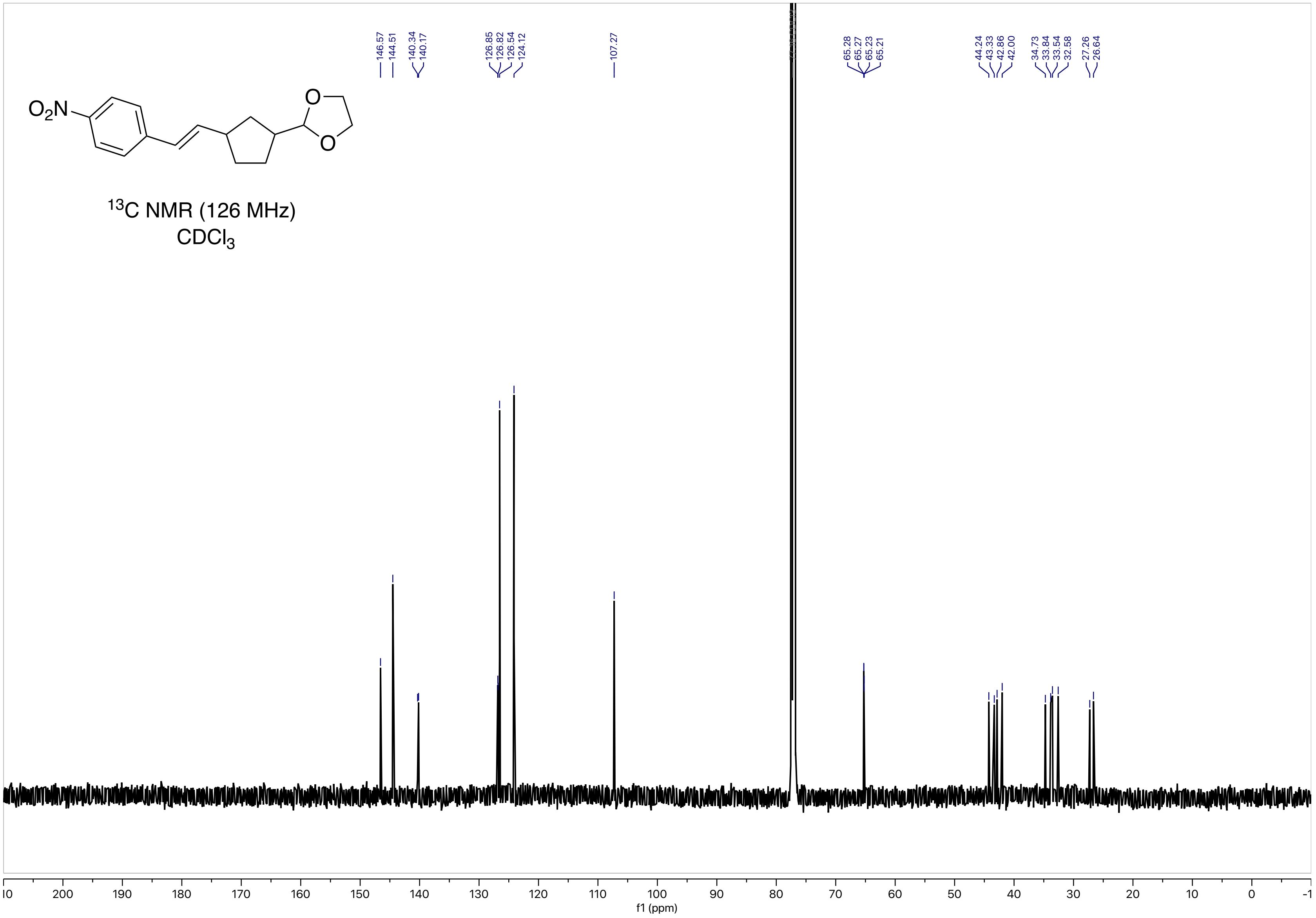

O2N
O
O
13C NMR (126 MHz)
CDCl3
146.57
144.51
140.34
140.17
126.85
126.82
126.54
124.12
107.27
65.28
65.27
65.23
65.21
44.24
43.33
42.86
42.00
34.73
33.84
33.54
32.58
27.26
26.64
f1 (ppm)

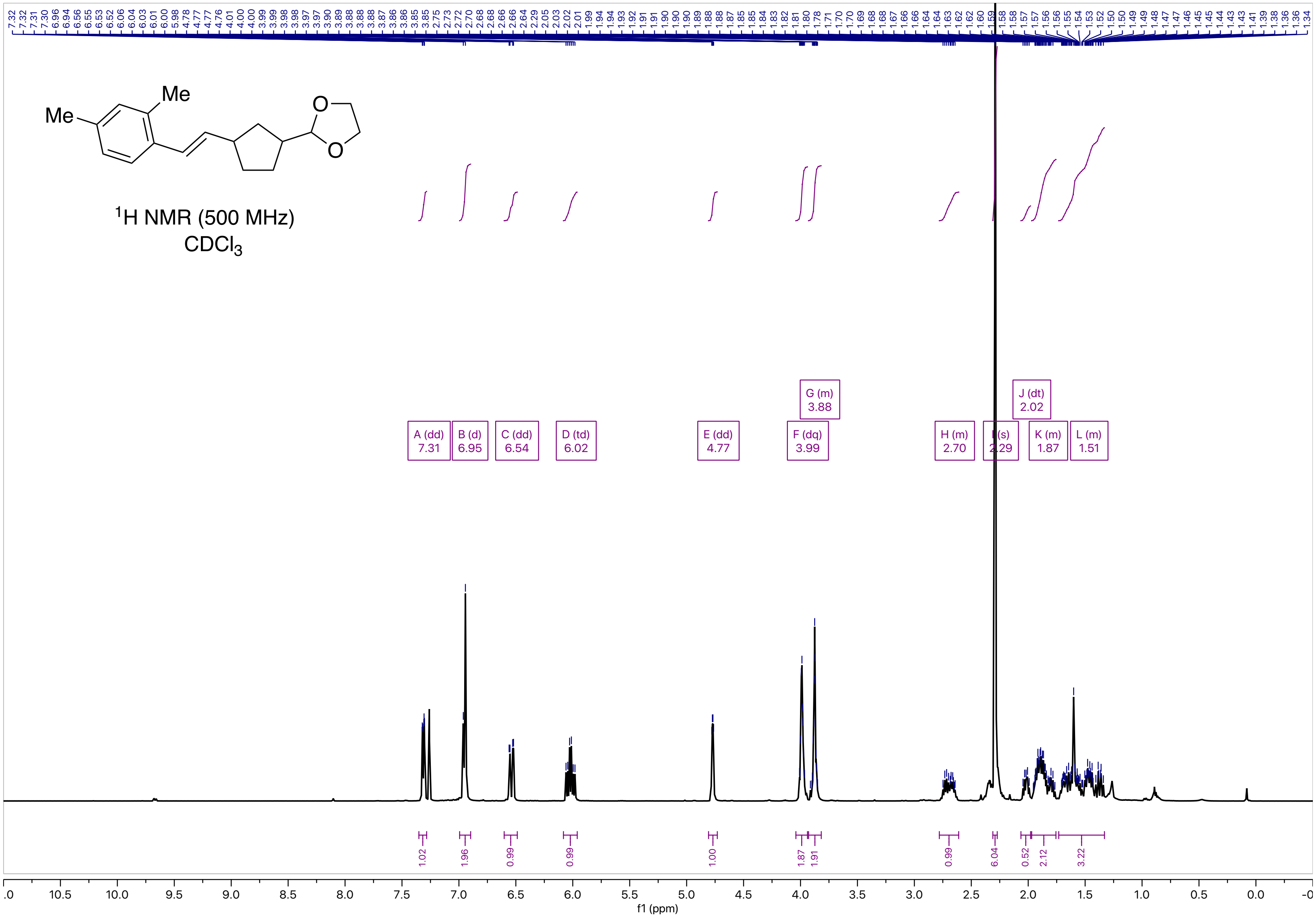

Me
Me
O
O
^{1}H NMR (500 MHz)
CDCl$_3$
A (dd) 7.31
B (d) 6.95
C (dd) 6.54
D (td) 6.02
E (dd) 4.77
F (dq) 3.99
G (m) 3.88
H (m) 2.70
I (s) 2.29
J (dt) 2.02
K (m) 1.87
L (m) 1.51
1.02
1.96
0.99
0.99
1.00
1.87
1.91
0.99
6.04
0.52
2.12
3.22
f1 (ppm)

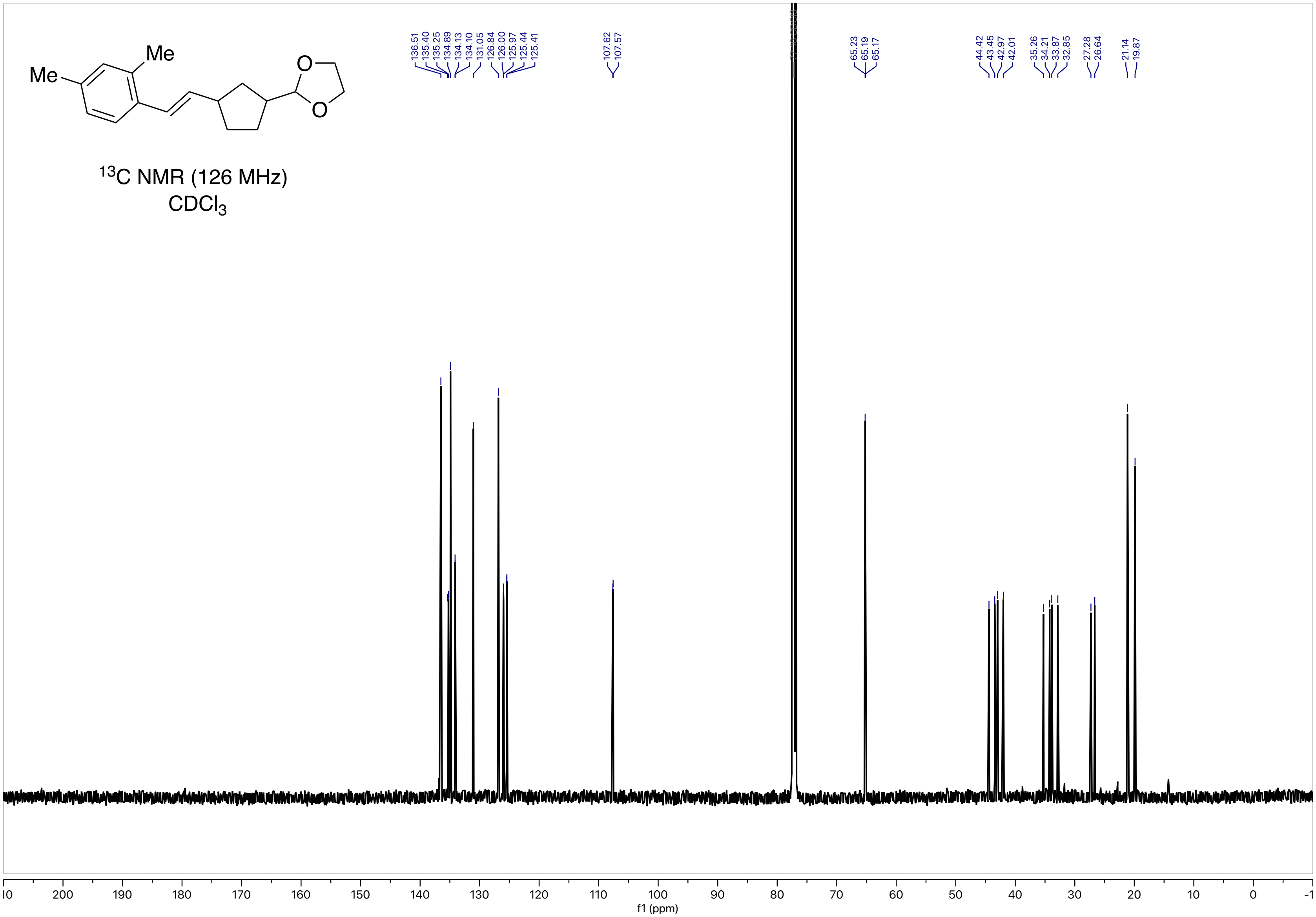

Me
Me
O
O
^{13}C NMR (126 MHz)
$CDCl_3$
136.51
135.40
135.25
134.89
134.13
134.10
131.05
126.84
126.00
125.97
125.44
125.41
107.62
107.57
65.23
65.19
65.17
44.42
43.45
42.97
42.01
35.26
34.21
33.87
32.85
27.28
26.64
21.14
19.87
f1 (ppm)

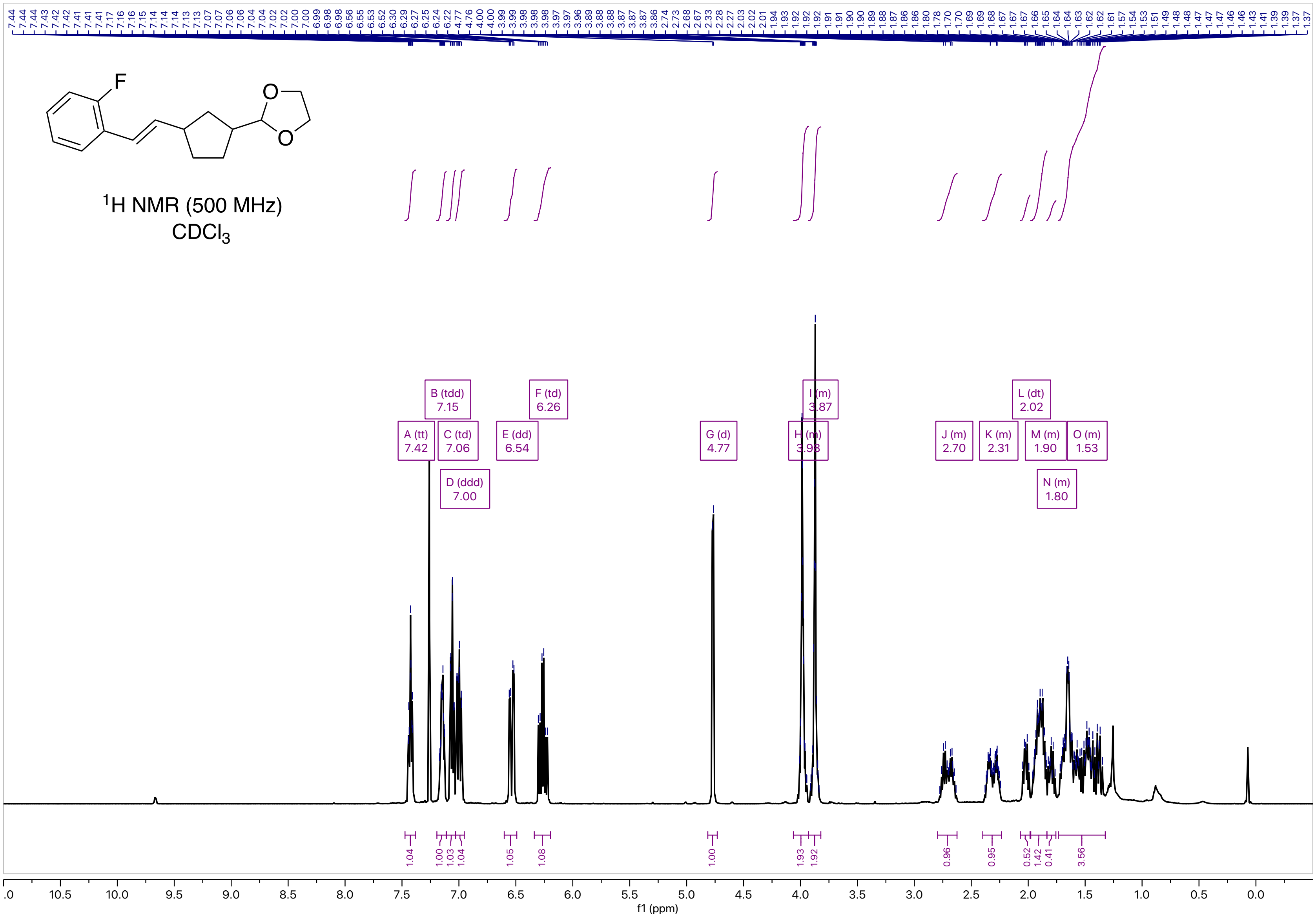

F
O
O
^{1}H NMR (500 MHz)
CDCl$_3$
A (tt) 7.42
B (tdd) 7.15
C (td) 7.06
D (ddd) 7.00
E (dd) 6.54
F (td) 6.26
G (d) 4.77
H (m) 3.98
I (m) 3.87
J (m) 2.70
K (m) 2.31
L (dt) 2.02
M (m) 1.90
N (m) 1.80
O (m) 1.53
f1 (ppm)

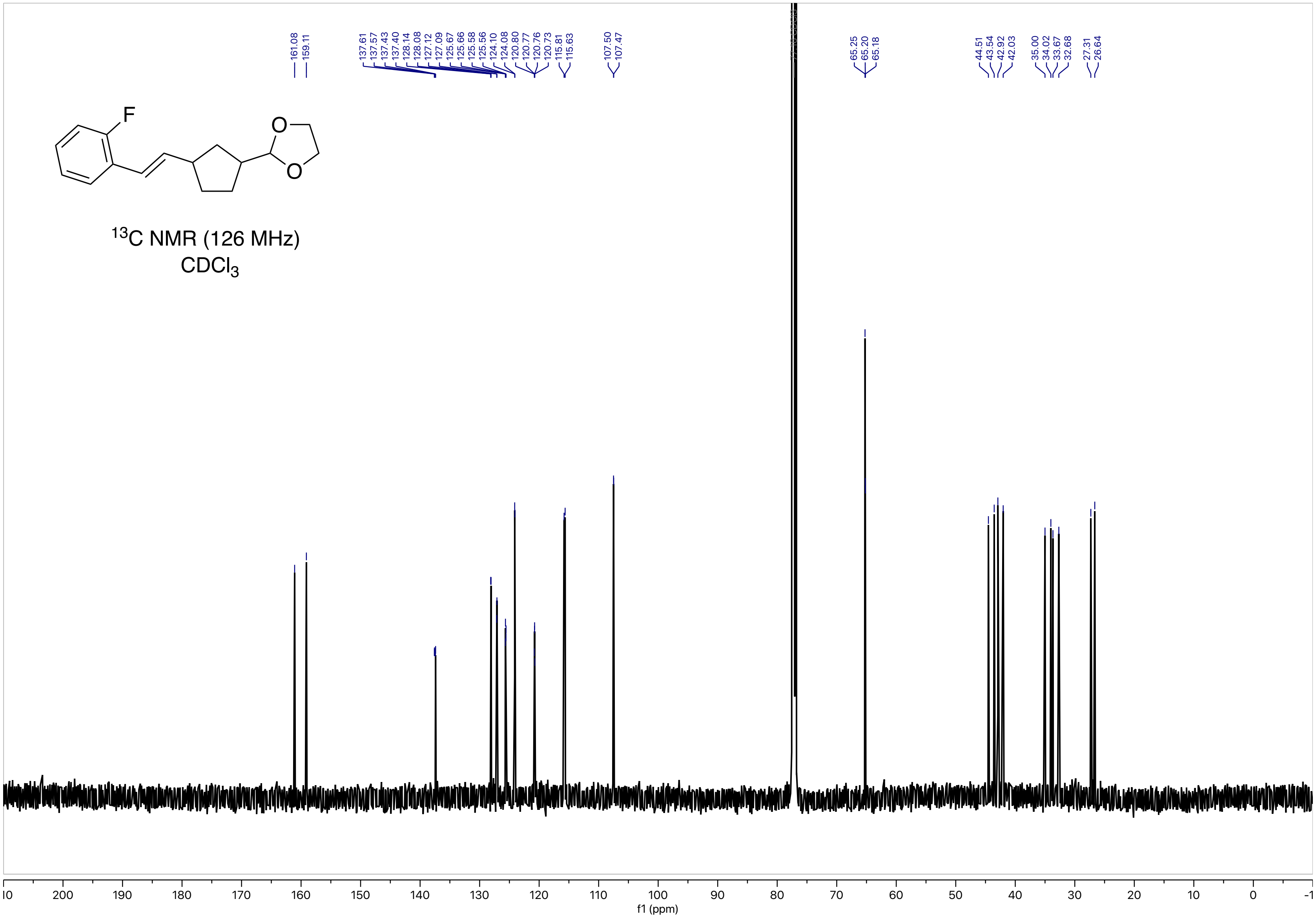

F
O
O
^{13}C NMR (126 MHz)
CDCl$_3$
161.08
159.11
137.61
137.57
137.43
137.40
128.14
128.08
127.12
127.09
125.67
125.66
125.58
125.56
124.10
124.08
120.80
120.77
120.76
120.73
115.81
115.63
107.50
107.47
65.25
65.20
65.18
44.51
43.54
42.92
42.03
35.00
34.02
33.67
32.68
27.31
26.64
f1 (ppm)

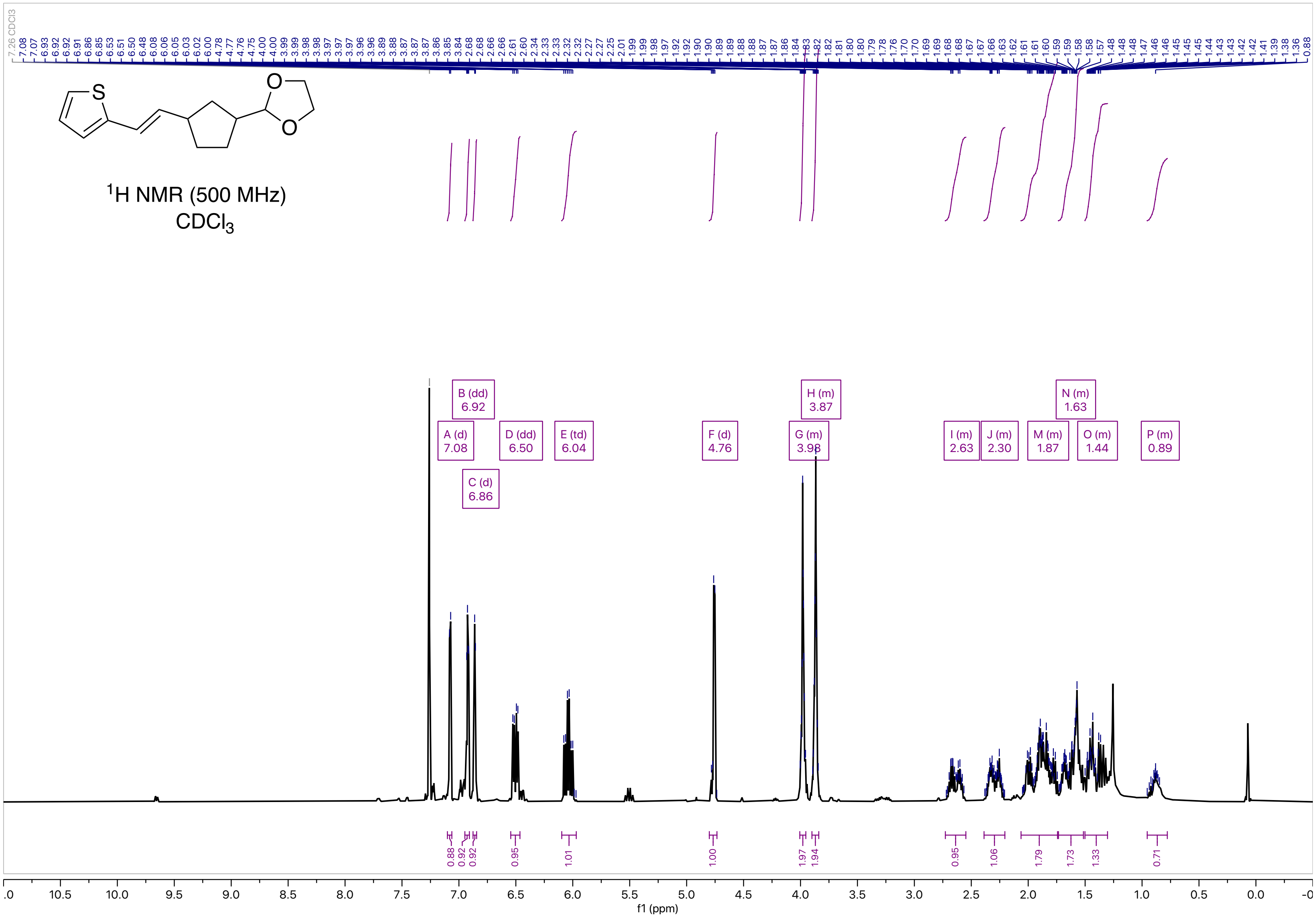

S
O
O
1H NMR (500 MHz)
CDCl3
A (d) 7.08
B (dd) 6.92
C (d) 6.86
D (dd) 6.50
E (td) 6.04
F (d) 4.76
G (m) 3.98
H (m) 3.87
I (m) 2.63
J (m) 2.30
M (m) 1.87
N (m) 1.63
O (m) 1.44
P (m) 0.89
0.88
0.92
0.92
0.95
1.01
1.00
1.97
1.94
0.95
1.06
1.79
1.73
1.33
0.71
f1 (ppm)

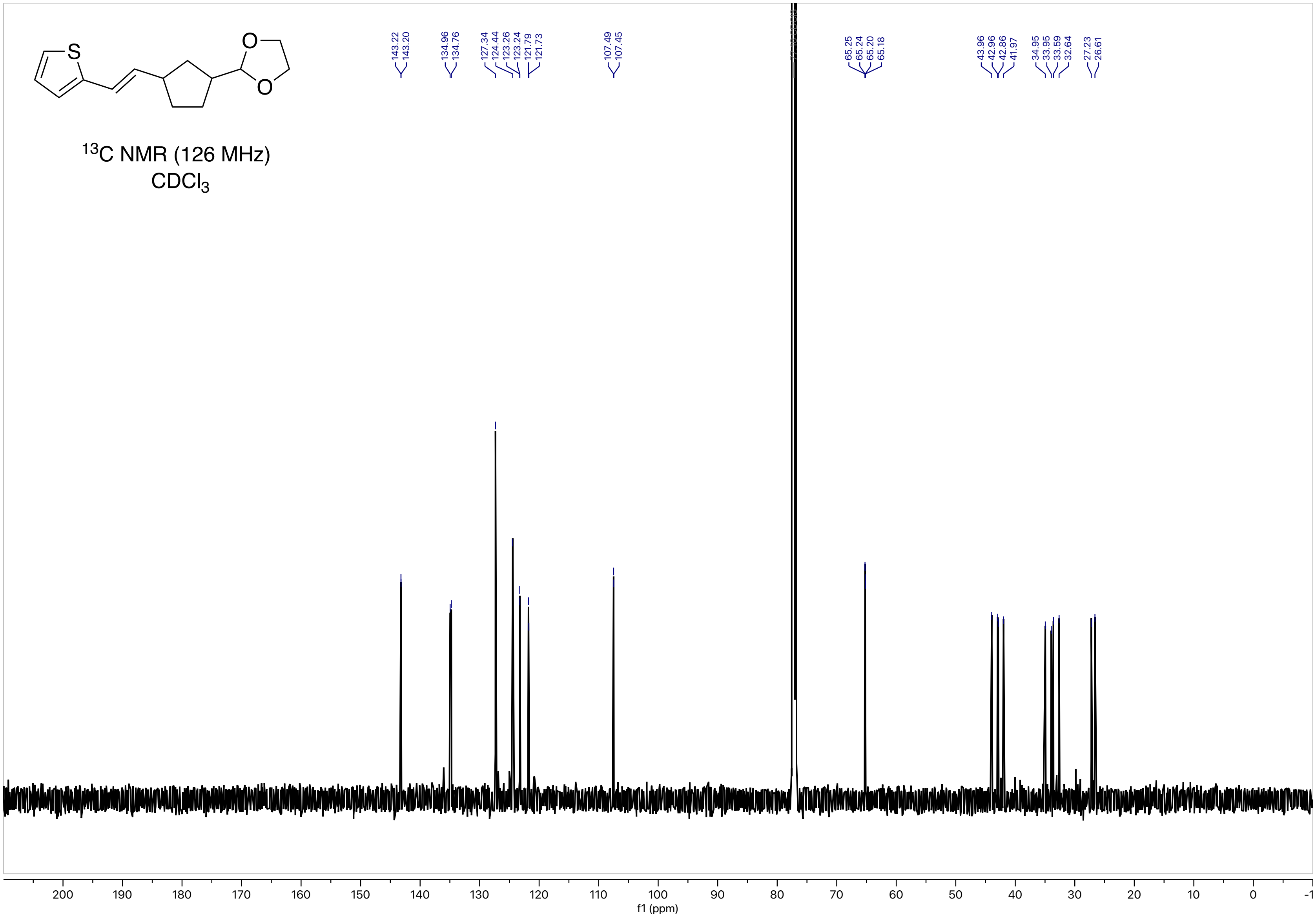
S
O
O
^{13}C NMR (126 MHz)
CDCl$_3$
143.22
143.20
134.96
134.76
127.34
124.44
123.26
123.24
121.79
121.73
107.49
107.45
65.25
65.24
65.20
65.18
43.96
42.96
42.86
41.97
34.95
33.95
33.59
32.64
27.23
26.61
200
190
180
170
160
150
140
130
120
110
100
90
80
70
60
50
40
30
20
10
0
-1
f1 (ppm)

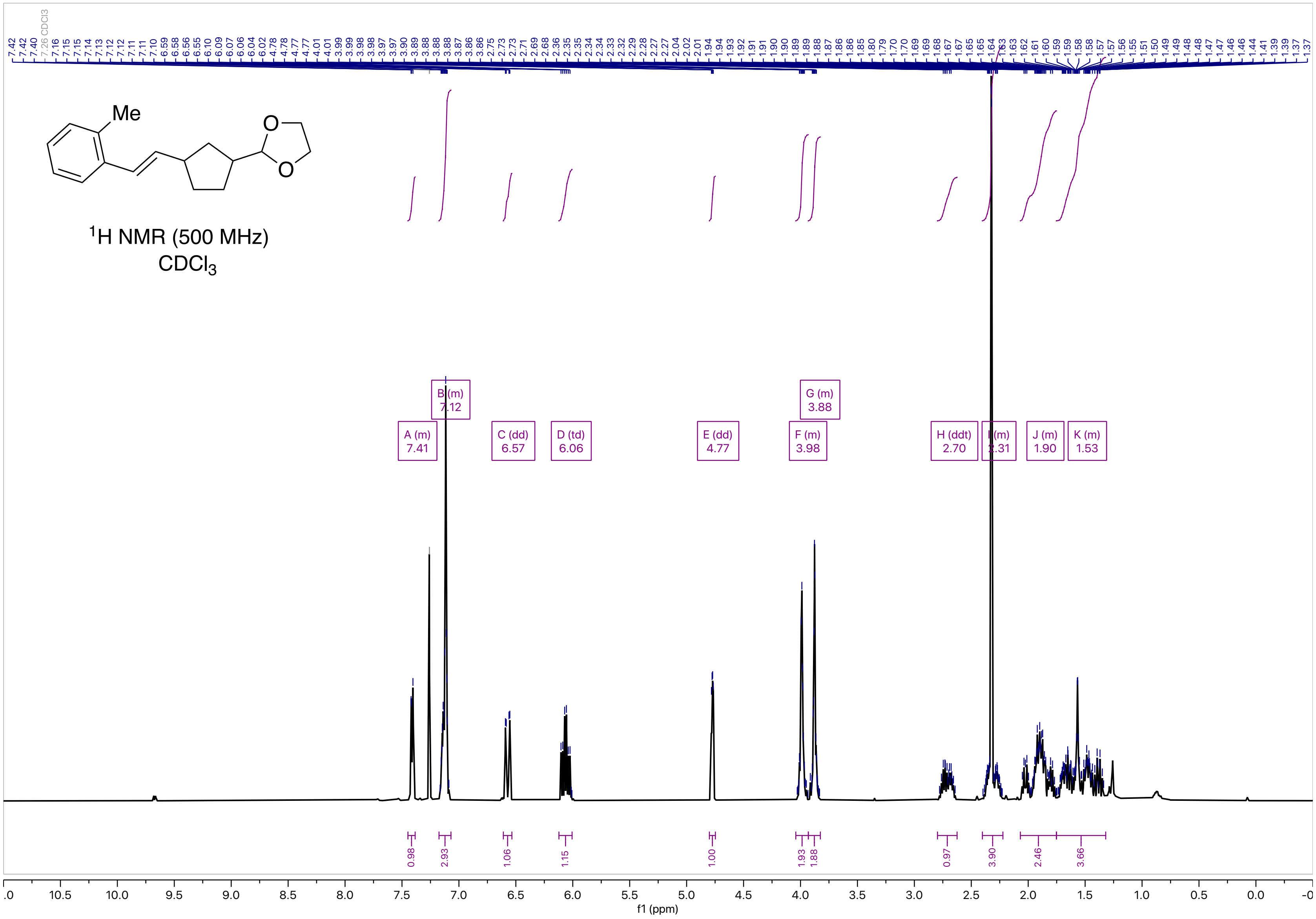

^{1}H NMR (500 MHz)
$CDCl_3$
A (m) 7.41
B (m) 7.12
C (dd) 6.57
D (td) 6.06
E (dd) 4.77
F (m) 3.98
G (m) 3.88
H (ddt) 2.70
I (m) 2.31
J (m) 1.90
K (m) 1.53
0.98
2.93
1.06
1.15
1.00
1.93
1.88
0.97
3.90
2.46
3.66
f1 (ppm)

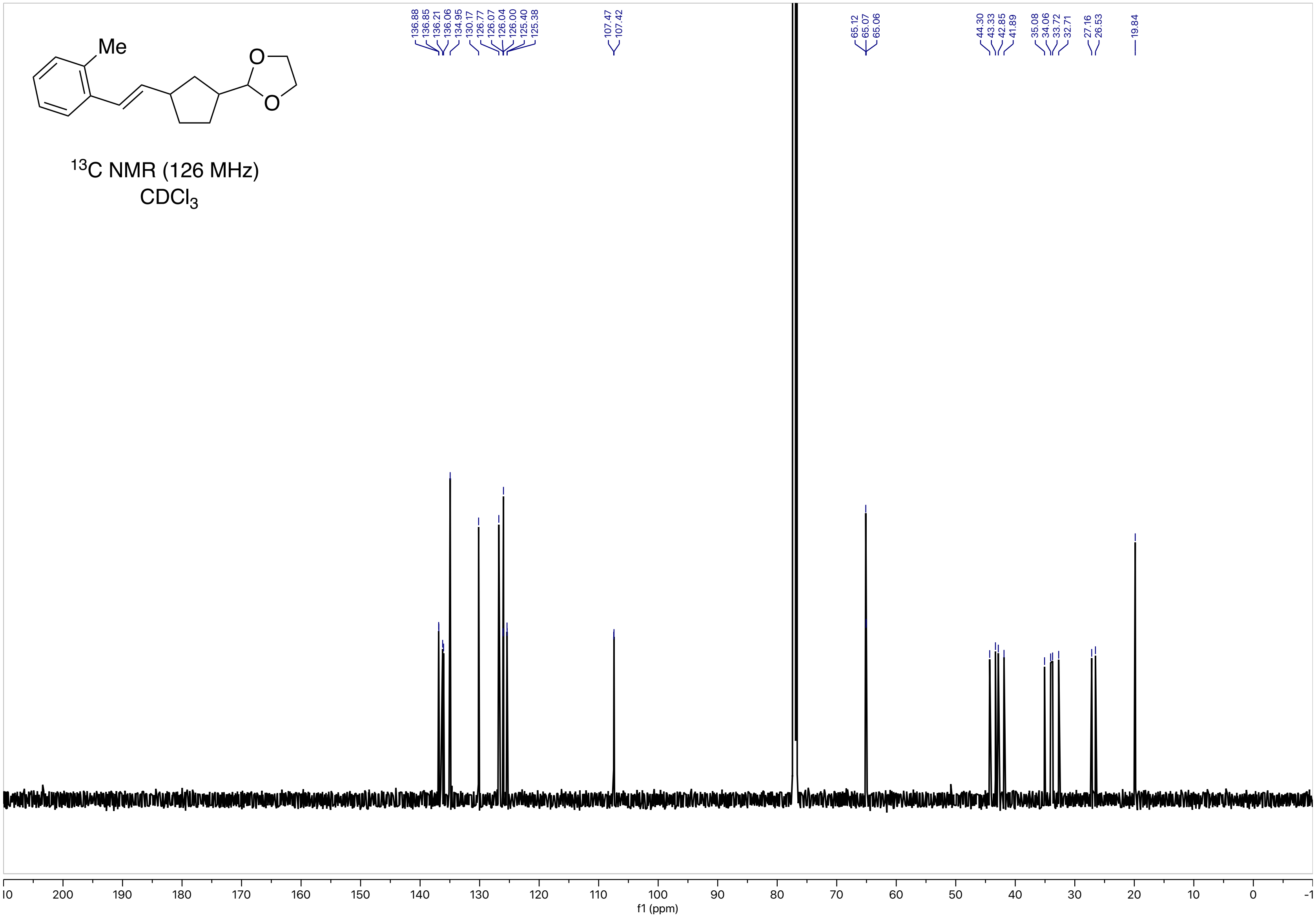
Me
O
O
^{13}C NMR (126 MHz)
CDCl$_3$
136.88
136.85
136.21
136.06
134.95
130.17
126.77
126.07
126.04
126.00
125.40
125.38
107.47
107.42
65.12
65.07
65.06
44.30
43.33
42.85
41.89
35.08
34.06
33.72
32.71
27.16
26.53
19.84
f1 (ppm)

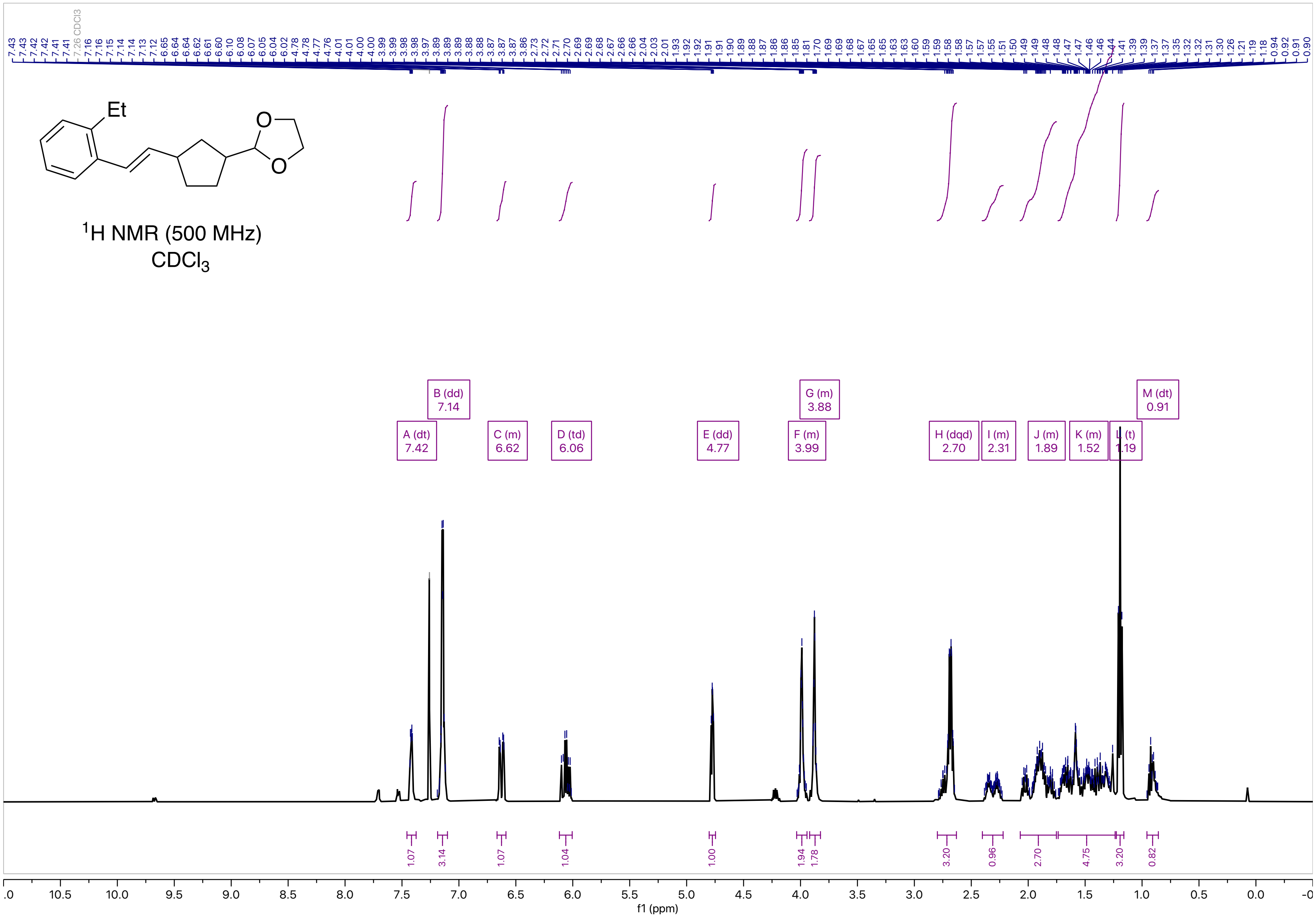
Et
O
O
¹H NMR (500 MHz)
CDCl3
A (dt) 7.42
B (dd) 7.14
C (m) 6.62
D (td) 6.06
E (dd) 4.77
F (m) 3.99
G (m) 3.88
H (dqd) 2.70
I (m) 2.31
J (m) 1.89
K (m) 1.52
L (t) 1.19
M (dt) 0.91
f1 (ppm)

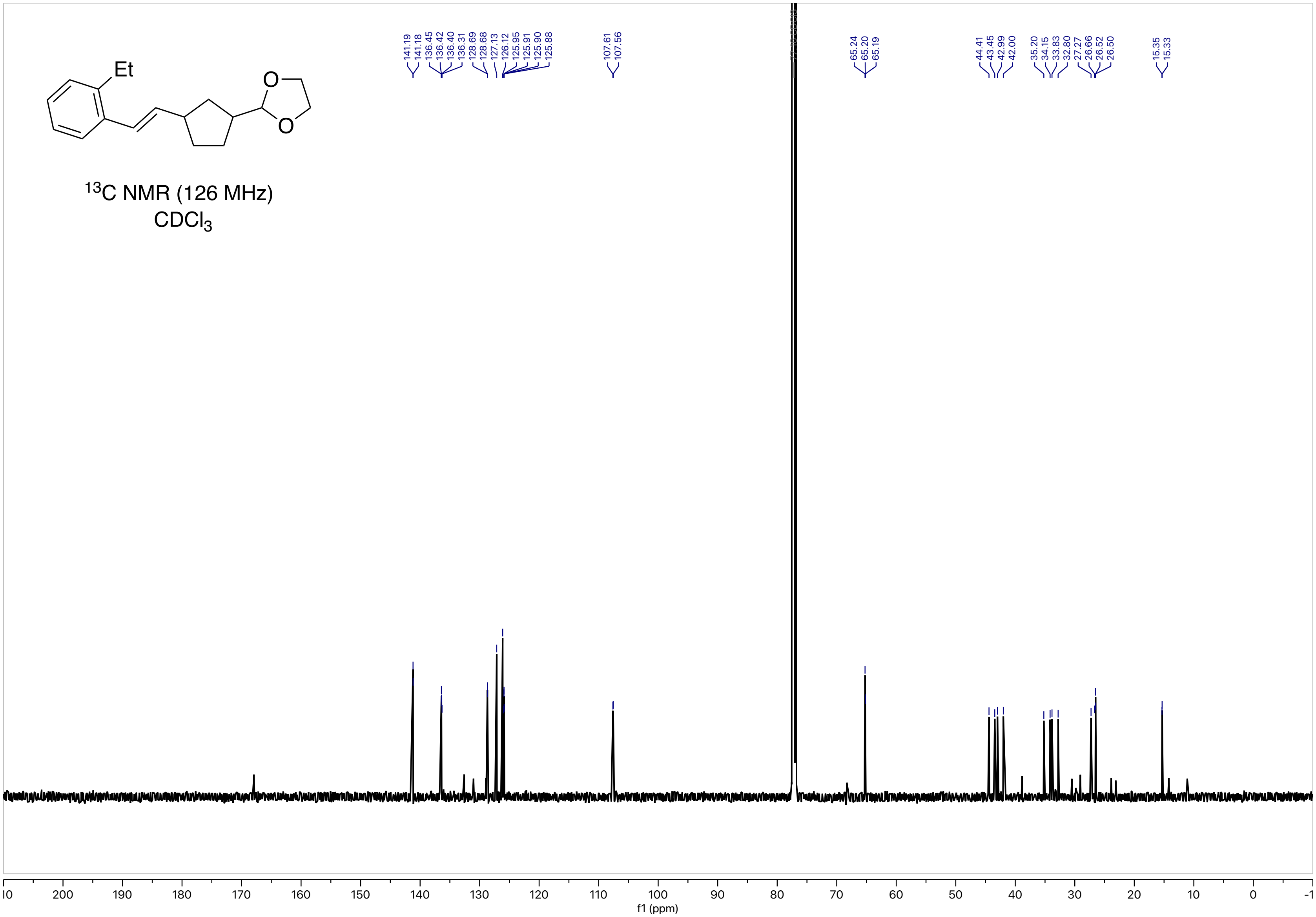
Et
O
O
^{13}C NMR (126 MHz)
CDCl$_3$
141.19
141.18
136.45
136.42
136.40
136.31
128.69
128.68
127.13
126.12
125.95
125.91
125.90
125.88
107.61
107.56
65.24
65.20
65.19
44.41
43.45
42.99
42.00
35.20
34.15
33.83
32.80
27.27
26.66
26.52
26.50
15.35
15.33
f1 (ppm)

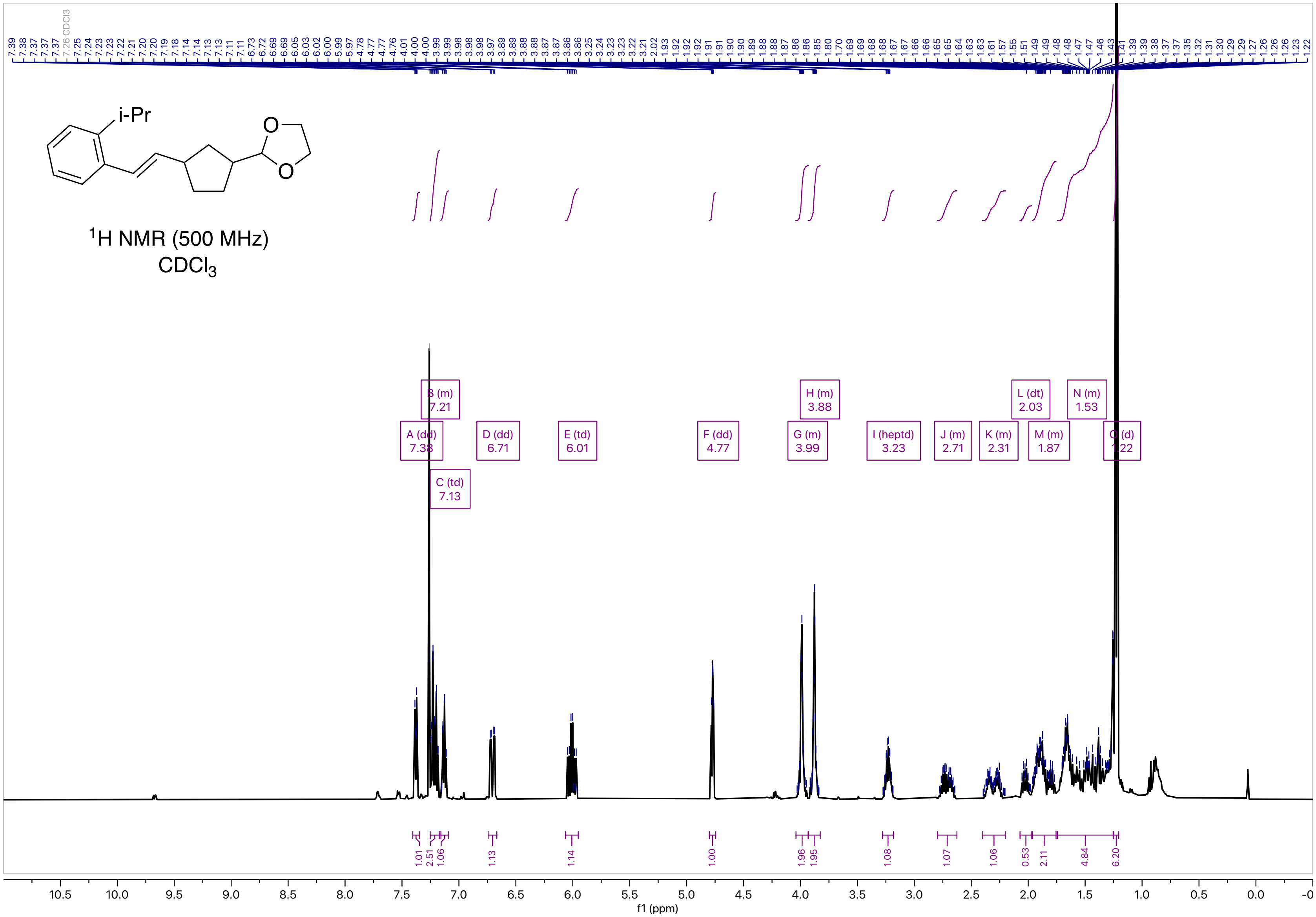
i-Pr
O
O
^{1}H NMR (500 MHz)
CDCl$_3$
A (dd) 7.38
B (m) 7.21
C (td) 7.13
D (dd) 6.71
E (td) 6.01
F (dd) 4.77
G (m) 3.99
H (m) 3.88
I (heptd) 3.23
J (m) 2.71
K (m) 2.31
L (dt) 2.03
M (m) 1.87
N (m) 1.53
O (d) 1.22
f1 (ppm)

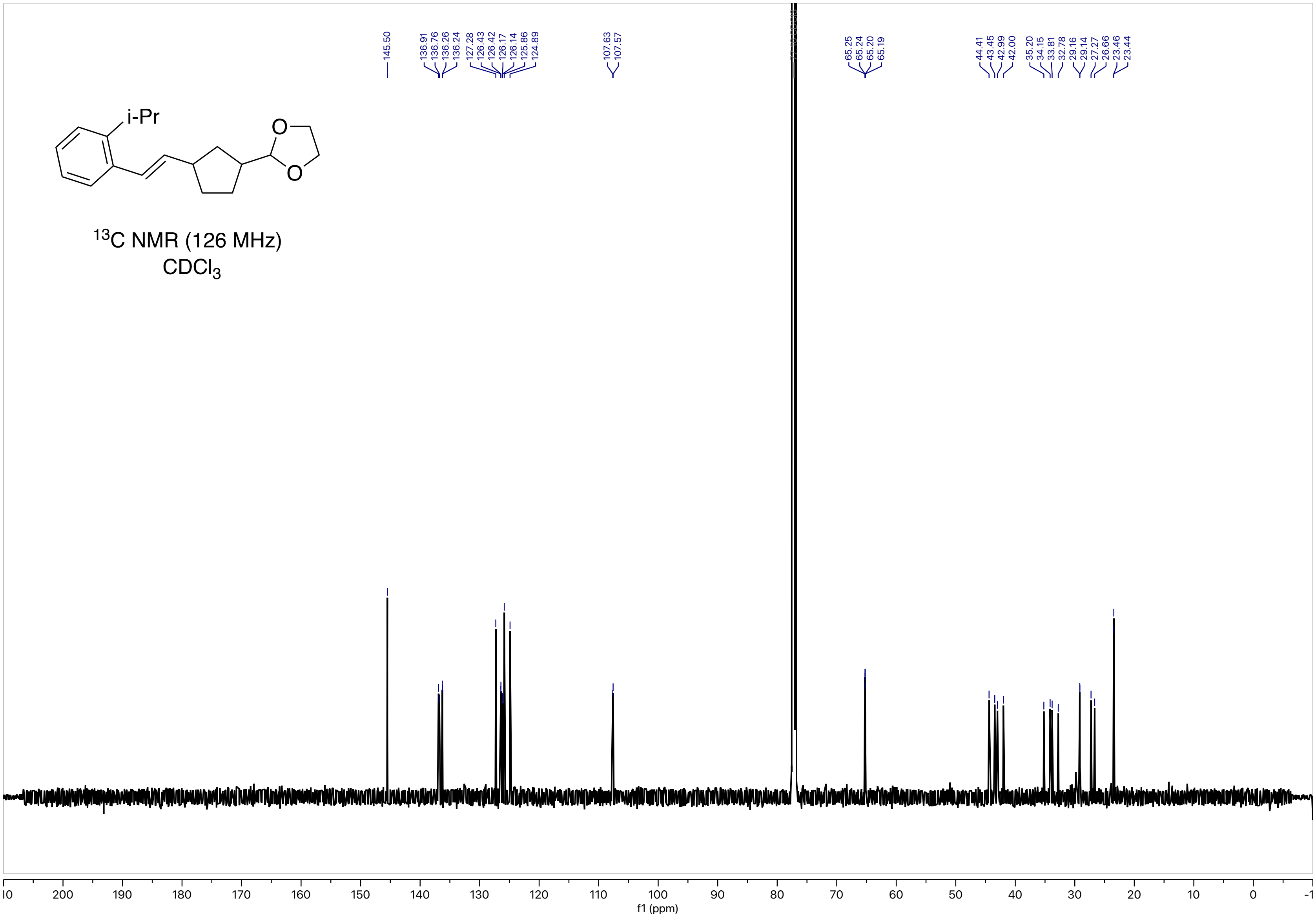

i-Pr
O
O
^{13}C NMR (126 MHz)
CDCl$_3$
145.50
136.91
136.76
136.26
136.24
127.28
126.43
126.42
126.17
126.14
125.86
124.89
107.63
107.57
65.25
65.24
65.20
65.19
44.41
43.45
42.99
42.00
35.20
34.15
33.81
32.78
29.16
29.14
27.27
26.66
23.46
23.44
f1 (ppm)

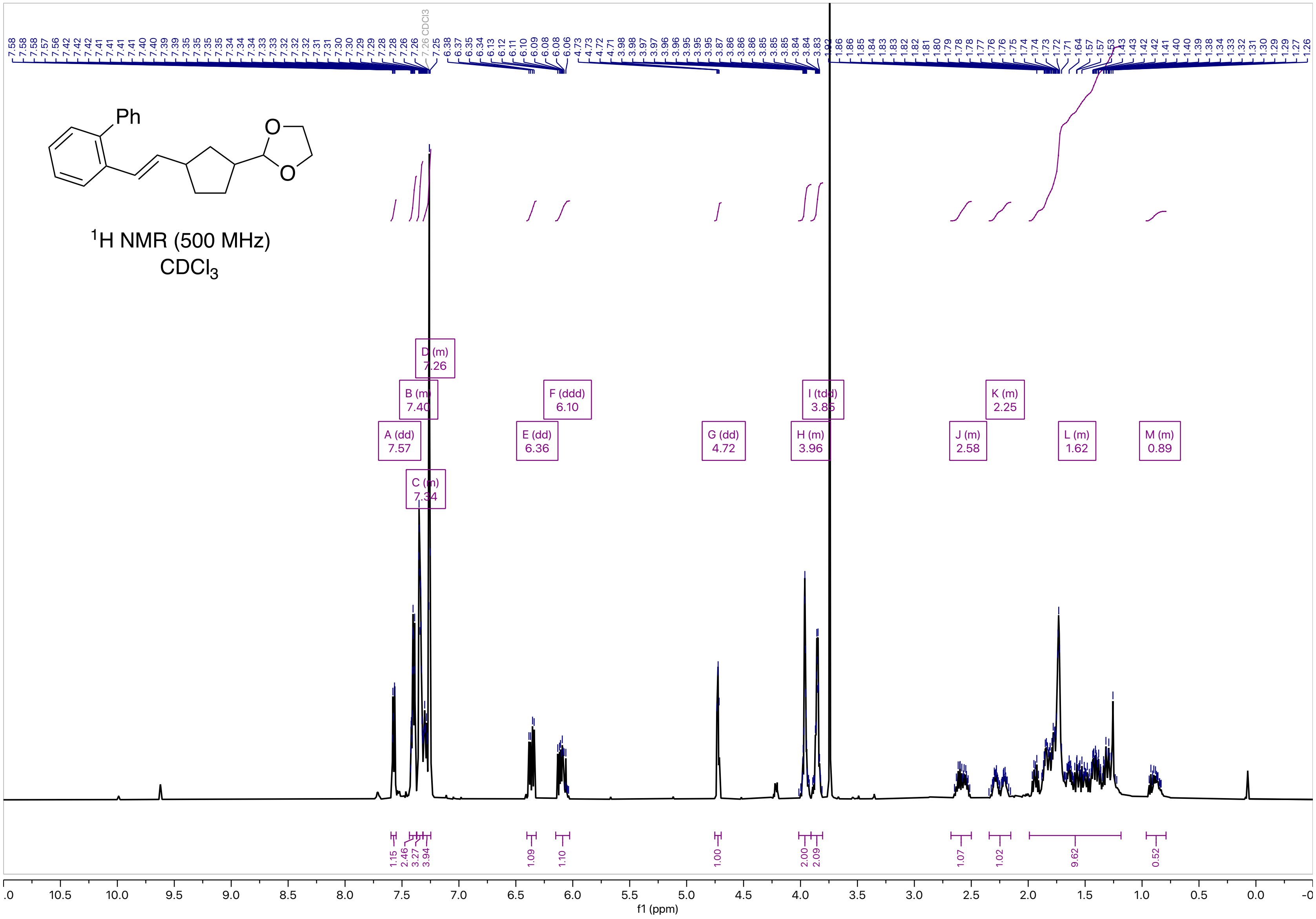
Ph
O
O
^{1}H NMR (500 MHz)
CDCl$_3$
A (dd) 7.57
B (m) 7.40
C (m) 7.34
D (m) 7.26
E (dd) 6.36
F (ddd) 6.10
G (dd) 4.72
H (m) 3.96
I (tdd) 3.85
J (m) 2.58
K (m) 2.25
L (m) 1.62
M (m) 0.89
1.15
2.46
3.27
3.94
1.09
1.10
1.00
2.00
2.09
1.07
1.02
9.62
0.52
f1 (ppm)

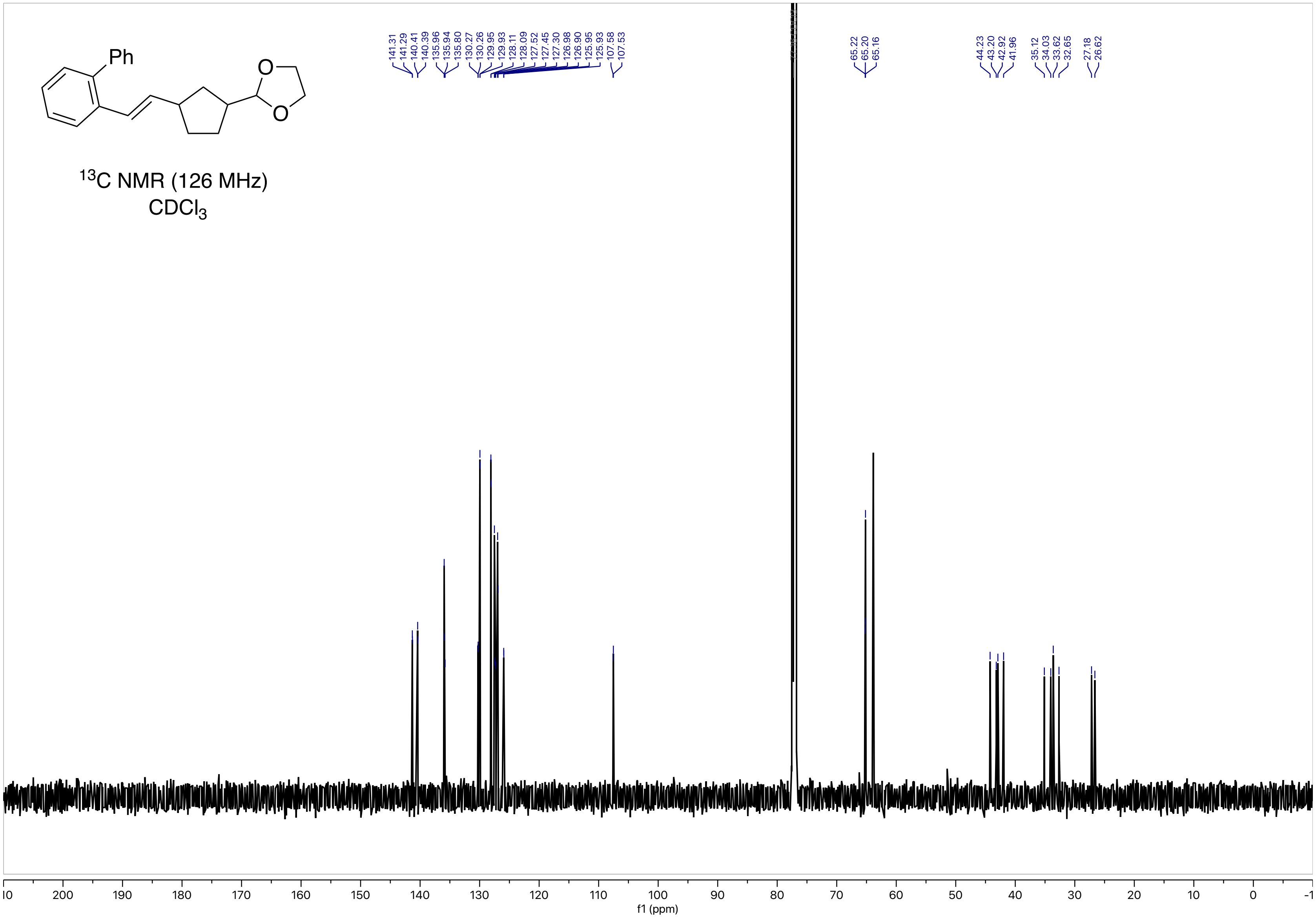

Ph
O
O
^{13}C NMR (126 MHz)
CDCl$_3$
141.31
141.29
140.41
140.39
135.96
135.94
135.80
130.27
130.26
129.95
129.93
128.11
128.09
127.52
127.45
127.30
126.98
126.90
125.95
125.93
107.58
107.53
65.22
65.20
65.16
44.23
43.20
42.92
41.96
35.12
34.03
33.62
32.65
27.18
26.62
f1 (ppm)

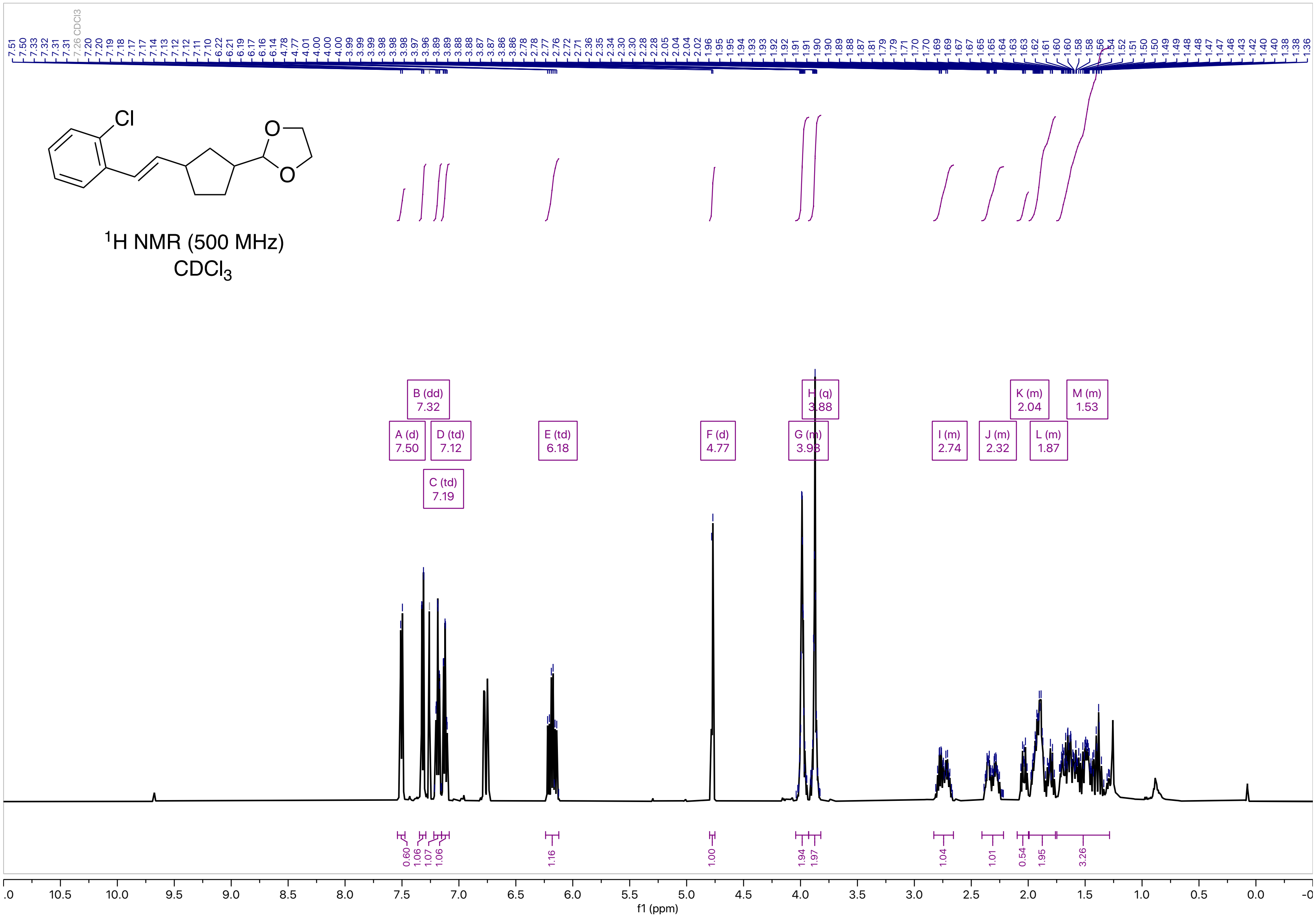

Cl
O
O
^{1}H NMR (500 MHz)
CDCl3
A (d) 7.50
B (dd) 7.32
C (td) 7.19
D (td) 7.12
E (td) 6.18
F (d) 4.77
G (m) 3.98
H (q) 3.88
I (m) 2.74
J (m) 2.32
K (m) 2.04
L (m) 1.87
M (m) 1.53
f1 (ppm)

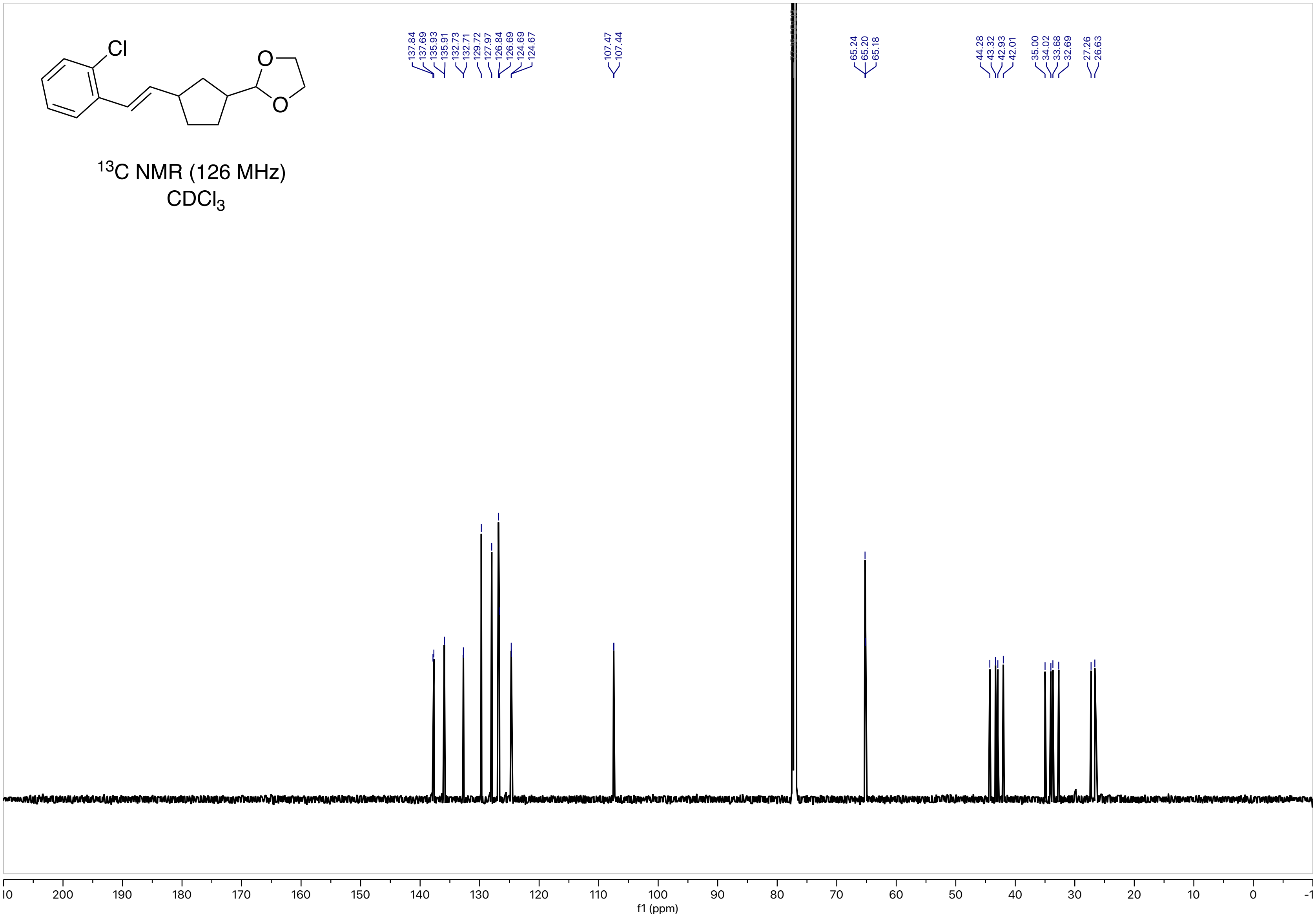
Cl
O
O
^{13}C NMR (126 MHz)
$CDCl_3$
137.84
137.69
135.93
135.91
132.73
132.71
129.72
127.97
126.84
126.69
124.69
124.67
107.47
107.44
65.24
65.20
65.18
44.28
43.32
42.93
42.01
35.00
34.02
33.68
32.69
27.26
26.63
f1 (ppm)

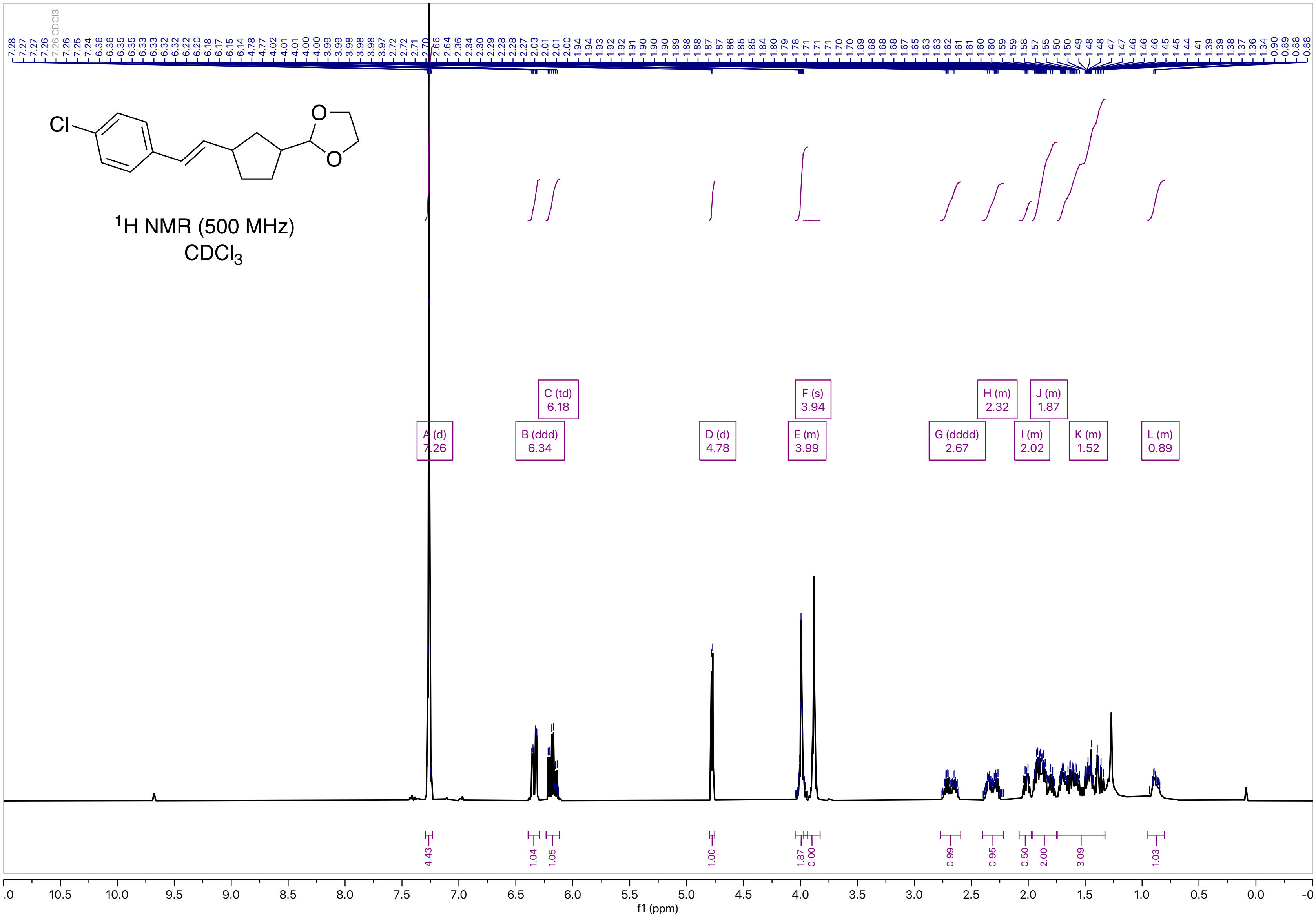

Cl
O
O
^{1}H NMR (500 MHz)
CDCl$_3$
A (d) 7.26
B (ddd) 6.34
C (td) 6.18
D (d) 4.78
E (m) 3.99
F (s) 3.94
G (dddd) 2.67
H (m) 2.32
I (m) 2.02
J (m) 1.87
K (m) 1.52
L (m) 0.89
f1 (ppm)

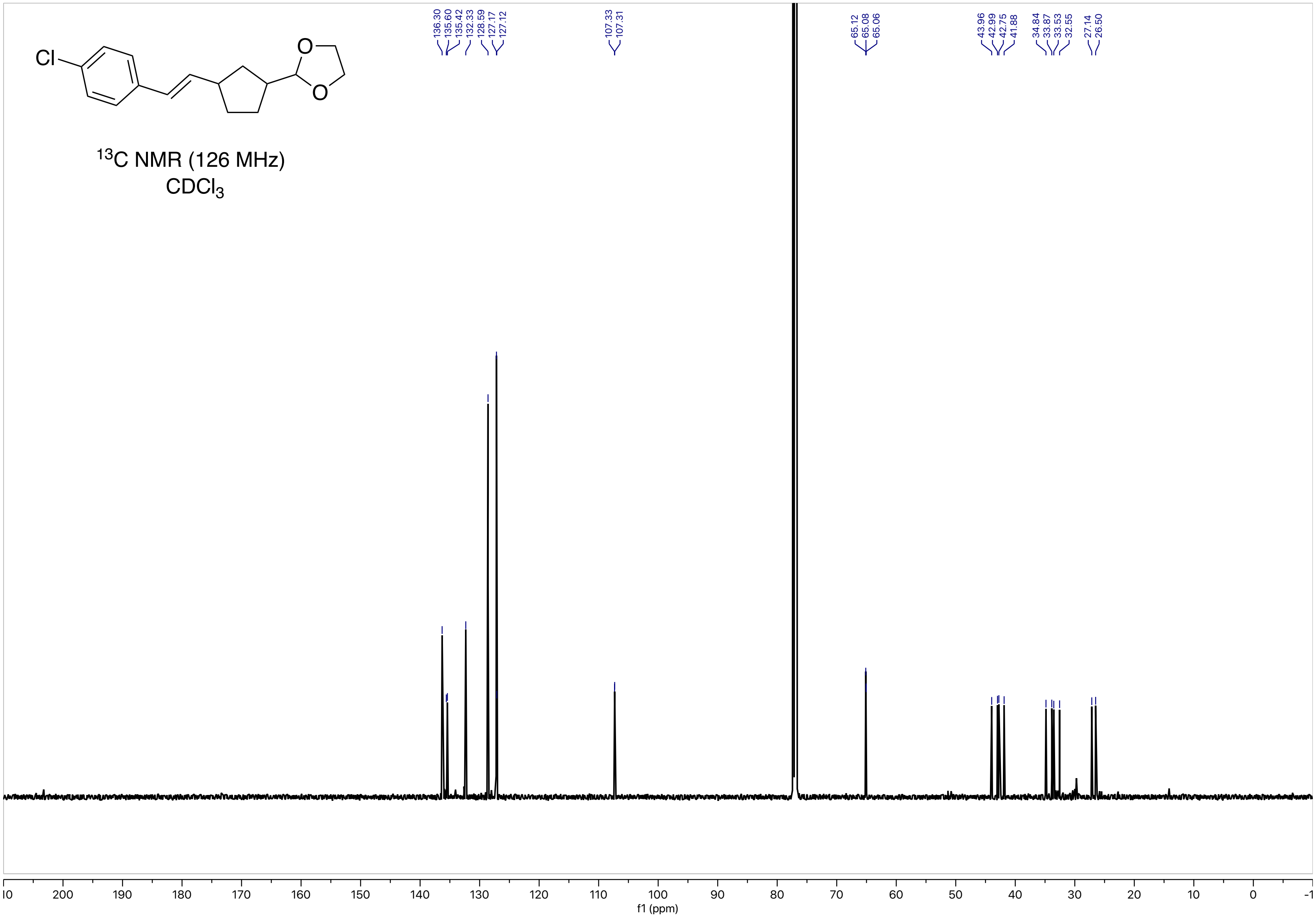
Cl
O
O
^{13}C NMR (126 MHz)
$CDCl_3$
136.30
135.60
135.42
132.33
128.59
127.17
127.12
107.33
107.31
65.12
65.08
65.06
43.96
42.99
42.75
41.88
34.84
33.87
33.53
32.55
27.14
26.50
200
190
180
170
160
150
140
130
120
110
100
90
80
70
60
50
40
30
20
10
0
f1 (ppm)

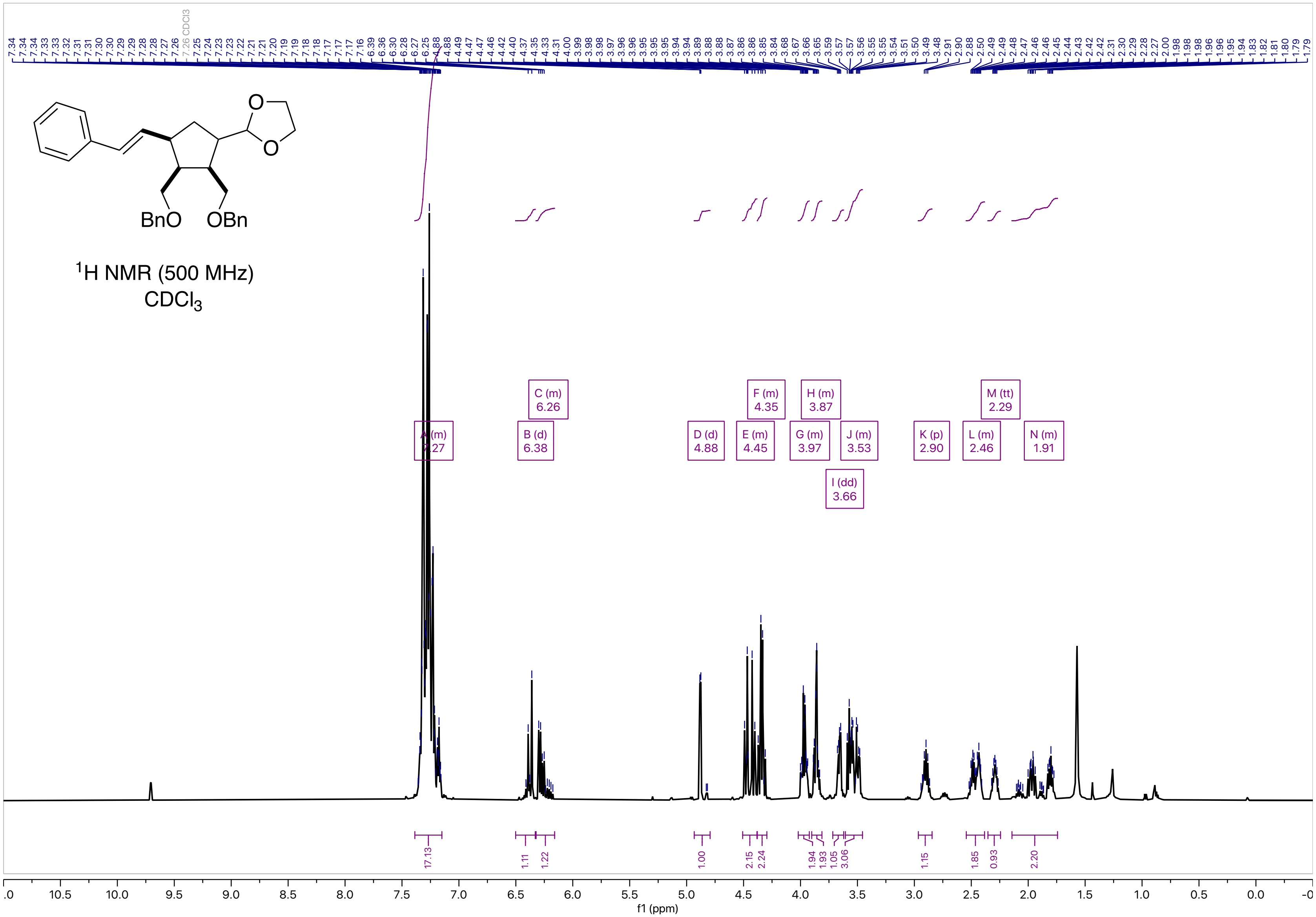
BnO
OBn
^{1}H NMR (500 MHz)
$CDCl_3$
A (m)
7.27
B (d)
6.38
C (m)
6.26
D (d)
4.88
E (m)
4.45
F (m)
4.35
G (m)
3.97
H (m)
3.87
I (dd)
3.66
J (m)
3.53
K (p)
2.90
L (m)
2.46
M (tt)
2.29
N (m)
1.91
f1 (ppm)

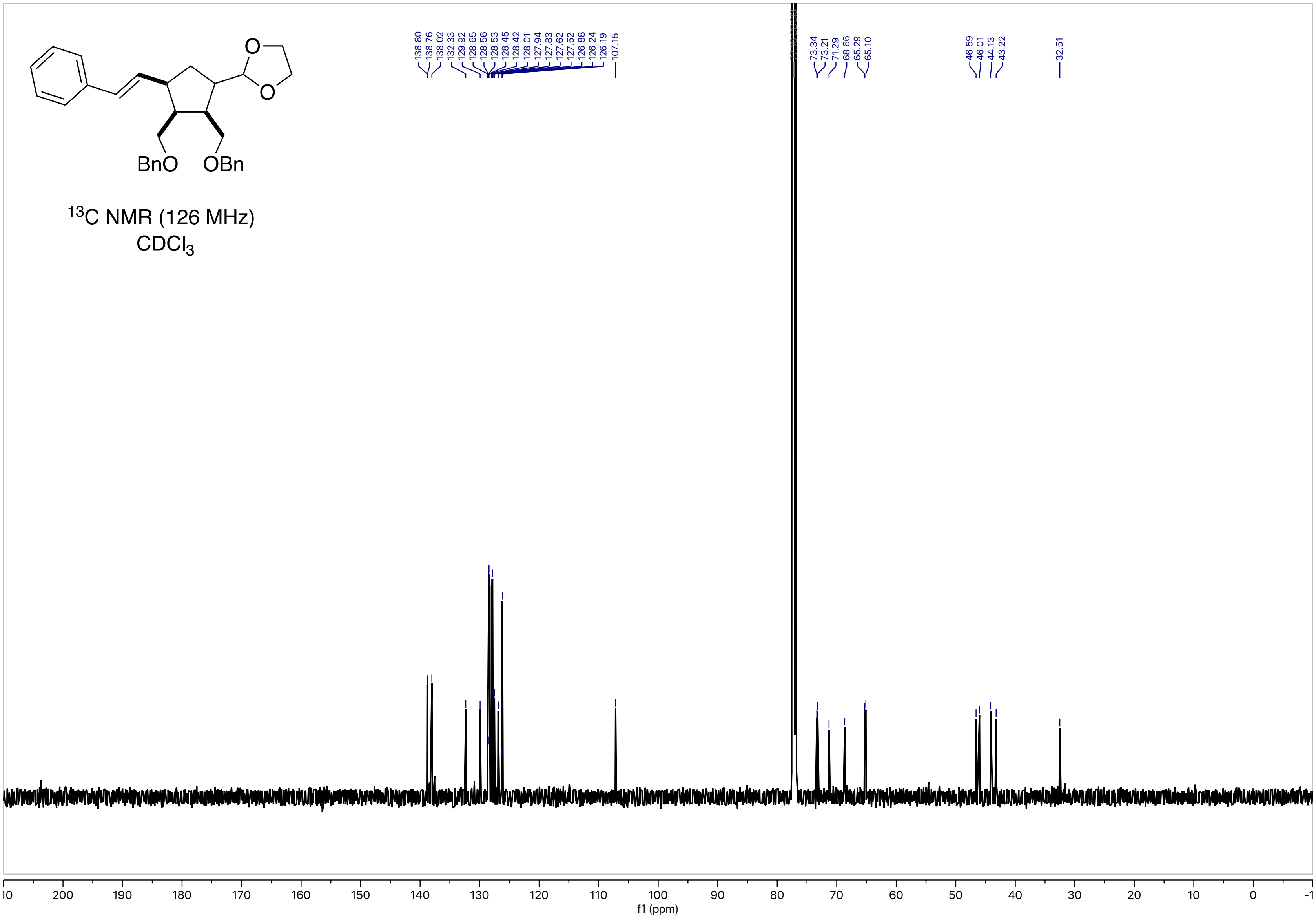
BnO
OBn
O
O
13C NMR (126 MHz)
CDCl3
138.80
138.76
138.02
132.33
129.92
128.65
128.56
128.53
128.45
128.42
128.01
127.94
127.83
127.62
127.52
126.88
126.24
126.19
107.15
73.34
73.21
71.29
68.66
65.29
65.10
46.59
46.01
44.13
43.22
32.51
f1 (ppm)

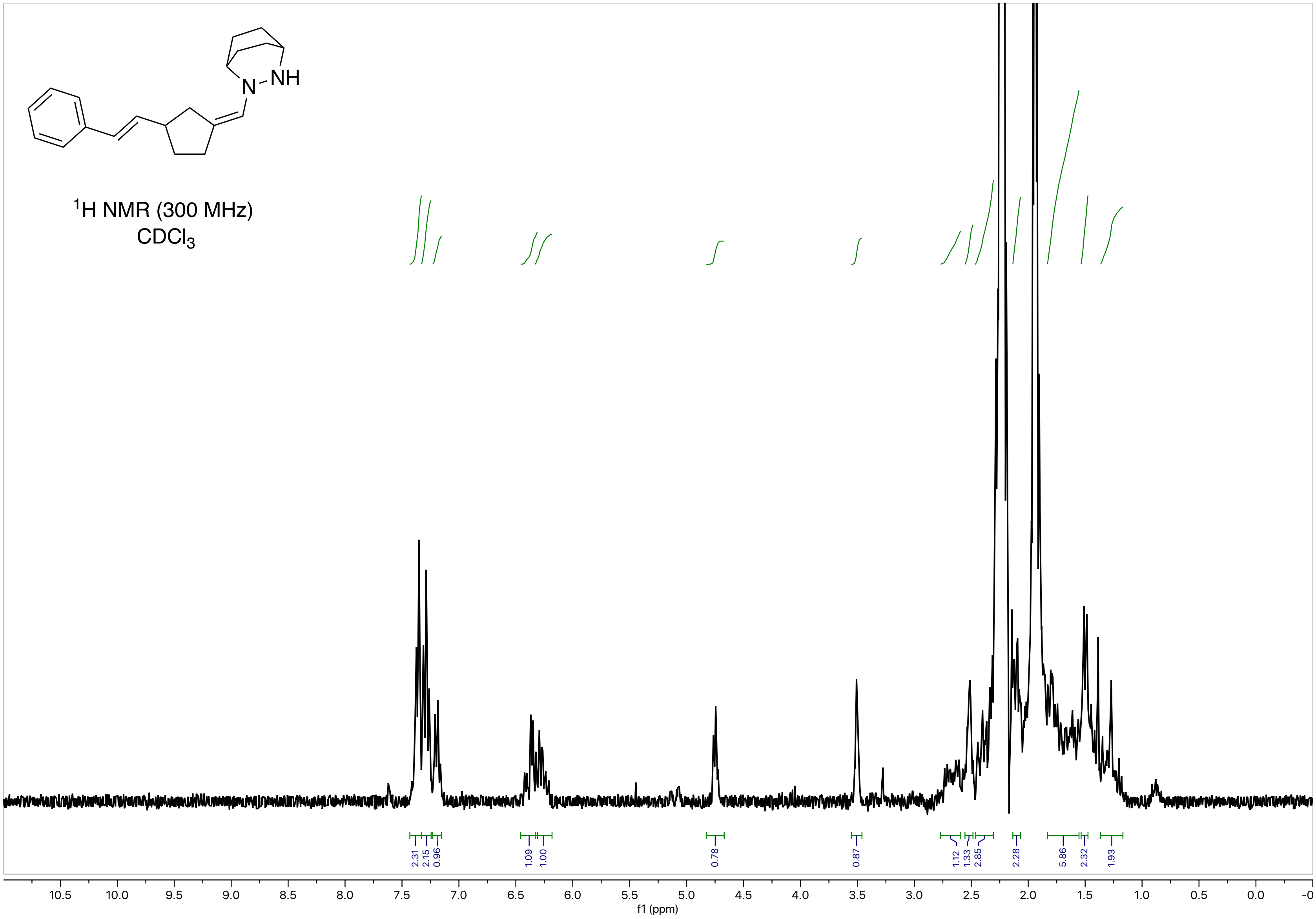

N
NH
^{1}H NMR (300 MHz)
$CDCl_3$
2.31
2.15
0.96
1.09
1.00
0.78
0.87
1.12
1.33
2.85
2.28
5.86
2.32
1.93
10.5
10.0
9.5
9.0
8.5
8.0
7.5
7.0
6.5
6.0
5.5
5.0
4.5
4.0
3.5
3.0
2.5
2.0
1.5
1.0
0.5
0.0
f1 (ppm)

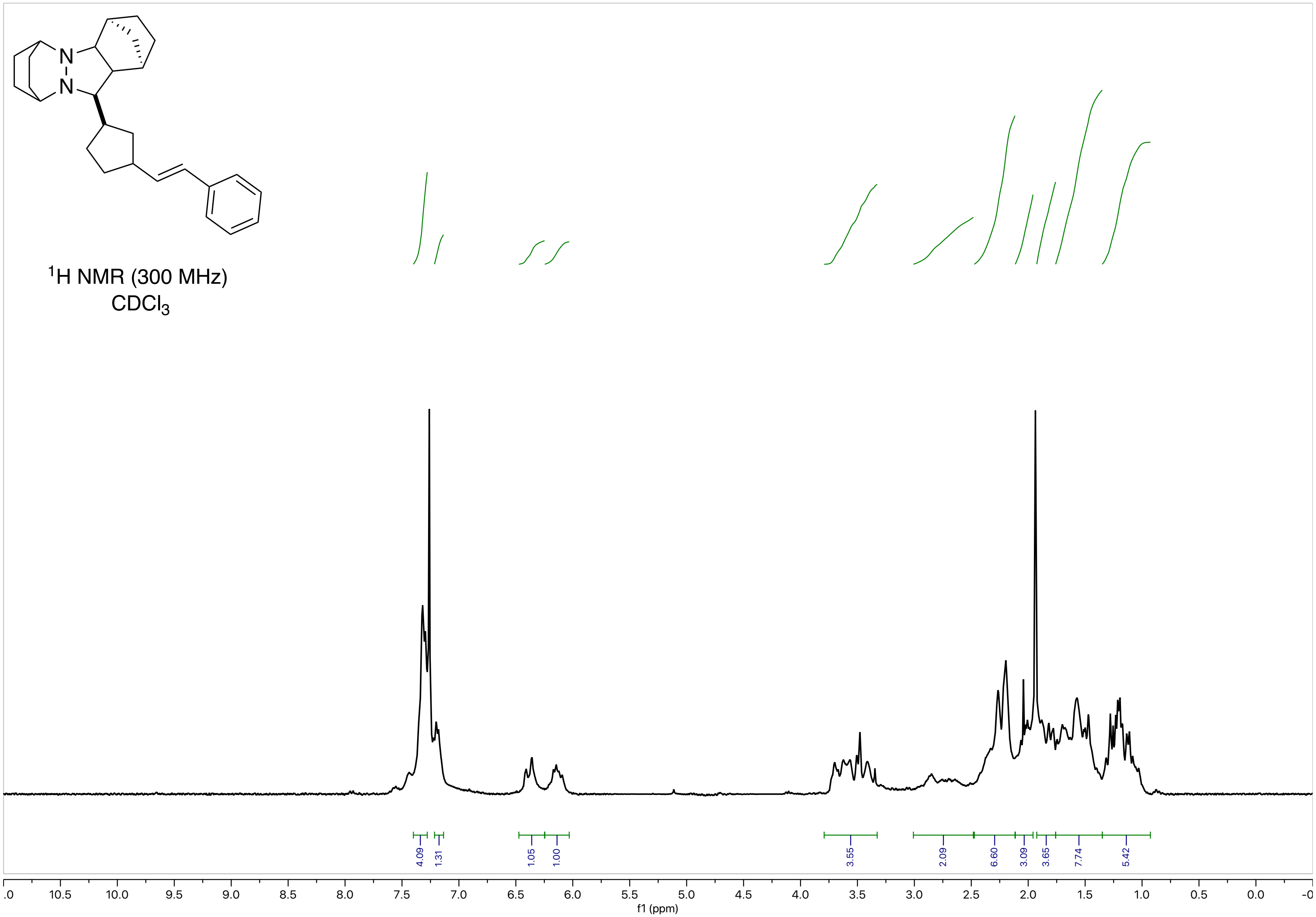

N
N
^{1}H NMR (300 MHz)
CDCl3
4.09
1.31
1.05
1.00
3.55
2.09
6.60
3.09
3.65
7.74
5.42
f1 (ppm)

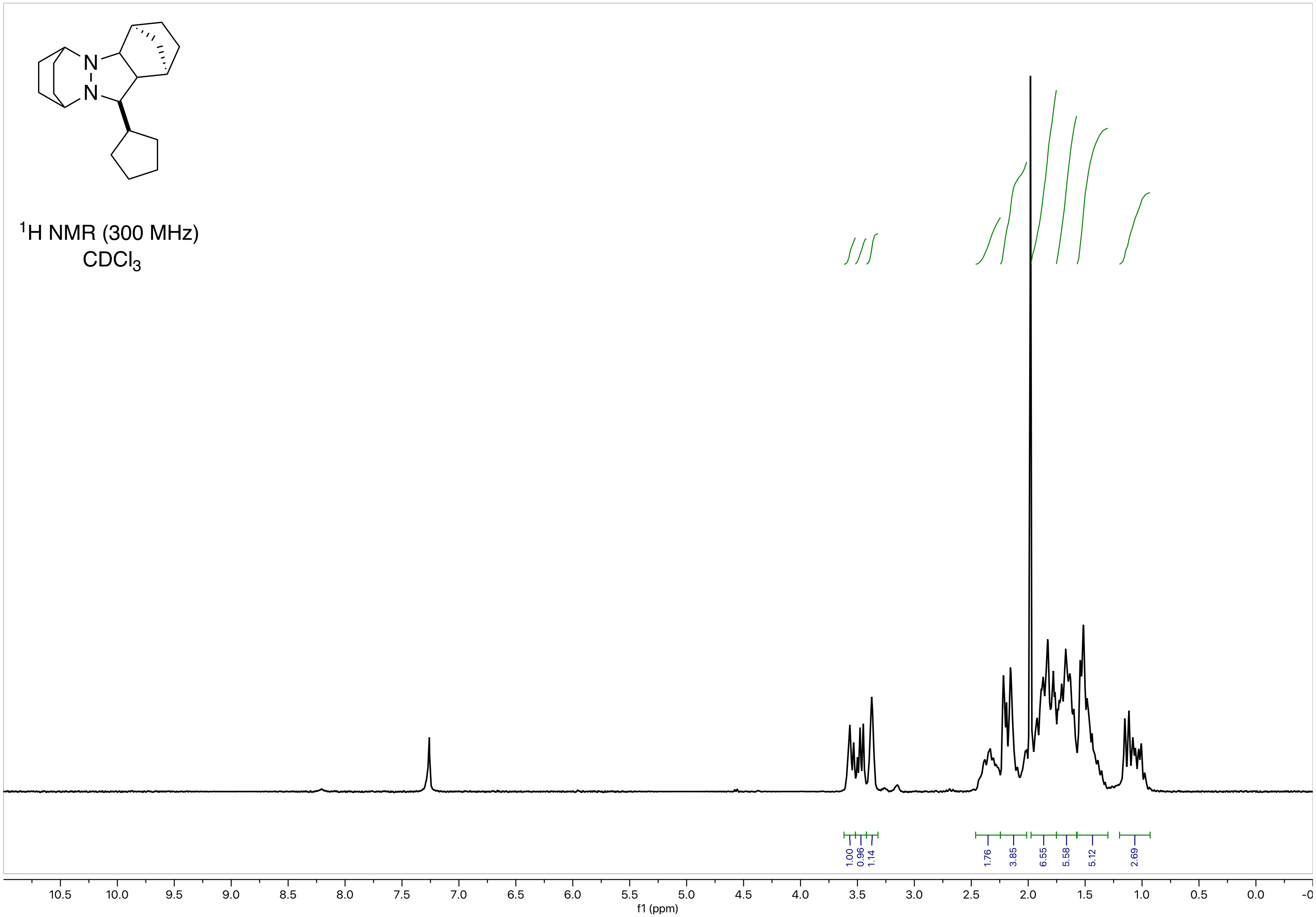

^{1}H NMR (300 MHz)
$CDCl_3$
1.00
0.96
1.14
1.76
3.85
6.55
5.58
5.12
2.69
10.5
10.0
9.5
9.0
8.5
8.0
7.5
7.0
6.5
6.0
5.5
5.0
4.5
4.0
3.5
3.0
2.5
2.0
1.5
1.0
0.5
0.0
f1 (ppm)

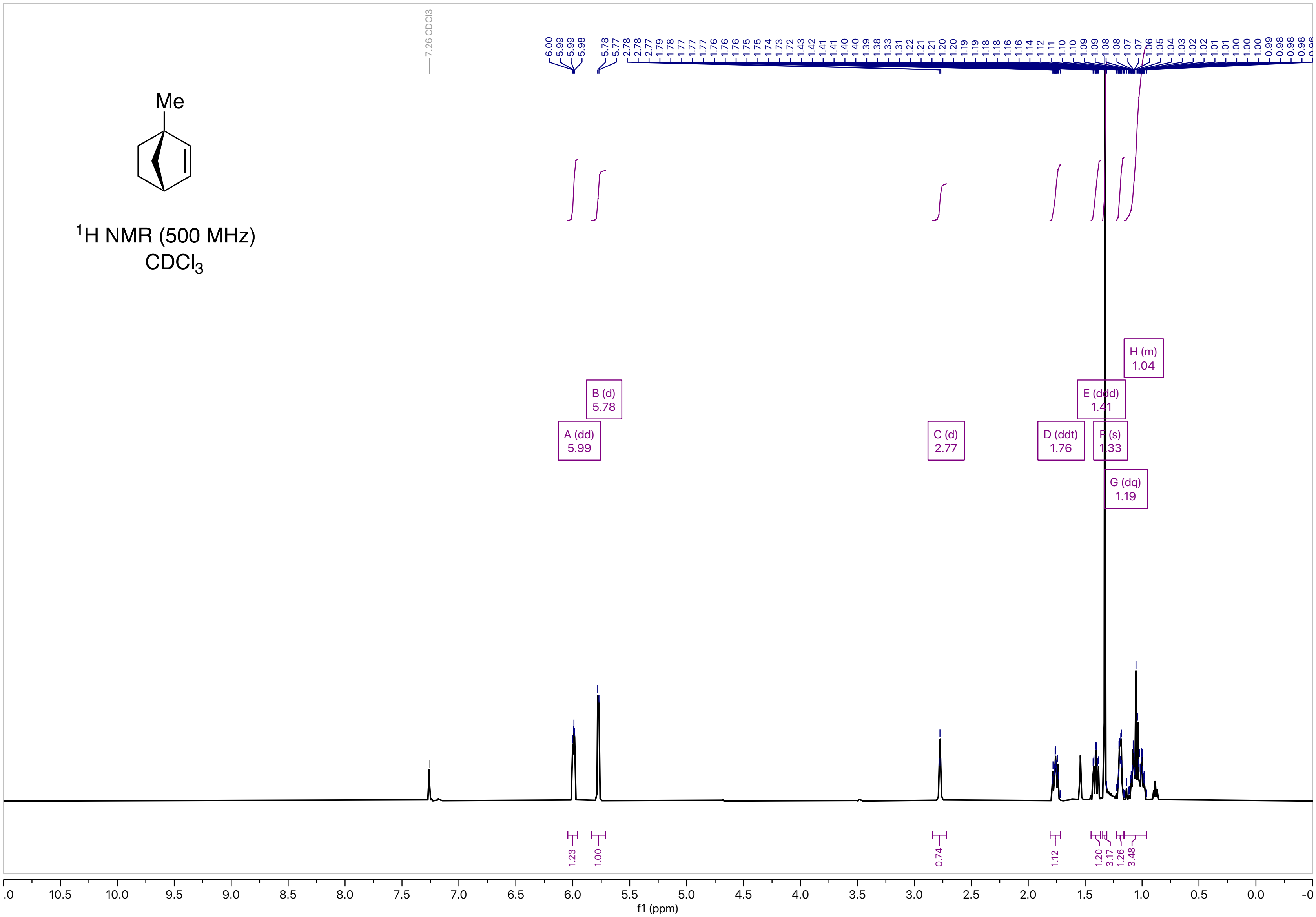
Me
1H NMR (500 MHz)
CDCl3
7.26 CDCl3
A (dd)
5.99
B (d)
5.78
C (d)
2.77
D (ddt)
1.76
E (ddd)
1.41
F (s)
1.33
G (dq)
1.19
H (m)
1.04
1.23
1.00
0.74
1.12
1.20
3.17
1.26
3.48
f1 (ppm)

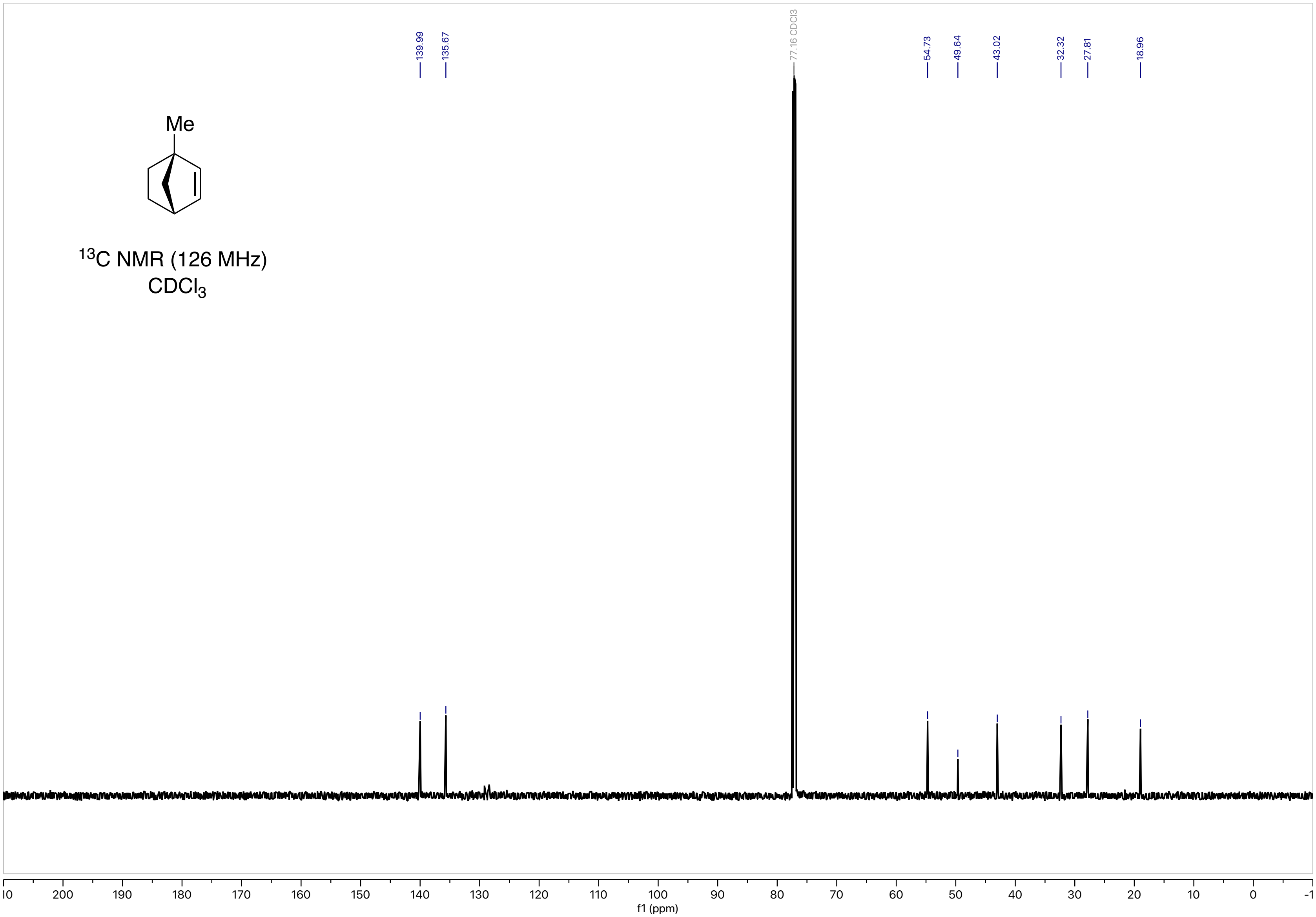
139.99
135.67
77.16 CDCl3
54.73
49.64
43.02
32.32
27.81
18.96
Me
^{13}C NMR (126 MHz)
$CDCl_3$
f1 (ppm)

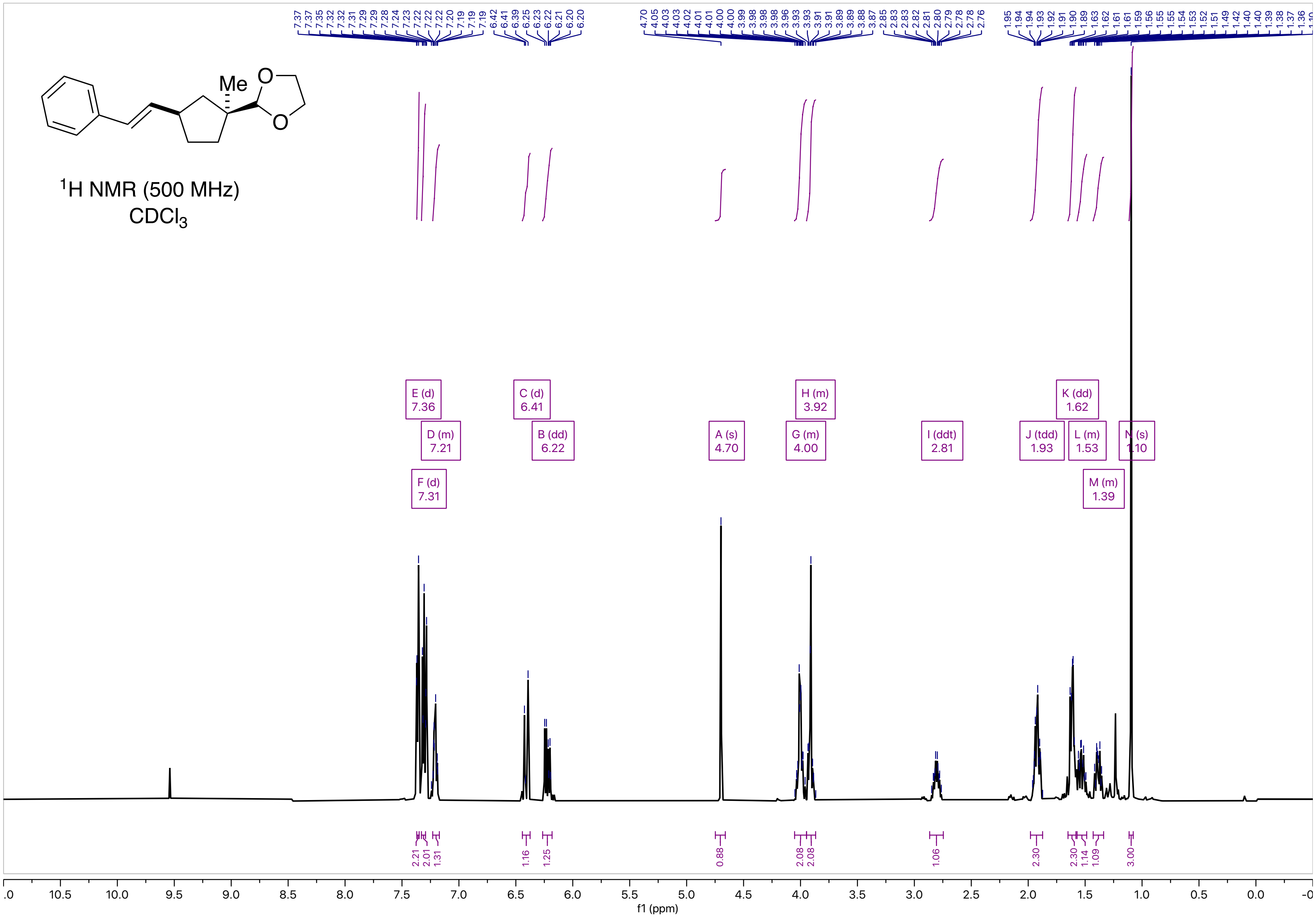
Me
O
O
¹H NMR (500 MHz)
CDCl3
E (d) 7.36
D (m) 7.21
F (d) 7.31
C (d) 6.41
B (dd) 6.22
A (s) 4.70
H (m) 3.92
G (m) 4.00
I (ddt) 2.81
J (tdd) 1.93
K (dd) 1.62
L (m) 1.53
M (m) 1.39
N (s) 1.10
2.21
2.01
1.31
1.16
1.25
0.88
2.08
2.08
1.06
2.30
2.30
1.14
1.09
3.00
f1 (ppm)

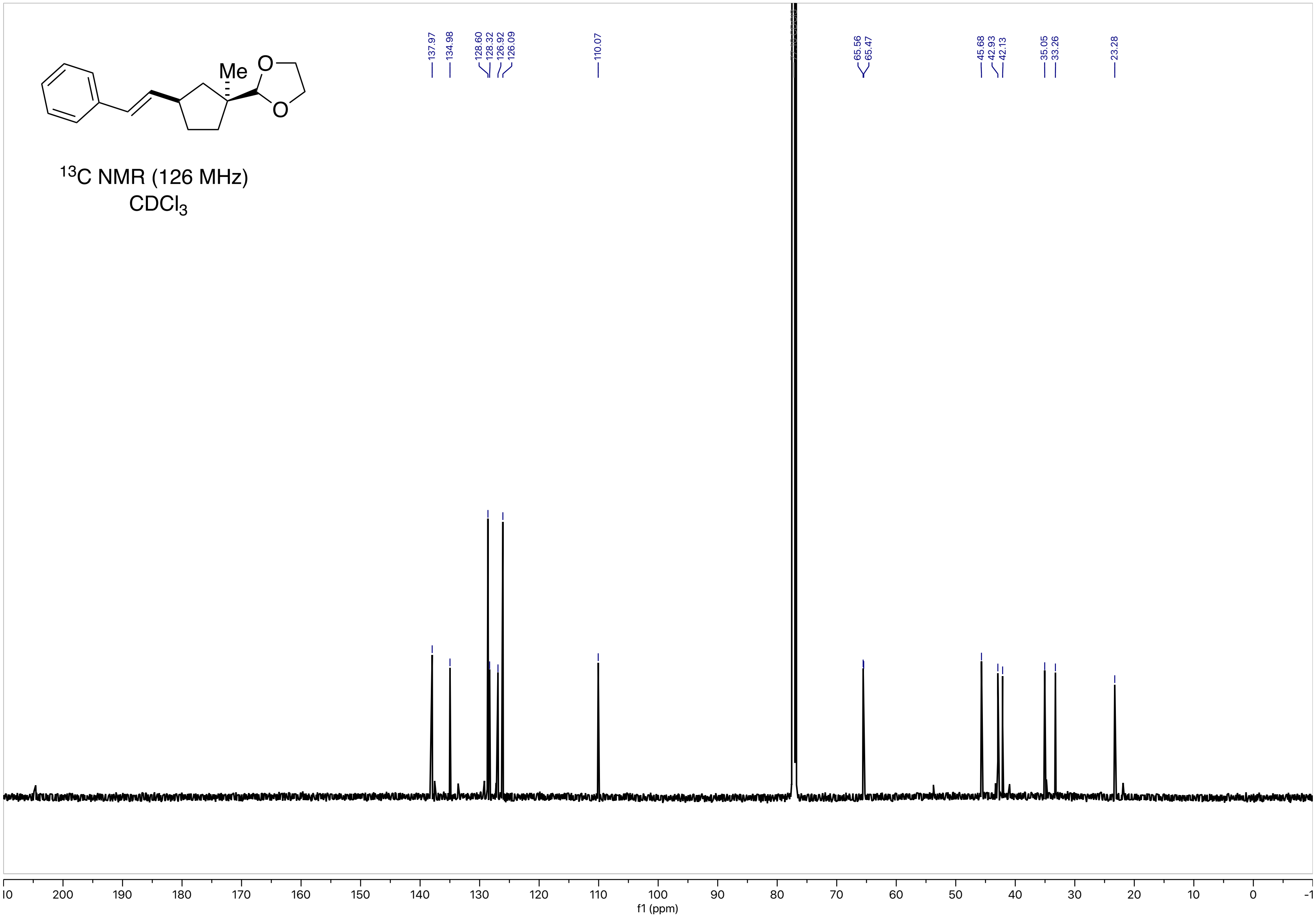

Me
O
O
^{13}C NMR (126 MHz)
CDCl$_3$
137.97
134.98
128.60
128.32
126.92
126.09
110.07
65.56
65.47
45.68
42.93
42.13
35.05
33.26
23.28
f1 (ppm)

X-ray Crystallography

Low-temperature X-ray diffraction data for **32** were collected on a Rigaku XtaLAB Synergy diffractometer coupled to a Rigaku Hypix detector with Cu Kα radiation (λ = 1.54184 Å), from a PhotonJet micro-focus X-ray source at 100 K. The diffraction images were processed and scaled using the CrysAlisPro software.[5)] The structures were solved through intrinsic phasing using SHELXT[6)] and refined against F^2 on all data by full-matrix least squares with SHELXL[7)] following established refinement strategies.[8)] All non-hydrogen atoms were refined anisotropically. All hydrogen atoms bound to carbon were included in the model at geometrically calculated positions and refined using a riding model. Hydrogen atoms bound to nitrogen were located in the difference Fourier synthesis and subsequently refined semi-freely with the help of distance restraints. The isotropic displacement parameters of all hydrogen atoms were fixed to 1.2 times the Ueq value of the atoms they are linked to (1.5 times for methyl groups). Details of the data quality and a summary of the residual values of the refinements are listed in Tables 1-6.

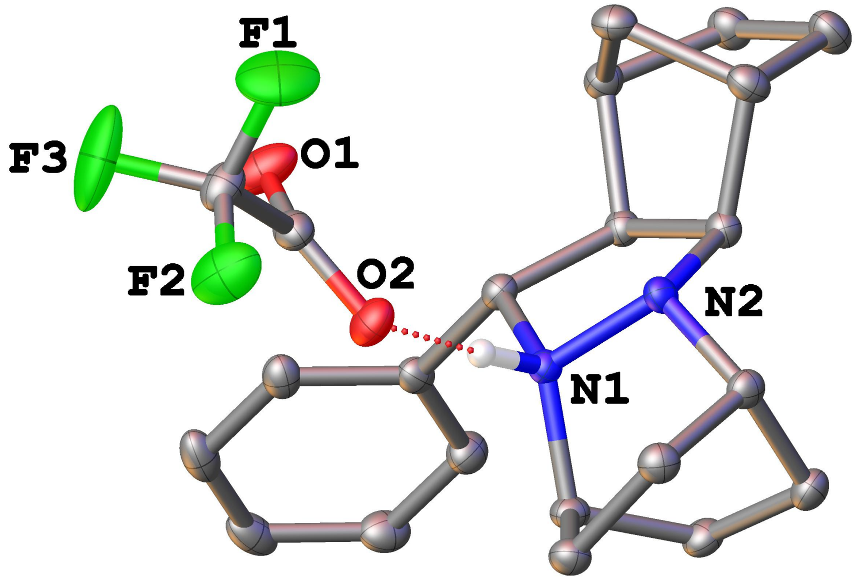

Table 1. Crystal data and structure refinement for **32**.

Identification code	rkpq3_abs	
Empirical formula	C22 H27 F3 N2 O2	
Formula weight	408.45	
Temperature	100.00(10) K	
Wavelength	1.54184 Å	
Crystal system	Monoclinic	
Space group	P 1 21/n 1	
Unit cell dimensions	a = 11.36910(10) Å	a= 90°.
	b = 13.80880(10) Å	b= 95.4170(10)°.
	c = 12.46330(10) Å	g = 90°.
Volume	1947.92(3) $Å^3$	
Z	4	
Density (calculated)	1.393 Mg/m^3	
Absorption coefficient	0.912 mm^{-1}	
F(000)	864	
Crystal size	0.159 x 0.124 x 0.102 mm^3	
Theta range for data collection	4.791 to 78.042°.	
Index ranges	-14<=h<=13, -17<=k<=17, -15<=l<=15	
Reflections collected	87403	
Independent reflections	4166 [R(int) = 0.0312]	
Completeness to theta = 67.684°	100.0 %	
Absorption correction	Gaussian	
Max. and min. transmission	1.000 and 0.657	

Refinement method	Full-matrix least-squares on F^2
Data / restraints / parameters	4166 / 1 / 265
Goodness-of-fit on F^2	1.034
Final R indices [I>2sigma(I)]	R1 = 0.0386, wR2 = 0.0988
R indices (all data)	R1 = 0.0396, wR2 = 0.0996
Extinction coefficient	n/a
Largest diff. peak and hole	0.340 and -0.398 e.Å^{-3}

Table 2. Atomic coordinates (x 10^4) and equivalent isotropic displacement parameters ($Å^2$x 10^3) for **32**. U(eq) is defined as one third of the trace of the orthogonalized U^{ij} tensor.

	x	y	z	U(eq)
F(1)	7064(1)	2225(1)	5909(1)	51(1)
F(2)	8722(1)	2702(1)	6648(1)	42(1)
F(3)	8654(1)	1670(1)	5367(1)	62(1)
O(1)	7868(1)	3022(1)	3911(1)	35(1)
O(2)	8317(1)	4110(1)	5230(1)	24(1)
C(21)	8106(1)	3286(1)	4851(1)	20(1)
C(22)	8153(1)	2468(1)	5703(1)	24(1)
N(1)	7724(1)	5628(1)	3963(1)	14(1)
N(2)	6552(1)	5887(1)	4305(1)	15(1)
C(1)	8623(1)	6423(1)	4225(1)	17(1)
C(2)	8895(1)	6366(1)	5453(1)	19(1)
C(3)	7709(1)	6325(1)	5972(1)	19(1)
C(4)	6726(1)	6662(1)	5134(1)	17(1)
C(5)	7110(1)	7630(1)	4658(1)	20(1)
C(6)	8075(1)	7401(1)	3901(1)	19(1)
C(7)	7480(1)	5249(1)	2812(1)	15(1)
C(8)	6357(1)	5767(1)	2342(1)	15(1)
C(9)	5421(1)	5059(1)	1819(1)	18(1)
C(10)	4368(1)	5662(1)	1320(1)	21(1)
C(11)	3799(1)	6050(1)	2328(1)	23(1)
C(12)	4584(1)	5608(1)	3277(1)	20(1)
C(13)	5768(1)	6154(1)	3332(1)	16(1)
C(14)	4909(1)	4623(1)	2813(1)	20(1)
C(15)	8567(1)	5290(1)	2205(1)	16(1)
C(16)	8860(1)	6099(1)	1612(1)	21(1)
C(17)	9903(1)	6104(1)	1110(1)	24(1)
C(18)	10652(1)	5312(1)	1188(1)	25(1)
C(19)	10359(1)	4504(1)	1765(1)	25(1)
C(20)	9318(1)	4492(1)	2271(1)	19(1)

Table 3. Bond lengths [Å] and angles [°] for **32**.

F(1)-C(22)	1.3308(16)
F(2)-C(22)	1.3288(16)
F(3)-C(22)	1.3272(15)
O(1)-C(21)	1.2323(16)
O(2)-C(21)	1.2456(15)
C(21)-C(22)	1.5484(16)
N(1)-N(2)	1.4808(12)
N(1)-C(1)	1.5145(13)
N(1)-C(7)	1.5276(13)
N(2)-C(4)	1.4872(14)
N(2)-C(13)	1.4817(14)
C(1)-C(2)	1.5339(15)
C(1)-C(6)	1.5256(15)
C(2)-C(3)	1.5502(15)
C(3)-C(4)	1.5283(15)
C(4)-C(5)	1.5415(15)
C(5)-C(6)	1.5452(16)
C(7)-C(8)	1.5305(14)
C(7)-C(15)	1.5104(15)
C(8)-C(9)	1.5440(15)
C(8)-C(13)	1.5531(15)
C(9)-C(10)	1.5404(16)
C(9)-C(14)	1.5399(15)
C(10)-C(11)	1.5605(16)
C(11)-C(12)	1.5387(16)
C(12)-C(13)	1.5388(15)
C(12)-C(14)	1.5363(16)
C(15)-C(16)	1.3960(16)
C(15)-C(20)	1.3925(16)
C(16)-C(17)	1.3928(16)
C(17)-C(18)	1.3838(19)
C(18)-C(19)	1.3845(19)
C(19)-C(20)	1.3927(16)

O(1)-C(21)-O(2)	130.37(11)
O(1)-C(21)-C(22)	115.24(11)
O(2)-C(21)-C(22)	114.39(10)
F(1)-C(22)-C(21)	110.14(11)
F(2)-C(22)-F(1)	105.97(11)
F(2)-C(22)-C(21)	114.37(10)
F(3)-C(22)-F(1)	106.62(12)
F(3)-C(22)-F(2)	107.09(12)
F(3)-C(22)-C(21)	112.18(10)
N(2)-N(1)-C(1)	111.68(8)
N(2)-N(1)-C(7)	105.33(8)
C(1)-N(1)-C(7)	120.66(8)
N(1)-N(2)-C(4)	108.03(8)
N(1)-N(2)-C(13)	108.23(8)
C(13)-N(2)-C(4)	114.84(9)
N(1)-C(1)-C(2)	104.31(8)
N(1)-C(1)-C(6)	109.51(9)
C(6)-C(1)-C(2)	110.34(9)
C(1)-C(2)-C(3)	108.44(9)
C(4)-C(3)-C(2)	108.19(9)
N(2)-C(4)-C(3)	107.21(9)
N(2)-C(4)-C(5)	112.37(9)
C(3)-C(4)-C(5)	108.18(9)
C(4)-C(5)-C(6)	107.46(9)
C(1)-C(6)-C(5)	108.39(9)
N(1)-C(7)-C(8)	105.70(8)
C(15)-C(7)-N(1)	111.87(8)
C(15)-C(7)-C(8)	118.94(9)
C(7)-C(8)-C(9)	112.47(9)
C(7)-C(8)-C(13)	105.26(8)
C(9)-C(8)-C(13)	102.80(8)
C(10)-C(9)-C(8)	107.82(9)
C(14)-C(9)-C(8)	101.86(9)
C(14)-C(9)-C(10)	101.38(9)
C(9)-C(10)-C(11)	103.06(9)
C(12)-C(11)-C(10)	103.23(9)

C(11)-C(12)-C(13)	106.34(9)
C(14)-C(12)-C(11)	101.76(9)
C(14)-C(12)-C(13)	102.09(9)
N(2)-C(13)-C(8)	107.08(8)
N(2)-C(13)-C(12)	111.79(9)
C(12)-C(13)-C(8)	103.74(9)
C(12)-C(14)-C(9)	94.60(9)
C(16)-C(15)-C(7)	122.81(10)
C(20)-C(15)-C(7)	117.85(10)
C(20)-C(15)-C(16)	119.32(10)
C(17)-C(16)-C(15)	119.84(11)
C(18)-C(17)-C(16)	120.56(12)
C(17)-C(18)-C(19)	119.80(11)
C(18)-C(19)-C(20)	120.11(11)
C(15)-C(20)-C(19)	120.36(11)

Symmetry transformations used to generate equivalent atoms:

Table 4. Anisotropic displacement parameters ($Å^2 x 10^3$) for **32**. The anisotropic displacement factor exponent takes the form: $-2p^2[h^2 a^{*2}U^{11} + ... + 2 h k a^* b^* U^{12}]$

	U^{11}	U^{22}	U^{33}	U^{23}	U^{13}	U^{12}
F(1)	52(1)	56(1)	47(1)	12(1)	17(1)	-26(1)
F(2)	62(1)	33(1)	28(1)	11(1)	-7(1)	-6(1)
F(3)	119(1)	31(1)	38(1)	10(1)	23(1)	39(1)
O(1)	57(1)	23(1)	24(1)	3(1)	-4(1)	-12(1)
O(2)	32(1)	16(1)	24(1)	2(1)	1(1)	1(1)
C(21)	19(1)	18(1)	23(1)	4(1)	3(1)	-1(1)
C(22)	33(1)	18(1)	24(1)	2(1)	9(1)	0(1)
N(1)	14(1)	14(1)	16(1)	0(1)	3(1)	0(1)
N(2)	13(1)	17(1)	16(1)	-1(1)	3(1)	1(1)
C(1)	15(1)	16(1)	19(1)	-1(1)	3(1)	-3(1)
C(2)	17(1)	20(1)	19(1)	-1(1)	0(1)	-2(1)
C(3)	20(1)	20(1)	16(1)	-1(1)	2(1)	-1(1)
C(4)	19(1)	17(1)	16(1)	-3(1)	3(1)	0(1)
C(5)	23(1)	15(1)	21(1)	-2(1)	3(1)	1(1)
C(6)	22(1)	15(1)	21(1)	1(1)	4(1)	-3(1)
C(7)	17(1)	14(1)	15(1)	0(1)	2(1)	0(1)
C(8)	16(1)	14(1)	16(1)	1(1)	3(1)	1(1)
C(9)	19(1)	17(1)	18(1)	-1(1)	1(1)	0(1)
C(10)	19(1)	25(1)	20(1)	0(1)	-1(1)	1(1)
C(11)	16(1)	28(1)	24(1)	-3(1)	-1(1)	3(1)
C(12)	15(1)	25(1)	19(1)	-1(1)	3(1)	-2(1)
C(13)	16(1)	16(1)	17(1)	0(1)	2(1)	1(1)
C(14)	18(1)	20(1)	22(1)	1(1)	1(1)	-5(1)
C(15)	16(1)	18(1)	15(1)	-3(1)	2(1)	0(1)
C(16)	22(1)	20(1)	22(1)	-1(1)	6(1)	-1(1)
C(17)	24(1)	28(1)	23(1)	-2(1)	8(1)	-6(1)
C(18)	16(1)	41(1)	19(1)	-8(1)	4(1)	-2(1)
C(19)	20(1)	34(1)	20(1)	-5(1)	0(1)	9(1)
C(20)	20(1)	21(1)	17(1)	-2(1)	0(1)	3(1)

Table 5. Hydrogen coordinates (x 10^4) and isotropic displacement parameters (Å^{2}x 10 ^{3}) for **32**.

	x	y	z	U(eq)
H(1)	7965(12)	5099(10)	4358(11)	17
H(1A)	9352	6306	3854	20
H(2A)	9353	6942	5718	22
H(2B)	9370	5781	5650	22
H(3A)	7552	5654	6201	23
H(3B)	7745	6750	6613	23
H(4)	5980	6758	5485	21
H(5A)	7426	8070	5242	23
H(5B)	6427	7948	4250	23
H(6A)	7723	7378	3144	23
H(6B)	8689	7911	3963	23
H(7)	7271	4549	2871	18
H(8)	6534	6300	1839	18
H(9)	5730	4572	1322	21
H(10A)	3804	5255	865	26
H(10B)	4637	6201	881	26
H(11A)	3819	6766	2355	28
H(11B)	2971	5828	2328	28
H(12)	4212	5575	3973	24
H(13)	5643	6870	3286	19
H(14A)	4211	4208	2622	24
H(14B)	5506	4268	3291	24
H(16)	8348	6644	1552	25
H(17)	10103	6657	710	29
H(18)	11364	5323	847	30
H(19)	10869	3958	1815	30
H(20)	9120	3935	2664	23

Table 6. Hydrogen bonds for **32** [Å and °].

D-H...A	d(D-H)	d(H...A)	d(D...A)	<(DHA)
N(1)-H(1)...O(2)	0.908(12)	1.768(12)	2.6721(12)	173.5(13)

Symmetry transformations used to generate equivalent atoms:

Kinetic Data

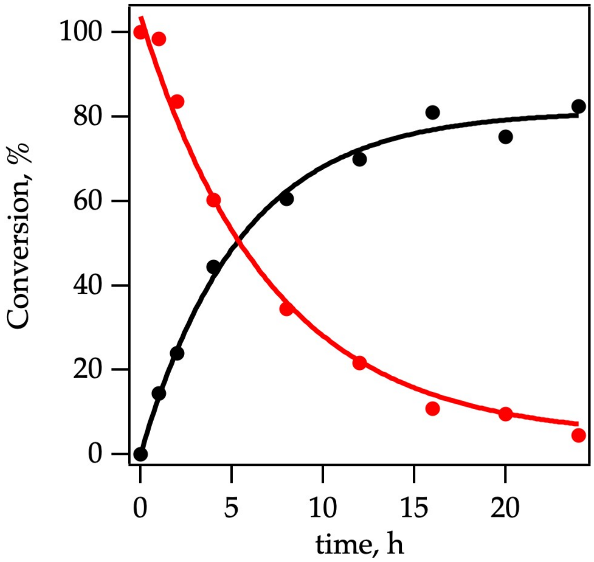

Figure 1. Plot of product yield and the amount of remaining starting material versus time (NMR spectroscopy with mesitylene internal standard) for the formation of **49** (6.17–6.41 ppm in CD_3CN). Red curve represents a fit to $f(x) = ae^{-bx} + c$. Black curve represents a fit to $f(x) = (a-1)e^{-bx} + c$.

Time, h	Product, %	Starting material, %
0	0	100
1	14	98
2	24	84
4	44	60
8	61	34
12	70	22
16	81	11
20	75	9
24	83	5

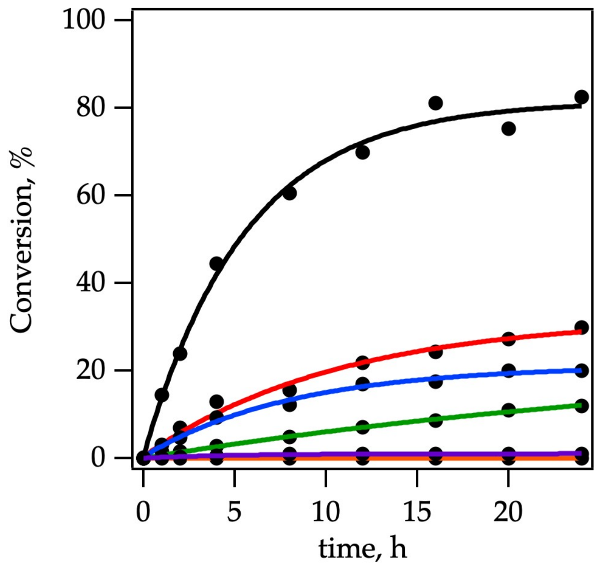

Figure 2. Plot of product yields from respective hydrazines versus time (NMR spectroscopy with mesitylene internal standard) for the formation of **49** (6.17–6.41 ppm in CD_3CN). Curves represents first-order fits to $f(x) = (a–1)e^{–bx} + c$.

Time, h	Product yield by hydrazine					
	[2.2.2]	5 memb	[3.2.2]	[2.2.1]	6 memb	7 memb
0	0	0	0	0	–	–
1	14	2	3	1	–	–
2	24	7	5	2	–	–
4	44	13	9	3	–	–
8	61	16	12	5	–	–
12	70	22	17	7	–	–
16	81	24	18	9	–	–
20	75	27	20	11	–	–
24	83	30	20	12	–	–

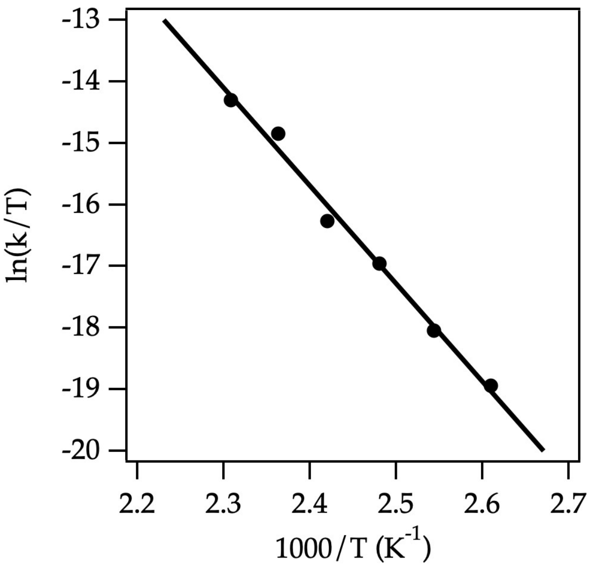

Figure 3. Eyring plot for formation of **49** (NMR spectroscopy with mesitylene internal standard, 6.17–6.41 ppm in CD_3CN). Rate constants were obtained from first-order fits to $f(x) = (a-1)e^{-bx} + c$. Activation parameters are shown below.

Temp, °C	Temp, °K	k, s^{-1}	ln k	1000/T	ln(k/T)
110	383.15	$2.28{\bullet}10^{-6}$	–1.30	2.61	–18.9
120	393.15	$5.69{\bullet}10^{-6}$	–1.21	2.54	–18.1
130	403.15	$1.74{\bullet}10^{-5}$	–1.10	2.48	–17.0
140	413.15	$3.56{\bullet}10^{-5}$	–1.02	2.42	–16.3
150	423.15	$1.52{\bullet}10^{-4}$	–8.79	2.36	–14.8
160	433.15	$2.66{\bullet}10^{-4}$	–8.23	2.31	–14.3

$\Delta H^‡$	31.6 ± 0.7	kcal/mol
$\Delta S^‡$	–3 ± 2	e.u.
$\Delta G^‡$	32.4 ± 0.9	kcal/mol

Computational Data

All DFT calculations were performed with the Gaussian 09 program package.[9)] The geometry optimization of all the minima and transition states involved was carried out at the M06-2X level of theory[10)] with the 6-31G(d) basis set.[11)] The vibrational frequencies were computed at the same level to check whether each optimized structure is an energy minimum or a transition state and to evaluate its zero-point vibrational energy (ZPVE) and thermal corrections at 298 K. Solvent effects in acetonitrile were computed at the M06-2X/6-311+G(d,p) level using the gas-phase optimized structures. Solvation energies were evaluated by a self-consistent reaction field (SCRF) using the PCM model.

Table 7. Geometric coordinates and thermally corrected M06-2X energies for **4**.

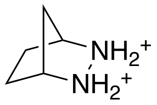

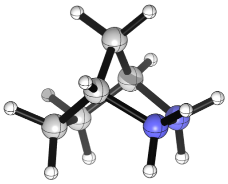

$G_{Acetonitrile}$ = –306.651320753 Hartree

Atom	X	Y	Z	Atom	X	Y	Z
C	0.00000000	0.00000000	0.00000000	H	-2.43261200	0.31831100	-0.90968200
C	-1.19014400	0.34414500	0.89699900	H	-3.33910600	0.30661300	0.49391600
C	-1.23921100	-0.78041800	1.92865200	H	-3.33902900	-1.86746100	0.49376400
C	-1.19000800	-1.90486900	0.89690100	H	-2.43247600	-1.87890000	-0.90980200
C	0.00010100	-1.56050400	-0.00004900	H	-2.11029100	-0.78050600	2.59217800
H	-0.06621200	-2.00738300	-0.99712600	H	-0.33544700	-0.78040600	2.54568400
H	0.90491600	-1.96811300	0.45927900	H	-1.32426700	1.37888300	1.21852400
H	-1.32406100	-2.93965200	1.21830400	H	-0.06641100	0.44692900	-0.99704900
N	-2.46678200	-1.51948800	0.05971000	H	0.90476600	0.40771000	0.459328000
N	-2.46684100	-0.04122800	0.05978800				

Table 8. Geometric coordinates and thermally corrected M06-2X energies for **6**.

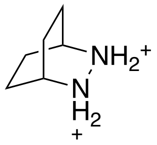

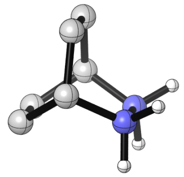

$G_{Acetonitrile}$ = –345.936858564 Hartree

Atom	X	Y	Z	Atom	X	Y	Z
C	0.00000000	0.00000000	0.00000000	H	-3.15628400	1.27845900	-0.79888500
C	0.52220600	1.27586600	-0.64588500	N	-1.51310500	1.27706400	-2.11960200
H	1.60574700	1.27516600	-0.79800300	H	-1.84974200	2.10892700	-2.63238000
C	0.00181500	2.55160500	0.00172900	H	-1.84953400	0.44459500	-2.63154200
C	-1.55083800	2.55321300	0.00038000	N	-0.03693800	1.27724300	-2.11933400
C	-2.07280100	1.27768100	-0.64635400	H	0.29967700	2.10997300	-2.63071900
C	-1.55265300	0.00161500	0.00080500	H	0.29988300	0.44564500	-2.63241700
H	-1.95961100	-0.88538200	-0.49616600	H	-1.95529100	3.44059900	-0.49797800
H	-1.94950000	-0.01919700	1.02005000	H	-1.94905200	2.57588300	1.01905100

H	0.40894200	3.43885300	-0.49465900
H	0.39830200	2.57191500	1.02112300

H	0.40462500	-0.88713100	-0.49867300
H	0.39785200	-0.02320200	1.01879900

Table 9. Geometric coordinates and thermally corrected M06-2X energies for **18**.

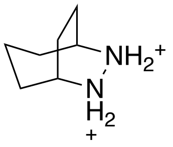

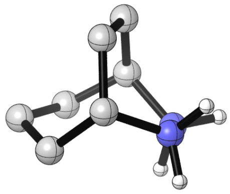

$G_{Acetonitrile}$ = –385.212993610 Hartree

Atom	X	Y	Z
C	0.00000000	0.00000000	0.00000000
H	0.59731000	-0.91755800	-0.02125600
C	0.07192500	0.68860500	1.35998200
C	-0.19698900	2.21510200	1.26995900
C	-0.80450200	2.67562900	-0.05182500
H	-0.73865300	3.76331100	-0.15373100
N	0.16896300	2.20219900	-1.21887000
H	0.90218900	2.90927300	-1.39164100
H	-0.38558100	2.12016200	-2.08692100
N	0.81954100	0.91237500	-0.97298200
H	1.74782500	1.09384700	-0.55595300
H	0.97705500	0.44064600	-1.87925800
C	-2.21186600	2.19366700	-0.38709300

Atom	X	Y	Z
C	-2.47924200	0.72131600	-0.07269100
H	-3.43709000	0.43366200	-0.51377400
H	-2.60253100	0.59357300	1.00747300
C	-1.40230100	-0.24690900	-0.57310600
H	-1.67495000	-1.26228500	-0.26596600
H	-1.37001900	-0.29047500	-1.67262700
H	-2.44238200	2.43217200	-1.43547700
H	-2.88989500	2.82504200	0.19869400
H	0.72303600	2.77762800	1.46372600
H	-0.89660100	2.52910100	2.05128600
H	1.05204700	0.51395900	1.81420300
H	-0.65182000	0.19268500	2.01172800

Table 10. Geometric coordinates and thermally corrected M06-2X energies for **19**.

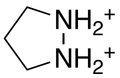

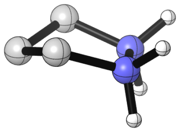

$G_{Acetonitrile}$ = –229.278920566 Hartree

Atom	X	Y	Z
C	0.00000000	0.00000000	0.00000000

Atom	X	Y	Z
C	-0.80775300	1.19374900	-0.48510700

C	-0.00033400	2.38781900	-0.00020400
H	-0.15312300	3.32104000	-0.54729600
H	-0.06683900	2.55502200	1.07716100
N	1.46283500	1.93362000	-0.27926600
H	1.79069200	2.26997200	-1.20159900
H	2.13191600	2.28855500	0.42680800
N	1.46294500	0.45445000	-0.27950500
H	2.13242200	0.09935900	0.42611400
H	1.79038900	0.11847100	-1.20212500
H	-0.93655600	1.19363600	-1.57182200
H	-1.80316000	1.19365400	-0.02883300
H	-0.06626500	-0.16691100	1.07742700
H	-0.15269300	-0.93342600	-0.54677900

Table 11. Geometric coordinates and thermally corrected M06-2X energies for **20**.

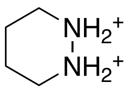

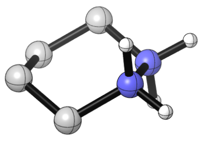

$G_{Acetonitrile}$ = –268.561940283 Hartree

Atom	X	Y	Z
C	0.00000000	0.00000000	0.00000000
C	-1.24082100	0.73551500	0.47658300
C	-1.24011100	2.19563400	0.01348600
C	0.00135200	2.92996700	0.49020800
H	0.09475300	2.98759700	1.57967700
H	0.12275000	3.92927400	0.06197500
N	1.25863700	2.15801200	0.01052300
H	2.12840500	2.62301600	0.32875000
H	1.28006400	2.13352500	-1.02512000
N	1.25804400	0.77087600	0.47954600
H	1.27959900	0.79537300	1.51518700
H	2.12736800	0.30509400	0.16125800
H	-2.10335600	2.72555100	0.42899200
H	-1.32995700	2.26824500	-1.07735200
H	-1.33080900	0.66304400	1.56741800
H	-2.10455800	0.20641400	0.06105000
H	0.09332500	-0.05785800	-1.08946300
H	0.12038600	-0.99938800	0.42833500

Table 12. Geometric coordinates and thermally corrected M06-2X energies for **21**.

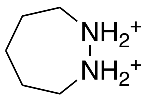

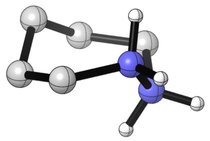

$G_{Acetonitrile}$ = –307.830754037 Hartree

Atom	X	Y	Z	Atom	X	Y	Z

C	0.00000000	0.00000000	0.00000000
C	1.25599000	-0.72703200	-0.50332300
C	1.29889700	-2.23038000	-0.30155100
H	1.37067000	-2.54549700	0.74583000
H	2.10516600	-2.71056700	-0.86453700
N	0.01139000	-2.92742400	-0.83072200
H	-0.21741600	-2.58366800	-1.78042300
H	0.16914900	-3.94744700	-0.89871100
N	-1.15419500	-2.68646600	0.02529200
C	-2.06554600	-1.50649700	-0.41962400
C	-1.25054100	-0.29392600	-0.84768300
H	-0.98512600	-0.36304300	-1.91043400
H	-1.95307500	0.54513400	-0.78904900
H	-2.71361200	-1.90740300	-1.20321400
H	-2.66007100	-1.32401300	0.48129300
H	-1.71580000	-3.55304200	0.08963800
H	-0.78397700	-2.49088100	0.97063900
H	1.41795800	-0.49597400	-1.56273600
H	2.14107200	-0.34999900	0.02285900
H	-0.17927000	-0.18940000	1.06860500
H	0.20490400	1.07136700	-0.06404800

Table 13. Geometric coordinates and thermally corrected M06-2X energies for **TFA anion**.

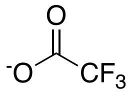

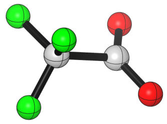

$G_{Acetonitrile}$ = –526.369750577 Hartree

Atom	X	Y	Z
C	0.00000000	0.00000000	0.00000000
C	1.56986700	-0.00520900	-0.00000900
O	2.09114500	1.11976200	0.00003200
O	2.02819700	-1.15935400	-0.00000600
F	-0.51134700	-0.63826100	1.08021900
F	-0.51137800	-0.63866600	-1.07991500
F	-0.55696900	1.22770300	-0.00023100

Table 14. Geometric coordinates and thermally corrected M06-2X energies for **H_3O^+**.

H_3O^+

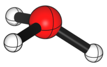

$G_{Acetonitrile}$ = –76.8007163883 Hartree

Atom	X	Y	Z
C	0.00000000	0.00000000	0.00000000
C	1.56986700	-0.00520900	-0.00000900
O	2.09114500	1.11976200	0.00003200
O	2.02819700	-1.15935400	-0.00000600
F	-0.51134700	-0.63826100	1.08021900
F	-0.51137800	-0.63866600	-1.07991500
F	-0.55696900	1.22770300	-0.00023100

Table 15. Geometric coordinates and thermally corrected M06-2X energies for **10**.

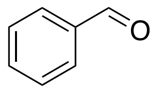

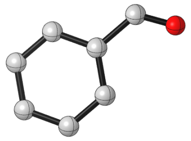

$G_{Acetonitrile}$ = –345.452697982 Hartree

Atom	X	Y	Z
C	0.00000000	0.00000000	0.00000000
O	-0.84028500	-0.86808600	0.00004100
C	1.46106700	-0.25382200	0.00001100
C	2.35079200	0.81978000	0.00001800
C	3.72253000	0.58555800	0.00002600
C	4.19784100	-0.72362900	0.00002800
C	3.30789700	-1.79964500	0.00002100
C	1.93954700	-1.56667400	0.00001200
H	1.22112500	-2.38101700	0.00000600
H	3.68746400	-2.81658500	0.00002100
H	5.26782900	-0.90878200	0.00003400
H	4.41831900	1.41847900	0.00003200
H	1.96516700	1.83706500	0.00001700
H	-0.28939400	1.07207500	0.00004500

Table 16. Geometric coordinates and thermally corrected M06-2X energies for **11-*E***.

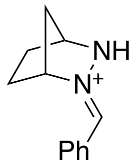

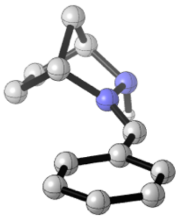

$G_{Acetonitrile}$ = –575.303788331 Hartree

Atom	X	Y	Z
C	0.00000000	0.00000000	0.00000000
C	-0.68474900	-0.90863900	1.03989200
C	0.50474000	-1.48540500	1.82091000
C	1.17276300	-2.03480500	0.56088700
C	1.30856800	-0.78424800	-0.32660300
H	1.39456800	-1.04132300	-1.38593300
H	2.19882500	-0.21256700	-0.05483000
H	2.06692500	-2.64191600	0.69450900
N	0.07183300	-2.89387000	0.05069900
H	0.13556200	-3.05166800	-0.95562200
N	-1.09771100	-2.13813200	0.31137600
C	-2.26828200	-2.61138800	0.04571000
H	-2.25809800	-3.63198600	-0.33677400
C	-3.54616000	-1.94778900	0.21677500
C	-4.66312100	-2.78181600	0.38877700
C	-5.92173100	-2.22859500	0.57743500
C	-6.07860900	-0.84387000	0.56046800
C	-4.97998800	-0.00941900	0.34684500
C	-3.71593900	-0.55302600	0.17628000
H	-2.87696600	0.10158500	-0.03738800

H	-5.11486600	1.06573800	0.30083800
H	-7.06432200	-0.41074200	0.69558600
H	-6.77955500	-2.87512300	0.72676600
H	-4.53635800	-3.86096500	0.38997300
H	0.22573300	-2.25570100	2.54221100
H	1.08476500	-0.69763400	2.30450800
H	-1.52338800	-0.50343600	1.60188400
H	-0.62852800	0.17069300	-0.87786900
H	0.20744500	0.97020000	0.45754700

Table 17. Geometric coordinates and thermally corrected M06-2X energies for **11-*Z***.

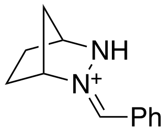

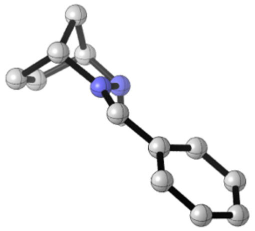

$G_{Acetonitrile}$ = –575.306798352 Hartree

Atom	X	Y	Z
C	0.00000000	0.00000000	0.00000000
C	-0.65105300	-0.14614700	1.38834500
C	0.07983500	-1.35534000	1.98091500
C	-0.29770400	-2.25589900	0.80090600
C	0.24687400	-1.48192600	-0.41365000
H	-0.27082700	-1.75631700	-1.33680300
H	1.30929200	-1.69059000	-0.55883300
H	-0.03333800	-3.30971900	0.87198900
N	-1.77906300	-2.12185200	0.85840500
H	-2.24914700	-2.34726500	-0.02230700
N	-1.98597300	-0.76380800	1.14507000
C	-3.12837200	-0.16362700	1.19487500
C	-4.44886200	-0.71283800	0.97243400
C	-5.47379300	0.22986500	0.77025600
C	-6.77914900	-0.18774700	0.56502800
C	-7.07864200	-1.54942000	0.58712200
C	-6.07427100	-2.49111900	0.81603300
C	-4.76037600	-2.08655300	1.00000700
H	-3.99629000	-2.82493600	1.21254700
H	-6.31930900	-3.54706700	0.85263900
H	-8.10120500	-1.87961500	0.43467100
H	-7.56244700	0.54355300	0.39944100
H	-5.23780400	1.29066600	0.77078600
H	-3.05254600	0.89926400	1.41382700
H	-0.32333200	-1.69428800	2.93674100
H	1.15439100	-1.18139700	2.05544700
H	-0.76095000	0.75524600	1.98976100
H	-0.64027200	0.54751100	-0.69547600
H	0.93451700	0.55653500	0.10442800

Table 18. Geometric coordinates and thermally corrected M06-2X energies for **8-*E***.

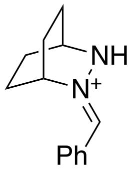

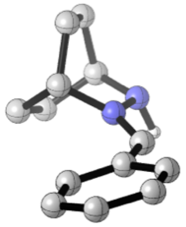

$G_{Acetonitrile}$ = –614.588230053 Hartree

Atom	X	Y	Z
C	0.00000000	0.00000000	0.00000000
N	-1.22861200	-0.81054400	-0.19288500
H	-1.33873500	-1.09365500	-1.16509300
N	-2.37158900	-0.08628900	0.18369000
C	-3.54625900	-0.55714200	-0.07935900
H	-3.53878200	-1.54223500	-0.54701300
C	-4.83630100	0.06086200	0.18919200
C	-5.90240300	-0.80929800	0.46610300
C	-7.16560100	-0.29878900	0.73164200
C	-7.38164000	1.07716800	0.68411800
C	-6.33742700	1.94499300	0.36254600
C	-5.06764900	1.44438000	0.11534200
H	-4.27212900	2.12108100	-0.18176900
H	-6.51849100	3.01202800	0.29146400
H	-8.37214500	1.47581000	0.87798000
H	-7.98283900	-0.97295800	0.96348100

Atom	X	Y	Z
H	-5.73275700	-1.88228900	0.48954900
C	-2.04181100	1.10514600	1.01764600
C	-1.32649600	2.11481400	0.11175200
C	-0.17918100	1.38639200	-0.63047600
H	0.75606000	1.94740600	-0.56210500
H	-0.41131200	1.27823400	-1.69506200
H	-0.94698000	2.91566700	0.75301800
H	-2.02744900	2.57060000	-0.59350200
H	-2.97804600	1.47665100	1.42884300
C	-1.10648300	0.60925700	2.12965100
H	-0.94807300	1.44309800	2.81857800
H	-1.60497400	-0.18573100	2.69072700
C	0.21929200	0.11495000	1.50840700
H	1.03809900	0.81388300	1.69986800
H	0.50284400	-0.85443300	1.92230000
H	0.79799500	-0.56654400	-0.48363100

Table 19. Geometric coordinates and thermally corrected M06-2X energies for **8-Z**.

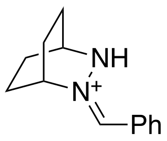

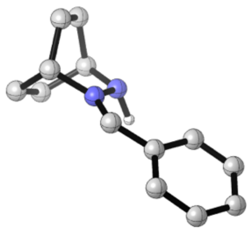

$G_{Acetonitrile}$ = –614.592782532 Hartree

Atom	X	Y	Z
C	0.00000000	0.00000000	0.00000000
N	1.41341200	-0.26422400	0.37197500
H	2.06464500	-0.07075000	-0.39238000
N	1.58554400	-1.59142000	0.76144900
C	2.74296900	-2.14870000	0.91632700
C	4.05709300	-1.56746800	0.72047900
C	5.10257900	-2.48567700	0.51218000
C	6.40135400	-2.03931700	0.32660300
C	6.67619400	-0.67310800	0.38039500
C	5.65309600	0.24332000	0.62309900
C	4.34457100	-0.19004200	0.78508200

Atom	X	Y	Z
H	3.56576100	0.52867800	1.01289800
H	5.87766900	1.30238600	0.68996400
H	7.69374700	-0.32085900	0.24538500
H	7.19939900	-2.75275900	0.15312500
H	4.88722900	-3.55061200	0.48974600
H	2.69238900	-3.19721600	1.19826600
C	0.29635700	-2.29649000	1.01954200
C	-0.41507200	-2.43861500	-0.33095400
C	-0.48619500	-1.04733400	-1.00749700
H	-1.50546200	-0.80980600	-1.32213400
H	0.14123700	-1.01718800	-1.90395600

H	-1.41234600	-2.84271700	-0.13446400
H	0.10993800	-3.16313500	-0.95887900
H	0.55159000	-3.26055000	1.46241400
C	-0.50806500	-1.41872100	1.98262500
H	-1.42050300	-1.95987700	2.24552900
H	0.06120100	-1.27297800	2.90464300
C	-0.81795300	-0.06931400	1.29167400
H	-1.87914800	0.01784500	1.04381000
H	-0.55785900	0.76713500	1.94290900
H	-0.00236300	1.00646700	-0.42268700

Table 20. Geometric coordinates and thermally corrected M06-2X energies for **96-*E***.

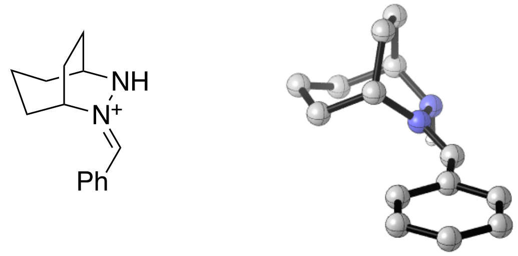

$G_{Acetonitrile}$ = –653.863692595 Hartree

Atom	X	Y	Z
C	0.00000000	0.00000000	0.00000000
H	-0.95221800	0.27705900	0.45144300
C	0.92964100	-0.51815500	1.10858200
C	2.13182900	-1.33277300	0.57500200
C	2.09679000	-1.44666100	-0.94941400
H	2.74525600	-2.27027500	-1.26176800
C	2.54953300	-0.16964100	-1.68047300
C	2.06767700	1.13047100	-1.03827100
H	2.36365500	1.97936400	-1.66095800
H	2.57442700	1.27011200	-0.07639400
C	0.55638000	1.17944300	-0.81771800
H	0.29781100	2.09543700	-0.27511200
H	0.03382000	1.22652500	-1.78181500
H	2.22014100	-0.22389600	-2.72715200
H	3.64436400	-0.16813800	-1.70552500
N	0.75450400	-1.93079200	-1.33445100
N	-0.32339900	-1.15165100	-0.88687800
C	-1.51619700	-1.57439900	-1.16315700

Atom	X	Y	Z
H	-1.53354200	-2.55052900	-1.64792900
C	-2.79313700	-0.92832100	-0.90743400
C	-3.89048100	-1.78691700	-0.72837400
C	-5.15229700	-1.26535600	-0.47996900
C	-5.33660600	0.11554300	-0.44868600
C	-4.26106900	0.97620100	-0.67238300
C	-2.99279900	0.46339100	-0.89976700
H	-2.17422400	1.14255000	-1.11583300
H	-4.41623300	2.04959500	-0.68103300
H	-6.32527600	0.52527000	-0.26906600
H	-5.99181100	-1.93389200	-0.32377800
H	-3.74554700	-2.86308400	-0.76847700
H	0.67661500	-2.02127900	-2.34553300
H	2.10769600	-2.33883400	0.99945300
H	3.08020300	-0.87844800	0.87380200
H	0.33357600	-1.13317300	1.78760600
H	1.25712400	0.35479900	1.68092300

Table 21. Geometric coordinates and thermally corrected M06-2X energies for **96-*Z***.

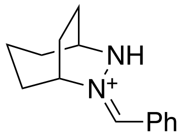

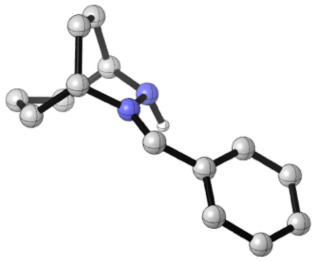

$G_{Acetonitrile}$ = –653.868836620 Hartree

Atom	X	Y	Z
C	0.00000000	0.00000000	0.00000000
H	0.33665300	-0.80633500	0.65505400
C	-0.87333000	0.96957300	0.80343900
C	-1.09005400	2.32712100	0.09137700
C	-0.25003300	2.42296700	-1.18485500
H	-0.12465500	3.47478300	-1.45599800
C	-0.85106800	1.67325000	-2.38853200
C	-1.55011200	0.36301200	-2.02665700
H	-1.89741300	-0.13040000	-2.93883200
H	-2.45099100	0.58424900	-1.44242000
C	-0.67168800	-0.60911200	-1.24085900
H	-1.28323600	-1.44677700	-0.88895000
H	0.10600100	-1.03954800	-1.88372300
H	-0.06041500	1.49624900	-3.13011000
H	-1.57656600	2.33895100	-2.86768300
N	1.12674300	2.01477300	-0.83005600
N	1.26064100	0.70711300	-0.39264600
C	2.41360200	0.12649800	-0.28577000

Atom	X	Y	Z
H	2.35819000	-0.90926500	0.03845400
C	3.72831100	0.66543600	-0.58471500
C	4.72757400	-0.28431800	-0.86476800
C	6.02302400	0.12250700	-1.14268200
C	6.34422000	1.47922900	-1.10812800
C	5.37055600	2.42607900	-0.79244400
C	4.06350000	2.03253400	-0.53956800
H	3.32586600	2.77457800	-0.25520300
H	5.63202600	3.47747400	-0.74020000
H	7.36051800	1.79862700	-1.31526200
H	6.78475800	-0.61481800	-1.37145100
H	4.47880000	-1.34217000	-0.87012800
H	1.79110500	2.16783400	-1.59069300
H	-0.79839300	3.13774800	0.76216600
H	-2.14208300	2.48000500	-0.16216400
H	-0.40177200	1.12939400	1.77645100
H	-1.82409200	0.46254800	0.99280500

Table 22. Geometric coordinates and thermally corrected M06-2X energies for **97-*E***.

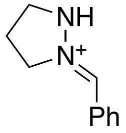

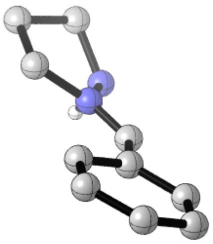

$G_{Acetonitrile}$ = –497.932857090 Hartree

Atom	X	Y	Z
C	0.00000000	0.00000000	0.00000000
N	-0.33302700	-1.43523400	-0.21744500
N	0.82786400	-2.24765300	-0.29343400
C	1.83309100	-1.48322400	0.46323600

Atom	X	Y	Z
C	1.52688400	-0.01255000	0.15117500
H	1.85963300	0.66505000	0.93826100
H	2.00198800	0.28858400	-0.78577800
H	1.69852700	-1.70794000	1.52434200

H	2.82948100	-1.80349300	0.15854500
H	1.09031100	-2.28758500	-1.28130000
C	-1.48686900	-2.02418700	-0.19061200
C	-2.80946700	-1.45917500	-0.10077200
C	-3.83603300	-2.40407400	0.10089200
C	-5.15406100	-1.99651700	0.22854400
C	-5.46502600	-0.64087800	0.13916400
C	-4.46035500	0.30555300	-0.07914900
C	-3.13909100	-0.09154600	-0.19799400
H	-2.38477500	0.66035100	-0.38730800
H	-4.71424500	1.35671700	-0.16102300
H	-6.49651000	-0.31611500	0.23182300
H	-5.93590100	-2.73013300	0.38967200
H	-3.58795400	-3.46012100	0.16193100
H	-1.40590000	-3.10872100	-0.23001700
H	-0.33570300	0.58244400	-0.86038600
H	-0.52700700	0.33356700	0.89818700

Table 23. Geometric coordinates and thermally corrected M06-2X energies for **97-*Z***.

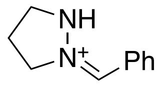

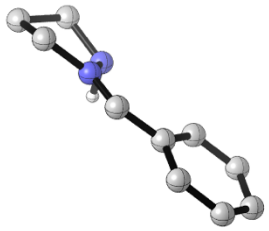

$G_{Acetonitrile}$ = –497.935331899 Hartree

Atom	X	Y	Z
C	0.00000000	0.00000000	0.00000000
N	-1.29271700	0.73422300	0.15288800
N	-1.09038400	2.11423500	0.30987400
C	0.35783100	2.33420900	0.11983100
C	1.01420600	1.01050800	0.52081200
H	1.99624300	0.87054900	0.06797900
H	1.11203200	0.93483700	1.60718500
H	0.53167400	2.56232800	-0.93482600
H	0.66890200	3.18979400	0.71909900
H	-1.39095400	2.35834400	1.25645900
C	-2.45945300	0.17487700	0.10217700
C	-3.77246700	0.76739600	0.16890500
C	-4.83359400	-0.15316800	0.28650500
C	-6.14444700	0.28966000	0.34522500
C	-6.41202500	1.65601500	0.26636400
C	-5.37071800	2.57639200	0.12876500
C	-4.05361500	2.14752200	0.08854500
H	-3.25462000	2.86597900	-0.03945600
H	-5.59061800	3.63582500	0.05410000
H	-7.43808800	2.00756900	0.30688000
H	-6.95480400	-0.42385900	0.44438900
H	-4.62031400	-1.21774200	0.33370200
H	-2.41224800	-0.90910600	0.01729700
H	-0.05277600	-0.93281200	0.56286900
H	0.13802900	-0.21478000	-1.06338500

Table 24. Geometric coordinates and thermally corrected M06-2X energies for **98-*E***.

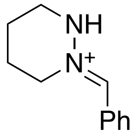

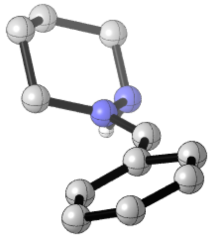

$G_{Acetonitrile}$ = –537.210881000 Hartree

Atom	X	Y	Z
C	0.00000000	0.00000000	0.00000000
C	1.23371500	-0.79023000	-0.42847200
H	1.65528800	-0.36945500	-1.35048500
H	2.01034200	-0.72948600	0.33849100
C	0.83520300	-2.24218700	-0.65964000
N	-0.25827300	-2.38303300	-1.64097700
N	-1.37865400	-1.57792200	-1.30358000
C	-1.13977200	-0.14660000	-1.01093500
H	-0.89509800	0.34027600	-1.96241100
H	-2.06211800	0.27632900	-0.61688500
C	-2.51013900	-2.20935100	-1.24479000
C	-3.84067100	-1.67885200	-1.04023800
C	-4.27099800	-0.41201000	-1.47843600
C	-5.58174800	-0.01990900	-1.25803300
C	-6.46642300	-0.87203500	-0.59333300
C	-6.05611600	-2.13864600	-0.18007500
C	-4.75538900	-2.55250900	-0.42508100
H	-4.43474100	-3.54652700	-0.12571100
H	-6.75326700	-2.80605000	0.31429500
H	-7.48889000	-0.55314200	-0.41762800
H	-5.92180700	0.94658300	-1.61330400
H	-3.60760100	0.23691700	-2.04061600
H	-2.39508900	-3.28552900	-1.36466000
H	0.05410600	-2.06118300	-2.55978500
H	0.49317500	-2.70913800	0.27004500
H	1.65736600	-2.84646900	-1.04956700
H	0.21949400	1.06643000	0.09922300
H	-0.34870300	-0.35294200	0.97729700

Table 25. Geometric coordinates and thermally corrected M06-2X energies for **98-*Z***.

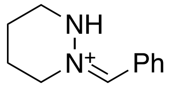

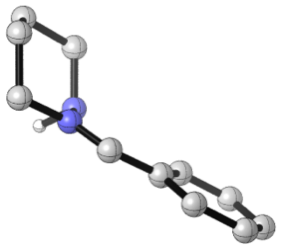

$G_{Acetonitrile}$ = –537.214530912 Hartree

Atom	X	Y	Z
C	0.00000000	0.00000000	0.00000000
C	-0.21911500	1.50475300	-0.16529900
H	-0.75539600	1.70362200	-1.10220900
H	-0.83947600	1.89175300	0.64768300
C	1.12408300	2.22829700	-0.18262800
N	2.05370200	1.68830200	-1.20247800
N	2.20848800	0.29737400	-1.02716700
C	0.97041500	-0.51374200	-1.06316600
H	0.54956300	-0.40021500	-2.06885800
H	1.24007000	-1.56084200	-0.91327200
C	3.37842700	-0.21001000	-0.77834300
C	4.67842800	0.39593800	-0.66076500
C	5.71404500	-0.52191600	-0.38026900
C	7.02186000	-0.08975300	-0.23975900
C	7.31193700	1.26676800	-0.37844100
C	6.29703600	2.18670100	-0.65624200
C	4.98525300	1.76771700	-0.79847700
H	4.19978200	2.47753200	-1.01458900
H	6.53618400	3.23925300	-0.76246800
H	8.33513600	1.61285800	-0.27034700
H	7.81070400	-0.80213800	-0.02577200
H	5.48377800	-1.57878800	-0.27415100
H	3.34995000	-1.28949000	-0.63911300
H	1.65823300	1.81581800	-2.13704700
H	1.63267900	2.13999600	0.78329700
H	1.01859600	3.29203600	-0.40740800
H	-0.94028600	-0.54995100	-0.09300400
H	0.41124800	-0.21712400	0.99280400

Table 26. Geometric coordinates and thermally corrected M06-2X energies for **99-*E***.

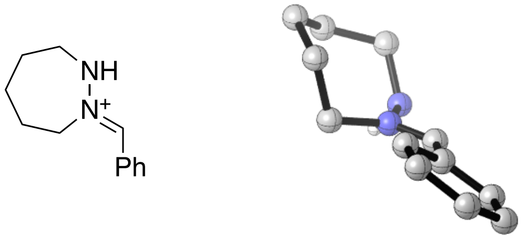

$G_{Acetonitrile}$ = –576.480387457 Hartree

Atom	X	Y	Z
C	0.00000000	0.00000000	0.00000000
N	1.04680900	-0.50527900	0.91568600
N	2.10571900	0.41347300	1.10309500
C	1.81226600	1.73412800	1.69405100
H	1.09719200	1.55554300	2.50277400
H	2.73698800	2.09871000	2.14348800
C	1.24694200	2.69277900	0.63838000
H	1.79501100	2.52644200	-0.29705300
H	1.47798300	3.71661100	0.95011200
C	-0.26728500	2.56517000	0.39585900
C	-0.85885700	1.15156000	0.53187700
H	-1.09957400	0.93802100	1.58231900
H	-1.81957500	1.13200900	0.00819400
H	-0.47640500	2.95109700	-0.60640500
H	-0.79998800	3.22234100	1.09010000
C	3.26652700	0.00038700	0.69378600
C	4.53847400	0.68664300	0.65259300
C	4.72388200	2.08231100	0.58745500
C	6.00645400	2.60326300	0.53086300
C	7.11380700	1.75247600	0.54835600
C	6.94301100	0.37006700	0.59208500
C	5.66283900	-0.16149600	0.62202000
H	5.52326900	-1.23891500	0.63724800
H	7.80396000	-0.28893800	0.58730800
H	8.11415900	2.17157200	0.50799500
H	6.14739500	3.67641000	0.46267100
H	3.88110500	2.76054600	0.52748400
H	3.24121200	-1.03430400	0.35797000
H	0.64281500	-0.67739400	1.83845900
H	0.49135300	0.25174300	-0.94612200
H	-0.62625200	-0.87576000	-0.19035100

Table 27. Geometric coordinates and thermally corrected M06-2X energies for **99-*Z***.

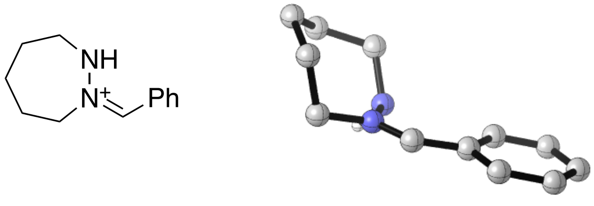

$G_{Acetonitrile}$ = –576.483692615 Hartree

Atom	X	Y	Z
C	0.00000000	0.00000000	0.00000000
N	-0.87116400	-0.61467800	1.02804600
N	-1.00005300	-2.00626200	0.89165300
C	0.20888300	-2.86457700	0.98529300

H	0.78955100	-2.50479100	1.84028400
H	-0.13596300	-3.87460500	1.21653100
C	1.01077900	-2.83990200	-0.31948200
H	0.29409700	-2.89529500	-1.14769100
H	1.59265500	-3.76546700	-0.36519500
C	1.95752800	-1.64092200	-0.49659900
C	1.49283300	-0.31498000	0.12455100
H	1.75903700	-0.27729800	1.18990600
H	2.05610400	0.49941200	-0.34170200
H	2.12035600	-1.50020800	-1.56953600
H	2.93595800	-1.88730000	-0.07296100
C	-2.15962500	-2.51571900	0.59703600
C	-3.44938600	-1.91141500	0.38613400
C	-4.47542500	-2.83998300	0.10529700
C	-5.77404900	-2.41368300	-0.11613300
C	-6.06496200	-1.05153200	-0.06101000
C	-5.05953400	-0.12043000	0.21337900
C	-3.75716800	-0.53389500	0.43627200
H	-2.98038100	0.18531900	0.65192000
H	-5.29870200	0.93672300	0.25228500
H	-7.08094800	-0.71011400	-0.23338500
H	-6.55504000	-3.13523300	-0.32829200
H	-4.24505200	-3.90137400	0.06507600
H	-2.13190200	-3.59946700	0.49488500
H	-0.48170100	-0.44695500	1.95756000
H	-0.40432700	-0.28259200	-0.97850100
H	-0.16706300	1.07432500	0.11777800

Table 28. Geometric coordinates and thermally corrected M06-2X energies for **7**.

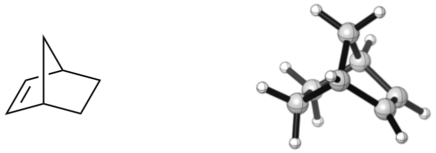

$G_{Acetonitrile}$ = –272.552850884 Hartree

**Atom**	**X**	**Y**	**Z**
C	0.00000000	0.00000000	0.00000000
C	0.05406800	1.12467600	1.05120100
C	-0.00006200	2.24931500	-0.00001300
C	1.26505800	1.90215700	-0.84619200
C	1.26510000	0.34719900	-0.84616300
H	2.16515200	-0.04804100	-0.36471100
H	1.20856400	-0.07735400	-1.85141400
H	2.16509000	2.29746700	-0.36476300
H	1.20846700	2.32667400	-1.85145400
H	-0.02943600	3.27648800	0.36729700
C	-1.19053800	1.79293400	-0.82550500
H	-1.83120700	2.44973700	-1.40427400
C	-1.19049100	0.45630700	-0.82551200
H	-1.83112100	-0.20052200	-1.40429300
H	0.98060300	1.12469800	1.63675500
H	-0.81244800	1.12464700	1.71739600
H	-0.02934100	-1.02715500	0.36735800

Table 29. Geometric coordinates and thermally corrected M06-2X energies for **12a**.

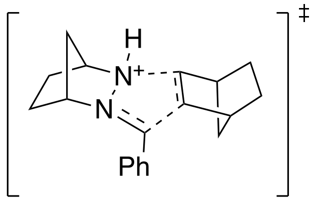

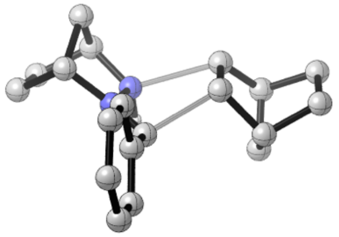

$G_{Acetonitrile}$ = –847.815575397 Hartree

Atom	X	Y	Z
C	0.00000000	0.00000000	0.00000000
C	0.44813400	-1.12102100	0.95964200
C	1.49558700	-0.41209300	1.82496000
C	2.33390500	-0.00766900	0.60528500
C	1.32485900	0.77773300	-0.25453100
H	1.61575400	0.79131500	-1.30844300
H	1.26200400	1.81503400	0.08315600
H	3.29993900	0.46345500	0.78384100
N	2.54171300	-1.35047400	0.00291900
N	1.32524600	-2.00403200	0.15156200
C	1.27743700	-3.31491100	-0.09648300
C	0.08853400	-4.12995000	0.22514000
C	-0.44521300	-4.19714900	1.51885500
C	-1.55468600	-4.99191700	1.77419300
C	-2.13555800	-5.73164000	0.74379600
C	-1.59585500	-5.68826400	-0.53843200
C	-0.47995100	-4.89806200	-0.79570400
H	-0.05474400	-4.86705600	-1.79546500
H	-2.03895300	-6.27271600	-1.33776700
H	-3.00156900	-6.35284900	0.94759400
H	-1.96043700	-5.04658500	2.77884000
H	0.02826700	-3.65354200	2.33324200
H	1.82279200	-3.62099600	-0.98471100
H	2.84366800	-1.33281400	-0.97255800
H	2.01719700	-1.07244100	2.52154200
H	1.08157600	0.44810100	2.35368500
H	-0.34649300	-1.68303100	1.44058200
H	-0.44642800	-0.40651600	-0.90929300
H	-0.74783100	0.62387200	0.49592200
C	3.83937100	-3.08525900	1.11901400
H	3.92282600	-2.35500000	1.91736200
C	2.90253100	-4.09927000	1.05151900
H	2.25892400	-4.35402700	1.88904500
C	3.51855800	-5.16783600	0.14892700
C	4.68708200	-5.78475400	0.96886200
C	5.70606300	-4.61454600	1.05775800
C	5.00711500	-3.46971000	0.24892200
C	4.31300800	-4.29852200	-0.84477900
H	3.70438600	-3.69679100	-1.52789000
H	5.01982500	-4.88178100	-1.44124400
H	5.67346900	-2.66450100	-0.06221800
H	5.92771300	-4.31646100	2.08532600
H	6.65290200	-4.86840600	0.57340000
H	4.35452400	-6.13477300	1.94870100
H	5.11266600	-6.63926400	0.43605500
H	2.81515700	-5.89262800	-0.26793800

Table 30. Geometric coordinates and thermally corrected M06-2X energies for **24a**.

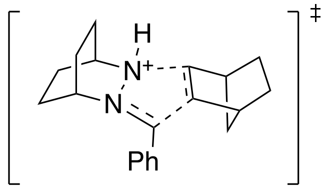

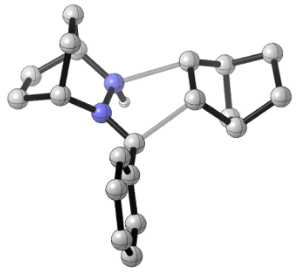

$G_{Acetonitrile}$ = –887.104609021 Hartree

Atom	X	Y	Z
C	0.00000000	0.00000000	0.00000000
C	-0.81131800	1.00497100	-0.86720300
C	-0.92336000	2.27311700	-0.02310800
C	0.34564800	2.81579500	0.00818300
H	0.71943600	3.49232400	0.76950800
C	1.24786900	1.93704600	-0.81941500
C	1.41328200	0.64621500	0.05097600
H	1.74257500	0.87605300	1.06713700
H	2.16726200	-0.00152300	-0.40454500
C	0.26892700	1.41638700	-1.88549400
H	-0.04193600	2.17832800	-2.60845100
H	0.66956300	0.56431900	-2.44148700
H	2.18662500	2.37682700	-1.15839000
H	-1.61313400	2.34807800	0.81281200
H	-1.75296000	0.61625600	-1.26224300
H	-0.44332100	-0.13241100	0.98946800
H	0.03120800	-0.97875500	-0.48612500
N	-0.12913500	4.83216400	-1.28828300
N	-1.49107000	4.80480800	-1.04396600
C	-2.09550200	3.62558900	-1.21873900
C	-3.50442600	3.37506100	-0.85172800
C	-3.99494400	3.57063200	0.44662400
C	-5.32374700	3.29436900	0.74019500
C	-6.17249900	2.81021000	-0.25528000
C	-5.68698000	2.58974600	-1.54082200
C	-4.35461200	2.86090500	-1.83650300
H	-3.97395200	2.68390500	-2.83902000
H	-6.34254200	2.20117400	-2.31280700
H	-7.20969300	2.59220800	-0.02272600
H	-5.69641200	3.44389900	1.74797900
H	-3.33043900	3.91446200	1.23620900
H	-1.74484100	3.07950000	-2.09083000
C	-1.93646700	5.94032500	-0.20734000
C	-1.01358200	6.01181500	1.01601000
H	-1.39163600	6.77268000	1.70362400
H	-1.03778400	5.05281600	1.54645900
C	0.41774100	6.34487200	0.52821000
C	0.45825900	6.16156000	-0.99293300
H	1.48176200	6.12902600	-1.37149300
C	-0.37791500	7.24891900	-1.67713600
H	0.09564000	8.22295200	-1.52955100
H	-0.40885800	7.06510000	-2.75570000
C	-1.79733700	7.20741400	-1.06522000
H	-1.97797700	8.06968200	-0.41611100
H	-2.56532600	7.21766000	-1.84198800
H	0.68333400	7.38031600	0.75980300
H	1.16049000	5.70644400	1.01216800
H	-2.97510900	5.75996900	0.06041300
H	0.06541000	4.51654900	-2.23904800

Table 31. Geometric coordinates and thermally corrected M06-2X energies for **100a**.

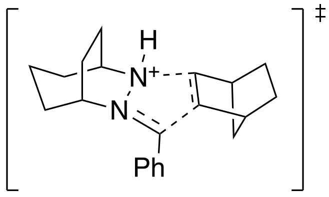

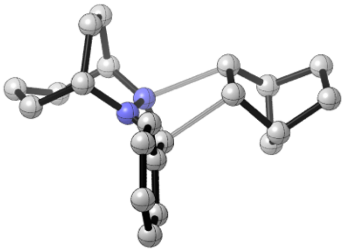

$G_{Acetonitrile}$ = –926.380600345 Hartree

Atom	X	Y	Z	Atom	X	Y	Z

C	0.00000000	0.00000000	0.00000000
C	0.53261900	1.10697000	0.87580700
C	-0.36186600	2.15592100	0.82509900
C	-1.49954500	1.68991700	-0.08013200
C	-2.25490900	0.59795900	0.73086500
C	-1.21946300	-0.56063700	0.80503700
H	-0.94756100	-0.82629800	1.82934100
H	-1.59285700	-1.46398500	0.31491200
H	-2.55855500	0.96028100	1.71558900
H	-3.15641900	0.28781700	0.19584800
C	-0.74180800	0.80159800	-1.08359100
H	-0.07795600	1.34011800	-1.76893000
H	-1.41045500	0.17749300	-1.68265300
H	-2.13427300	2.47985000	-0.48840200
H	-0.53166500	2.82609000	1.66267200
H	1.25547200	0.93707500	1.66717700
H	0.71473000	-0.75773000	-0.32425000
N	2.36347600	2.16778600	-0.32589500
N	1.95759500	3.45215800	-0.04813300
C	0.67002700	3.70312300	-0.30068200
C	-0.00815300	4.97770400	0.02162100
C	-0.12480200	5.47810400	1.32548100
C	-0.79968700	6.66918400	1.55932800
C	-1.37447400	7.36709400	0.49753300
C	-1.28578800	6.86393800	-0.79706300
C	-0.61471600	5.66837200	-1.03321600
H	-0.55207600	5.27271800	-2.04351700
H	-1.74500200	7.39640100	-1.62313400
H	-1.90306900	8.29610900	0.68409000
H	-0.88870500	7.04819600	2.57189200
H	0.29167800	4.92474000	2.16413200
H	0.29762100	3.20803100	-1.19364700
C	2.84734900	4.23746100	0.84033500
C	3.33686400	3.33726700	1.98122800
H	4.04197500	3.91454300	2.58661000
H	2.48104800	3.10930600	2.62639700
C	3.97917300	2.02115000	1.47704600
C	3.77525800	1.84611200	-0.03221400
H	3.84764000	0.78594400	-0.29235800
C	4.78136300	2.63992600	-0.88683000
H	5.68820400	2.03395400	-0.98656800
H	4.37386800	2.75772600	-1.89946400
C	5.15781100	3.99925200	-0.29661500
C	3.96151300	4.89962300	0.01090500
H	3.51197200	5.28157600	-0.91308800
H	4.30672800	5.77334500	0.57402100
H	5.83039600	4.51969600	-0.98462900
H	5.73257400	3.84252000	0.62372600
H	5.05008800	1.99274800	1.69343000
H	3.53314400	1.17014500	1.99758700
H	2.22354200	5.03184100	1.24344600
H	2.09584400	1.91260400	-1.27592500

Table 32. Geometric coordinates and thermally corrected M06-2X energies for **101a**.

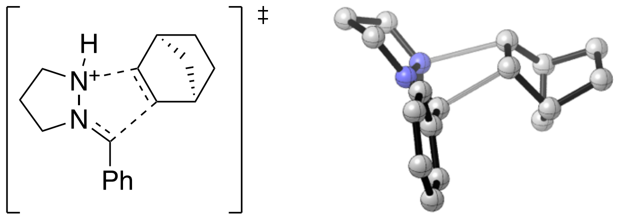

$G_{Acetonitrile}$ = –770.451015626 Hartree

Atom	X	Y	Z
C	0.00000000	0.00000000	0.00000000
C	0.32520100	1.27579600	-0.77378100
C	-0.82243500	2.04012900	-0.73465700
H	-1.07816700	2.82972400	-1.43525400

C	-1.84973500	1.29850700	0.07768500
C	-2.27380900	0.11609200	-0.86020100
C	-1.00485900	-0.78164900	-0.89509400
H	-0.61712900	-0.93157100	-1.90525500
H	-1.20507900	-1.76628100	-0.46460500
H	-2.58069400	0.46443900	-1.84915500
H	-3.12365800	-0.40630400	-0.41262500
C	-0.96072300	0.53934400	1.07682300
H	-0.50110800	1.18705300	1.83043600
H	-1.50019500	-0.25820900	1.59475100
H	-2.68098400	1.88371400	0.47165000
H	1.00566900	1.28401800	-1.62171400
H	0.86227700	-0.57980400	0.33735500
N	0.08200300	3.94869600	0.57488300
N	1.39393000	3.60617500	0.27996000
C	1.73228500	2.32794900	0.46394900
C	3.05432900	1.77283000	0.12844200
C	3.70117500	1.98248800	-1.09783200
C	4.93976900	1.40220000	-1.33847900
C	5.54132500	0.60335100	-0.36625300
C	4.89499900	0.37127600	0.84480000
C	3.65264800	0.94542200	1.08721600
H	3.14638700	0.76052900	2.03105500
H	5.35415800	-0.25979100	1.59819000
H	6.50873400	0.15153400	-0.55957700
H	5.43250600	1.56376900	-2.29130700
H	3.22903100	2.56779200	-1.88057900
H	1.26366100	1.88382400	1.33663800
C	1.92204400	4.53766600	-0.72466700
C	1.21023300	5.83243000	-0.34604300
H	1.15784700	6.53498700	-1.17817300
H	1.72341200	6.31180700	0.49095700
C	-0.16963400	5.32185600	0.07993600
H	-0.85318600	5.25692400	-0.77169700
H	-0.63835700	5.92583800	0.85769100
H	3.00890100	4.57381000	-0.64641400
H	1.63206500	4.19816400	-1.72883300
H	-0.10568800	3.81878700	1.56707100

Table 33. Geometric coordinates and thermally corrected M06-2X energies for **102a**.

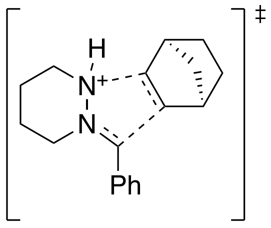

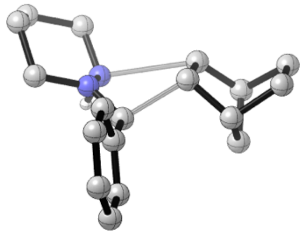

$G_{Acetonitrile}$ = –809.718414090 Hartree

Atom	X	Y	Z
C	0.00000000	0.00000000	0.00000000
C	-0.49011800	-1.27709200	0.67900700
H	-0.96302700	-1.03302800	1.63900300
H	-1.23918800	-1.78716300	0.06702600
C	0.70214700	-2.19997500	0.91022700
N	1.79766300	-1.56609100	1.65809000
N	2.16568200	-0.32210400	1.14150700
C	3.47173100	-0.01820200	1.36792300
C	4.01701100	1.32399000	1.05721500
C	3.87672900	1.92478100	-0.20150100
C	4.42552700	3.17671100	-0.44091500
C	5.12654600	3.83724100	0.56986900
C	5.28672000	3.23828100	1.81558400
C	4.74286000	1.97952700	2.05604800
H	4.87569700	1.50815800	3.02652600
H	5.83868300	3.74589200	2.59934800
H	5.55701000	4.81495000	0.37953100
H	4.31519200	3.63647200	-1.41732300

H	3.35011500	1.40489600	-0.99910400
H	3.86368900	-0.44758900	2.28627500
C	1.09103700	0.64556700	0.84350100
H	0.67182800	0.98108700	1.80347100
H	1.52930500	1.51051000	0.35067400
H	1.56958100	-1.48190400	2.65139000
H	1.10957100	-2.51769500	-0.05751600
H	0.42918500	-3.09888800	1.46832900
H	-0.81431000	0.71820500	-0.12874900
H	0.39775100	-0.23297600	-0.99530900
C	4.53653000	-1.20774600	0.19416200
C	4.09034100	-2.48301900	0.51889900
H	3.22793200	-2.98411200	0.09439400
C	5.16227300	-3.18636600	1.27527700

C	6.18303500	-3.52882600	0.11861700
C	6.75591500	-2.14170300	-0.27462500
C	5.99238200	-1.16887600	0.66688200
C	5.91611200	-2.02139700	1.94551900
H	5.37641100	-1.56654200	2.77968600
H	6.90236000	-2.32853600	2.30350900
H	6.42377500	-0.17013500	0.75234400
H	6.59635200	-1.90144000	-1.32831000
H	7.82948400	-2.08805900	-0.07799300
H	5.71053700	-4.05539800	-0.71382200
H	6.94980900	-4.18718700	0.53584900
H	4.86702800	-4.04242700	1.87999200
H	4.22425200	-0.73207700	-0.73192900

Table 34. Geometric coordinates and thermally corrected M06-2X energies for **103a**.

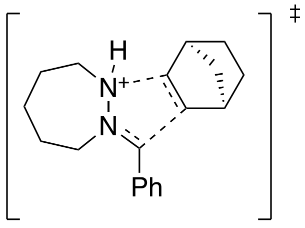

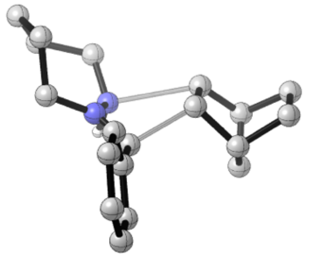

$G_{Acetonitrile}$ = –848.988645215 Hartree

Atom	X	Y	Z
C	0.00000000	0.00000000	0.00000000
N	1.28604400	-0.36930100	-0.61004300
N	1.93039700	-1.52037000	-0.17475700
C	3.27951800	-1.43277500	-0.28119400
C	4.17972500	-2.56288500	0.04286800
C	4.15640700	-3.21868600	1.28155500
C	5.05155400	-4.24690100	1.54297000
C	5.98220000	-4.62603500	0.57454100
C	6.02342200	-3.96787500	-0.65072700
C	5.13251700	-2.93096000	-0.91192000
H	5.17172100	-2.40896200	-1.86475500
H	6.75214200	-4.25507300	-1.40139300
H	6.68229000	-5.42862200	0.78233700
H	5.03123900	-4.74888900	2.50455500

Atom	X	Y	Z
H	3.44569800	-2.91335200	2.04722000
H	3.60063100	-0.85361600	-1.14156600
C	1.17794700	-2.72066500	0.23451700
H	0.60012300	-3.05114800	-0.63735600
H	1.92105900	-3.49035400	0.43057600
C	0.25953700	-2.51248600	1.44120500
H	0.74186400	-1.80580400	2.12780100
H	0.18064200	-3.45864000	1.98553900
C	-1.14516700	-2.03182400	1.05006000
C	-1.14317300	-1.01384300	-0.09906100
H	-1.08641900	-1.51812000	-1.07150700
H	-2.09267200	-0.46999500	-0.10066800
H	-1.62633200	-1.59250600	1.92984700
H	-1.75853300	-2.89112300	0.76071700

H	1.23594200	-0.35235700	-1.62858500	C	4.80780200	1.36930900	-0.73935200
H	0.18484400	0.25354900	1.05013600	H	4.41986500	0.81119900	-1.59552700
H	-0.26633500	0.93080900	-0.50869100	H	5.61681300	2.01516600	-1.09111700
C	3.86046600	0.02255400	0.97249400	H	5.96925300	-0.25966300	0.31466900
C	2.98374500	1.07019600	0.72639200	H	5.61768500	1.29767900	2.50531900
H	2.03277200	1.22907500	1.22204200	H	6.65630500	1.97533100	1.24864800
C	3.72828800	2.15861300	0.02251200	H	4.00997200	3.03465800	2.05660000
C	4.60536900	2.76266000	1.18182800	H	5.08425100	3.67269800	0.81015900
C	5.63950900	1.63995000	1.46810200	H	3.13597600	2.89260300	-0.52292100
C	5.22130300	0.51908100	0.47606300	H	3.76935200	-0.58443900	1.87010500

Table 35. Geometric coordinates and thermally corrected M06-2X energies for **13**.

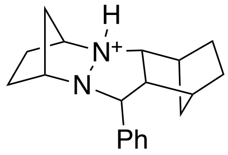

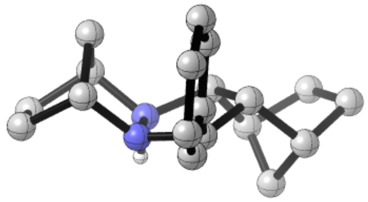

$G_{Acetonitrile}$ = –847.887987041 Hartree

Atom	X	Y	Z	Atom	X	Y	Z
C	0.00000000	0.00000000	0.00000000	H	-2.99892200	-4.62881300	3.13393400
C	-0.22498000	-1.36335100	0.67255100	H	-0.65756600	-4.14034300	2.58065800
C	0.46390000	-1.17596000	2.03108900	H	0.58310600	-4.16594000	-0.98690900
C	1.80017500	-0.81139700	1.38703200	C	1.56592100	-4.40152500	0.90984600
C	1.44670900	0.35332300	0.45381700	C	2.55743300	-3.25473900	1.20968400
H	2.15393900	0.44399400	-0.37686200	H	2.64089900	-2.98957100	2.26600000
H	1.49028700	1.28908200	1.01566500	C	3.87738700	-3.71533700	0.58227100
H	2.68529000	-0.69470600	2.01313800	C	4.40294900	-4.86043500	1.47440100
N	1.99061300	-2.03665200	0.49964900	C	3.43494400	-6.03789700	1.15449800
N	0.65964700	-2.32263700	-0.04340700	C	2.45042400	-5.41552300	0.14879800
C	0.43991600	-3.80284100	0.03700700	C	3.37379300	-4.48280100	-0.65531100
C	-0.97539100	-4.15911300	0.43954900	H	2.84319600	-3.86224500	-1.38966900
C	-1.37617500	-4.26543300	1.77477600	H	4.17666800	-5.01244500	-1.17431900
C	-2.70184500	-4.54221800	2.09378800	H	1.86853100	-6.13994200	-0.42502900
C	-3.64305700	-4.71542200	1.08104900	H	2.92783300	-6.42262900	2.04338700
C	-3.25356000	-4.61236800	-0.25117400	H	3.97154500	-6.87166700	0.69571000
C	-1.92641700	-4.33566200	-0.56792400	H	4.41012700	-4.58695800	2.53366800
H	-1.62320800	-4.25727200	-1.60913700	H	5.43074200	-5.10210100	1.19492700
H	-3.98032300	-4.75335300	-1.04438000	H	4.60074100	-2.91013800	0.42136800
H	-4.67556400	-4.93664400	1.33085200	H	1.16343000	-4.83378700	1.82795800

H	2.60065600	-1.80077900	-0.29105800
H	0.49085200	-2.07578500	2.65156200
H	0.05851900	-0.34337600	2.60981300
H	-1.24907200	-1.72883000	0.66761500
H	-0.10044900	-0.07410600	-1.08334800
H	-0.72228000	0.73260000	0.36792200

Table 36. Geometric coordinates and thermally corrected M06-2X energies for **14a**.

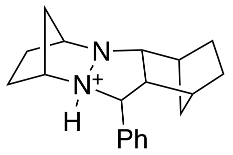

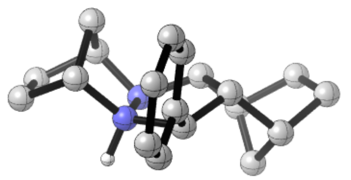

$G_{Acetonitrile}$ = –847.887968736 Hartree

Atom	X	Y	Z
C	0.00000000	0.00000000	0.00000000
C	-0.21955500	-1.35614300	0.68499700
C	0.54518800	-1.20103100	1.99631600
C	1.85803900	-0.84674400	1.28093000
C	1.46480700	0.34700500	0.39866900
H	2.13449500	0.43338400	-0.45901700
H	1.51514100	1.27827300	0.96667300
H	2.75052300	-0.70311500	1.88931500
N	2.06998200	-2.01998500	0.39310700
C	2.60001600	-3.21005000	1.10507100
C	1.61203500	-4.38738700	0.87655900
C	0.46905200	-3.85327100	0.00982200
C	-0.94796600	-4.18836200	0.39860000
C	-1.35392800	-4.28696400	1.73325800
C	-2.68474600	-4.54716400	2.04389500
C	-3.62162800	-4.71451100	1.02589700
C	-3.22503100	-4.62385600	-0.30551300
C	-1.89391400	-4.36179600	-0.61582700
H	-1.58542500	-4.30473300	-1.65753500
H	-3.94820400	-4.76583400	-1.10161600
H	-4.65759700	-4.92439500	1.27091800
H	-2.98907600	-4.62663500	3.08232700
H	-0.63710700	-4.16761700	2.54057300
H	0.62803600	-4.17709400	-1.02350100
N	0.70172100	-2.32399400	-0.06682300
H	0.67176800	-2.06737500	-1.05694100
C	2.48274800	-5.42682900	0.13174700
C	3.48231500	-6.00778300	1.14806900
C	4.45575400	-4.81891300	1.40490800
C	3.91200200	-3.71024700	0.48254500
C	3.39278400	-4.51839600	-0.71595400
H	2.86248200	-3.90153400	-1.45160300
H	4.18016400	-5.07167100	-1.23470800
H	4.61574600	-2.90405800	0.26712600
H	4.47897100	-4.50917500	2.45430800
H	5.47842500	-5.07741100	1.12106000
H	2.98775600	-6.35925900	2.05812400
H	4.00871000	-6.86048800	0.71230400
H	1.89451000	-6.17787900	-0.40174400
H	1.22781600	-4.78850000	1.81652800
H	2.71855400	-2.96943500	2.16823500
H	0.59459800	-2.11111700	2.60031000
H	0.14029600	-0.38429800	2.59767900
H	-1.23283000	-1.75211800	0.69820300
H	-0.16189100	-0.04563700	-1.08186800
H	-0.71708000	0.72038400	0.39992200

Table 37. Geometric coordinates and thermally corrected M06-2X energies for **32b**.

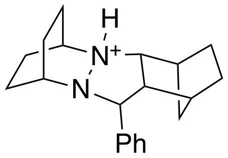

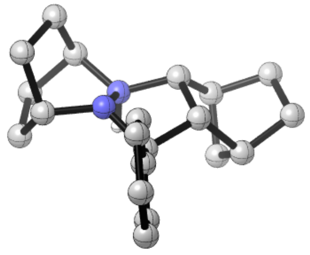

$G_{Acetonitrile}$ = –887.170706672 Hartree

Atom	X	Y	Z
N	0.00000000	0.00000000	0.00000000
N	1.40422600	0.25802600	-0.12067300
C	2.02180000	-0.96292900	0.39628600
C	3.50365500	-0.98386300	0.12927900
C	4.00070700	-0.61140100	-1.12322800
C	5.36835500	-0.64861000	-1.36892000
C	6.24729600	-1.06435500	-0.36915900
C	5.75566000	-1.43727000	0.87774800
C	4.38596300	-1.39111900	1.12810600
H	4.00326400	-1.67516600	2.10562500
H	6.43632800	-1.75928000	1.65903900
H	7.31441000	-1.09450700	-0.56352500
H	5.75076200	-0.35392600	-2.34089000
H	3.31127100	-0.28773100	-1.89961100
H	1.85195300	-1.07339800	1.48405800
C	1.19012600	-2.02718200	-0.37914500
C	-0.16563500	-1.32003300	-0.70911000
H	-0.32882000	-1.09448700	-1.76487900
C	-1.22082200	-2.22099000	-0.06410500
C	-1.26510400	-3.49783900	-0.93593700
C	0.06468000	-4.22919300	-0.57954900
C	0.73901700	-3.26542600	0.41571300
C	-0.48471400	-2.73965800	1.18905400
H	-0.24682600	-1.97899200	1.94356800
H	-1.04230500	-3.53370700	1.69287200
H	1.53732900	-3.71338600	1.01187100
H	0.68959700	-4.41995900	-1.45582600
H	-0.13490100	-5.19126500	-0.10172800
H	-1.35065000	-3.26406400	-2.00110500
H	-2.13538800	-4.09897800	-0.66420700
H	-2.19579500	-1.74801900	0.08430900
H	1.73303300	-2.29414500	-1.28884300
C	1.68917000	1.64584500	0.30003500
C	1.36268300	2.48063900	-0.94739100
H	1.30296900	3.54097000	-0.68182600
H	2.14885900	2.36612600	-1.69653000
C	0.01205900	1.96078400	-1.50441100
C	-0.75428200	1.26573300	-0.37914400
H	-1.76577300	0.96438100	-0.66158000
C	-0.69794300	2.11259000	0.90246300
H	-0.99933700	3.13278300	0.64992100
H	-1.41220600	1.74433800	1.64626000
C	0.75754600	2.07076400	1.44668800
H	1.05949200	3.05048600	1.82350600
H	0.84721100	1.37604100	2.29127500
H	-0.61069000	2.77739100	-1.87889900
H	0.17094100	1.26946900	-2.33691400
H	2.74433600	1.69069900	0.57790100
H	-0.24006000	-0.18145700	0.99108100

Table 38. Geometric coordinates and thermally corrected M06-2X energies for **32a**.

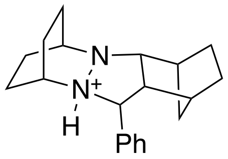

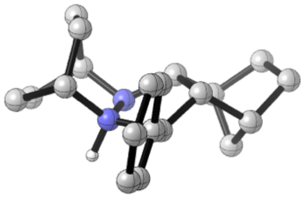

$G_{Acetonitrile}$ = –887.170567730 Hartree

Atom	X	Y	Z
C	0.00000000	0.00000000	0.00000000
C	-1.44903500	-0.18829000	0.36886600
C	-1.88426100	-0.29802700	1.69297600
C	-3.23978200	-0.43178600	1.97709000
C	-4.17376300	-0.45789000	0.94397000
C	-3.74950500	-0.35423600	-0.37806700
C	-2.39373000	-0.22196100	-0.66153500
H	-2.06487700	-0.16162800	-1.69712700
H	-4.47033200	-0.38863800	-1.18823600
H	-5.22942400	-0.56915700	1.16890200
H	-3.56575500	-0.52391300	3.00789100
H	-1.17530600	-0.29226700	2.51521800
H	0.14352500	-0.35906200	-1.02413200
C	1.08868600	-0.61556100	0.87849200
C	2.25103000	0.41299300	0.90994800
H	2.48091300	0.74117300	1.92882400
C	3.43157200	-0.35413800	0.28979700
C	3.86438900	-1.41140700	1.32418400
C	2.71113300	-2.45843600	1.26766800
C	1.74304700	-1.85780100	0.23141200
C	2.72396100	-1.21510500	-0.76727300
H	2.25382800	-0.62140400	-1.55837700
H	3.38746300	-1.94885000	-1.23299200
H	1.01668500	-2.56739700	-0.17280400
H	2.23145900	-2.61690600	2.23774700

Atom	X	Y	Z
H	3.07802300	-3.42781400	0.92190200
H	3.99922900	-0.98299000	2.32212400
H	4.81731800	-1.85816600	1.03134400
H	4.23283400	0.29934700	-0.06232000
N	1.83305900	1.55912800	0.07231200
C	2.17813000	2.90316200	0.60856300
C	1.67568200	3.09171700	2.05174800
C	0.23811100	2.53391400	2.11382200
C	-0.34906300	2.54319200	0.70515600
H	-1.40751300	2.28046300	0.68469100
C	-0.04290700	3.86729700	-0.00455300
H	-0.62342300	3.95495000	-0.92877600
H	-0.36821700	4.68232100	0.64812500
C	1.48190800	3.93586100	-0.28387400
H	1.70698400	3.72055300	-1.33333900
H	1.87208300	4.93311200	-0.06983400
N	0.38241600	1.49202600	-0.11803500
H	0.22211200	1.74368000	-1.09882800
H	0.22247600	1.51615900	2.51810900
H	-0.40311100	3.13786200	2.76064300
H	2.31974500	2.61125500	2.79062900
H	1.68843300	4.16148000	2.28294600
H	3.26521100	2.98047700	0.53083300
H	0.71071900	-0.83680200	1.87907900

Table 39. Geometric coordinates and thermally corrected M06-2X energies for **104a**.

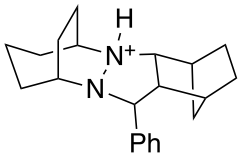

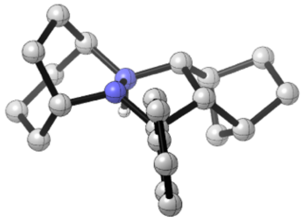

$G_{Acetonitrile}$ = –926.451434947 Hartree

Atom	X	Y	Z

Atom	X	Y	Z

N	0.00000000	0.00000000	0.00000000
N	1.28842500	-0.61976700	0.09577300
C	2.17952100	0.36015800	-0.53590200
C	3.62741000	0.00942600	-0.31560200
C	4.07433100	-0.38666700	0.94873100
C	5.41706600	-0.68460800	1.15066000
C	6.32276300	-0.58187200	0.09522000
C	5.88156500	-0.18676700	-1.16367500
C	4.53422600	0.10235300	-1.36969700
H	4.18859500	0.40396500	-2.35568400
H	6.58284600	-0.10814600	-1.98791400
H	7.37089300	-0.81212800	0.25616900
H	5.75973500	-0.99597100	2.13215000
H	3.36420000	-0.46571400	1.76854800
H	1.98963600	0.42712200	-1.62371400
C	1.70446200	1.66557000	0.15597700
C	0.23444300	1.37390400	0.58000300
H	0.06910100	1.28548400	1.65603600
C	-0.58105000	2.46455000	-0.11709800
C	-0.24722500	3.77648700	0.63045300
C	1.20874800	4.09528500	0.17291200
C	1.55727700	2.90630000	-0.74306900
C	0.20148300	2.66137400	-1.43251500
H	0.19107000	1.80384700	-2.11806400
H	-0.15185200	3.53072400	-1.99348100
H	2.41500300	3.07353800	-1.39858100
H	1.90478400	4.18562100	1.01090700
H	1.24495200	5.03218600	-0.38805000
H	-0.33961000	3.66586400	1.71482800
H	-0.94163000	4.56166300	0.32349100
H	-1.65205200	2.25429900	-0.19493200
H	2.34224400	1.85542400	1.02270100
C	1.21377800	-2.02763300	-0.33189100
C	0.63243800	-2.75342400	0.89009200
H	0.28358400	-3.75203900	0.61725700
H	1.42711600	-2.88235400	1.62842000
C	-0.49691000	-1.90157400	1.52590700
C	-1.11258100	-0.87672300	0.56840200
H	-1.76588200	-0.19007400	1.11562100
C	-1.87176600	-1.46694800	-0.62267000
C	-1.09660100	-2.53657300	-1.40741600
H	-1.57854800	-2.67314600	-2.37970500
H	-1.19832100	-3.49478100	-0.89002900
C	0.40035100	-2.25745600	-1.62711100
H	0.82730400	-3.12224000	-2.14345000
H	0.54742200	-1.41382100	-2.31955400
H	-2.80689800	-1.89273600	-0.24616900
H	-2.16911100	-0.63973700	-1.28373400
H	-1.31296300	-2.53226600	1.89101400
H	-0.10463300	-1.36568000	2.39507300
H	2.25395300	-2.32308600	-0.49320700
H	-0.23081500	0.14493500	-0.99752500

Table 40. Geometric coordinates and thermally corrected M06-2X energies for **104b**.

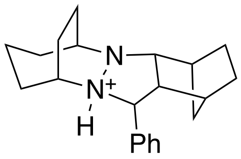

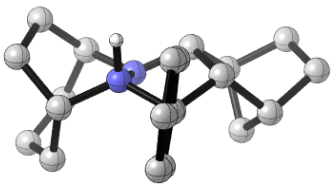

$G_{Acetonitrile}$ = –926.451277625 Hartree

Atom	X	Y	Z
C	0.00000000	0.00000000	0.00000000
C	-1.47903500	-0.17805900	-0.19803800
C	-2.06538400	-0.02209100	-1.45919300
C	-3.42871400	-0.23665300	-1.62914000
C	-4.21866700	-0.59986800	-0.53939400
C	-3.64252300	-0.75467300	0.71831600
C	-2.27560800	-0.55090900	0.88615100
H	-1.82591400	-0.67271300	1.86875400

H	-4.25509600	-1.03320800	1.56924000
H	-5.28388200	-0.75795400	-0.67234300
H	-3.87710900	-0.11024400	-2.60899300
H	-1.46573300	0.28417600	-2.31504500
H	0.26403800	-0.10074300	1.05630300
C	0.68021300	1.24364100	-0.60725100
C	2.06578900	0.73769900	-1.13463100
H	2.15887900	0.78618000	-2.23179600
C	3.07165800	1.64172400	-0.40781900
C	2.91031900	3.05651700	-1.00180400
C	1.56096400	3.54915400	-0.39747800
C	1.07588300	2.33180500	0.41178900
C	2.40658600	1.79324600	0.97059000
H	2.32355500	0.84620400	1.51256900
H	2.91500400	2.52263800	1.60719800
H	0.28942900	2.55557700	1.13694100
H	0.83926800	3.85549800	-1.15999600
H	1.71814500	4.40257700	0.26681000
H	2.90338100	3.04379100	-2.09612300
H	3.74047900	3.69426400	-0.69024900
H	4.09557300	1.25856100	-0.42166500
N	2.11107800	-0.64046100	-0.64713200
C	3.10512900	-1.62028200	-1.14110500
C	2.44443500	-2.68451900	-2.03748300
C	1.20541400	-3.36016100	-1.38787600
C	0.68842000	-2.53391100	-0.20216700
C	1.49397500	-2.72105100	1.09508100
C	2.95464200	-3.15750400	0.89963000
C	3.82656500	-2.19932800	0.08040000
H	4.73416200	-2.72014300	-0.24366800
H	4.14303400	-1.35221300	0.69891400
H	3.40231000	-3.29536500	1.88802200
H	2.96616200	-4.14845000	0.43271400
H	0.97951000	-3.49878600	1.66842900
H	1.44576400	-1.80965800	1.70154800
H	-0.37712400	-2.70972100	-0.02704500
N	0.76710600	-1.10871900	-0.69835000
H	0.40211000	-1.12696500	-1.66916900
H	1.42813000	-4.37145000	-1.04230700
H	0.40766500	-3.46044300	-2.13162300
H	3.19725200	-3.43420300	-2.29545800
H	2.15237700	-2.21448900	-2.98396500
H	3.82194200	-1.05082800	-1.74383300
H	0.05772700	1.65467700	-1.40675400

Table 41. Geometric coordinates and thermally corrected M06-2X energies for **105a**.

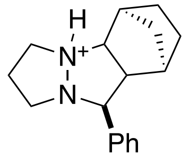

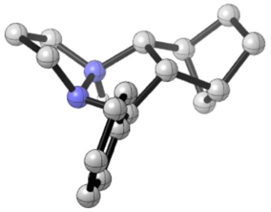

$G_{Acetonitrile}$ = –770.519798553 Hartree

Atom	X	Y	Z
N	0.00000000	0.00000000	0.00000000
N	1.45101500	-0.10062700	0.05016800
C	1.69943900	-1.56699100	0.05513900
C	3.14385700	-1.86502600	-0.28203600
C	3.58020400	-2.17476100	-1.57182600
C	4.93243300	-2.39520100	-1.82213000
C	5.86042200	-2.30281100	-0.78922200
C	5.43398600	-1.98872200	0.49912300
C	4.08338700	-1.77338400	0.74891500
H	3.75070900	-1.53063100	1.75521100
H	6.15147800	-1.91721900	1.30991100
H	6.91259500	-2.47977200	-0.98666800
H	5.25816700	-2.64433700	-2.82673100
H	2.87855200	-2.25489500	-2.39688500
H	1.53300300	-1.88331000	1.09280300
C	0.59223500	-2.19734700	-0.82322900

C	-0.53372900	-1.13609300	-0.87433000
H	-0.71934900	-0.70670300	-1.86114600
C	-1.74964900	-1.83645100	-0.25963800
C	-2.20513100	-2.88245200	-1.30216500
C	-1.10030600	-3.97811800	-1.22209900
C	-0.11779700	-3.40954200	-0.18130400
C	-1.08767600	-2.73427500	0.80581200
H	-0.59504500	-2.19763200	1.62629700
H	-1.79791000	-3.43575600	1.25113200
H	0.57571800	-4.14074200	0.23921700
H	-0.61819600	-4.16385700	-2.18545500
H	-1.51602000	-4.92662400	-0.87420900
H	-2.30807600	-2.44959500	-2.30143400
H	-3.18194600	-3.28058500	-1.01882600
H	-2.54305200	-1.15912000	0.06968800
H	0.95797800	-2.43250400	-1.82412700
C	1.87230900	0.62425300	-1.16288200
C	0.99629400	1.87716500	-1.12484400
H	0.82379500	2.28841900	-2.11986200
H	1.46224900	2.64653600	-0.50788600
C	-0.31174400	1.40354400	-0.46397100
H	-1.16626500	1.34742100	-1.13856200
H	-0.58218000	1.99587900	0.40927100
H	2.93784800	0.83777600	-1.08231400
H	1.70261400	0.03757800	-2.07798600
H	-0.34017300	-0.14327400	0.95735100

Table 42. Geometric coordinates and thermally corrected M06-2X energies for **105b**.

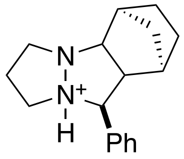

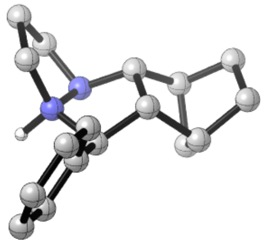

$G_{Acetonitrile}$ = –770.521416805 Hartree

Atom	X	Y	Z
C	0.00000000	0.00000000	0.00000000
N	0.47606000	1.05128500	0.93872100
N	1.91782600	0.99459600	0.84524000
C	2.24889900	-0.49721800	0.85257600
C	1.12905600	-1.07359200	-0.01976200
C	0.43752200	-2.31410400	0.58971900
C	-0.53613100	-2.85343000	-0.47487300
C	-1.64833600	-1.76327800	-0.52212200
C	-1.20886500	-0.75904900	0.56320000
C	-0.53807500	-1.67755300	1.59749900
H	-0.06209000	-1.12778500	2.41499200
H	-1.23026300	-2.40839100	2.02423500
H	-2.00220900	-0.10278200	0.92833800
H	-1.74096400	-1.29436900	-1.50669500
H	-2.62470100	-2.18410600	-0.27160100
H	-0.04961200	-3.00890800	-1.44195700
H	-0.94415500	-3.81544200	-0.15546300
H	1.13959000	-3.05518400	0.97934000
H	1.48002200	-1.27225400	-1.03467000
C	3.69616800	-0.73949600	0.51001800
C	4.13757300	-0.99795100	-0.79053900
C	5.49412600	-1.17462400	-1.04739600
C	6.42043200	-1.09256200	-0.01099700
C	5.98910100	-0.83949100	1.28896200
C	4.63339800	-0.66784400	1.54609600
H	4.29771900	-0.49530100	2.56711000
H	6.70487500	-0.78745100	2.10258700
H	7.47645000	-1.23646200	-0.21455600
H	5.82583600	-1.38479800	-2.05882200
H	3.43332500	-1.08204800	-1.61297600
H	2.07165700	-0.78183500	1.89460700
H	2.30192200	1.43214800	1.68986900
C	2.33579200	1.79735100	-0.37191500
H	3.32037200	2.22573500	-0.18446600

H	2.41349100	1.09617900	-1.20371900
C	1.19589200	2.81265000	-0.55178000
C	0.15783300	2.42579500	0.53072800
H	-0.87481100	2.45341700	0.17951200
H	0.23369500	3.07672900	1.40610200
H	0.77839700	2.73016600	-1.55658700
H	1.54100100	3.83862600	-0.41835800
H	-0.19733500	0.42953200	-0.99233500

Table 43. Geometric coordinates and thermally corrected M06-2X energies for **27c**.

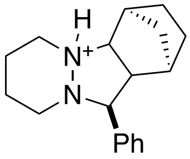

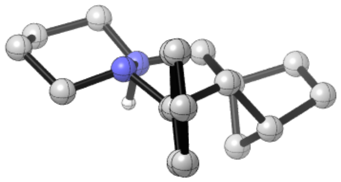

$G_{Acetonitrile}$ = –809.810746310 Hartree

Atom	X	Y	Z
C	0.00000000	0.00000000	0.00000000
C	-1.52437200	-0.09676800	-0.09714800
H	-1.97145900	-0.10312600	0.90592400
H	-1.94291400	0.76535800	-0.62299200
C	-1.93252300	-1.36209100	-0.84042100
N	-1.29614100	-2.54278700	-0.17470100
N	0.14308700	-2.43185400	-0.21450000
C	0.61069700	-3.75150600	0.25213700
C	2.07960000	-3.93802900	-0.02805100
C	2.60178300	-3.62975100	-1.28729900
C	3.95164200	-3.83299900	-1.54909400
C	4.78538700	-4.35148000	-0.55844800
C	4.26821800	-4.65981200	0.69559700
C	2.91730200	-4.44741900	0.96253100
H	2.51428200	-4.68014900	1.94556900
H	4.91522700	-5.05997000	1.46926700
H	5.83826800	-4.51275100	-0.76558900
H	4.35536300	-3.58952200	-2.52661200
H	1.94797800	-3.22271400	-2.05457400
H	0.44341600	-3.83860300	1.34136200
C	-0.32654400	-4.72398500	-0.49285700
C	-1.59898800	-3.88949200	-0.79926800
H	-1.77941700	-3.69663100	-1.85869700
C	-2.72676000	-4.64440100	-0.08970200
C	-2.94817300	-5.93045000	-0.91887500
C	-1.69359700	-6.79500800	-0.58906500
C	-0.88007000	-5.88700300	0.35257400
C	-2.00518500	-5.20486600	1.15267600
H	-1.65667700	-4.44862100	1.86759300
H	-2.62679300	-5.91598900	1.70303600
H	-0.11162900	-6.40348700	0.93214200
H	-1.12681400	-7.07537300	-1.48048800
H	-1.97907200	-7.71666200	-0.07650400
H	-3.05307000	-5.71875200	-1.98684600
H	-3.86737100	-6.42204700	-0.59310000
H	-3.63622800	-4.05893700	0.07484400
H	0.16099500	-5.06791300	-1.40825500
C	0.57905200	-1.28419400	0.58586000
H	0.27303400	-1.41369100	1.64115800
H	1.67102800	-1.28822800	0.55084200
H	-1.61882400	-2.56768900	0.81063400
H	-1.55921200	-1.37449900	-1.86730200
H	-3.01161100	-1.53668400	-0.83510500
H	0.28728600	0.85018500	0.62339500
H	0.42867500	0.15763300	-0.99508700

Table 44. Geometric coordinates and thermally corrected M06-2X energies for **27a**.

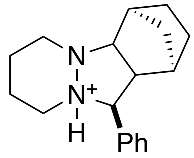

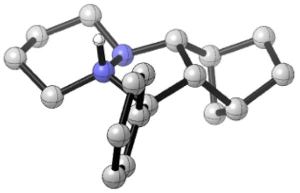

$G_{Acetonitrile}$ = –809.809889903 Hartree

Atom	X	Y	Z
C	0.00000000	0.00000000	0.00000000
C	-1.52539700	-0.02987400	-0.12014900
H	-1.97980200	-0.01400600	0.87596400
H	-1.88061200	0.85014700	-0.66217300
C	-1.97297000	-1.29489900	-0.84558700
H	-1.64689100	-1.28310700	-1.90223600
H	-3.06075700	-1.39751200	-0.83546600
N	-1.44116900	-2.47454400	-0.15907200
C	-1.81714200	-3.77836800	-0.73826700
C	-0.64523500	-4.73124300	-0.34932800
C	0.39805600	-3.83018700	0.32697700
C	1.85298900	-4.08317200	0.04202600
C	2.30914700	-4.24316100	-1.27127500
C	3.66292900	-4.43460300	-1.52320900
C	4.56943800	-4.47749700	-0.46484700
C	4.12170700	-4.32322800	0.84388500
C	2.76729900	-4.11968300	1.09583100
H	2.41729600	-4.00000900	2.11839300
H	4.82430400	-4.36174500	1.66964700
H	5.62442900	-4.63614000	-0.66249600
H	4.01032500	-4.56060800	-2.54328100
H	1.60954500	-4.23677800	-2.10601100
H	0.21842400	-3.78107700	1.40577300

Atom	X	Y	Z
N	-0.00217300	-2.45034600	-0.17585800
C	0.51682200	-1.29337200	0.61821100
H	0.13385400	-1.44500200	1.63082100
H	1.60695000	-1.37078400	0.61492100
H	0.35632700	-2.35916100	-1.14546000
C	-1.32594400	-5.74945800	0.59064300
C	-2.19850800	-6.66027200	-0.29384100
C	-3.35207800	-5.71479500	-0.74684700
C	-3.03003700	-4.39046600	-0.02395500
C	-2.39427500	-4.88620200	1.28525700
H	-1.99555300	-4.07434900	1.90089600
H	-3.08273500	-5.48197100	1.89071400
H	-3.87596100	-3.70513400	0.06866300
H	-3.39836500	-5.59229400	-1.83331100
H	-4.32349000	-6.09327600	-0.42124600
H	-1.63888200	-7.08212000	-1.13344600
H	-2.58512700	-7.49589000	0.29463200
H	-0.62365600	-6.28374800	1.23540400
H	-0.20589100	-5.22364100	-1.22056000
H	-1.95643900	-3.68995800	-1.82672900
H	0.33055500	0.83739800	0.61985600
H	0.45722300	0.13869500	-0.98850700

Table 45. Geometric coordinates and thermally corrected M06-2X energies for **106a**.

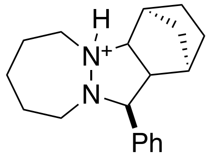

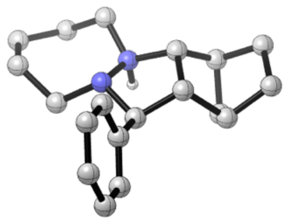

$G_{Acetonitrile}$ = –849.078339842 Hartree

Atom	X	Y	Z	Atom	X	Y	Z
C	0.00000000	0.00000000	0.00000000	H	0.90062000	-2.85677100	2.74045700
N	0.93223800	-0.96130900	0.68401800	H	0.24043700	-4.48896100	2.59121000
N	2.31839400	-0.56073400	0.66010400	H	2.82701200	-4.48433900	1.89501700
C	3.03541400	-1.76058000	1.14963800	H	2.03565300	-5.39847800	-0.52114500
C	4.51342000	-1.65969900	0.87249000	H	1.28244000	-6.16439500	0.87681300
C	4.96941900	-1.26660100	-0.38929800	H	-0.09936400	-4.45488800	-1.11437500
C	6.33345800	-1.20881200	-0.64922800	H	-0.80538200	-5.27758000	0.27282300
C	7.25015100	-1.54923800	0.34521600	H	-1.06490900	-2.90459100	0.88958200
C	6.79957900	-1.94002600	1.60204200	H	2.88702000	-3.17850800	-0.47925800
C	5.43244000	-1.98937600	1.86688900	C	2.57209500	0.65292600	1.45140700
H	5.08113300	-2.28864100	2.85166300	H	2.10501500	0.57611200	2.45070500
H	7.50970300	-2.20171800	2.37968500	H	3.65444700	0.64893600	1.60369100
H	8.31462600	-1.50615500	0.13906300	C	2.16843500	1.92855400	0.71389800
H	6.68385100	-0.89823200	-1.62821600	H	2.42998600	1.78168000	-0.33983500
H	4.25146700	-0.99753900	-1.16017700	H	2.79873400	2.74840300	1.07023400
H	2.88474100	-1.86450500	2.24014900	C	0.69929400	2.35047500	0.85692400
C	2.32281000	-2.91391000	0.41795700	C	-0.32615400	1.20569600	0.87554900
C	0.92509100	-2.34906600	0.06853800	H	-0.48573900	0.84702500	1.90204300
H	0.74090000	-2.20221000	-0.99765100	H	-1.29919000	1.58864100	0.55467700
C	-0.05295900	-3.30054800	0.76239100	H	0.45936800	3.03567500	0.03829200
C	0.01010800	-4.62133700	-0.03876900	H	0.57014700	2.92495400	1.77945500
C	1.39782800	-5.21573400	0.34745400	H	0.61323600	-1.05231800	1.66584900
C	1.98807100	-4.14443600	1.28367800	H	0.48401200	0.25825000	-0.94542300
C	0.72695400	-3.68152400	2.03786800	H	-0.90172000	-0.58256300	-0.20766300

Table 46. Geometric coordinates and thermally corrected M06-2X energies for **106b**.

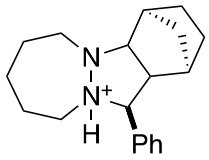

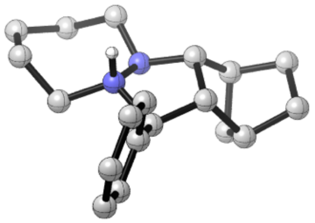

$G_{Acetonitrile}$ = –849.077816290 Hartree

Atom	X	Y	Z	Atom	X	Y	Z
C	0.00000000	0.00000000	0.00000000	C	0.92573600	2.28910600	0.80191000
C	-0.20160600	1.24796400	0.85799800	C	2.35083700	1.72367300	0.70372100

C	2.60947200	0.42702800	1.46556500
H	2.06379200	0.35674700	2.41031400
H	3.67818100	0.29244500	1.65186100
N	2.20185200	-0.78135600	0.67149200
C	2.81054300	-2.08628200	1.18181700
C	4.28680600	-2.12707300	0.89830100
C	4.75882100	-2.17529500	-0.41808700
C	6.12537800	-2.18827700	-0.67252800
C	7.03113000	-2.16575300	0.38730200
C	6.56853700	-2.12373100	1.69903700
C	5.19924800	-2.09709800	1.95373300
H	4.83795700	-2.06464300	2.97891700
H	7.27085400	-2.11156200	2.52584700
H	8.09749000	-2.18545000	0.18806000
H	6.48442000	-2.22691200	-1.69559400
H	4.06233300	-2.22109800	-1.25453000
H	2.62277600	-2.05962600	2.26011800
C	1.93072400	-3.14420800	0.50580100
C	0.63613100	-2.38822500	0.09418000
H	0.50506000	-2.32296800	-0.99691600
C	-0.47826500	-3.18588200	0.78703500
C	-0.56348600	-4.54930500	0.06951500
C	0.71039600	-5.29744700	0.56398200
C	1.40851100	-4.24804600	1.44925400
C	0.20047900	-3.56160500	2.11469600
H	0.44864300	-2.69084800	2.72898800
H	-0.39597200	-4.25678000	2.71169600
H	2.17636800	-4.65382100	2.11257900
H	1.34852600	-5.64013500	-0.25541500
H	0.44339200	-6.17374300	1.15965100
H	-0.59446700	-4.44014600	-1.01901900
H	-1.47249500	-5.07605400	0.36812300
H	-1.42770800	-2.64906200	0.85544600
N	0.78565000	-1.03981200	0.68074500
H	2.45387200	-3.56931100	-0.35499900
H	2.54694500	-0.64352400	-0.29642800
H	2.63184400	1.56375000	-0.34655100
H	3.05976600	2.46897400	1.07545400
H	0.77184800	2.95313800	-0.05442700
H	0.85022900	2.92196300	1.69144800
H	-0.36158700	0.90434400	1.88582600
H	-1.13357200	1.73163400	0.55098900
H	0.42814500	0.23519900	-0.99122000
H	-0.96910300	-0.47292500	-0.17912300

Table 47. Geometric coordinates and thermally corrected M06-2X energies for **15**.

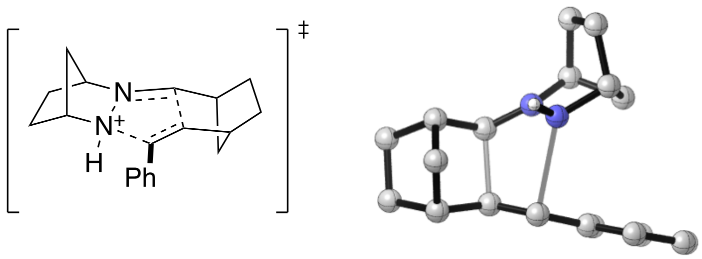

$G_{Acetonitrile}$ = –847.830516418 Hartree

Atom	X	Y	Z
C	0.00000000	0.00000000	0.00000000
C	1.36627000	-0.03596600	-0.71627800
H	1.27468400	-0.07206500	-1.80379700
N	2.34573700	0.82621900	-0.36683400
N	2.84437600	0.79220900	0.92761200
H	2.11495200	1.02643400	1.60566100
C	3.94473900	1.79682100	0.91382800
C	4.58805900	1.47453100	-0.43803000
C	3.26170000	1.64347500	-1.18818200
C	2.89945900	3.10495000	-0.84046300
H	3.47312400	3.79201500	-1.46791100
H	1.83750600	3.30514100	-0.99453500
C	3.33130600	3.19288400	0.65702800
H	4.08153700	3.97043300	0.81924100

H	2.49319900	3.39861900	1.32916200
H	3.20725100	1.35451100	-2.23756000
H	5.31649300	2.23330200	-0.73099900
H	5.02712000	0.48152700	-0.50376300
H	4.55718800	1.68626300	1.80823900
C	-0.99113400	-0.80516300	-0.86224900
C	-0.62134200	-2.28319400	-0.54739700
C	0.47604600	-2.16177600	0.52613200
C	1.81505100	-1.81670000	-0.12996000
C	2.93791600	-1.72596600	0.70704100
C	4.31433200	-1.78995700	0.26205200
C	4.66234100	-1.94873400	-1.09133800
C	5.99630400	-1.95764700	-1.47772600
C	7.00257000	-1.80980300	-0.52254300
C	6.67304800	-1.66201200	0.82614200
C	5.34180400	-1.65472700	1.21371000
H	5.07982700	-1.53463000	2.26220300
H	7.45593600	-1.55672800	1.56977700
H	8.04399200	-1.81936600	-0.82782100
H	6.25489200	-2.08689900	-2.52355100
H	3.89279100	-2.07142100	-1.84711400
H	2.78161100	-1.74405200	1.78439300
H	1.99476300	-2.25506100	-1.11177000
C	0.08074000	-0.87316100	1.25705200
H	0.81423800	-0.55400200	2.00375800
H	-0.89254400	-0.95346400	1.75004900
H	0.55804100	-3.04589600	1.16304700
H	-0.27070500	-2.82888100	-1.42811400
H	-1.48177800	-2.82801100	-0.15204400
H	-0.91638400	-0.55889300	-1.92518500
H	-2.01370900	-0.57931200	-0.55039200
H	-0.29511400	1.04280500	0.15597200

Table 48. Geometric coordinates and thermally corrected M06-2X energies for **107**.

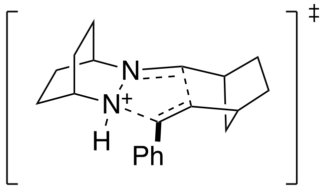

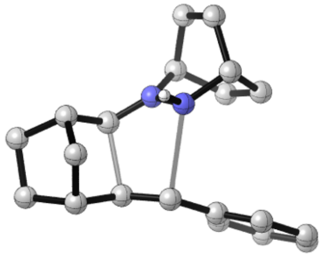

$G_{Acetonitrile}$ = –887.117248914 Hartree

Atom	X	Y	Z
C	0.00000000	0.00000000	0.00000000
C	1.29501700	0.16848800	-0.81593300
H	1.11390100	0.20517400	-1.89127900
N	2.24427000	1.05986500	-0.47785600
C	2.93720900	1.99293300	-1.38808700
C	4.43238200	1.67950600	-1.43723400
C	5.00382400	1.77122200	-0.00204100
C	3.84537900	2.00398300	0.97358600
H	4.16981500	1.91374800	2.01248400
C	3.18644600	3.36384500	0.69326500
H	2.33944300	3.50970200	1.37153900
H	3.90544100	4.16019700	0.90318200
C	2.72384400	3.39380700	-0.78649200
H	1.67167500	3.67777000	-0.86490900
H	3.30378100	4.11272000	-1.37205200
N	2.83939400	0.93080600	0.76156700
H	2.12380300	0.94679400	1.49139200
H	5.54693800	0.86090600	0.25753200
H	5.70144100	2.60803400	0.09521900
H	4.58946000	0.68507600	-1.85777700
H	4.91102900	2.39952700	-2.10701400
H	2.46075800	1.89292200	-2.36504600
C	-0.98915600	-0.76775400	-0.89699700
C	-0.46376000	-2.22987700	-0.82711900
C	0.66652800	-2.16840500	0.21931500
C	1.96857700	-1.67182100	-0.41549100

C	3.07622300	-1.55767100	0.42932600
C	4.47520400	-1.62313200	0.04451400
C	4.89447600	-1.69885300	-1.29350200
C	6.24696300	-1.76754500	-1.60513000
C	7.20010100	-1.78128600	-0.58781800
C	6.79792900	-1.73580700	0.74839200
C	5.44897000	-1.65532400	1.05959500
H	5.13140000	-1.61399500	2.09887800
H	7.53779000	-1.76612700	1.54157300
H	8.25504200	-1.84317300	-0.83477500
H	6.55962200	-1.82327200	-2.64278500
H	4.16314100	-1.71263300	-2.09694900
H	2.89742000	-1.63321700	1.50103600
H	2.16548800	-1.98759400	-1.43984400
C	0.21807700	-1.01526000	1.12561900
H	0.95894700	-0.73073600	1.87879100
H	-0.71938300	-1.23377500	1.64564700
H	0.82066700	-3.11932000	0.73525900
H	-0.10051100	-2.59407900	-1.79237800
H	-1.24942300	-2.91471200	-0.50041800
H	-1.03103400	-0.37111600	-1.91512500
H	-1.99536700	-0.68980700	-0.47815900
H	-0.35759100	0.98818700	0.30900500

Table 49. Geometric coordinates and thermally corrected M06-2X energies for **108**.

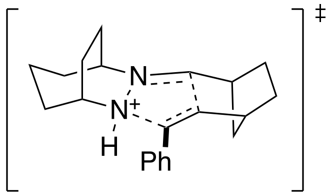

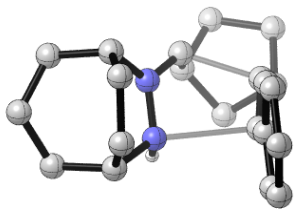

$G_{Acetonitrile}$ = –926.394239564 Hartree

Atom	X	Y	Z
C	0.00000000	0.00000000	0.00000000
C	1.29399900	0.03013200	-0.83048300
H	1.10529700	0.06835800	-1.90372900
N	2.34899400	0.80186600	-0.51351500
C	3.08291100	1.64705000	-1.48137200
C	4.59189300	1.40265600	-1.41319400
C	5.12181700	1.34870800	0.03981900
C	4.00364500	1.53696600	1.07021400
C	3.53590700	3.00026600	1.20774400
C	3.50981600	3.78343800	-0.10933900
C	2.70933200	3.12266700	-1.23598700
H	2.88908700	3.67070100	-2.16743500
H	1.63264100	3.18081600	-1.03655700
H	3.10586100	4.78292000	0.07642600
H	4.54134700	3.93525900	-0.44843100
H	4.21512800	3.50721400	1.90145900
H	2.54509700	3.01384300	1.68225700
H	4.34435000	1.17711400	2.04590800
N	2.88657100	0.62367700	0.73570300
H	2.14880000	0.68543200	1.43880800
H	5.87342800	2.12432500	0.21236700
H	5.61042700	0.38718200	0.21194700
H	5.07923800	2.20126100	-1.98094800
H	4.82370300	0.46469400	-1.91941700
H	2.70188800	1.34352500	-2.46030500
C	-1.06993600	-0.67198300	-0.88190900
C	-0.69811600	-2.17987500	-0.79931800
C	0.43320500	-2.22787100	0.24792900
C	1.77845600	-1.88484700	-0.39124400
C	2.89515400	-1.84014100	0.44415200
C	4.28294000	-1.97816000	0.03315200
C	4.67622300	-1.97370400	-1.31355200
C	6.01814800	-2.09929200	-1.65508400
C	6.98364600	-2.24492200	-0.66001100

C	6.60403400	-2.27525400	0.68338400
C	5.26691900	-2.13922500	1.02534800
H	4.96758700	-2.15803600	2.07063200
H	7.35246800	-2.40642400	1.45785200
H	8.02929100	-2.34995500	-0.93071800
H	6.31240000	-2.09338000	-2.69980500
H	3.93332600	-1.87266200	-2.10031200
H	2.72622500	-1.91535900	1.51769100
H	1.94127000	-2.22693300	-1.41294500
C	0.11128300	-1.02103100	1.13804300
H	0.87699000	-0.81286100	1.89113300
H	-0.84504800	-1.13077000	1.65804100
H	0.48218700	-3.18197000	0.77897300
H	-0.37212100	-2.58546400	-1.76150900
H	-1.55119900	-2.77735500	-0.47063500
H	-1.07942900	-0.28372300	-1.90406300
H	-2.05924600	-0.48667000	-0.45653200
H	-0.25334300	1.02348100	0.29985800

Table 50. Geometric coordinates and thermally corrected M06-2X energies for **109**.

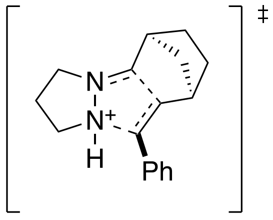

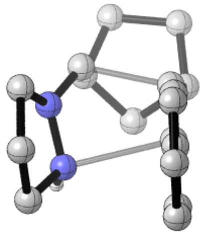

$G_{Acetonitrile}$ = –770.465971525 Hartree

Atom	X	Y	Z
C	0.00000000	0.00000000	0.00000000
C	1.34076500	-0.02902400	-0.74386600
H	1.24386900	-0.10363700	-1.82774000
N	2.35419500	0.79327300	-0.41494600
N	2.82948500	0.73206800	0.87987900
H	2.10343600	0.99560400	1.54609200
C	4.04442100	1.56087000	0.96563100
H	3.79393700	2.59227600	1.23456100
H	4.70174800	1.13764400	1.72824800
C	4.63569400	1.45895800	-0.44400700
C	3.40753600	1.26138500	-1.33884200
H	3.56692000	0.52339400	-2.12823600
H	3.06800000	2.19831200	-1.78907300
H	5.31051600	0.60424400	-0.51500600
H	5.18752000	2.36042700	-0.71183500
C	-1.00805900	-0.80125800	-0.84806100
C	-0.64300800	-2.28212100	-0.54114300
C	0.45461400	-2.17737700	0.53530000
C	1.80703300	-1.88377000	-0.10939100
C	2.91842100	-1.73386600	0.71834000
C	4.30815300	-1.77063200	0.28140200
C	4.67309600	-1.95738700	-1.06218100
C	6.00963200	-1.93712100	-1.43877500
C	7.00524700	-1.73661400	-0.48054800
C	6.65961100	-1.57064200	0.86051300
C	5.32275100	-1.59080200	1.23678300
H	5.05034800	-1.46743100	2.28270000
H	7.43222400	-1.43363200	1.60981200
H	8.04888700	-1.72487000	-0.77782400
H	6.28008000	-2.08936100	-2.47864200
H	3.91372700	-2.12972600	-1.81940100
H	2.76238800	-1.73648600	1.79603500
H	1.98331500	-2.30633900	-1.09825800
C	0.08407000	-0.87582900	1.25766300
H	0.82277400	-0.56481100	2.00159700
H	-0.88883400	-0.93783500	1.75428300
H	0.50574800	-3.05642000	1.18296000
H	-0.29351000	-2.82285700	-1.42538700
H	-1.50712300	-2.82572900	-0.15256300
H	-0.95199900	-0.55319900	-1.91155900

H	-2.02310800	-0.56999500	-0.51663400
H	-0.29537600	1.04158200	0.16541300

Table 51. Geometric coordinates and thermally corrected M06-2X energies for **28**.

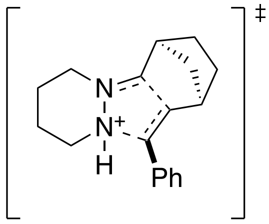

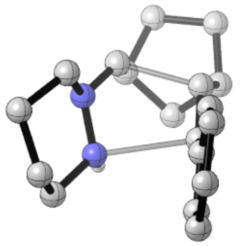

$G_{Acetonitrile}$ = –809.744221366 Hartree

Atom	X	Y	Z
C	0.00000000	0.00000000	0.00000000
C	1.26822900	0.07975300	-0.84894600
H	1.08712400	0.04518300	-1.92242900
N	2.31795400	0.87216200	-0.56793700
N	2.85951700	0.68707100	0.69281500
H	2.09277400	0.68553700	1.36351000
C	3.90023800	1.64732300	1.11836600
C	4.96944800	1.79946300	0.04840300
C	4.29720900	2.18664800	-1.26435400
H	5.02852300	2.28887300	-2.07008800
H	3.78067100	3.14806600	-1.16098400
C	3.30240400	1.10451600	-1.64959500
H	3.82511900	0.16247300	-1.84759200
H	2.72189500	1.38580900	-2.52966800
H	5.51724700	0.85738600	-0.07139000
H	5.68197900	2.56379100	0.36887800
H	3.44411800	2.62079900	1.34066200
H	4.30934900	1.23670000	2.04518400
C	-1.05754100	-0.70306200	-0.87458600
C	-0.61787200	-2.19461600	-0.82934700
C	0.51809800	-2.22566400	0.21609600
C	1.86037900	-1.86005800	-0.41986800
C	2.97350200	-1.75756700	0.40851100
C	4.38314500	-1.87494300	0.03102200
C	4.80442400	-2.17013400	-1.27449300
C	6.15678100	-2.24887100	-1.57717000
C	7.11243200	-2.05186600	-0.57860100
C	6.70826300	-1.79065200	0.72939400
C	5.35423300	-1.70592200	1.02964600
H	5.03661700	-1.50084000	2.04984300
H	7.44755400	-1.65817600	1.51242500
H	8.16871200	-2.11941700	-0.81794900
H	6.46999100	-2.47968900	-2.59012100
H	4.07368700	-2.34698100	-2.05891300
H	2.79883400	-1.78837900	1.48333000
H	2.02815400	-2.17893500	-1.44889900
C	0.16233200	-1.03864700	1.12137600
H	0.91952700	-0.81601200	1.87849100
H	-0.78758000	-1.18797400	1.64320600
H	0.58343800	-3.18326500	0.73926900
H	-0.27807600	-2.56145300	-1.80230700
H	-1.44389500	-2.83653600	-0.51608200
H	-1.10670900	-0.29539700	-1.88784700
H	-2.04394800	-0.57008900	-0.42427300
H	-0.28976500	1.00615100	0.32218500

Table 52. Geometric coordinates and thermally corrected M06-2X energies for **110**.

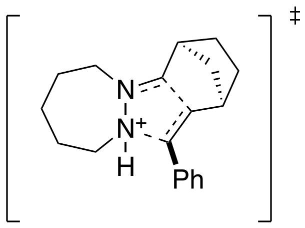

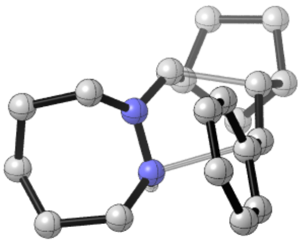

$G_{Acetonitrile}$ = –849.015529657 Hartree

**Atom**	**X**	**Y**	**Z**	**Atom**	**X**	**Y**	**Z**
C	0.00000000	0.00000000	0.00000000	C	-1.56304400	-2.05961500	0.27695000
C	-1.30102100	-0.14496600	0.80717700	C	-2.69337800	-2.06241300	-0.54018500
H	-1.13695900	-0.14857400	1.88463500	C	-4.06383000	-2.13002500	-0.06776500
N	-2.42430400	0.53632600	0.50253700	C	-4.39213700	-1.99276500	1.29108700
N	-2.84642300	0.41790200	-0.80570900	C	-5.71999400	-2.00363700	1.69962600
H	-2.10632900	0.73241900	-1.43371000	C	-6.73955100	-2.16636900	0.76079200
C	-4.15078800	0.99158100	-1.15097000	C	-6.42824800	-2.31681400	-0.59086400
H	-4.30448400	0.70445900	-2.19477100	C	-5.10261900	-2.29121900	-1.00134800
H	-4.90130600	0.44017000	-0.57858200	H	-4.85686600	-2.40293800	-2.05473500
C	-4.29002700	2.52550500	-0.97997800	H	-7.21916800	-2.45434900	-1.32057800
H	-4.18039600	3.00422600	-1.95750700	H	-7.77522200	-2.18478100	1.08443400
H	-5.31041700	2.73912600	-0.64365100	H	-5.96296100	-1.89893100	2.75221700
C	-3.27900800	3.16306700	-0.02077200	H	-3.60691500	-1.87581200	2.03336100
C	-3.33761000	2.64606300	1.41992700	H	-2.55554300	-2.14718700	-1.61714800
C	-3.33775700	1.11482300	1.50123800	H	-1.69004200	-2.44812200	1.28743600
H	-4.33898300	0.70843300	1.35409600	C	-0.00189200	-0.96141600	-1.19626200
H	-2.99425100	0.76584800	2.47723000	H	-0.78708500	-0.79181100	-1.93783700
H	-4.24412300	3.00603400	1.91881300	H	0.96269000	-0.94007300	-1.71216800
H	-2.48355900	3.04469000	1.97666700	H	-0.15832000	-3.16767900	-0.96468100
H	-2.26495500	3.01137700	-0.41242900	H	0.65363000	-2.64975400	1.60325500
H	-3.43625000	4.24525000	-0.01672000	H	1.83745900	-2.61582200	0.30322700
C	1.12480600	-0.61233100	0.85837500	H	1.07878300	-0.29367000	1.90336600
C	0.92417800	-2.14369100	0.67201700	H	2.09304700	-0.29205900	0.46629400
C	-0.19889300	-2.24545300	-0.37934500	H	0.15679200	1.05845200	-0.23969300

Table 53. Geometric coordinates and thermally corrected M06-2X energies for **16-*E***.

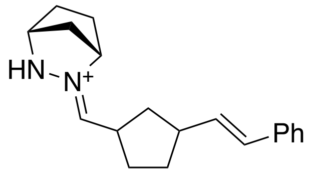

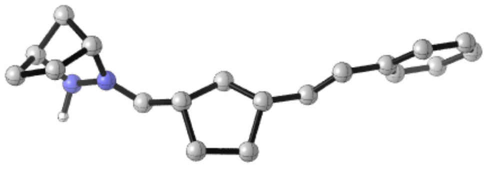

$G_{Acetonitrile}$ = –847.873406303 Hartree

Atom	X	Y	Z	Atom	X	Y	Z
C	0.00000000	0.00000000	0.00000000	H	-2.62900400	-1.85794600	2.14865900
C	1.54148100	-0.00268200	0.08975800	H	-0.42147000	-1.23611400	1.72822300
H	1.99530900	0.32667500	-0.85092100	C	3.05046200	-1.95349500	-0.41802800
H	1.91427300	0.66615800	0.86879700	H	2.96496800	-1.83672600	-1.49971300
C	1.93867300	-1.47663400	0.43498200	N	4.13598600	-2.50552700	-0.01450700
C	0.64734500	-2.29486900	0.18889800	C	4.56024600	-2.87655700	1.36580400
H	0.53064900	-2.50865800	-0.88251300	C	5.36874800	-4.14053600	1.04593400
H	0.64096000	-3.24655200	0.72644300	C	6.22882400	-3.42416500	0.00340800
C	-0.46078800	-1.32452500	0.63371600	C	6.82535900	-2.24737300	0.79680400
C	-1.83367100	-1.75794100	0.22392100	H	7.70885400	-2.56540600	1.35501800
H	-1.99734200	-1.85314400	-0.85070900	H	7.12966700	-1.42590000	0.14246800
C	-2.82631500	-2.00034700	1.08495800	C	5.65242700	-1.85908300	1.74774700
C	-4.19824700	-2.41997900	0.75776900	H	5.91431000	-2.00047000	2.79895700
C	-4.56259700	-2.89741800	-0.50811200	H	5.31099800	-0.82856700	1.62311700
C	-5.87520400	-3.26304600	-0.77451200	H	6.92399900	-4.03352400	-0.57198700
C	-6.84815800	-3.16414500	0.21980200	N	5.15472600	-2.93133100	-0.89788200
C	-6.49704100	-2.70323400	1.48486500	H	5.44642300	-2.13720100	-1.46810800
C	-5.18133200	-2.33898200	1.75036500	H	5.93985600	-4.47719400	1.91260000
H	-4.90922700	-1.97787600	2.73914000	H	4.77267200	-4.95742000	0.63535100
H	-7.24683600	-2.62654900	2.26583400	H	3.69867900	-2.96373700	2.02323800
H	-7.87326300	-3.45134800	0.00860500	H	2.20951900	-1.54291100	1.49216600
H	-6.14199900	-3.63320900	-1.75949500	H	-0.31668800	0.02041000	-1.04877400
H	-3.81211800	-3.00048900	-1.28649300	H	-0.44464600	0.86876400	0.48944400

Table 54. Geometric coordinates and thermally corrected M06-2X energies for **16-Z**.

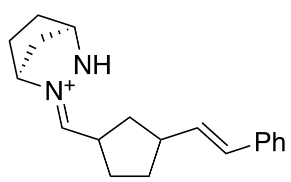

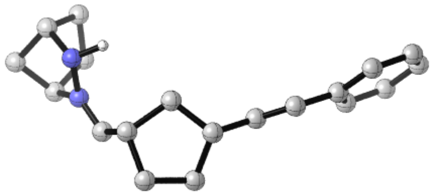

$G_{Acetonitrile}$ = –847.875561638 Hartree

Atom	X	Y	Z
C	0.00000000	0.00000000	0.00000000
C	-1.35176900	0.49747200	-0.51124600
H	-2.00099700	0.91137900	0.26667200
H	-1.20354200	1.28276200	-1.25796200
C	-1.96728600	-0.73839400	-1.20402200
C	-0.74330700	-1.63694900	-1.61411500
H	-0.77535000	-2.60494400	-1.09417800
H	-0.72806400	-1.84086200	-2.68840800
C	0.51953900	-0.86193200	-1.16081500
C	1.66021400	-1.76489700	-0.80720400
H	1.47252000	-2.46136200	0.01234500
C	2.84428200	-1.76286500	-1.42635400
C	3.99714600	-2.62085000	-1.10747500
C	4.11525500	-3.29499500	0.11508500
C	5.20737200	-4.11391800	0.36861900
C	6.20630400	-4.26929800	-0.59202900
C	6.10878600	-3.59283800	-1.80435700
C	5.01494400	-2.77136800	-2.05605500
H	4.94189500	-2.24297800	-3.00342100
H	6.88625200	-3.70172000	-2.55371000
H	7.06162800	-4.90579000	-0.38908000
H	5.28892600	-4.62371600	1.32356500
H	3.35999000	-3.15784400	0.88369200
H	2.98857400	-1.07738000	-2.26275300
H	0.82585400	-0.19313300	-1.97508200
C	-2.86639600	-1.48676500	-0.28671500
H	-2.88842200	-1.29850700	0.78568300
N	-3.64321700	-2.41911800	-0.70271900
C	-4.56348700	-3.28738000	0.08447300
C	-5.66376900	-3.49767400	-0.96125300
C	-4.66831100	-3.96232300	-2.02870900
C	-3.95134300	-5.14126700	-1.34396100
H	-4.53383800	-6.05990800	-1.44466900
H	-2.96610300	-5.32645700	-1.78050700
C	-3.87510000	-4.66501500	0.13798100
H	-4.44245800	-5.31638500	0.80692800
H	-2.85493200	-4.59580400	0.52317400
H	-5.05695700	-4.13510400	-3.03088800
N	-3.76745100	-2.77924200	-2.05014000
H	-2.84141800	-2.95764700	-2.44253900
H	-6.35968300	-4.28217300	-0.66006900
H	-6.20184800	-2.58719400	-1.23045700
H	-4.80914000	-2.80916000	1.03167800
H	-2.56468000	-0.47289300	-2.08625000
H	-0.13156900	-0.62949500	0.89266400
H	0.68671700	0.80655000	0.26545600

Table 55. Geometric coordinates and thermally corrected M06-2X energies for **9-*E***.

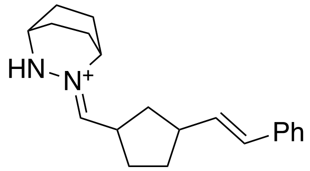

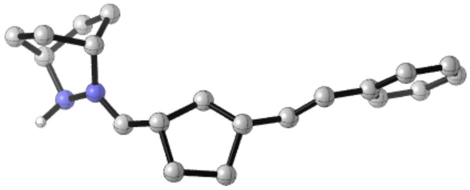

$G_{Acetonitrile}$ = –887.158806330 Hartree

Atom	X	Y	Z
C	0.00000000	0.00000000	0.00000000
C	1.48276000	0.26988900	0.31001800
H	2.01205800	0.70184300	-0.54535800
H	1.58996700	0.97897100	1.13418400
C	2.08045500	-1.10433600	0.74115500
C	0.90912500	-2.12408700	0.60018900
H	0.89021000	-2.54823300	-0.41288800
H	0.99339900	-2.95314300	1.30913900

C	-0.34929100	-1.26262700	0.80429300
C	-1.60624100	-1.94796500	0.36768500
H	-1.64962200	-2.21539900	-0.68922500
C	-2.63366300	-2.21648600	1.17875500
C	-3.89711100	-2.88025200	0.81917800
C	-4.09908700	-3.50788300	-0.41759600
C	-5.31342200	-4.11076300	-0.71533600
C	-6.34936600	-4.10369300	0.21831300
C	-6.15948800	-3.49180500	1.45345000
C	-4.94176400	-2.88848700	1.75006700
H	-4.79699500	-2.41067200	2.71588500
H	-6.95907600	-3.48423300	2.18745800
H	-7.29733300	-4.57726700	-0.01607300
H	-5.45344900	-4.59238100	-1.67802300
H	-3.29837500	-3.53682500	-1.15090200
H	-2.55803900	-1.90583100	2.22201500
H	-0.42347800	-1.00244500	1.86924800
C	3.21996700	-1.53093100	-0.10811000
H	3.28549600	-1.17566100	-1.13741100
N	4.15664500	-2.34539900	0.22961200
C	4.25175900	-3.11436300	1.50266600
C	3.99277000	-4.58168600	1.13056000
H	4.18913400	-5.18290200	2.02320100
H	2.94112300	-4.72316800	0.86675400
C	4.92648300	-4.97161100	-0.04264000
C	5.93412900	-3.84293900	-0.26141600
C	6.68488100	-3.52575200	1.03775600
C	5.67215700	-2.92349000	2.04240200
H	5.72987700	-3.41487300	3.01689000
H	5.85868200	-1.85870900	2.21249900
H	7.13551000	-4.44304200	1.42742600
H	7.50346700	-2.82576500	0.84664100
H	6.61432800	-4.07764300	-1.08276400
N	5.16237900	-2.66082600	-0.71667400
H	5.76824900	-1.84299200	-0.79872500
H	5.46141500	-5.89957300	0.17086700
H	4.35543400	-5.12290800	-0.96222700
H	3.49516700	-2.72543500	2.18333400
H	2.38882800	-1.06111500	1.78967400
H	-0.13689200	-0.21060000	-1.06771300
H	-0.64211700	0.84667000	0.25075000

Table 56. Geometric coordinates and thermally corrected M06-2X energies for **9-*Z***.

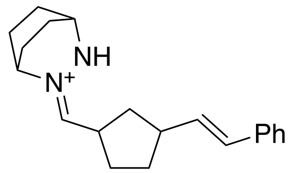

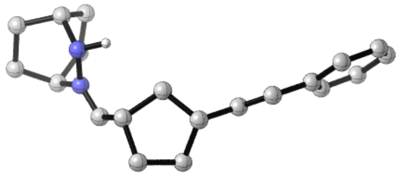

$G_{Acetonitrile}$ = –887.161239870 Hartree

Atom	X	Y	Z
C	0.00000000	0.00000000	0.00000000
C	-1.34084800	0.48398500	-0.55298200
H	-1.99129600	0.94775000	0.19518200
H	-1.17420700	1.22732600	-1.33754300
C	-1.97093800	-0.78003300	-1.18529100
C	-0.76259800	-1.74149500	-1.48017800
H	-0.81681400	-2.64170700	-0.85188200
H	-0.73164900	-2.06579900	-2.52475500
C	0.50840600	-0.94399100	-1.09930000
C	1.64772600	-1.83045700	-0.70268500
H	1.47390000	-2.45700500	0.17406400
C	2.80892900	-1.89592400	-1.36058000
C	3.96237200	-2.74876700	-1.03201700
C	4.08924800	-3.40733800	0.19831100
C	5.18409900	-4.22140000	0.45482100
C	6.17695200	-4.38870600	-0.51047400
C	6.06999100	-3.72953200	-1.73126200
C	4.97296600	-2.91330200	-1.98608600

H	4.89192600	-2.39883600	-2.94047500
H	6.84179900	-3.84803400	-2.48497700
H	7.03379600	-5.02235300	-0.30507000
H	5.27181800	-4.72018500	1.41499700
H	3.33700100	-3.26659700	0.96915800
H	2.93179700	-1.27258300	-2.24771200
H	0.80677000	-0.33637300	-1.96318200
C	-2.94984800	-1.41714400	-0.26408200
H	-3.05787000	-1.09249900	0.76893800
N	-3.71338500	-2.38892000	-0.62136500
C	-4.71935800	-3.05094500	0.25796900
C	-6.05824800	-3.00509100	-0.48474100
H	-6.82432300	-3.40840700	0.18223300
H	-6.32266700	-1.96496600	-0.69360100
C	-5.93868600	-3.82870400	-1.78983400
C	-4.46114400	-4.16271700	-2.00391400
C	-3.96079500	-5.12809200	-0.92289600
C	-4.24630300	-4.49542900	0.46192400
H	-5.04186500	-5.02744200	0.99116100
H	-3.36055100	-4.51609300	1.10199300
H	-4.46008100	-6.09422800	-1.02973200
H	-2.88836000	-5.30454400	-1.05629800
H	-4.27679600	-4.54349500	-3.01004100
N	-3.71381800	-2.88316100	-1.92104900
H	-2.75047800	-2.96241300	-2.24504400
H	-6.50750700	-4.76028600	-1.72783500
H	-6.32063400	-3.26077700	-2.64023500
H	-4.74254100	-2.47912200	1.18717200
H	-2.51296500	-0.55067100	-2.11305200
H	-0.14646000	-0.56706000	0.93093300
H	0.69795800	0.81206800	0.21431700

Table 57. Geometric coordinates and thermally corrected M06-2X energies for **111-*E***.

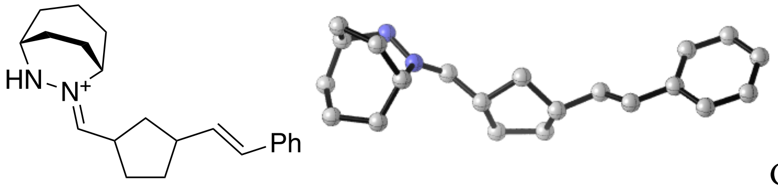

$G_{Acetonitrile}$ = –926.434387360 Hartree

Atom	X	Y	Z
C	0.00000000	0.00000000	0.00000000
C	1.54446300	0.02950800	0.04064000
H	1.95865900	0.36138300	-0.91727300
H	1.92875200	0.71331700	0.80105200
C	1.98281700	-1.43064600	0.37612300
C	0.71094400	-2.28220000	0.13506300
H	0.58443800	-2.48189900	-0.93788900
H	0.73872900	-3.24300900	0.65670600
C	-0.41507600	-1.34978400	0.61389800
C	-1.78162900	-1.81178300	0.21433100
H	-1.95771600	-1.88802300	-0.85985000
C	-2.75520200	-2.10108600	1.08271700
C	-4.11903700	-2.55293900	0.76393200
C	-4.48780600	-3.00941400	-0.50843800
C	-5.79260900	-3.40795300	-0.76572000
C	-6.75320000	-3.36402000	0.24423400
C	-6.39730600	-2.92440400	1.51552800
C	-5.08944000	-2.52676000	1.77170500
H	-4.81385900	-2.18255100	2.76550700
H	-7.13722700	-2.89089600	2.30897100
H	-7.77213500	-3.67734800	0.04039600
H	-6.06260300	-3.76095300	-1.75610400
H	-3.74653200	-3.07017900	-1.30003600
H	-2.54759100	-1.97664900	2.14671400
H	-0.36204100	-1.28235700	1.70928200
C	3.10120000	-1.88651000	-0.48496300
H	3.03358600	-1.66409600	-1.55157500
N	4.13823800	-2.55902600	-0.12591400

C	4.40447800	-2.98771600	1.28853400
H	3.41779700	-3.12907500	1.73069900
C	5.12692700	-4.34064000	1.27687600
C	6.40970900	-4.36144300	0.41457600
C	6.43901000	-3.13369500	-0.49817600
H	7.04978500	-3.34094200	-1.38055900
C	6.96112200	-1.85691600	0.19221300
C	6.63196300	-1.76313300	1.68575900
H	7.02609800	-0.82375500	2.08354700
H	7.16111500	-2.55969000	2.21946400
C	5.13948600	-1.85084100	2.01386800
H	5.00877700	-2.01459600	3.08874400
H	4.63536500	-0.90513100	1.77769400
H	6.57907700	-0.97649400	-0.34303100
H	8.04955800	-1.83334900	0.07818600
N	5.07228500	-3.00648100	-1.05596700
H	5.04279200	-2.39167600	-1.86686600
H	6.41073900	-5.26191400	-0.20231900
H	7.31243000	-4.38762300	1.02899400
H	4.42373800	-5.09367700	0.91153100
H	5.34245100	-4.58972900	2.32013500
H	2.25171500	-1.49041100	1.43332100
H	-0.35016300	0.03994600	-1.03743500
H	-0.44917900	0.84539000	0.52481500

Table 58. Geometric coordinates and thermally corrected M06-2X energies for **111-*Z***.

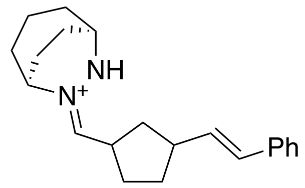

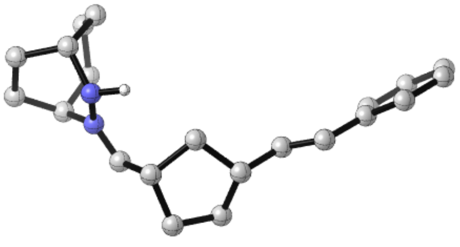

$G_{Acetonitrile}$ = –926.436995221 Hartree

Atom	X	Y	Z
C	0.00000000	0.00000000	0.00000000
C	-1.30679700	0.58717400	-0.53213300
H	-1.94214600	1.03857100	0.23622500
H	-1.09305100	1.36482300	-1.27096300
C	-1.98967800	-0.60322900	-1.24220500
C	-0.81511300	-1.56941100	-1.64657200
H	-0.91489600	-2.54073700	-1.14142400
H	-0.78721700	-1.75904000	-2.72375200
C	0.48493800	-0.87856800	-1.16271000
C	1.56492900	-1.85317900	-0.80893100
H	1.32780500	-2.54611700	0.00061200
C	2.75166700	-1.91269700	-1.41997500
C	3.85272200	-2.84044600	-1.11406600
C	3.91030400	-3.57663500	0.07672800
C	4.95705200	-4.45751300	0.31364000
C	5.97045600	-4.61463000	-0.63153500
C	5.93299900	-3.87822400	-1.81173800
C	4.88427900	-2.99530700	-2.04682200
H	4.85855100	-2.41958500	-2.96862700
H	6.72176400	-3.98843700	-2.54897500
H	6.78948000	-5.30105600	-0.44216400
H	4.99085900	-5.01689800	1.24332100
H	3.14348000	-3.44262300	0.83435800
H	2.94101300	-1.22261000	-2.24361800
H	0.84277400	-0.21861500	-1.96317600
C	-2.94897000	-1.28970000	-0.33592700
H	-3.00325400	-1.04518400	0.72274300
N	-3.74947500	-2.21764300	-0.73295600
C	-4.70922400	-2.90011700	0.19051800
H	-4.77942100	-2.22686600	1.04791600
C	-6.07321700	-2.99075600	-0.50340200
C	-5.99496100	-3.59760300	-1.92644600
C	-4.54381400	-3.83062900	-2.35342500
H	-4.49410300	-3.89467000	-3.44364400

C	-3.91007200	-5.09985100	-1.75403500
C	-4.34660200	-5.40072000	-0.32003700
H	-3.81329800	-6.28190800	0.04756700
H	-5.40969300	-5.66805700	-0.31596700
C	-4.12029100	-4.24334400	0.65130100
H	-4.58882300	-4.47892600	1.61270000
H	-3.05032900	-4.10480500	0.85177500
H	-2.81629300	-5.01299400	-1.81345800
H	-4.18541900	-5.94695500	-2.39079400
N	-3.79764800	-2.58804300	-2.06429500
H	-2.85287700	-2.59614400	-2.44384500
H	-6.46059800	-2.91177300	-2.63693000
H	-6.53924000	-4.54348500	-1.98475300
H	-6.50169900	-1.98627700	-0.54993900
H	-6.72424200	-3.58245500	0.14668800
H	-2.55678800	-0.28784200	-2.12858000
H	-0.18888200	-0.62949300	0.88221500
H	0.73142200	0.75843500	0.28726900

Table 59. Geometric coordinates and thermally corrected M06-2X energies for **112-*E***.

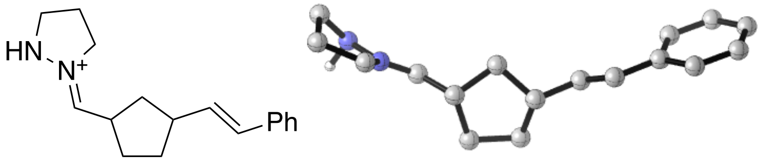

$G_{Acetonitrile}$ = –770.502364254 Hartree

Atom	X	Y	Z
C	0.00000000	0.00000000	0.00000000
C	1.53674700	0.00225400	0.14527600
H	2.02524300	0.33456500	-0.77665400
H	1.87969500	0.66819400	0.94005700
C	1.92643100	-1.47237200	0.50694400
C	0.64108700	-2.29389500	0.22327700
H	0.55780300	-2.51436400	-0.84965900
H	0.61639500	-3.24117400	0.76766500
C	-0.48110700	-1.31988500	0.62605400
C	-1.83683500	-1.76275400	0.17171400
H	-1.96683400	-1.84921000	-0.90813000
C	-2.84966600	-2.02892300	1.00191900
C	-4.20620400	-2.46508400	0.63558800
C	-4.54341500	-2.89234000	-0.65560800
C	-5.84242100	-3.27974300	-0.95499600
C	-6.82890000	-3.25294200	0.03051400
C	-6.50505700	-2.84098700	1.31938900
C	-5.20289300	-2.45422300	1.61775400
H	-4.95241400	-2.13190600	2.62545500
H	-7.26550000	-2.82065700	2.09366500
H	-7.84340200	-3.55698600	-0.20655400
H	-6.08791200	-3.60955200	-1.95961200
H	-3.78324900	-2.93733700	-1.43017100
H	-2.68220000	-1.89830800	2.07226200
H	-0.47832300	-1.22596100	1.72062400
C	3.04373300	-1.95791900	-0.32227200
H	2.96616700	-1.90618500	-1.40851500
N	4.13917700	-2.46581400	0.11695700
C	4.48640300	-2.77289400	1.54037400
C	5.88429000	-3.38328500	1.42391700
C	5.89703200	-3.91091000	-0.01558500
N	5.13205800	-2.91506400	-0.78787400
H	5.72014100	-2.10635800	-0.99941400
H	5.38276000	-4.87193900	-0.09762900
H	6.89307900	-4.00796100	-0.44741200
H	6.04444100	-4.16628000	2.16583100
H	6.64991800	-2.61503900	1.55981500
H	4.44729800	-1.85265500	2.12520500
H	3.74320100	-3.47980100	1.91729500
H	2.17174000	-1.54583300	1.57015900
H	-0.27898300	0.01405700	-1.05945100
H	-0.46123200	0.87178700	0.46835100

Table 60. Geometric coordinates and thermally corrected M06-2X energies for **112-*Z***.

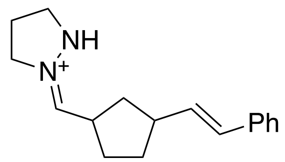

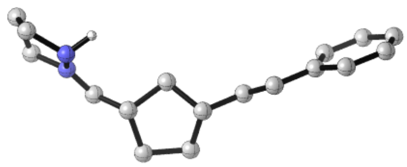

$G_{Acetonitrile}$ = –770.504335784 Hartree

Atom	X	Y	Z	Atom	X	Y	Z
C	0.00000000	0.00000000	0.00000000	H	5.91889100	-3.62336800	2.37400600
C	-1.52644900	0.05784300	-0.21287100	H	3.66695300	-2.85091000	1.76247500
H	-2.03334100	0.54198500	0.62849900	H	2.59839900	-2.24992000	-1.85638000
H	-1.79148400	0.62745100	-1.10612400	H	0.44223600	-1.39083900	-1.59927300
C	-1.98534200	-1.41844300	-0.39266200	C	-3.13602700	-1.78869800	0.45557600
C	-0.73419500	-2.27526200	-0.03023300	H	-3.28942100	-1.31532000	1.42491300
H	-0.66307400	-2.41616100	1.05640600	N	-3.98432400	-2.70793200	0.15484500
H	-0.73888400	-3.26270900	-0.50202700	C	-5.18399500	-3.09937700	0.95827000
C	0.43267700	-1.38791900	-0.50074000	C	-5.58342200	-4.42620400	0.32366400
C	1.76108500	-1.85054100	0.01006600	C	-5.15537900	-4.21379700	-1.13170300
H	1.88330500	-1.82454300	1.09383000	N	-3.91090600	-3.42365800	-1.04941700
C	2.75761100	-2.26752100	-0.77703400	H	-3.07912100	-4.01282500	-1.01152000
C	4.08433900	-2.74356400	-0.35514000	H	-5.89509200	-3.63022200	-1.68559200
C	4.41162800	-2.99266100	0.98466100	H	-4.95685900	-5.13580400	-1.67808000
C	5.68114600	-3.43358600	1.33199700	H	-6.65171600	-4.61943600	0.42716300
C	6.64822400	-3.63860100	0.34774500	H	-5.03026700	-5.25276900	0.77772400
C	6.33435600	-3.40258600	-0.98695500	H	-4.90108700	-3.15356300	2.00996100
C	5.06154500	-2.96121500	-1.33312000	H	-5.94239800	-2.32515000	0.81591000
H	4.81966100	-2.77514500	-2.37666100	H	-2.25539500	-1.62391500	-1.43468000
H	7.08000100	-3.56125800	-1.75936700	H	0.23891900	0.08261700	1.06669600
H	7.63959500	-3.98395000	0.62289600	H	0.52557900	0.80734300	-0.51340100

Table 61. Geometric coordinates and thermally corrected M06-2X energies for **113-*E***.

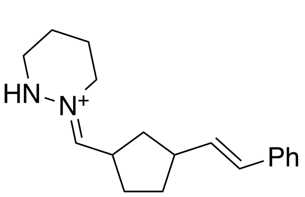

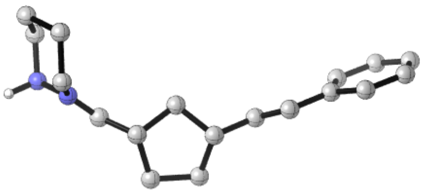

$G_{Acetonitrile}$ = –809.783791716 Hartree

Atom	X	Y	Z
C	0.00000000	0.00000000	0.00000000
C	-1.53217100	0.13040800	-0.15777700
H	-1.99723100	0.49288700	0.76481800
H	-1.81220000	0.83166600	-0.94657500
C	-2.03952100	-1.29699200	-0.52754000
C	-0.84698600	-2.23410000	-0.17999600
H	-0.81593500	-2.43140900	0.90014600
H	-0.90068700	-3.19364600	-0.70193400
C	0.37104600	-1.37862100	-0.57470400
C	1.66687200	-1.93204600	-0.07030400
H	1.76295800	-1.98956000	1.01480300
C	2.66650200	-2.32621500	-0.86519200
C	3.96875200	-2.87404500	-0.45512300
C	4.27875000	-3.18539100	0.87591500
C	5.52746000	-3.69011900	1.21102700
C	6.49072300	-3.89730800	0.22336800
C	6.19412200	-3.59850600	-1.10267800
C	4.94207700	-3.09321300	-1.43665500
H	4.71347900	-2.85818500	-2.47332800
H	6.93719300	-3.75741800	-1.87750500
H	7.46614500	-4.29210000	0.48911600
H	5.75266500	-3.92629800	2.24631500
H	3.53811600	-3.03919400	1.65670700
H	2.53081700	-2.22749300	-1.94346600
H	0.40411400	-1.31962500	-1.67114300
C	-3.21624800	-1.70158100	0.26094100
H	-3.24995300	-1.45078600	1.32140200
N	-4.23656400	-2.38398700	-0.13351200
N	-5.20027900	-2.70446300	0.85949200
C	-5.33968900	-4.17230700	0.98109500
C	-5.64469200	-4.85156400	-0.35072900
C	-4.58973000	-4.46017200	-1.38633100
C	-4.47075000	-2.93752000	-1.48484800
H	-5.40450700	-2.49002900	-1.84344600
H	-3.65891300	-2.63014000	-2.14195000
H	-4.84078600	-4.84769800	-2.37721900
H	-3.61296600	-4.87496700	-1.10978300
H	-6.64074100	-4.54996000	-0.69944500
H	-5.66588500	-5.93676300	-0.21958500
H	-4.40174200	-4.54406600	1.40686300
H	-6.12558700	-4.33484000	1.72176000
H	-6.08222500	-2.29671800	0.54041500
H	-2.22968200	-1.35885500	-1.60123500
H	0.27407800	0.02968000	1.06025000
H	0.53946100	0.80916900	-0.49573200

Table 62. Geometric coordinates and thermally corrected M06-2X energies for **113-*Z***.

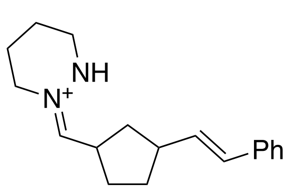

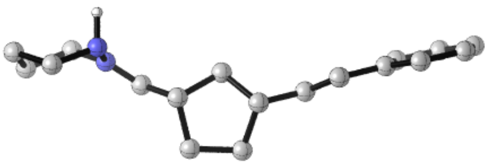

$G_{Acetonitrile}$ = –809.785840123 Hartree

Atom	X	Y	Z
C	0.00000000	0.00000000	0.00000000
C	1.54303400	-0.02516500	0.08965900
H	1.99368300	-0.16952600	-0.89838100
H	1.95564300	0.90315800	0.49010400
C	1.88745300	-1.20106900	1.05902700
C	0.57672200	-2.03245300	1.13189600
H	0.46807800	-2.66572100	0.24020600
H	0.53569900	-2.67294100	2.01613300
C	-0.50605700	-0.93836800	1.10990100
C	-1.88480400	-1.47500800	0.88338700
H	-2.04614300	-1.97431700	-0.07316600
C	-2.88458800	-1.35230000	1.76164300
C	-4.26248000	-1.84503300	1.60748500
C	-4.63292800	-2.75993700	0.61300300
C	-5.95084200	-3.18019000	0.49480700
C	-6.92354400	-2.69899400	1.37101400
C	-6.56672900	-1.79915500	2.37047800
C	-5.24570300	-1.38038100	2.48836800
H	-4.96942900	-0.67627700	3.26946300
H	-7.31564700	-1.42323100	3.06019600
H	-7.95253000	-3.03097300	1.27710200
H	-6.22211800	-3.89070000	-0.27986400
H	-3.88402700	-3.15825900	-0.06536900

Atom	X	Y	Z
H	-2.68802400	-0.81440900	2.69036500
H	-0.47599600	-0.41274200	2.07382400
C	2.97819100	-2.04008400	0.54517200
H	2.91320000	-2.40060000	-0.48259400
N	4.01811300	-2.44227800	1.19433800
N	4.20322200	-2.01583200	2.53110600
C	5.50488600	-1.32419600	2.67076800
C	6.67925500	-2.15526400	2.16273100
C	6.42335100	-2.59074700	0.71892100
C	5.07543600	-3.30148800	0.61118600
H	5.07284500	-4.23194700	1.19014200
H	4.79857100	-3.52771600	-0.42035000
H	7.20107800	-3.27266400	0.36520800
H	6.42347100	-1.71969500	0.05354200
H	6.80979700	-3.03780500	2.80230100
H	7.60227400	-1.57287100	2.22724300
H	5.42110100	-0.38109700	2.12032400
H	5.59706800	-1.08040000	3.73138600
H	4.19351100	-2.86352300	3.10245500
H	2.14162500	-0.83533500	2.05459200
H	-0.32838100	-0.38638700	-0.97109000
H	-0.40506600	1.00886800	0.09970000

Table 63. Geometric coordinates and thermally corrected M06-2X energies for **114-*E***.

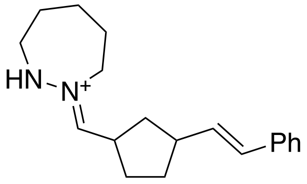

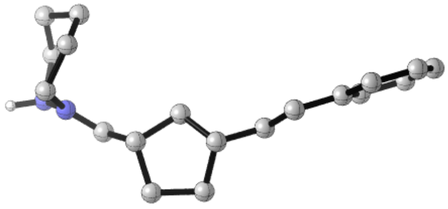

$G_{Acetonitrile}$ = –849.055142649 Hartree

Atom	X	Y	Z
C	0.00000000	0.00000000	0.00000000
C	1.53460100	0.12811900	0.12911400
H	1.97946000	0.52206000	-0.79056500
H	1.83285600	0.80201900	0.93497600

Atom	X	Y	Z
C	2.05303900	-1.31137700	0.44054600
C	0.85403500	-2.23689800	0.09093300
H	0.80009600	-2.40132000	-0.99398500
H	0.91532100	-3.21251300	0.58176900

C	-0.35806900	-1.39701400	0.53626000
C	-1.66100200	-1.93732300	0.03575000
H	-1.78174200	-1.94324000	-1.04839700
C	-2.63884700	-2.38103100	0.83157400
C	-3.94218500	-2.92514400	0.41951900
C	-4.25814700	-3.21243100	-0.91548300
C	-5.50785800	-3.71254400	-1.25397500
C	-6.46570500	-3.93996200	-0.26563000
C	-6.16243000	-3.66683100	1.06448000
C	-4.90939500	-3.16619600	1.40184900
H	-4.67589200	-2.95076600	2.44167000
H	-6.90118800	-3.84194200	1.83996500
H	-7.44177400	-4.33140700	-0.53391800
H	-5.73735000	-3.93079600	-2.29225500
H	-3.52004800	-3.05380300	-1.69628500
H	-2.48353100	-2.32981200	1.91044500
H	-0.37205200	-1.37438200	1.63442000
C	3.21432600	-1.67942100	-0.38602500
H	3.16827100	-1.52744500	-1.46484300
N	4.30567200	-2.23052700	0.02103300
N	5.22471800	-2.63238800	-0.97590000
C	5.14369400	-4.08177800	-1.25876900
C	5.18693800	-5.03662200	-0.06270000
C	3.94157800	-4.95212500	0.83393800
C	3.97450200	-3.84971200	1.90234300
C	4.62339900	-2.54158100	1.43605500
H	5.71232200	-2.60955500	1.47564900
H	4.32609500	-1.68968500	2.05027200
H	2.95631500	-3.65078000	2.25378900
H	4.54528900	-4.18248200	2.77524800
H	3.06074000	-4.81299500	0.19213500
H	3.78860800	-5.90711700	1.34331600
H	6.10553000	-4.88477000	0.52007700
H	5.25701600	-6.04804900	-0.47540100
H	4.21955000	-4.24245200	-1.82528500
H	5.97535800	-4.27000200	-1.94293900
H	6.15199800	-2.38705800	-0.62992000
H	2.27227900	-1.41631500	1.50638600
H	-0.29584600	0.06402300	-1.05287800
H	-0.52912100	0.79252300	0.53255700

Table 64. Geometric coordinates and thermally corrected M06-2X energies for **114-*Z***.

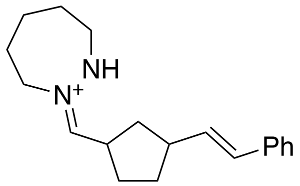

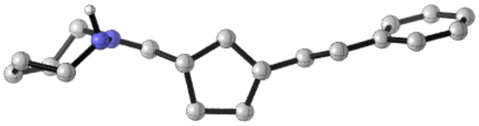

$G_{Acetonitrile}$ = –849.053451607 Hartree

Atom	X	Y	Z
C	0.00000000	0.00000000	0.00000000
C	1.51806100	-0.15861200	-0.21627600
H	2.08909200	0.54592300	0.39886700
H	1.87409700	-1.15914200	0.03955800
C	1.74514200	0.07022700	-1.75886600
C	0.38772800	0.59861700	-2.27909700
H	0.29648900	1.67974800	-2.10261800
H	0.24981700	0.41696500	-3.34744400
C	-0.63284800	-0.13920200	-1.39385800
C	-2.02241400	0.40658800	-1.50098600
H	-2.14559500	1.44984800	-1.20578500
C	-3.07239600	-0.31168800	-1.90992200
C	-4.46488200	0.14574300	-2.03934300
C	-4.82784100	1.49811200	-1.98541200
C	-6.15862600	1.87724300	-2.09753800
C	-7.15229900	0.91366300	-2.27017400
C	-6.80424100	-0.43187100	-2.33613900
C	-5.47050700	-0.80963300	-2.22500600

H	-5.20082200	-1.86168200	-2.27517000
H	-7.57025800	-1.18796100	-2.47514800
H	-8.19147600	1.21382100	-2.35897500
H	-6.42428800	2.92899800	-2.05701100
H	-4.06382100	2.26143100	-1.86934800
H	-2.90704700	-1.35986100	-2.16408300
H	-0.63672600	-1.19860000	-1.68435200
C	2.82607400	1.04654100	-1.95017700
H	2.66486900	2.06459100	-1.59138100
N	3.98626400	0.83551600	-2.47589900
N	4.28268100	-0.46209400	-2.94026100
C	5.40066800	-1.10733900	-2.21659000
C	6.78761700	-0.50826400	-2.46608300
C	7.15486100	0.71084200	-1.60754000
C	6.02670700	1.72729400	-1.36569000
C	5.03041800	1.89432700	-2.51674000
H	5.50869700	1.83236800	-3.49865500
H	4.50848800	2.85143200	-2.45334700
H	5.45268700	1.46687900	-0.46809400
H	6.46468700	2.70982000	-1.16607300
H	7.51388700	0.37180900	-0.63107700
H	8.00123300	1.21665700	-2.08190100
H	6.86275100	-0.26107000	-3.53413300
H	7.53684500	-1.28774900	-2.29725800
H	5.13714500	-1.11660700	-1.15292500
H	5.36991400	-2.14439100	-2.56091600
H	4.51833500	-0.36856200	-3.92969100
H	2.00655000	-0.86775000	-2.24849900
H	-0.22421800	0.99756500	0.39509800
H	-0.39355700	-0.72844500	0.71203100

Table 65. Geometric coordinates and thermally corrected M06-2X energies for **17**.

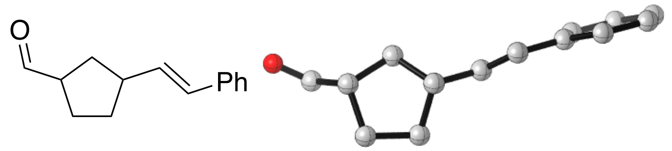

$G_{Acetonitrile}$ = –618.018398821 Hartree

Atom	X	Y	Z
C	0.00000000	0.00000000	0.00000000
C	1.48724400	-0.02542800	-0.39560100
H	2.13616400	0.20947400	0.45561400
H	1.78312700	-1.01562600	-0.75167500
C	1.64403600	1.00890500	-1.55142300
C	0.23577100	1.61751600	-1.73261000
H	0.11814500	2.49427600	-1.07760400
H	0.04493300	1.94515500	-2.75737100
C	-0.72503700	0.51612800	-1.25257200
C	-2.11729300	0.99992600	-0.99795100
H	-2.21028100	1.77898800	-0.23830700
C	-3.20723700	0.53838900	-1.61742900
C	-4.59464900	0.98364200	-1.39997600
C	-4.91139300	2.18061400	-0.74355900
C	-6.23445900	2.55251100	-0.54311300
C	-7.27146600	1.74140200	-1.00178400
C	-6.97171800	0.55724000	-1.66847800
C	-5.64590800	0.18662100	-1.86742000
H	-5.41567400	-0.73968300	-2.38807000
H	-7.77084500	-0.07939900	-2.03599900
H	-8.30474400	2.03582000	-0.84645000
H	-6.45888600	3.48487100	-0.03371800
H	-4.11461400	2.83506500	-0.40236200
H	-3.08517400	-0.26233900	-2.34792300
H	-0.74879200	-0.28219400	-2.00752900
C	2.65090900	2.07448500	-1.20587600
H	2.44398400	2.59397800	-0.24042200
O	3.60499300	2.37761000	-1.87517500
H	1.97728300	0.52577700	-2.47381300
H	-0.16391400	0.71379500	0.81786400
H	-0.37250900	-0.97152800	0.33511800

Table 66. Geometric coordinates and thermally corrected M06-2X energies for **12b**.

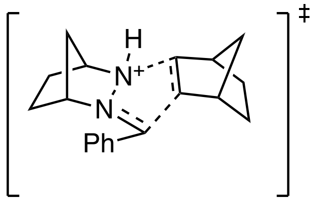

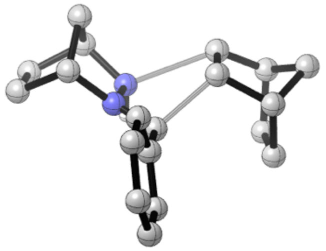

$G_{Acetonitrile}$ = –847.802000053 Hartree

Atom	X	Y	Z	Atom	X	Y	Z
C	0.00000000	0.00000000	0.00000000	H	-0.75717000	2.95408200	-1.10263900
C	0.52625300	1.08888900	0.95663800	H	-0.59264500	2.28504500	2.45178700
C	-0.73084100	1.45149000	1.75919200	H	-1.14992500	0.59243700	2.28616400
C	-1.52231400	1.80563700	0.49299900	H	1.44428000	0.85352000	1.48771600
C	-1.43698200	0.50494300	-0.32751400	H	0.63640000	-0.09474800	-0.88152600
H	-1.59249700	0.68791900	-1.39427400	H	-0.01644100	-0.96407100	0.51430200
H	-2.20307600	-0.20235200	-0.00024800	C	-0.28711600	6.25484000	0.64804400
H	-2.51354300	2.24116100	0.61772900	C	0.33954800	6.25917000	-0.78195400
N	-0.60462300	2.81504900	-0.10233800	C	1.86748300	6.22674400	-0.50484600
N	0.67798300	2.30704700	0.13188200	C	1.92717000	5.98149400	1.02040700
C	1.68048100	3.20224800	0.06474900	C	0.82437000	6.97949100	1.45999600
C	3.09179200	2.80980400	0.31147800	H	0.63967700	6.97209600	2.53711900
C	3.54942000	2.32934600	1.54438600	H	0.99918300	8.00750300	1.12413400
C	4.88417300	1.97888800	1.70516400	H	2.91765200	6.11176100	1.45732200
C	5.77524700	2.11146000	0.64101300	C	1.24162700	4.65279600	1.40724700
C	5.33151100	2.60445600	-0.58259900	H	1.62090900	4.05661700	2.23247500
C	3.99632800	2.96031700	-0.74439800	C	-0.12151400	4.88845200	1.26148800
H	3.65057900	3.34382900	-1.70101200	H	-0.92608300	4.35848700	1.75372900
H	6.02414400	2.71698100	-1.40985800	H	2.42355300	5.50487900	-1.10750700
H	6.81770300	1.84004500	0.77132300	H	2.31629900	7.20110200	-0.71405500
H	5.23293600	1.61136700	2.66439700	H	-0.01375100	5.40931800	-1.37504300
H	2.87280000	2.24947700	2.39156000	H	0.04501700	7.16563900	-1.31531400
H	1.54841100	3.87550100	-0.77613300	H	-1.29578400	6.66375400	0.69829400

Table 67. Geometric coordinates and thermally corrected M06-2X energies for **12c**.

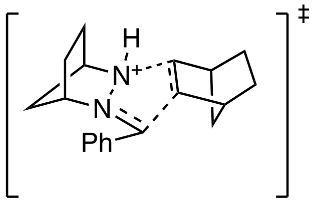

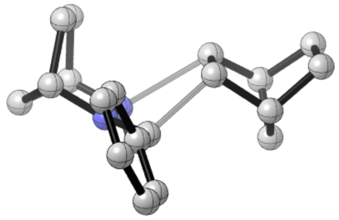

$G_{Acetonitrile}$ = –847.815309678 Hartree

Atom	X	Y	Z	Atom	X	Y	Z
C	0.00000000	0.00000000	0.00000000	H	1.67544000	-3.61039700	-2.46953400
C	0.12595000	-0.07456200	-1.52795800	H	1.87906600	0.47948700	-2.77731300
C	1.53554100	0.49680100	-1.74094800	H	1.65382600	1.49549100	-1.31593600
C	2.17737500	-0.59830900	-0.87809600	H	-0.69831400	0.34640400	-2.10206300
C	1.40622800	-0.48558300	0.45979800	H	-0.82194300	-0.60641100	0.38113200
H	1.36641300	-1.44716200	0.98129100	H	-0.18415500	1.03442700	0.30051400
H	1.89593700	0.23294800	1.12130600	C	-0.42397400	-3.42343600	-0.41791200
H	3.26326700	-0.63895700	-0.82714400	H	-0.74365000	-2.64540600	0.26529000
N	1.65857600	-1.77045300	-1.61407600	C	0.80113300	-4.07226800	-0.37113300
N	0.31067100	-1.51993100	-1.87147200	H	1.45578800	-3.98519700	0.49307300
H	0.08188000	-1.73882500	-2.84211300	C	0.56972200	-5.41502200	-1.06870900
C	2.06700300	-3.04798800	-1.62598700	C	-0.34122100	-6.23337700	-0.11230100
C	3.43990800	-3.42250600	-1.22362900	C	-1.68484100	-5.45310800	-0.15913800
C	4.00293200	-3.02114300	-0.00511900	C	-1.39028900	-4.29121000	-1.17164900
C	5.29152400	-3.41450600	0.33088200	C	-0.45342200	-5.01883000	-2.14947300
C	6.02635900	-4.21819600	-0.54078100	H	-0.06749100	-4.37194800	-2.94401700
C	5.46562200	-4.63857100	-1.74328100	H	-0.92816300	-5.88619600	-2.61597600
C	4.17297500	-4.24954200	-2.08076100	H	-2.27991300	-3.80569800	-1.57286700
H	3.73456900	-4.58002900	-3.01894600	H	-1.99961800	-5.07958900	0.81825100
H	6.03211200	-5.27164600	-2.41788600	H	-2.49596800	-6.07003500	-0.55569400
H	7.03280200	-4.52438300	-0.27504300	H	0.07840300	-6.29827800	0.89413400
H	5.72087200	-3.10225200	1.27705900	H	-0.46386500	-7.25185500	-0.48961400
H	3.42615200	-2.42083500	0.69378500	H	1.47464500	-5.93189900	-1.39753400

Table 68. Geometric coordinates and thermally corrected M06-2X energies for **12d**.

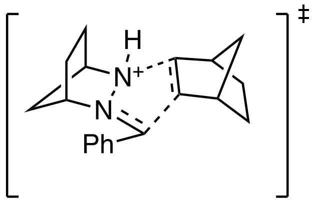

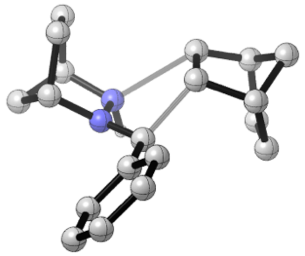

$G_{Acetonitrile}$ = –847.796008104 Hartree

Atom	X	Y	Z
C	0.00000000	0.00000000	0.00000000
C	0.89529600	-0.13076800	-1.25492800
C	0.39469000	1.00856700	-2.15254800
C	-1.05579000	0.49713800	-2.09846100
C	-1.33333600	0.54723800	-0.59038100
H	-2.21729400	-0.02534500	-0.30603600
H	-1.50678500	1.58300100	-0.28767500
H	-1.79638300	0.97307300	-2.73996700
N	-0.90470500	-0.94567300	-2.46979800
N	0.40196800	-1.26102800	-2.06992100
C	0.72955200	-2.56442300	-2.01778500
C	2.16683400	-2.88518700	-1.71947300
C	2.57913400	-3.84499400	-0.79484000
C	3.93511000	-4.11642100	-0.62386800
C	4.88961900	-3.43796700	-1.37308300
C	4.48547500	-2.48372600	-2.30423000
C	3.13463700	-2.21109200	-2.47913100
H	2.82117300	-1.47647600	-3.21664000
H	5.22188200	-1.95238500	-2.89817500
H	5.94420400	-3.65174600	-1.23433400
H	4.24192500	-4.86179000	0.10249000
H	1.85898500	-4.38976700	-0.19628400
H	0.30521600	-3.10572100	-2.85952000

Atom	X	Y	Z
H	-0.99721200	-1.09139700	-3.47644700
H	0.84013800	0.99463300	-3.15015400
H	0.50931400	1.99446600	-1.69810200
H	1.96505500	-0.22854300	-1.08267500
H	-0.11609100	-0.95834800	0.51333700
H	0.45119800	0.69787500	0.70898400
C	-1.71967400	-2.92584600	-0.93158100
H	-2.06291600	-1.99143900	-0.51231100
C	-0.46135700	-3.49769200	-0.74861200
H	0.14530100	-3.20531100	0.10586800
C	-0.65590500	-4.99136600	-1.10735200
C	-0.81098500	-5.18286600	-2.63397100
C	-2.08047300	-4.34580700	-2.94844100
C	-2.62403700	-3.96072300	-1.53419700
C	-2.13762200	-5.17374700	-0.69175000
H	-2.30539000	-5.04582700	0.38063800
H	-2.57278000	-6.12307600	-1.02158700
H	-3.68572300	-3.71837200	-1.51100100
H	-1.87886600	-3.45689100	-3.55358000
H	-2.83079600	-4.93345000	-3.48185800
H	0.06876800	-4.91223600	-3.22256700
H	-0.99125500	-6.24247300	-2.83068200
H	0.06402700	-5.66756300	-0.64533200

Table 69. Geometric coordinates and thermally corrected M06-2X energies for **12e**.

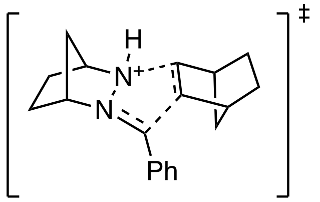

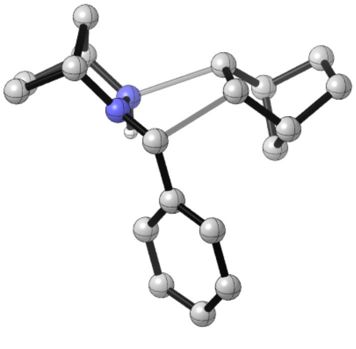

$G_{Acetonitrile}$ = –847.809298982 Hartree

Atom	X	Y	Z
C	0.00000000	0.00000000	0.00000000

Atom	X	Y	Z
C	0.94307900	-1.02247200	-0.66720800

C	0.49770200	-2.33475500	-0.01927600
C	0.79128400	-1.81513200	1.39357800
C	-0.07148700	-0.53532700	1.46305100
H	0.32094100	0.17568200	2.19522000
H	-1.09408900	-0.78247600	1.75749800
H	0.70410500	-2.51385000	2.22479700
N	2.21569200	-1.43171500	1.21480900
N	2.25546100	-0.79497400	-0.01243000
C	3.40910500	-0.47946500	-0.60368500
C	4.40406100	0.42268800	0.00966900
C	5.58976500	0.68840300	-0.68390200
C	6.53377500	1.55954200	-0.15405100
C	6.29310800	2.19301200	1.06353300
C	5.08936600	1.97885900	1.73201200
C	4.14628200	1.10453900	1.20529100
H	3.18516400	1.00407800	1.70409300
H	4.87728900	2.50672300	2.65583800
H	7.02931800	2.87420000	1.47688600
H	7.45252300	1.75437300	-0.69709000
H	5.76711400	0.21282400	-1.64514100
H	3.30809700	-0.38082900	-1.68413800
H	2.62335000	-0.88181800	1.96943300
H	1.08422100	-3.20507100	-0.31431600
H	-0.56432600	-2.52827100	-0.17916000
H	1.04145400	-0.97170200	-1.75105800
H	0.39469400	1.01538800	-0.06915400
H	-0.97710700	-0.02378600	-0.48900400
C	3.79973900	-3.10528300	0.58798400
H	2.90430100	-3.71248500	0.51942500
C	4.42278200	-2.47259500	-0.47896300
H	4.12008400	-2.66631200	-1.50593800
C	5.89579500	-2.39016200	-0.07907700
C	6.41786600	-3.85743000	-0.11457500
C	5.67733400	-4.52252100	1.07730800
C	4.82624400	-3.34586600	1.66077600
C	5.78680800	-2.16779400	1.43846500
H	5.36654400	-1.19895300	1.71685900
H	6.74223700	-2.30685200	1.95206200
H	4.44608400	-3.52080400	2.66811000
H	5.05968200	-5.37355200	0.77949700
H	6.37679600	-4.87214800	1.84149700
H	6.21120400	-4.34509000	-1.07036600
H	7.50041900	-3.86912100	0.03780700
H	6.51220200	-1.68726200	-0.63804500

Table 70. Geometric coordinates and thermally corrected M06-2X energies for **12f**.

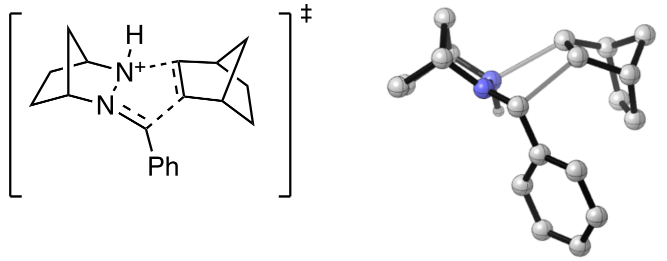

$G_{Acetonitrile}$ = –847.791693810 Hartree

Atom	X	Y	Z
C	0.00000000	0.00000000	0.00000000
C	-0.66247300	1.13383600	-0.81131200
C	0.01821200	2.37335300	-0.22498100
C	-0.47220400	2.05711400	1.19277800
C	0.08722200	0.63505000	1.42280000
H	-0.49601900	0.08852500	2.16961100
H	1.11702500	0.68670400	1.78390400
H	-0.28421400	2.79160700	1.97471400
N	-1.93361100	1.96893500	0.93296900
N	-2.02738000	1.23797200	-0.24242900
C	-3.20225700	1.06920300	-0.86683500
C	-4.31686200	0.30550600	-0.25596500
C	-5.53818700	0.22473900	-0.93210900
C	-6.59160000	-0.49525300	-0.38523100

C	-6.42968700	-1.15275900	0.83481300
C	-5.20012600	-1.11578600	1.48729400
C	-4.14305800	-0.39466800	0.94055800
H	-3.17191400	-0.41401700	1.43052500
H	-5.05963300	-1.65695400	2.41719600
H	-7.25518300	-1.71033500	1.26473100
H	-7.53979900	-0.54770500	-0.90998300
H	-5.66459400	0.74160900	-1.88047500
H	-3.06391400	0.87026700	-1.93036200
H	-2.47539700	1.55159400	1.69012200
H	-0.33381900	3.32266800	-0.62824900
H	1.10496500	2.31773100	-0.30850300
H	-0.70194300	1.01041000	-1.89321300
H	-0.59525300	-0.91475900	-0.02840600
H	0.99011100	-0.22029200	-0.40722000
C	-3.17413100	3.81142100	-0.07949200
H	-2.15598600	4.07815300	-0.32869600
C	-4.02483400	3.04690800	-0.86963400
H	-3.86219400	2.96921400	-1.94234200
C	-5.43655700	3.35699800	-0.33568600
C	-5.65638500	2.83027200	1.10766600
C	-4.64022300	3.64872500	1.94651900
C	-4.00893400	4.60362000	0.89768800
C	-5.23944700	4.86276900	-0.01642700
H	-5.00123700	5.46653100	-0.89555300
H	-6.08411500	5.30935500	0.51982700
H	-3.50360900	5.47049000	1.32403000
H	-3.88828200	3.03102800	2.44399500
H	-5.13577700	4.23733800	2.72263900
H	-5.53842100	1.75562600	1.22263300
H	-6.68081000	3.07118200	1.40464600
H	-6.24491300	3.11124500	-1.02522800

Table 71. Geometric coordinates and thermally corrected M06-2X energies for **12g**.

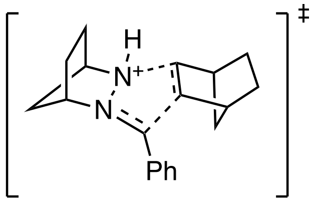

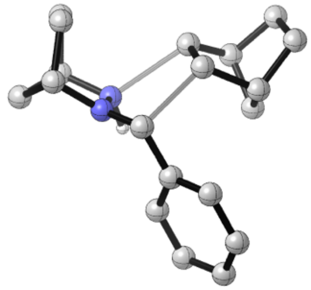

$G_{Acetonitrile}$ = –847.804146990 Hartree

Atom	X	Y	Z
C	0.00000000	0.00000000	0.00000000
C	-0.83844900	-1.28851400	-0.15524600
C	-0.20383300	-1.96825400	-1.37826000
C	-0.41422100	-0.71741300	-2.24908900
C	0.40099400	0.32414800	-1.47006100
H	0.18128400	1.34630600	-1.77919300
H	1.46666100	0.15131100	-1.63971200
H	-0.21545500	-0.79028100	-3.31744400
N	-1.86014400	-0.39731400	-2.00803700
N	-2.14345000	-0.96836600	-0.77296600
C	-3.30290300	-0.74544600	-0.14131900
C	-4.60399000	-1.18368800	-0.69660400
C	-5.75596500	-0.99282800	0.07353200
C	-6.99143900	-1.43548800	-0.38381300
C	-7.08608800	-2.09234900	-1.60873800
C	-5.93600900	-2.33339200	-2.35755100
C	-4.70057300	-1.88899200	-1.90194500
H	-3.80948300	-2.14907700	-2.46673400
H	-5.99834300	-2.88430900	-3.29015100
H	-8.04943100	-2.43922700	-1.96719000
H	-7.87824300	-1.27738100	0.22097700
H	-5.67878000	-0.49988600	1.03971200
H	-3.21118600	-0.89163900	0.93489600
H	-2.49353100	-0.70965500	-2.74124700
H	-0.75586200	-2.84984100	-1.71073900
H	0.85211100	-2.20697600	-1.23694100

H	-0.99446000	-1.88569900	0.74203700
H	-0.57444000	0.79652800	0.48134200
H	0.87188600	-0.20027000	0.62699900
C	-3.46475900	1.41885700	-0.19993100
H	-3.12212000	1.46691000	0.83229300
C	-2.62885800	1.80744200	-1.23948000
H	-1.56701400	1.98939800	-1.13982700
C	-3.45750000	2.46847000	-2.30043300
C	-3.79740500	3.85913200	-1.66329400
C	-4.75650900	3.48476000	-0.50155400
C	-4.84751300	1.93391400	-0.60466500
C	-4.80427300	1.74709900	-2.13011100
H	-4.78692000	0.70206200	-2.44725900
H	-5.62113700	2.26427500	-2.64081800
H	-5.69892100	1.50354300	-0.07987100
H	-4.38934300	3.81161100	0.47432800
H	-5.74605100	3.92507500	-0.64913200
H	-2.90399000	4.39365400	-1.33064700
H	-4.29426500	4.47964100	-2.41409400
H	-3.01974400	2.52487000	-3.29755100

Table 72. Geometric coordinates and thermally corrected M06-2X energies for **12h**.

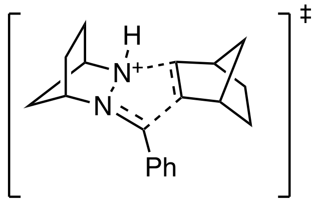

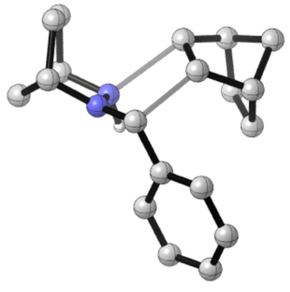

$G_{Acetonitrile}$ = –847.788208809 Hartree

Atom	X	Y	Z
C	0.00000000	0.00000000	0.00000000
C	0.94972300	-1.18538200	0.27165800
C	0.40756900	-1.75300200	1.59389300
C	0.56495700	-0.39670700	2.30247500
C	-0.33300100	0.50109300	1.43852800
H	-0.13540700	1.56143400	1.60260100
H	-1.38119400	0.31945700	1.68832100
H	0.39754400	-0.35146100	3.37781400
N	1.98082000	-0.02601600	1.98296500
N	2.24621400	-0.69992000	0.78854700
C	3.37665400	-0.47163400	0.09443000
C	4.70478500	-0.87172900	0.63088000
C	5.85131300	-0.60151000	-0.12219200
C	7.10137700	-0.98252300	0.34669300
C	7.21548100	-1.65070300	1.56596500
C	6.07196900	-1.96546400	2.29587500
C	4.81842400	-1.58446600	1.82686300
H	3.92913400	-1.88183300	2.37685200
H	6.15361300	-2.51930100	3.22535900
H	8.19244300	-1.94542600	1.93432500
H	7.98766100	-0.76244100	-0.23908000
H	5.76157200	-0.07927700	-1.07200600
H	3.24587300	-0.72889200	-0.95770600
H	2.64633600	-0.28419100	2.71128300
H	1.03066500	-2.55118200	2.00359900
H	-0.63339100	-2.07599200	1.52772800
H	1.11750400	-1.87834200	-0.55175900
H	0.46649500	0.75590300	-0.63818600
H	-0.89183400	-0.35690400	-0.52082600
C	2.58909700	2.13994300	0.79134900
H	1.52927200	2.04622300	0.60503900
C	3.59521500	1.59939600	-0.00078100
H	3.42071300	1.45257300	-1.06559600
C	4.87602700	2.32629100	0.45756700
C	5.27271700	1.95372800	1.91074500
C	4.08874400	2.49085500	2.75607700
C	3.18584900	3.18225600	1.69561700
C	4.26805400	3.72888000	0.72142400

H	3.84637500	4.19696200	-0.17161600
H	4.96501200	4.42098800	1.20614100
H	2.46666400	3.88975000	2.10841200
H	3.54978000	1.71000300	3.29675200
H	4.41613000	3.23187700	3.48960700
H	5.46548600	0.89530400	2.06962500
H	6.19433000	2.48723900	2.15856900
H	5.70141800	2.28439300	-0.25342400

Table 73. Geometric coordinates and thermally corrected M06-2X energies for **13b**.

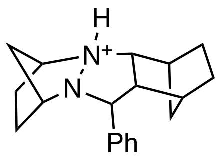

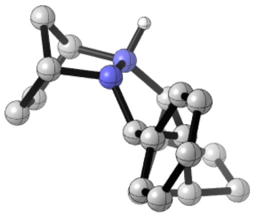

$G_{Acetonitrile}$ = –847.876723734 Hartree

Atom	X	Y	Z
C	0.00000000	0.00000000	0.00000000
C	-0.80412700	-0.18797800	-1.29792800
C	0.23893100	-0.85823100	-2.20502400
C	1.23777300	0.30308900	-2.05870000
C	1.40524200	0.39649700	-0.53960200
H	1.75337800	1.36936700	-0.20029300
H	2.16709200	-0.33182100	-0.24909200
H	2.15114300	0.28811700	-2.65419000
N	0.32862700	1.40732900	-2.57872500
N	-1.00680400	1.08771300	-2.03229900
C	-1.52205500	2.27350800	-1.32656300
C	-3.02799500	2.34893300	-1.43401400
C	-3.67374000	2.11039500	-2.64917300
C	-5.05483000	2.23580000	-2.74186900
C	-5.80059700	2.60313300	-1.62206000
C	-5.16149600	2.83971100	-0.40931100
C	-3.77765200	2.70828500	-0.31516600
H	-3.28047400	2.88489200	0.63623300
H	-5.73810200	3.11964000	0.46617500
H	-6.87888400	2.69985500	-1.69629300
H	-5.55222500	2.04578500	-3.68754200
H	-3.09164800	1.81382600	-3.51759600
H	-1.26414100	2.23708900	-0.26183200
C	-0.78911900	3.47247700	-1.96860100
C	0.55875500	2.91192400	-2.49381100
H	0.77360300	3.20092600	-3.52445200
C	1.61289900	3.52415400	-1.55028500
C	1.77275100	4.96939000	-2.08531700
C	0.41354100	5.64091500	-1.72250300
C	-0.32896000	4.53112600	-0.94925500
C	0.83817700	3.81047000	-0.24884800
H	0.53952800	2.92970300	0.32378000
H	1.40067300	4.47239600	0.41525400
H	-1.14001300	4.88459400	-0.30859600
H	-0.14512600	5.96634300	-2.60455000
H	0.56551200	6.51801100	-1.08964100
H	1.99884900	5.00072400	-3.15480600
H	2.60185500	5.45240000	-1.56315300
H	2.55601900	2.97754900	-1.48410700
H	-1.40127700	3.89563700	-2.76997400
H	0.23817800	1.21838900	-3.58072700
H	-0.11029000	-1.02937800	-3.22694600
H	0.63352300	-1.78727000	-1.78996600
H	-1.76682200	-0.68967100	-1.19988600
H	-0.42414100	0.73939800	0.68119800
H	0.03511800	-0.94582300	0.54635900

Table 74. Geometric coordinates and thermally corrected M06-2X energies for **14b**.

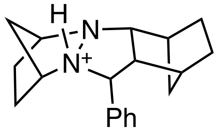

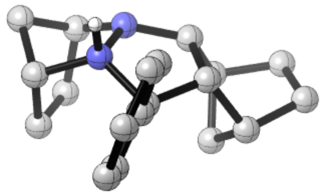

$G_{Acetonitrile}$ = –847.876606048 Hartree

Atom	X	Y	Z
C	0.00000000	0.00000000	0.00000000
C	0.84082700	0.05061400	-1.28154000
C	-0.12966100	0.71265600	-2.27378300
C	-1.21496400	-0.35406700	-2.04620400
C	-1.44587800	-0.23487900	-0.52901600
H	-1.92677700	-1.10983100	-0.09647700
H	-2.08615900	0.62646000	-0.31983400
H	-2.10273400	-0.28534800	-2.67494700
N	-0.43914000	-1.54826700	-2.43373500
C	-0.80045400	-2.96848800	-2.30296000
C	0.45371000	-3.67554100	-1.69721700
C	1.34054600	-2.55516300	-1.12698500
C	2.83437800	-2.70840400	-1.23606600
C	3.43230300	-3.05519400	-2.45265000
C	4.81643000	-3.14537400	-2.55049100
C	5.61178500	-2.89781500	-1.43271900
C	5.02246300	-2.55472000	-0.21941700
C	3.63709400	-2.45369200	-0.12348000
H	3.17702200	-2.18439700	0.82462500
H	5.63904000	-2.36377600	0.65269200
H	6.69130000	-2.97666800	-1.50866800
H	5.27423200	-3.41877000	-3.49544700
H	2.82205200	-3.27394100	-3.32779700
H	1.06862200	-2.36078700	-0.09024400
N	0.90114500	-1.33164100	-1.91612300
H	1.53957700	-1.26969800	-2.72068400
C	-0.15247100	-4.57643500	-0.60345300
C	-1.00342300	-5.65454500	-1.30559900
C	-2.26090900	-4.86388000	-1.77783900
C	-1.94328600	-3.41216400	-1.35843800
C	-1.24001600	-3.66292100	-0.01143300
H	-0.85218000	-2.76791200	0.48345700
H	-1.88429500	-4.18665900	0.70054100
H	-2.81581700	-2.75476600	-1.36076100
H	-2.44542900	-4.95547500	-2.85160500
H	-3.15847500	-5.20968300	-1.25957600
H	-0.46470400	-6.12686100	-2.13210600
H	-1.27509500	-6.44359800	-0.60059800
H	0.59189800	-4.97043000	0.09284500
H	1.00603700	-4.25411500	-2.44235700
H	-1.00637800	-3.32948000	-3.31530800
H	0.23473400	0.75166400	-3.30280300
H	-0.42532900	1.70927600	-1.94083600
H	1.85559600	0.43870100	-1.18962900
H	0.33570900	-0.74418500	0.72474400
H	0.09734700	0.97111600	0.49199400

Table 75. Geometric coordinates and thermally corrected M06-2X energies for **14b TS**.

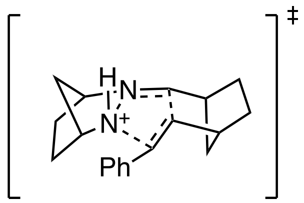

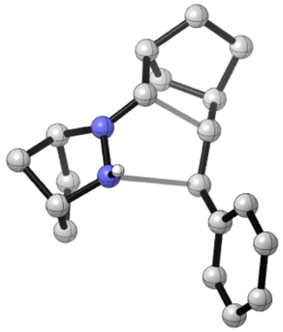

$G_{Acetonitrile}$ = –847.821892989 Hartree

Atom	X	Y	Z	Atom	X	Y	Z
C	0.00000000	0.00000000	0.00000000	H	4.98790100	-2.32060300	0.45046200
C	0.05133900	-0.15020600	-1.52462800	H	5.75732400	-3.63193500	-1.51243300
C	-1.38327800	0.23428800	-1.92540800	H	4.12121400	-4.92127100	-2.85903300
C	-1.98608900	-0.90631100	-1.08355500	H	1.74412400	-4.92454200	-2.25555100
C	-1.40160700	-0.59370500	0.32094700	H	0.37438700	-2.88512200	0.68342700
H	-1.34339100	-1.46863100	0.96927200	H	-0.06759400	-5.15253900	-1.30156300
H	-2.04992800	0.13351200	0.81650000	C	-2.76713900	-3.87323600	0.12341200
H	-3.06107500	-1.07147200	-1.13742000	H	-2.55890800	-2.89289500	0.55042400
N	-1.26043700	-2.01585600	-1.73447200	H	-3.68071400	-4.25930400	0.58563600
C	-1.50532000	-3.34137300	-1.84303200	H	-1.31191400	-5.12506800	1.25490500
C	-2.85512200	-3.87796600	-1.41289600	H	-1.46723000	-6.80409300	-0.83883900
C	-2.93121100	-5.38921300	-1.73545300	H	-2.94213900	-6.59332000	0.10255000
C	-2.21987700	-6.07345900	-0.53221700	H	-2.45865600	-5.62250000	-2.69472200
C	-1.61617200	-4.88263600	0.23432600	H	-3.97458600	-5.70191300	-1.81013200
C	-0.42777300	-4.38029000	-0.62113200	H	-3.67663900	-3.29966700	-1.84569700
C	0.59357000	-3.54643200	-0.15663000	H	-1.03807600	-3.77145100	-2.73308900
C	1.99064500	-3.59624800	-0.55654500	N	0.07494700	-1.62284100	-1.77393500
C	2.44097800	-4.34082900	-1.66089000	H	0.51870700	-1.89536900	-2.65495900
C	3.78544500	-4.34369600	-2.00404800	H	-1.59590900	0.13595000	-2.99164600
C	4.70542900	-3.61788800	-1.24628100	H	-1.66679100	1.22581700	-1.56686200
C	4.27412500	-2.88036400	-0.14470100	H	0.88063900	0.33832100	-2.03376500
C	2.92768500	-2.86288500	0.19064800	H	0.81678100	-0.54351500	0.47946000
H	2.59177500	-2.29023600	1.05200800	H	0.07309000	1.05235900	0.28486500

Table 76. Geometric coordinates and thermally corrected M06-2X energies for **27b**.

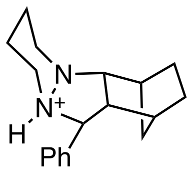

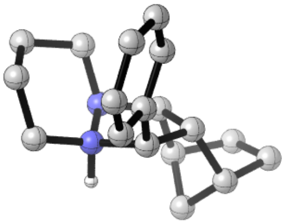

$G_{Acetonitrile}$ = –809.790422263 Hartree

Atom	X	Y	Z	Atom	X	Y	Z
C	0.00000000	0.00000000	0.00000000	H	1.31382900	0.07738400	-1.73831800
C	1.39665100	-0.01916700	-0.65287100	C	1.98334600	-1.40615000	-0.20476100

C	0.79570700	-2.04890400	0.54490300
C	-0.29695100	-2.36075400	-0.49783600
C	-0.87206900	-0.95340500	-0.84355100
H	-0.82351100	-0.72355400	-1.91152200
H	-1.91808200	-0.87032400	-0.53918400
H	0.10842700	-2.87993100	-1.37106000
H	-1.06375700	-3.00584200	-0.06268300
C	0.17209000	-0.84772000	1.27602300
H	0.79449000	-0.41311500	2.06960400
H	-0.78778900	-1.09337700	1.73899900
H	1.08511500	-2.90374500	1.16047100
C	3.19087800	-1.08665600	0.68980800
N	3.02788300	0.44469800	0.91651200
N	2.31940500	1.04341100	-0.20227800
C	3.22487600	1.49556700	-1.27973100
C	4.29163200	2.44100100	-0.75482300
C	5.12131500	1.72657700	0.30741700
C	4.20353200	1.25100500	1.41931100
H	3.75595900	2.10824000	1.92955300
H	4.70843800	0.61969700	2.15287300
H	5.67276400	0.88795900	-0.12873100
H	5.85333900	2.40229600	0.75903300
H	4.92328300	2.76400300	-1.58601300
H	3.81586700	3.33318300	-0.33204100
H	3.69251900	0.64928800	-1.80030100
H	2.56951900	2.00656300	-1.98909600
H	2.33705900	0.50717700	1.66817900
C	4.56430100	-1.47876900	0.20349300
C	4.88572800	-1.59872700	-1.15156900
C	6.18342000	-1.91158900	-1.54365700
C	7.17440400	-2.11801800	-0.58583500
C	6.85737000	-2.03499600	0.76730600
C	5.55761700	-1.72619800	1.15663100
H	5.31105400	-1.69341700	2.21608200
H	7.61612200	-2.22328700	1.51957200
H	8.18557900	-2.36302300	-0.89360700
H	6.41849100	-2.00240000	-2.59903000
H	4.12701800	-1.45594000	-1.91548000
H	3.05029000	-1.50285400	1.69415200
H	2.29562600	-2.02701300	-1.04570200
H	-0.40318300	1.00706000	0.11697500

Table 77. Geometric coordinates and thermally corrected M06-2X energies for **27a TS**.

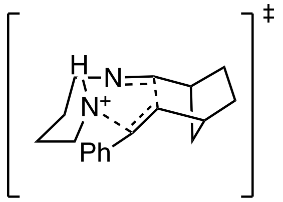

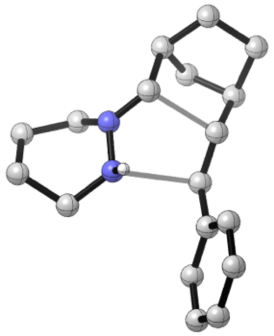

$G_{Acetonitrile}$ = –809.735856652 Hartree

**Atom**	**X**	**Y**	**Z**
C	0.00000000	0.00000000	0.00000000
C	-1.36577300	-0.18444900	0.68685900
H	-1.44417600	0.45177800	1.57273400
H	-2.17100700	0.10403700	0.00764600
C	-1.56983100	-1.64121200	1.14104500
H	-2.62634700	-1.89211000	1.20977700
H	-1.11089000	-1.81605300	2.12355700
N	-0.93945700	-2.54117600	0.17169800
C	-1.39420300	-3.74733100	-0.21519900
C	-2.72940500	-4.25904400	0.26280800
C	-3.01447300	-5.63360400	-0.38595800
C	-2.27294100	-6.65403400	0.52249900
C	-1.48781500	-5.76541800	1.50263300
C	-0.26287300	-5.20721100	0.76248300
C	0.76856800	-4.49541800	1.35754700
C	2.18108900	-4.50971800	0.96315500

C	2.64664700	-5.16142800	-0.18879400	H	-1.18954400	-6.29338000	2.41210200
C	3.99364200	-5.11225400	-0.52891100	H	-1.62548500	-7.33285600	-0.03834800
C	4.89805600	-4.42141300	0.27570000	H	-2.98835400	-7.26837100	1.07513300
C	4.44932300	-3.77591100	1.42683500	H	-2.67762100	-5.66667200	-1.42646300
C	3.10278300	-3.81664100	1.76315500	H	-4.09009100	-5.82066200	-0.39486200
H	2.75344000	-3.31519500	2.66303000	H	-3.53155700	-3.53572400	0.08744400
H	5.15004100	-3.24518700	2.06313400	H	-1.00064700	-4.08056600	-1.17557300
H	5.94998500	-4.39441500	0.01098300	N	0.43556200	-2.42461100	0.16675800
H	4.34129700	-5.62591800	-1.41943000	C	0.96288000	-1.08214300	0.48070900
H	1.96638800	-5.72884900	-0.81728700	H	1.10716000	-1.02248400	1.56537800
H	0.56611500	-4.01013800	2.31513500	H	1.94739300	-1.01532300	0.01522000
H	0.03055200	-5.81198800	-0.09613800	H	0.81551700	-2.79934300	-0.70377100
C	-2.48813900	-4.62999500	1.74353700	H	0.41539100	0.98787200	0.21174900
H	-2.08533200	-3.81680500	2.35277800	H	-0.10472000	-0.08201500	-1.08653400
H	-3.40451300	-4.99306900	2.21963000				

Table 78. Geometric coordinates and thermally corrected M06-2X energies for neutral **8-*E***.

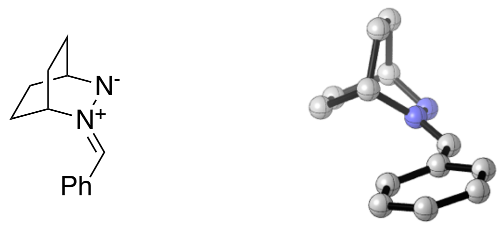

$G_{Acetonitrile}$ = –614.132781802 Hartree

Atom	X	Y	Z	Atom	X	Y	Z
C	0.00000000	0.00000000	0.00000000	H	-5.66957000	-1.89170500	0.46107800
N	-1.21425800	-0.62204000	-0.50965100	C	-2.04242000	1.01041800	1.01709000
N	-2.28044600	-0.08006500	0.03184600	C	-1.27043300	2.12134600	0.30136700
C	-3.49314700	-0.56510300	-0.20416400	C	0.00007100	1.50540700	-0.32673200
H	-3.47793800	-1.49083000	-0.76756500	H	0.90873900	1.98011000	0.05870100
C	-4.77056700	0.03822400	0.14876800	H	-0.00734100	1.62962800	-1.41233700
C	-5.84340800	-0.81899100	0.45414500	H	-1.03148000	2.89608600	1.03680900
C	-7.10158800	-0.31833300	0.75884400	H	-1.90818900	2.58135900	-0.46010500
C	-7.32939600	1.05696400	0.76935000	H	-3.00054600	1.34728200	1.40406000
C	-6.28711500	1.92064400	0.44355100	C	-1.19378000	0.39007700	2.13627500
C	-5.03074900	1.42057200	0.12065300	H	-0.98482200	1.17408200	2.87110800
H	-4.25015300	2.10676700	-0.19685100	H	-1.78627300	-0.38027900	2.63844300
H	-6.45815600	2.99280800	0.41516200	C	0.09701900	-0.19315500	1.52563000
H	-8.31170700	1.44926700	1.01247700	H	0.99132600	0.30254200	1.91896700
H	-7.90844400	-1.00522800	0.99691500	H	0.18316200	-1.25994100	1.74600800

H 0.83177000 -0.49789900 -0.50309800

Table 79. Geometric coordinates and thermally corrected M06-2X energies for neutral **8-Z**.

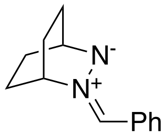

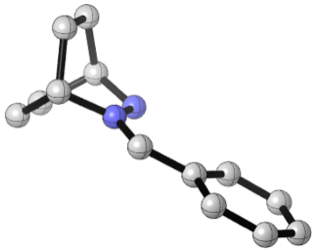

$G_{Acetonitrile}$ = –614.142373201 Hartree

Atom	X	Y	Z
C	0.00000000	0.00000000	0.00000000
N	1.41703400	-0.34636900	-0.00024700
N	1.57184800	-1.65137400	0.00003900
C	2.73714800	-2.28129200	0.00016300
C	4.05301800	-1.68169100	0.00012900
C	5.14775000	-2.57147300	0.00020800
C	6.45273800	-2.10937000	0.00028000
C	6.70885600	-0.73692900	0.00029600
C	5.64055900	0.15285400	0.00021200
C	4.32320800	-0.29974600	0.00009600
H	3.49819600	0.39765400	-0.00003200
H	5.82924800	1.22272200	0.00020200
H	7.73072000	-0.37030300	0.00038000
H	7.27538400	-2.81847300	0.00031700
H	4.95672900	-3.64228800	0.00021700
H	2.66901500	-3.36082100	0.00040200

Atom	X	Y	Z
C	0.32938000	-2.47088300	0.00045400
C	-0.45344400	-2.09583300	-1.26119800
C	-0.68514500	-0.56772800	-1.25662000
H	-1.75272200	-0.32267800	-1.25335600
H	-0.23937700	-0.10806300	-2.14201500
H	-1.39467800	-2.65464300	-1.25799900
H	0.11373100	-2.41243800	-2.14127200
H	0.61850200	-3.52222100	0.00061800
C	-0.45300800	-2.09530000	1.26223000
H	-1.39429300	-2.65402800	1.25952000
H	0.11441900	-2.41166100	2.14222600
C	-0.68457900	-0.56719300	1.25718200
H	-1.75213100	-0.32204100	1.25434100
H	-0.23831700	-0.10721000	2.14216200
H	-0.04697400	1.09122700	-0.00020800

Table 80. Geometric coordinates and thermally corrected M06-2X energies for neutral **24a**.

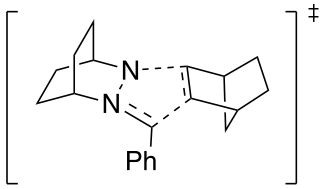

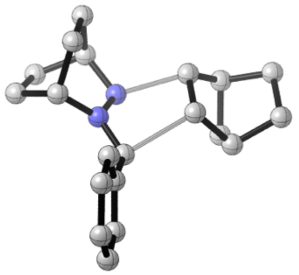

$G_{Acetonitrile}$ = –886.650340396 Hartree

Atom	X	Y	Z	Atom	X	Y	Z
C	0.00000000	0.00000000	0.00000000	C	-6.28799400	1.96396400	-1.38961800
C	-0.97537300	0.88431500	-0.83024900	C	-5.00451700	2.36719400	-1.74070600
C	-1.36602800	2.04532200	0.07454000	H	-4.64649500	2.21706900	-2.75586000
C	-0.25166700	2.84650100	0.17412900	H	-6.92765900	1.49797400	-2.13320800
H	-0.03086100	3.49494700	1.01637400	H	-7.75751600	1.84915900	0.18254200
C	0.84700400	2.19363500	-0.64124000	H	-6.26161700	2.87996300	1.87617000
C	1.25720100	0.91040100	0.14112600	H	-3.96776500	3.54276800	1.26795600
H	1.50438700	1.12998200	1.18389500	H	-2.38203700	2.80870500	-2.07080300
H	2.13519700	0.44321100	-0.31822900	C	-2.77432800	5.61242300	-0.19063700
C	0.02405300	1.57351200	-1.77965800	C	-1.93769300	5.78249100	1.08144800
H	-0.41185500	2.33488500	-2.42784500	H	-2.39824000	6.54189600	1.72100600
H	0.59793800	0.85638900	-2.37798400	H	-1.93761700	4.83788000	1.63749800
H	1.67873100	2.83954500	-0.93185800	C	-0.50748900	6.17562300	0.65581500
H	-2.12625600	1.93801000	0.84330400	C	-0.37706600	5.89539600	-0.85628300
H	-1.80342400	0.33755100	-1.29097500	H	0.66923500	5.92156300	-1.16993800
H	-0.43082600	-0.27991500	0.96545700	C	-1.20619500	6.94723000	-1.61464400
H	0.24176200	-0.92276200	-0.53878900	H	-0.78398500	7.94699300	-1.47021800
N	-0.88437600	4.56727500	-1.21185100	H	-1.17032500	6.71763100	-2.68295600
N	-2.18437300	4.49617600	-0.95429300	C	-2.65380600	6.86043800	-1.08177800
C	-2.78814600	3.32498700	-1.20972400	H	-2.91588400	7.73577200	-0.47776600
C	-4.15652700	2.97910600	-0.80630400	H	-3.37771000	6.79702000	-1.89854300
C	-4.62921200	3.13520800	0.50707400	H	-0.31809700	7.23885100	0.84470200
C	-5.91733800	2.74542600	0.85506700	H	0.24122600	5.61112600	1.21604700
C	-6.75475500	2.16070000	-0.09233000	H	-3.81662300	5.38342000	0.02053800

Table 81. Geometric coordinates and thermally corrected M06-2X energies for neutral **32b**.

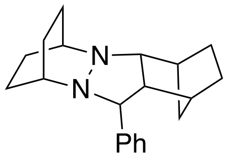

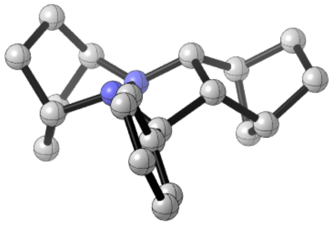

$G_{Acetonitrile}$ = –886.730153350 Hartree

Atom	X	Y	Z	Atom	X	Y	Z
C	0.00000000	0.00000000	0.00000000	C	-4.24523000	-0.08108400	-0.72555900
C	-1.48561000	-0.05912400	-0.24559500	C	-3.74830000	0.42798800	0.47065000
C	-1.99222100	-0.56790500	-1.44558600	C	-2.37599900	0.43280500	0.70912300
C	-3.36291500	-0.58190500	-1.68162000	H	-1.98819600	0.82102400	1.64826200

H	-4.42942600	0.81604400	1.22213600
H	-5.31487300	-0.09102900	-0.91186000
H	-3.74565400	-0.98372300	-2.61528700
H	-1.29354400	-0.95670700	-2.18111500
H	0.19096300	0.09076600	1.08490900
C	0.71744000	1.16544100	-0.74344200
C	2.11553200	0.56838700	-1.13481500
H	2.23355800	0.44119700	-2.22340300
C	3.10249200	1.58037600	-0.53407300
C	2.97844200	2.88302200	-1.34971900
C	1.61230100	3.47110000	-0.87976700
C	1.08757100	2.39902900	0.09521000
C	2.39590000	1.95856400	0.77953600
H	2.28151400	1.10292400	1.44885500
H	2.89049000	2.78038400	1.30857000
H	0.28309500	2.74466400	0.75130600
H	0.91947100	3.64738200	-1.70818100
H	1.75628900	4.42388200	-0.36079300
H	3.00349500	2.69257600	-2.42780400
H	3.80174000	3.56487200	-1.11811800
H	4.12379600	1.19906300	-0.44270500
N	2.09181600	-0.68899800	-0.40902800
C	2.96830400	-1.82445500	-0.71730000
C	2.35492100	-2.72260700	-1.80562400
C	1.07221700	-3.37239000	-1.22429900
C	0.61050200	-2.49166000	-0.05008200
C	1.56846700	-2.70445900	1.13537600
C	3.02294900	-2.61231800	0.60360200
H	3.45004500	-3.60796800	0.43219100
H	3.65964700	-2.09215300	1.32468200
H	1.37518600	-3.67605100	1.60133200
H	1.39283100	-1.93449600	1.89332000
H	-0.43303300	-2.68027000	0.22030200
N	0.71250000	-1.12582100	-0.58249100
H	1.26596100	-4.39332400	-0.87341700
H	0.28932100	-3.42319400	-1.98644000
H	3.07970700	-3.48148900	-2.11786000
H	2.10946000	-2.11774500	-2.68396100
H	3.95077900	-1.43929500	-1.00889900
H	0.12826700	1.43589000	-1.62508200

Table 82. Geometric coordinates and thermally corrected M06-2X energies for neutral **32a**.

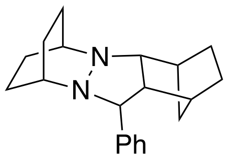

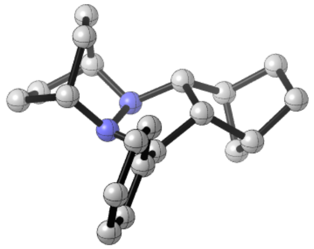

$G_{Acetonitrile}$ = –886.728979617 Hartree

Atom	X	Y	Z
C	0.00000000	0.00000000	0.00000000
C	-1.45509200	-0.18499800	0.37261300
C	-1.89601700	-0.28809800	1.69451300
C	-3.25107400	-0.41784700	1.98582000
C	-4.18988800	-0.44512100	0.95793700
C	-3.76442800	-0.33668100	-0.36296700
C	-2.40830200	-0.20742500	-0.64823400
H	-2.07503400	-0.11922700	-1.67931000
H	-4.48765600	-0.35596200	-1.17277500
H	-5.24629400	-0.55067800	1.18528100
H	-3.57369500	-0.49877100	3.01953600
H	-1.18061700	-0.25928300	2.51149900
H	0.11772300	-0.39693000	-1.01823700
C	1.04573300	-0.72379900	0.87877500
C	2.24503300	0.25800700	0.91752600
H	2.48170700	0.57652100	1.94137700
C	3.40124400	-0.56526600	0.32228600
C	3.77238700	-1.64876700	1.35320500
C	2.57712700	-2.64771300	1.26537500
C	1.64741700	-1.98668200	0.23080200

C	2.67201500	-1.38628300	-0.75143100
H	2.23339500	-0.75353300	-1.52726700
H	3.30671600	-2.14806700	-1.21667200
H	0.89606700	-2.65878800	-0.19414600
H	2.08034200	-2.79924200	2.22880200
H	2.91267200	-3.62682300	0.90961500
H	3.89745300	-1.23188100	2.35820300
H	4.71313800	-2.13691100	1.08201400
H	4.23910300	0.04876200	-0.01836200
N	1.85381800	1.37938800	0.04061300
C	2.30929600	2.70832800	0.48585400
C	1.94929500	2.97966000	1.96358300
C	0.50035400	2.48546600	2.17978100
C	-0.18339200	2.43162200	0.80287000
H	-1.25482300	2.24163000	0.89255600
C	0.08588400	3.75750500	0.07066500
H	-0.57251000	3.84094800	-0.79741100
H	-0.12621200	4.60233100	0.73727400
C	1.57079100	3.74449900	-0.37038600
H	1.66047000	3.45961900	-1.42179100
H	2.03322300	4.72836900	-0.24245300
N	0.39867800	1.41124200	-0.08859400
H	0.49042600	1.48927400	2.63832200
H	-0.05221900	3.15258700	2.84995800
H	2.64561000	2.50139600	2.65774900
H	2.02511400	4.05784700	2.14748600
H	3.39098700	2.75697100	0.32196600
H	0.65930200	-0.93926100	1.87928700

Table 83. Geometric coordinates and thermally corrected M06-2X energies for neutral **107**.

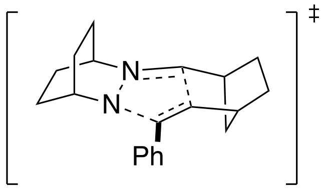

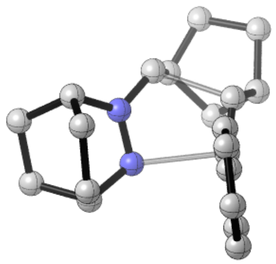

$G_{Acetonitrile}$ = –886.665535749 Hartree

Atom	X	Y	Z
C	0.00000000	0.00000000	0.00000000
C	-1.25762200	0.25391900	0.82293200
H	-1.07439700	0.33512900	1.89396200
N	-2.20225700	1.13605300	0.41785100
C	-2.84255700	2.10568400	1.32559500
C	-4.34880000	1.85201400	1.37391800
C	-4.89664000	1.93649400	-0.06554600
C	-3.69879000	2.05274700	-1.02715900
H	-4.02111200	1.95351900	-2.06628100
C	-3.01321100	3.41574200	-0.79250000
H	-2.14836300	3.49551700	-1.45669700
H	-3.70484800	4.22664900	-1.04178900
C	-2.57514000	3.48365500	0.69135000
H	-1.51365100	3.73102900	0.78057100
H	-3.13986100	4.23970800	1.24638500
N	-2.69156800	1.00932000	-0.79773100
H	-5.48849800	1.05160600	-0.30293800
H	-5.54266300	2.81224200	-0.19666600
H	-4.54170100	0.86859000	1.80668800
H	-4.80596300	2.60043100	2.02972300
H	-2.37189100	2.00872400	2.30578400
C	0.91511300	-0.89349300	0.85539000
C	0.25603000	-2.29549800	0.72598200
C	-0.85700900	-2.09860600	-0.32612300
C	-2.13810200	-1.57695500	0.31382000
C	-3.22175100	-1.30284200	-0.52150200
C	-4.62163200	-1.37857700	-0.11878600
C	-5.03840500	-1.46174300	1.22160000
C	-6.38785800	-1.51549500	1.55489400
C	-7.36535500	-1.50569300	0.56299900

C	-6.97163100	-1.45307500	-0.77461500	C	-0.31866600	-0.93619700	-1.16899600
C	-5.62576100	-1.39063100	-1.10673400	H	-1.04940000	-0.51493900	-1.86062000
H	-5.32738200	-1.34011600	-2.15157900	H	0.59236500	-1.20831700	-1.71588600
H	-7.72001500	-1.45889300	-1.56233700	H	-1.05293700	-3.01077300	-0.89871900
H	-8.41739200	-1.54943200	0.82640800	H	-0.15126800	-2.65503400	1.67645300
H	-6.67766900	-1.56978400	2.60079200	H	0.98255900	-3.03907000	0.38488300
H	-4.29622500	-1.47841700	2.01568400	H	0.99313000	-0.54966700	1.89174500
H	-3.05509100	-1.34492400	-1.59437200	H	1.92595500	-0.89694300	0.43607800
H	-2.34813800	-1.94300200	1.31846100	H	0.45987000	0.95034600	-0.29658100

Table 84. Geometric coordinates and thermally corrected M06-2X energies for **115-*E***.

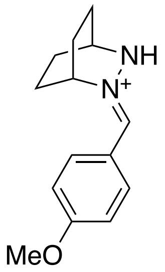

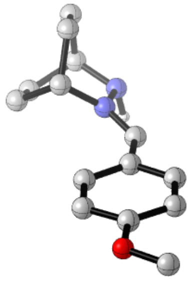

$G_{Acetonitrile}$ = –729.078623740 Hartree

Atom	X	Y	Z	Atom	X	Y	Z
C	0.00000000	0.00000000	0.00000000	H	-9.68516100	-0.07369900	1.62928600
N	-1.20050700	-0.85277500	-0.18598700	H	-7.98762600	-1.30488400	0.58862000
H	-1.27635600	-1.14095200	-1.16073200	H	-5.68345000	-2.07695000	0.31652100
N	-2.37270700	-0.12727900	0.12827300	C	-2.09473300	1.13311700	0.86791400
C	-3.52632300	-0.67642600	-0.09384500	C	-1.37738700	2.08853100	-0.09180500
H	-3.45814000	-1.70848200	-0.43936400	C	-0.18175800	1.33641900	-0.73010900
C	-4.84127800	-0.10630600	0.05672900	H	0.73723000	1.92396400	-0.66259800
C	-5.89590600	-1.01255900	0.26030100	H	-0.36475500	1.14989000	-1.79350100
C	-7.20138800	-0.58086200	0.41558000	H	-1.04452200	2.95282500	0.49012800
C	-7.48405000	0.79107500	0.32725900	H	-2.06568000	2.45964100	-0.85689100
C	-6.44328000	1.70727800	0.06902000	H	-3.04873600	1.51165800	1.22837300
C	-5.14757800	1.26867500	-0.06263100	C	-1.18409500	0.75019800	2.04340300
H	-4.37437800	1.98780000	-0.31464600	H	-1.05300400	1.64259000	2.66131700
H	-6.70130300	2.75473200	-0.03992800	H	-1.68920700	0.00013000	2.65799800
O	-8.69799400	1.32311700	0.44716500	C	0.16347500	0.22123500	1.50272500
C	-9.80711500	0.46228600	0.68278400	H	0.97271400	0.93644100	1.67360900
H	-10.6774090	1.11293000	0.73761700	H	0.43607900	-0.71565900	1.99209400
H	-9.92759100	-0.24787000	-0.14124700	H	0.82924300	-0.57712900	-0.41442500

Table 85. Geometric coordinates and thermally corrected M06-2X energies for **115-*Z***.

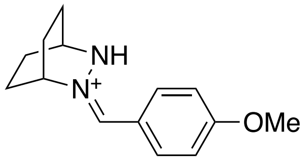

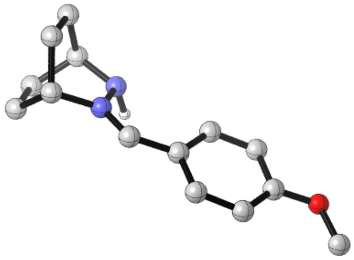

$G_{Acetonitrile}$ = –729.083591855 Hartree

Atom	X	Y	Z	Atom	X	Y	Z
C	0.00000000	0.00000000	0.00000000	H	7.16705500	3.11027200	-0.17095900
N	1.42821700	0.38894100	-0.09807500	H	4.82844400	3.80246800	-0.28301400
H	1.92870400	0.19275100	0.77330400	H	2.62221500	3.42706100	-0.61373800
N	1.54712100	1.76538300	-0.34682000	C	0.24600900	2.46938600	-0.50792000
C	2.70004500	2.35258500	-0.46627500	C	-0.52712900	2.30815400	0.80440900
C	4.02188400	1.80010200	-0.39321600	C	-0.70196800	0.79751600	1.10471700
C	5.06702300	2.74229500	-0.30775200	H	-1.75817800	0.51709300	1.13885500
C	6.39204100	2.35801500	-0.24730700	H	-0.27350600	0.54318200	2.07905500
C	6.70810900	0.98992600	-0.29757900	H	-1.49166500	2.80942500	0.68711200
C	5.67719500	0.03447700	-0.41257600	H	0.00499300	2.81805100	1.61206600
C	4.36023200	0.42380700	-0.45131800	H	0.47767400	3.51392100	-0.72415300
H	3.59062900	-0.32748900	-0.57305400	C	-0.49134100	1.79514600	-1.66941400
H	5.95829100	-1.01117300	-0.47018000	H	-1.46639700	2.27956500	-1.77057600
O	7.94250800	0.49893600	-0.25203400	H	0.05428100	1.96430900	-2.60124700
C	9.04110700	1.39791200	-0.13784600	C	-0.63100000	0.28225700	-1.36388300
H	9.93100600	0.77244100	-0.11790000	H	-1.67960600	-0.02406700	-1.34302900
H	9.08033000	2.06996600	-1.00061000	H	-0.12572500	-0.31530100	-2.12579000
H	8.97273600	1.97531200	0.78936600	H	0.00818200	-1.07050800	0.21504900

Table 86. Geometric coordinates and thermally corrected M06-2X energies for **116**.

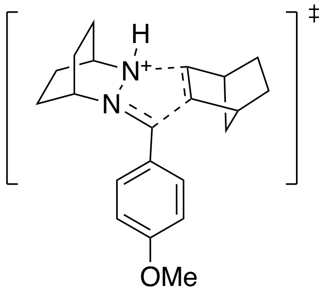

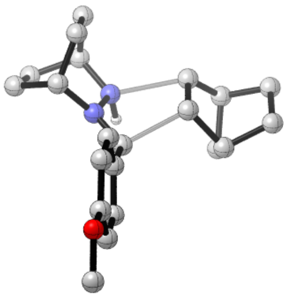

$G_{Acetonitrile}$ = –1001.594512520 Hartree

Atom	X	Y	Z
C	0.00000000	0.00000000	0.00000000
C	0.55254600	-1.15433800	-0.88392600
C	0.36543900	-2.42566700	-0.05891100
C	-0.99656500	-2.65383300	-0.02758500
H	-1.51870100	-3.21886200	0.73739400
C	-1.66732300	-1.57848200	-0.84304700
C	-1.52635300	-0.29682800	0.04508800
H	-1.90259600	-0.45593600	1.05850400
H	-2.10607200	0.51594300	-0.40124700
C	-0.59380200	-1.28570900	-1.90446500
H	-0.47010800	-2.08766900	-2.63967700
H	-0.78339000	-0.35472100	-2.44582200
H	-2.68324300	-1.78131300	-1.18456200
H	1.01558900	-2.66614000	0.77754900
H	1.55919500	-0.99289600	-1.27726200
H	0.46045900	0.00819400	0.99068400
H	0.20005400	0.96677600	-0.46967000
N	-1.00486900	-4.70239500	-1.32017000
N	0.33032000	-4.99958200	-1.09812500
C	1.19115600	-3.98550000	-1.26954300
C	2.61600600	-4.05504900	-0.91516100
C	3.07760900	-4.39259900	0.37106000
C	4.42609600	-4.40196000	0.65673900
C	5.36248700	-4.06152500	-0.33594000
C	4.91823800	-3.69565000	-1.61095400
C	3.55463800	-3.68509800	-1.88127900
H	3.21838000	-3.39173300	-2.87251400
H	5.61807100	-3.41375000	-2.38798200
O	6.64750000	-4.10177400	0.04162900
C	7.64047700	-3.74880300	-0.90908700
H	8.59411600	-3.84957500	-0.39414800
H	7.61438000	-4.42474000	-1.77047600
H	7.51023400	-2.71394500	-1.24355900
H	4.79537000	-4.65225600	1.64517700
H	2.36967000	-4.61992300	1.16507400
H	0.96737900	-3.37754500	-2.14258800
C	0.50639900	-6.20646400	-0.26289600
C	-0.38936400	-6.05906400	0.97362200
H	-0.18990300	-6.88478600	1.66161500
H	-0.13803700	-5.13024000	1.49864700
C	-1.86513900	-6.05222700	0.50543200
C	-1.88324100	-5.85748400	-1.01536800
H	-2.87629000	-5.58523700	-1.37850100
C	-1.33482900	-7.10822200	-1.71103500
H	-2.02430300	-7.94330700	-1.56195100
H	-1.26978400	-6.93097300	-2.78923500
C	0.05947100	-7.40636900	-1.11206900
H	0.03451400	-8.28530700	-0.46088400
H	0.79473900	-7.60131800	-1.89580600
H	-2.35850900	-7.00031100	0.73843200
H	-2.43489200	-5.26312400	1.00131400
H	1.56237600	-6.27716500	-0.01151400
H	-1.13304000	-4.35344000	-2.27061500

Table 87. Geometric coordinates and thermally corrected M06-2X energies for **117a**.

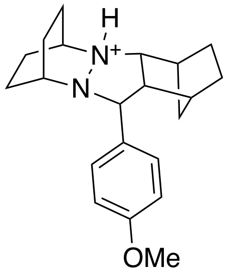

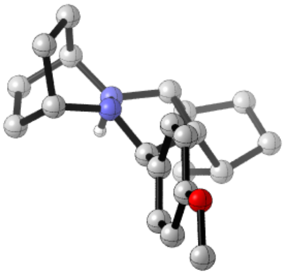

$G_{Acetonitrile}$ = –1001.658909950 Hartree

Atom	X	Y	Z	Atom	X	Y	Z

N	0.00000000	0.00000000	0.00000000
N	-1.37573800	-0.38757700	-0.11281200
C	-2.10117100	0.77156300	0.41016400
C	-3.57708100	0.66258100	0.15026900
C	-4.05241200	0.28605300	-1.11472100
C	-5.40887300	0.21714100	-1.36509000
C	-6.33030300	0.53473500	-0.35399300
C	-5.86865100	0.90822700	0.90980700
C	-4.49597800	0.96248900	1.14894200
H	-4.14557800	1.25099700	2.13730800
H	-6.55899000	1.15200500	1.70814000
O	-7.62860100	0.44729400	-0.70108100
C	-8.60104800	0.78295000	0.27215700
H	-9.56702700	0.66168200	-0.21532000
H	-8.54081400	0.11228200	1.13674300
H	-8.48440400	1.82136500	0.60252800
H	-5.79429600	-0.07489100	-2.33604700
H	-3.34456600	0.04288500	-1.90404900
H	-1.93389100	0.89496300	1.49739800
C	-1.37131100	1.90804000	-0.36545300
C	0.03906100	1.33019200	-0.70955800
H	0.21368900	1.12031700	-1.76668800
C	1.01344500	2.32319400	-0.07185800
C	0.93128900	3.60235700	-0.93674800
C	-0.45774300	4.20464500	-0.56572400
C	-1.02979200	3.17860400	0.43244000
C	0.24585800	2.76715500	1.19151300
H	0.08901300	1.98661300	1.94713700
H	0.73330000	3.60872200	1.69093900
H	-1.86092500	3.54696800	1.03805800
H	-1.10676900	4.33633400	-1.43535500
H	-0.34463100	5.18065000	-0.08788700
H	1.03025300	3.38304400	-2.00383800
H	1.74417200	4.28024700	-0.66740300
H	2.02939100	1.94166200	0.06503400
H	-1.94284500	2.13055100	-1.26961400
C	-1.52803000	-1.79466500	0.30977800
C	-1.12916300	-2.59707600	-0.93849400
H	-0.97179200	-3.64765400	-0.67404300
H	-1.92533600	-2.55404200	-1.68468600
C	0.16640500	-1.95617800	-1.49939000
C	0.86723200	-1.18982100	-0.37749300
H	1.84608600	-0.79729700	-0.66355800
C	0.89360200	-2.03618300	0.90564800
H	1.28805200	-3.02436000	0.65359600
H	1.57289400	-1.60236400	1.64689400
C	-0.55809200	-2.12908900	1.45450900
H	-0.76553300	-3.13196600	1.83446900
H	-0.70925500	-1.44325200	2.29750000
H	0.86112200	-2.71260700	-1.87357200
H	-0.05699700	-1.28514200	-2.33366800
H	-2.57407700	-1.93672200	0.59003100
H	0.22561800	0.20658200	0.98921500

Table 88. Geometric coordinates and thermally corrected M06-2X energies for **117b**.

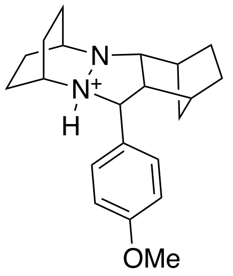

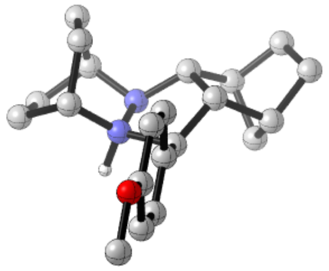

$G_{Acetonitrile}$ = –1001.659362760 Hartree

Atom	X	Y	Z
C	0.00000000	0.00000000	0.00000000
C	-1.45768600	0.01594600	0.35752300
C	-1.91929900	0.02703500	1.68331000
C	-3.27103100	0.08280200	1.96241800

C	-4.21006600	0.12411500	0.91898500
C	-3.76771100	0.10676400	-0.40733500
C	-2.40179000	0.05263400	-0.66896200
H	-2.07122800	0.02449300	-1.70546400
H	-4.46940500	0.12228100	-1.23230700
O	-5.49714100	0.17183600	1.29486000
C	-6.49246600	0.18607100	0.28418000
H	-7.44664400	0.21931400	0.80707700
H	-6.39229700	1.07206900	-0.35253900
H	-6.44076100	-0.72001300	-0.32904200
H	-3.63893300	0.08368500	2.98264400
H	-1.22268600	-0.01854400	2.51538900
H	0.10085400	-0.37435500	-1.02381100
C	0.98541400	-0.76223300	0.88630200
C	2.28580500	0.08510000	0.91883800
H	2.55794100	0.38091500	1.93728200
C	3.34324100	-0.84753900	0.30534200
C	3.61472900	-1.95317100	1.34566000
C	2.32098800	-2.82063500	1.29111200
C	1.45408700	-2.09009700	0.24919500
C	2.52063300	-1.60122400	-0.74947900
H	2.14246900	-0.94746400	-1.54269100
H	3.07028000	-2.42498400	-1.21295200
H	0.63264400	-2.68769900	-0.15456100
H	1.82072200	-2.90274300	2.26040300
H	2.54272400	-3.83535400	0.95141400
H	3.80836400	-1.54463100	2.34239000
H	4.49266300	-2.53574600	1.05754500
H	4.23279700	-0.32016300	-0.04633500
N	2.04225100	1.27612000	0.07384400
C	2.58456200	2.55786400	0.59555500
C	2.12373100	2.83030400	2.03962200
C	0.62089500	2.48610400	2.11340000
C	0.03229700	2.57016900	0.70774000
H	-1.05311600	2.46112300	0.69340600
C	0.52314600	3.83401200	-0.00979500
H	-0.04479800	4.00517400	-0.93011400
H	0.32756500	4.68935400	0.64319900
C	2.03991900	3.67637100	-0.29994700
H	2.22326600	3.42497200	-1.34963900
H	2.57385200	4.60670300	-0.09482000
N	0.59956700	1.42345100	-0.11497400
H	0.47560900	1.69556400	-1.09542300
H	0.46469300	1.48383800	2.52568200
H	0.07643200	3.17924100	2.75970600
H	2.69521300	2.26661700	2.77952600
H	2.29330800	3.88852400	2.26191100
H	3.67084500	2.47563500	0.51187200
H	0.57301800	-0.92096700	1.88532700

Table 89. Geometric coordinates and thermally corrected M06-2X energies for **118**.

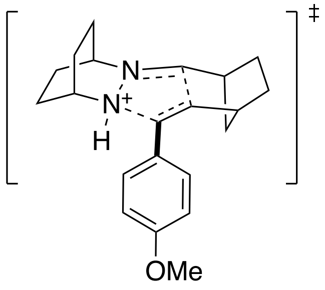

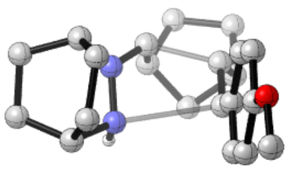

$G_{Acetonitrile}$ = –1001.606446020 Hartree

Atom	X	Y	Z
C	0.00000000	0.00000000	0.00000000
C	1.27188000	0.40895900	-0.77266600
H	1.14903800	0.34080000	-1.85477300
N	1.90688200	1.56109600	-0.45948800
C	2.50210800	2.51820300	-1.40502600
C	4.02227800	2.56969900	-1.23178100
C	4.33971300	2.98722400	0.22554500
C	3.02945500	3.02064400	1.01546100

H	3.20986200	3.13949000	2.08625200
C	2.10799200	4.12196800	0.47035300
H	1.15026700	4.09153100	1.00065300
H	2.55298700	5.10073900	0.66910500
C	1.90532600	3.89105300	-1.04904800
H	0.84561400	3.91962900	-1.31324000
H	2.41442500	4.65756800	-1.64074700
N	2.37629700	1.69645100	0.85141900
H	1.57535400	1.62506600	1.48455900
H	5.03714700	2.28381100	0.68508400
H	4.79675000	3.98038700	0.26665200
H	4.45667700	1.59544100	-1.46213200
H	4.42651500	3.28812800	-1.95063200
H	2.21474100	2.19784600	-2.40874000
C	-0.76263000	-1.00231100	-0.88531800
C	0.06943000	-2.30733900	-0.73251700
C	1.12846000	-1.94556400	0.32709700
C	2.28488100	-1.17253700	-0.32150200
C	3.35139300	-0.82927400	0.52711800
C	4.71489000	-0.60555800	0.15016600
C	5.18867900	-0.75103700	-1.17617800
C	6.51885200	-0.57564300	-1.47794600
C	7.43992200	-0.25502400	-0.46030200
C	6.99845800	-0.11683300	0.86625200
C	5.65677400	-0.29584900	1.15144100
H	5.31398700	-0.18952800	2.17802000
H	7.69290900	0.11668600	1.66392100
O	8.70614100	-0.11056200	-0.85383900
C	9.69838000	0.18398600	0.12086500
H	10.6387670	0.24083000	-0.42382400
H	9.49402200	1.14407800	0.60588400
H	9.75392700	-0.61130800	0.87104800
H	6.89661000	-0.69285400	-2.48765600
H	4.50263900	-1.01728100	-1.97476100
H	3.16281900	-0.83926700	1.59921700
H	2.56897400	-1.49780700	-1.32280800
C	0.40611700	-0.89390300	1.17648900
H	1.04865700	-0.41527700	1.92141200
H	-0.46939800	-1.30190200	1.69021000
H	1.49226100	-2.81395500	0.88171900
H	0.53021800	-2.62664600	-1.67209100
H	-0.55385600	-3.13388500	-0.38360400
H	-0.85144300	-0.66523400	-1.92179100
H	-1.77642100	-1.13406000	-0.49914800
H	-0.57565600	0.89690700	0.25122100

Table 90. Geometric coordinates and thermally corrected M06-2X energies for **119-*E***.

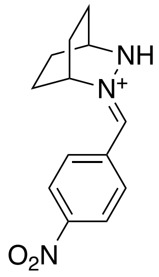

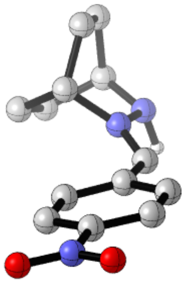

$G_{Acetonitrile}$ = –819.085582079 Hartree

Atom	X	Y	Z
C	0.00000000	0.00000000	0.00000000
N	-1.12685300	-0.92867200	-0.26397800
H	-1.14968200	-1.24637600	-1.23115300
N	-2.35617000	-0.34372300	0.04802200
C	-3.45579000	-0.93924700	-0.27300100
H	-3.32421100	-1.91245200	-0.74583200
C	-4.81950100	-0.45822500	-0.05567800
C	-5.78771000	-1.42460000	0.25388300
C	-7.10878900	-1.04940500	0.45504200
C	-7.43865000	0.28959400	0.30635700
C	-6.51373900	1.26472800	-0.04042500
C	-5.19222900	0.88422300	-0.22246500

H	-4.46380400	1.62488200	-0.53768800
H	-6.83987900	2.28959800	-0.17211000
N	-8.84885800	0.69669700	0.50643900
O	-9.63365900	-0.17958300	0.80061600
O	-9.10317400	1.87413300	0.36161900
H	-7.87783700	-1.76868500	0.71112100
H	-5.50477900	-2.46863200	0.35100300
C	-2.20444800	0.88089100	0.89192500
C	-1.55026300	1.95834500	0.01832500
C	-0.28370000	1.36277600	-0.64395400
H	0.57854400	2.02087300	-0.51165900
H	-0.42905400	1.23789100	-1.72184300
H	-1.30429100	2.79901100	0.67340900
H	-2.25233800	2.33129100	-0.73282100
H	-3.19846300	1.15193900	1.24265800
C	-1.28848800	0.48838300	2.05939500
H	-1.25790800	1.33883700	2.74545700
H	-1.73372600	-0.35010300	2.60163500
C	0.11480700	0.13238500	1.51908900
H	0.84536800	0.91054800	1.75552100
H	0.47053900	-0.80379200	1.95289500
H	0.87827300	-0.48190400	-0.43341200

Table 91. Geometric coordinates and thermally corrected M06-2X energies for **119-*Z***.

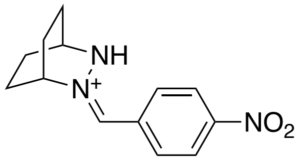

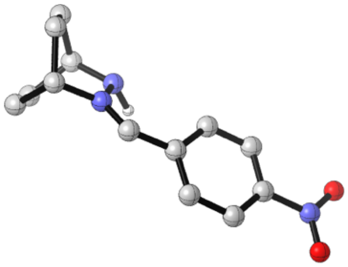

$G_{Acetonitrile}$ = –819.089456716 Hartree

Atom	X	Y	Z
C	0.00000000	0.00000000	0.00000000
N	1.35865100	0.39142500	-0.45717500
H	2.07743200	0.26307400	0.25774400
N	1.38480400	1.71114700	-0.88424400
C	2.46840300	2.38378800	-1.09395800
C	3.84331300	1.93032200	-0.92640300
C	4.80122700	2.93093400	-0.68797900
C	6.13885200	2.60033900	-0.53436200
C	6.49871500	1.26567100	-0.65580800
C	5.58708200	0.25630600	-0.92984400
C	4.24569900	0.58914800	-1.05871800
H	3.52883200	-0.18497200	-1.30822000
H	5.93589800	-0.76359500	-1.03962100
N	7.92656500	0.90255700	-0.49894100
O	8.20564800	-0.27338600	-0.60305100
O	8.69953900	1.81018900	-0.27689900
H	6.89786800	3.34791100	-0.33680400
H	4.49452300	3.97039700	-0.61573100
H	2.30264000	3.41212100	-1.40384900
C	0.01854800	2.27192200	-1.11607500
C	-0.65110600	2.39405400	0.25878500
C	-0.53451200	1.03623900	0.99444300
H	-1.50205500	0.70916000	1.38262700
H	0.14547200	1.11269400	1.84878300
H	-1.69532700	2.66997300	0.08607600
H	-0.19238500	3.20255500	0.83352100
H	0.15784500	3.23760300	-1.60416700
C	-0.72719900	1.27587900	-2.00918400
H	-1.69573100	1.71603700	-2.25987100
H	-0.17894100	1.14160500	-2.94554600
C	-0.88338500	-0.06265100	-1.24778300
H	-1.91915400	-0.22952500	-0.94034200
H	-0.58400200	-0.90224100	-1.87767500
H	0.12315100	-0.98340500	0.45693600

Table 92. Geometric coordinates and thermally corrected M06-2X energies for **120**.

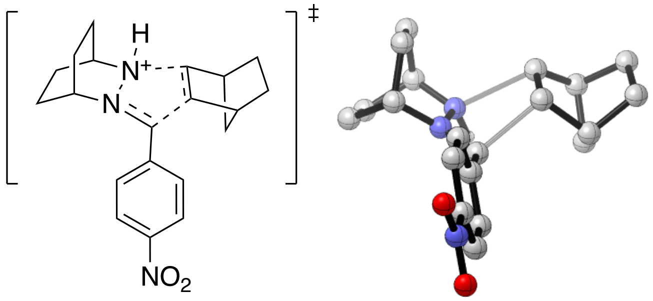

$G_{Acetonitrile}$ = –1091.604481090 Hartree

Atom	X	Y	Z
C	0.00000000	0.00000000	0.00000000
C	0.56012500	-1.13537200	-0.90492300
C	0.47605700	-2.40424900	-0.05973600
C	-0.86781500	-2.69824200	0.04387600
H	-1.32100400	-3.29826300	0.82629300
C	-1.63173400	-1.66378800	-0.74260300
C	-1.50522400	-0.36751000	0.12789300
H	-1.81955100	-0.53434500	1.16090100
H	-2.14639800	0.41143500	-0.29351700
C	-0.63070000	-1.33180400	-1.86125100
H	-0.50882800	-2.13519600	-2.59574300
H	-0.89173300	-0.41740300	-2.40081200
H	-2.65318700	-1.91822600	-1.02740300
H	1.18520800	-2.61544600	0.73608900
H	1.53527000	-0.92696800	-1.35175800
H	0.51116600	0.03943400	0.96454200
H	0.13058300	0.96979000	-0.48696100
N	-0.84387300	-4.78967200	-1.27597000
N	0.50943400	-5.00904500	-1.09952100
C	1.30688100	-3.96500200	-1.32263200
C	2.75939800	-3.97546200	-1.04031800
C	3.28129900	-4.25521400	0.23071700
C	4.65095900	-4.22384800	0.44916800
C	5.48061600	-3.89785800	-0.61548300
C	4.99924000	-3.58953000	-1.87767300
C	3.62455900	-3.62247100	-2.08184000

Atom	X	Y	Z
H	3.22358100	-3.38170800	-3.06220900
H	5.69187500	-3.32889800	-2.66914700
N	6.94099400	-3.86358400	-0.38540800
O	7.63893200	-3.54932200	-1.32670600
O	7.32838800	-4.15523600	0.72747500
H	5.08415700	-4.43181400	1.42031900
H	2.61600800	-4.47375600	1.06199400
H	1.00977900	-3.35486900	-2.17097400
C	0.78262600	-6.21248100	-0.28048100
C	-0.07625000	-6.11717600	0.98683100
H	0.19178000	-6.93657600	1.65863700
H	0.14698400	-5.18042100	1.51032500
C	-1.56660600	-6.18364700	0.57219900
C	-1.64891100	-5.99071100	-0.94665100
H	-2.66655700	-5.76979000	-1.27430300
C	-1.05934900	-7.20888200	-1.66618600
H	-1.69398500	-8.08112100	-1.48972900
H	-1.04843700	-7.03004100	-2.74587800
C	0.37216400	-7.42909000	-1.12431400
H	0.42382600	-8.31113700	-0.47904900
H	1.08718200	-7.57759100	-1.93646400
H	-2.00399300	-7.15419900	0.82285700
H	-2.15525700	-5.42173100	1.08802500
H	1.84869300	-6.22709200	-0.06477400
H	-1.02779700	-4.42945300	-2.21258600

Table 93. Geometric coordinates and thermally corrected M06-2X energies for **121a**.

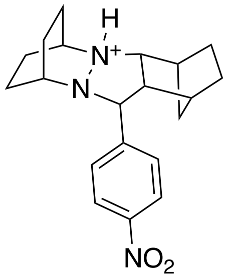

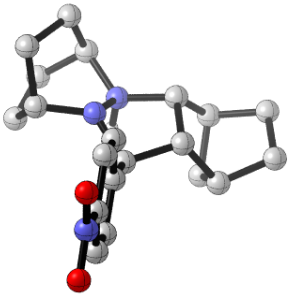

$G_{Acetonitrile}$ = –1091.671825200 Hartree

Atom	X	Y	Z
N	0.00000000	0.00000000	0.00000000
N	-1.38722000	-0.36051200	-0.00656300
C	-2.05210500	0.81936100	0.54180400
C	-3.54510400	0.74350400	0.34732500
C	-4.07024700	0.31985200	-0.87853600
C	-5.44337100	0.27552200	-1.07561000
C	-6.27129500	0.66617800	-0.03119200
C	-5.78327800	1.09198600	1.19371000
C	-4.40412800	1.12334100	1.37790400
H	-3.99938100	1.44833500	2.33252200
H	-6.47459100	1.38367700	1.97531700
N	-7.73327200	0.62057900	-0.23507300
O	-8.43273800	0.96890800	0.69420600
O	-8.12777500	0.23681800	-1.31794300
H	-5.88099700	-0.05043500	-2.01167600
H	-3.39715800	0.02202100	-1.67790800
H	-1.83172200	0.95164100	1.61751600
C	-1.35102900	1.93731400	-0.28668800
C	0.01754200	1.32135200	-0.72461100
H	0.10945400	1.09921700	-1.78966400
C	1.05800200	2.29549700	-0.16600300
C	0.94442100	3.57213600	-1.03058300
C	-0.39925000	4.20926300	-0.56374800
C	-0.92265500	3.20183300	0.47949700
C	0.39349000	2.76153700	1.14684000
H	0.27370200	1.98816500	1.91700500
H	0.93398600	3.59323900	1.60643800
H	-1.69764900	3.59525900	1.14121000
H	-1.10616100	4.35503400	-1.38451600
H	-0.22924400	5.18331700	-0.09924500
H	0.96133300	3.34630900	-2.10061700
H	1.79029500	4.23123500	-0.82403800
H	2.07165100	1.88994100	-0.09967200
H	-1.97530600	2.17270600	-1.15201600
C	-1.53357200	-1.75862400	0.45415600
C	-1.24982300	-2.59017100	-0.80512500
H	-1.09066500	-3.63826400	-0.53273300
H	-2.10077700	-2.54860100	-1.48828500
C	0.00845400	-1.98327900	-1.47788600
C	0.81134400	-1.21562000	-0.42751900
H	1.77115100	-0.84671200	-0.79630700
C	0.92200200	-2.04171100	0.86451900
H	1.27440600	-3.04227000	0.59997200
H	1.66554200	-1.61146100	1.54344500
C	-0.48422900	-2.09359600	1.52628200
H	-0.68268100	-3.08534700	1.93865000
H	-0.55537800	-1.39214900	2.36720900
H	0.65694900	-2.75900500	-1.89307600
H	-0.26798400	-1.32089900	-2.30308100
H	-2.55628200	-1.87647400	0.81916200
H	0.30360100	0.20913100	0.96754000

Table 94. Geometric coordinates and thermally corrected M06-2X energies for **121b**.

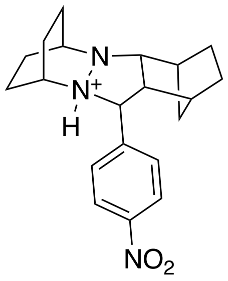

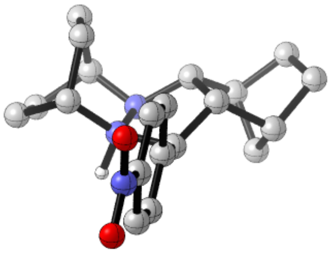

$G_{Acetonitrile} = –1091.669765810$ Hartree

Atom	X	Y	Z
C	0.00000000	0.00000000	0.00000000
C	-1.48262800	0.02560200	0.28101100
C	-2.00596000	0.01651200	1.57861000
C	-3.37920300	0.07640200	1.78500000
C	-4.21169100	0.14334700	0.67738300
C	-3.73146000	0.14455800	-0.62246900
C	-2.35550400	0.08439200	-0.81054300
H	-1.96011500	0.06777400	-1.82323000
H	-4.42608900	0.18433100	-1.45307600
N	-5.67325800	0.21222900	0.89340300
O	-6.05888800	0.21742100	2.04424800
O	-6.37290200	0.26223300	-0.09678900
H	-3.81119600	0.06757500	2.77885000
H	-1.35276000	-0.04313900	2.44323900
H	0.14419600	-0.37291400	-1.01872700
C	0.93197500	-0.78203200	0.92539800
C	2.23699400	0.05473800	1.01859600
H	2.46710000	0.34239000	2.04943700
C	3.31148400	-0.88238000	0.44296300
C	3.53081500	-1.99833700	1.48444000
C	2.23274500	-2.85284100	1.37178700
C	1.41656000	-2.10943900	0.29783900
C	2.52851700	-1.62168700	-0.65152500
H	2.19122800	-0.96083700	-1.45733400
H	3.09076800	-2.44735700	-1.09580900
H	0.60932000	-2.69896500	-0.14469800
H	1.69183100	-2.93495600	2.31899900
H	2.45790800	-3.86801600	1.03632400
H	3.69016400	-1.60016400	2.49120300
H	4.41361200	-2.58666800	1.22475300
H	4.21867900	-0.35920200	0.13297900
N	2.03778200	1.25172100	0.17007600
C	2.56981900	2.52749900	0.71875300
C	2.05890300	2.79374300	2.14663500
C	0.55194500	2.46012800	2.16442100
C	0.01429500	2.56190500	0.73938800
H	-1.07128700	2.46831000	0.68334900
C	0.54255900	3.82543600	0.04797700
H	0.00823300	4.00894300	-0.88985900
H	0.33199700	4.67769400	0.70022200
C	2.06690000	3.65472600	-0.19002100
H	2.28477100	3.40741200	-1.23405100
H	2.60145500	4.57910900	0.03834400
N	0.60246200	1.41459400	-0.07438700
H	0.52399700	1.70107400	-1.05575800
H	0.37385500	1.45531200	2.56273800
H	-0.00968000	3.15211600	2.79706400
H	2.59910700	2.22205300	2.90354400
H	2.22704100	3.84911800	2.38243800
H	3.65758100	2.43641700	0.67331200
H	0.47667800	-0.94346900	1.90516900

Table 95. Geometric coordinates and thermally corrected M06-2X energies for **122**.

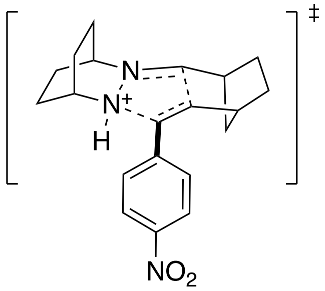

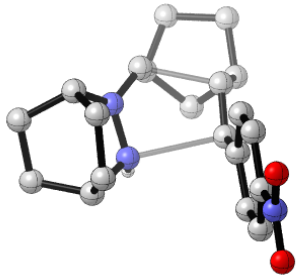

$G_{Acetonitrile}$ = –1091.616170220 Hartree

Atom	X	Y	Z
C	0.00000000	0.00000000	0.00000000
C	1.23984900	0.45833700	-0.78556100
H	1.07346600	0.46975300	-1.86378600
N	1.99357800	1.51309300	-0.42597900
C	2.52399400	2.54760300	-1.33948100
C	4.04842000	2.46776300	-1.39554600
C	4.60508500	2.69660100	0.02895900
C	3.43320900	2.70633100	1.01688800
H	3.77365500	2.66117600	2.05338100
C	2.54092800	3.92949300	0.75228600
H	1.67561000	3.90970300	1.42241000
H	3.10345100	4.83821300	0.98115400
C	2.09583200	3.89674900	-0.73199900
H	1.01456100	4.02278200	-0.82255400
H	2.56973600	4.69596600	-1.30878500
N	2.62124700	1.47769500	0.79491400
H	1.93895200	1.32933700	1.54070600
H	5.33072500	1.92615000	0.29324300
H	5.11407800	3.66154100	0.10631600
H	4.35380500	1.49388300	-1.78413800
H	4.40952100	3.22649300	-2.09507700
H	2.06257500	2.37499200	-2.31308800
C	-0.77103200	-0.95736300	-0.92811100
C	0.07317700	-2.26151600	-0.86306600
C	1.12528700	-1.97209100	0.22717300
C	2.31684600	-1.20460700	-0.35333800

Atom	X	Y	Z
C	3.32096900	-0.84037100	0.54610900
C	4.74098600	-0.65830400	0.25656300
C	5.27052300	-0.69920700	-1.04261200
C	6.62888600	-0.50933100	-1.26011000
C	7.45207100	-0.29936500	-0.16257900
C	6.97577200	-0.29174200	1.14295400
C	5.61625000	-0.47070900	1.34159700
H	5.21740300	-0.46162400	2.35259600
H	7.66669300	-0.14585400	1.96471500
N	8.89511600	-0.08754100	-0.39163400
O	9.58725500	0.10008300	0.58839800
O	9.28032900	-0.10872500	-1.54329400
H	7.06036600	-0.53242000	-2.25395900
H	4.62447000	-0.89037800	-1.89403800
H	3.10157800	-0.95940300	1.60612200
H	2.62232400	-1.46025900	-1.36782100
C	0.40713700	-0.95595800	1.12396900
H	1.03938900	-0.52217300	1.90505500
H	-0.47313000	-1.38352000	1.61286700
H	1.46092800	-2.87451200	0.74410200
H	0.54307300	-2.50687900	-1.81998200
H	-0.54446100	-3.11664400	-0.58066100
H	-0.88518600	-0.56406200	-1.94176800
H	-1.77514800	-1.11755700	-0.52818200
H	-0.57540500	0.88047400	0.30503100

References

1) Potier, J.; Commarieu, B.; Soldera, A.; Claverie, J. P. Thermodynamic Control in the Catalytic Insertion Polymerization of Norbornenes as Rationale for the Lack of Reactivity of Endo-Substituted Norbornenes. *ACS Catal.* **2018**, *8*, 6047–6054.
2) Kang, H. A.; Bronstein, H. E.; Swager, T. M. Conductive block copolymers integrated into polynorbornene-derived scaffolds. *Macromolecules* **2008**, *41*, 5540–5547.
3) Liu, W.; RajanBabu, T. V. Reactivity and Selectivity in Hydrovinylation of Strained Alkenes. *J. Org. Chem*. **2010**, *75*, 7636–7643.
4) Wang, J.; Li, R.; Dong, Z.; Liu, P.; Dong, G. Complementary site-selectivity in arene functionalization enabled by overcoming the ortho constraint in palladium/norbornene catalysis. *Nature Chem*. **2018**, *10*, 866–872.
5) *CrysAlisPro*; Rigaku OD, The Woodlands, TX, **2015**.
6) Sheldrick, G. M. SHELXT - Integrated space-group and crystal-structure determination. *Acta Cryst*. **2015**, *A71*, 3–8.
7) Sheldrick, G.M. A short history of SHELX. *Acta Cryst.* **2008**, *A64*, 112–122.
8) Müller, P. Practical suggestions for better crystal structures. *Crystallography Reviews* **2009**, *15*, 57–83.
9) Frisch, M.; Trucks, G.; Schlegel, H.; Scuseria, G.; Robb, M.; Cheeseman, J.; Scalmani, G.; Barone, V.; Mennucci, B.; Petersson, G.; Nakatsuji, H.; Caricato, M.; Li, X.; Hratchian, H.; Izmaylov, A.; Bloino, J.; Zheng, G.; Sonnenberg, J.; Hada, M.; Ehara, M.; Toyota, K.; Fukuda, R.; Hasegawa, J.; Ishida, M.; Nakajima, T.; Honda, Y.; Kitao, O.; Nakai, H.; Vreven, T.; Montgomery, J., JA; Peralta, J.; Ogliaro, F.; Bearpark, M.; Heyd, J.; Brothers, E.; Kudin, K.; Staroverov, V.; Keith, T.; Kobayashi, R.; Normand, J.; Raghavachari, K.; Rendell, A.; Burant, J.; Iyengar, S.; Tomasi, J.; Cossi, M.; Rega, N.; Millam, J.; Klene, M.; Knox, J.; Cross, J.; Bakken, V.; Adamo, C.; ramillo, J.; Gomperts, R.; Stratmann, R.; Yazyev, O.; Austin, A.; Cammi, R.; Pomelli, C.; Ochterski, J.; Martin, R.; Morokuma, K.; Zakrzewski, V.; Voth, G.; Salvador, P.; Dannenberg, J.; Dapprich, S.; Daniels, A.; Farkas, O.; Foresman, J.; Ortiz, J.; Cioslowski, J.; Fox, D. *Gaussian 09, Revision D.01*, *Gaussian, Inc.*: **2009**.
10) a) Zhao, Y.; Truhlar, D. G. The M06 Suite of Density Functionals for Main Group Thermochemistry, Thermochemical Kinetics, Noncovalent Interactions, Excited States, and Transition Elements: Two New Functionals and Systematic Testing of Four M06-Class Functionals and 12 Other Functionals. *Theor. Chem. Acc*. **2008**, *120*, 215; (b) Zhao, Y.; Truhlar, D. G. Density Functionals with Broad Applicability in Chemistry. *Acc. Chem. Res*. **2008**, *41*, 157.
11) Hehre, W. J.; Radom, L.; Schleyer, P. v. R.; Pople, J. A.; Ab Initio Molecular Orbital Theory, Wiley: New York, **1986**.
